# Stable and Broad Spectrum Cross-Protection Against Pepino Mosaic Virus Attained by Mixed Infection

**DOI:** 10.3389/fpls.2018.01810

**Published:** 2018-12-06

**Authors:** Jesús Agüero, Cristina Gómez-Aix, Raquel N. Sempere, Julio García-Villalba, Jorge García-Núñez, Yolanda Hernando, Miguel A. Aranda

**Affiliations:** ^1^R&D Department, Abiopep S.L., Murcia, Spain; ^2^Centro de Edafología y Biología Aplicada del Segura, Consejo Superior de Investigaciones Científicas (CSIC), Murcia, Spain

**Keywords:** acquired immunity, challenge infection, cross-immunization, PepMV, prophylactic inoculation, protective inoculation, superinfection exclusion, tomato

## Abstract

While recent pepino mosaic virus (PepMV; species *Pepino mosaic virus*, genus *Potexvirus*, family *Alphaflexiviridae*) epidemics seem to be predominantly caused by isolates of the CH2 strain, PepMV epidemics in intensive tomato crops in Spain are caused by both CH2 and EU isolates that co-circulate, representing a challenge in terms of control, including cross-protection. In this work, we hypothesized that mixed infections with two mild isolates of the EU and CH2 strains (PepMV-Sp13 and -PS5, respectively) may be useful in PepMV cross-protection in Spanish epidemics, providing protection against a broad range of aggressive isolates. Thus, we performed a range of field trials and an experimental evolution assay to determine the phenotypic and genetic stability of PepMV-Sp13 and -PS5 mixed infections, as well as their cross-protective efficiency. Our results showed that: (i) the phenotype of PepMV-Sp13 and -PS5 mixed infections was mild and did not change significantly when infecting different tomato cultivars or under different environmental conditions in Spain, (ii) PepMV-Sp13 and -PS5 mixed infections provided more efficient protection against two aggressive EU and CH2 isolates than single infections, and (iii) PepMV-Sp13 and -PS5, either in single or in mixed infections, were less variable than other two PepMV isolates occurring naturally in PepMV epidemics in Spain.

## Introduction

Pepino mosaic virus (PepMV; species *Pepino mosaic virus*, genus *Potexvirus*, family *Alphaflexiviridae*) is a widespread plant virus that causes a major disease in tomato crops worldwide (Hanssen and Thomma, 2010; Werkman and Sansford, 2010; Gómez et al., 2012a). Only in Europe, its presence has been described in 19 countries and is included in the EPPO A2 list of pests recommended for regulation as a quarantine pest (EPPO, 2016). The PepMV genome consists of a 6.4 kb single stranded RNA of positive polarity (+ssRNA) containing five open reading frames, including a replicase gene, a triple gene block (TGB) encoding TGB1, TGB2, and TGB3, involved in viral movement and silencing suppression, and a coat protein (CP) that has a structural as well as a silencing suppressor role and is necessary for viral movement (Aguilar et al., 2002; Mathioudakis et al., 2014; Agirrezabala et al., 2015). PepMV isolates can be classified into six strains based on molecular and biological characteristics: European strain (EU), North American strain (US1/CH1), Chilean strain (CH2), the recombinant strain (US2), the original Peruvian strain (LP), and the new Peruvian strain (PES) (Hanssen and Thomma, 2010; Moreno-Pérez et al., 2014). Isolates of the EU and CH2 strains appear to be most common (Pagán et al., 2006; Hanssen et al., 2008; Gómez et al., 2009; Davino et al., 2017); EU isolates initially spread in European tomato crops, whereas CH2 isolates spread epidemically later on, becoming predominant in most of the same areas (Gómez et al., 2009; Hanssen and Thomma, 2010). Isolates of the same strain share sequence identities varying between 95 and 100%, while isolates from different strains have sequence identities varying from 78 to 94%. The CH2 and EU are the most divergent strains, with a sequence identity of around 78% (Hanssen and Thomma, 2010; Moreno-Pérez et al., 2014). Genetic determinants of specific symptoms have been identified for PepMV. These include necrosis, for which it is known that an amino acid substitution in the TGB3 at position 67 (glutamic acid instead of lysine) is necessary though not sufficient for the virus to induce systemic necrosis (Hasiów-Jaroszewska et al., 2011; Hasiów-Jaroszewska and Borodynko, 2012; Sempere et al., 2016), and yellow bright mosaic, which has been associated to amino acid substitutions in positions 155 (lysine instead of glutamic acid) and 166 (glycine instead of aspartic acid) of the CP (Hasiów-Jaroszewska et al., 2013). In addition to the genetic viral determinants, there is a strong interaction between symptom expression, tomato cultivar and environmental conditions (see for example, Sempere et al., 2016).

PepMV control relies on the maintenance of strict hygiene measures, as the virus is transmitted very efficiently through mechanical means and there are no commercially available resistant tomato varieties. Cross-protection using attenuated isolates offers an interesting alternative. Cross-protection is a natural phenomenon in which infection with mild or attenuated virus strains protects plants against subsequent (or “challenge”) infections with more severe strains of the same virus (Hull, 2014). The phenomenon was first reported with tobacco mosaic virus (TMV) in 1929 (McKinney, 1929). Since then, cross-protection has been demonstrated for many plant viruses and used under commercial growing conditions in several occasions (reviewed in Ziebell and Carr, 2010). Mild strains for cross protection need to comply with a number of requirements (Fulton, 1986; Lecoq, 1998). For PepMV, mild isolates useful for cross protection have been identified (Hanssen et al., 2010; Schenk et al., 2010; Vermunt and Kaarsemaker, 2017) and in fact, cross-protection is being widely used in the Netherlands, Belgium and Morocco for the control of the PepMV-induced disease, and a commercial product based on a mild strain of PepMV (PMV-01) has been officially registered in several European countries^1^.

While recent PepMV epidemics seem to be predominantly caused by isolates of the CH2 strain, Spanish PepMV epidemics are caused by both CH2 and EU isolates that co-circulate (Pagán et al., 2006; Gómez et al., 2009, 2012a; our unpublished results). Interestingly, mixed infections with native Spanish PepMV-CH2 and PepMV-EU isolates result in symptom attenuation, a phenomenon that may be due to an asymmetric antagonistic interaction (Gómez et al., 2009, 2012b). This represents a challenge in terms of control, as measures based on genetic resistance or cross-protection need to provide efficient control for isolates of the two strains. Thus, we hypothesized that simultaneous infection of plants with isolates of the EU and CH2 strains may be useful in PepMV cross-protection in epidemics in Spain, providing protection against a broad range of aggressive PepMV isolates. However, the outcome of mixed infections might vary according to the genotype of the protected plant and environmental conditions; indeed, enhanced symptom display has been reported associated to infections by a PepMV-LP isolate challenged by subsequent inoculation with a PepMV-CH2 isolate (Hanssen et al., 2010). Thus, if PepMV mixed infections are going to be used in cross-protection, their outcome needs to be tested for a broad enough range of conditions and tomato cultivars. Also, a pre-requisite for the safe and efficient use of cross-protection is the genetic stability of mild isolates. Mutation and recombination are the major evolutionary forces in plant viruses that generate genetic variability. In mixed infections, mutations may arise within the production process of each mild isolate, but recombinant viruses may also arise among the two isolates with altered features compared to parental viruses (e.g., Miras et al., 2014). Recombination has been reported in many instances for plant viruses, including PepMV (Pagán et al., 2006; Hanssen et al., 2008), although recombinants seem to be infrequent in nature, at least for +ssRNA plant viruses (e.g., Fraile et al., 1997; Desbiez et al., 2011). As for mutation, a rate of molecular evolution of 5.570 × 10^-3^ substitutions/site/year has been reported for PepMV, a value that seems to be higher than rates reported for other plant RNA viruses (Gómez et al., 2012b). However, a recent report has shown that the PepMV genome has a surprisingly high robustness against mutations, and that fitness consequences for a given mutation depend on the strain considered (Minicka et al., 2017).

Thus, this study had a triple objective: first of all, to check the phenotypic outcome of mixed infections for a broad enough range of conditions and tomato cultivars. Second, to test if mixed infections could provide broader protection spectrum than single infections. And third, to assess the genetic stability of two PepMV mild native Spanish isolates, applied either in single or in mixed inoculations after passaging in tomato, and to compare their stability with that of two other naturally occurring aggressive PepMV isolates. Our results suggest that mixed infections with our mild native Spanish isolates could be safely used for cross-protection against a broad range of aggressive PepMV isolates, at least under the conditions of intensive tomato crops in Spain.

## Materials and Methods

### PepMV Isolates

PepMV-Sp13, a EU mild isolate, was sampled from tomato plants showing mild symptoms in commercial greenhouses in Murcia (Southeast Spain) in 2001 (Aguilar et al., 2002). Similarly, PepMV-PS5, a CH2 mild isolate, was sampled from a commercial tomato crop in Águilas, Murcia (Southeast Spain) in 2007 (Gómez et al., 2009). All PepMV isolates were kept as dried plant material and conserved at 4°C.

### Field Experiments

Three independent experiments with layouts as described below were conducted in three different greenhouses. The greenhouses were similar to those used for commercial tomato production in each area and had good confinement conditions and no assisted cooling or heating, apart from air circulation through the roof and lateral windows during the hottest hours of the day. All the windows were protected with insect-proof nets. Trials were carried out in accordance with the principles of good experimental practices as certified by an officially recognized organization. PepMV isolates were mechanically inoculated by rubbing the inoculum onto the carborundum-dusted, first fully expanded leaf of the seedlings grown on trays, before transplanting. PepMV isolates used for challenging were mechanically inoculated by rubbing the inoculum onto the carborundum-dusted leaves 20 days post inoculation (dpi) of the mild isolates. The inocula were prepared by grinding infected tomato plant material with 30 mM potassium phosphate buffer pH 8.0 in a 1:3 ratio.

*Experiment 1* was conducted at Experimental Station “Cajamar Las Palmerillas” (El Ejido, Almería, Southeast Spain) from September 2014 to May 2015. In this experiment, 3 tomato (*Solanum lycopersicum*) cultivars were used, cultivar (cv.) Caniles (Zeraim Iberica), cv. Ventero (Seminis) and cv. Angelle (Syngenta), all grafted onto cv. Multifort (De Ruiter). The trial encompassed 8 treatments for cv. Caniles and 4 for each of the other two cultivars (Table 1), as single pre-inoculations with isolates PepMV-Sp13 and PepMV-PS5 could not be included for these cultivars due to logistic limitations. Four replicates of 4 plants (cvs. Caniles and Ventero) or 8 plants (cv. Angelle) were used per treatment; plants were grown in hydroponic growing bags (Pelemix, Murcia, Spain) with two stems per plant.

**Table 1:** Treatments in field experiments for each tomato cultivar.

Treatment^∗^						
Preinoculation	Challenge 20 dpi	Ventero	Angelle	Caniles	Pitenza	Boludo
Untreated	–	X	X	X	X	X
Sp13 + PS5	–	X	X	X	X	X
Sp13	–			X	X	X
PS5	–			X	X	X
Untreated	KLP2	X	X	X	X	X
Sp13 + PS5	KLP2	X	X	X	X	X
Sp13	KLP2			X	X	X
PS5	KLP2			X	X	X
Untreated	H30				X	X
Sp13 + PS5	H30				X	X
Sp13	H30				X	X
PS5	H30				X	X

*^∗^Pre-inoculation of seedlings with single (Sp13, PepMV-Sp13; PS5, PepMV-PS5) or mixed mild isolates. Challenge with aggressive (KLP2, PepMV-KLP2; H30, PepMV-H30) isolates 20 days post pre-inoculation. Four replicates per treatment (Angelle, 8 plants per replicate; Pitenza and Boludo, 5 plants per replicate; Ventero and Caniles, 4 plants per replicate)*.

*Experiment 2* was carried out at Instituto de “Investigación y Formación Agraria y Pesquera” (IFAPA, Centro La Mojonera, Almería, Southeast Spain) using non-grafted tomato cv. Pitenza (Enza Zaden), from September 2016 to March 2017. The trial encompassed 12 treatments (Table 1), with 4 replicates per treatment and 5 plants per replicate in hydroponic growing bags and was performed in three separate greenhouse compartments with similar environmental conditions, one with unchallenged controls and the other two with the challenged treatments.

*Experiment 3* was carried out in a commercial greenhouse at El Albujón (Murcia, Southeast Spain) using non-grafted tomato cv. Boludo (Seminis), from November 2016 to May 2017. The trial encompassed 12 treatments (Table 1); in this case, plants grew directly on soil, with 4 replicates per treatment and 5 plants per replicate. Tomato yellow leaf curl virus-infected plants were detected at 60 dpi, therefore the observation period for this experiment was shortened as compared with the other two, and no statistically significant data on marketable production could be obtained for it (see section “Results”).

Experiments were visited at least twice a week, and observations and assessment of potential phytotoxicity effects, virus symptoms in vegetative parts of the plants, virus symptoms on fruits and production were made periodically. Virus infection was monitored by tissue print hybridization (see below) of leaf petiole cross sections 15 dpi of mild isolates and by observation of symptoms for the aggressive isolates. Virus diagnosis was also carried out at the end of the period to detect potential cross contaminations; control plants remained PepMV-free up to the end of the observation periods (data not shown). The assessment of yellowing symptoms was conducted according to an appropriate severity scale (see section “Results”). Assessment interval depended on the evolution of the symptoms. Assessments of the number and weight of fruits per replicate and per treatment were conducted differentiating between total production and marketable production, from the start of harvest until the 9th cluster of tomato fruits was harvested. All harvested fruits were classified according to the symptoms observed, differentiating PepMV symptoms of discoloration and necrosis.

### PepMV Detection and Quantification

For detection of PepMV in cross-protection experiments (see above) and during passaging in the experimental evolution assay (see below), we used molecular hybridization with digoxigenin (DIG)-labeled RNA specific probes (Más and Pallás, 1995) on tissue-prints of petiole cross-sections or dot-blots of total RNA extracts. RNA DIG-labeled probes were complementary to nucleotides 6152-6346 (PepMV-EU) and 6010-6343 (PepMV-CH2) and synthesized by transcription with T7 RNA polymerase (Promega Corporation, Madison, WI, United States) from pGEM-T Easy vectors (Promega Corporation) with the corresponding cDNA inserts. Prehybridization and hybridization of membranes and virus detection were performed as described by Marco et al. (2003).

Accumulation of viral RNA of isolates PepMV-Sp13 and -PS5 in the 4 replicates of cv. Caniles plants (field experiment 1) at 43, 68, and 123 days post inoculation was measured by real time one-step quantitative RT-PCR. Leaves from 4 plants of each replicate were pooled and total RNA was extracted using the Nucleo-Spin^®^ RNA plant kit (Macherey-Nagel GmbH, Düren, Germany) according to the manufacturer’s instructions. Tenfold serial dilutions of purified viral RNA transcripts of known concentration were used to generate external standard curves. The real time RT-PCR was conducted in a final volume of 20 μL with KAPA SYBR^®^ FAST Universal One-Step qRT-PCR Kit (KAPA Biosystems, United States) using 2 μL/reaction of each RNA transcript dilution or plant total RNA (30 ng), according to the manufacturer’s instructions. Specific primer pairs for each isolate were used (Gómez et al., 2009), and three technical replicates per sample or dilution were included in each plate. The reaction was carried out in a StepOnePlus (Applied Biosystems, United States) apparatus. The amount of PepMV RNA per 100 ng of total RNA was calculated from the mean number of copies of PepMV genome of the four replicates as determined from the quantification and the molecular weight of PepMV genome (2.06 ^∗^ 10^6^g/mol).

### Experimental Evolution Assay

Three independent lineages were set up per isolate (PepMV-Sp13, -PS5, -H30, and -KLP2), plus three additional lineages corresponding to the mixed inoculation of PepMV-Sp13 + -PS5. Founder plants were set up after reviving the PepMV isolates in tomato plants (cv. Moneymaker). The virus population from the founder plants was sampled, pooled, and used to determine the ancestral sequences. Up to eight passages were carried out on cv. Moneymaker plants with a periodicity of 12–14 days over a period of 104 days. For each passage, PepMV isolates were mechanically inoculated onto seedlings 14 days after sowing by rubbing the inoculum onto the carborundum-dusted first fully expanded leaf. Approximately 20 μL of inoculum per leaf were used, and the inoculum was prepared by grinding 50 mg of dried infected tissue in 2 mL of 30 mM potassium phosphate buffer pH 8.0. Inoculated plants were physically separated enough to avoid cross-contaminations. To set up founder plants of the mixed infection treatment (PepMV-Sp13 + -PS5), 200 mg of apical leaves from plants infected with each of these isolates were collected and ground together in 4 mL of 30 mM potassium phosphate buffer pH 8.0, and the homogenate was used as inoculum. After passage zero, the treatment of mixed infection (PepMV-Sp13 + -PS5) was handled as the other treatments. PepMV infection was checked 2 days before sampling by hybridization in tissue-prints using strain-specific probes able to discriminate between EU and CH2 isolates (see above). Symptom severity was annotated during the entire period of study. After passage 8, two 500-mg samples were taken from apical leaves from each plant 12 days after inoculation; one of the samples was used for RNA extraction and sequencing (see below) and the other was stored at -80°C. Inoculated plants were grown in 1.1 L pots and kept in an insect-proof glasshouse at 24–26°C day, 16–18°C night, with 16 h photoperiod.

#### RNA Extraction, RT-PCR and cDNA Cloning

Plant material was collected from the three founder plants separately (replicates) and after the final passage of the experimental evolution assay, and total RNA was extracted using the Nucleo-Spin^®^ RNA plant kit (Macherey-Nagel GmbH), according to the manufacturer’s instructions, and dissolved in 30 μL of sterile water. After the extraction, the RNA was analyzed by dot-blot and molecular hybridization to check the presence of the virus in the infected samples. All samples were subjected to RT-PCR using the primers described by Gómez et al. (2009). First strand cDNAs were synthesized using 1 μg × μL^-1^ of RNA, oligo (dT) primer (500 nM) and the Expand reverse transcriptase (Roche Diagnostics) according to the manufacturer’s protocol. A 2200 nt region containing the complete TGB and CP genes was amplified by PCR using 200 ng of cDNA and the Expand High Fidelity PCR system (Roche Diagnostics) according to the manufacturer’s protocol. The region sequenced included the genetic determinants responsible for necrosis (in TGB3) and bright yellow mosaics (in CP). The PCR products were separated on a 0.7% agarose gel and were purified using Geneclean turbo kit (MP Biomedicals, United States) following the manufacturer’s instructions. All products were ligated into the pGEM-T Easy vector (Promega Corporation) and plasmids were transformed into *Escherichia coli* Stellar^TM^ competent cells (Clontech Laboratories, United States). Plasmid DNA was isolated and digested with *Eco*RI restriction enzyme to verify cloning efficiency.

#### Sequencing of cDNA Clones, Alignment of Sequences and Population Genetics Analysis

All cDNA clones were submitted for sequencing to Secugen (Madrid, Spain) using universal primers M13F and M13R and two internal primers, as in Gómez et al. (2009). Four sequences were obtained from each clone, which were checked using BioEdit (Hall, 1999) and assembled into full-length contigs using Geneious 10.1.3^2^ (Kearse et al., 2012). Given the difficulty associated to the analysis of overlapping genes, such as those encoding TGB proteins (see below), all analyses were carried out on independent ORF sequences or on concatenated ORF sequences, bypassing overlapping. Multiple sequence alignments were generated by using CLUSTAL W in MEGA7 (Kumar et al., 2016). Recombination analyses were performed using RDP4 (Martin et al., 2015) and by visual inspection of alignments of informative nucleotide positions. All other evolutionary and population genetics analyses of nucleotide sequences were performed using MEGA7 (Kumar et al., 2016). Nucleotide diversity (π) was estimated using the Kimura 2-parameter (K2P) model and was expressed as the average number of nucleotide substitutions per site between each pair of sequences.

## Results

### Phenotypic Stability of PepMV-Sp13 and -PS5 Mixed Infections in Field Experiments

Phenotypic stability of mixed PepMV-Sp13 and -PS5 infections was checked in the three field experiments described in the Materials and Methods section. The effects on tomato plants were monitored for mixed infections in comparison to single infections and uninfected controls. PepMV-KLP2, a CH2-type isolate, was used as reference for the effects caused by an aggressive isolate. PepMV-KLP2 was sampled from tomato plants showing fruit necrosis, bright yellowing and vein banding in leaves in a commercial tomato crop in Granada (Spain) in 2014, and induces severe symptoms in *Nicotiana benthamiana* plants including marked mosaic, occasional vein necrosis, chlorosis and leaf distortion (data not shown), and bright yellow mosaic, vein banding and growth reduction in tomato (see below).

**FIGURE 1:**
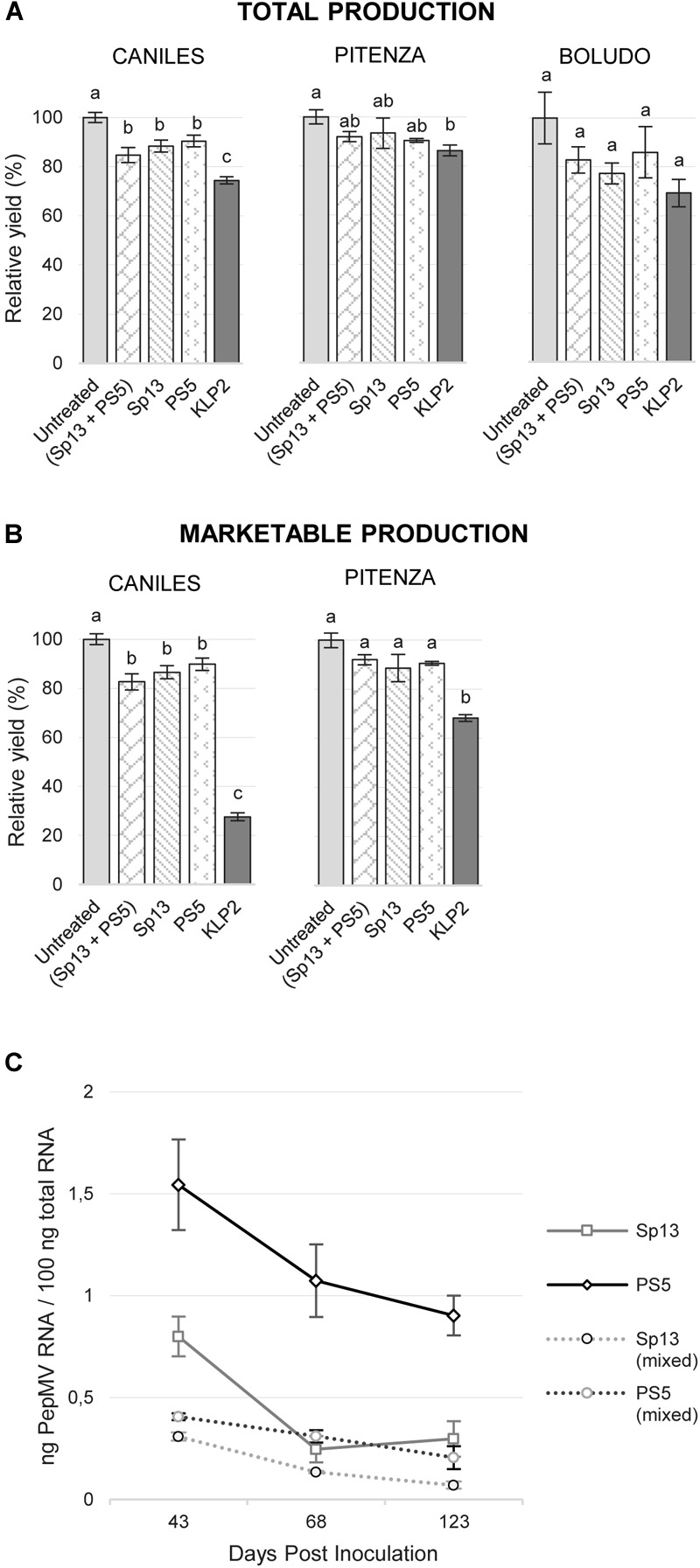
Mean total production **(A)** and marketable production **(B)** per treatment (infection with isolates PepMV-Sp13, -PS5, -SP13 + -PS5 or -KLP2), relative to the untreated control, for three tomato cultivars, Caniles, Pitenza, and Boludo. No statistically significant data on marketable production could be obtained for cv. Boludo. Treatments with no letters in common are significantly different, LSD test (*P* ≤ 0,05), *n* = 4. Bars represent standard error of the mean. **(C)** Viral RNA accumulation of PepMV-Sp13 and -PS5 in single and mixed infections in tomato plants, cv. Caniles, at different dates after inoculation. Mean ± SE, *n* = 4.

Aggressive PepMV-KLP2 symptoms appeared as soon as 6 dpi, while no obvious symptoms could be observed on the vegetative parts of the plants for any of the treatments including PepMV-Sp13 and -PS5 infections, apart from occasional and rather inconspicuous leaf narrowing or bubbling of the leaflets’ laminae. We thus focused on fruit production, using the results from the uninfected control as the reference (Figure 1). With regard to total fruit production, PepMV infection caused significant reductions, which averaged 15.5% for cv. Caniles and 9.5% for cv. Pitenza. For cv. Boludo, tendencies were similar, though the dispersion of measurements prevented the identification of statistically significant differences. There were no significant differences in total production among treatments involving PepMV-Sp13 or -PS5 in either single or mixed infections, and reduction in total production associated to these isolates averaged 12.1 and 8.1% for cvs. Caniles and Pitenza, respectively. PepMV-KLP2 caused significantly more important reductions which averaged 25.6 and 13.7% for cvs. Caniles and Pitenza, respectively (Figure 1A). Marketable fruit production was drastically reduced for cv. Caniles plants infected with PepMV-KLP2, and significantly reduced for cv. Pitenza plants infected with the same isolate. Symptoms on fruits included the characteristic uneven distribution of pigments usually associated to PepMV infection, but also necrosis, reduction of the fruit size and malformations for plants infected with PepMV-KLP2 (Supplementary Figure S1). Importantly, marketable production was not significantly reduced for cv. Pitenza plants either singly or mixed infected with PepMV-Sp13 and -PS5, and no significant differences were observed among treatments of cv. Caniles plants singly or mixed infected with PepMV-Sp13 and -PS5 (Figure 1B).

We have described a drastic reduction of PepMV-PS5 accumulation in plants mixed-infected with PepMV-Sp13 as compared to single-infected plants (Gómez et al., 2009). To determine if this effect lasted for longer periods than those originally tested (32 dpi) and in a tomato cultivar that was different to the one originally used (cv. Boludo), we measured the absolute accumulation of PepMV-Sp13 and -PS5 at 43, 68, and 123 dpi in single and mixed infected plants in experiment 1 (cv. Caniles). On average, the accumulation of PepMV-PS5 (the CH2-type isolate) more than doubled that of PepMV-Sp13 (the EU-type isolate) in single infections, but this was reversed in mixed infections (Figure 1C), confirming our previous observations (Gómez et al., 2009).

### Range of Cross-Protection Provided by PepMV-Sp13 and -PS5 Mixed Infections

**FIGURE 2:**
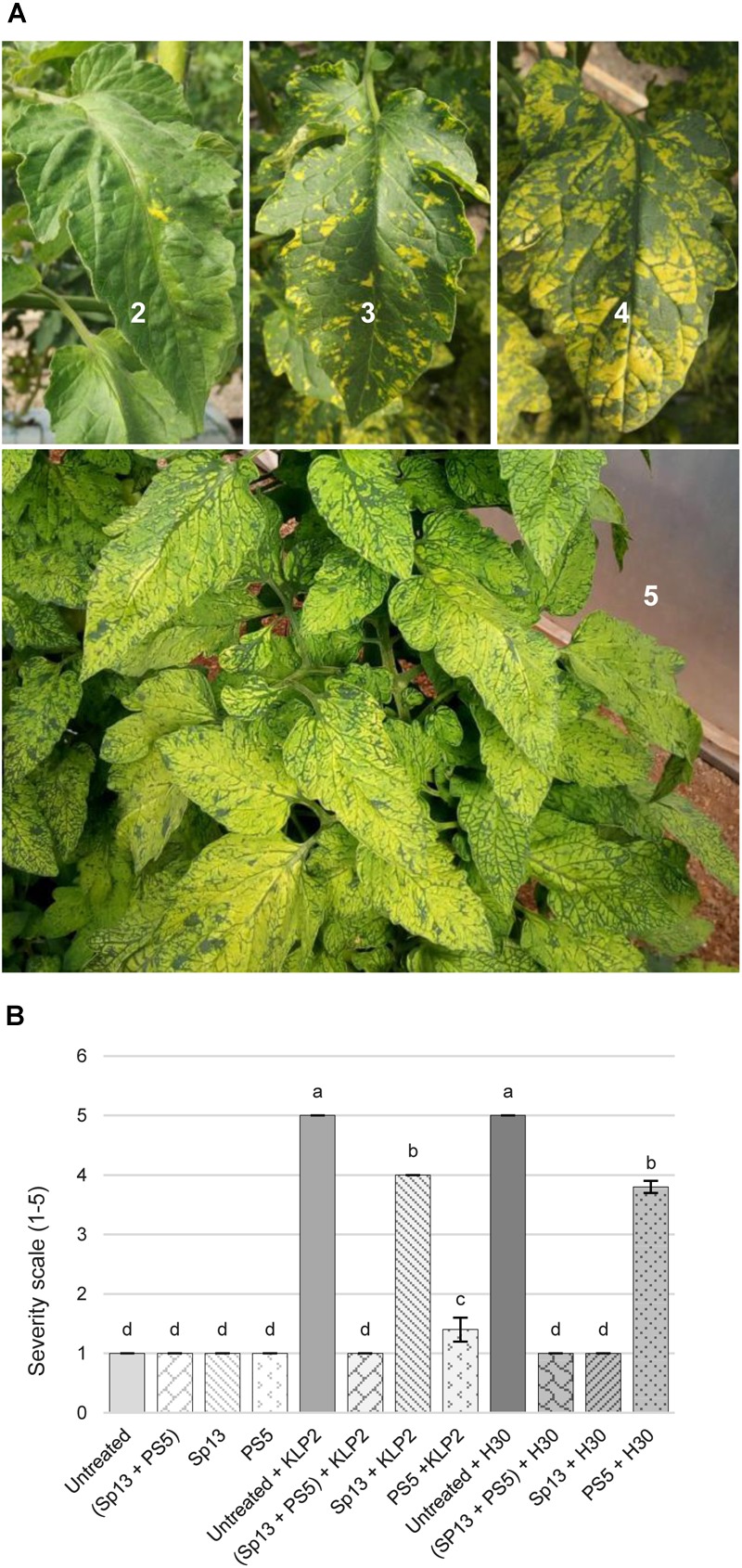
Cross-protection against aggressive PepMV isolates that induce yellow bright mosaic in leaves. **(A)** Yellow bright mosaic severity scale on leaves: 1, no symptoms; 2, occasional isolated spots; 3, spots along the leaflet lamina in 10–30% of the leaves; 4, coalescent spots in 30–60% of the leaves; 5, yellowing in more than 60% of the leaves. **(B)** Mean severity of bright yellow mosaic symptoms 182 days after pre-inoculation (infection with PepMV-Sp13, -PS5 or -SP13 + -PS5) and 158 days after challenge with an aggressive CH2-type isolate, PepMV-KLP2, or an aggressive EU-type isolate, PepMV-H30, in cv. Pitenza plants. Non-pre-inoculated controls (untreated) were used as reference. Treatments with no letters in common are significantly different, LSD test (*P* ≤ 0,05), *n* = 4. Bars represent standard error of the mean.

**FIGURE 3:**
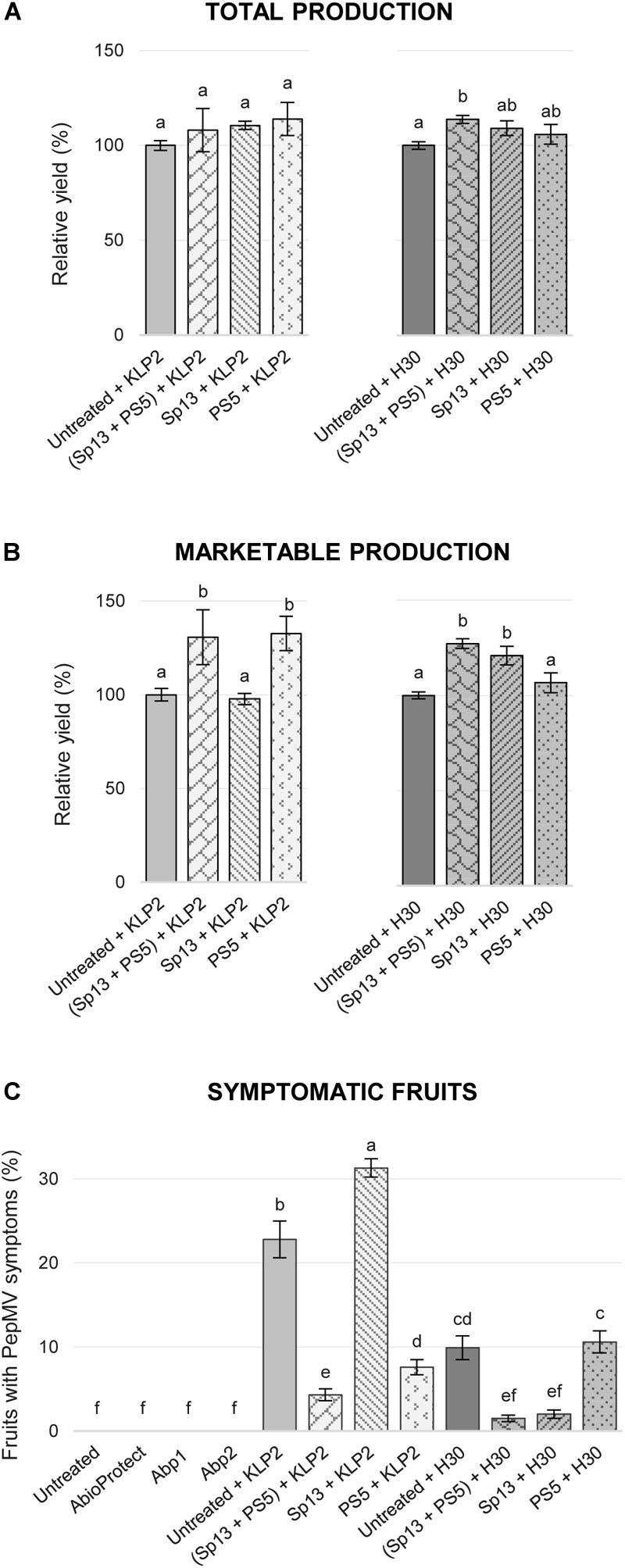
Mean total production **(A)** and marketable production **(B)** in tomato cv. Pitenza for treatments (untreated, infection with isolates PepMV-Sp13, -PS5, -SP13 + -PS5) challenged with an aggressive CH2-type isolate (PepMV-KLP2) or an aggressive EU-type isolate (PepMV-H30), represented as percentage relative to the untreated control. **(C)** Mean of PepMV fruits with symptoms per treatment (%). Treatments with no letters in common are significantly different, LSD test (*P* ≤ 0,05), *n* = 4. Bars represent standard error of the mean.

This was studied in field experiment 2 (see section “Materials and Methods”). Thus, to test if PepMV-Sp13 and -PS5 mixed infections could broaden the cross-protection range provided by single infections, pre-infected plants (as described above) were challenged with either PepMV-KLP2 (see above) or PepMV-H30, an aggressive EU-type isolate. PepMV-H30 was sampled from tomato plants showing bright yellowing and vein banding in leaves from a commercial tomato crop in Alicante (Spain) in 2015, and induces aggressive symptoms of infection in *N. benthamiana* plants including marked mosaic, bright yellow mosaic and leaf distortion (data not shown), and bright yellow mosaic, vein banding and growth reduction in tomato as early as 6 dpi (Figure 2A). Infections with PepMV-KLP2 or PepMV-H30 result in yellow bright mosaics in leaves, providing a visual and very efficient disease severity marker. Hence, the severity of yellowing symptoms was assessed periodically up to 158 days after the challenge. For simplicity, challenging with an aggressive isolate of the same strain will be referred to as homologous combination, and heterologous when challenging with an aggressive isolate of the different strain. By day 14 after the challenge, non-pre-inoculated and challenged controls already scored maximum severity levels. Plants pre-inoculated and challenged in heterologous combinations showed clear disease symptoms, which were evident all throughout the observation period. This was in contrast with plants mixed-pre-inoculated, which showed no yellowing symptoms all throughout the observation period, similar to plants pre-inoculated and challenged in homologous combinations, plants only pre-inoculated with the mild isolates and uninfected plants (Figure 2B). In the same experiment, total fruit production, marketable production and the proportion of fruits with PepMV symptoms for each treatment (Figure 3) was measured. Challenging with PepMV-KLP2 had no statistically significant effects on total production for any pre-inoculated treatments, while challenging with PepMV-H30 resulted in significant increases of total production for treatments that had been either mixed or singly pre-inoculated (Figure 3A). Differences in marketable production were remarkable. In treatments mixed-pre-inoculated or singly pre-inoculated and challenged with the homologous virus, significantly higher yields were obtained; the increase in yield ranged from 21 to 33%. In contrast, treatments singly inoculated and challenged with the heterologous virus had similar yields as non-pre-inoculated and challenged plants (Figure 3B). These effects resulted mainly from discarding fruits with PepMV symptoms (Figure 3C). Challenge infection with either PepMV-KLP2 or PepMV-H30 had a strong and significant effect on the proportion of fruits with symptoms for the singly-pre-inoculated with the heterologous viruses PepMV-Sp13 or PepMV-PS5 treatments, respectively, which were higher or similar than those for the non-pre-inoculated controls (Figure 3C). Importantly, mixed-pre-inoculated plants produced a very small proportion of symptomatic fruits when challenged with any of the aggressive isolates, thus protecting from yield losses that ranged between 10 and more than 30% (Figure 3C).

**FIGURE 4:**
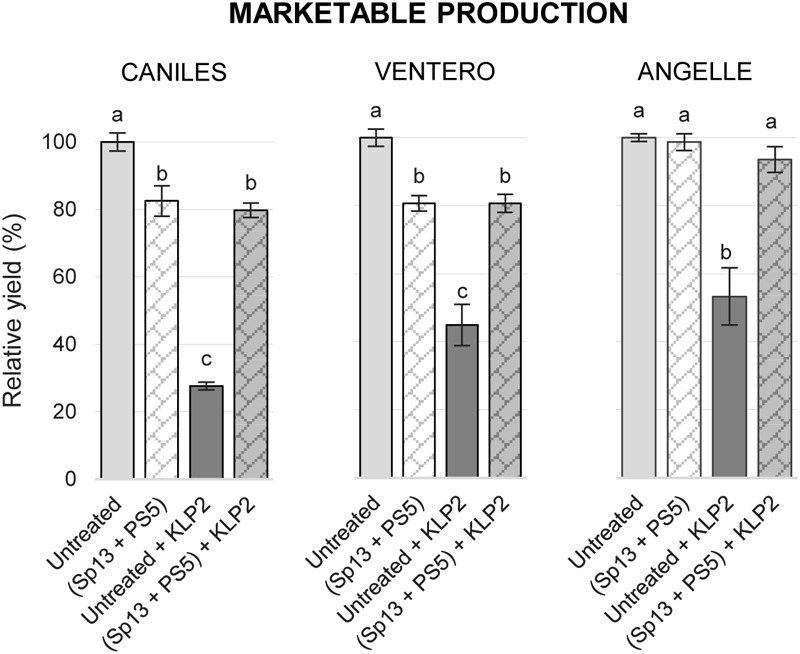
Mean marketable production for non-pre-inoculated or pre-inoculated with PepMV-Sp13 + -PS5, challenged with an aggressive CH2-type isolate, PepMV-KLP2, in 3 tomato cultivars, Caniles, Ventero, and Angelle, compared with the unchallenged mixed infection (PepMV-Sp13 + -PS5). Production is represented relative to the untreated control. Data of untreated and PepMV-Sp13 + -PS5 mixed pre-inoculation of cv. Caniles have already been shown in Figure 1A, but are included here for clarity. Treatments with no letters in common are significantly different, LSD test (*P* ≤ 0,05), *n* = 4. Bars represent standard error of the mean.

The cross-protective effect of PepMV-Sp13 and -PS5 mixed infections was also tested in experiment 1. In this case, PepMV-KLP2 was the only virus used to challenge plants, and two additional tomato cultivars were included in the experiment, cvs. Ventero and Angelle. Due to logistic limitations, for these cultivars, no single PepMV-Sp13 or -PS5 pre-inoculations were assayed. For cv. Caniles, very efficient cross-protection was achieved, which resulted in trice the yield of the untreated and challenged control. For cvs. Ventero and Angelle, mixed pre-inoculation resulted in almost doubling the yield of the untreated and challenged control (Figure 4). In conclusion, PepMV-Sp13 and -PS5 mixed pre-inoculations provided efficient cross-protection against aggressive isolates of the two strains, broadening the cross-protective range conferred by single pre-inoculations.

### Experimental Evolution of PepMV-Sp13 and -PS5 in Single and Mixed Infections

**FIGURE 5:**
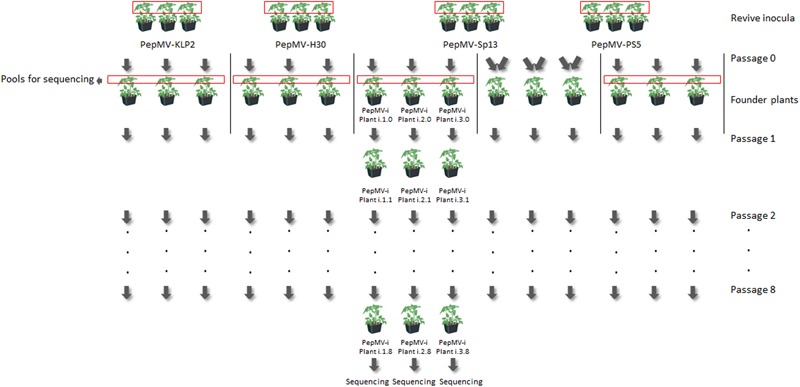
Diagram illustrating the setting up of founder plants and passaging of lineages for the experimental evolution study. To set up founder plants of the mixed infection treatment (PepMV-Sp13 + -PS5), apical leaves from plants infected with each of these isolates were collected, ground together and the homogenate was used as inoculum. After passage 0, the mixed infection treatment (PepMV-SP13 + -PS5) was handled as the other treatments. Viral populations were passaged eight times every 10–12 days to virus-free tomato plants (cv. Moneymaker). Virus populations from founder plants were sampled, pooled, and sequenced to determine ancestral sequences. After passage 8, each independent lineage was sequenced.

To assess the genetic stability of PepMV-Sp13 and -PS5 either in single or mixed infections, and to compare their stability with that of PepMV-H30 and -KLP2, which are two naturally occurring aggressive PepMV isolates, the evolution of the viruses was forced in a passaging experiment and the genetic diversity of their progenies was analyzed. Thus, three lineages were established per isolate and for the PepMV-Sp13 + PS5 mixed infection, and eight passages were carried out per lineage (Figure 5). PepMV infection was checked after passages 1, 3, 4, 5, 7, and 8 two days before sampling by hybridization in tissue-prints using strain-specific probes. No cross-contaminations were detected at any time. However, after passage 5, lineage 3 of treatment PepMV-KLP2 was lost, as no hybridization signal was observed with any of the two probes used (data not shown).

Symptoms in individual plants were recorded the day of material collection for the next passage according to the following 0–3 scale: 0, asymptomatic infection; 1, leaf bubbling, mild green mosaic; 2, leaf bubbling and non-generalized though severe yellow bright mosaic; 3, severe and generalized yellow bright mosaic and obvious growth reduction. No conspicuous symptoms were observed at any time for treatments PepMV-Sp13 and -Sp13 + -PS5. For treatment PepMV-PS5, leaf bubbling was observed sporadically, normally in only one of the three lineages. During initial passages, symptoms for treatments PepMV-H30 and -KLP2 were similarly severe, with all the plants from every lineage showing generalized yellow bright mosaics and growth reduction. However, significant differences appeared between these two treatments along passages; while for treatment PepMV-H30 these symptoms persisted up to passage 5, for treatment PepMV-KLP2 symptom severity decayed rapidly, with plants showing mild symptoms (leaf bubbling, mild green mosaic) or even no symptoms at all after passage 3 (Supplementary Figure S2).

**FIGURE 6:**
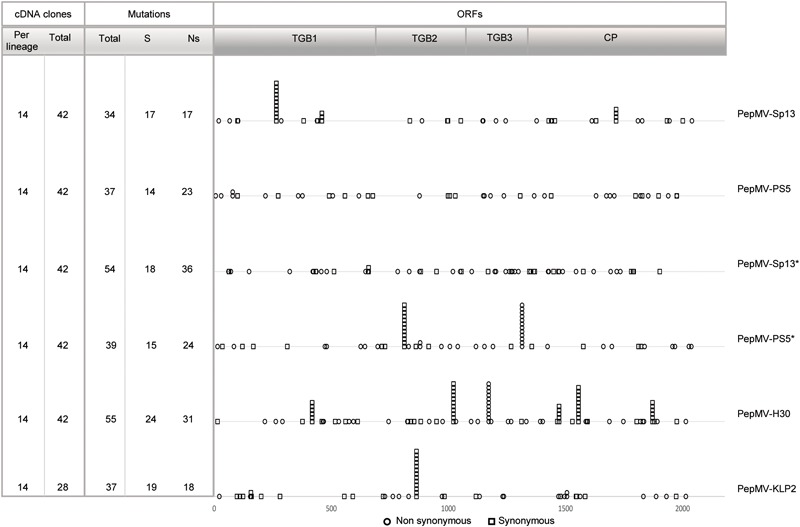
Counting and mapping mutations identified after passaging against ancestral sequences. On the left-hand side, the number of cDNA clones sequenced per lineage and the total of cDNA clones sequenced per treatment are indicated. The number of mutations ranged from 34 for PepMV-Sp13 passages, to 55 for PepMV-H30 passages. Mutations appearing in more than three clones were identified for 12 nucleotide positions. Non-synonymous vs. synonymous mutations were distinguished; ratios ranged from 17/17 (1.0) for PepMV-Sp13 passaging to 36/18 (2.0) for passaging of the same virus in mixed infection (PepMV-Sp13^∗^ and PepMV-PS5^∗^).

A region of the PepMV genome spanning approximately 2200 nucleotides was targeted for sequencing; this included the genes encoding proteins TGB1, TGB2, TGB3, and CP (Aguilar et al., 2002). To determine ancestral sequences, sixteen clones were sequenced from pooled samples from each of the treatments of founder plants; these sequences were aligned and the consensus sequence from each alignment was used as the ancestor for each treatment. After passaging, 238 full-length good quality sequences could be determined, corresponding to 14 to 16 clones per lineage, totalizing 28–42 clones per treatment (Figure 6). It is important to mention that the cDNAs were obtained from a single RT-PCR reaction, allowing the estimation of the frequency of recombination events along the full-length sequence. That was critical for treatment PepMV-Sp13 + -PS5, where mixed infections could give rise to recombinant viruses. After analyzing all sequences obtained for such treatment, no recombinant viruses were identified.

Next, we analyzed the nature of the mutations arising along passages and the resulting population diversity. We first searched for mutations at positions that have been shown to be genetic determinants of necrosis or bright yellow mosaic (Hasiów-Jaroszewska et al., 2011, 2013; Hasiów-Jaroszewska and Borodynko, 2012; Sempere et al., 2016). No necrosis-determining substitutions in TGB3 or yellowing substitutions in codon 166 of CP were observed for any PepMV isolate. However, the substitution of amino acid 155 (lysine vs. glutamic acid) of the PepMV-KLP2 CP was maintained in all clones after passaging, even if symptom expression was attenuated after passage 3, suggesting that the presence of this substitution was not the sole determinant of yellowing. Secondly, we counted and mapped all mutations identified after passaging against the ancestral sequences (Figure 6 and Supplementary File S1). The number of mutations ranged from 34 for PepMV-Sp13 passages, to 55 for PepMV-H30 passages. Mutations appearing in more than three clones were identified for 12 nucleotide positions, suggesting the fixation of these mutations along passaging. No such mutations were identified for inoculations with PepMV-PS5 in single infection, or for PepMV-Sp13 in mixed infection, suggesting that in the passages of these two viruses no mutation fixation occurred. A high ratio of non-synonymous to synonymous mutations was observed, which ranged from 17/17 (1.0) for PepMV-Sp13 passaging to 36/18 (2.0) for passaging of the same virus in mixed infection (Figure 6).

Thirdly, we estimated the genetic diversity of viral populations using Nei’s nucleotide diversity index (π) (Nei and Li, 1979). We estimated π for the whole sequence of passaged populations (Table 2) and also for each gene separately (Table 2). Nucleotide diversity values were low, and ranged from 0.0012 ± 0.0003 for PepMV-Sp13 and PS5 in single infections, to 0.0022 ± 0.0005 for PepMV-H30 (Table 2). PepMV-Sp13 and -PS5 passaged populations, either in single or in mixed infections, had lower nucleotide diversity values than those of aggressive isolates PepMV-H30 or -KLP2 (Table 2). Correspondingly, higher nucleotide diversity values were observed for passaged populations of these two isolates when separately considering the four coding regions, with the ORFs encoding PepMV-H30 TGB3 and PepMV-KLP2 TGB2 being the more variable regions, with nucleotide diversity values of 0.0027 ± 0.0016 and 0.0027 ± 0.0014, respectively (Table 2).

**Table 2:** Nucleotide diversity values (π) for passaged populations for each gene and for the concatenated sequences.

	Concatenated sequence	TGB1	TGB2	TGB3	CP
Sp13	0.0012 ± 0.0003	0.0015 ± 0.0007	0.0006 ± 0.0003	0.0008 ± 0.0004	0.0012 ± 0.0003
PS5	0.0012 ± 0.0003	0.0011 ± 0.0003	0.0005 ± 0.0002	0.0010 ± 0.0004	0.0017 ± 0.0008
Sp13^∗^	0.0013 ± 0.0002	0.0011 ± 0.0003	0.0010 ± 0.0004	0.0019 ± 0.0006	0.0015 ± 0.0003
PS5^∗^	0.0013 ± 0.0003	0.0007 ± 0.0002	0.0024 ± 0.0012	0.0026 ± 0.0017	0.0009 ± 0.0002
H30	0.0022 ± 0.0005	0.0017 ± 0.0005	0.0024 ± 0.0011	0.0027 ± 0.0016	0.0026 ± 0.0008
KLP2	0.0016 ± 0.0003	0.0014 ± 0.0004	0.0027 ± 0.0014	0.0012 ± 0.0006	0.0014 ± 0.0004

*Sp13^∗^ and PS5^∗^ correspond to PepMV-Sp13 and PepMV-PS5 from mixed infections. Nucleotide diversity (π) was estimated with MEGA7 using the K2P model and was expressed as the average number of nucleotide substitutions per site between each pair of sequences. Standard error estimates are shown and were calculated using 1000 bootstrap replicates.*

## Discussion

Cross-protection was first reported with TMV in 1929 (McKinney, 1929). Since then, cross-protection has been demonstrated for many plant viruses and used under commercial growing conditions in several occasions, including for the control of zucchini yellow mosaic virus in cucurbits, papaya ringspot virus in papaya and citrus tristeza virus in citrus trees (reviewed in Ziebell and Carr, 2010). Requirements for field application of mild virus strains for cross-protection were proposed by Fulton (1986) and Lecoq (1998) and recently reviewed by Ziebell and Carr (2010). In the following lines we will discuss, under the light of the results presented in this article, whether mixed infections with PepMV-Sp13 and -PS5 can be used in cross-protection in compliance with these requirements.

First of all, a protective strain needs to be able to systemically infect plants of the target crop inducing mild symptoms, but it should not significantly affect crop quality and yield. Both PepMV-Sp13 and -PS5 are native isolates sampled from systemically infected field tomato plants; they have been characterized in depth (Aguilar et al., 2002; Gómez et al., 2009; Sempere et al., 2011, 2016) and can systemically infect tomato plants. Their host range is rather narrow and essentially restricted to a subset of species in the family *Solanaceae* (Aguilar et al., 2002; Gómez et al., 2009; Sempere et al., 2016); these are two PepMV generic features favorable for cross-protection. Results presented here showed that both isolates, either in single or in mixed infections, induce rather inconspicuous symptoms in plants of diverse tomato cultivars, and that the phenotype of the infection was stable and independent of the cultivar and the environmental conditions tested. Contrasting with our former (Gómez et al., 2009) and current (Figure 1C) results on virus accumulation in mixed infected plants, where an asymmetric antagonistic interaction could be identified in association with better growth of mixed infected plants (Gómez et al., 2009), we did not find any significant differences in total fruit production or marketable production between PepMV-Sp13 and -PS5 single and mixed infections; any or a subset of the myriad of factors that influence fruit production could be buffering this effect. Infection of tomato plants with PepMV-Sp13 and/or -PS5 resulted in small though significant reductions of the total fruit production for at least two of the three cultivars tested, which contrasts with earlier findings in greenhouse tomato production in the United Kingdom, where no effect on total production was found for PepMV infections (Spence et al., 2006); this could be attributed to the very different growing conditions in the two sites, perhaps being more favorable for symptomatic PepMV infections in the Spanish crops, but other factors cannot be ruled out. Importantly, as regards to cross-protection, the mild isolates tested here did not have any effects on marketable production in the cv. Pitenza, although they again had a small though significant effect on cv. Caniles; however, this effect was rather negligible compared to the huge losses that the aggressive PepMV-KLP2 isolate caused, which in our experiments were approximately 72% of the marketable production for cv. Caniles (Figure 1B). In our experience, the frequency of very aggressive PepMV outbreaks is not too high in Spain, although losses in commercial crops often range between 20 and 40% of the marketable production at least in the Murcia region (Spain). Thus, losses caused by PepMV-Sp13 and/or -PS5 are in the same range than those reported for other mild PepMV strains (Hanssen et al., 2010; Schenk et al., 2010; Vermunt and Kaarsemaker, 2017), and therefore, it seems that PepMV-Sp13 and -PS5, in single or mixed infections, are in this respect at least as good as any other PepMV mild isolate reported in the literature.

Cross-protection has to be effective against a broad enough range of viral isolates and has to avoid the propensity of breaking down (Fulton, 1986; Lecoq et al., 1991). Cross protection mechanisms are still far from well know (Gal-On and Shiboleth, 2006; Ziebell and Carr, 2010; Zhang et al., 2017) but, whatever the mechanisms, it is widely accepted that cross-protection functions in a homology-dependent manner. Therefore, the simultaneous use of two isolates from two well-differentiated PepMV strains could theoretically broaden the cross-protection spectrum. Indeed, results presented in this report fully support the theoretical predictions, as tomato plants mixed-infected with PepMV-Sp13 and -PS5 were protected against aggressive isolates from both the EU and CH2 strains, whereas singly infected plants were only fully protected against the homologous aggressive isolate but partially protected against the heterologous one (Figures 2, 3). Our unpublished results suggest that challenging isolates are excluded from super infection; in this respect, we believe that PepMV constitutes a very interesting experimental system for the study of the underlying mechanisms and research in this direction should be conducted. Also, our findings on the protection spectrum associated to PepMV-Sp13 and -PS5 mixed infections have important practical implications, particularly in an epidemiological context such as the Spanish one, where it is well documented that CH2 and EU isolates co-circulate during epidemics (Pagán et al., 2006; Gómez et al., 2009, 2012a; our unpublished results). In this regard, perhaps the most important requirement that cross-protective mixed infections need to fulfill is the mild isolates’ genetic stability; quoting Ziebell and Carr (2010) “the protective strain needs to be genetically stable within the plant so that it does not mutate into a severe strain” (Ziebell and Carr, 2010). Our experimental evolution analyses allowed us to conclude that this indeed seems to be the case. The following conclusions could be drawn from these analyses. (i) No conspicuous symptoms were observed at any time for infections with PepMV-Sp13 and PepMV-Sp13 + PepMV-PS5. For infections with PepMV-PS5, leaf bubbling was observed sporadically but no change in aggressiveness was fixed along passages. Contrastingly, symptoms for infections with PepMV-H30 and PepMV-KLP2 were similarly severe during initial passages, with all plants from every lineage showing generalized yellow bright mosaics and growth reduction. However, symptom severity decayed for these aggressive isolates between passages 3 and 5; sequencing of progeny viruses (see below) did not provide clear clues on the genetic determinism of yellowing symptoms, as amino acid substitutions that have been shown to determine yellowing were retained in progeny viruses that lost their yellowing-induction capability. (ii) After passaging all four isolates and the PepMV-Sp13 + PepMV-PS5 mixed infection, we sequenced and analyzed 238 full-length cDNA sequences spanning approximately 2200 nt of the PepMV genome, which is a good representation of the entire PepMV genome. Importantly, cDNAs were obtained out of a single RT-PCR reaction, allowing for the estimation of the frequency of recombination events along the full-length sequence. That was critical for PepMV-Sp13 + PepMV-PS5, where mixed infections could give rise to recombinant viruses. After analyzing all sequences obtained for PepMV-Sp13 + PepMV-PS5 lineages, it could be concluded that no recombinant viruses were identified. (iii) The amino acid substitutions that have been described as being responsible for necrosis and bright yellow mosaic induction (Hasiów-Jaroszewska et al., 2011, 2013; Hasiów-Jaroszewska and Borodynko, 2012; Sempere et al., 2016) did not occur after passaging PepMV-Sp13, PepMV-PS5, or PepMV-Sp13 + PepMV-PS5. (iv) Nucleotide diversity values of passaged virus populations were low, and ranged from 0.0012 ± 0.0003 for PepMV-Sp13 and PS5 in single infections, to 0.0022 ± 0.0005 for PepMV-H30. A surprising aspect was the high ratio of non-synonymous to synonymous mutations, which ranged from 17/17 (1.0) for passaging of PepMV-Sp13 to 36/18 (2.0) for passaging of the same virus in mixed infection; we do not have an explanation for this phenomenon, but it is in agreement with previous reports on the experimental evolution of PepMV after passaging (Minicka et al., 2015). Interestingly, PepMV-Sp13 and PS5 passaged populations, either in single or in mixed infections, had lower nucleotide diversity values than those of the aggressive isolates PepMV-H30 or PepMV-KLP2. Therefore, our genetic stability study showed that PepMV-Sp13 and PepMV-PS5, either in single or in mixed infections, were less variable than other PepMV isolates occurring naturally in PepMV epidemics in Spain, and thus they could be safely used for cross-protection against PepMV aggressive isolates.

Cross-protection may provide an efficient means of control of viral diseases, particularly for emergent diseases (Aranda and Freitas-Astua, 2017) for which no other methods of control are yet available. Disadvantages of cross-protection have been identified, including heteroencapsidation and synergisms with unrelated viruses (for a review, see Ziebell and Carr, 2010). Given that after almost two decades of research on PepMV no resistant cultivars have been made commercially available yet, and the very broad distribution and prevalence of PepMV in intensive tomato cultivation, benefits of cross-protection largely outweigh potential disadvantages, at least while resistant tomato cultivars become available. Mixed infections with PepMV-Sp13 + PepMV-PS5 may provide a good cross-protection solution, particularly in those areas where EU, CH2 and alike strains co-circulate, as is the case for the Spanish intensive tomato crops.

## Author Contributions

JA, CG-A, RS, YH, and MA: conceptualization. JA, CG-A, JG-N, and JG-V: data curation. YH and MA: formal analysis, funding acquisition, and project administration. JA, CG-A, RS, JG-N, JG-V, and MA: investigation and methodology. JA, CG-A, and MA: writing of the first draft. JA, CG-A, RS, and MA: writing, review, and editing.

## Conflict of Interest Statement

Abiopep S.L. is developing products related with the research reported. The authors declare that the research was conducted in the absence of any commercial or financial relationships that could be construed as a potential conflict of interest.

## Footnotes

1 http://news.agropages.com/News/NewsDetail---16754-e.htm

2 http://www.geneious.com
