# Supplementary material for: Stable and Broad Spectrum Cross-Protection Against Pepino Mosaic Virus Attained by Mixed Infection
Source: Front Plant Sci. 2018 Dec 6;9:1810. doi: 10.3389/fpls.2018.01810 (PMC6291676; doi:10.3389/fpls.2018.01810)
Supplement: Supplementary file 1 [file Table_1.DOCX]

**Supplementary File S1.** Sequences from the experimental evolution study.

>H30_P1_consensus_sequence

ATGGAAAGATCAACTCTGATTAATTTACTTCAATTGCACCACTTCGAGCCAAAACTCAGTGTTGAAGGAATCATAGTTGTGCACGGAATTGCAGGCACTGGGAAAACCACTTTACTTAGGACTTTATTTTCTGCTTACCCTAGCTTAGTTATAGGTTCACCTAGGCCTTGCTATTTAGATAAACAAAACAAAATTTCACAAGTTTGCTTATCTTGCTTTCCCAATACCCATTGTGATATTGTCGATGAGTATCATTTGCTAGAAAGTTTTCTAGAACCAAAATTGGCTATCTTTGGTGACCCCTGTCAATGCACATACATTGAGAGACTTAGAGTCCCACATTACACTTCCTTCAGAACTCATAGATTTGGAAAGTCAACTGCTGAGATTTTGAACAAACTGTTTGACCTTAATATAGTCTCAGTTAAGAAAGAAGACGACATCGTTGAATTCTTTAACCCTTTTGAAGTTGACCCCACTGAGCATATCTCTGCCTCTGAAGAAGAAGTCTTGGACTTTGTTTCTGACCAAGTGGTGACCACTAGCTCAGAGGAACTAGCAGGAATTGAGTTTGCAGAAACAACTTTCTACTGCACAACATTGGCCGCAGCTGTTGCTGAAAATCCTGCTAAGACTTTCATCTCTCTGACTAGACACACCCACAAACTCACCATTGGGGAACTAAATGCCAGGTCTAACTCCTAGATGCCAGGTCTAACTCCTAGAGCTGACCTCACTGACACATACAAAATCATTGCCATTGCTTTCTTGTTGTCAGCTTGCATTTACTTCCAAAATAGCCACTACCAACCTGTTGCTGGAGACAACTTGCACCGTTTGCCTTTTGGTGGCCAATATCAAGACGGCACCAAAAAGATATCTTATTTTCCACAACAGCAGTCATACTTTCATTCTGGAAACAAATTAAATGTCCTCATACTTATCTTCATTCTCACGTTGGGTATTGTCCTCACCAATAAATTTAGTTTTAGCTTTAGTCGTACTACTCACCAGCATTCTTGCTATAACACACATTCAGCAACCAACAATACACAACCATTGTCAGGTCATCATTGAATGTCCTCATACTTATCTTCATTCTCACGTTGGGTATTGTCCTCACCAATAAATTTAGTTTTAGCTTTAGTCGTACTACTCACCAGCATTCTTGCTATAACACACATTCAGCAACCAACAATACACAACCATTGTCAGGTCATCATTGACGGTTCTGCAATAGTCATAACAAATTGTGAGAACACACCAGAAGTGCTTAAAGCAATAAACTTCTCCCCTTGGAACGGGTTAAGTTTTCCTAAATTTGAAAATTAAATGCCTGACACAACACCTGTTGCTGCCACTTCAAGTGCACCACCCACAGCCAAAGATGCTGGTGCCAAAGCTCCTTCTGACTTCTCAAATCCCAATACAGCTCCTAGTCTCAGTGATTTGAAGAAAGTCAAGTATGTCTCCACCGTGACCTCCGTGGCCACACCAGCTGAAATTGAAGCCCTAGGCAAAATCTTCACCGCCATGGGCCTTGCCGCCAATGAGACTGGTCCGGCCATGTGGGATCTAGCTCGTGCATATGCTGATGTGCAGAGTTCTAAATCGGCACAGCTGATTGGAGCTACCCCTTCCAACCCTGCACTATCACGCCGAGCCCTTGCTGCTCAGTTTGATCGAATCAATATAACCCCCAGGCAATTTTGCATGTACTTTGCCAAAGTTGTTTGGAACATACTTCTCGACAGCAACATTCCACCAGCAAATTGGGCCAAACTTGGTTACCAAGAAGATACAAAATTTGCTGCATTTGACTTCTTCGATGGAGTCACCAACCCTGCCAGCCTGCAGCCTGCTGATGGTCTTATCAGGCAGCCAAATGAGAAAGAACTAGCTGCTCACTCCGTAGCTAAGTACGGCGCCTTGGCTAGGCAAAAGATCTCCACAGGTAATTATATTACCACACTTGGAGAAGTCACACGTGGACACATGGGAGGAGCTAACACCATGTACGCGATAGACGCACCCCCTAAACTTTAA

>H30_P8_c14

ATGGAAAGATCAACTCTGATTAATTTACTTCAATTGCACCACTTCGAGCCAAAACTCAGTGTTGAAGGAATCATAGTTGTGCACGGAATTGCAGGCACTGGGAAAACCACTTTACTTAGGACTTTATTTTCTGCTTACCCTAGCTTAGTTATAGGTTCACCTAGGCCTTGCTATTTAGATAAACAAAACAAAATTTCACAAGTTTGCTTATCTTGCTTTCCCAATACCCATTGTGATATTGTCGATGAGTATCATTTGCTAGAAAGTTTTCTAGAACCAAAATTGGCTATCTTTGGTGACCCCTGTCAATGCACATACATTGAGAGACTTAGAGTCCCACATTACACTTCCTTCAGAACTCATAGATTTGGAAAGTCAACTGCTGAGATTTTGAACAAACTGTTTGACCTTAATATAGTATCAGTTAAGAAAGAAGACGACATCGTTGAATTCTTTAACCCTTTTGAAGTTGACCCCACTGAGCATATCTCTGCCTCTGAAGAAGAAGTCTTGGACTTTGTTTCTGACCAAGTGGTGACCACTAGCTCAGAGGAACTAGCAGGAATTGAGTTTGCAGAAACAACTTTCTACTGCACAACATTGGCCGCAGCTGTTGCTGAAAATCCTGCTAAGACTTTCATCTCTCTGACTAGACACACCCACAAACTCACCATTGGGGAACTAAATGCCAGGTCTAACTCCTAGATGCCAGGTCTAACTCCTAGAGCTGACCTCACTGACACATACAAAATCATTGCCATTGCTTTCTTGTTGTCAGCTTGCATTTACTTCCAAAATAGCCACTACCAACCTGTTGCTGGAGACAACTTGCACCGTTTGCCTTTTGGTGGCCAATATCAAGACGGCACCAAAAAGATATCTTATTTTCCACAACAGCAGTCATACTTTCATTCTGGAAACAAATTAAATGTCCTCATACTTATCTTCATTCTCACGTTGGGTATTGTCCTCACCAATAAATTTAGTTTTAGCTTTAGTCGTACTACTCACCAGCATTCTTGCTATAACACACATTCAGCAACCAACAATACACAACCATTGTCAGGTCATCATTGAATGTCCTCATACTTATCTTCATTCTCACGTTGGGTATTGTCCTCACCAATAAATTTAGTTTTAGCTTTAGTCGTACTACTCACCAGCATTCTTGCTATAACACACATTCAGCAACCAACAATACACAACCATTGTCAGGTCATCATTGACGGTTCTGCAATAGTCATAACAAATTGTGAGAACACACCAGAAGTGCTTAAAGCAATAAACTTCTCCCCTTGGAACGGGTTAAGTTTTCCTAAATTTGAAAATTAAATGCCTGACACAACACCTGTTGCTGCCACTTCAAGTGCACCACCCACAGCCAAAGATGCTGGTGCCAAAGCTCCTTCTGACTTCTCAAATCCCAATACAGCTCCTAGTCTCAGTGATTTGAAGAAAGTCAAGTATGTCTCCACCGTGACCTCCGTGGCCACACCAGCTGAAATTGAAGCCCTAGGCAAAATCTTCACCGCCATGGGCCTTGCCGCCAATGAGACTGGCCCGGCCATGTGGGATCTAGCTCGTGCATATGCTGATGTGCAGAGTTCTAAATCGGCACAGCTGATTGGAGCTACCCCTTCCAACCCTGCACTATCACGCCGAGCCCTTGCTGCTCAGTTTGATCGAATCAATATAACCCCCAGGCAATTTTGCATGTACTTTGCCAAAGTTGTTTGGAACATACTTCTCGACAGCAACATTCCACCAGCAAATTGGGCCAAACTTGGTTACCAAGAAGATACAAAATTTGCTGCATTTGACTTCTTCGATGGAGTCACCAACCCTGCCAGCCTGCAGCCTGCTGATGGTCTTATCAGGCAGCCAAATGAGAAAGAACTAGCTGCTCACTCCGTAGCTAAGTACGGCGCCTTGGCTAGGCAAAAGATCTCCACAGGTAATTATATTACCACACTTGGAGAAGTCACACGTGGACACATGGGAGGAGCTAACACCATGTACGCGATAGACGCACCCCCTAAACTTTAA

>H30_P8_c13

ATGGAAAGATCAACTCTGATTAATTTACTTCAATTGCACCACTTCGAGCCAAAACTCAGTGTTGAAGGAATCATAGTTGTGCACGGAATTGCAGGCACTGGGAAAACCACTTTACTTAGGACTTTATTTTCTGCTTACCCTAGCTTAGTTATAGGTTCACCTAGGCCTTGCTATTTAGATAAACAAAACAAAATTTCACAAGTTTGCTTATCTTGCTTTCCCAATACCCATTGTGATATTGTCGATGAGTATCATTTGCTAGAAAGTTTTCTAGAACCAAAATTGGCTATCTTTGGTGACCCCTGTCAATGCACATACATTGAGAGACTTAGAGTCCCACATTACACTTCCTTCAGAACTCATAGATTTGGAAAGTCAACTGCTGAGATTTTGAACAAACTGTTTGACCTTAATATAGTATCAGTTAAGAAAGAAGACGACATCGTTGAATTCTTTAACCCTTTTGAAGTTGACCCCACTGAGCATATCTCTGCCTCTGAAGAAGAAGTCTTGGACTTTGTTTCTGACCAAGTGGTGACCACTAGCTCAGAGGAACTAGCAGGAATTGAGTTTGCAGAAACAACTTTCTACTGCACAACATTGGCCGCAGCTGTTGCTGAAAATCCTGCTAAGACTTTCATCTCTCTGACTAGACACACCCACAAACTCACCATTGGGGAACTAAATGCCAGGTCTAACTCCTAGATGCCAGGTCTAACTCCTAGAGCTGACCTCACTGACACATACAAAATCATTGCCATTGCTTTCTTGTTGTCAGCTTGCATTTACTTCCAAAATAGCCACTACCAACCTGTTGCTGGAGACAACTTGCACCGTTTGCCTTTTGGTGGCCAATATCAAGACGGCACCAAAAAGATATCTTATTTTCCACAACAGCAGTCATACTTTCATTCTGGAAACAAATTAAATGTCCTCATACTTATCTTCATTCTCACGTTGGGTATTGTCCTCACCAATAAATTTAGTTTTAGCTTTAGTCGTACTACTCACCAGCATTCTTGCTATAACACACATTCAGCAACCAACAATACACAACCATTGTCAGGTCATCATTGAATGTCCTCATACTTATCTTCATTCTCACGTTGGGTATTGTCCTCACCAATAAATTTAGTTTTAGCTTTAGTCGTACTACTCACCAGCATTCTTGCTATAACACACATTCAGCAACCAACAATACACAACCATTGTCAGGTCATCATTGACGGTTCTGCAATAGTCATAACAAATTGTGAGAACACACCAGAAGTGCTTAAAGCAATAAACTTCTCCCCTTGGAACGGGTTAAGTTTTCCTAAATTTGAAAATTAAATGCCTGACACAACACCTGTTGCTGCCACTTCAAGTGCACCACCCACAGCCAAAGATGCTGGTGCCAAAGCTCCTTCTGACTTCTCAAATCCCAATACAGCTCCTAGTCTCAGTGATTTGAAGAAAGTCAAGTATGTCTCCACCGTGACCTCCGTGGCCACACCAGCTGAAATTGAAGCCCTAGGCAAAATCTTCACCGCTATGGGCCTTGCCGCCAATGAGACTGGCCCGGCCATGTGGGATCTAGCTCGTGCATATGCTGATGTGCAGAGTTCTAAATCGGCACAGCTGATTGGAGCTACCCCTTCCAACCCTGCACTATCACGCCGAGCCCTTGCTGCTCAGTTTGATCGAATCAGTATAACCCCCAGGCAATTTTGCATGTACTTTGCCAAAGTTGTTTGGAACATACTTCTCGACAGCAACATTCCACCAGCAAATTGGGCCAAACTTGGTTACCAAGAAGATACAAAATTTGCTGCATTTGACTTCTTCGATGGAGTCACCAACCCTGCCAGCCTGCAGCCTGCTGATGGTCTTATCAGGCAGCCAAATGAGAAAGAACTAGCTGCTCACTCCGTAGCTAAGTACGGCGCCTTGGCTAGGCAAAAGATCTCCACAGGTAATTATATTACCACACTTGGAGAAGTCACACGTGGACACATGGGAGGAGCTAACACCATGTACGCGATAGACGCACCCCCTAAACTTTAA

>H30_P8_c12

ATGGAAAGATCAACTCTGATTAATTTACTTCAATTGCACCACTTCGAGCCAAAACTCAGTGTTGAAGGAATCATAGTTGTGCACGGAATTGCAGGCACTGGGAAAACCACTTTACTTAGGACTTTATTTTCTGCTTACCCTAGCTTAGTTATAGGTTCACCTAGGCCTTGCTATTTAGATAAACAAAACAAAATTTCACAAGTTTGCTTATCTTGCTTTCCCAATACCCATTGTGATATTGTCGATGAGTATCATTTGCTAGAAAGTTTTCTAGAACCAAAATTGGCTATCTTTGGTGACCCCTGTCAATGCACATACATTGAGAGACTTAGAGTCCCACATTACACTTCCTTCAGAACTCATAGATTTGGAAAGTCGACTGCTGAGATTTTGAACAAACTGTTTGACCTTAATATAGTCTCAGTTAAGAAAGAAGACGACATCGTTGAATTCTTTAACCCTTTTGAAGTTGACCCCACTGAGCATATCTCTGCCTCTGAAGAAGAAGTCTTGGACTTTGTTTCTGACCAAGTGGTGACCACTAGCTCAGAGGAACTAGCAGGAATTGAGTTTGCAGAAACAACTTTCTACTGCACAACATTGGCCGCAGCTGTTGCTGAAAATCCTGCTAAGACTTTCATCTCTCTGACTAGACACACCCACAAACTCACCATTGGGGAACTAAATGCCAGGTCTAACTCCTAGATGCCAGGTCTAACTCCTAGAGCTGACCTCACTGACACATACAAAATCATTGCCATTGCTTTCTTGTTGTCAGCTTGCATTTACTTCCAAAATAGCCACTACCAACCTGTTGCTGGAGACAACTTGCACCGTTTGCCTTTTGGTGGCCAATATCAAGACGGCACCAAAAAGATATCTTATTTTCCACAACAGCAGTCATACTTTCATTCTGGAAACAAATTAAATGTCCTCATACTTATCTTCATTCTCACGTTGGGTATTGTCCTCACCAATAAATTTAGTTTTAGCTTTAGTCGTACTACTCACCAGCATTCTTGCTATAACACACATTCAGCAACCAACAATACACAACCATTGTCAGGTCATCATTGAATGTCCTCATACTTATCTTCATTCTCACGTTGGGTATTGTCCTCACCAATAAATTTAGTTTTAGCTTTAGTCGTACTACTCACCAGCATTCTTGCTATAACACACATTCAGCAACCAACAATACACAACCATTGTCAGGTCATCATTGACGGTTCTGCAATAGTCATAACAAATTGTGAGAACACACCAGAAGTGCTTAAAGCAATAAACTTCTCCCCTTGGAACGGGTTAAGTTTTCCTAAATTTGAAAATTAAATGCCTGACACAACACCTGTTGCTGCCACTTCAAGTGCACCACCCACAGCCAAAGATGCTGGTGCCAAAGCTCCTTCTGACTTCTCAAATCCCAATACAGCTCCTAGTCTCAGTGATTTGAAGAAAGTCAAGTATGTCTCCACAGTGACCTCCGTGGCCACACCAGCTGAAATTGAAGCCCTAGGCAAAATCTTCACCGCCATGGGCCTTGCCGCCAATGAGACTGGCCCGGCCATGTGGGATCTAGCTCGTGCATATGCTGATGTGCAGAGTTCTAAATCGGCACAGCTGATTGGAGCTACCCCTTCCAACCCTGCACTATCACGCCGAGCCCTTGCTGCTCAGTTTGATCGAATCAATATAACCCCCAGGCAATTTTGCATGTACTTTGCCAAAGTTGTTTGGAACATACTTCTCGACAGCAACATTCCACCAGCAAATTGGGCCAAACTTGGTTACCAAGAAGATACAAAATTTGCTGCATTTGACTTCTTCGATGGAGTCACCAACCCTGCCAGCCTGCAGCCTGCTGATGGTCTTATCAGGCAGCCAAATGAGAAAGAACTAGCTGCTCACTCCGTAGCTAAGTACGGCGCCTTGGCTAGGCAAAAGATCTCCACAGGTAATTATATTACCACACTTGGAGAAGTCACACGTGGACACATGGGAGGAGCTAACACCATGTACGCGATAGACGCACCCCCTAAACTTTAA

>H30_P8_c11

ATGGAAAGATCAACTCTGATTAATTTACTTCAATTGCACCACTTCGAGCCAAAACTCAGTGTTGAAGGAATCATAGTTGTGCACGGAATTGCAGGCACTGGGAAAACCACTTTACTTAGGACTTTATTTTCTGCTTACCCTAGCTTAGTTATAGGTTCACCTAGGCCTTGCTATTTAGATAAACAAAACAAAATTTCACAAGTTTGCTTATCTTGCTTTCCCAATACCCATTGTGATATTGTCGATGAGTATCATTTGCTAGAAAGTTTTCTAGAACCAAAATTGGCTATCTTTGGTGACCCCTGTCAATGCACATACATTGAGAGACTTAGAGTCCCACATTACACTTCCTTCAGAACTCATAGATTTGGAAAGTCGACTGCTGAGATTTTGAACAAACTGTTTGACCTTAATATAGTCTCAGTTAAGAAAGAAGACGACATCGTTGAATTCTTTAACCCTTTTGAAGTTGACCCCACTGAGCATATCTCTGCCTCTGAAGAAGAAGTCTTGGACTTTGTTTCTGACCAAGTGGTGACCACTAGCTCAGAGGAACTAGCAGGAATTGAGTTTGCAGAAACAACTTTCTACTGCACAACATTGGCCGCAGCTGTTGCTGAAAATCCTGCTAAGACTTTCATCTCTCTGACTAGACACACCCACAAACTCACCATTGGGGAACTAAATGCCAGGTCTAACTCCTAGATGCCAGGTCTAACTCCTAGAGCTGACCTCACTGACACATACAAAATCATTGCCATTGCTTTCTTGTTGTCAGCTTGCATTTACTTCCAAAATAGCCACTACCAACCTGTTGCTGGAGACAACTTGCACCGTTTGCCTTTTGGTGGCCAATATCAAGACGGCACCAAAAAGATATCTTATTTTCCACAACAGCAGTCATACTTTCATTCTGGAAACAAATTAAATGTCCTCATACTTATCTTCATTCTCACGTTGGGTATTGTCCTCACCAATAAATTTAGTTTTAGCTTTAGTCGTACTACTCACCAGCATTCTTGCTATAACACACATTCAGCAACCAACAATACACAACCATTGTCAGGTCATCATTGAATGTCCTCATACTTATCTTCATTCTCACGTTGGGTATTGTCCTCACCAATAAATTTAGTTTTAGCTTTAGTCGTACTACTCACCAGCATTCTTGCTATAACACACATTCAGCAACCAACAATACACAACCATTGTCAGGTCATCATTGACGGTTCTGCAATAGTCATAACAAATTGTGAGAACACACCAGAAGTGCTTAAAGCAATAAACTTCTCCCCTTGGAACGGGTTAAGTTTTCCTAAATTTGAAAATTAAATGCCTGACACAACACCTGTTGCTGCCACTTCAAGTGCACCACCCACAGCCAAAGATGCTGGTGCCAAAGCTCCTTCTGACTTCTCAAATCCCAATACAGCTCCTAGTCTCAGTGATTTGAAGAAAGTCAAGTATGTCTCCACCGTGACCTCCGTGGCCACACCAGCTGAAATTGAAGCCCTAGGCAAAATCTTCACCGCCATGGGCCTTGCCGCCAATGAGACTGGTCCGGCCATGTGGGATCTAGCTCGTGCATATGCTGATGTGCAGAGTTCTAAATCGGCACAGCTGATTGGAGCTACCCCTTCCAACCCTGCACTATCACGCCGAGCCCTTGCTGCTCAGTTTGATCGAATCAATATAACCCCCAGGCAATTTTGCATGTACTTTGCCAAAGTTGTTTGGAACATACTTCTCGACAGCAACATTCCACCAGCAAATTGGGCCAAACTTGGTTACCAAGAAGATACAAAATTTGCTGCATTTGACTTCTTCGATGGAGTCACCAACCCTGCCAGCCTGCAGCCTGCTGATGGTCTTATCAGGCAGCCAAATGAGAAAGAACTAGCTGCTCACTCCGTAGCTAAGTACGGCGCCTTGGCTAGGCAAAAGATCTCCACAGGTAATTATATTACCACACTTGGAGAAGTCACACGTGGACACATGGGAGGAGCTAACACCATGTACGCGATAGACGCACCCCCTAAACTTTAA

>H30_P8_c10

ATGGAAAGATCAACTCTGATTAATTTACTTCAATTGCACCACTTCGAGCCAAAACTCAGTGTTGAAGGAATCATAGTTGTGCACGGAATTGCAGGCACTGGGAAAACCACTTTACTTAGGACTTTATTTTCTGCTTACCCTAGCTTAGTTATAGGTTCACCTAGGCCTTGCTATTTAGATAAACAAAACAAAATTTCACAAGTTTGCTTATCTTGCTTTCCCAATACCCATTGTGATATTGTCGATGAGTATCATTTGCTAGAAAGTTTTCTAGAACCAAAATTGGCTATCTTTGGTGACCCCTGTCAATGCACATACATTGAGAGACTTAGAGTCCCACATTACACTTCCTTCAGAACTCATAGATTTGGAAAGTCGACTGCTGAGATTTTGAACAAACTGTTTGACCTTAATATAGTCTCAGTTAAGAAAGAAGACGACATCGTTGAATTCTTTAACCCTTTTGAAGTTGACCCCACTGAGCATATCTCTGCCTCTGAAGAAGAAGTCTTGGACTTTGTTTCTGACCAAGTGGTGACCACTAGCTCAGAGGAACTAGCAGGAATTGAGTTTGCAGAAACAACTTTCTACTGCACAACATTGGCCGCAGCTGTTGCTGAAAATCCTGCTAAGACTTTCATCTCTCTGACTAGACACACCCACAAACTCACCATTGGGGAACTAAATGCCAGGTCTAACTCCTAGATGCCAGGTCTAACTCCTAGAGCTGACCTCACTGACACATACAAAATCATTGCCATTGCTTTCTTGTTGTCAGCTTGCATTTACTTCCAAAATAGCCACTACCAACCTGTTGCTGGAGACAACTTGCACCGTTTGCCTTTTGGTGGCCAATATCAAGACGGCACCAAAAAGATATCTTATTTTCCACAACAGCAGTCATACTTTCATTCTGGAAACAAATTAAATGTCCTCATACTTATCTTCATTCTCACGTTGGGTATTGTCCTCACCAATAAATTTAGTTTTAGCTTTAGTCGTACTACTCACCAGCATTCTTGCTATAACACACATTCAGCAACCAACAATACACAACCATTGTCAGGTCATCATTGAATGTCCTCATACTTATCTTCATTCTCACGTTGGGTATTGTCCTCACCAATAAATTTAGTTTTAGCTTTAGTCGTACTACTCACCAGCATTCTTGCTATAACACACATTCAGCAACCAACAATACACAACCATTGTCAGGTCATCATTGACGGTTCTGCAATAGTCATAACAAATTGTGAGAACACACCAGAAGTGCTTAAAGCAATAAACTTCTCCCCTTGGAACGGGTTAAGTTTTCCTAAATTTGAAAATTAAATGCCTGACACAACACCTGTTGCTGCCACTTCAAGTGCACCACCCACAGCCAAAGATGCTGGTGCCAAAGCTCCTTCTGACTTCTCAAATCCCAATACAGCTCCTAGTCTCAGTGATTTGAAGAAAGTCAAGTATGTCTCCACCGTGACCTCCGTGGCCACACCAGCTGAAATTGAAGCCCTAGGCAAAATCTTCACCGCCATGGGCCTTGCCGCCAATGAGACTGGTCCGGCCATGTGGGATCTAGCTCGTGCATATGCTGATGTGCAGAGTTCTAAATCGGCACAGCTGATTGGAGCTACCCCTTCCAACCCTGCACTATCACGCCGAGCCCTTGCTGCTCAGTTTGATCGAATCAATATAACCCCCAGGCAATTTTGCATGTACTTTGCCAAAGTTGTTTGGAACATACTTCTCGACAGCAACATTCCACCAGCAAATTGGGCCAAACTTGGTTACCAAGAAGATACAAAATTTGCTGCATTTGACTTCTTCGATGGAGTCACCAACCCTGCCAGCCTGCAGCCTGCTGATGGTCTTATCAGGCAGCCAAATGAGAAAGAACTAGCTGCTCACTCCGTAGCTAAGTACGGCGCCTTGGCTAGGCAAAAGATCTCCACAGGTAATTATATTACCACACTTGGAGAAGTCACACGTGGACACATGGGAGGAGCTAACACCATGTACGCGATAGACGCACCCCCTAAACTTTAA

>H30_P8_c9

ATGGAAAGATCAACTCTGATTAATTTACTTCAATTGCACCACTTCGAGCCAAAACTCAGTGTTGAAGGAATCATAGTTGTGCACGGAATTGCAGGCACTGGGAAAACCACTTTACTTAGGACTTTATTTTCTGCTTACCCTAGCTTAGTTATAGGTTCACCTAGGCCTTGCTATTTAGATAAACAAAACAAAATTTCACAAGTTTGCTTATCTTGCTTTCCCAATACCCATTGTGATATTGTCGATGAGTATCATTTGCTAGAAAGTTTTCTAGAACCAAAATTGGCTATCTTTGGTGACCCCTGTCAATGCACATACATTGAGAGACTTAGAGTCCCACATTACACTTCCTTCAGAACTCATAGATTTGGAAAGTCAACTGCTGAGATTTTGAACAAACTGTTTGACCTTAATATAGTATCAGTTAAGAAAGAAGACGACATCGTTGAATTCTTTAACCCCTTTGAAGTTGACCCCACTGAGCATATCTCTGCCTCTGAAGAAGAAGTCTTGGACTTTGTTTCTGACCAAGTGGTGACCACTAGCTCAGAGGAACTAGCAGGAATTGAGTTTGCAGAAACAACTTTCTACTGCACAACATTGGCCGCAGCTGTTGCTGAAAATCCTGCTAAGACTTTCATCTCTCTGACTAGACACACCCACAAACTCACCATTGGGGAACTAAATGCCAGGTCTAACTCCTAGATGCCAGGTCTAACTCCTAGAGCTGACCTCACTGACACACACAAAATCATTGCCATTGCTTTCTTGTTGTCAGCTTGCATTTACTTCCAAAATAGCCACTACCAACCTGTTGCTGGAGACAACTTGCACCGTTTGCCTTTTGGTGGCCAATATCAAGACGGCACCAAAAAGATATCTTATTTTCCACAACAGCAGTCATACTTTCATTCTGGAAACAAATTAAATGTCCTCATACTTATCTTCATTCTCACGTTGGGTATTGTCCTCACCAATAAATTTAGTTTTAGCTTTAGTCGTACTACTCACCAGCATTCTTGCTATAACACACATTCAGCAACCAACAATACACAACCATTGTCAGGTCATCATTGAATGTCCTCATACTTATCTTCATTCTCACGTTGGGTATTGTCCTCACCAATAAATTTAGTTTTAGCTTTAGTCGTACTACTCACCAGCATTCTTGCTATAACACACATTCAGCAACCAACAATACACAACCATTGTCAGGTCATCATTGACGGTTCTGCAATAGTCATAACAAATTGTGAGAACACACCAGAAGTGCTTAAAGCAATAAACTTCTCCCCTTGGAACGGGTTAAGTTTTCCTAAATTTGAAAATTAAATGCCTGACACAACACCTGTTGCTGCCACTTCAAGTGCACCACCCACAGCCAAAGATGCTGGTGCCAAAGCTCCTTCTGACTTCTCAAATCCCAATACAGCTCCTAGTCTCAGTGATTTGAAGAAAGTCAAGTATGTCTCCACCGTGACCTCCGTGGCCACACCAGCTGAAATTGAAGCCCTAGGCAAAATCTTCACCGCCATGGGCCTTGCCGCCAATGAGACTGGCCCGGCCATGTGGGATCTAGCTCGTGCATATGCTGATGTGCAGAGTTCTAAATCGGCACAGCTGATTGGAGCTACCCCTTCCAACCCTGCACTATCACGCCGAGCCCTTGCTGCTCAGTTTGATCGAATCAATATAACCCCCAGGCAATTTTGCATGTACTTTGCCAAAGTTGTTTGGAACATACTTCTCGACAGCAACATTCCACCAGCAAATTGGGCCAAACTTGGTTACCAAGAAGATACAAAATTTGCTGCATTTGACTTCTTCGATGGAGTCACCAACCCTGCCAGCCTGCAGCCTGCTGATGGTCTTATCAGGCAGCCAAATGAGAAAGAACTAGCTGCTCACTCCGTAGCTAAGTACGGCGCCTTGGCTAGGCAAAAGATCTCCACAGGTAATTATATTACCACACTTGGAGAGGTCACACGTGGACACATGGGAGGAGCTAACACCATGTACGCGATAGACGCACCCCCTAAACTTTAA

>H30_P8_c8

ATGGAAAGATCAACTCTGATTAATTTACTTCAATTGCACCACTTCGAGCCAAAACTCAGTGTTGAAGGAATCATAGTTGTGCACGGAATTGCAGGCACTGGGAAAACCACTTTACTTAGGACTTTATTTTCTGCTTACCCTAGCTTAGTTATAGGTTCACCTAGGCCTTGCTATTTAGATAAACAAAACAAAATTTCACAAGTTTGCTTATCTTGCTTTCCCAATACCCATTGTGATATTGTCGATGAGTATCATTTGCTAGAAAGTTTTCTAGAACCAAAATTGGCTATCTTTGGTGACCCCTGTCAATGCACATACATTGAGAGACTTAGAGTCCCACATTACACTTCCTTCAGAACTCATAGATTTGGAAAGTCAACTGCTGAGATTTTGAACAAACTGTTTGACCTTAATATAGTCTCAGTTAAGAAAGAAGACGACATCGTTGAATTCTTTAACCCTTTTGAAGTTGACCCCACTGAGCATATCTCTGCCTCTGAAGAAGAAGTCTTGGACTTTGTTTCTGACCAAGTGGTGACCACTAGCTCAGAGGAACTAGCAGGAATTGAGTTTGCAGAAACAACTTTCTACTGCACAACATTGGCCGCAGCTGTTGCTGAAAATCCTGCTAAGACTTTCATCTCTCTGACTAGACACACCCACAAACTCACCATTGGGGAACTAAATGCCAGGTCTAACTCCTAGATGCCAGGTCTAACTCCTAGAGCTGACCTCACTGACACATACAAAATCATTGCCATTGCTTTCTTGTTGTCAGCTTGCATTTACTTCCAAAATAGCCACTACCAACCTGTTGCTGGAGACAACTTGCACCGTTTGCCTTTTGGTGGCCAATATCAAGACGGCACCAAAAAGATATCTTATTTTCCACAACAGCAGTCATACTTTCATTCTGGAAACAAATTAAATGTCCTCATACTTATCTTCATTCTCACGTTGGGTATTGTCCTCACCAATAAATTTAGTTTTAGCTTTAGTCGTACTACTCACCAGCATTCTTGCTATAACACACATTCAGCAACCAACAATACACAACCATTGTCAGGTCATCATTGAATGTCCTCATACTTATCTTCATTCTCACGTTGGGTATTGTCCTCACCAATAAATTTAGTTTTAGCTTTAGTCGTACTACTCACCAGCATTCTTGCTATAACACACATTCAGCAACCAACAATACACAACCATTGTCAGGTCATCATTGACGGTTCTGCAATAGTCATAACAAATTGTGAGAACACACCAGAAGTGCTTAAAGCAATAAACTTCTCCCCTTGGAACGGGTTAAGTTTTCCTAAATTTGAAAATTAAATGCCTGACACAACACCTGTTGCTGCCACTTCAAGTGCACCACCCACAGCCAAAGATGCTGGTGCCAAAGCTCCTTCTGACTTCTCAAATCCCAATACAGCTCCTAGTCTCAGTGATTTGAAGAAAGTCAAGTATGTCTCCACAGTGACCTCCGTGGCCACACCAGCTGAAATTGAAGCCCTAGGCAAAATCTTCACCGCCATGGGCCTTGCCGCCAATGAGACTGGCCCGGCCATGTGGGATCTAGCTCGTGCATATGCTGATGTGCAGAGTTCTAAATCGGCACAGCTGATTGGAGCTACCCCTTCCAACCCTGCACTATCACGCCGAGCCCTTGCTGCTCAGTTTGATCGAATCAATATAACCCCCAGGCAATTTTGCATGTACTTTGCCAAAGTTGTTTGGAACATACTTCTCGACAGCAACATTCCACCAGCAAATTGGGCCAAACTTGGTTACCAAGAAGATACAAAATTTGCTGCATTTGACTTCTTCGATGGAGTCACCAACCCTGCCAGCCTGCAGCCTGCTGATGGTCTTATCAGGCAGCCAAATGAGAAAGAACTAGCTGCTCACTCCGTAGCTAAGTACGGCGCCTTGGCTAGGCAAAAGATCTCCACAGGTAATTATATTACCACACTTGGAGAAGTCACACGTGGACACATGGGAGGAGCTAACACCATGTACGCGATAGACGCACCCCCTAAACTTTAA

>H30_P8_c7

ATGGAAAGATCAACTCTGATTAATTTACTTCAATTGCACCACTTCGAGCCAAAACTCAGTGTTGAAGGAATCATAGTTGTGCACGGAATTGCAGGCACTGGGAAAACCACTTTACTTAGGACTTTATTTTCTGCTTACCCTAGCTTAGTTATAGGTTCACCTAGGCCTTGCTATTTAGATAAACAAAACAAAATTTCACAAGTTTGCTTATCTTGCTTTCCCAATACCCATTGTGATATTGTCGATGAGTATCATTTGCTAGAAAGTTTTCTAGAACCAAAATTGGCTATCTTTGGTGACCCCTGTCAATGCACATACATTGAGAGACTTAGAGTCCCACATTACACTTCCTTCAGAACTCATAGACTTGGAAAGTCAACTGCTGAGATTTTGAACAAACTGTTTGACCTTAATATAGTCTCAGTTAAGAAAGAAGACGACATCGTTGAATTCTTTAACCCTTTTGAAGTTGACCCCACTGAGCATATCTCTGCCTCTGAAGAAGAAGTCTTGGACTTTGTTTCTGACCAAGCGGTGACCACTAGCTCAGAGGAACTAGCAGGAATTGAGTTTGCAGAAACAACTTTCTACTGCACAACATTGGCCGCAGCTGTTGCTGAAAATCCTGCTAAGACTTTCATCTCTCTGACTAGACACACCCACAAACTCACCATTGGGGAACTAAATGCCAGGTCTAACTCCTAGATGCCAGGTCTAACTCCTAGAGCTGACCTCACTGACACATACAAAATCATTGCCATTGCTTTCTTGTTGTCAGCTTGCATTTACTTCCAAAATAGCCACTACCAACCTGTTGCTGGAGACAACTTGCACCGTTTGCCTTTTGGTGGCCAATATCAAGACGGCACCAAAAAGATATCTTATTTTCCACAACAGCAGTCATACTTTCATTCTGGAAACAAATTAAATGTCCTCATACTTATCTTCATTCTCACGTTGGGTATTGTCCTCACCAATAAATTTAGTTTTAGCTTTAGTCGTACTACTCACCAGCATTCTTGCTATAACACACATTCAGCAACCAACAATACACAACCATTGTCAGGTCATCATTGAATGTCCTCATACTTATCTTCATTCTCACGTTGGGTATTGTCCTCACCAATAAATTTAGTTTTAGCTTTAGTCGTACTACTCACCAGCATTCTTGCTATAACACACATTCAGCAACCAACAATACACAACCATTGTCAGGTCATCATTGACGGTTCTGCAATAGTCATAACAAATTGTGAGAACACACCAGAAGTGCTTAAAGCAATAAACTTCTCCCCTTGGAACGGGTTAAGTTTTCCTAAATTTGAAAATTAAATGCCTGACACAACACCTGTTGCTGCCACTTCAAGTGCACCACCCACAGCCAAAGATGCTGGTGCCAAAGCTCCTTCTGACTTCTCAAATCCCAATACAGCTCCTAGTCTCAGTGATTTGAAGAAAGTCAAGTATGTCTCCACAGTGACCTCCGTGGCCACACCAGCTGAAATTGAAGCCCTAGGCAAAATCTTCACCGCCATGGGCCTTGCCGCCAATGAGACTGGCCCGGCCATGTGGGATCTAGCTCGTGCATATGCTGATGTGCAGAGTTCTAAATCGGCACAGCTGATTGGAGCTACCCCTTCCAACCCTGCACTATCACGCCGAGCCCTTGCTGCTCAGTTTGATCGAATCAATATAACCCCCAGGCAATTTTGCATGTACTTTGCCAAAGTTGTTTGGAACATACTTCTCGACAGCAACATTCCACCAGCAAATTGGGCCAAACTTGGTTACCAAGAAGATACAAAATTTGCTGCATTTGACTTCTTCGATGGAGTCACCAACCCTGCCAGCCTGCAGCCTGCTGATGGTCTTATCAGGCAGCCAAATGAGAAAGAACTAGCTGCTCACTCCGTAGCTAAGTACGGCGCCTTGGCTAGGCAAAAGATCTCCACAGGTAATTATATTACCACACTTGGAGAAGTCACACGTGGACACATGGGAGGAGCTAACACCATGTACGCGATAGACGCACCCCCTAAACTTTAA

>H30_P8_c6

ATGGAAAGATCAACTCTGATTAATTTACTTCAATTGCACCACTTCGAGCCAAAACTCAGTGTTGAAGGAATCATAGTTGTGCACGGAATTGCAGGCACTGGGAAAACCACTTTACTTAGGACTTTATTTTCTGCTTACCCTAGCTTAGTTATAGGTTCACCTAGGCCTTGCTATTTAGATAAACAAAACAAAATTTCACAAGTTTGCTTATCTTGCTTTCCCAATACCCATTGTGATATTGTCGATGAGTATCATTTGCTAGAAAGTTTTCTAGAACCAAAATTGGCTATCTTTGGTGACCCCTGTCAATGCACATACATTGAGAGACTTAGAGTCCCACATTACACTTCCTTCAGAACTCATAGATTTGGAAAGTCGACTGCTGAGATTTTGAACAAACTGTTTGACCTTAATATAGTCTCAGTTAAGAAAGAAGACGACATCGTTGAATTCTTTAACCCTTTTGAAGCTGACCCCACTGAGCATATCTCTGCCTCTGAAGAAGAAGTCTTGGACTTTGTTTCTGACCAAGTGGTGACCACTAGCTCAGAGGAACTAGCAGGAATTGAGTTTGCAGAAACAACTTTCTACTGCACAACATTGGCCGCAGCTGTTGCTGAAAATCCTGCTAAGACTTTCATCTCTCTGACTAGACACACCCACAAACTCACCATTGGGGAACTAAATGCCAGGTCTAACTCCTAGATGCCAGGTCTAACTCCTAGAGCTGACCTCACTGACACATACAAAATCATTGCCATTGCTTTCTTGTTGTCAGCTTGCATTTACTTCCAAAATAGCCACTACCAACCTGTTGCTGGAGACAACTTGCACCGTTTGCCTTTTGGTGGCCAATATCAAGACGGCACCAAAAAGATATCTTATTTTCCACAACAGCAGTCATACTTTCATTCTGGAAACAAATTAAATGTCCTCATACTTATCTTCATTCTCACGTTGGGTATTGTCCTCACCAATAAATTTAGTTTTAGCTTTAGTCGTACTACTCACCAGCATTCTTGCTATAACACACATTCAGCAACCAACAATACACAACCATTGTCAGGTCATCATTGAATGTCCTCATACTTATCTTCATTCTCACGTTGGGTATTGTCCTCACCAATAAATTTAGTTTTAGCTTTAGTCGTACTACTCACCAGCATTCTTGCTATAACACACATTCAGCAACCAACAATACACAACCATTGTCAGGTCATCATTGACGGTTCTGCAATAGTCATAACAAATTGTGAGAACACACCAGAAGTGCTTAAAGCAATAAACTTCTCCCCTTGGAACGGGTTAAGTTTTCCTAAATTTGAAAATTAAATGCCTGACACAACACCTGTTGCTGCCACTTCAAGTGCACCACCCACAGCCAAAGATGCTGGTGCCAAAGCTCCTTCTGACTTCTCAAATCCCAATACAGCTCCTAGTCTCAGTGATTTGAAGAAAGTCAAGTATGTCTCCACCGTGACCTCCGTGGCCACACCAGCTGAAATTGAAGCCCTAGGCAAAATCTTCACCGCCATGGGCCTTGCCGCCAATGAGACTGGCCCGGCCATGTGGGATCTAGCTCGTGCATATGCTGATGTGCAGAGTTCTAAATCGGCACAGCTGATTGGAGCTACCCCTTCCAACCCTGCACTATCACGCCGAGCCCTTGCTGCTCAGTTTGATCGAATCAATATAACCCCCAGGCAATTTTGCATGTACTTTGCCAAAGTTGTTTGGAACATACTTCTCGACAGCAACATTCCACCAGCAAATTGGGCCAAACTTGGTTACCAAGAAGATACAAAATTTGCTGCATTTGACTTCTTCGATGGAGTCACCAACCCTGCCAGCCTGCAGCCTGCTGATGGTCTTATCAGGCAGCCAAATGAGAAAGAACTAGCTGCTCACTCCGTAGCTAAGTACGGCGCCTTGGCTAGGCAAAAGATCTCCACAGGTAATTATATTACCACACTTGGAGAAGTCACACGTGGACACATGGGAGGAGCTAACACCATGTACGCGATAGACGCACCCCCTAAACTTTAA

>H30_P8_c5

ATGGAAAGATCAACTCTGATTAATTTACTTCAATTGCACCACTTCGAGCCAAAACTCAGTGTTGAAGGAATCATAGTTGTGCACGGAATTGCAGGCACTGGGAAAACCACTTTACTTAGGACTTTATTTTCTGCTTACCCTAGCTTAGTTATAGGTTCACCTAGGCCTTGCTATTTAGATAAACAAAACAAAATTTCACAAGTTTGCTTATCTTGCTTTCCCAATACCCATTGTGATATTGTCGATGAGTATCATTTGCTAGGAAGTTTTCTAGAACCAAAATTGGCTATCTTTGGTGACCCCTGTCAATGCACATACATTGAGAGACTTAGAGTCCCACATTACACTTCCTTCAGAACTCATAGATTTGGAAAGTCAACTGCTGAGATTTTGAACAAACTGTTTGACCTTAATATAGTATCAGTTAAGAAAGAAGACGACATCGTTGAATTCTTTAACCCTTTTGAAGTTGACCCCACTGAGCATATCTCTGCCTCTGAAGAAGAAGTCTTGGACTTTGTTTCTGACCAAGTGGTGACCACTAGCTCAGAGGAACTAGCAGGAATTGAGTTTGCAGAAACAACTTTCTACTGCACAACATTGGCCGCAGCTGTTGCTGAAAATCCTGCTAAGACTTTCATCTCTCTGACTAGACACACCCACAAACTCACCATTGGGGAACTAAATGCCAGGTCTAACTCCTAGATGCCAGGTCTAACTCCTAGAGCTGACCTCACTGACACATACAAAATCATTGCCATTGCTTTCTTGTTGTCAGCTTGCATTTACTTCCAAAATAGCCACTACCAACCTGTTGCTGGAGACAACTTGCACCGTTTGCCTTTTGGTGGCCAATATCAAGACGGCACCAAAAAGATATCTTATTTTCCACAACAGCAGTCATACTTTCATTCTGGAAACAAATTAAATGTCCTCATACTTATCTTCATTCTCACGTTGGGTATTGTCCTCACCAATAAATTTAGTTTTAGCTTTAGTCGTACTACTCACCAGCATTCTTGCTATAACACACATTCAGCAACCAACAATACACAACCATTGTCAGGTCATCATTGAATGTCCTCATACTTATCTTCATTCTCACGTTGGGTATTGTCCTCACCAATAAATTTAGTTTTAGCTTTAGTCGTACTACTCACCAGCATTCTTGCTATAACACACATTCAGCAACCAACAATACACAACCATTGTCAGGTCATCATTGACGGTTCTGCAATAGTCATAACAAATTGTGAGAACACACCAGAAGTGCTTAAAGCAATAAACTTCTCCCCTTGGAACGGGTTAAGTTTTCCTAAATTTGAAAATTAAATGCCTGACACAACACCTGTTGCTGCCACTTCAAGTGCACCACCCACAGCCAAAGATGCTGGTGCCAAAGCTCCTTCTGACTTCTCAAATCCCAATACAGCTCCTAGTCTCAGTGATTTGAAGAAAGTCAAGTATGTCTCCACAGTGACCTCCGTGGCCACACCAGCTGAAATTGAAGCCCTAGGCAAAATCTTCACCGCCATGGGCCTTGCCGCCAATGAGACTGGCCCGGCCATGTGGGATCTAGCTCGTGCATATGCTGATGTGCAGAGTTCTAAATCGGCACAGCTGATTGGAGCTACCCCTTCCAACCCTGCACTATCACGCCGAGCCCTTGCTGCTCAGTTTGATCGAATCAATATAACCCCCAGGCAATTTTGCATGTACTTTGCCAAAGTTGTTTGGAACATACTTCTCGACAGCAACATTCCACCAGCAAATTGGGCCAAACTTGGTTACCAAGAAGATACAAAATTTGCTGCATTTGACTTCTTCGATGGAGTCACCAACCCTGCCAGCCTGCAGCCTGCTGATGGTCTTATCAGGCAGCCAAATGAGAAAGAACTAGCTGCTCACTCCGTAGCTAAGTACGGCGCCTTGGCTAGGCAAAAGATCTCCACAGGTAATTATATTACCACACTTGGAGAAGTCACACGTGGACACATGGGAGGAGCTAACACCATGTACGCGATAGACGCACCCCCTAAACTTTAA

>H30_P8_c4

ATGGAAAGATCAACTCTGATTAATTTACTTCAATTGCACCACTTCGAGCCAAAACTCAGTGTTGAAGGAATCATAGTTGTGCACGGAATTGCAGGCACTGGGAAAACCACTTTACTTAGGACTTTATTTTCTGCTTACCCTAGCTTAGTTATAGGTTCACCTAGGCCTTGCTATTTAGATAAACAAAACAAAATTTCACAAGTTTGCTTATCTTGCTTTCCCAATACCCATTGTGATATTGTCGATGAGTATCATTTGCTAGAAAGTTTTCTAGAACCAAAATTGGCTATCTTTGGTGACCCCTGTCAATGCACATACATTGAGAGACTTAGAGTCCCACATTACACTTCCTTCAGAACTCATAGATTTGGAAAGTCAACTGCTGAGATTTTGAACAAACTGTTTGACCTTAATATAGTATCAGTTAAGAAAGAAGACGACATCGTTGAATTCTTTAACCCTTTTGGAGTTGACCCCACTGAGCATATCTCTGCCTCTGAAGAAGAAGTCTTGGACTTTGTTTCTGACCAAGTGGTGACCACTAGCTCAGAGGAACTAGCAGGAATTGAGTTTGCAGAAACAACTTTCTACTGCACAACATTGGCCGCAGCTGTTGCTGAAAATCCTGCTAAGACTTTCATCTCTCTGACTAGACACACCCACAAACTCACCATTGGGGAACTAAATGCCAGGTCTAACTCCTAGATGCCAGGTCTAACTCCTAGAGCTGACCTCACTGACACATACAAAATCATTGCCATTGCTTTCTTGTTGTCAGCTTGCATTTACTTCCAAAATAGCCACTACCAACCTGTTGCTGGAGACAACTTGCACCGTTTGCCTTTTGGTGGCCAATATCAAGACGGCACCAAAAAGATATCTTATTTTCCACAACAGCAGTCATACTTTCATTCTGGAAACAAATTAAATGTCCTCATACTTATCTTCATTCTCACGTTGGGTATTGTCCTCACCAATAAATTTAGTTTTAGCTTTAGTCGTACTACTCACCAGCATTCTTGCTATAACACACATTCAGCAACCAACAATACACAACCATTGTCAGGTCATCATTGAATGTCCTCATACTTATCTTCATTCTCACGTTGGGTATTGTCCTCACCAATAAATTTAGTTTTAGCTTTAGTCGTACTACTCACCAGCATTCTTGCTATAACACACATTCAGCAACCAACAATACACAACCATTGTCAGGTCATCATTGACGGTTCTGCAATAGTCATAACAAATTGTGAGAACACACCAGAAGTGCTTAAAGCAATAAACTTCTCCCCTTGGAACGGGTTAAGTTTTCCTAAATTTGAAAATTAAATGCCTGACACAACACCTGTTGCTGCCACTTCAAGTGCACCACCCACAGCCAAAGATGCTGGTGCCAAAGCTCCTTCTGACTTCTCAAATCCCAATACAGCTCCTAGTCTCAGTGATTTGAAGAAAGTCAAGTATGTCTCCACCGTGACCTCCGTGGCCACACCAGCTGAAATTGAAGCCCTAGGCAAAATCTTCACCGCCATGGGCCTTGCCGCCAATGAGACTGGCCCGGCCATGTGGGATCTAGCTCGTGCATATGCTGATGTGCAGAGTTCTAAATCGGCACAGCTGATTGGAGCTACCCCTTCCAACCCTGCACTATCACGCCGAGCCCTTGCTGCTCAGTTTGATCGAATCAATATAACCCCCAGGCAATTTTGCATGTACTTTGCCAAAGTTGTTTGGAACATACTTCTCGACAGCAACATTCCACCAGCAAATTGGGCCAAACTTGGTTACCAAGAAGATACAAAATTTGCTGCATTTGACTTCTTCGATGGAGTCACCAACCCTGCCAGCCTGCAGCCTGCTGATGGTCTTATCAGGCAGCCAAATGAGAAAGAACTAGCTGCTCACTCCGTAGCTAAGTACGGCGCCTTGGCTAGGCAAAAGATCTCCACAGGTAATTATATTACCACACTTGGAGAAGTCACACGTGGACACATGGGAGGAGCTAACACCATGTACGCGATAGACGCACCCCCTAAACTTTAA

>H30_P8_c3

ATGGAAAGATCAACTCTGATTAATTTACTTCAATTGCACCACTTCGAGCCAAAACTCAGTGTTGAAGGAATCATAGTTGTGCACGGAATTGCAGGCACTGGGAAAACCACTTTACTTAGGACTTTATTTTCTGCTTACCCTAGCTTAGTTATAGGTTCACCTAGGCCTTGCTATTTAGATAAACAAAACAAAATTTCACAAGTTTGCTTATCTTGCTTTCCCAATACCCATTGTGATATTGTCGATGAGTATCATTTGCTAGAAAGTTTTCTAGAACCAAAATTGGCTATCTTTGGTGACCCCTGTCAATGCACATACATTGAGAGACTTAGAGTCCCACATTACACTTCCTTCAGAACTCATAGATTTGGAAAGTCGACTGCTGAGATTTTGAACAAACTGTTTGACCTTAATATAGTCTCAGTTAAGAAAGAAGACGACATCGTTGAATTCTTTAACCCTTTTGAAGTTGACCCCACTGAGCATATCTCTGCCTCTGAAGAAGAAGTCTTGGACTTTGTTTCTGACCAAGTGGTGACCACTAGCTCAGAGGAACTAGCAGGAATTGAGTTTGCAGAAACAACTTTCTACTGCACAACATTGGCCGCAGCTGTTGCTGAAAATCCTGCTAAGACTTTCATCTCTCTGACTAGACACACCCACAAACTCACCATTGGGGAACTAAATGCCAGGTCTAACTCCTAGATGCCAGGTCTAACTCCTAGAGCTGACCTCACTGACACATACAAAATCATTGCCATTGCTTTCTTGTTGTCAGCTTGCATTTACTTCCAAAATAGCCACTACCAACCTGTTGCTGGAGACAACTTGCACCGTTTGCCTTTTGGTGGCCAATATCAAGACGGCACCAAAAAGATATCTTATTTTCCACAACAGCAGTCATACTTTCATTCTGGAAACAAATTAAATGTCCTCATACTTATCTTCATTCTCACGTTGGGTATTGTCCTCACCAATAAATTTAGTTTTAGCTTTAGTCGTACTACTCACCAGCATTCTTGCTATAACACACATTCAGCAACCAACAATACACAACCATTGTCAGGTCATCATTGAATGTCCTCATACTTATCTTCATTCTCACGTTGGGTATTGTCCTCACCAATAAATTTAGTTTTAGCTTTAGTCGTACTACTCACCAGCATTCTTGCTATAACACACATTCAGCAACCAACAATACACAACCATTGTCAGGTCATCATTGACGGTTCTGCAATAGTCATAACAAATTGTGAGAACACACCAGAAGTGCTTAAAGCAATAAACTTCTCCCCTTGGAACGGGTTAAGTTTTCCTAAATTTGAAAATTAAATGCCTGACACAACACCTGTTGCTGCCACTTCAAGTGCACCACCCACAGCCAAAGATGCTGGTGCCAAAGCTCCTTCTGACTTCTCAAATCCCAATACAGCTCCTAGTCTCAGTGATTTGAAGAAAGTCAAGTATGTCTCCACCGTGACCTCCGTGGCCACACCAGCTGAAATTGAAGCCCTAGGCAAAATCTTCACCGCCATGGGCCTTGCCGCCAATGAGACTGGTCCGGCCATGTGGGATCTAGCTCGTGCATATGCTGATGTGCAGAGTTCTAAATCGGCACAGCTGATTGGAGCTACCCCTTCCAACCCTGCACTATCACGCCGAGCCCTTGCTGCTCAGTTTGATCGAATCAATATAACCCCCAGGCAATTTTGCATGTACTTTGCCAAAGTTGTTTGGAACATACTTCTCGACAGCAACATTCCACCAGCAAATTGGGCCAAACTTGGTTACCAAGAAGATACAAAATTTGCTGCATTTGACTTCTTCGATGGAGTCGCCAACCCTGCCAGCCTGCAGCCTGCTGATGGTCTTATCAGGCAGCCAAATGAGAAAGGACTAGCTGCTCACTCCGTAGCTAAGTACGGCGCCTTGGCTAGGCAAAAGATCTCCACAGGTAATTATATTACCACACTTGGAGAAGTCACACGTGGACACATGGGAGGAGCTAACACCATGTGCGCGATAGACGCACCCCCTAAACTTTAA

>H30_P8_c2

ATGGAAAGATCAACTCTGATTAATTTACTTCAATTGCACCACTTCGAGCCAAAACTCAGTGTTGAAGGAATCATAGTTGTGCACGGAATTGCAGGCACTGGGAAAACCACTTTACTTAGGACTTTATTTTCTGCTTACCCTAGCTTAGTTATAGGTTCACCTAGGCCTTGCTATTTAGATAAACAAAACAAAATTTCACAAGTTTGCTTATCTTGCTTTCCCAATACCCATTGTGATATTGTCGATGAGTATCATTTGCTAGAAAGTTTTCTAGAACCAAAATTGGCTATCTTTGGTGACCCCTGTCAATGCACATACATTGAGAGACTTAGAGTCCCACATTACACTTCCTTCAGAACTCATAGATTTGGAAAGTCGACTGCTGAGATTTTGAACAAACTGTTTGACCTTAATATAGTCTCAGTTAAGAAAGAAGACGACATCGTTGAATTCTTTAACCCTTTTGAAGTTGACCCCACTGAGCATATCTCTGCCTCTGAAGAAGAAGTCTTGGACTTTGTTTCTGACCAAGTGGTGACCACTAGCTCAGAGGAACTAGCAGGAATTGAGTTTGCAGAAACAACTTTCTACTGCACAACATTGGCCGCAGCTGTTGCTGAAAATCCTGCTAAGACTTTCATCTCTCTGACTAGACACACCCACAAACTCACCATTGGGGAACTAAATGCCAGGTCTAACTCCTAGATGCCAGGTCTAACTCCTAGAGCTGACCTCACTGACACATACAAAATCATTGCCATTGCTTTCTTGTTGTCAGCTTGCATTTACTTCCAAAATAGCCACTACCAACCTGTTGCTGGAGACGACTTGCACCGTTTGCCTTTTGGTGGCCAATATCAAGACGGCACCAAAAAGATATCTTATTTTCCACAACAGCAGTCATACTTTCATTCTGGAGACAAATTAAATGTCCTCATACTTATCTTCATTCTCACGTTGGGTATTGTCCTCACCAATAAATTTAGTTTTAGCTTTAGTCGTACTACTCACCAGCATTCTTGCTATAACACACATTCAGCAACCAACAATACACAACCATTGTCAGGTCATCATTGAATGTCCTCATACTTATCTTCATTCTCACGTTGGGTATTGTCCTCACCAATAAATTTAGTTTTAGCTTTAGTCGTACTACTCACCAGCATTCTTGCTATAACACACATTCAGCAACCAACAATACACAACCATTGTCAGGTCATCATTGACGGTTCTGCAATAGTCATAACAAATTGTGAGAACACACCAGAAGTGCTTAAAGCAATAAACTTCTCCCCTTGGAACGGGTTAAGTTTTCCTAAATTTGAAAATTAAATGCCTGACACAACACCTGTTGCTGCCACTTCAAGTGCACCACCCACAGCCAAAGATGCTGGTGCCAAAGCTCCTTCTGACTTCTCAAATCCCAATACAGCTCCTAGTCTCAGTGATTTGAAGAAAGTCAAGTATGTCTCCACCGTGACCTCCGTGGCCACACCAGCTGAAATTGAAGCCCTAGGCAAAATCTTCACCGCCATGGGCCTTGCCGCCAATGAGACTGGCCCGGCCATGTGGGATCTAGCTCGTGCATATGCTGATGTGCAGAGTTCTAAATCGGCACAGCTGATTGGAGCTACCCCTTCCAACCCTGCACTATCACGCCGAGCCCTTGCTGCTCAGTTTGATCGAATCAATATAACCCCCAGGCAATTTTGCATGTACTTTGCCAAAGTTGTTTGGAACATACTTCTCGACAGCAACATTCCACCAGCAAATTGGGCCAAACTTGGTTACCAAGAAGATACAAAATTTGCTGCATTTGACTTCTTCGATGGAGTCACCAACCCTGCCAGCCTGCAGCCTGCTGATGGTCTTATCAGGCAGCCAAATGAGAAAGAACTAGCTGCTCACTCCGTAGCTAAGTACGGCGCCTTGGCTAGGCAAAAGATCTCCACAGGTAATTATATTACCACACTTGGAGAAGTCACACGTGGACACATGGGAGGAGCTAACACCATGTACGCGATAGACGCACCCCCTAAACTTTAA

>H30_P8_c1

ATGGAAAGATCAACTCTGATTAATTTACTTCAATTGCACCACTTCGAGCCAAAACTCAGTGTTGAAGGAATCATAGTTGTGCACGGAATTGCAGGCACTGGGAAAACCACTTTACTTAGGACTTTATTTTCTGCTTACCCTAGCTTAGTTATAGGTTCACCTAGGCCTTGCTATTTAGATAAACAAAACAAAATTTCACAAGTTTGCTTATCTTGCTTTCCCAATACCCATTGTGATATTGTCGATGAGTATCATTTGCTAGAAAGTTTTCTAGAACCAAAATTGGCTATCTTTGGTGACCCCTGTCAATGCACATACATTGAGAGACTTAGAGTCCCACATTACACTTCCTTCAGAACTCATAGATTTGGAAAGTCAACTGCTGAGATTTTGAACAAACTGTTTGACCTTAATATAGTATCAGTTAAGAAAGAAGACGACATCGTTGAATTCTTTAACCCTTTTGAAGTTGACCCCACTGAGCATATCTCTGCCTCTGAAGAAGAAGTCTTGGACTTTGTTTCTGACCAAGTGGTGACCACTAGCTCAGAGGAACTAGCAGGAATTGAGTTTGCAGAAACAACTTTCTACTGCACAACATTGGCCGCAGCTGTTGCTGAAAATCCTGCTAAGACTTTCATCTCTCTGACTAGACACACCCACAAACTCACCATTGGGGAACTAAATGCCAGGTCTAACTCCTAGATGCCAGGTCTAACTCCTAGAGCTGACCTCACTGACACATACAAAATCATTGCCATTGCTTTCTTGTTGTCAGCTTGCATTTACTTCCAAAATAGCCACTACCAACCTGTTGCTGGAGACAACTTGCACCGTTTGCCTTTTGGTGGCCAATATCAAGACGGCACCAAAAAGATATCTTATTTTCCACAACAGCAGTCATACTTTCATTCTGGAAACAAATTAAATGTCCTCATACTTATCTTCATTCTCACGTTGGGTATTGTCCTCACCAATAAATTTAGTTTTAGCTTTAGTCGTACTACTCACCAGCATTCTTGCTATAACACACATTCAGCAACCAACAATACACAACCATTGTCAGGTCATCATTGAATGTCCTCATACTTATCTTCATTCTCACGTTGGGTATTGTCCTCACCAATAAATTTAGTTTTAGCTTTAGTCGTACTACTCACCAGCATTCTTGCTATAACACACATTCAGCAACCAACAATACACAACCATTGTCAGGTCATCATTGACGGTTCTGCAATAGTCATAACAAATTGTGAGAACACACCAGAAGTGCTTAAAGCAATAAACTTCTCCCCTTGGAACGGGTTAAGTTTTCCTAAATTTGAAAATTAAATGCCTGACACAACACCTGTTGCTGCCACTTCAAGTGCACCACCCACAGCCAAAGATGCTGGTGCCAAAGCTCCTTCTGACTTCTCAAATCCCAATACAGCTCCTAGTCTCAGTGATTTGAAGAAAGTCAAGTATGTCTCCACCGTGACCTCCGTGGCCACACCAGCTGAAATTGAAGCCCTAGGCAAAATCTTCACCGCCATGGGCCTTGCCGCCAATGAGACTGGCCCGGCCATGTGGGATCTAGCTCGTGCATATGCTGATGTGCAGAGTTCTAAATCGGCACAGCTGATTGGAGCTACCCCTTCCAACCCTGCACTATCACGCCGAGCCCTTGCTGCTCAGTTTGATCGAATCAATATAACCCCCAGGCAATTTTGCATGTACTTTGCCAAAGTTGTTTGGAACATACTTCTCGACAGCAACATTCCACCAGCAAATTGGGCCAAACTTGGTTACCAAGAAGATACAAAATTTGCTGCATTTGACTTCTTCGATGGAGTCACCAACCCTGCCAGCCTGCAGCCTGCTGATGGTCTTATCAGGCAGCCAAATGAGAAAGAACTAGCTGCTCACTCCGTAGCTAAGTACGGCGCCTTGGCTAGGCAAAAGATCTCCACAGGTAATTATATTACCACACTTGGAGAAGTCACACGTGGACACATGGGAGGAGCTAACACCATGTGCGCGATAGACGCACCCCCTAAACTTTAA

>H30_P8_b14

ATGGAAAGATCAACTCTGATTAATTTACTTCAATTGCACCACTTCGAGCCAAAACTCAGTGTTGAAGGAATCATAGTTGTGCACGGAATTGCAGGCACTGGGAAAACCACTTTACTTAGGACTTTATTTTCTGCTTACCCTAGCTTAGTTATAGGTTCACCTAGGCCTTGCTATTTAGATAAACAAAACAAAATTTCACAAGTTTGCTTATCTTGCTTTCCCAATACCCATTGTGATATTGTCGATGAGTATCATTTGCTAGAAAGTTTTCTAGAACCAAAATTGGCTATCTTTGGTGACCCCTGTCAATGCACATACATTGAGAGACTTAGAGTCCCACATTACACTTCCTTCAGAACTCATAGATTTGGAAAGTCAACTGCTGAGATTTTGAACAAACTGTTTGACCTTAATATAGTCTCAGTTAAGAAAGAAGACGACATCGTTGAATTCTTTAACCCTTTTGAAGTTGACCCCACTGAGCATATCTCTGCCTCTGAAGAAGAAGTCTTGGACTTTGTTTCTGACCAAGTGGTGACCACTAGCTCAGAGGAACTAGCAGGAATTGAGTTTGCAGAAACAACTTTCTACTGCATAACATTGGCCGCAGCTGTTGCTGAAAATCCTGCTAAGACTTTCATCTCTCTGACTAGACACACCCACAAACTCACCATTGGGGAACTAAATGCCAGGTCTAACTCCTAGATGCCAGGTCTAACTCCTAGAGCTGACCTCACTGACACATACAAAATCATTGCCATTGCTTTCTTGTTGTCAGCTTGCATTTACTTCCAAAATAGCCACTACCAACCTGTTGCTGGAGACAACTTGCACCGTTTGCCTTTTGGTGGCCAATATCAAGACGGCACCAAAAAGATATCTTATTTTCCACAACAGCAGTCATACTTTCATTCTGGAAACAAATTAAATGTCCTCATACTTATCTTCATTCTCACGTTGGGTATTGTCCTCACCAATAAATTTAGTTTTAGCTTTAGTCGTACTACTCACCAGCATTCTTGCTATAACACACATTCAGCAACCAACAATACACAACCATTGTCAGGTCATCATTGAATGTCCTCATACTTATCTTCATTCTCACGTTGGGTATTGTCCTCACCAATAAATTTAGTTTTAGCTTTAGTCGTACTACTCACCAGCATTCTTGCTATAACACACATTCAGCAACCAACAATACACAACCATTGTCAGGTCATCATTGACGGTTCTGCAATAGTCATAACAAATTGTGAGAACACACCAGAAGTGCTTAAAGCAATAAACTTCTCCCCTTGGAACGGGTTAAGTTTTCCTAAATTTGAAAATTAAATGCCTGACACAACACCTGTTGCTGCCACTTCAAGTGCACCACCCACAGCCAAAGATGCTGGTGCCAAAGCTCCTTCTGACTTCTCAAATCCCAATACAGCTCCTAGTCTCAGTGATTTGAAGAAAGTCAAGTATGTCTCCACCGTGACCTCCGTGGCCACACCAGCTGAAATTGAAGCCCTAGGCAAAATCTTCACCGCCATGGGCCTTGCCGCCAATGAGACTGGTCCGGCCATGTGGGATCTAGCTCGTGCATATGCTGATGTGCAGAGTTCTAAATCGGCACAGCTGATTGGAGCTACCCCTTCCAACCCTGCACTATCACGCCGAGCCCTTGCTGCTCAGTTTGATCGAATCAATATAACCCCCAGGCAATTTTGCATGTACTTTGCCAAAGTTGTTTGGAACATACTTCTCGACAGCAACATTCCACCAGCAAATTGGGCCAAACTTGGTTACCAAGAAGATACAAAATTTGCTGCATTTGACTTCTTCGATGGAGTCACCAACCCTGCCAGCCTGCAGCCTGCTGATGGTCTTATCAGGCAGCCAAATGAGAAAGAACTAGCTGCTCACTCCGTAGCTAAGTACGGCGCCTTGGCTAGGCAAAAGATCTCCACAGGTAATTATATTACCACACTTGGAGAAGTCACACGTGGACACATGGGAGGAGCTAACACCATGTACGCGATAGACGCACCCCCTAAACTTTAA

>H30_P8_b13

ATGGAAAGATCAACTCTGATTAATTTACTTCAATTGCACCACTTCGAGCCAAAACTCAGTGTTGAAGGAATCATAGTTGTGCACGGAATTGCAGGCACTGGGAAAACCACTTTACTTAGGACTTTATTTTCTGCTTACCCTAGCTTAGTTATAGGTTCACCTAGGCCTTGCTATTTAGATAAACAAAACAAAATTTCACAAGTTTGCTTATCTTGCTTTCCCAATACCCATTGTGATATTGTCGATGAGTATCATTTGCTAGAAAGTTTTCTAGAACCAAAATTGGCTATCTTTGGTGACCCCTGTCAATGCACATACATTGAGAGACTTAGAGTCCCACATTACACTTCCTTCAGAACTCATAGATTTGGAAAGTCAACTGCTGAGATTTTGAACAAACTGTTTGACCTTAATATAGTCTCAGTTAAGAAAGAAGACGACATCGTTGAATTCTTTAACCCTTTTGAAGTTGACCCCACTGAGCATATCTCTGCCTCTGAAGAAGAAGTCTTGGACTTCGTTTCTGACCAAGTGGTGACCACTAGCTCAGAGGAACTAGCAGGAATTGAGTTTGCAGAAACAACTTTCTACTGCACAACATTGGCCGCAGCTGTTGCTGAAAATCCTGCTAAGACTTTCATCTCTCTGACTAGACACACCCACAAACTCACCATTGGGGAACTAAATGCCAGGTCTAACTCCTAGATGCCAGGTCTAACTCCTAGAGCTGACCTCACTGACACATACAAAATCATTGCCATTGCTTTCTTGTTGTCAGCTTGCATTTACTTCCAAAATAGCCACTACCAACCTGTTGCTGGAGACAACTTGCACCGTTTGCCTTTTGGTGGCCAATATCAAGACGGCACCAAAAAGATATCTTATTTTCCACAACAGCAGTCATACTTTCATTCTGGAAACAAATTAAATGTCCTCATACTTATCTTCATTCTCACGTTGGGTATTGTCCTCACCAATAAATTTAGTTTTAGCTTTAGTCGTACTACTCACCAGCATTCTTGCTATAACACACATTCAGCAACCAACAATACACAACCATTGTCAGGTCATCATTGAATGTCCTCATACTTATCTTCATTCTCACGTTGGGTATTGTCCTCACCAATAAATTTAGTTTTAGCTTTAGTCGTACTACTCACCAGCATTCTTGCTATAACACACATTCAGCAACCAACAATACACAACCATTGTCAGGTCATCATTGACGGTTCTGCAATAGTCATAACAAATTGTGAGAACACACCAGAAGTGCTTAAAGCAATAAACTTCTCCCCTTGGAACGGGTTAAGTTTTCCTAAATTTGAAAATTAAATGCCTGACACAACACCTGTTGCTGCCACTTCAAGTGCACCACCCACAGCCAAAGATGCTGGTGCCAAAGCTCCTTCTGACTTCTCAAATCCCAATACAGCTCCTAGTCTCAGTGATTTGAAGAAAGTCAAGTATGTCTCCACCGTGACCTCCGTGGCCACACCAGCTGAAATTGAAGCCCTAGGCAAAATCTTCACCGCCATGGGCCTTGCCGCCAATGAGACTGGTCCGGCCATGTGGGATCTAGCTCGTGCATATGCTGATGTGCAGAGTTCTAAATCGGCACAGCTGATTGGAGCTACCCCTTCCAACCCTGCACTATCACGCCGAGCCCTTGCTGCTCAGTTTGATCGAATCAATATAACCCCCAGGCAATTTTGCATGTACTTTGCCAAAGTTGTTTGGAACATACTTCTCGACAGCAACATTCCACCAGCAAATTGGGCCAAACTTGGTTACCAAGAAGATACAAAATTTGCTGCATTTGACTTCTTCGATGGAGTCACCAACCCTGCCAGCCTGCAGCCTGCTGATGGTCTTATCAGGCAGCCAAATGAGAAAGAACTAGCTGCTCACTCCGTAGCTAAGTACGGCGCCTTGGCTAGGCAAAAGATCTCCACAGGTAATTATATTACCACACTTGGAGAAGTCACACGTGGACACATGGGAGGAGCTAACACCATGTACGCGATAGACGCACCCCCTAAACTTTAA

>H30_P8_b12

ATGGAAAGATCAACTCTGATTAATTTACTTCAATTGCACCACTTCGAGCCAAAACTCAGTGTTGAAGGAATCATAGTTGTGCACGGAATTGCAGGCACTGGGAAAACCACTTTACTTAGGACTTTATTTTCTGCTTACCCTAGCTTAGTTATAGGTTCACCTAGGCCTTGCTATTTAGATAAACAAAACAAAATTTCACAAGTTTGCTTATCTTGCTTTCCCAATACCCATTGTGATATTGTCGATGAGTATCATTTGCTAGAAAGTTTTCTAGAACCAAAATTGGCTATCCTTGGTGACCCCTGTCAATGCACATACATTGAGAGACTTAGAGTCCCACATTACACTTCCTTCAGAACTCATAGATTTGGAAAGTCAACTGCTGAGATTTTGAACAAACTGTTTGACCTTAATATAGTCTCAGTTAAGAAAGAAGACGACATCGTTGAATTCTTTAACCCTTTTGAAGTTGACCCCACTGAGCATATCTCTGCCTCTGAAGAAGAAGTCTTGGACTTTGTTTCTGACCAAGTGGTGACCACTAGCTCAGAGGAACTAGCAGGAATTGAGTTTGCAGGAACAACTTTCTACTGCACAACATTGGCCGCAGCTGTTGCTGAAAATCCTGCTAAGACTTTCATCTCTCTGACTAGACACACCCACAAACTCACCATTGGGGAACTAAATGCCAGGTCTAACTCCTAGATGCCAGGTCTAACTCCTAGAGCTGACCTCACTGACACATACAAAATCATTGCCATTGCTTTCTTGTTGTCAGCTTGCATTTACTTCCAAAATAGCCACTACCAACCTGTTGCTGGAGACAACTTGCACCGTTTGCCTTTTGGTGGCCAGTATCAAGACGGCACCAAAAAGATATCTTATTTTCCACAACAGCAGTCATACTTTCATTCTGGAAACAAATTAAATGTCCTCATACTTATCTTCATTCTCACGTTGGGTATTGTCCTCACCAATAAATTTAGTTTTAGCTTTAGTCGTACTACTCACCAGCATTCTTGCTATAACACACATTCAGCAACCAACAATACACAACCATTGTCAGGTCATCATTGAATGTCCTCATACTTATCTTCATTCTCACGTTGGGTATTGTCCTCACCAATAAATTTAGTTTTAGCTTTAGTCGTACTACTCACCAGCATTCTTGCTATAACACACATTCAGCAACCAACAATACACAACCATTGTCAGGTCATCATTGACGGTTCTGCAATAGTCATAACAAATTGTGAGAACACACCAGAAGTGCTTAAAGCAATAAACTTCTCCCCTTGGAACGGGTTAAGTTTTCCTAAATTTGAAAATTAAATGCCTGACACAACACCTGTTGCTGCCACTTCAAGTGCACCACCCACAGCCAAAGATGCTGGTGCCAAAGCTCCTTCTGACTTCTCAAATCCCAATACAGCTCCTAGTCTCAGTGATTTGAAGAAAGTCAAGTATGTCTCCACCGTGACCTCCGTGGCCACACCAGCTGAAATTGAAGCCCTAGGCAAAATCTTCACCGCCATGGGCCTTGCCGCCAATGAGACTGGTCCGGCCATGTGGGATCTAGCTCGTGCATATGCTGATGTGCAGAGTTCTAAATCGGCACAGCTGATTGGAGCTACCCCTTCCAACCCTGCACTATCACGCCGAGCCCTTGCTGCTCAGTTTGATCGAATCAATATAACCCCCAGGCAATTTTGCATGTACTTTGCCAAAGTTGTTTGGAACATACTTCTCGACAGCAACATTCCACCAGCAAATTGGGCCAAACTTGGTTACCAAGAAGATACAAAATTTGCTGCATTTGACTTCTTCGATGGAGTCACCAACCCTGCCAGCCTGCAGCCTGCTGATGGTCTTATCAGGCAGCCAAATGAGAAAGAACTAGCTGCTCACTCCGTAGCTAAGTACGGCGCCTTGGCTAGGCAAAAGATCTCCACAGGTAATTATATTACCACACTTGGAGAAGTCACACGTGGACACATGGGAGGAGCTAACACCATGTACGCGATAGACGCACCCCCTAAACTTTAA

>H30_P8_b11

ATGGAAAGATCAACCCTGATTAATTTACTTCAATTGCACCACTTCGAGCCAAAACTCAGTGTTGAAGGAATCATAGTTGTGCACGGAATTGCAGGCACTGGGAAAACCACTTTACTTAGGACTTTATTTTCTGCTTACCCTAGCTTAGTTATAGGTTCACCTAGGCCTTGCTATTTAGATAAACAAAACAAAATTTCACAAGTTTGCTTATCTTGCTTTCCCAATACCCATTGTGATATTGTCGATGAGTATCATTTGCTAGAAAGTTTTCTAGAACCAAAATTGGCTATCTTTGGTGACCCCTGTCAATGCACATACATTGAGAGACTTAGAGTCCCACATTACACTTCCTTCAGAACTCATAGATTTGGAAAGTCAACTGCTGAGATTTTGAACAAACTGTTTGACCTTAATATAGTCTCAGTTAAGAAAGAAGACGACATCGTTGAATTCTTTAACCCTTTTGAAGTTGACCCCACTGAGCATATCTCTGCCTCTGAAGAAGAAGTCTTGGACTTTGTTTCTGACCAAGTGGTGACCACTAGCTCAGAGGAACTAGCAGGAATTGAGTTTGCAGAAACAACTTTCTACTGCACAACATTGGCCGCAGCTGTTGCTGAAAATCCTGCTAAGACTTTCATCTCTCTGACTAGACACACCCACAAACTCACCATTGGGGAACTAAATGCCAGGTCTAACTCCTAGATGCCAGGTCTAACTCCTAGAGCTGACCTCACTGACACATACAAAATCATTGCCATTGCTTTCTTGTTGTCAGCTTGCATTTACTTCCAAAATAGCCACTACCAACCTGTTGCTGGAGACAACTTGCACCGTTTGCCTTTTGGTGGCCAATATCAAGACGGCACCAAAAAGATATCTTATTTTCCACAACAGCAGTCATACTTTCATTCTGGAAACAAATTAAATGTCCTCATACTTATCTTCATTCTCACGTTGGGTATTGTCCTCACCAATAAATTTAGTTTTAGCTTTAGTCGTACTACTCACCAGCATTCTTGCTATAACACACATTCAGCAACCAACAATACACAACCATTGTCAGGTCATCATTGAATGTCCTCATACTTATCTTCATTCTCACGTTGGGTATTGTCCTCACCAATAAATTTAGTTTTAGCTTTAGTCGTACTACTCACCAGCATTCTTGCTATAACACACATTCAGCAACCAACAATACACAACCATTGTCAGGTCATCATTGACGGTTCTGCAATAGTCATAACAAATTGTGAGAACACACCAGAAGTGCTTAAAGCAATAAACTTCTCCCCTTGGAACGGGTTAAGTTTTCCTAAATTTGAAAATTAAATGCCTGACACAACACCTGTTGCTGCCACTTCAAGTGCACCACCCACAGCCAAAGATGCTGGTGCCAAAGCTCCTTCTGACTTCTCAAATCCCAATACAGCTCCTAGTCTCAGTGATTTGAAGAAAGTCAAGTATGTCTCCACCGTGACCTCCGTGGCCACACCAGCTGAAATTGAAGCCCTAGGCAAAATCTTCACCGCCATGGGCCTTGCCGCCAATGAGACTGGTCCGGCCATGTGGGATCTAGCTCGTGCATATGCTGATGTGCAGAGTTCTAAATCGGCACAGCTGATTGGAGCTACCCCTTCCAACCCTGCACTATCACGCCGAGCCCTTGCTGCTCAGTTTGATCGAATCAATATAACCCCCAGGCAATTTTGCATGTACTTTGCCAAAGTTGTTTGGAACATACTTCTCGACAGCAACATTCCACCAGCAAATTGGGCCAAACTTGGTTACCAAGAAGATACAAAATTTGCTGCATTTGACTTCTTCGATGGAGTCACCAACCCTGCCAGCCTGCAGCCTGCTGATGGTCTTATCAGGCAGCCAAATGAGAAAGAACTAGCTGCTCACTCCGTAGCTAAGTACGGCGCCTTGGCTAGGCAAAAGATCTCCACAGGTAATTATATTACCACACTTGGAGAAGTCACACGTGGACACATGGGAGGAGCTAACACCATGTACGCGATAGACGCACCCCCTAAACTTTAA

>H30_P8_b10

ATGGAAAGATCAACTCTGATTAATTTACTTCAATTGCACCACTTCGAGCCAAAACTCAGTGTTGAAGGAATCATAGTTGTGCACGGAATTGCAGGCACTGGGAAAACCACTTTACTTAGGACTTTATTTTCTGCTTACCCTAGCTTAGTTATAGGTTCACCTAGGCCTTGCTATTTAGATAAACAAAACAAAATTTCACAAGTTTGCTTATCTTGCTTACCCAATACCCATTGTGATATTGTCGATGAGTATCATTTGCTAGAAAGTTTTCTAGAACCAAAATTGGCTATCTTTGGTGACCCCTGTCAATGCACATACATTGAGAGACTTAGAGTCCCACATTACACTTCCTTCAGAACTCATAGATTTGGAAAGTCAACTGCTGAGATTTTGAACAAACTGTTTGACCTTAATATAGTCTCAGTTAAGAAAGAAGACGACATCGTTGAATTCTTTAACCCTTTTGAAGTTGACCCCACTGAGCATATCTCTGCCTCTGAAGAAGAAGTCTTGGACTTTGTTTCTGACCAAGTGGTGACCACTAGCTCAGAGGAACTAGCAGGAATTGAGTTTGCAGAAACAACTTTCTACTGCACAACATTGGCCGCAGCTGTTGCTGAAAATCCTGCTAAGACTTTCATCTCTCTGACTAGACACACCCACAAACTCACCATTGGGGAACTAAATGCCAGGTCTAACTCCTAGATGCCAGGTCTAACTCCTAGAGCTGACCTCACTGACACATACAAAATCATTGCCATTGCTTTCTTGTTGTCAGCTTGCATTTACTTCCAAAATAGCCACTACCAACCTGTTGCTGGAGACAACTTGCACCGTTTGCCTTTTGGTGGCCAATATCAAGACGGCACCAAAAAGATATCTTATTTTCCACAACAGCAGTCATACTTTCATTCTGGAAACAAATTAAATGTCCTCATACTTATCTTCATTCTCACGTTGGGTATTGTCCTCACCAATAAATTTAGTTTTAGCTTTAGTCGTACTACTCACCAGCATTCTTGCTATAACACACGTTCAGCAACCAACAATACACAACCATTGTCAGGTCATCATTGAATGTCCTCATACTTATCTTCATTCTCACGTTGGGTATTGTCCTCACCAATAAATTTAGTTTTAGCTTTAGTCGTACTACTCACCAGCATTCTTGCTATAACACACGTTCAGCAACCAACAATACACAACCATTGTCAGGTCATCATTGACGGTTCTGCAATAGTCATAACAAATTGTGGGAACACACCAGAAGTGCTTAAAGCAATAAACTTCTCCCCTTGGAACGGGTTAAGTTTTCCTAAATTTGAAAATTAAATGCCTGACACAACACCTGTTGCTGCCACTTCAAGTGCACCACCCACAGCCAAAGATGCTGGTGCCAAAGCTCCTTCTGACTTCTCAAATCCCAATACAGCTCCTAGTCTCAGTGATTTGAAGAAAGTCAAGTATGTCTCCACCGTGACCTCCGTGGCCACACCAGCTGAAATTGAAGCCCTAGGCAAAATCTTCACCGCCATGGGCCTTGCCGCCAATGAGACTGGTCCGGCCATGTGGGATCTAGCTCGTGCATATGCTGATGTGCAGAGTTCTAAATCGGCACAGCTGATTGGAGCTACCCCTTCCAACCCTGCACTATCACGCCGAGCCCTTGCTGCTCAGTTTGATCGAATCAATATAACCCCCAGGCAATTTTGCATGTACTTTGCCAAAGTTGTTTGGAACATACTTCTCGACAGCAACATTCCACCAGCAAATTGGGCCAAACTTGGTTACCAAGAAGATACAAAATTTGCTGCATTTGACTTCTTCGATGGAGTCACCAACCCTGCCAGCCTGCAGCCTGCTGATGGTCTTATCAGGCAGCCAAATGAGAAAGAACTAGCTGCTCACTCCGTAGCTAAGTACGGCGCCTTGGCTAGGCAAAAGATCTCCACAGGTAATTATATTACCACACTTGGAGAAGTCACACGTGGACACATGGGAGGAGCTAACACCATGTACGCGATAGACGCACCCCCTAAACTTTAA

>H30_P8_b9

ATGGAAAGATCAACTCTGATTAATTTACTTCAATTGCACCACTTCGAGCCAAAACTCAGTGTTGAAGGAATCATAGTTGTGCACGGAATTGCAGGCACTGGGAAAACCACTTTACTTAGGACTTTATTTTCTGCTTACCCTAGCTTAGTTATAGGTTCACCTAGGCCTTGCTATTTAGATAAACAAAACAAAATTTCACAAGTTTGCTTATCTTGCTTTCCCAATACCCATTGTGATATTGTCGATGAGTATCATTTGCTAGAAAGTTTTCTAGAACCAAAATTGGCTATCTTTGGTGACCCCTGTCAATGCACATACATTGAGAGACTTAGAGTCCCACATTACACTTCCTTCAGAACTCATAGATTTGGAAAGTCAACTGCTGAGATTTTGAACAAACTGTTTGACCTTAATATAGTCTCAGTTAAGAAAGAAGACGACATCGTTGAATTCTTTAACCCTTTTGAAGTTGACCCCACTGAGCATATCTCTGCCTCTGAAGAAGAAGTCTTGGACTTTGTTTCTGACCAAGTGGTGACCACTAGCTCAGAGGAACTAGCAGGAATTGAGTTTGCAGAAACAACTTTCTACTGCACAACATTGGCCGCAGCTGTTGCTGAAAATCCTGCTAAGACTTTCATCTCTCTGACTAGACACACCCACAAACTCACCATTGGGGAACTAAATGCCAGGTCTAACTCCTAGATGCCAGGTCTAACTCCTAGAGCTGACCTCACTGACACATACAAAATCATTGCCATTGCTTTCTTGTTGTCAGCTTGCATTTACTTCCAAAATAGCCACTACCAACCTGTTGCTGGAGACAACTTGCACCGTTTGCCTTTTGGTGGCCAATATCAAGACGGCACCAAAAAGATATCTTATTTTCCACAACAGCAGTCATACTTTCATTCTGGAAACAAATTAAATGTCCTCATACTTATCTTCATTCTCACGTTGGGTATTGTCCTCACCAATAAATTTAGTTTTAGCTTTAGTCGTACTACTCACCAGCATTCTTGCTATAACACACATTCAGCAACCAACAATACACAACCATTGTCAGGTCATCATTGAATGTCCTCATACTTATCTTCATTCTCACGTTGGGTATTGTCCTCACCAATAAATTTAGTTTTAGCTTTAGTCGTACTACTCACCAGCATTCTTGCTATAACACACATTCAGCAACCAACAATACACAACCATTGTCAGGTCATCATTGACGGTTCTGCAATAGTCATAACAAATTGTGAGAACACACCAGAAGTGCTTAAAGCAATAAACTTCTCCCCTTGGAACGGGTTAAGTTTTCCTAAATTTGAAAATTAAATGCCTGACACAACACCTGTTGCTGCCACTTCAAGTGCACCACCCACAGCCAAAGATGCTGGTGCCAAAGCTCCTTCTGACTTCTCAAATCCCAATACAGCTCCTAGTCTCAGTGATTTGAAGAAAGTCAAGTATGTCTCCACCGTGACCTCCGTGGCCACACCAGCTGAAATTGAAGCCCTAGGCAAAATCTTCACCGCCATGGGCCTTGCCGCCAATGAGACTGGTCCGGCCATGTGGGATCTAGCTCGTGCATATGCTGATGTGCAGAGTTCTAAATCGGCACAGCTGATTGGAGCTACCCCTTCCAACCCTGCACTATCACGCCGAGCCCTTGCTGCTCAGTTTGATCGAATCAATATAACCCCCAGGCAATTTTGCATGTACTTTGCCAAAGTTGTTTGGAACATACTTCTCGACAGCAACATTCCACCAGCAAATTGGGCCAAACTTGGTTACCAAGAAGATACAAAATTTGCTGCATTTGACTTCTTCGATGGAGTCACCAACCCTGCCAGCCTGCAGCCTGCTGATGGTCTTATCAGGCAGCCAAATGAGAAAGAACTAGCTGCTCACTCCGTAGCTAAGTACGGCGCCTTGGCTAGGCAAAAGATCTCCACAGGTAATTATATTACCACACTTGGAGAAGTCACACGTGGACACATGGGAGGAGCTAACACCATGTACGCGATAGACGCACCCCCTAAACTTTAA

>H30_P8_b8

ATGGAAAGATCAACTCTGATTAATTTACTTCAATTGCACCACTTCGAGCCAAAACTCAGTGTTGAAGGAATCATAGTTGTGCACGGAATTGCAGGCACTGGGAAAACCACTTTACTTAGGACTTTATTTTCTGCTTACCCTAGCTTAGTTATAGGTTCACCTAGGCCTTGCTATTTAGATAAACAAAACAAAATTTCACAAGTTTGCTTATCTTGCTTTCCCAATACCCATTGTGATATTGTCGATGAGTATCATTTGCTAGAAAGTTTTCTAGAACCAAAATTGGCTATCTTTGGTGACCCCTGTCAATGCACATACATTGAGAGACTTAGAGTCCCACATTACACTTCCTTCAGAACTCATAGATTTGGAAAGTCAACTGCTGAGATTTTGAACAAACTGTTTGACCTTAATATAGTCTCAGTTAAGAAAGAAGACGACATCGTTGAATTCTTTAACCCTTTTGAAGTTGACCCCACTGAGCATATCTCTGCCTCTGAAGAAGAAGTCTTGGACTTTGTTTCTGACCAAGTGGTGACCACTAGCTCAGAGGAACTAGCAGGAATTGAGTTTGCAGAAACAACTTTCTACTGCACAACATTGGCCGCAGCTGTTGCTGAAAATCCTGCTAAGACTTTCATCTCTCTGACTAGACACACCCACAAACTCACCATTGGGGAACTAAATGCCAGGTCTAACTCCTAGATGCCAGGTCTAACTCCTAGAGCTGACCTCACTGACACATACAAAATCATTGCCATTGCTTTCTTGTTGTCAGCTTGCATTTACTTCCAAAATAGCCACTACCAACCTGTTGCTGGAGACAACTTGCACCGTTTGCCTTTTGGTGGCCAATATCAAGACGGCACCAAAAAGATATCTTATTTTCCACAACAGCAGTCATACTTTCATTCTGGAAACAAATTAAATGTCCTCATACTTATCTTCATTCTCACGTTGGGTATTGTCCTCACCAATAAATTTAGTTTTAGCTTTAGTCGTACTACTCACCAGCATTCTTGCTATAACACACATTCAGCAACCAACAATACACAACCATTGTCAGGTCATCATTGAATGTCCTCATACTTATCTTCATTCTCACGTTGGGTATTGTCCTCACCAATAAATTTAGTTTTAGCTTTAGTCGTACTACTCACCAGCATTCTTGCTATAACACACATTCAGCAACCAACAATACACAACCATTGTCAGGTCATCATTGACGGTTCTGCAATAGTCATAACAAATTGTGAGAACACACCAGAAGTGCTTAAAGCAATAAACTTCTCCCCTTGGAACGGGTTAAGTTTTCCTAAATTTGAAAATTAAATGCCTGACACAACACCTGTTGCTGCCACTTCAAGTGCACCACCCACAGCCAAAGATGCTGGTGCCAAAGCTCCTTCTGACTTCTCAAATCCCAATACAGCTCCTAGTCTCAGTGATTTGAAGAAAGTCAAGTATGTCTCCACCGTGACCTCCGTGGCCACACCAGCTGAAATTGAAGCCCTAGGCAAAATCTTCACCGCCATGGGCCTTGCCGCCAATGAGACTGGTCCGGCCATGTGGGATCTAGCTCGTGCATATGCTGATGTGCAGAGTTCTAAATCGGCACAGCTGATTGGAGCTACCCCTTCCAACCCTGCACTATCACGCCGAGCCCTTGCTGCTCAGTTTGATCGAATCAATATAACCCCCAGGCAATTTTGCATGTACTTTGCCAAAGTTGTTTGGAACATACTTCTCGACAGCAACATTCCACCAGCAAATTGGGCCAAACTTGGTTACCAAGAAGATACAAAATTCGCTGCATTTGACTTCTTCGATGGAGTCACCAACCCTGCCAGCCTGCAGCCTGCTGATGGTCTTATCAGGCAGCCAAATGAGAAAGAACTAGCTGCTCACTCCGTAGCTAAGTACGGCGCCTTGGCTAGGCAAAAGATCTCCACAGGTAATTATATTACCACACTTGGAGAAGTCACACGTGGACACATGGGAGGAGCTAACACCATGTACGCGATAGACGCACCCCCTAAACTTTAA

>H30_P8_b7

ATGGAAAGATCAACTCTGATTAATTTACTTCAATTGCACCACTTCGAGCCAAAACTCAGTGTTGAAGGAATCATAGTTGTGCACGGAATTGCAGGCACTGGGAAAACCACTTTACTTAGGACTTTATTTTCTGCTTACCCTAGCTTAGTTATAGGTTCACCTAGGCCTTGCTATTTAGATAAACAAAACAAAATTTCACAAGTTTGCTTATCTTGCTTTCCCAATACCCATTGTGATATTGTCGATGAGTATCATTTGCTAGAAAGTTTTCTAGAACCAAAATTGGCTATCTTTGGTGACCCCTGTCAATGCACATACATTGAGAGACTTAGAGTCCCACATTACACTTCCTTCAGAACTCATAGATTTGGAAAGTCAACTGCTGAGATTTTGAACAAACTGTTTGACCTTAATATAGTCTCAGTTAAGAAAGAAGACGACATCGTTGAATTCTTTAACCCTTTTGAAGTTGACCCCACTGAGCATATCTCTGCCTCTGAAGAAGAAGTCTTGGACTTTGTTTCTGACCAAGTGGTGACCACTAGCTCAGAGGAACTAGCAGGAATTGAGTTTGCAGAAACAACTTTCTACTGCACAACATTGGCCGCAGCTGTTGCTGAAAATCCTGCTAAGACTTTCATCTCTCTGACTAGACACACCCACAAACTCACCATTGGGGAACTAAATGCCAGGTCTAACTCCTAGATGCCAGGTCTAACTCCTAGAGCTGACCTCACTGACACATACAAAATCATTGCCATTGCTTTCTTGTTGTCAGCTTGCATTTACTTCCAAAATAGCCACTACCAACCTGTTGCTGGAGACAACTTGCACCGTTTGCCTTTTGGTGGCCAATATCAAGACGGCACCAAAAAGATATCTTACTTTCCACAACAGCAGTCATACTTTCATTCTGGAAACAAATTAAATGTCCTCATACTTATCTTCATTCTCACGTTGGGTATTGTCCTCACCAATAAATTTAGTTTTAGCTTTAGTCGTACTACTCACCAGCATTCTTGCTATAACACACATTCAGCAACCAACAATACACAACCATTGTCAGGTCATCATTGAATGTCCTCATACTTATCTTCATTCTCACGTTGGGTATTGTCCTCACCAATAAATTTAGTTTTAGCTTTAGTCGTACTACTCACCAGCATTCTTGCTATAACACACATTCAGCAACCAACAATACACAACCATTGTCAGGTCATCATTGACGGTTCTGCAATAGTCATAACAAATTGTGAGAACACACCAGAAATGCTTAAAGCAATAAACTTCTCCCCTTGGAACGGGTTAAGTTTTCCTAAATTTGAAAATTAAATGCCTGACACAACACCTGTTGCTGCCACTTCAAGTGCACCACCCACAGCCAAAGATGCTGGTGCCAAAGCTCCTTCTGACTTCTCAAATCCCAATACAGCTCCTAGTCTCAGTGATTTGAAGAAAGTCAAGTATGTCTCCACCGTGACCTCCGTGGCCACACCAGCTGAAATTGAAGCCCTAGGCAAAATCTTCACCGCCATGGGCCTTGCCGCCAATGAGACTGGTCCGGCCATGTGGGATCTAGCTCGTGCATATGCTGATGTGCAGAGTTCTAAATCGGCACAGCTGATTGGAGCTACCCCTTCCAACCCTGCACTATCACGCCGAGCCCTTGCTGCTCAGTTTGATCGAATCAATATAACCCCCAGGCAATTTTGCATGTACTTTGCCAAAGTTGTTTGGAACATACTTCTCGACAGCAACATTCCACCAGCAAATTGGGCCAAACTTGGTTACCAAGAAGATACAAAATTTGCTGCATTTGACTTCTTCGATGGAGTCACCAACCCTGCCAGCCTGCAGCCTGCTGATGGTCTTATCAGGCAGCCAAATGAGAAAGAACTAGCTGCTCACTCCGTAGCTAAGTACGGCGCCTTGGCTAGGCAAAAGATCTCCACAGGTAATTATATTACCACACTTGGAGAAGTCACACGTGGACACATGGGAGGAGCTAACACCATGTACGCGATAGACGCACCCCCTAAACTTTAA

>H30_P8_b6

ATGGAAAGATCAACTCTGATTAATTTACTTCAATTGCACCACTTCGAGCCAAAACTCAGTGTTGAAGGAATCATAGTTGTGCACGGAATTGCAGGCACTGGGAAAACCACTTTACTTAGGACTTTATTTTCTGCTTACCCTAGCTTAGTTATAGGTTCACCTAGGCCTTGCTATTTAGATAAACAAAACAAAATTTCACAAGTTTGCTTATCTTGCTTTCCCAATACCCATTGTGATATTGTCGATGAGTATCATTTGCTAGAAAGTTTTCTAGAACCAAAATTGGCTATCTTTGGTGACCCCTGTCAATGCACATACATTGAGAGACTTAGAGTCCCACATTACACTTCCTTCAGAACTCATAGATTTGGAAAGTCAACTGCTGAGATTTTGAACAAACTGTTTGACCTTAATATAGTCTCAGTTAAGAAAGAAGACGACATCGTTGAATTCTTTAACCCTTTTGAAGTTGACCCCACTGAGCATATCTCTGCCTCTGAAGAAGAAGTCTTGGACTTTGTTTCTGACCAAGTGGTGACCACTAGCTCAGAGGAACTAGCAGGAATTGAGTTTGCAGAAACAACTTTCTACTGCACAACATTGGCCGCAGCTGTTGCTGAAAATCCTGCTAAGACTTTCATCTCTCTGACTAGACACACCCACAAACTCACCATTGGGGAACTAAATGCCAGGTCTAACTCCTAGATGCCAGGTCTAACTCCTAGAGCTGACCTCACTGACACATACAAAATCATTGCCATTGCTTTCTTGTTGTCAGCTTGCATTTACTTCCAAAATAGCCACTACCAACCTGTTGCTGGAGACAACTTGCACCGTTTGCCTTTTGGTGGCCAATATCAAGACGGCACCAAAAAGATATCTTATTTTCCACAACAGCAGTCATACTTTCATTCTGGAAACAAATTAAATGTCCTCATACTTATCTTCATTCTCACGTTGGGTATTGTCCTCACCAATAAATTTAGTTTTAGCTTTAGTCGTACTACTCACCAGCATTCTTGCTATAACACACATTCAGCAACCAACAATACACAACCATTGTCAGGTCATCATTGAATGTCCTCATACTTATCTTCATTCTCACGTTGGGTATTGTCCTCACCAATAAATTTAGTTTTAGCTTTAGTCGTACTACTCACCAGCATTCTTGCTATAACACACATTCAGCAACCAACAATACACAACCATTGTCAGGTCATCATTGACGGTTCTGCAATAGTCATAACAAATTGTGAGAACACACCAGAAGTGCTTAAAGCAATAAACTTCTCCCCTTGGAACGGGTTAAGTTTTCCTAAATTTGAAAATTAAATGCCTGACACAACACCTGTTGCTGCCACTTCAAGTGCACCACCCACAGCCAAAGATGCTGGTGCCAAAGCTCCTTCTGACTTCTCAAATCCCAATACAGCTCCTAGTCTCAGTGATTTGAAGAAAGTCAAGTATGTCTCCACCGTGACCTCCGTGGCCACACCAGCTGAAATTGAAGCCCTAGGCAAAATCTTCACCGCCATGGGCCTTGCCGCCAATGAGACTGGTCCGGCCATGTGGGATCTAGCTCGTGCATATGCTGATGTGCAGAGTTCTAAATCGGCACAGCTGATTGGAGCTACCCCTTCCAACCCTGCACTATCACGCCGAGCCCTTGCTGCTCAGTTTGATCGAATCAATATAACCCCCAGGCAATTTTGCATGTACTTTGCCAAAGTTGTTTGGAACATACTTCTCGACAGCAACATTCCACCAGCAAATTGGGCCAAACTTGGTTACCAAGAAGATACAAAATTTGCTGCATTTGACTTCTTCGATGGAGTCACCAACCCTGCCAGCCTGCAGCCTGCTGATGGTCTTATCAGGCAGCCAAATGAGAAAGAACTAGCTGCTCACTCCGTAGCTAAGTACGGCGCCTTGGCTAGGCAAAAGATCTCCACAGGTAATTATATTACCACACTTGGAGAAGTCACACGTGGACACATGGGAGGAGCTAACACCATGTACGCGATAGACGCACCCCCTAAACTTTAA

>H30_P8_b5

ATGGAAAGATCAACTCTGATTAATTTACTTCAATTGCACCACTTCGAGCCAAAACTCAGTGTTGAAGGAATCATAGTTGTGCACGGAATTGCAGGCACTGGGAAAACCACTTTACTTAGGACTTTATTTTCTGCTTACCCTAGCTTAGTTATAGGTTCACCTAGGCCTTGCTATTTAGATAAACAAAACAAAATTTCACAAGTTTGCTTATCTTGCTTTCCCAATACCCATTGTGATATTGTCGATGAGTATCATTTGCTAGAAAGTTTTCTAGAACCAAAATTGGCTATCTTTGGTGACCCCTGTCAATGCACATACATTGAGAGACTTAGAGTCCCACATTACACTTCCTTCAGAACTCATAGATTTGGAAAGTCAACTGCTGAGATTTTGAACAAACTGTTTGACCTTAATATAGTCTCAGTTAAGAAAGAAGACGACATCGTTGAATTCTTTAACCCTTTTGAAGTTGACCCCACTGAGCATATCTCTGCCTCTGAAGAAGAAGTCTTGGACTTTGTTTCTGACCAAGTGGTGACCACTAGCTCAGAGGAACTAGCAGGAATTGAGTTTGCAGAAACAACTTTCTACTGCACAACATTGGCCGCAGCTGTTGCTGAAAATCCTGCTAAGACTTTCATCTCTCTGACTAGACACACCCACAAACTCACCATTGGGGAACTAAATGCCAGGTCTAACTCCTAGATGCCAGGTCTAACTCCTAGAGCTGACCTCACTGACACATACAAAATCATTGCCATTGCTTTCTTGTTGTCAGCTTGCATTTACTTCCAAAATAGCCACTACCAACCTGTTGCTGGAGACAACTTGCACCGTTTGCCTTTTGGTGGCCAATATCAAGACGGCACCAAAAAGATATCTTATTTTCCACAACAGCAGTCATACTTTCATTCTGGAAACAAATTAAATGTCCTCATACTTATCTTCATTCTCACGTTGGGTATTGTCCTCACCAATAAATTTAGTTTTAGCTTTAGTCGTACTACTCACCAGCATTCTTGCTATAACACACATTCAGCAACCAACAATACACAACCATTGTCAGGTCATCATTGAATGTCCTCATACTTATCTTCATTCTCACGTTGGGTATTGTCCTCACCAATAAATTTAGTTTTAGCTTTAGTCGTACTACTCACCAGCATTCTTGCTATAACACACATTCAGCAACCAACAATACACAACCATTGTCAGGTCATCATTGACGGTTCTGCAATAGTCATAACAAATTGTGAGAACACACCAGAAGTGCTTAAAGCAATAAACTTCTCCCCTTGGAACGGGTTAAGTTTTCCTAAATTTGAAAATTAAATGCCTGACACAACACCTGTTGCTGCCACTTCAAGTGCACCACCCACAGCCAAAGATGCTGGTGCCAAAGCTCCTTCTGACTTCTCAAATCCCAATACAGCTCCTAGTCTCAGTGATTTGAAGAAAGTCAAGTATGTCTCCACCGTGACCTCCGTGGCCACACCAGCTGAAATTGAAGCCCTAGGCAAAATCTTCACCGCCATGGGCCTTGCCGCCAATGAGACTGGTCCGGCCATGTGGGATCTAGCTCGTGCATATGCTGATGTGCAGAGTTCTAAATCGGCACAGCTGATTGGAGCTACCCCTTCCAACCCTGCACTATCACGCCGAGCCCTTGCTGCTCAGTTTGATCGAATCAATATAACCCCCAGGCAATTTTGCATGTACTTTGCCAAAGTTGTTTGGAACATACTTCTCGACAGCAACATTCCACCAGCAAATTGGGCCAAACTTGGTTACCAAGAAGATACAAAATTTGCTGCATTTGACTTCTTCGATGGAGTCACCAACCCTGCCAGCCTGCAGCCTGCTGATGGTCTTATCAGGCAGCCAAATGAGAAAGAACTAGCTGCTCACTCCGTAGCTAAGTACGGCGCCTTGGCTAGGCAAAAGATCTCCACAGGTAATTATATTACCACACTTGGAGAAGTCACACGTGGACACATGGGAGGAGCTAACACCATGTACGCGATAGACGCACCCCCTAAACTTTAA

>H30_P8_b4

ATGGAAAGATCAACTCTGATTAATTTACTTCAATTGCACCACTTCGAGCCAAAACTCAGTGTTGAAGGAATCATAGTTGTGCACGGAATTGCAGGCACTGGGAAAACCACTTTACTTAGGACTTTATTTTCTGCTTACCCTAGCTTAGTTATAGGTTCACCTAGGCCTTGCTATTTAGATAAACAAAACAAAATTTCACAAGTTTGCTTATCTTGCTTTCCCAATACCCATTGTGATATTGTCGATGAGTATCATTTGCTAGAAAGTTTTCTAGAACCAAAATTGGCTATCTTTGGTGACCCCTGTCAATGCACATACATTGAGAGACTTAGAGTCCCACATTACACTTCCTTCAGAACTCATAGATTTGGAAAGTCAACTGCTGAGATTTTGAACAAACTGTTTGACCTTAATATAGTCTCAGTTAAGAAAGAAGACGACATCGTTGAATTCTTTAACCCTTTTGAAGTTGACCCCACTGAGCATATCTCTGCCTCTGAAGAAGAAGTCTTGGACTTTGTTTCTGACCAAGTGGTGACCACTAGCTCAGAGGAACTAGCAGGAATTGAGTTTGCAGAAACAACTTTCTACTGCACAACATTGGCCGCAGCTGTTGCTGAAAATCCTGCTAAGACTTTCATCTCTCTGACTAGACACACCCACAAACTCACCATTGGGGAACTAAATGCCAGGTCTAACTCCTAGATGCCAGGTCTAACTCCTAGAGCTGACCTCACTGACACATACAAAATCATTGCCATTGCTTTCTTGTTGTCAGCTTGCATTTACTTCCAAAATAGCCACTACCAACCTGTTGCTGGAGACAACTTGCACCGTTTGCCTTTTGGTGGCCAATATCAAGACGGCACCAAAAAGATATCTTATTTTCCACAACAGCAGTCATACTTTCATTCTGGAAACAAATTAAATGTCCTCATACTTATCTTCATTCTCACGTTGGGTATTGTCCTCACCAATAAATTTAGTTTTAGCTTTAGTCGTACTACTCACCAGCATTCTTGCTATAACACACATTCAGCAACCAACAATACACAACCATTGTCAGGTCATCATTGAATGTCCTCATACTTATCTTCATTCTCACGTTGGGTATTGTCCTCACCAATAAATTTAGTTTTAGCTTTAGTCGTACTACTCACCAGCATTCTTGCTATAACACACATTCAGCAACCAACAATACACAACCATTGTCAGGTCATCATTGACGGTTCTGCAATAGTCATAACAAATTGTGAGAACACACCAGAAGTGCTTAAAGCAATAAACTTCTCCCCTTGGAACGGGTTAAGTTTTCCTAAATTTGAAAATTAAATGCCTGACACAACACCTGTTGCTGCCACTTCAAGTGCACCACCCACAGCCAAAGATGCTGATGCCAAAGCTCCTTCTGACTTCTCAAATCCCAATACAGCTCCTAGTCTCAGTGATTTGAAGAAAGTCAAGTATGTCTCCACCGTGACCTCCGTGGCCACACCAGCTGAAATTGAAGCCCTAGGCAAAATCTTCACCGCCATGGGCCTTGCCGCCAATGAGACTGGTCCGGCCATGTGGGATCTAGCTCGTGCATATGCTGATGTGCAGAGTTCTAAATCGGCACAGCTGATTGGAGCTACCCCTTCCAACCCTGCACTATCACGCCGAGCCCTTGCTGCTCAGTTTGATCGAATCAATATAACCCCCAGGCAATTTTGCATGTACTTTGCCAAAGTTGTTTGGAACATACTTCTCGACAGCAACATTCCACCAGCAAATTGGGCCAAACTTGGTTACCAAGAAGATACAAAATTTGCTGCATTTGACTTCTTCGATGGAGTCACCAACCCTGCCAGCCTGCAGCCTGCTGATGGTCTTATCAGGCAGCCAAATGAGAAAGAACTAGCTGCTCACTCCGTAGCTAAGTACGGCGCCTTGGCTAGGCAAAAGATCTCCACAGGTAATTATATTACCACACTTGGAGAAGTCACACGTGGACACATGGGAGGAGCTAACACCATGTACGCGATAGACGCACCCCCTAAACTTTAA

>H30_P8_b3

ATGGAAAGATCAACTCTGATTAATTTACTTCAATTGCACCACTTCGAGCCAAAACTCAGTGTTGAAGGAATCATAGTTGTGCACGGAATTGCAGGCACTGGGAAAACCACTTTACTTAGGACTTTATTTTCTGCTTACCCTAGCTTAGTTATAGGTTCACCTAGGCCTTGCTATTTAGATAAACAAAACAAAATTTCACAAGTTTGCTTATCTTGCTTTCCCAATACCCATTGTGATATTGTCGATGAGTATCATTTGCTAGAAAGTTTTCTAGAACCAAAATTGGCTATCTTTGGTGACCCCTGTCAATGCACATACATTGAGAGACTTAGAGTCCCACATTACACTTCCTTCAGAACTCATAGATTTGGAAAGTCAACTGCTGAGATTTTGAACAAACTGTTTGACCTTAATATAGTCTCAGTTAAGAAAGAAGACGACATCGTTGAATTCTTTAACCCTTTTGAAGTTGACCCCACTGAGCATATCTCTGCCTCTGAAGAAGAAGTCTTGGACTTTGTTTCTGACCAAGTGGTGACCACTAGCTCAGAGGAACTAGCAGGAATTGAGTTTGCAGAAACAACTTTCTACTGCACAACATTGGCCGCAGCTGTTGCTGAAAATCCTGCTAAGACTTTCATCTCTCTGACTAGACACACCCACAAACTCACCATTGGGGAACTAAATGCCAGGTCTAACTCCTAGATGCCAGGTCTAACTCCTAGAGCTGACCTCACTGACACATACAAAATCATTGCCATTGCTTTCTTGTTGTCAGCTTGCATTTACTTCCAAAATAGCCACTACCAACCTGTTGCTGGAGACAACTTGCACCGTTTGCCTTTTGGTGGCCAATATCAAGACGGCACCAAAAAGATATCTTATTTTCCACAACAGCAGTCATACTTTCATTCTGGAAACAAATTAAATGTCCTCATACTTATCTTCATTCTCACGTTGGGTATTGTCCTCACCAATAAATTTAGTTTTAGCTTTAGTCGTACTACTCACCAGCATTCTTGCTATAACACACATTCAGCAACCAACAATACACAACCATTGTCAGGTCATCATTGAATGTCCTCATACTTATCTTCATTCTCACGTTGGGTATTGTCCTCACCAATAAATTTAGTTTTAGCTTTAGTCGTACTACTCACCAGCATTCTTGCTATAACACACATTCAGCAACCAACAATACACAACCATTGTCAGGTCATCATTGACGGTTCTGCAATAGTCATAACAAATTGTGAGAACACACCAGAAGTGCTTAAAGCAATAAACTTCTCCCCTTGGAACGGGTTAAGTTTTCCTAAATTTGAAAATTAAATGCCTGACACAACACCTGTTGCTGCCACTTCAAGTGCACCACCCACAGCCAAAGATGCTGGTGCCAAAGCTCCTTCTGACTTCTCAAATCCCAATACAGCTCCTAGTCTCAGTGATTTGAAGAAAGTCAAGTATGTCTCCACCGTGACCTCCGTGGCCACACCAGCTGAAATTGAAGCCCTAGGCAAAATCTTCACCGCCATGGGCCTTGCCGCCAATGAGACTGGTCCGGCCATGTGGGATCTAGCTCGTGCATATGCTGATGTGCAGAGTTCTAAATCGGCACAGCTGATTGGAGCTACCCCTTCCAACCCTGCACTATCACGCCGAGCCCTTGCTGCTCAGTTTGATCGAATCAATATAACCCCCAGGCAATTTTGCATGTACTTTGCCAAAGTTGTTTGGAACATACTTCTCGACAGCAACATTCCACCAGCAAATTGGGCCAAACTTGGTTACCAAGAAGATACAAAATTTGCTGCATTTGACTTCTTCGATGGAGTCACCAACCCTGCCAGCCTGCAGCCTGCTGATGGTCTTATCAGGCAGCCAAATGAGAAAGAACTAGCTGCTCACTCCGTAGCTAAGTACGGCGCCTTGGCTAGGCAAAAGATCTCCACAGGTAATTATATTACCACACTTGGAGAAGTCACACGTGGACACATGGGAGGAGCTAACACCATGTACGCGATAGACGCACCCCCTAAACTTTAA

>H30_P8_b2

ATGGAAAGATCAACTCTGATTAATTTACTTCAATTGCACCACTTCGAGCCAAAACTCAGTGTTGAAGGAATCATAGTTGTGCACGGAATTGCAGGCACTGGGAAAACCACTTTACTTAGGACTTTATTTTCTGCTTACCCTAGCTTAGTTATAGGTTCACCTAGGCCTTGCTATTTAGATAAACAAAACAAAATTTCACAAGTTTGCTTATCTTGCTTTCCCAATACCCATTGTGATATTGTCGATGAGTATCATTTGCTAGAAAGTTTTCTAGAACCAAAATTGGCTATCTTTGGTGACCCCTGTCAATGCACATACATTGAGAGACTTAGAGTCCCACATTACACTTCCTTCAGAACTCATAGATTTGGAAAGTCAACTGCTGAGATTTTGAACAAACTGTTTGACCTTAATATAGTCTCAGTTAAGAAAGAAGACGACATCGTTGAATTCTTTAACCCTTTTGAAGTTGACCCCACTGAGCATATCTCTGCCTCTGAAGAAGAAGTCTTGGACTTTGTTTCTGACCAAGTGGTGACCACTAGCTCAGAGGAACTAGCAGGAATTGAGTTTGCAGAAACAACTTTCTACTGCACAACATTGGCCGCAGCTGTTGCTGAAAATCCTGCTAAGACTTTCATCTCTCTGACTAGACACACCCACAAACTCACCATTGGGGAACTAAATGCCAGGTCTAACTCCTAGATGCCAGGTCTAACTCCTAGAGCTGACCTCACTGACACATACAAAATCATTGCCATTGCTTTCTTGTTGTCAGCTTGCATTTACTTCCAAAATAGCCACTACCAACCTGTTGCTGGAGACAACTTGCACCGTTTGCCTTTTGGTGGCCAATATCAAGACGGCACCAAAAAGATATCTTATTTTCCACAACAGCAGTCATACTTTCATTCTGGAAACAAATTAAATGTCCTCATACTTATCTTCATTCTCACGTTGGGTATTGTCCTCACCAATAAATTTAGTTTTAGCTTTAGTCGTACTACTCACCAGCATTCTTGCTATAACACACATTCAGCAACCAACAATACACAACCATTGTCAGGTCATCATTGAATGTCCTCATACTTATCTTCATTCTCACGTTGGGTATTGTCCTCACCAATAAATTTAGTTTTAGCTTTAGTCGTACTACTCACCAGCATTCTTGCTATAACACACATTCAGCAACCAACAATACACAACCATTGTCAGGTCATCATTGACGGTTCTGCAATAGTCATAACAAATTGTGAGAACACACCAGAAGTGCTTAAAGCAATAAACTTCTCCCCTTGGAACGGGTTAAGTTTTCCTAAATTTGAAAATTAAATGCCTGACACAACACCTGTTGCTGCCACTTCAAGTGCACCACCCACAGCCAAAGATGCTGGTGCCAAAGCTCCTTCTGACTTCTCAAATCCCAATACAGCTCCTAGTCTCAGTGATTTGAAGAAAGTCAAGTATGTCTCCACCGTGACCTCCGTGGCCACACCAGCTGAAATTGAAGCCCTAGGCAAAATCTTCACCGCCATGGGCCTTGCCGCCAATGAGACTGGTCCGGCCATGTGGGATCTAGCTCGTGCATATGCTGATGTGCAGAGTTCTAAATCGGCACAGCTGATTGGAGCTACCCCTTCCAACCCTGCACTATCACGCCGAGCCCTTGCTGCTCAGTTTGATCGAATCAATATAACCCCCAGGCAATTTTGCATGTACTTTGCCAAAGTTGTTTGGAACATACTTCTCGACAGCAACATTCCACCAGCAAATTGGGCCAAACTTGGTTACCAAGAAGATACAAAATTTGCTGCATTTGACTTCTTCGATGGAGTCACCAACCCTGCCAGCCTGCAGCCTGCTGATGGTCTTATCAGGCAGCCAAATGAGAAAGAACTAGCTGCTCACTCCGTAGCTAAGTACGGCGCCTTGGCTAGGCAAAAGATCTCCACAGGTAATTATATTACCACACTTGGAGAAGTCACACGTGGACACATGGGAGGAGCTAACACCATGTACGCGATAGACGCACCCCCTAAACTTTAA

>H30_P8_b1

ATGGAAAGATCAACTCTGATTAATTTACTTCAATTGCACCACTTCGAGCCAAAACTCAGTGTTGAAGGAATCATAGTTGTGCACGGAATTGCAGGCACTGGGAAAACCACTTTACTTAGGACTTTATTTTCTGCTTACCCTAGCTTAGTTATAGGTTCACCTAGGCCTTGCTATTTAGATAAACAAAACAAAATTTCACAAGTTTGCTTATCTTGCTTTCCCAATACCCATTGTGATATTGTCGATGAGTATCATTTGCTAGAAAGTTTTCTAGAACCAAAATTGGCTATCTTTGGTGACCCCTGTCAATGCACATACATTGAGAGACTTAGAGTCCCACATTACACTTCCTTCAGAACTCATAGATTTGGAAAGTCAACTGCTGAGATTTTGAACAAACTGTTTGACCTTAATATAGTCTCAGTTAAGAAAGAAGACGACATCGTTGAATTCTTTAACCCTTTTGAAGTTGACCCCACTGAGCATATCTCTGCCTCTGAAGAAGAAGTCTTGGACTTTGTTTCTGACCAAGTGGTGACCACTAGCTCAGAGGAACTAGCAGGAATTGAGTTTGCAGAAACAACTTTCTACTGCACAACATTGGCCGCAGCTGTTGCTGAAAATCCTGCTAAGACTTTCATCTCTCTGACTAGACACACCCACAAACTCACCATTGGGGAACTAAATGCCAGGTCTAACTCCTAGATGCCAGGTCTAACTCCTAGAGCTGACCTCACTGACACATACAAAATCATTGCCATTGCTTTCTTGTTGTCAGCTTGCATTTACTTCCAAAATAGCCACTACCAACCTGTTGCTGGAGACAACTTGCACCGTTTGCCTTTTGGTGGCCAATATCAAGACGGCACCAAAAAGATATCTTATTTTCCACAACAGCAGTCATACTTTCATTCTGGAAACAAATTAAATGTCCTCATACTTATCTTCATTCTCACGTTGGGTATTGTCCTCACCAATAAATTTAGTTTTAGCTTTAGTCGTACTACTCACCAGCATTCTTGCTATAACACACATTCAGCAACCAACAATACACAACCATTGTCAGGTCATCATTGAATGTCCTCATACTTATCTTCATTCTCACGTTGGGTATTGTCCTCACCAATAAATTTAGTTTTAGCTTTAGTCGTACTACTCACCAGCATTCTTGCTATAACACACATTCAGCAACCAACAATACACAACCATTGTCAGGTCATCATTGACGGTTCTGCAATAGTCATAACAAATTGTGAGAACACACCAGAAGTGCTTAAAGCAATAAACTTCTCCCCTTGGAACGGGTTAAGTTTTCCTAAATTTGAAAATTAAATGCCTGACACAACACCTGTTGCTGCCACTTCAAGTGCACCACCCACAGCCAAAGATGCTGGTGCCAAAGCTCCTTCTGACTTCTCAAATCCCAATACAGCTCCTAGTCTCAGTGATTTGAAGAAAGTCAAGTATGTCTCCACCGTGACCTCCGTGGCCACACCAGCTGAAATTGAAGCCCTAGGCAAAATCTTCACCGCCATGGGCCTTGCCGCCAATGAGACTGGTCCGGCCATGTGGGATCTAGCTCGTGCATATGCTGATGTGCAGAGTTCTAAATCGGCACAGCTGATTGGAGCTACCCCTTCCAACCCTGCACTATCACGCCGAGCCCTTGCTGCTCAGTTTGATCGAATCAATATAACCCCCAGGCAATTTTGCATGTACTTTGCCAAAGTTGTTTGGAACATACTTCTCGACAGCAACATTCCACCAGCAAATTGGGCCAAACTTGGTTACCAAGAAGATACAAAATTTGCTGCATTTGACTTCTTCGATGGAGTCACCAACCCTGCCAGCCTGCAGCCTGCTGATGGTCTTATCAGGCAGCCAAATGAGAAAGAACTAGCTGCTCACTCCGTAGCTAAGTACGGCGCCTTGGCTAGGCAAAAGATCTCCACAGGTAATTATATTACCACACTTGGAGAAGTCACACGTGGACACATGGGAGGAGCTAACACCATGTACGCGATAGACGCACCCCCTAAACTTTAA

>H30_P8_a14

ATGGAAAGATCAACTCTGATTAATTTACTTCAATTGCACCACTTCGAGCCAAAACTCAGTGTTGAAGGAATCATAGTTGTGCACGGAATTGCAGGCACTGGGAAAACCACTTTACTTAGGACTTTATTTTCTGCTTACCCTAGCTTAGTTATAGGTTCACCTAGGCCTTGCTATTTAGATAAACAAAACAAAATTTCACAAGTTTGCTTATCTTGCTTTCCCAATACCCATTGTGATATTGTCGATGAGTATCATTTGCTAGAAAGTTTTCTAGAACCAAAATTGGCTATCTTTGGTGACCCCTGTCAATGCACATACATTGAGAGACTTAGAGTCCCACATTACACTTCCTTCAGAACTCATAGATTTGGAAAGTCAACTGCTGAGATTTTGAACAAACTGTTTGACCTTAATATAGTCTCAGTTAAGAAAGAAGACGACATCGTTGAATTCTTTAACCCTTTTGAAGTTGACCCCACTGAGCATATCTCTGCCTCTGAAGAAGAAGTCTTGGACTTTGTTTCTGACCAAGTGGTGACCACTAGCTCAGAGGAACTAGCAGGAATTGAGTTTGCAGAAACAACTTTCTACTGCACAACATTGGCCGCAGCTGTCGCTGAAAATCCTGCTAAGACTTTCATCTCTCTGACTAGACACACCCACAAACTCACCATTGGGGAACTAAATGCCAGGTCTAACTCCTAGATGCCAGGTCTAACTCCTAGAGCTGACCTCACTGACACATACAAAATCATTGCCATTGCTTTCTTGTTGTCAGCTTGCATTTACTTCCAAAATAGCCACTACCAACCTGTTGCTGGAGACAACTTGCACCGTTTGCCTTTTGGTGGCCAATATCAAGACGGCACCAAAAAGATATCTTATTTTCCACAACAGCAGTCATACTTTCATTCTGGAAACAAATTAAATGTCCTCATACTTATCTTCATTCTCACGTTGGGTATTGTCCTCACCAGTAAATTTAGTTTTAGCTTTAGTCGTACTACTCACCAGCATTCTTGCTACAACACACATTCAGCAACCAACAATACACAACCATTGTCAGGTCATCATTGAATGTCCTCATACTTATCTTCATTCTCACGTTGGGTATTGTCCTCACCAGTAAATTTAGTTTTAGCTTTAGTCGTACTACTCACCAGCATTCTTGCTACAACACACATTCAGCAACCAACAATACACAACCATTGTCAGGTCATCATTGACGGTTCTGCAATAGTCATAACAAATTGTGAGAACACACCAGAAGTGCTTAAAGCAATAAACTTCTCCCCTTGGAACGGGTTAAGTTTTCCTAAATTTGAAAATTAAATGCCTGACACAACACCTGTTGCTGCCACTTCAAGTGCACCACCCACAGCCAAAGATGCTGGTGCCAAAGCTCCTTCTGACTTCTCAAATCCCAATACAGCTCCTAGTCTCAGTGATTTGAAGAAAGTCAAGTATGTCTCCACCGTGACCTCCGTGGCCACACCAGCTGAAATTGAAGCCCTAGGCAAAATCTTCACCGCCATGGGCCTTGCCGCCAATGAGACTGGTCCGGCCATGTGGGATCTAGCTCGTGCATATGCTGATGTACAGAGTTCTAAATCGGCACAGCTGATTGGAGCTACCCCTTCCAACCCTGCACTATCACGCCGAGCCCTTGCTGCTCAGTTTGATCGAATCAATATAACCCCCAGGCAATTTTGCATGTACTTTGCCAAAGTTGTTTGGAACATACTTCTCGACAGCAACATTCCACCAGCAAATTGGGCCAAACTTGGTTACCAAGAAGATACAAAATTTGCTGCATTTGACTTCTTCGATGGAGTCACCAACCCTGCCAGCCTGCAGCCTGCTGATGGTCTTATTAGGCAGCCAAATGAGAAAGAACTAGCTGCTCACTCCGTAGCTAAGTACGGCGCCTTGGCTAGGCAAAAGATCTCCACAGGTAATTATATTACCACACTTGGAGAAGTCACACGTGGACACATGGGAGGAGCTAACACCATGTACGCGATAGACGCACCCCCTAAACTTTAA

>H30_P8_a13

ATGGAAAGATCAACTCTGATTAATTTACTTCAATTGCACCACTTCGAGCCAAAACTCAGTGTTGAAGGAATCATAGTTGTGCACGGAATTGCAGGCACTGGGAAAACCACTTTACTTAGGACTTTATTTTCTGCTTACCCTAGCTTAGTTATAGGTTCACCTAGGCCTTGCTATTTAGATAAACAAAACAAAATTTCACAAGTTTGCTTATCTTGCTTTCCCAATACCCATTGTGATATTGTCGATGAGTATCATTTGCTAGAAAGTTTTCTAGAACCAAAATTGGCTATCTTTGGTGACCCCTGTCAATGCACATACATTGAGAGACTTAGAGTCCCACATTACACTTCCTTCAGAACTCATAGATTTGGAAAGTCAACTGCTGAGATTTTGAACAAACTGTTTGACCTTAATATAGTCTCAGTTAAGAAAGAAGACGACATCGTTGAATTCTTTAACCCTTTTGAAGTTGACCCCACTGAGCATATCTCTGCCTCTGAAGAAGAAGTCTTGGACTTTGTTTCTGACCAAGTGGTGACCACTAGCTCAGAGGAACTAGCAGGAATTGAGTTTGCAGAAACAACTTTCTACTGCACAACATTGGCCGCAGCTGTTGCTGAAAATCCTGCTAAGACTTTCATCTCTCTGACTAGACACACCCACAAACTCACCATTGGGGAACTAAATGCCAGGTCTAACTCCTAGATGCCAGGTCTAACTCCTAGAGCTGACCTCACTGACACATACAAAATCATTGCCATTGCTTTCTTGTTGTCAGCTTGCATTTACTTCCAAAATAGCCACTACCAACCTGTTGCTGGAGACAACTTGCACCGTTTGCCTTTTGGTGGCCAATATCAAGACGGCACCAAAAAGATATCTTATTTTCCACAACAGCAGTCATACTTTCATTCTGGAAACAAATTAAATGTCCTCATACTTATCTTCATTCTCACGTTGGGTATTGTCCTCACCAATAAATTTAGTTTTAGCTTTAGTCGTACTACTCACCAGCATTCTTGCTACAACACACATTCAGCAACCAACAATACACAACCATTGTCAGGTCATCATTGAATGTCCTCATACTTATCTTCATTCTCACGTTGGGTATTGTCCTCACCAATAAATTTAGTTTTAGCTTTAGTCGTACTACTCACCAGCATTCTTGCTACAACACACATTCAGCAACCAACAATACACAACCATTGTCAGGTCATCATTGACGGTTCTGCAATAGTCATAACAAATTGTGAGAACACACCAGAAGTGCTTAAAGCAATAAACTTCTCCCCTTGGAACGGGTTAAGTTTTCCTAAATTTGAAAATTAAATGCCTGACACAACACCTGTTGCTGCCACTTCAAGTGCACCACCCACAGCCAAAGATGCTGGTGCCAAAGCTCCTTCTGACTTCTCAAATCCCAATACAGCTCCTAGTCTCAGTGATTTGAAGAAAGTCAAGTACGTCTCCACCGTGACCTCCGTGGCCACACCAGCTGAAATTGAAGCCCTAGGCAAAATCTTCACCGCCATGGGCCTTGCCGCCAATGAGACTGGTCCGGCCATGTGGGATCTAGCTCGTGCATATGCTGATGTGCAGAGTTCTAAATCGGCACAGCTGATTGGAGCTACCCCTTCCAACCCTGCACTATCACGCCGAGCCCTTGCTGCTCAGTTTGATCGAATCAATATAACCCCCAGGCAATTTTGCATGTACTTTGCCAAAGTTGTTTGGAACATACTTCTCGACAGCAACATTCCACCAGCAAATTGGGCCAAACTTGGTTACCAAGAAGATACAAAATTTGCTGCATTTGACTTCTTCGATGGAGTCACCAACCCTGCCAGCCTGCAGCCTGCTGATGGTCTTATTAGGCAGCCAAATGAGAAAGAACTAGCTGCTCACTCCGTAGCTAAGTACGGCGCCTTGGCTAGGCAAAAGATCTCCACAGGTAATTATATTACCACACTTGGAGAAGTCACACGTGGACACATGGGAGGAGCTAACACCATGTACGCGATAGACGCACCCCCTAAACTTTAA

>H30_P8_a12

ATGGAAAGATCAACTCTGATTAATTTACTTCAATTGCACCACTTCGAGCCAAAACTCAGTGTTGAAGGAATCATAGTTGTGCACGGAATTGCAGGCACTGGGAAAACCACTTTACTTAGGACTTTATTTTCTGCTTACCCTAGCTTAGTTATAGGTTCACCTAGGCCTTGCTATTTAGATAAACAAAACAAAATTTCACAAGTTTGCTTATCTTGCTTTCCCAATACCCATTGTGATATTGTCGATGAGTATCATTTGCTAGAAAGTTTTCTAGAACCAAAATTGGCTATCTTTGGTGACCCCTGTCAATGCACATACATTGAGAGACTTAGAGTCCCACATTACACTTCCTTCAGAACTCATAGATTTGGAAAGTCAACTGCTGAGATTTTGAACAAACTGTTTGACCTTAATATAGTCTCAGTTAAGAAAGAAGACGACATCGTTGAATTCTTTAACCCTTTTGAAGTTGACCCCACTGAGCATATCTCTGCCTCTGAAGAAGAAGTCTTGGACTTTGTTTCTGACCAAGTGGTGACCACTAGCTCAGAGGAACTAGCAGGAATTGAGTTTGCAGAAACAACTTTCTACTGCACAACATTGGCCGCAGCTGTTGCTGAAAATCCTGCTAAGACTTTCATCTCTCTGACTAGACACACCCACAAACTCACCATTGGGGAACTAAATGCCAGGTCTAACTCCTAGATGCCAGGTCTAACTCCTAGAGCTGACCTCACTGACACATACAAAATCATTGCCATTGCTTTCTTGTTGTCAGCTTGCATTTACTTCCAAAATAGCCACTACCAACCTGTTGCTGGAGACAACCTGCACCGTTTGCCTTTTGGTGGCCAATATCAAGACGGCACCAAAAAGATATCTTATTTTCCACAACAGCAGTCATACTTTCATTCTGGAAACAAATTAAATGTCCTCATACTTATCTTCATTCTCACGTTGGGTATTGTCCTCACCAATAAATTTAGTTTTAGCTTTAGTCGTACTACTCACCAGCATTCTTGCTACAACACACATTCAGCAACCAACAATACACAACCATTGTCAGGTCATCATTGAATGTCCTCATACTTATCTTCATTCTCACGTTGGGTATTGTCCTCACCAATAAATTTAGTTTTAGCTTTAGTCGTACTACTCACCAGCATTCTTGCTACAACACACATTCAGCAACCAACAATACACAACCATTGTCAGGTCATCATTGACGGTTCTGCAATAGTCATAACAAATTGTGAGAACACACCAGAAGTGCTTAAAGCAATAAACTTCTCCCCTTGGAACGGGTTAAGTTTTCCTAAATTTGAAAATTAAATGCCTGACACAACACCTGTTGCTGCCACTTCAAGTGCACCACCCACAGCCAAAGATGCTGGTGCCAAAGCTCCTTCTGACTTCTCAAATCCCAATACAGCTCCTAGTCTCAGTGATTTGAAGAAAGTCAAGTATGTCTCCACCGTGACCTCCGTGGCCACACCAGCTGAAATTGAAGCCCTAGGCAAAATCTTCACCGCCATGGGCCTTGCCGCCAATGAGACTGGTCCGGCCATGTGGGATCTAGCTCGTGCATATGCTGATGTGCAGAGTTCTAAATCGGCACAGCTGATTGGAGCTACCCCTTCCAACCCTGCACTATCACGCCGAGCCCTTGCTGCTCAGTTTGATCGAATCAATATAACCCCCAGGCAATTTTGCATGTACTTTGCCAAAGTTGTTTGGAACATACTTCTCGACAGCAACATTCCACCAGCAAATTGGGCCAAACTTGGTTACCAAGAAGATACAAAATTTGCTGCATTTGACTTCTTCGATGGAGTCACCAACCCTGCCAGCCTGCAGCCTGCTGATGGTCTTATCAGGCAGCCAAATGAGAAAGAACTAGCTGCTCACTCCGTAGCTAAGTACGGCGCCTTGGCTAGGCAAAAGATCTCCACAGGTAATTATATTACCACACTTGGAGAAGTCACACGTGGACACATGGGAGGAGCTAACACCATGTACGCGATAGACGCACCCCCTAAACTTTAA

>H30_P8_a11

ATGGAAAGATCAACTCTGATTAATTTACTTCAATTGCACCACTTCGAGCCAAAACTCAGTGTTGAAGGAATCATAGTTGTGCACGGAATTGCAGGCACTGGGAAAACCACTTTACTTAGGACTTTATTTTCTGCTTACCCTAGCTTAGTTATAGGTTCACCTAGGCCTTGCTATTTAGATAAACAAAACAAAATTTCACAAGTTTGCTTATCTTGCTTTCCCAATACCCATTGTGATATTGTCGATGAGTATCATTTGCTAGAAAGTTTTCTAGAACCAAAATTGGCTATCTTTGGTGACCCCTGTCAATGCACATACATTGAGAGACTTAGAGTCCCACATTACACTTCCTTCAGAACTCATAGATTTGGAAAGTCAACTGCTGAGATTTTGAACAAACTGTTTGACCTTAATATAGTCTCAGTTAAGAAAGAAGACGACATCGTTGAATTCTTTAACCCTTTTGAAGTTGACCCCACTGAGCATATCTCTGCCTCTGAAGAAGAAGTCTTGGACTTTGTTTCTGACCAAGTGGTGACCACTAGCTCAGAGGAACTAGCAGGAATTGAGTTTGCAGAAACAACTTTCTACTGCACAACATTGGCCGCAGCTGTTGCTGAAAATCCTGCTAAGACTTTCATCTCTCTGACTAGACACACCCACAAACTCACCATTGGGGAACTAAATGCCAGGTCTAACTCCTAGATGCCAGGTCTAACTCCTAGAGCTGACCTCACTGACACATACAAAATCATTGCCATTGCTTTCTTGTTGTCAGCTTGCATTTACTTCCAAAATAGCCACTACCAACCTGTTGCTGGAGACAACTTGCACCGTTTGCCTTTTGGTGGCCAATATCAAGACGGCACCAAAAAGATATCTTATTTTCCACAACAGCAGTCATACTTTCATTCTGGAAACAAATTAAATGTCCTCATACTTATCTTCATCCTCACGTTGGGTATTGTCCTCACCAATAAATTTAGTTTTAGCTTTAGTCGTACTACTCACCAGCATTCTTGCTATAACACACATTCAGCAACCAACAATACACAACCATTGTCAGGTCATCATTGAATGTCCTCATACTTATCTTCATCCTCACGTTGGGTATTGTCCTCACCAATAAATTTAGTTTTAGCTTTAGTCGTACTACTCACCAGCATTCTTGCTATAACACACATTCAGCAACCAACAATACACAACCATTGTCAGGTCATCATTGACGGTTCTGCAATAGTCATAACAAATTGTGAGAACACACCAGAAGTGCTTAAAGCAATAAACTTCTCCCCTTGGAACGGGTTAAGTTTTCCTAAATTTGAAAATTAAATGCCTGACACAACACCTGTTGCTGCCACTTCAAGTGCACCACCCACAGCCAAAGATGCTGGTGCCAAAGCTCCTTCTGACTTCTCAAATCCCAATACAGCTCCTAGTCTCAGTGATTTGAAGAAAGTCAAGTATGTCTCCACCGTGACCTCCGTGGCCACACCAGCTGAAATTGAAGCCCTAGGCAAAATCTTCACCGCCATGGGCCTTGCCGCCAATGAGACTGGTCCGGCCATGTGGGATCTAGCTCGTGCATATGCTGATGTGCAGAGTTCTAAATCGGCACAGCTGATTGGAGCTACCCCTTCCAACCCTGCACTATCACGCCGAGCCCTTGCTGCTCAGTTTGATCGAATCAATATAACCCCCAGGCAATTTTGCATGTACTTTGCCAAAGTTGTTTGGAACATACTTCTCGACAGCAACATTCCACCAGCAAATTGGGCCAAACTTGGTTACCAAGAAGATACAAAATTTGCTGCATTTGACTTCTTCGATGGAGTCACCAACCCTGCCAGCCTGCAGCCTGCTGATGGTCTTATCAGGCAGCCAAATGAGAAAGAACTAGCTGCTCACTCCGTAGCTAAGTACGGCGCCTTGGCTAGGCAAAAGATCTCCACAGGTAATTATATTACCACACTTGGAGAAGTCACACGTGGACACATGGGAGGAGCTAACACCATGTACGCGATAGACGCACCCCCTAAACTTTAA

>H30_P8_a10

ATGGAAAGATCAACTCTGATTAATTTACTTCAATTGCACCACTTCGAGCCAAAACTCAGTGTTGAAGGAATCATAGTTGTGCACGGAATTGCAGGCACTGGGAAAACCACTTTACTTAGGACTTTATTTTCTGCTTACCCTAGCTTAGTTATAGGTTCACCTAGGCCTTGCTATTTAGATAAACAAAACAAAATTTCACAAGTTTGCTTATCTTGCTTTCCCAATACCCATTGTGATATTGTCGATGAGTATCATTTGCTAGAAAGTTTTCTAGAACCAAAATTGGCTATCTTTGGTGACCCCTGTCAATGCACATACATTGAGAGACTTAGAGTCCCACATTACACTTCCTTCAGAACTCATAGATTTGGAAAGTCAACTGCTGAGATTTTGAACAAACTGTTTGACCTTAATATAGTCTCAGTTAAGAAAGAAGACGACATCGTTGAATTCTTTAACCCTTTTGAAGTTGACCCCACTGAGCATATCTCTGCCTCTGAAGAAGAAGTCTTGGACTTTGTTTCTGACCAAGTGGTGACCACTAGCTCAGAGGAACTAGCAGGAATTGAGTTTGCAGAAACAACTTTCTACTGCACAACATTGGCCGCAGCTGTTGCTGAAAATCCTGCTAAGACTTTCATCTCTCTGACTAGACACACCCACAAACTCACCATTGGGGAACTAAATGCCAGGTCTAACTCCTAGATGCCAGGTCTAACTCCTAGAGCTGACCTCACTGACACATACAAAATCATTGCCATTGCTTTCTTGTTGTCAGCTTGCATTTACTTCCAAAATAGCCACTACCAACCTGTTGCTGGAGACAACTTGCACCGTTTGCCTTTTGGTGGCCAATATCAAGACGGCACCAAAAAGATATCTTATTTTCCACAACAGCAGTCATACTTTCATTCTGGAAACAAATTAAATGTCCTCATACTTATCTTCATTCTCACGTTGGGTATTGTCCTCACCAATAAATTTAGTTTTAGCTTTAGTCGTACTACTCACCAGCATTCTTGCTACAACACACATTCAGCAACCAACAATACACAACCATTGTCAGGTCATCATTGAATGTCCTCATACTTATCTTCATTCTCACGTTGGGTATTGTCCTCACCAATAAATTTAGTTTTAGCTTTAGTCGTACTACTCACCAGCATTCTTGCTACAACACACATTCAGCAACCAACAATACACAACCATTGTCAGGTCATCATTGACGGTTCTGCAATAGTCATAACAAATTGTGAGAACACACCAGAAGTGCTTAAAGCAATAAACTTCTCCCCTTGGAACGGGTTAAGTTTTCCTAAATTTGAAAATTAAGTGCCTGACACAACACCTGTTGCTGCCACTTCAAGTGCACCACCCACAGCCAAAGATGCTGGTGCCAAAGCTCCTTCTGACTTCTCAAATCCCAATACAGCTCCTAGTCTCAGTGATTTGAAGAAAGTCAAGTATGTCTCCACCGTGACCTCCGTGGCCACACCAGCTGAAATTGAAGCCCTAGGCAAAATCTTCACCGCCATGGGCCTTGCCGCCAATGAGACTGGTCCGGCCATGTGGGATCTAGCTCGTGCACATGCTGATGTGCAGAGTTCTAAATCGGCACAGCTGATTGGAGCTACCCCTTCCAACCCTGCACTATCACGCCGAGCCCTTGCTGCTCAGTTTGATCGAATCAATATAACCCCCAGGCAATTTTGCATGTACTTTGCCAAAGTTGTTTGGAACATACTTCTCGACAGCAACATTCCACCAGCAAATTGGGCCAAACTTGGTTACCAAGAAGATACAAAATTTGCTGCATTTGACCTCTTCGATGGAGTCACCAACCCTGCCAGCCTGCAGCCTGCTGATGGTCTTATTAGGCAGCCAAATGAGAAAGAACTAGCTGCTCACTCCGTAGCTAAGTACGGCGCCTTGGCTAGGCAAAAGATCTCCACAGGTAATTATATTACCACACTTGGAGAAGTCACACGTGGACACATGGGAGGAGCTAACACCATGTACGCGATAGACGCACCCCCTAAACTTTAA

>H30_P8_a9

ATGGAAAGATCAACTCTGATTAATTTACTTCAATTGCACCACTTCGAGCCAAAACTCAGTGTTGAAGGAATCATAGTTGTGCACGGAATTGCAGGCACTGGGAAAACCACTTTACTTAGGACTTTATTTTCTGCTTACCCTAGCTTAGTTATAGGTTCACCTAGGCCTTGCTATTTAGATAAACAAAACAAAATTTCACAAGTTTGCTTATCTTGCTTTCCCAATACCCATTGTGATATTGTCGATGAGTATCATTTGCTAGAAAGTTTTCTAGAACCAAAATTGGCTATCTTTGGTGACCCCTGTCAATGCACATACATTGAGAGACTTAGAGTCCCACATTACACTTCCTTCAGAACTCATAGATTTGGAAAGTCAACTGCTGAGATTTTGAACAAACTGTTTGACCTTAATATAGTCTCAGTTAAGAAAGAAGACGACATCGTTGAATTCTTTAACCCTTTTGAAGTTGACCCCACTGAGCATATCTCTGCCTCTGAAGAAGAAGTCTTGGACTTTGTTTCTGACCAAGTGGTGACCACTAGCTCAGAGGAACTAGCAGGAATTGAGTTTGCAGAAACAACTTTCTACTGCACAACATTGGCCGCAGCTGTTGCTGAAAATCCTGCTAAGACTTTCATCTCTCTGACTAGACACACCCACAAACTCACCATTGGGGAACTAAATGCCAGGTCTAACTCCTAGATGCCAGGTCTAACTCCTAGAGCTGACCTCACTGACACATACAAAATCATTGCCATTGCTTTCTTGTTGTCAGCTTGCATTTACTTCCAAAATAGCCACTACCAACCTGTTGCTGGAGACAACTTGCACCGTTTGCCTTTTGGTGGCCAATATCAAGACGGCACCAAAAAGATATCTTATTTTCCACAACAGCAGTCATACTTTCATTCTGGAAACAAATTAAATGTCCTCATACTTATCTTCATTCTCACGTTGGGTATTGTCCTCACCAATAAATTTAGTTTTAGCTTTAGTCGTACTACTCACCAGCATTCTTGCTACAACACACATTCAGCAACCAACAATACACAACCATTGTCAGGTCATCATTGAATGTCCTCATACTTATCTTCATTCTCACGTTGGGTATTGTCCTCACCAATAAATTTAGTTTTAGCTTTAGTCGTACTACTCACCAGCATTCTTGCTACAACACACATTCAGCAACCAACAATACACAACCATTGTCAGGTCATCATTGACGGTTCTGCAATAGTCATAACAAATTGTGAGAACACACCAGAAGTGCTTAAAGCAATAAACTTCTCCCCTTGGAACGGGTTAAGTTTTCCTAAATTTGAAAATTAAATGCCTGACACAACACCTGTTGCTGCCACTTCAAGTGCACCACCCACAGCCAAAGATGCTGGTGCCAAAGCTCCTTCTGACTTCTCAAATCCCAATACAGCTCCTAGTCTCAGTGATTTGAAGAAAGTCAAGTATGTCTCCACCGTGACCTCCGTGGCCACACCAGCTGAAATTGAAGCCCTAGGCAAAATCTTCACCGCCATGGGCCTTGCCGCCAATGAGACTGGTCCGGCCATGTGGGATCTAGCTCGTGCATATGCTGGTGTGCAGAGTTCTAAATCGGCACAGCTGATTGGAGCTACCCCTTCCAACCCTGCACTATCACGCCGAGCCCTTGCTGCTCAGTTTGATCGAATCAATATAACCCCCAGGCAATTTTGCATGTACTTTGCCAAAGTTGTTTGGAACATACTTCTCGACAGCAACATTCCACCAGCAAATTGGGCCAAACTTGGTTACCAAGAAGATACAAAATTTGCTGCATTTGACTTCTTCGATGGAGTCACCAACCCTGCCAGCCTGCAGCCTGCTGATGGTCTTATCAGGCAGCCAAATGAGAAAGAACTAGCTGCTCACTCCGTAGCTAAGTACGGCGCCTTGGCTAGGCAAAAGATCTCCACAGGTAATTATATTACCACACTTGGAGAAGTCACACGTGGACACATGGGAGGAGCTAACACCATGTACGCGATAGACGCACCCCCTAAACTTTAA

>H30_P8_a8

ATGGAAAGATCAACTCTGATTAATTTACTTCAATTGCACCACTTCGAGCCAAAACTCAGTGTTGAAGGAATCATAGTTGTGCACGGAATTGCAGGCACTGGGAAAACCACTTTACTTAGGACTTTATTTTCTGCTTACCCTAGCTTAGTTATAGGTTCACCTAGGCCTTGCTATTTAGATAAACAAAACAAAATTTCACAAGTTTGCTTATCTTGCTTTCCCAATACCCATTGTGATATTGTCGATGAGTATCATTTGCTAGAAAGTTTTCTAGAACCAAAATTGGCTATCTTTGGTGACCCCTGTCAATGCACATACATTGAGAGACTTAGAGTCCCACATTACACTTCCTTCAGAACTCATAGATTTGGAAAGTCAACTGCTGAGATTTTGAACAAACTGTTTGACCTTAATATAGTCTCAGTTAAGAAAGAAGACGACATCGTTGAATTCTTTAACCCTTTTGAAGTTGACCCCACTGAGCATATCTCTGCCTCTGAAGAAGAAGTCTTGGACTTTGTTTCTGACCAAGTGGTGACCACTAGCTCAGAGGAACTAGCAGGGATTGAGTTTGCAGAAACAACTTTCTACTGCACAACATTGGCCGCAGCTGTTGCTGAAAATCCTGCTAAGACTTTCATCTCTCTGACTAGACACACCCACAAACTCACCATTGGGGAACTAAATGCCAGGTCTAACTCCTAGATGCCAGGTCTAACTCCTAGAGCTGACCTCACTGACACATACAAAATCATTGCCATTGCTTTCTTGTTGTCAGCTTGCATTTACTTCCAAAATAGCCACTACCAACCTGTTGCTGGAGACAACTTGCACCGTTTGCCTTTTGGTGGCCAATATCAAGACGGCACCAAAAAGATATCTTATTTTCCACAACAGCAGTCATACTTTCATTCTGGAAACAAATTAAATGTCCTCATACTTATCTTCATTCTCACGTTGGGTATTGTCCTCACCAATAAATTTAGTTTTAGCTTTAGTCGTACTACTCACCAGCATTCTTGCTATAACACACATTCAGCAACCAACAATACACAACCATTGTCAGGTCATCATTGAATGTCCTCATACTTATCTTCATTCTCACGTTGGGTATTGTCCTCACCAATAAATTTAGTTTTAGCTTTAGTCGTACTACTCACCAGCATTCTTGCTATAACACACATTCAGCAACCAACAATACACAACCATTGTCAGGTCATCATTGACGGTTCTGCAATAGTCATAACAAATTGTGAGAACACACCAGAAGTGCTTAAAGCAATAAACTTCTCCCCTTGGAACGGGTTAAGTTTTCCTAAATTTGAAAATTAAATGCCTGACACAACACCTGTTGCTGCCACTTCAAGTGCACCACCCACAGCCAAAGATGCTGGTGCCAAAGCTCCTTCTGACTTCTCAAATCCCAATACAGCTCCTAGTCTCAGTGATTTGAAGAAAGTCAAGTATGTCTCCACCGTGACCTCCGTGGCCACACCAGCTGAAATTGAAGCCCTAGGCAAAATCTTCACCGCCATGGGCCTTGCCGCCAATGAGACTGGTCCGGCCATGTGGGATCTAGCTCGTGCATATGCTGATGTGCAGAGTTCTAAATCGGCACAGCTGATTGGAGCTACCCCTTCCAACCCTGCACTATCACGCCGAGCCCTTGCTGCTCAGTTTGATCGAATCAATATAACCCCCAGGCAATTTTGCATGTACTTTGCCAAAGTTGTTTGGAACATACTTCTCGACAGCAACATTCCACCAGCAAATTGGGCCAAACTTGGTTACCAAGAAGATACAAAATTTGCTGCATTTGACTTCTTCGATGGAGTCACCAACCCTGCCAGCCTGCAGCCTGCTGATGGTCTTATTAGGCAGCCAAATGAGAAAGAACTAGCTGCTCACTCCGTAGCTAAGTACGGCGCCTTGGCTAGGCAAAAGATCTCCACAGGTAATTATATTACCACACTTGGAGAAGTCACACGTGGACACATGGGAGGAGCTAACACCATGTACGCGATAGACGCACCCCCTAAACTTTAA

>H30_P8_a7

ATGGAAAGATCAACTCTGATTAATTTACTTCAATTGCACCACTTCGAGCCAAAACTCAGTGTTGAAGGAATCATAGTTGTGCACGGAATTGCAGGCACTGGGAAAACCACTTTACTTAGGACTTTATTTTCTGCTTACCCTAGCTTAGTTATAGGTTCACCTAGGCCTTGCTATTTAGATAAACAAAACAAAATTTCACAAGTTTGCTTATCTTGCTTTCCCAATACCCATTGTGATATTGTCGATGAGTATCATTTGCTAGAAAGTTTTCTAGAACCAAAATTGGCTATCTTTGGTGACCCCTGTCAATGCACATACATTGAGAGACTTAGAGTCCCACATTACACTTCCTTCAGAACTCATAGATTTGGAAAGTCAACTGCTGAGATTTTGAACAAACTGTTTGACCTTAATATAGTCTCAGTTAAGAAAGAAGACGACATCGTTGAATTCTTTAACCCTTTTGAAGTTGACCCCACTGAGCATATCTCTGCCTCTGAAGAAGAAGTCTTGGACTTTGTTTCTGACCAAGTGGTGACCACTAGCTCAGAGGAACTAGCAGGAATTGAGTTTGCAGAAACAACTTTCTACTGCACAACATTGGCCGCAGCTGTTGCTGAAAATCCTGCTAAGACTTTCATCTCTCTGACTAGACACACCCACAAACTCACCATTGGGGAACTAAATGCCAGGTCTAACTCCTAGATGCCAGGTCTAACTCCTAGAGCTGACCTCACTGACACATACAAAATCATTGCCATTGCTTTCTTGTTGTCAGCTTGCATTTACTTCCAAAATAGCCACTACCAACCTGTTGCTGGAGACAACTTGCACCGTTTGCCTTTTGGTGGCCAATATCAAGACGGCACCAAAAAGATATCTTATTTTCCACAACAGCAGTCATACTTTCATTCTGGAAACAAATTAAATGTCCTCATACTTATCTTCATTCTCACGTTGGGTATTGTCCTCACCAATAAATTTAGTTTTAGCTTTAGTCGTACTACTCACCAGCATTCTTGCTACAACACACATTCAGCAACCAACAATACACAACCATTGTCAGGTCATCATTGAATGTCCTCATACTTATCTTCATTCTCACGTTGGGTATTGTCCTCACCAATAAATTTAGTTTTAGCTTTAGTCGTACTACTCACCAGCATTCTTGCTACAACACACATTCAGCAACCAACAATACACAACCATTGTCAGGTCATCATTGACGGTTCTGCAATAGTCATAACAAATTGTGAGAACACACCAGAAGTGCTTAAAGCAATAAACTTCTCCCCTTGGAACGGGTTAAGTTTTCCTAAATTTGAAAATTAAATGCCTGACACAACACCTGTTGCTGCCACTTCAAGTGCACCACCCACAGCCAAAGATGCTGGTGCCAAAGCTCCTTCTGACTTCTCAAATCCCAATACAGCTCCTAGTCTCAGTGATTTGAAGAAAGTCAAGTATGTCTCCACCGTGACCTCCGTGGCCACACCAGCTGAAATTGAAGCCCTAGGCAAAATCTTCACCGCCATGGGCCTTGCCGCCAATGAGACTGGTCCGGCCATGTGGGATCTAGCTCGTGCATATGCTGATGTGCAGAGTTCTAAATCGGCACAGCTGATTGGAGCTACCCCTTCCAACCCTGCACTATCACGCCGAGCCCTTGCTGCTCAGTTTGATCGAATCAATATAACCCCCAGGCAATTTTGCATGTACTTTGCCAAAGTTGTTTGGAACATACTTCTCGGCAGCAACATTCCACCAGCAAATTGGGCCAAACTTGGTTACCAAGAAGATACAAAATTTGCTGCATTTGACTTCTTCGATGGAGTCACCAACCCTGCCAGCCTGCAGCCTGCTGATGGTCTTATTAGGCAGCCAAATGAGAAAGAACTAGCTGCTCACTCCGTAGCTAAGTACGGCGCCTTGGCTAGGCAAAAGATCTCCACAGGTAATTATATTACCACACTTGGAGAAGTCACACGTGGACACATGGGAGGAGCTAACACCATGTACGCGATAGACGCACCCCCTAAACTTTAA

>H30_P8_a6

ATGGAAAGATCAACTCTGATTAATTTACTTCAATTGCACCACTTCGAGCCAAAACTCAGTGTTGAAGGAATCATAGTTGTGCACGGAATTGCAGGCACTGGGAAAACCACTTTACTTAGGACTTTATTTTCTGCTTACCCTAGCTTAGTTATAGGTTCACCTAGGCCTTGCTATTTAGATAAACAAAACAAAATTTCACAAGTTTGCTTATCTTGCTTTCCCAATACCCATTGTGATATTGTCGATGAGTATCATTTGCTAGAAAGTTTTCTAGAACCAAAATTGGCTATCTTTGGTGACCCCTGTCAATGCACATACATTGAGAGACTTAGAGTCCCACATTACACTTCCTTCAGAACTCATAGATTTGGAAAGTCAACTGCTGAGATTTTGAACAAACTGTTTGACCTTAATATAGTCTCAGTTAAGAAAGAAGACGACATCGTTGAATTCTTTAACCCTTTTGAAGTTGACCCCACTGAGCATATCTCTGCCTCTGAAGAAGAAGTCTTGGACTTTGTTTCTGACCAAGTGGTGACCACTAGCTCAGAGGAACTAGCAGGAATTGAGTTTGCAGAAACAACTTTCTACTGCACAACATTGGCCGCAGCTGTTGCTGAAAATCCTGCTAAGACTTTCATCTCTCTGACTAGACACACCCACAAACTCACCATTGGGGAACTAAATGCCAGGTCTAACTCCTAGATGCCAGGTCTAACTCCTAGAGCTGACCTCACTGACACATACAAAATCATTGCCATTGCTTTCTTGTTGTCAGCTTGCATTTACTTCCAAAATAGCCACTACCAACCTGTTGCTGGAGACAACTTGCACCGTTTGCCCTTTGGTGGCCAATATCAAGACGGCACCAAAAAGATATCTTATTTTCCACAACAGCAGTCATACTTTCATTCTGGAAACAAATTAAATGTCCTCATACTTATCTTCATTCTCACGTTGGGTATTGTCCTCACCAATAAATTTAGTTTTAGCTTTAGTCGTACTACTCACCAGCATTCTTGCTACAACACACATTCAGCAACCAACAATACACAACCATTGTCAGGTCATCATTGAATGTCCTCATACTTATCTTCATTCTCACGTTGGGTATTGTCCTCACCAATAAATTTAGTTTTAGCTTTAGTCGTACTACTCACCAGCATTCTTGCTACAACACACATTCAGCAACCAACAATACACAACCATTGTCAGGTCATCATTGACGGTTCTGCAATAGTCATAACAAATTGTGAGAACACACCAGAAGTGCTTAAAGCAATAAACTTCTCCCCTTGGAACGGGTTAAGTTTTCCTAAATTTGAAAATTAAATGCCTGACACAACACCTGTTGCTGCCACTTCAAGTGCACCACCCACAGCCAAAGATGCTGGTGCCAAAGCTCCTTCTGACTTCTCAAATCCCAATACAGCTCCTAGTCTCAGTGATTTGAAGAAAGTCAAGTATGTCTCCACCGTGACCTCCGTGGCCACACCAGCTGAAATTGAAGCCCTAGGCAAAATCTTCACCGCCATGGGCCTTGCCGCCAATGAGACTGGTCCGGCCATGTGGGATCTAGCTCGTGCATATGCTGATGTGCAGAGTTCTAAATCGGCACAGCTGATTGGAGCTACCCCTTCCAACCCTGCACTATCACGCCGAGCCCTTGCTGCTCAGTTTGATCGAATCAATATAACCCCCAGGCAATTTTGCATGTACTTTGCCAAAGTTGTTTGGAACATACTTCTCGACAGCAACATTCCACCAGCAAATTGGGCCAAACTTGGTTACCAAGAAGATACAAAATTTGCTGCATTTGACTTCTTCGATGGAGTCACCAACCCTGCCAGCCTGCAGCCTGCTGATGGTCTTATCAGGCAGCCAAATGAGAAAGAACTAGCTGCTCACTCCGTAGCTAAGTACGGCGCCTTGGCTAGGCAAAAGATCTCCACAGGTAATTATATTACCACACTTGGAGAAGTCACACGTGGACACATGGGAGGAGCTAACACCATGTACGCGATAGACGCACCCCCTAAACTTTAA

>H30_P8_a5

ATGGAAAGATCAACTCTGATTAATTTACTTCAATTGCACCACTTCGAGCCAAAACTCAGTGTTGAAGGAATCATAGTTGTGCACGGAATTGCAGGCACTGGGAAAACCACTTTACTTAGGACTTTATTTTCTGCTTACCCTAGCTTAGTTATAGGTTCACCTAGGCCTTGCTATTTAGATAAACAAAACAAAATTTCACAAGTTTGCTTATCTTGCTTTCCCAATACCCATTGTGATATTGTCGATGAGTATCATTTGCTAGAAAGTTTTCTAGAACCAAAATTGGCTATCTTTGGTGACCCCTGTCAATGCACATACATTGAGAGACTTAGAGTCCCACATTACACTTCCTTCAGAACTCATAGATTTGGAAAGTCAACTGCTGAGATTTTGAACAAACTGTTTGACCTTAATATAGTCTCAGTTAAGAAAGAAGACGACATCGTTGAATTCTTTAACCCTTTTGAAGTTGACCCCACTGAGCATATCTCTGCCTCTGAAGAAGAAGTCTTGGACTTTGTTTCTGACCAAGTGGTGACCACTAGCTCAGAGGAACTAGCAGGAATTGAGTTTGCAGAAACAACTTTCTACTGCACAACATTGGCCGCAGCTGTTGCTGAAAATCCTGCTAAGACTTTCATCTCTCTGACTAGACACACCCACAAACTCACCATTGGGGAACTAAATGCCAGGTCTAACTCCTAGATGCCAGGTCTAACTCCTAGAGCTGACCTCACTGACACATACAAAATCATTGCCATTGCTTTCTTGTTGTCAGCTTGCATTTACTTCCAAAATAGCCACTACCAACCTGTTGCTGGAGACAACTTGCACCGTTTGCCTTTTGGTGGCCAATATCAAGACGGCACCAAAAAGATATCTTATTTTCCACAACAGCAGTCATACTTTCATTCTGGAAACAAATTAAATGTCCTCATACTTATCTTCATTCTCACGTTGGGTATTGTCCTCACCAATAAATTTAGTTTTAGCTTTAGTCGTACTACTCACCAGCATTCTTGCTATAACACACATTCAGCAACCAACAATACACAACCATTGTCAGGTCATCATTGAATGTCCTCATACTTATCTTCATTCTCACGTTGGGTATTGTCCTCACCAATAAATTTAGTTTTAGCTTTAGTCGTACTACTCACCAGCATTCTTGCTATAACACACATTCAGCAACCAACAATACACAACCATTGTCAGGTCATCATTGACGGTTCTGCAATAGTCATAACAAATTGTGAGAACACACCAGAAGTGCTTAAAGCAATAAACTTCTCCCCTTGGAACGGGTTAAGTTTTCCTAAATTTGAAAATTAAATGCCTGACACAACACCTGTTGCTGCCACTTCAAGTGCACCACCCACAGCCAAAGATGCTGGTGCCAAAGCTCTTTCTGACTTCTCAAATCCCAATACAGCTCCTAGTCTCAGTGATTTGAAGAAAGTCAAGTATGTCTCCACCGTGACCTCCGTGGCCACACCAGCTGAAATTGAAGCCCTAGGCAAAATCTTCACCGCCATGGGCCTTGCCGCCAATGAGACTGGTCCGGCCATGTGGGATCTAGCTCGTGCATATGCTGATGTGCAGAGTTCTAAATCGGCACAGCTGATTGGAGCTACCCCTTCCAACCCTGCACTATCACGCCGAGCCCTTGCTGCTCAGTTTGATCGAATCAATATAACCCCCAGGCAATTTTGCATGTACTTTGCCAAAGTTGTTTGGAACATACTTCTCGACAGCAACATTCCACCAGCAAATTGGGCCAAACTTGGTTACCAAGAAGATACAAAATTTGCTGCATTTGACTTCTTCGATGGAGTCACCAACCCTGCCAGCCTGCAGCCTGCTGATGGTCTTATCAGGCAGCCAAATGAGAAAGAACTAGCTGCTCACTCCGTAGCTAAGTACGGCGCCTTGGCTAGGCAAAAGATCTCCACAGGTAATTATATTACCACACTTGGAGAAGTCACACGTGGACACATGGGAGGAGCTAACACCATGTACGCGATAGACGCACCCCCTAAACTTTAA

>H30_P8_a4

ATGGAAAGATCAACTCTGATTAATTTACTTCAATTGCACCACTTCGAGCCAAAACTCAGTGTTGAAGGAATCATAGTTGTGCACGGAATTGCAGGCACTGGGAAAACCACTTTACTTAGGACTTTATTTTCTGCTTACCCTAGCTTAGTTATAGGTTCACCTAGGCCTTGCTATTTAGATAAACAAAACAAAATTTCACAAGTTTGCTTATCTTGCTTTCCCAATACCCATTGTGATATTGTCGATGAGTATCATTTGCTAGAAAGTTTTCTAGAACCAAAATTGGCTATCTTTGGTGACCCCTGTCAATGCACATACATTGAGAGACTTAGAGTCCCACATTACACTTCCTTCAGAACTCATAGATTTGGAAAGTCAACTGCTGAGATTTTGAACAAACTGTTTGACCTTAATATAGTCTCAGTTAAGAAAGAAGACGACATCGTTGAATTCTTTAACCCTTTTGAAGTTGACCCCACTGAGCATATCTCTGCCTCTGAAGAAGAAGTCTTGGACTTTGTTTCTGACCAAGTGGTGACCACTAGCTCAGAGGAACTAGCAGGAATTGAGTTTGCAGAAACAACTTTCTACTGCACAACATTGGCCGCAGCTGTTGCTGAAAATCCTGCTAAGACTTTCATCTCTCTGACTAGACACACCCACAAACTCACCATTGGGGAACTAAATGCCAGGTCTAACTCCTAGATGCCAGGTCTAACTCCTAGAGCTGACCTCACTGACACATACAAAATCATTGCCATTGCTTTCTTGTTGTCAGCTTGCATTTACTTCCAAAATAGCCACTACCAACCTGTTGCTGGAGACAACTTGCACCGTTTGCCTTTTGGTGGCCAATATCAAGACGGCACCAAAAAGATATCTTATTTTCCACAACAGCAGTCATACTTTCATTCTGGAAACAAATTAAATGTCCTCATACTTATCTTCATTCTCACGTTGGGTATTGTCCTCACCAATAAATTTAGTTTTAGCTTTAGTCGTACTACTCACCAGCATTCTTGCTACAACACACATTCAGCAACCAACAATACACAACCATTGTCAGGTCATCATTGAATGTCCTCATACTTATCTTCATTCTCACGTTGGGTATTGTCCTCACCAATAAATTTAGTTTTAGCTTTAGTCGTACTACTCACCAGCATTCTTGCTACAACACACATTCAGCAACCAACAATACACAACCATTGTCAGGTCATCATTGACGGTTCTGCAATAGTCATAACAAATTGTGAGAACACACCAGAAGTGCTTAAAGCAATAAACTTCTCCCCTTGGAACGGGTTAAGTTTTCCTAAATTTGAAAATTAAATGCCTGACACAACACCTGTTGCTGCCACTTCAAGTGCACCACCCACAGCCAAAGATGCTGGTGCCAAAGCTCCTTCTGACTTCTCAAATCCCAATACAGCTCCTAGTCTCAGTGATTTGAAGAAAGTCAAGTATGTCTCCACCGTGACCTCCGTGGCCACACCAGCTGAAATTGAAGCCCTAGGCAAAATCTTCACCGCCATGGGCCTTGCCGCCAATGAGACTGGTCCGGCCATGTGGGATCTAGCTCGTGCATATGCTGATGTGCAGAGTTCTAAATCGGCACAGCTGATTGGAGCTACCCCTTCCAACCCTGCACTATCACGCCGAGCCCTTGCTGCTCAGTTTGATCGAATCAATATAACCCCCAGGCAATTTTGCATGTACTTTGCCAAAGTTGTTTGGAACATACTTCTCGACAGCAACATTCCACCAGCAAATTGGGCCAAACTTGGTTACCAAGAAGATACAAAATTTGCTGCATTTGACTTCTTCGATGGAGTCACCAACCCTGCCAGCCTGCAGCCTGCTGATGGTCTTATTAGGCAGCCAAATGAGAAAGAACTAGCTGCTCACTCCGTAGCTAAGTACGGCGCCTTGGCTAGGCAAAAGATCTCCACAGGTAATTATATTACCACACTTGGAGAAGTCACACGTGGACACATGGGAGGAGCTAACACCATGTACGCGATAGACGCACCCCCTAAACTTTAA

>H30_P8_a3

ATGGAAAGATCAACTCTGATTAATTTACTTCAATTGCACCACTTCGAGCCAAAACTCAGTGTTGAAGGAATCATAGTTGTGCACGGAATTGCAGGCACTGGGAAAACCACTTTACTTAGGACTTTATTTTCTGCTTACCCTAGCTTAGTTATAGGTTCACCTAGGCCTTGCTATTTAGATAAACAAAACAAAATTTCACAAGTTTGCTTATCTTGCTTTCCCAATACCCATTGTGATATTGTCGATGAGTATCATTTGCTAGAAAGTTTTCTAGAACCAAAATTGGCTATCTTTGGTGACCCCTGTCAATGCACATACATTGAGAGACTTAGAGTCCCACATTACACTTCCTTCAGAACTCATAGATTTGGAAAGTCAACTGCTGAGATTTTGAACAAACTGTTTGACCTTAATATAGTCTCAGTTAAGAAAGAAGACGACATCGTTGAATTCTTTAACCCTTTTGAAGTTGACCCCACTGAGCATATCTCTGCCTCTGAAGAAGAAGTCTTGGACTTTGTTTCTGACCAAGTGGTGACCACTAGCTCAGAGGAACTAGCAGGAATTGAGTTTGCAGAAACAACTTTCTACTGCACAACATTGGCCGCAGCTGTTGCTGAAAATCCTGCTAAGACTTTCATCTCTCTGACTAGACACACCCACAAACTCACCATTGGGGAACTAAATGCCAGGTCTAACTCCTAGATGCCAGGTCTAACTCCTAGAGCTGACCTCACTGACACATACAAAATCATTGCCATTGCTTTCTTGTTGTCAGCTTGCATTTACTTCCAAAATAGCCACTACCAACCTGTTGCTGGAGACAACTTGCACCGTTTGCCTTTTGGTGGCCAATATCAAGACGGCACCAAAAAGATATCTTATTTTCCACAACAGCAGTCATACTTTCATTCTGGAAACAAATTAAATGTCCTCATACTTATCTTCATTCTCACGTTGGGTATTGTCCTCACCAATAAATTTAGTTTTAGCTTTAGTCGTACTACTCACCAGCATTCTTGCTACAACACACATTCAGCAACCAACAATACACAACCATTGTCAGGTCATCATTGAATGTCCTCATACTTATCTTCATTCTCACGTTGGGTATTGTCCTCACCAATAAATTTAGTTTTAGCTTTAGTCGTACTACTCACCAGCATTCTTGCTACAACACACATTCAGCAACCAACAATACACAACCATTGTCAGGTCATCATTGACGGTTCTGCAATAGTCATAACAAATTGTGAGAACACACCAGAAGTGCTTAAAGCAATAAACTTCTCCCCTTGGAACGGGTTAAGTTTTCCTAAATTTGAAAATTAAATGCCTGACACAACACCTGTTGCTGCCACTTCAAGTGCACCACCCACAGCCAAAGATGCTGGTGCCAAAGCTCCTTCTGACTTCTCAAATCCCAATACAGCTCCTAGTCTCAGTGATTTGAAGAAAGTCAAGTATGTCTCCACAGTGACCTCCGTGGCCACACCAGCTGAAATTGAAGCCCTAGGCAAAATCTTCACCGCCATGGGCCTTGCCGCCAATGAGACTGGTCCGGCCATGTGGGATCTAGCTCGTGCATATGCTGATGTGCAGAGTTCTAAATCGGCACAGCTGATTGGAGCTACCCCTTCCAACCCTGCACTATCACGCCGAGCCCTTGCTGCTCAGTTTGATCGAATCAATATAACCCCCAGGCAATTTTGCATGTACTTTGCCAAAGTTGTTTGGAACATACTTCTCGACAGCAACATTCCACCAGCAAATTGGGCCAAACTTGGTTACCAAGAAGATACAAAATTTGCTGCATTTGACTTCTTCGATGGAGTCACCAACCCTGCCAGCCTGCAGCCTGCTGATGGTCTTATCAGGCAGCCAAATGAGAAAGAACTAGCTGCTCACTCCGTAGCTAAGTACGGCGCCTTGGCTAGGCAAAAGATCTCCACAGGTAATTATATTACCACACTTGGAGAAGTCACACGTGGACACATGGGAGGAGCTAACACCATGTACGCGATAGACGCACCCCCTAAACTTTAA

>H30_P8_a2

ATGGAAAGATCAACTCTGATTAATTTACTTCAATTGCACCACTTCGAGCCAAAACTCAGTGTTGAAGGAATCATAGTTGTGCACGGAATTGCAGGCACTGGGAAAACCACTTTACTTAGGACTTTATTTTCTGCTTACCCTAGCTTAGTTATAGGTTCACCTAGGCCTTGCTATTTAGATAAACAAAACAAAATTTCACAAGTTTGCTTATCTTGCTTTCCCAATACCCATTGTGATATTGTCGATGAGTATCATTTGCTAGAAAGTTTTCTAGAACCAAAATTGGCTATCTTTGGTGACCCCTGTCAATGCACATACATTGAGAGACTTAGAGTCCCACATTACACTTCCTTCAGAACTCATAGATTTGGAAAGTCAACTGCTGAGATTTTGAACAAACTGTTTGACCTTAATATAGTCTCAGTTAAGAAAGAAGACGACATCGTTGAATTCTTTAACCCTTTTGAAGTTGACCCCACTGAGCATATCTCTGCCTCTGAAGAAGAAGTCTTGGACTTTGTTTCTGACCAAGTGGTGACCACTAGCTCAGAGGAACTAGCAGGAATTGAGTTTGCAGAAACAACTTTCTACTGCACAACATTGGCCGCAGCTGTTGCTGAAAATCCTGCTAAGACTTTCATCTCTCTGACTAGACACACCCACAAACTCACCATTGGGGAACTAAATGCCAGGTCTAACTCCTAGATGCCAGGTCTAACTCCTAGAGCTGACCTCACTGACACATACAAAATCATTGCCATTGCTTTCTTGTTGTCAGCTTGCATTTACTTCCAAAATAGCCACTACCAACCTGTTGCTGGAGACAACTTGCACCGTTTGCCTTTTGGTGGCCAATATCAAGACGGCACCAAAAAGATATCTTATTTTCCACAACAGCAGTCATACTTTCATTCTGGAAACAAATTAAATGTCCTCATACTTATCTTCATTCTCACGTTGGGTATTGTCCTCACCAATAAATTTAGTTTTAGCTTTAGTCGTACTACTCACCAGCATTCTTGCTACAACACACATTCAGCAACCAACAATACACAACCATTGTCAGGTCATCATTGAATGTCCTCATACTTATCTTCATTCTCACGTTGGGTATTGTCCTCACCAATAAATTTAGTTTTAGCTTTAGTCGTACTACTCACCAGCATTCTTGCTACAACACACATTCAGCAACCAACAATACACAACCATTGTCAGGTCATCATTGACGGTTCTGCAATAGTCATAACAAATTGTGAGAACACACCAGAAGTGCTTAAAGCAATAAACTTCTCCCCTTGGAACGGGTTAAGTTTCCCTAAATTTGAAAATTAAATGCCTGACACAACACCTGTTGCTGCCACTTCAAGTGCACCACCCACAGCCAAAGATGCTGGTGCCAAAGCTCCTTCTGACTTCTCAAATCCCAATACAGCTCCTAGTCTCAGTGATTTGAAGAAAGTCAAGTATGTCTCCACCGTGACCTCCGTGGCCACACCAGCTGAAATTGAAGCCCTAGGCAAAATCTTCACCGCCATGGGCCTTGCCGCCAATGAGACTGGTCCGGCCATGTGGGATCTAGCTCGTGCATATGCTGATGTGCAGAGTTCTAAATCGGCACAGCTGATTGGAGCTACCCCTTCCAACCCTGCACTATCACGCCGAGCCCTTGCTGCTCAGTTTGATCGAATCAATATAACCCCCAGGCAATTTTGCATGTACTTTGCCAAAGTTGTTTGGAACATACTTCTCGACAGCAACATTCCACCAGCAAATTGGGCCAAACTTGGTTACCAAGAAGATACAAAATTTGCTGCATTTGACTTCTTCGATGGGGTCACCAACCCTGCCAGCCTGCAGCCTGCTGATGGTCTTATTAGGCAGCCGAATGAGAAAGAACTAGCTGCTCACTCCGTAGCTAAGTACGGCGCCTTGGCTAGGCAAAAGATCTCCACAGGTAATTATATTACCACACTTGGAGAAGTCACACGTGGACACATGGGAGGAGCTAACACCATGTACGCGATAGACGCACCCCCTAAACTTTAA

>H30_P8_a1

ATGGAAAGATCAACTCTGATTAATTTACTTCAATTGCACCACTTCGAGCCAAAACTCAGTGTTGAAGGAATCATAGTTGTGCACGGAATTGCAGGCACTGGGAAAACCACTTTACTTAGGACTTTATTTTCTGCTTACCCTAGCTTAGTTATAGGTTCACCTAGGCCTTGCTATTTAGATAAACAAAACAAAATTTCACAAGTTTGCTTATCTTGCTTTCCCAATACCCATTGTGATATTGTCGATGAGTATCATTTGCTAGAAAGTTTTCTAGAACCAAAATTGGCTATCTTTGGTGACCCCTGTCAATGCACATACATTGAGAGACTTAGAGTCCCACATTACACTTCCTTCAGAACTCATAGATTTGGAAAGTCAACTGCTGAGATTTTGAACAAACTGTTTGACCTTAATATAGTCTCAGTTAAGAAAGAAGACGACATCGTTGAATTCTTTAACCCTTTTGAAGTTGACCCCACTGAGCATATCTCTGCCTCTGAAGAAGAAGTCTTGGACTTTGTTTCTGACCAAGTGGTGACCACTAGCTCAGAGGAACTAGCAGGAATTGAGTTTGCAGAAACAACTTTCTACTGCACAACATTGGCCGCAGCTGTTGCTGAAAATCCTGCTAAGACTTTCATCTCTCTGACTAGACACACCCACAAACTCACCATTGGGGAACTAAATGCCAGGTCTAACTCCTAGATGCCAGGTCTAACTCCTAGAGCTGACCTCACTGACACATACAAAATCATTGCCATTGCTTTCTTGTTGTCAGCTTGCATTTACTTCCAAAATAGCCACTACCAACCTGTTGCTGGAGACAACTTGCACCGTTTGCCTTTTGGTGGCCAATATCAAGACGGCACCAAAAAGATATCTTATTTTCCACAACAGCAGTCATACTTTCATTCTGGAAACAAATTAAATGTCCTCATACTTATCTTCATTCTCACGTTGGGTATTGTCCTCACCAATAAATTTAGTTTTAGCTTTAGTCGTACTACTCACCAGCATTCTTGCTACAACACACATTCAGCAACCAACAATACACAACCATTGTCAGGTCATCATTGAATGTCCTCATACTTATCTTCATTCTCACGTTGGGTATTGTCCTCACCAATAAATTTAGTTTTAGCTTTAGTCGTACTACTCACCAGCATTCTTGCTACAACACACATTCAGCAACCAACAATACACAACCATTGTCAGGTCATCATTGACGGTTCTGCAATAGTCATAACAAATTGTGAGAACACACCAGAAGTGCTTAAAGCAATAAACTTCTCCCCTTGGAACGGGTTAAGTTTTCCTAAATTTGAAAATTAAATGCCTGACACAACACCTGTTGCTGCCACTTCAAGTGCACCACCCACAGCCAAAGATGCTGGTGCCAAAGCTCCTTCTGACTTCTCAAATCCCAATACAGCTCCTAGTCTCAGTGATTTGAAGAAAGTCAAGTATGTCTCCACCGTGACCTCCGTGGCCACACCAGCTGAAATTGAAGCCCTAGGCAAAATCTTCACCGCCATGGGCCTTGCCGCCAATGAGACTGGTCCGGCCATGTGGGATCTAGCTCGTGCATATGCTGATGTGCAGAGTTCTAAATCGGCACAGCTGATTGGAGCTACCCCTTCCAACCCTGCACTATCACGCCGAGCCCTTGCTGCTCAGTTTGATCGAATCAATATAACCCCCAGGCAATTTTGCATGTACTTTGCCAAAGTTGTTTGGAACATACTTCTCGACAGCAACATTCCACCAGCAAATTGGGCCAAACTTGGTTACCAAGAAGATACAAAATTTGCTGCATTTGACTTCTTCGATGGAGTCACCAACCCTGCCAGCCTGCAGCCTGCTGATGGTCTTATTAGGCAGCCAAATGAGAAAGAACTAGCTGCTCACTCCGTAGCTAAGTACGGCGCCTTGGCTAGGCAAAAGATCTCCACAGGTAATTATATTACCACACTTGGAGAAGTCACACGTGGACACATGGGAGGAGCTAACACCATGTACGCGATAGACGCACCCCCTAAACTTTAA

>KLP2_P1_consensus_sequence

ATGGAAAGATCAACTTTGATCAATTTACTTCTGTTACACAAATTTGAACACAAGATTAACACTGAAGGAATCATTGTTGTGCACGGAATTGCTGGAACTGGGAAAACCACATTGCTTAGGACTTTATTTTCTGCATACCCTAGCTTAGTTATAGGTTCACCTAGGCCTTGCTACTTAGATAAAGCTAATAAAATTTCACAAGTTTGCCTTTCTTGTTTTCCAAATACCTTGTGTGACATTGTTGACGAGTACCATCTCTTAGAAAGTTTTCCTGAACCAAAACTAGCCATTTTTGGTGACCCCTGTCAGTGCACTTACATTGAAAGGTTGAGAACACCCAACTACACATCCTTCAGAACACACCGATTTGGCAAATCCACTGCTGCTCTACTAAACAAGTTATTTGATCTTAACATTGAGTCAGTCAAAGCACAAGACGACACAGTGGAATACTTTGATCCTTTCGCAGTGGACCCCTCTGAACACATTTCTGCATCAGAAAAAGAAGTTTTGGAATTTGTAGGTGATCAAGTTGAGACTACAAGCTCTGAAGAACTAGCTGGTCTCGAGTTTAGTGAAGTTACTTTCTACTGTACCACACTTGCTGGTGCTGTTCAAGAAAATCCTGCCAAAACTTTCATTTCACTAACTAGACACACTTCAAAGCTCACAATTGGTGAACTAAATGCCAGGTCTGACTCCTAGATGCCAGGTCTGACTCCTAGAGCTGACCTTACTGACACGTATAAAATCATTGCTATAGCCCTTCTACTGTCAGCTTGCATTTACTTCCAAAACAGTCATTATCAACCAGTTGCAGGTGATAACTTGCACAGACTACCCTTCGGTGGCCAGTATCAAGACGGAACTAAGAAGATTTCTTACTTTCCGCAGCAACAATCCTACTTTCACTCAGGAAACAAGCTTAATGTCCTCATACTTATCTTCATTCTTACACTGGGTATTGTCCTCACCAATAAATTTAGTTTTAGCATTAGCCGTAATACTCACCAGCATCATTGCTACAATACACATTCTGCAACCCAAACAGGTCAATCAGTGCCAGGTCATCATTGAATGTCCTCATACTTATCTTCATTCTTACACTGGGTATTGTCCTCACCAATAAATTTAGTTTTAGCATTAGCCGTAATACTCACCAGCATCATTGCTACAATACACATTCTGCAACCCAAACAGGTCAATCAGTGCCAGGTCATCATTGACGGTGCAGCCATAGTTATAACAAATTGTCCAAACACACCCGAAGTTCTTAAAGCAATCAACTTCTCCCCTTGGAACGGGTTAAGTTTTCCTCAATTGTGAATGGAAAACCAACCTACAGCTTCTAACCCATCAGATGTACCACCAACTGCCGCTCAAGCTGGTGCCCAGAGCCCGGCCGACTTCTCAAATCCTAATACAGCTCCTTCCCTAAGTGATTTGAAGAAAATCAAATACGTGTCAACTGTCACTTCAGTTGCCACGCCTGCTGAAATTGAGGCCCTTGGCAAGATCTTTACTGCCATGGGTTTAGCAGCCAATGAGACCGGACCTGCCATGTGGGACCTCGCTCGTGCTTATGCTGATGTGCAAAGTTCAAAATCTGCACAACTTATAGGTGCCACACCATCCAACCCTGCTTTGTCTAGACGTGCACTTGCTGCACAGTTTGATCGTATCAATATCACACCCAGACAATTCTGCATGTATTTTGCAAAAATTGTTTGGAACATACTGTTAGACAGCAATGTGCCACCTGCCAACTGGGCAAAATTGGGCTATCAGAAAGATACCAAATTTGCTGCTTTTGACTTCTTTGATGGAGTCACAAATCCAGCTAGTCTACAGCCTGCAGATGGCCTAATCAGGCAGCCCAATGAAAAAGAGCTTGCTGCCCACTCGGTTGCTAAATATGGCGCCCTTGCCCGCCAGAAAATATCCACTGGTAACTACATCACCACCCTTGGTGAAGTTACACGTGGTCACATGGGCGGCGCCAACACTATGTACGCAATTGATGCACCTCCTGAACTTTAA

>KLP2_P8_b14

ATGGAAAGATCAACTTTGATCAATTTACTTCTGTTACACAAATTTGAACACAAGATTAACACTGAAGGAATCATTGTTGTGCACGGAATTGCTGGAACTGGGAAAACCACATTGCTTAGGACTTTATTTTCTGCATACCCTAGCTTAGTTATAGGTTCACCTAGGCCTTGCTACTTAGATAAAGCTAATAAAATTTCACAAGTTTGCCTTTCTTGTTTTCCAAATACCTTGTGTGACATTGTTGACGAGTACCATCTCTTAGAAAGTTTTCCTGAACCAAAACTAGCCATTTTTGGTGACCCCTGTCAGTGCACTTACATTGAAAGGTTGAGAACACCCAACTACACATCCTTCAGAACACACCGATTTGGCAAATCCACTGCTGCTCTACTAAACAAGTTATTTGATCTTAACATTGAGTCAGTCAAAGCACAAGACGACACAGTGGAATACTTTGATCCTTTCGCAGTGGACCCCTCTGAACACATTTCTGCATCAGAAAAAGAAGTTTTGGAATTTGTAGGTGATCAAGTTGAGACTACAAGCTCTGAAGAACTAGCTGGTCTCGAGTTTAGTGAAGTTACTTTCTACTGTACCACACTTGCTGGTGCTGTTCAAGAAAATCCTGCCAAAACTTTCATTTCACTAACTAGACACACTTCAAAGCTCACAATTGGTGAACTAAATGCCAGGTCTGACTCCTAGATGCCAGGTCTGACTCCTAGAGCTGACCTTACTGACACGTATAAAATCATTGCTATAGCCCTTCTACTGTCAGCTTGCATTTACTTCCAAAACAGTCATTATCAACCAGTTGCAGGTGATAACTTGCACAGACTACCCTTCGGTGGCCAGTATCAAGACGGGACTAAGAAGATTTCTTACTTTCCGCAGCAACAATCCTACTTTCACTCAGGAAACAAGCTTAATGTCCTCATACTTATCTTCATTCTTACACTGGGTATTGTCCTCACCAATAAATTTAGTTTTAGCATTAGCCGTAATACTCACCAGCATCATTGCTACAATACACATTCTGCAACCCAAACAGGTCAATCAGTGCCAGGTCATCATTGAATGTCCTCATACTTATCTTCATTCTTACACTGGGTATTGTCCTCACCAATAAATTTAGTTTTAGCATTAGCCGTAATACTCACCAGCATCATTGCTACAATACACATTCTGCAACCCAAACAGGTCAATCAGTGCCAGGTCATCATTGACGGTGCAGCCATAGTTATAACAAATTGTCCAAACACACCCGAAGTTCTTAAAGCAATCAACTTCTCCCCTTGGAACGGGTTAAGTTTTCCTCAATTGTGAATGGAAAACCAACCTACAGCTTCTAACCCATCAGATGTACCACCAACTGCCGCTCAAGCTGGTGCCCAGAGCCCGGCCGACTTCTCAAATCCTAATACAGCTCCTTCCCTAAGTGATTTGAAGAAAATCAAATACGTGTCAACTGTCACTTCAGTTGCCACGCCTGCTGAAATTGAGGCCCTTGGCAAGATCTTTACTGCCATGGGTTTAGCAGCCAATGAGACCGGACCTGCCATGTGGGACCTCGCTCGTGCTTATGCTGATGTGCAAAGTTCAAAATCTGCACAACTTATAGGTGCCACACCATCCAACCCTGCTTTGTCTAGACGTGCACTTGCTGCACAGTTTGATCGTATCAATATCACACCCAGACAATTCTGCATGTATTTTGCAAAAATTGTTTGGAACATACTGTTAGACAGCAATGTGCCACCTGCCAACTGGGCAAAATTGGGCTATCAGAAAGATACCAAATTTGCTGCTTTTGACTTCTTTGATGGAGTCACAAATCCAGCTAGTCTACAGCCTGCAGATGGCCTAATCAGGCAGCCCAATGAAAAAGAGCTTGCTGCCCACTCGGTTGCTAAATATGGCGCCCTTGCCCGCCAGAAAATATCCACTGGTAACTACATCACCACCCTTGGTGAAGTTACACGTGGTCACATGGGCGGCGCCAACACTATGTACGCAATTGATGCACCTCCTGAACTTTAA

>KLP2_P8_b13

ATGGAAAGATCAACTTTGATCAATTTACTTCTGTTACACAAATTTGAACACAAGATTAACACTGAAGGAATCATTGTTGTGCACGGAATTGCTGGAACTGGGAAAACCACATTGCTTAGGACTTTATTTTCTGCATACCCTAGCTTAGTTATAGGTTCACCTAGGCCTTGCTACTTAGATAAAGCTAATAAAATTTCACAAGTTTGCCTTTCTTGTTTTCCAAATACCTTGTGTGACATTGTTGACGAGTACCATCTCTTAGAAAGTTTTCCTGAACCAAAACTAGCCATTTTTGGTGACCCCTGTCAGTGCACTTACATTGAAAGGTTGAGAACACCCAACTACACATCCTTCAGAACACACCGATTTGGCAAATCCACTGCTGCTCTACTAAACAAGTTATTTGATCTTAACATTGAGTCAGTCAAAGCACAAGACGACACAGTGGAATACTTTGATCCTTTCGCAGTGGACCCCTCTGAACACATTTCTGCATCAGAAAAAGAAGTTTTGGAATTTGTAGGTGATCAAGTTGAGACTACAAGCTCTGAAGAACTAGCTGGTCTCGAGTTTAGTGAAGTTACTTTCTACTGTACCACACTTGCTGGTGCTGTTCAAGAAAATCCTGCCAAAACTTTCATTTCACTAACTAGACACACTTCAAAGCTCACAATTGGTGAACTAAATGCCAGGTCTGACTCCTAGATGCCAGGTCTGACTCCTAGAGCTGACCTTACTGACACGTATAAAATCATTGCTATAGCCCTTCTACTGTCAGCTTGCATTTACCTCCAAAACAGTCATTATCAACCAGTTGCAGGTGATAACTTGCACAGACTACCCTTCGGTGGCCAGTATCAAGACGGGACTAAGAAGATTTCTTACTTTCCGCAGCAACAATCCTACTTTCACTCAGGAAACAAGCTTAATGTCCTCATACTTATCTTCATTCTTACACTGGGTATTGTCCTCACCAATAAATTTAGTTTTAGCATTAGCCGTAATACTCACCAGCATCATTGCTACAATACACATTCTGCAACCCAAACAGGTCAATCAGTGCCAGGTCATCATTGAATGTCCTCATACTTATCTTCATTCTTACACTGGGTATTGTCCTCACCAATAAATTTAGTTTTAGCATTAGCCGTAATACTCACCAGCATCATTGCTACAATACACATTCTGCAACCCAAACAGGTCAATCAGTGCCAGGTCATCATTGACGGTGCAGCCATAGTTATAACAAATTGTCCAAACACACCCGAAGTTCTTAAAGCAATCAACTTCTCCCCTTGGAACGGGTTAAGTTTTCCTCAATTGTGAATGGAAAACCAACCTACAGCTTCTAACCCATCAGATGTACCACCAACTGCCGCTCAAGCTGGTGCCCAGAGCCCGGCCGACTTCTCAAATCCTAATACAGCTCCTTCCCTAAGTGATTTGAAGAAAATCAAATACGTGTCAACTGTCACTTCAGTTGCCACGCCTGCTGAAATTGAGGCCCTTGGCAAGATCTTTACTGCCATGGGTTTAGCAGCCAATGAGACCGGACCTGCCATGTGGGACCTCGCTCGTGCTTATGCTGATGTGCAAAGTTCAAAATCTGCACAACTTATAGGTGCCACACCATCCAACCCTGCTTTGTCTAGACGTGCACTTGCTGCACAGTTTGATCGTATCAATATCACACCCAGACAATTCTGCATGTATTTTGCAAAAATTGTTTGGAACATACTGTTAGACAGCAATGTGCCACCTGCCAACTGGGCAAAATTGGGCTATCAGAAAGATACCAAATTTGCTGCTTTTGACTTCTTTGATGGAGTCACAAATCCAGCTAGTCTACAGCCTGCAGATGGCCTAATCAGGCAGCCCAATGAAAAAGAGCTTGCTGCCCACTCGGTTGCTAAATATGGCGCCCTTGCCCGCCAGAAAATATCCACTGGTAACTACATCACCACCCTTGGTGAAGTTACACGTGGTCACATGGGCGGCGCCAACACTATGTACGCAATTGATGCACCTCCTGAACTTTAA

>KLP2_P8_b12

ATGGAAAGATCAACTTTGATCAATTTACTTCTGTTACACAAATTTGAACACAAGATTAACACTGAAGGAATCATTGTTGTGCACGGAATTGCTGGAACTGGGAAAACCACATTGCTTAGGACTTTATTTTCTGCATACCCTAGCTTAGTTATAGGTTCGCCTAGGCCTTGCTACTTAGATAAAGCTAATAAAATTTCACAAGTTTGCCTTTCTTGTTTTCCAAATACCTTGTGTGACATTGTTGACGAGTACCATCTCTTAGAAAGTTTTCCTGAACCAAAACTAGCCATTTTTGGTGACCCCTGTCAGTGCACTTACATTGAAAGGTTGAGAACACCCAACTACACATCCTTCAGAACACACCGATTTGGCAAATCCACTGCTGCTCTACTAAACAAGTTATTTGATCTTAACATTGAGTCAGTCAAAGCACAAGACGACACAGTGGAATACTTTGATCCTTTCGCAGTGGACCCCTCTGAACACATTTCTGCATCAGAAAAAGAAGTTTTGGAATTTGTAGGTGATCAAGTTGAGACTACAAGCTCTGAAGAACTAGCTGGTCTCGAGTTTAGTGAAGTTACTTTCTACTGCACCACACTTGCTGGTGCTGTTCAAGAAAATCCTGCCAAAACTTTCATTTCACTAACTAGACACACTTCAAAGCTCACAATTGGTGAACTAAATGCCAGGTCTGACTCCTAGATGCCAGGTCTGACTCCTAGAGCTGACCTTACTGACACGTATAAAATCATTGCTATAGCCCTTCTACTGTCAGCTTGCATTTACTTCCAAAACAGTCATTATCAACCAGTTGCAGGTGATAACTTGCACAGACTACCCTTCGGTGGCCAGTATCAAGACGGGACTAAGAAGATTTCTTACTTTCCGCAGCAACAATCCTACTTTCACTCAGGAAACAAGCTTAATGTCCTCATACTTATCTTCATTCTTACACTGGGTATTGTCCTCACCAATAAATTTAGTTTTAGCATTAGCCGTAATACTCACCAGCATCATTGCTACAATACACATTCTGCAACCCAAACAGGTCAATCAGTGCCAGGTCATCATTGAATGTCCTCATACTTATCTTCATTCTTACACTGGGTATTGTCCTCACCAATAAATTTAGTTTTAGCATTAGCCGTAATACTCACCAGCATCATTGCTACAATACACATTCTGCAACCCAAACAGGTCAATCAGTGCCAGGTCATCATTGACGGTGCAGCCATAGTTATAACAAATTGTCCAAACACACCCGAAGTTCTTAAAGCAATCAACTTCTCCCCTTGGAACGGGTTAAGTTTTCCTCAATTGTGAATGGAAAACCAACCTACAGCTTCTAACCCATCAGATGTACCACCAACTGCCGCTCAAGCTGGTGCCCAGAGCCCGGCCGACTTCTCAAATCCTAATACAGCTCCTTCCCTAAGTGATTTGAAGAAAATCAAATACGTGTCAACTGTCACTTCAGTTGCCACGCCTGCTGAAATTGAGGCCCTTGGCAAGATCTTTACTGCCATGGGTTTAGCAGCCAATGAGACCGGACCTGCCATGTGGGACCTCGCTCGTGCTTATGCTGATGTGCAAAGTTCAAAATCTGCACAACTTATAGGTGCCACACCATCCAACCCTGCTTTGTCTAGACGTGCACTTGCTGCACAGTTTGATCGTATCAATATCACACCCAGACAATTCTGCATGTATTTTGCAAAAATTGTTTGGAACATACTGTTAGACAGCAATGTGCCACCTGCCAACTGGGCAAAATTGGGCTATCAGAAAGATACCAAATTTGCTGCTTTTGACTTCTTTGATGGAGTCACAAATCCAGCTAGTCTACAGCCTGCAGATGGCCTAATCAGGCAGCCCAATGAAAAAGAGCTTGCTGCCCACTCGGTTGCTAAATATGGCGCCCTTGCCCGCCAGAAAATATCCACTGGTAACTACATCACCACCCTTGGTGAAGTTACACGTGGTCACATGGGCGGCGCCAACACTATGTACGCAATTGATGCACCTCCTGAACTTTAA

>KLP2_P8_b11

ATGGAAAGATCAACTTTGATCAATTTACTTCTGTTACACAAATTTGAACACAAGATTAACACTGAAGGAATCATTGTTGTGCACGGAATTGCTGGAACCGGGAAAACCACATTGCTTAGGACTTTATTTTCTGCATACCCTAGCTTAGTTATAGATTCACCTAGGCCTTGCTACTTAGATAAAGCTAATAAAATTTCACAAGTTTGCCTTTCTTGTTTTCCAAATACCTTGTGTGACATTGTTGACGAGTACCATCTCTTAGAAAGTTTTCCTGAACCAAAACTAGCCATTTTTGGTGACCCCTGTCAGTGCACTTACATTGAAAGGTTGAGAACACCCAACTACACATCCTTCAGAACACACCGATTTGGCAAATCCACTGCTGCTCTACTAAACAAGTTATTTGATCTTAACATTGAGTCAGTCAAAGCACAAGACGACACAGTGGAATACTTTGATCCTTTCGCAGTGGACCCCTCTGAACACATTTCTGCATCAGAAAAAGAAGTTTTGGAATTTGTAGGTGATCAAGTTGAGACTACAAGCTCTGAAGAACTAGCTGGTCTCGAGTTTAGTGAAGTTACTTTCTACTGTACCACACTTGCTGGTGCTGTTCAAGAAAATCCTGCCAAAACTTTCATTTCACTAACTAGACACACTTCAAAGCTCACAATTGGTGAACTAAATGCCAGGTCTGACTCCTAGATGCCAGGTCTGACTCCTAGAGCTGACCTTACTGACACGTATAAAATCATTGCTATAGCCCTTCTACTGTCAGCTTGCATTTACTTCCAAAACAGTCATTATCAACCAGTTGCAGGTGATAACTTGCACAGACTACCCTTCGGTGGCCAGTATCAAGACGGGACTAAGAAGATTTCTTACTTTCCGCAGCAACAATCCTACTTTCACTCAGGAAACAAGCTTAATGTCCTCATACTTATCTTCATTCTTACACTGGGTATTGTCCTCACCAATAAATTTAGTTTTAGCATTAGCCGTAATACTCACCAGCATCATTGCTACAATACACATTCTGCAACCCAAACAGGTCAATCAGTGCCAGGTCATCATTGAATGTCCTCATACTTATCTTCATTCTTACACTGGGTATTGTCCTCACCAATAAATTTAGTTTTAGCATTAGCCGTAATACTCACCAGCATCATTGCTACAATACACATTCTGCAACCCAAACAGGTCAATCAGTGCCAGGTCATCATTGACGGTGCAGCCATAGTTATAACAAATTGTCCAAACACACCCGAAGTTCTTAAAGCAATCAACTTCTCCCCTTGGAACGGGTTAAGTTTTCCTCAATTGTGAATGGAAAACCAACCTACAGCTTCTAACCCATCAGATGTACCACCAACTGCCGCTCAAGCTGGTGCCCAGAGCCCGGCCGACTTCTCAAATCCTAATACAGCTCCTTCCCTAAGTGATTTGAAGAAAATCAAATACGTGTCAACTGTCACTTCAGTTGCCACGCCTACTGAAATTGAGGCCCTTGGCAAGATCTTTACTGCCATGGGTTTAGCAGCCAATGAGACCGGACCTGCCATGTGGGACCTCGCTCGTGCTTATGCTGATGTGCAAAGTTCAAAATCTGCACAACTTATAGGTGCCACACCATCCAACCCTGCTTTGTCTAGACGTGCACTTGCTGCACAGTTTGATCGTATCAATATCACACCCAGACAATTCTGCATGTATTTTGCAAAAATTGTTTGGAACATACTGTTAGACAGCAATGTGCCACCTGCCAACTGGGCAAAATTGGGCTATCAGAAAGATACCAAATTTGCTGCTTTTGACTTCTTTGATGGAGTCACAAATCCAGCTAGTCTACAGCCTGCAGATGGCCTAATCAGGCAGCCCAATGAAAAAGAGCTTGCTGCCCACTCGGTTGCTAAATATGGCGCCCTTGCCCGCCAGAAAATATCCACTGGTAACTACATCACCACCCTTGGTGAAGTTACACGTGGTCACATGGGCGGCGCCAACACTATGTACGCAATTGATGCACCTCCTGAACTTTAA

>KLP2_P8_b10

ATGGAAAGATCAACTTTGATCGATTTACTTCTGTTACACAAATTTGAACACAAGATTAACACTGAAGGAATCATTGTTGTGCACGGAATTGCTGGAACTGGGAAAACCACATTGCTTAGGACTTTATTTTCTGCATACCCTAGCTTAGTTATAGGTTCACCTAGGCCTTGCTACTTAGATAAAGCTAATAAAATTTCACAAGTTTGCCTTTCTTGTTTTCCAAATACCTTGTGTGACATTGTTGACGAGTACCATCTCTTAGAAAGTTTTCCTGAACCAAAACTAGCCATTTTTGGTGACCCCTGTCAGTGCACTTACATTGAAAGGTTGAGAACACCCAACTACACATCCTTCAGAACACACCGATTTGGCAAATCCACTGCTGCTCTACTAAACAAGTTATTTGATCTTAACATTGAGTCAGTCAAAGCACAAGACGACACAGTGGAATACTTTGATCCTTTCGCAGTGGACCCCTCTGAACACATTTCTGCATCAGAAAAAGAAGTTTTGGAATTTGTAGGTGATCAAGTTGAGACTACAAGCTCTGAAGAACTAGCTGGTCTCGAGTTTAGTGAAGTTACTTTCTACTGTACCACACTTGCTGGTGCTGTTCAAGAAAATCCTGCCAAAACTTTCATTTCACTAACTAGACACACTTCAAAGCTCACAATTGGTGAACTAAATGCCAGGTCTGACTCCTAGATGCCAGGTCTGACTCCTAGAGCTGACCTTACTGACACGTATAAAATCATTGCTATAGCCCTTCTACTGTCAGCTTGCATTTACTTCCAAAACAGTCATTATCAACCAGTTGCAGGTGATAACTTGCACAGACTACCCTTCGGTGGCCAGTATCAAGACGGGACTAAGAAGATTTCTTACTTTCCGCAGCAACAATCCTACTTTCACTCAGGAAACAAGCTTAATGTCCTCATACTTATCTTCATTCTTACACTGGGTATTGTCCTCACCAATAAATTTAGTTTTAGCATTAGCCGTAATACTCACCAGCATCATTGCTACAATACACATTCTGCAACCCAAACAGGTCAATCAGTGCCAGGTCATCATTGAATGTCCTCATACTTATCTTCATTCTTACACTGGGTATTGTCCTCACCAATAAATTTAGTTTTAGCATTAGCCGTAATACTCACCAGCATCATTGCTACAATACACATTCTGCAACCCAAACAGGTCAATCAGTGCCAGGTCATCATTGACGGTGCAGCCATAGTTATAACAAATTGTCCAAACACACCCGAAGTTCTTAAAGCAATCAACTTCTCCCCTTGGAACGGGTTAAGTTTTCCTCAATTGTGAATGGAAAACCAACCTACAGCTTCTAACCCATCAGATGTACCACCAACTGCCGCTCAAGCTGGTGCCCAGAGCCCGGCCGACTTCTCAAATCCTAATACAGCTCCTTCCCTAAGTGATTTGAAGAAAATCAAATACGTGTCAACTGTCACTTCAGTTGCCACGCCTGCTGAAATTGAGGCCCTTGGCAAGATCTTTACTGCCATGGGTTTAGCAGCCAATGAGACCGGACCTGCCATGTGGGACCTCGCTCGTGCTTATGCTGATGTGCAAAGTTCAAAATCTGCACAACTTATAGGTGCCACACCATCCAACCCTGCTTTGTCTAGACGTGCACTTGCTGCACAGTTTGATCGTATCAATATCACACCCAGACAATTCTGCATGTATTTTGCAAAAATTGTTTGGAACATACTGTTAGACAGCAATGTGCCACCTGCCAACTGGGCAAAATTGGGCTATCAGAAAGATACCAAATTTGCTGCTTTTGACTTCTTTGATGGAGTCACAAATCCAGCTAGTCTACAGCCTGCAGATGGCCTAATCAGGCAGCCCAATGAAAAAGAGCTTGCTGCCCACTCGGTTGCTAAATATGGCGCCCTTGCCCGCCAGAAAATATCCACTGGTAACTACATCACCACCCTTGGTGAAGTTACACGTGGTCACATGGGCGGCGCCAACACTATGTACGCAATTGATGCACCTCCTGAACTTTAA

>KLP2_P8_b9

ATGGAAAGATCAACTTTGATCAATTTACTTCTGTTACACAAATTTGAACACAAGATTAACACTGAAGGAATCATTGTTGTGCACGGAATTGCTGGAACTGGGAAAACCACATTGCTTAGGACTTTATTTTCTGCATACCCTAGCTTAGTTATAGGTTCACCTAGGCCTTGCTACTTAGATAAAGCTAATAAAATTTCACAAGTTTGCCTTTCTTGTTTTCCAAATACCTTGTGTGACATTGTTGACGAGTACCATCTCTTAGAAAGTTTTCCTGAACCAAAACTAGCCATTTTTGGTGACCCCTGTCAGTGCACTTACATTGAAAGGTTGAGAACACCCAACTACACATCCTTCAGAACACACCGATTTGGCAAATCCACTGCTGCTCTACTAAACAAGTTATTTGATCTTAACATTGAGTCAGTCAAAGCACAAGACGACACAGTGGAATACTTTGATCCTTTCGCAGTGGACCCCTCTGAACACATTTCTGCATCAGAAAAAGAAGTTTTGGAATTTGTAGGTGATCAAGTTGAGACTACAAGCTCTGAAGAACTAGCTGGTCTCGAGTTTAGTGAAGTTACTTTCTACTGTACCACACTTGCTGGTGCTGTTCAAGAAAATCCTGCCAAAACTTTCATTTCACTAACTAGACACACTTCAAAGCTCACAATTGGTGAACTAAATGCCAGGTCTGACTCCTAGATGCCAGGTCTGACTCCTAGAGCTGACCTTACTGACACGTATAAAATCATTGCTATAGCCCTTCTACTGTCAGCTTGCATTTACTTCCAAAACAGTCATTATCAACCAGTTGCAGGTGATAACTTGCACAGACTACCCTTCGGTGGCCAGTATCAAGACGGGACTAAGAAGATTTCTTACTTTCCGCAGCAACAATCCTACTTTCACTCAGGAAACAAGCTTAATGTCCTCATACTTATCTTCATTCTTACACTGGGTATTGTCCTCACCAATAAATTTAGTTTTAGCATTAGCCGTAATACTCACCAGCATCATTGCTACAATACACATTCTGCAACCCAAACAGGTCAATCAGTGCCAGGTCATCATTGAATGTCCTCATACTTATCTTCATTCTTACACTGGGTATTGTCCTCACCAATAAATTTAGTTTTAGCATTAGCCGTAATACTCACCAGCATCATTGCTACAATACACATTCTGCAACCCAAACAGGTCAATCAGTGCCAGGTCATCATTGACGGTGCAGCCATAGTTATAACAAATTGTCCAAACACACCCGAAGTTCTTAAAGCAATCAACTTCTCCCCTTGGAACGGGTTAAGTTTTCCTCAATTGTGAATGGAAAACCAACCTACAGCTTCTAACCCATCAGATGTACCACCAACTGCCGCTCAAGCTGGTGCCCAGAGCCCGGCCGACTTCTCAAATCCTAATACAGCTCCTTCCCTAAGTGATTTGAAGAAAATCAAATACGTGTCAACTGTCACTTCAGTTGCCACGCCTGCTGAAATTGAGGCCCTTGGCAAGATCTTTACTGCCATGGGTTTAGCAGCCAATGAGACCGGACCTGCCATGTGGGACCTCGCTCGTGCTTATGCTGATGTGCAAAGTTCAAAATCTGCACAACTTATAGGTGCCACACCATCCAACCCTGCTTTGTCTAGACGTGCACTTGCTGCACAGTTTGATCGTATCAATATCACACCCAGACAATTCTGCATGTATTTTGCAAAAATTGTTTGGAACATACTGTTAGACAGCAATGTGCCACCTGCCAACTGGGCAAAATTGGGCTATCAGAAAGATACCAAATTTGCTGCTTTTGACTTCTTTGATGGAGTCACAAATCCAGCTAGTCTACAGCCTGCAGATGGCCTAATCAGGCAGCCCAATGAAAAAGAGCTTGCTGCCCACTCGGTTGCTAAATATGGCGCCCTTGCCCGCCAGAAAATATCCACTGGTAACTACATCACCACCCTTGGTGAAGTTACACGTGGTCACATGGGCGGCGCCAACACTATGTACGCAATTGATGCACCTCCTGAACTTTAA

>KLP2_P8_b8

ATGGAAAGATCAACTTTGATCAATTTACTTCTGTTACACAAATTTGAACACAAGATTAACACTGAAGGAATCATTGTTGTGCACGGAATTGCTGGAACTGGGAAAACCACATTGCTTAGGACTTTATTTTCTGCATACCCTAGCTTAGTTATAGGTTCACCCAGGCCTTGCTACTTAGATAAAGCTAATAAAATTTCACAAGTTTGCCTTTCTTGTTTTCCAAATACCTTGTGTGACATTGTTGACGAGTACCATCTCTTAGAAAGTTTTCCTGAACCAAAACTAGCCATTTTTGGTGACCCCTGTCAGTGCACTTACATTGAAAGGTTGAGAACACCCAACTACACATCCTTCAGAACACACCGATTTGGCAAATCCACTGCTGCTCTACTAAACAAGTTATTTGATCTTAACATTGAGTCAGTCAAAGCACAAGACGACACAGTGGAATACTTTGATCCTTTCGCAGTGGACCCCTCTGAACACATTTCTGCATCAGAAAAAGAAGTTTTGGAATTTGTAGGTGATCAAGTTGAGACTACAAGCTCTGAAGAACTAGCTGGTCTCGAGTTTAGTGAAGTTACTTTCTACTGTACCACACTTGCTGGTGCTGTTCAAGAAAATCCTGCCAAAACTTTCATTTCACTAACTAGACACACTTCAAAGCTCACAATTGGTGAACTAAATGCCAGGTCTGACTCCTAGATGCCAGGTCTGACTCCTAGAGCTGACCTTACTGACACGTATAAAATCATTGCTATAGCCCTTCTACTGTCAGCTTGCATTTACTTCCAAAACAGTCATTATCAACCAGTTGCAGGTGATAACTTGCACAGACTACCCTTCGGTGGCCAGTATCAAGACGGGACTAAGAAGATTTCTTACTTTCCGCAGCAACAATCCTACTTTCACTCAGGAAACAAGCTTAATGTCCTCATACTTATCTTCATTCTTACACTGGGTATTGTCCTCACCAATAAATTTAGTTTTAGCATTAGCCGTAATACTCACCAGCATCATTGCTACAATACACATTCTGCAACCCAAACAGGTCAATCAGTGCCAGGTCATCATTGAATGTCCTCATACTTATCTTCATTCTTACACTGGGTATTGTCCTCACCAATAAATTTAGTTTTAGCATTAGCCGTAATACTCACCAGCATCATTGCTACAATACACATTCTGCAACCCAAACAGGTCAATCAGTGCCAGGTCATCATTGACGGTGCAGCCATAGTTATAACAAATTGTCCAAACACACCCGAAGTTCTTAAAGCAATCAACTTCTCCCCTTGGAACGGGTTAAGTTTTCCTCAATTGTGAATGGAAAACCAACCTACAGCTTCTAACCCATCAGATGTACCACCAACTGCCGCTCAAGCTGGTGCCCAGAGCCCGGCCGACTTCTCAAATCCTAATACAGCTCCTTCCCTAAGTGATTTGAAGAAAATCAAATACGTGTCAACTGTCACTTCAGTTGCCACGCCTGCTGAAATTGAGGCCCTTGGCAAGATCTTTACTGCCATGGGTTTAGCAGCCAATGAGACCGGACCTGCCATGTGGGACCTCGCTCGTGCTTATGCTGATGTGCAAAGTTCAAAATCTGCACAACTTATAGGTGCCACACCATCCAACCCTGCTTTGTCTAGACGTGCACTTGCTGCACAGTTTGATCGTATCAATATCACACCCAGACAATTCTGCATGTATTTTGCAAAAATTGTTTGGAACATACTGTTAGACAGCAATGTGCCACCTGCCAACTGGGCAAAATTGGGCTATCAGAAAGATACCAAATTTGCTGCTTTTGACTTCTTTGATGGAGTCACAAATCCAGCTAGTCTACAGCCTGCAGATGGCCTAATCAGGCAGCCCAATGAAAAAGAGCTTGCTGCCCACTCGGTTGCTAAATATGGCGCCCTTGCCCGCCAGAAAATATCCACTGGTAACTACATCACCACCCTTGGTGAAGTTACACGTGGTCACATGGGCGGCGCCAACACTATGTACGCAATTGATGCACCTCCTGAACTTTAA

>KLP2_P8_b7

ATGGAAAGATCAACTTTGATCAATTTACTTCTGTTACACAAATTTGAACACAAGATTAACACTGAAGGAATCATTGTTGTGCACGGAATTGCTGGAACTGGGAAAACCACATTGCTTAGGACTTTATTTTCTGCATACCCTAGCTTAGTTATAGGTTCGCCTAGGCCTTGCTACTTAGATAAAGCTAATAAAATTTCACAAGTTTGCCTTTCTTGTTTTCCAAATACCTTGTGTGACATTGTTGACGAGTACCATCTCTTAGAAAGTTTTCCTGAACCAAAACTAGCCATTTTTGGTGACCCCTGTCAGTGCACTTACATTGAAAGGTTGAGAACACCCAACTACACATCCTTCAGAACACACCGATTTGGCAAATCCACTGCTGCTCTACTAAACAAGTTATTTGATCTTAACATTGAGTCAGTCAAAGCACAAGACGACACAGTGGAATACTTTGATCCTTTCGCAGTGGACCCCTCTGAACACATTTCTGCATCAGAAAAAGAAGTTTTGGAATTTGTAGGTGATCAAGTTGAGACTACAAGCTCTGAAGAACTAGCTGGTCTCGAGTTTAGTGAAGTTACTTTCTACTGTACCACACTTGCTGGTGCTGTTCAAGAAAATCCTGCCAAAACTTTCATTTCACTAACTAGACACACTTCAAAGCTCACAATTGGTGAACTAAATGCCAGGTCTGACTCCTAGATGCCAGGTCTGACTCCTAGAGCTGACCTTACTGACACGTATAAAATCATTGCTATAGCCCTTCTACTGTCAGCTTGCATTTACTTCCAAAACAGTCATTATCAACCAGTTGCAGGTGATAACTTGCACAGACTACCCTTCGGTGGCCAGTATCAAGACGGGACTAAGAAGATTTCTTACTTTCCGCAGCAACAATCCTACTTTCACTCAGGAAACAAGCTTAATGTCCTCATACTTATCTTCATTCTTACACTGGGTATTGTCCTCGCCAATAAATTTAGTTTTAGCATTAGCCGTAATACTCACCAGCATCATTGCTACAATACACATTCTGCAACCCAAACAGGTCAATCAGTGCCAGGTCATCATTGAATGTCCTCATACTTATCTTCATTCTTACACTGGGTATTGTCCTCGCCAATAAATTTAGTTTTAGCATTAGCCGTAATACTCACCAGCATCATTGCTACAATACACATTCTGCAACCCAAACAGGTCAATCAGTGCCAGGTCATCATTGACGGTGCAGCCATAGTTATAACAAATTGTCCAAACACACCCGAAGTTCTTAAAGCAATCAACTTCTCCCCTTGGAACGGGTTAAGTTTTCCTCAATTGTGAATGGAAAACCAACCTACAGCTTCTAACCCATCAGATGTACCACCAACTGCCGCTCAAGCTGGTGCCCAGAGCCCGGCCGACTTCTCAAATCCTAATACAGCTCCTTCCCTAAGTGATTTGAAGAAAATCAAATACGTGTCAACTGTCACTTCAGTTGCCACGCCTGCTGAAATTGAGGCCCTTGGCAAGATCTTTACTGCCATGGGTTTAGCAGCCAATGAGACCGGACCTGCCATGTGGGACCTCGCTCGTGCTTATGCTGATGTGCAAAGTTCAAAATCTGCACAACTTATAGGTGCCACACCATCCAACCCTGCTTTGTCTAGACGTGCACTTGCTGCACAGTTTGATCGTATCAATATCACACCCAGACAATTCTGCATGTATTTTGCAAAAATTGTTTGGAACATACTGTTAGACAGCAATGTGCCACCTGCCAACTGGGCAAAATTGGGCTATCAGAAAGATACCAAATTTGCTGCTTTTGACTTCTTTGATGGAGTCACAAATCCAGCTAGTCTACAGCCTGCAGATGGCCTAATCAGGCAGCCCAATGAAAAAGAGCTTGCTGCCCACTCGGTTGCTAAATATGGCGCCCTTGCCCGCCAGAAAATATCCACTGGTAACTACATCACCACCCTTGGTGAAGTTACACGTGGTCACATGGGCGGCGCCAACACTATGTACGCAATTGATGCACCTCCTGAACTTTAA

>KLP2_P8_b6

ATGGAAAGATCAACTTTGATCAATTTACTTCTGTTACACAAATTTGAACACAAGATTAACACTGAAGGAATCATTGTTGTGCACGGAATTGCTGGAACTGGGAAAACCACATTGCTTAGGACTTTATTTTCTGCATACCCTAGCTTAGTTATAGGTTCACCTAGGCCTTGCTACTTAGATAAAGCTAATAAAATTTCACAAGTTTGCCTTTCTTGTTTTCCAAATACCTTGTGTGACATTGTTGACGAGTACCATCTCTTAGAAAGTTTTCCTGAACCAAAACTAGCCATTTTTGGTGACCCCTGTCAGTGCACTTACATTGAAAGGTTGAGAACACCCAACTACACATCCTTCAGAACACACCGATTTGGCAAATCCACTGCTGCTCTACTAAACAAGTTATTTGATCTTAACATTGAGTCAGTCAAAGCACAAGACGACACAGTGGAATACTTTGATCCTTTCGCAGTGGACCCCTCTGAACACATTTCTGCATCAGAAAAAGAAGTTTTGGAATTTGTAGGTGATCAAGTTGAGACTACAAGCTCTGAAGAACTAGCTGGTCTCGAGTTTAGTGAAGTTACTTTCTACTGTACCACACTTGCTGGTGCTGTTCAAGAAAATCCTGCCAAAACTTTCATTTCACTAACTAGACACACTTCAAAGCTCACAATTGGTGAACTAAATGCCAGGTCTGACTCCTAGATGCCAGGTCTGACTCCTAGAGCTGACCTTACTGACACGTATAAAATCATTGCTATAGCCCTTCTACTGTCAGCTTGCATTTACTTCCAAAACAGTCATTATCAACCAGTTGCAGGTGATAACTTGCACAGACTACCCTTCGGTGGCCAGTATCAAGACGGGACTAAGAAGATTTCTTACTTTCCGCAGCAACAATCCTACTTTCACTCAGGAAACAAGCTTAATGTCCTCATACTTATCTTCATTCTTACACTGGGTATTGTCCTCACCAATAAATTTAGTTTTAGCATTAGCCGTAATACTCACCAGCATCATTGCTACAATACACATTCTGCAACCCAAACAGGTCAATCAGTGCCAGGTCATCATTGAATGTCCTCATACTTATCTTCATTCTTACACTGGGTATTGTCCTCACCAATAAATTTAGTTTTAGCATTAGCCGTAATACTCACCAGCATCATTGCTACAATACACATTCTGCAACCCAAACAGGTCAATCAGTGCCAGGTCATCATTGACGGTGCAGCCATAGTTATAACAAATTGTCCAAACACACCCGAAGTTCTTAAAGCAATCAACTTCTCCCCTTGGAACGGGTTAAGTTTTCCTCAATTGTGAATGGAAAACCAACCTACAGCTTCTAACCCATCAGATGTACCACCAACTGCCGCTCAAGCTGGTGCCCAGAGCCCGGCCGACTTCTCAAATCCTAATACAGCTCCTTCCCTAAGTGATTTGAAGAAAATCAAATACGTGTCAACTGTCACTTCAGTTGCCACGCCTGCTGAAATTGAGGCCCTTGGCAAGATCTTTACTGCCATGGGTTTAGCAGCCAATGAGACCGGACCTGCCATGTGGGACCTCGCTCGTGCTTATGCTGATGTGCAAAGTTCAAAATCTGCACAACTTATAGGTGCCACACCATCCAACCCTGCTTTGTCTAGACGTGCACTTGCTGCACAGTTTGATCGTATCAATATCACACCCAGACAATTCTGCATGTATTTTGCAAAAATTGTTTGGAACATACTGTTAGACAGCAATGTGCCACCTGCCAACTGGGCAAAATTGGGCTATCAGAAAGATACCAAATTTGCTGCTTTTGACTTCTTTGATGGAGTCACAAATCCAGCTAGTCTACAGCCTGCAGATGGCCTAATCAGGCAGCCCAATGAAAAAGAGCTTGCTGCCCACTCGGTTGCTAAATATGGCGCCCTTGCCCGCCAGAAAATATCCACTGGTAACTACATCACCACCCTTGGTGAAGTTACACGTGGTCACATGGGCGGCGCCAACACTATGTACGCAATTGATGCACCTCCTGAACTTTAA

>KLP2_P8_b5

ATGGAAAGATCAACTTTGATCAATTTACTTCTGTTACACAAATTTGAACACAAGATTAACACTGAAGGAATCATTGTTGTGCACGGAATTGCTGGAACTGGGAAAACCACATTGCTTAGGACTCTATTTTCTGCATACCCTAGCTTAGTTATAGGTTCGCCTAGGCCTTGCTACTTAGATAAAGCTAATAAAATTTCACAAGTTTGCCTTTCTTGTTTTCCAAATACCTTGTGTGACATTGTTGACGAGTACCATCTCTTAGAAAGTTTTCCTGAACCAAAACTAGCCATTTTTGGTGACCCCTGTCAGTGCACTTACATTGAAAGGTTGAGAACACCCAACTACACATCCTTCAGAACACACCGATTTGGCAAATCCACTGCTGCTCTACTAAACAAGTTATTTGATCTTAACATTGAGTCAGTCAAAGCACAAGACGACACAGTGGAATACTTTGATCCTTTCGCAGTGGACCCCTCTGAACACATTTCTGCATCAGAAAAAGAAGTTTTGGAATTTGTAGGTGATCAAGTTGAGACTACAAGCTCTGAAGAATTAGCTGGTCTCGAGTTTAGTGAAGTTACTTTCTACTGTACCACACTTGCTGGTGCTGTTCAAGAAAATCCTGCCAAAACTTTCATTTCACTAACTAGACACACTTCAAAGCTCACAATTGGTGAACTAAATGCCAGGTCTGACTCCTAGATGCCAGGTCTGACTCCTAGAGCTGACCTTACTGACACGTATAAAATCATTGCTATAGCCCTTCTACTGTCAGCTTGCATTTACTTCCAAAACAGTCATTATCAACCAGTTGCAGGTGATAACTTGCACAGACTACCCTTCGGTGGCCAGTATCAAGACGGGACTAAGAAGATTTCTTACTTTCCGCAGCAACAATCCTACTTTCACTCAGGAAACAAGCTTAATGTCCTCATACTTATCTTCATTCTTACACTGGGTATTGTCCTCACCAATAAATTTAGTTTTAGCATTAGCCGTAATACTCACCAGCATCATTGCTACAATACACATTCTGCAACCCAAACAGGTCAATCAGTGCCAGGTCATCATTGAATGTCCTCATACTTATCTTCATTCTTACACTGGGTATTGTCCTCACCAATAAATTTAGTTTTAGCATTAGCCGTAATACTCACCAGCATCATTGCTACAATACACATTCTGCAACCCAAACAGGTCAATCAGTGCCAGGTCATCATTGACGGTGCAGCCATAGTTATAACAAATTGTCCAAACACACCCGAAGTTCTTAAAGCAATCAACTTCTCCCCTTGGAACGGGTTAAGTTTTCCTCAATTGTGAATGGAAAACCAACCTACAGCTTCTAACCCATCAGATGTACCACCAACTGCCGCTCAAGCTGGTGCCCAGAGCCCGGCCGACTTCTCAAATCCTAATACAGCTCCTTCCCTAAGTGATTTGAAGAAAATCAAATACGTGTCAACTGTCACTTCAGTTGCCACGCCTGCTGAAATTGGGGCCCTTGGCAAGATCTTTACTGCCATGGGTTTAGCAGCCAATGAGACCGGACCTGCCATGTGGGACCTCGCTCGTGCCTATGCTGATGTGCAAAGTTCAAAATCTGCACAACTTATAGGTGCCACACCATCCAACCCTGCTTTGTCTAGACGTGCACTTGCTGCACAGTTTGATCGTATCAATATCACACCCAGACAATTCTGCATGTATTTTGCAAAAATTGTTTGGAACATACTGTTAGACAGCAATGTGCCACCTGCCAACTGGGCAAAATTGGGCTATCAGAAAGATACCAAATTTGCTGCTTTTGACTTCTTTGATGGAGTCACAAATCCAGCTAGTCTACAGCCTGCAGATGGCCTAATCAGGCAGCCCAATGAAAAAGAGCTTGCTGCCCACTCGGTTGCTAAATATGGCGCCCTTGCCCGCCAGAAAATATCCACTGGTAACTACATCACCACCCTTGGTGAAGTTACACGTGGTCACATGGGCGGCGCCAACACTATGTACGCAATTGATGCACCTCCTGAACTTTAA

>KLP2_P8_b4

ATGGAAAGATCAACTTTGATCAATTTACTTCTGTTACACAAATTTGAACACAAGATTAACACTGAAGGAATCATTGTTGTGCACGGAATTGCTGGAACTGGGAAAACCACATTGCTTAGGACTTTATTTTCTGCATACCCTAGCTTAGTTATAGGTTCACCTAGGCCTTGCTACTTAGATAAAGCTAATAAAATTTCACAAGTTTGCCTTTCTTGTTTTCCAAATACCTTGTGTGACATTGTTGACGAGTACCATCTCTTAGAAAGTTTTCCTGAACCAAAACTAGCCATTTTTGGTGACCCCTGTCAGTGCACTTACATTGAAAGGTTGAGAACACCCAACTACACATCCTTCAGAACACACCGATTTGGCAAATCCACTGCTGCTCTACTAAACAAGTTATTTGATCTTAACATTGAGTCAGTCAAAGCACAAGACGACACAGTGGAATACTTTGATCCTTTCGCAGTGGACCCCTCTGAACACATTTCTGCATCAGAAAAAGAAGTTTTGGAATTTGTAGGTGATCAAGTTGAGACTACAAGCTCTGAAGAACTAGCTGGTCTCGAGTTTAGTGAAGTTACTTTCTACTGTACCACACTTGCTGGTGCTGTTCAAGAAAATCCTGCCAAAACTTTCATTTCACTAACTAGACACACTTCAAAGCTCACAATTGGTGAACTAAATGCCAGGTCTGACTCCTAGATGCCAGGTCTGACTCCTAGAGCTGACCTTACTGACACGTATAAAATCATTGCTATAGCCCTTCTACTGTCAGCTTGCATTTACTTCCAAAACAGTCATTATCAACCAGTTGCAGGTGATAACTTGCACAGACTACCCTTCGGTGGCCAGTATCAAGACGGGACTAAGAAGATTTCTTACTTTCCGCAGCAACAATCCTACTTTCACTCAGGAAACAAGCTTAATGTCCTCATACTTATCTTCATTCTTACACTGGGTATTGTCCTCACCAATAAATTTAGTTTTAGCATTAGCCGTAATACTCACCAGCATCATTGCTACAATACACATTCTGCAACCCAAACAGGTCAATCAGTGCCAGGTCATCATTGAATGTCCTCATACTTATCTTCATTCTTACACTGGGTATTGTCCTCACCAATAAATTTAGTTTTAGCATTAGCCGTAATACTCACCAGCATCATTGCTACAATACACATTCTGCAACCCAAACAGGTCAATCAGTGCCAGGTCATCATTGACGGTGCAGCCATAGTTATAACAAATTGTCCAAACACACCCGAAGTTCTTAAAGCAATCAACTTCTCCCCTTGGAACGGGTTAAGTTTTCCTCAATTGTGAATGGAAAACCAACCTACAGCTTCTAACCCATCAGATGTACCACCAACTGCCGCTCAAGCTGGTGCCCAGAGCCCGGCCGACTTCTCAAATCCTAATACAGCTCCTTCCCTAAGTGATTTGAAGAAAATCAAATACGTGTCAACTGTCACTTCAGTTGCCACGCCTGCTGAAATTGAGGCCCTTGGCAAGATCTTTACTGCCATGGGTTTAGCAGCCAATGAGACCGGACCTACCATGTGGGACCTCGCTCGTGCTTATGCTGATGTGCAAAGTTCAAAATCTGCACAACTTATAGGTGCCACACCATCCAACCCTGCTTTGTCTAGACGTGCACTTGCTGCACAGTTTGATCGTATCAATATCACACCCAGACAATTCTGCATGTATTTTGCAAAAATTGTTTGGAACATACTGTTAGACAGCAATGTGCCACCTGCCAACTGGGCAAAATTGGGCTATCAGAAAGATACCAAATTTGCTGCTTTTGACTTCTTTGATGGAGTCACAAATCCAGCTAGTCTACAGCCTGCAGATGGCCTAATCAGGCAGCCCAATGAAAAAGAGCTTGCTGCCCACTCGGTTGCTAAATATGGCGCCCTTGCCCGCCAGAAAATATCCACTGGTAACTACATCACCACCCTTGGTGAAGTTACACGTGGTCACATGGGCGGCGCCAACACTATGTACGCAATTGATGCACCTCCTGAACTTTAA

>KLP2_P8_b3

ATGGAAAGATCAACTTTGATCAATTTACTTCTGTTACACAAATTTGAACACAAGATTAACACTGAAGGAATCATTGTTGTGCACGGAATTGCTGGAACTGGGAAAACCACATTGCTTAGGACTTTATTTTCTGCATACCCTAGCTTAGTTATAGGTTCACCTAGGCCTTGCTACTTAGATAAAGCTAATAAAATTTCACAAGTTTGCCTTTCTTGTTTTCCAAATACCTTGTGTGACATTGTTGACGAGTACCATCTCTTAGAAAGTTTTCCTGAACCAAAACTAGCCATTTTTGGTGACCCCTGTCAGTGCACTTACATTGAAAGGTTGAGAACACCCAACTACACATCCTTCAGAACACACCGATTTGGCAAATCCACTGCTGCTCTACTAAACAAGTTATTTGATCTTAACATTGAGTCAGTCAAAGCACAAGACGACACAGTGGAATACTTTGATCCTTTCGCAGTGGACCCCTCTGAACACATTTCTGCATCAGAAAAAGAAGTTTTGGAATTTGTAGGTGATCAAGTTGAGACTACAAGCTCTGAAGAACTAGCTGGTCTCGAGTTTAGTGAAGTTACTTTCTACTGTACCACACTTGCTGGTGCTGTTCAAGAAAATCCTGCCAAAACTTTCATTTCACTAACTAGACACACTTCAAAGCTCACAATTGGTGAACTAAATGCCAGGTCTGACTCCTAGATGCCAGGTCTGACTCCTAGAGCTGACCTTACTGACACGTATAAAATCATTGCTATAGCCCTTCTACTGTCAGCTTGCATTTACTTCCAAAACAGTCATTATCAACCAGTTGCAGGTGATAACTTGCACAGACTACCCTTCGGTGGCCAGTATCAAGACGGGACTAAGAAGATTTCTTACTTTCCGCAGCAACAATCCTACTTTCACTCAGGAAACAAGCTTAATGTCCTCATACTTATCTTCATTCTTACACTGGGTATTGTCCTCACCAATAAATTTAGTTTTAGCATTAGCCGTAATACTCACCAGCATCATTGCTACAATACACATTCTGCAACCCAAACAGGTCAATCAGTGCCAGGTCATCATTGAATGTCCTCATACTTATCTTCATTCTTACACTGGGTATTGTCCTCACCAATAAATTTAGTTTTAGCATTAGCCGTAATACTCACCAGCATCATTGCTACAATACACATTCTGCAACCCAAACAGGTCAATCAGTGCCAGGTCATCATTGACGGTGCAGCCATAGTTATAACAAATTGTCCAAACACACCCGAAGTTCTTAAAGCAATCAACTTCTCCCCTTGGAACGGGTTAAGTTTTCCTCAATTGTGAATGGAAAACCAACCTACAGCTTCTAACCCATCAGATGTACCACCAACTGCCGCTCAAGCTGGTGCCCAGAGCCCGGCCGACTTCTCAAATCCTAATACAGCTCCTTCCCTAAGTGATTTGAAGAAAATCAAATACGTGTCAACTGTCACCTCAGTTGCCACGCCTGCTGAAATTGAGGCCCTTGGCAAGATCTTTACTGCCATGGGTTTAGCAGCCAATGAGACCGGACCTGCCATGTGGGACCTCGCTCGTGCTTATGCTGATGTGCAAAGTTCAAAATCTGCACAACTTATAGGTGCCACACCATCCAACCCTGCTTTGTCTAGACGTGCACTTGCTGCACAGTTTGATCGTATCAATATCACACCCAGACAATTCTGCATGTATTTTGCAAAAATTGTTTGGAACATACTGTTAGACAGCAATGTGCCACCTGCCAACTGGGCAAAATTGGGCTATCAGAAAGATACCAAATTTGCTGCTTTTGACTTCTTTGATGGAGTCACAAATCCAGCTAGTCTACAGCCTGCAGATGGCCTAATCAGGCAGCCCAATGAAAAAGAGCTTGCTGCCCACTCGGTTGCTAAATATGGCGCCCTTGCCCGCCAGAAAATATCCACTGGTAACTACATCACCACCCTTGGTGAAGTTACACGTGGTCACATGGGCGGCGCCAACACTATGTACGCAATTGATGCACCTCCTGAACTTTAA

>KLP2_P8_b2

ATGGAAAGATCAACTTTGATCAATTTACTTCTGTTACACAAATTTGAACACAAGATTAACACTGAAGGAATCATTGTTGTGCACGGAATTGCTGGAACTGGGAAAACCACATTGCTTAGGACTTTATTTTCTGCATACCCTAGCTTAGTTATAGGTTCACCTAGGCCTTGCTACTTAGATAAAGCTAATAAAATTTCACAAGTTTGCCTTTCTTGTTTTCCAAATACCTTGTGTGACATTGTTGACGAGTACCATCTCTTAGAAAGTTTTCCTGAACCAAAACTAGCCATTTTTGGTGACCCCTGTCAGTGCACTTACATTGAAAGGTTGAGAACACCCAACTACACATCCTTCAGAACACACCGATTTGGCAAATCCACTGCTGCTCTACTAAACAAGTTATTTGATCTTAACATTGAGTCAGTCAAAGCACAAGACGACACAGTGGAATACTTTGATCCTTTCGCAGTGGACCCCTCTGAACACATTTCTGCATCAGAAAAAGAAGTTTTGGAATTTGTAGGTGATCAAGTTGAGACTACAAGCTCTGAAGAACTAGCTGGTCTCGAGTTTAGTGAAGTTACTTTCTACTGTACCACACTTGCTGGTGCTGTTCAAGAAAATCCTGCCAAAACTTTCATTTCACTAACTAGACACACTTCAAAGCTCACAATTGGTGAACTAAATGCCAGGTCTGACTCCTAGATGCCAGGTCTGACTCCTAGAGCTGACCTTACTGACACGTATAAAATCATTGCTATAGCCCTTCTACTGTCAGCTTGCATTTACTTCCAAAACAGTCATTATCAACCAGTTGCAGGTGATAACTTGCACAGACTACCCTTCGGTGGCCAGTATCAAGACGGGACTAAGAAGATTTCTTACTTTCCGCAGCAACAATCCTACTTTCACTCAGGAAACAAGCTTAATGTCCTCATACTTATCTTCATTCTTACACTGGGTATTGTCCTCACCAATAAATTTAGTTTTAGCATTAGCCGTAATACTCACCAGCATCATTGCTACAATACACATTCTGCAACCCAAACAGGTCAATCAGTGCCAGGTCATCATTGAATGTCCTCATACTTATCTTCATTCTTACACTGGGTATTGTCCTCACCAATAAATTTAGTTTTAGCATTAGCCGTAATACTCACCAGCATCATTGCTACAATACACATTCTGCAACCCAAACAGGTCAATCAGTGCCAGGTCATCATTGACGGTGCAGCCATAGTTATAACAAATTGTCCAAACACACCCGAAGTTCTTAAAGCAATCAACTTCTCCCCTTGGAACGGGTTAAGTTTTCCTCAATTGTGAATGGAAAACCAACCTACAGCTTCTAACCCATCAGATGTACCACCAACTGCCGCTCAAGCTGGTGCCCAGAGCCCGGCCGACTTCTCAAATCCTAATACAGCTCCTTCCCTAAGTGATTTGAAGAAAATCAAATACGTGTCAACTGTCACTTCAGTTGCCACGCCTGCTGAAATTGAGGCCCTTGGCAAGATCTTTACTGCCATGGGTTTAGCAGCCAATGAGACCGGACCTGCCATGTGGGACCTCGCTCGTGCTTATGCTGATGTGCAAAGTTCAAAATCTGCACAACTTATAGGTGCCACACCATCCAACCCTGCTTTGTCTAGACGTGCACTTGCTGCACAGTTTGATCGTATCAATATCACACCCAGACAATTCTGCATGTATTTTGCAAAAATTGTTTGGAACATACTGTTAGACAGCAATGTGCCACCTGCCAACTGGGCAAAATTGGGCTATCAGAAAGATACCAAATTTGCTGCTTTTGACTTCTTTGATGGAGTCACAAATCCAGCTAGTCTACAGCCTGCAGATGGCCTAATCAGGCAGCCCAATGAAAAAGAGCTTGCTGCCCACTCGGTTGCTAAATATGGCGCCCTTGCCCGCCAGAAAATATCCACTGGTAACTACATCACCACCCTTGGTGAAGTTACACGTGGTCACATGGGCGGCGCCAACACTATGTGCGCAATTGATGCACCTCCTGAACTTTAA

>KLP2_P8_b1

ATGGAAAGATCAACTTTGATCAATTTACTTCTGTTACACAAATTTGAACACAAGATTAACACTGAAGGAATCATTGTTGTGCACGGAATTGCTGGAACTGGGAAAACCACATTGCTTAGGACTTTATTTTCTGCATACCCTAGCTTAGTTATAGGTTCACCTAGGCCTTGCTACTTAGATAAAGCTAATAAAATTTCACAAGTTTGCCTTTCTTGTTTTCCAAATACCTTGTGTGACATTGTTGACGAGTACCATCTCTTAGAAAGTTTTCCTGAACCAAAACTAGCCATTTTTGGTGACCCCTGTCAGTGCACTTACATTGAAAGGTTGAGAACACCCAACTACACATCCTTCAGAACACACCGATTTGGCAAATCCACTGCTGCTCTACTAAACAAGTTATTTGATCTTAACATTGAGTCAGTCAAAGCACAAGACGACACAGTGGAATACTTTGATCCTTTCGCAGTGGACCCCTCTGAACACATTTCTGCATCAGAAAAAGAAGTTTTGGAATTTGTAGGTGATCAAGTTGAGACTACAAGCTCTGAAGAACTAGCTGGTCTCGAGTTTAGTGAAGTTACTTTCTACTGTACCACACTTGCTGGTGCTGTTCAAGAAAATCCTGCCAAAACTTTCATTTCACTAACTAGACACACTTCAAAGCTCACAATTGGTGAACTAAATGCCAGGTCTGACTCCTAGATGCCAGGTCTGACTCCTAGAGCTGACCTTACTGACACGTATAAAATCATTGCTATAGCCCTTCTACTGTCAGCTTGCATTTACTTCCAAAACAGTCATTATCAACCAGTTGCAGGTGATAACTTGTACAGACTACCCTTCGGTGGCCAGTATCAAGACGGGACTAAGAAGATTTCTTACTTTCCGCAGCAACAATCCTACTTTCACTCAGGAAACAAGCTTAATGTCCTCATACTTATCTTCATTCTTACACTGGGTATTGTCCTCACCAATAAATTTAGTTTTAGCATTAGCCGTAATACTCACCAGCATCATTGCTACAATACACATTCTGCAACCCAAACAGGTCAATCAGTGCCAGGTCATCATTGAATGTCCTCATACTTATCTTCATTCTTACACTGGGTATTGTCCTCACCAATAAATTTAGTTTTAGCATTAGCCGTAATACTCACCAGCATCATTGCTACAATACACATTCTGCAACCCAAACAGGTCAATCAGTGCCAGGTCATCATTGACGGTGCAGCCGTAGTTATAACAAATTGTCCAAACACACCCGAAGTTCTTAAAGCAATCAACTTCTCCCCTTGGAACGGGTTAAGTTTTCCTCAATTGTGAATGGAAAACCAACCTACAGCTTCTAACCCATCAGATGTACCACCAACTGCCGCTCAAGCTGGTGCCCAGAGCCCGGCCGACTTCTCAAATCCTAATACAGCTCCTTCCCTAAGTGATTTGAAGAAAATCAAATACGTGCCAACTGTCACTTCAGTTGCCACGCCTGCTGAAATTGAGGCCCTTGGCAAGATCTTTACTGCCATGGGTTTAGCAGCCAATGAGACCGGACCTGCCATGTGGGACCTCGCTCGTGCTTATGCTGATGTGCAAAGTTCAAAATCTGCACAACTTATAGGTGCCACACCATCCAACCCTGCTTTGTCTAGACGTGCACTTGCTGCACAGTTTGATCGTATCAATATCACACCCAGACAATTCTGCATGTATTTTGCAAAAATTGTTTGGAACATACTGTTAGACAGCAATGTGCCACCTGCCAACTGGGCAAAATTGGGCTATCAGAAAGATACCAAATTTGCTGCTTTTGACTTCTTTGATGGAGTCACAAATCCAGCTAGTCTACAGCCTGCAGATGGCCTAATCAGGCAGCCCAATGAAAAAGAGCTTGCTGCCCACTCGGTTGCTAAATATGGCGCCCTTGCCCGCCAGAAAATATCCACTGGTAACTACATCACCACCCTTGGTGAAGTTACACGTGGTCACATGGGCGGCGCCAACACTATGTACGCAATTGATGCACCTCCTGAACTTTAA

>KLP2_P8_a14

ATGGAAAGATCAACTTTGATCAATTTACTTCTGTTACACAAATTTGAACACAAGATTAACACTGAAGGAATCATTGTTGTGCACGGAATTGCTGGAACTGGGAAAACCACATTGCTTAGGACTTTATTTTCTGCATACCCTAGCTTAGTTATAGGTTCACCTAGGCCTTGCTACTTAGATAAAGCTAATAAAATTTCACAAGTTTGCCTTTCTTGTTTTCCAAATACCTTGTGTGACATTGTTGACGAGTACCATCTCTTAGAAAGTTTTCCTGAACCAAAACTAGCCATTTTTGGTGACCCCTGTCAGTGCACTTACATTGAAAGGTTGAGAACACCCAACTACACATCCTTCAGAACACACCGATTTGGCAAATCCACTGCTGCTCTACTAAACAAGTTATTTGATCTTAACATTGAGTCAGTCAAAGCACAAGACGACACAGTGGAATACTTTGATCCTTTCGCAGTGGACCCCTCTGAACACATTTCTGCATCAGAAAAAGAAGTTTTGGAATTTGTAGGTGATCAAGTTGAGACTACAAGCTCTGAAGAACTAGCTGGTCTCGAGTTTAGTGAAGTTACTTTCTACTGTACCACACTTGCTGGTGCTGTTCAAGAAAATCCTGCCAAAACTTTCATTTCACTAACTAGACACACTTCAAAGCTCACAATTGGTGAACTAAATGCCAGGTCTGACTCCTAGATGCCAGGTCTGACTCCTAGAGCTGGCCTTACTGACACGTATAAAATCATTGCTATAGCCCTTCTACTGTCAGCTTGCATTTACTTCCAAAACAGTCATTATCAACCAGTTGCAGGTGATAACTTGCACAGACTACCCTTCGGTGGCCAGTATCAAGACGGAACTAAGAAGATTTCTTACTTTCCGCAGCAACAATCCTACTTTCACTCAGGAAACAAGCTTAATGTCCTCATACTTATCTTCATTCTTACACTGGGTATTGTCCTCACCAATAAATTTAGTTTTAGCATTAGCCGTAATACTCACCAGCATCATTGCTACAATACACATTCTGCAACCCAAACAGGTCAATCAGTGCCAGGTCATCATTGAATGTCCTCATACTTATCTTCATTCTTACACTGGGTATTGTCCTCACCAATAAATTTAGTTTTAGCATTAGCCGTAATACTCACCAGCATCATTGCTACAATACACATTCTGCAACCCAAACAGGTCAATCAGTGCCAGGTCATCATTGACGGTGCAGCCATAGTTATAACAAATTGTCCAAACACACCCGAAGTTCTTAAAGCAATCAACTTCTCCCCTTGGAACGGGTTAAGTTTTCCTCAATTGTGAATGGAAAACCAACCTACAGCTTCTAACCCATCAGATGTACCACCAACTGCCGCTCAAGCTGGTGCCCAGAGCCCGGCCGACTTCTCAAATCCTAATACAGCTCCTTCCCTAAGTGATTTGAAGAAAATCAAATACGTGTCAACTGTCACTTCAGTTGCCACGCCTGCTGAAATTGAGGCCCTTGGCAAGATCTTTACTGCCATGGGTTTAGCAGCCAACGAGACCGGACCTGCCATGTGGGACCTCGCTCGTGCTTATGCTGATGTGCAAAGTTCAAAATCTGCACAACTTATAGGTGCCACACCATCCAACCCTGCTTTGTCTAGACGTGCACTTGCTGCACAGTTTGATCGTATCAATATCACACCCAGACAATTCTGCATGTATTTTGCAAAAATTGTTTGGAACATACTGTTAGACAGCAATGTGCCACCTGCCAACTGGGCAAAATTGGGCTATCAGAAAGATACCAAATTTGCTGCTTTTGACTTCTTTGATGGAGTCACAAATCCAGCTAGTCTACAGCCTGCAGATGGCCTAATCAGGCAGCCCAATGAAAAAGAGCTTGCTGCCCACTCGGTTGCTAAATATGGCGCCCTTGCCCGCCAGAAAATATCCACTGGTAACTACATCACCACCCTTGGTGAAGTTACACGTGGTCACATGGGCGGCGCCAACACTATGTACGCAATTGATGCACCTCCTGAACTTTAA

>KLP2_P8_a13

ATGGAAAGATCAACTTTGATCAATTTACTTCTGTTACACAAATTTGAACACAAGATTAACACTGAAGGAATCATTGTTGTGCACGGAATTGCTGGAACTGGGAAAACCACATTGCTTAGGACTTTATTTTCTGCATACCCTAGCTTAGTTATAGGTTCACCTAGGCCTTGCTACTTAGATAAAGCTAATAAAATTTCACAAGTTTGCCTTTCTTGTTTTCCAAATACCTTGTGTGACATTGTTGACGAGTACCATCTCTTAGAAAGTTTTCCTGAACCAAAGCTAGCCATTTTTGGTGACCCCTGTCAGTGCACTTACATTGAAAGGTTGAGAACACCCAACTACACATCCTTCAGAACACACCGATTTGGCAAATCCACTGCTGCTCTACTAAACAAGTTATTTGATCTTAACATTGAGTCAGTCAAAGCACAAGACGACACAGTGGAATACTTTGATCCTTTCGCAGTGGACCCCTCTGAACACATTTCTGCATCAGAAAAAGAAGTTTTGGAATTTGTAGGTGATCAAGTTGAGACTACAAGCTCTGAAGAACTAGCTGGTCTCGAGTTTAGTGAAGTTACTTTCTACTGTACCACACTTGCTGGTGCTGTTCAAGAAAATCCTGCCAAAACTTTCATTTCACTAACTAGACACACTTCAAAGCTCACAATTGGTGAACTAAATGCCAGGTCTGACTCCTAGATGCCAGGTCTGACTCCTAGAGCTGACCTTACTGACACGTATAAAATCATTGCTATAGCCCTTCTACTGTCAGCTTGCATTTACTTCCAAAACAGTCATTATCAACCAGTTGCAGGTGATAACTTGCACAGACTACCCTTCGGTGGCCAGTATCAAGACGGAACTAAGAAGATTTCTTACTTTCCGCAGCAACAATCCTACTTTCACTCAGGAAACAAGCTTAATGTCCTCATACTTATCTTCATTCTTACACTGGGTATTGTCCTCACCAATAAATTTAGTTTTAGCATTAGCCGTAATACTCACCAGCATCATTGCTACAATACACATTCTGCAACCCAAACAGGTCAATCAGTGCCAGGTCATCATTGAATGTCCTCATACTTATCTTCATTCTTACACTGGGTATTGTCCTCACCAATAAATTTAGTTTTAGCATTAGCCGTAATACTCACCAGCATCATTGCTACAATACACATTCTGCAACCCAAACAGGTCAATCAGTGCCAGGTCATCATTGACGGTGCAGTCATAGTTATAACAAATTGTCCAAACACACCCGAAGTTCTTAAAGCAATCAACTTCTCCCCTTGGAACGGGTTAAGTTTTCCTCAATTGTGAATGGAAAACCAACCTACAGCTTCTAACCCATCAGATGTACCACCAACTGCCGCTCAAGCTGGTGCCCAGAGCCCGGCCGACTTCTCAAATCCTAATACAGCTCCTTCCCTAAGTGATTTGAAGAAAATCAAATACGTGTCAACTGTCACTTCAGTTGCCACGCCTGCTGAAATTGAGGCCCTTGGCAAGATCTTTACTGCCATGGGTTTAGCAGCCAATGAGACCGGACCTGCCATGTGGGACCTCGCTCGTGCTTATGCTGATGTGCAAAGTTCAAAATCTGCACAACTTATAGGTGCCACACCATCCAACCCTGCTTTGTCTAGACGTGCACTTGCTGCACAGTTTGATCGTATCAATATCACACCCAGACAATTCTGCATGTATTTTGCAAAAATTGTTTGGAACATACTGTTAGACAGCAATGTGCCACCTGCCAACTGGGCAAAATTGGGCTATCAGAAAGATACCAAATTTGCTGCTTTTGACTTCTTTGATGGAGTCACAAATCCAGCTAGTCTACAGCCTGCAGATGGCCTAATCAGGCAGCCCAATGAAAAAGAGCTTGCTGCCCACTCGGTTGCTAAATATGGCGCCCTTGCCCGCCAGAAAATATCCACTGGTAACTACATCACCACCCTTGGTGAAGTTACACGTGGTCACATGGGCGGCGCCAACACTATGTACGCAATTGATGCACCTCCTGAACTTTAA

>KLP2_P8_a12

ATGGAAAGATCAACTTTGATCAATTTACTTCTGTTACACAAATTTGAACACAAGATTAACACTGAAGGAATCATTGTTGTGCACGGAATTGCTGGAACTGGGAAAACCACATTGCTTAGGACTTTATTTTCTGCATACCCTAGCTTAGTTATAGGTTCACCTAGGCCTTGCTACTTAGATAAAGCTAATAAAATTTCACAAGTTTGCCTTTCTTGTTTTCCAAATACCTTGTGTGACATTGTTGACGAGTACCATCTCTTAGAAAGTTTTCCTGAACCAAAACTAGCCATTTTTGGTGACCCCTGTCAGTGCACTTACATTGAAAGGTTGAGAACACCCAACTACACATCCTTCAGAACACACCGATTTGGCAAATCCACTGCTGCTCTACTAAACAAGTTATTTGATCTTAACATTGAGTCAGTCAAAGCACAAGACGACACAGTGGAATACTTTGATCCTTTCGCAGTGGACCCCTCTGAACACATTTCTGCATCAGAAAAAGAAGTTTTGGAATTTGTAGGTGATCAAGTTGAGACTACAAGCTCTGAAGAACTAGCTGGTCTCGAGTTTAGTGAAGTTACTTTCTACTGTACCACACTTGCTGGTGCTGTTCAAGAAAATCCTGCCAAAACTTTCATTTCACTAACTAGACACACTTCAAAGCTCACAATTGGTGAACTAAATGCCAGGTCTGACTCCTAGATGCCAGGTCTGACTCCTAGAGCTGACCTTACTGACACGTATAAAATCATTGCTATAGCCCTTCTACTGTCAGCTTGCATTTACTTCCAAAACAGTCATTATCAACCAGTTGCAGGTGATAACTTGCACAGACTACCCTTCGGTGGCCAGTATCAAGACGGAACTAAGAAGATTTCTTACTTTCCGCAGCAACAATCCTACTTTCACTCAGGAAACAAGCTTAATGTCCTCATACTTATCTTCATTCTTACACTGGGTATTGTCCTCACCAATAAATTTAGTTTTAGCATTAGCCGTAATACTCACCAGCATCATTGCTACAATACACATTCTGCAACCCAAACAGGTCAATCAGTGCCAGGTCATCATTGAATGTCCTCATACTTATCTTCATTCTTACACTGGGTATTGTCCTCACCAATAAATTTAGTTTTAGCATTAGCCGTAATACTCACCAGCATCATTGCTACAATACACATTCTGCAACCCAAACAGGTCAATCAGTGCCAGGTCATCATTGACGGTGCAGCCATAGTTATAACAAATTGTCCAAACACACCCGAAGTTCTTAAAGCAATCAACTTCTCCCCTTGGAACGGGTTAAGTTTTCCTCAATTGTGAATGGAAAACCAACCTACAGCTTCTAACCCATCAGATGTACCACCAACTGCCGCTCAAGCTGGTGCCCAGAGCCCGGCCGACTTCTCAAATCCTAATACAGCTCCTTCCCTAAGTGATTTGAAGAAAATCAAATACGTGTCAACTGTCACTTCAGTTGCCACGCCTGCTGAAATTGAGGCCCTTGGCAAGATCTTTACTGCCATGGGTTTAGCAGCCAATGAGACCGGACCTGCCATGTGGGACCTCGCTCGTGCTTATGCTGATGTGCAAAGTTCAAAATCTGCACAACTTATAGGTGCCACACCATCCAACCCTGCTTTGTCTAGACGTGCACTTGCTGCACAGTTTGATCGTATCAATATCACACCCAGACAATTCTGCATGTATTTTGCAAAAATTGTTTGGAACATACTGTTAGACAGCAATGTGCCACCTGCCAACTGGGCAAAATTGGGCTATCAGAAAGATACCAAATTTGCTGCTTTTGACTTCTTTGATGGAGTCACAAATCCAGCTAGTCTACAGCCTGCAGATGGCCTAATCAGGCAGCCCAATGAAAAAGAGCTTGCTGCCCACTCGGTTGCTAAATATGGCGCCCTTGCCCGCCAGAAAATATCCACTGGTAACTACATCACCACCCTTGGTGAAGTTACACGTGGTCACATGGGCGGCGCCAACACTATGTACGCAATTGATGCACCTCCTGAACTTTAA

>KLP2_P8_a11

ATGGAAAGATCAACTTTGATCAATTTACTTCTGTTACACAAATTTGAACACAAGATTAACACTGAAGGAATCATTGTTGTGCACGGAATTGCTGGAACTGGGAAAACCACATTGCTTAGGACTTTATTTTCTGCATACCCTAGCTTAGTTATAGGTTCACCTAGGCCTTGCTACTTAGATAAAGCTAATAAAATTTCACAAGTTTGCCTTTCTTGTTTTCCAAATACCTTGTGTGACATTGTTGACGAGTACCATCTCTTAGAAAGTTTTCCTGAACCAAAACTAGCCATTTTTGGTGACCCCTGTCAGTGCACTTACATTGAAAGGTTGAGAACACCCAACTACACATCCTTCAGAACACACCGATTTGGCAAATCCACTGCTGCTCTACTAAACAAGTTATTTGATCTTAACATTGAGTCAGTCAAAGCACAAGACGACACAGTGGAATACTTTGATCCTTTCGCAGTGGACCCCTCTGAACACATTTCTGCATCAGAAAAAGAAGTTTTGGAATTTGTAGGTGATCAAGTTGAGACTACAAGCTCTGAAGAACTAGCTGGTCTCGAGTTTAGTGAAGTTACTTTCTACTGTACCACACTTGCTGGTGCTGTTCAAGAAAATCCTGCCAAAACTTTCATTTCACTAACTAGACACACTTCAAAGCTCACAATTGGTGAACTAAATGCCAGGTCTGACTCCTAGATGCCAGGTCTGACTCCTAGAGCTGACCTTACTGACACGTATAAAATCATTGCTATAGCCCTTCTACTGTCAGCTTGCATTTACTTCCAAAACAGTCATTATCAACCAGTTGCAGGTGATAACTTGCACAGACTACCCTTCGGTGGCCAGTATCAAGACGGAACTAAGAAGATTTCTTACTTTCCGCAGCAACAATCCTACTTTCACTCAGGAAACAAGCTTAATGTCCTCATACTTATCTTCATTCTTACACTGGGTATTGTCCTCACCAATAAATTTAGTTTTAGCATTAGCCGTAATACTCACCAGCATCATTGCTACAATACACATTCTGCAACCCAAACAGGTCAATCAGTGCCAGGTCATCATTGAATGTCCTCATACTTATCTTCATTCTTACACTGGGTATTGTCCTCACCAATAAATTTAGTTTTAGCATTAGCCGTAATACTCACCAGCATCATTGCTACAATACACATTCTGCAACCCAAACAGGTCAATCAGTGCCAGGTCATCATTGACGGTGCAGCCATAGTTATAACAAATTGTCCAAACACACCCGAAGTTCTTAAAGCAATCAACTTCTCCCCTTGGAACGGGTTAAGTTTTCCTCAATTGTGAATGGAAAACCAACCTACAGCTTCTAACCCATCAGATGTACCACCAACTGCCGCTCAAGCTGGTGCCCAGAGCCCGGCCGACTTCTCAAATCCTAATACAGCTCCTTCCCTAAGTGATTTGAAGAAAATCAAATACGTGTCAACTGTCACTTCAGTTGCCACGCCTGCTGAAATTGAGGCCCTTGGCAAGATCTTTACTGCCATGGGTTTAGCAGCCAATGAGACCGGACCTGCCATGTGGGACCTCGCTCGTGCTTATGCTGATGTGCAAAGTTCAAAATCTGCACAACTTATAGGTGCCACACCATCCAACCCTGCTTTGTCTAGACGTGCACTTGCTGCACAGTTTGATCGTATCAATATCACACCCAGACAATTCTGCATGTATTTTGCAAAAATTGTTTGGAACATACTGTTAGACAGCAATGTGCCACCTGCCAACTGGGCAAAATTGGGCTATCAGAAAGATACCAAATTTGCTGCTTTTGACTTCTTTGATGGAGTCACAAATCCAGCTAGTCTACAGCCTGCAGATGGCCTAATCAGGCAGCCCAATGAAAAAGAGCTTGCTGCCCACTCGGTTGCTAAATATGGCGCCCTTGCCCGCCAGAAAATATCCACTGGTAACTACATCACCACCCTTGGCGAAGTTACACGTGGTCACATGGGCGGCGCCAACACTATGTACGCAATTGATGCACCTCCTGAACTTTAA

>KLP2_P8_a10

ATGGAAAGATCAACTTTGATCAATTTACTTCTGTTACACAAATTTGAACACAAGATTAACACTGAAGGAATCATTGTTGTGCACGGAATTGCTGGAACTGGGAAAACCACATTGCTTAGGACTTTATTTTCTGCATACCCTAGCTTAGTTATAGGTTCACCTAGGCCTTGCTACTTAGATAAAGCTAATAAAATTTCACAAGTTTGCCTTTCTTGTTTTCCAAATACCTTGTGTGACATTGTTGACGAGTACCATCTCTTAGAAAGTTTTCCTGAACCAAAACTAGCCATTTTTGGTGACCCCTGTCAGTGCACTTACATTGAAAGGTTGAGAACACCCAACTACACATCCTTCAGAACACACCGATTTGGCAAATCCACTGCTGCTCTACTAAACAAGTTATTTGATCTTAACATTGAGTCAGTCAAAGCACAAGACGACACAGTGGAATACTTTGATCCTTTCGCAGTGGACCCCTCTGAACACATTTCTGCATCAGAAAAAGAAGTTTTGGAATTTGTAGGTGATCAAGTTGAGACTACAAGCTCTGAAGAACTAGCTGGTCTCGAGTTTAGTGAAGTTACTTTCTACTGTACCACACTTGCTGGTGCTGTTCAAGAAAATCCTGCCAAAACTTTCATTTCACTAACTAGACACACTTCAAAGCTCACAATTGGTGAACTAAATGCCAGGTCTGACTCCTAGATGCCAGGTCTGACTCCTAGAGCTGACCTTACTGACACGTATAAAATCATTGCTATAGCCCTTCTACTGTCAGCTTGCATTTACTTCCAAAACAGTCATTATCAACCAGTTGCAGGTGATAACTTGCACAGACTACCCTTCGGTGGCCAGTATCAAGACGGAACTAAGAAGATTTCTTACTTTCCGCAGCAACAATCCTACTTTCACTCAGGAAACAAGCTTAATGTCCTCATACTTATCTTCATTCTTACACTGGGTATTGTCCTCACCAATAAATTTAGTTTTAGCATTAGCCGTAATACTCACCAGCATCATTGCTACAATACACATTCTGCAACCCAAACAGGTCAATCAGTGCCAGGTCATCATTGAATGTCCTCATACTTATCTTCATTCTTACACTGGGTATTGTCCTCACCAATAAATTTAGTTTTAGCATTAGCCGTAATACTCACCAGCATCATTGCTACAATACACATTCTGCAACCCAAACAGGTCAATCAGTGCCAGGTCATCATTGACGGTGCAGCCATAGTTATAACAAATTGTCCAAACACACCCGAAGTTCTTAAAGCAATCAACTTCTCCCCTTGGAACGGGTTAAGTTTTCCTCAATTGTGAATGGAAAACCAACCTACAGCTTCTAACCCATCAGATGTACCACCAACTGCCGCTCAAGCTGGTGCCCAGAGCCCGGCCGACTTCTCAAATCCTAATACAGCTCCTTCCCTAAGTGATTTGAAGAAAATCAAATACGTGTCAACTGTCACTTCAGTTGCCACGCCTGCTGAAATTGAGGCCCTTGGCAAGATCTTTACTGCCATGGGTTTAGCAGCCAATGAGACCGGACCTGCCATGTGGGACCTCGCTCGTGCTTATGCTGATGTGCAAAGTTCAAAATCTGCACAACTTATAGGTGCCACACCATCCAACCCTGCTTTGTCTAGACGTGCACTTGCTGCACAGTTTGATCGTATCAATATCACACCCAGACAATTCTGCATGTATTTTGCAAAAATTGTTTGGAACATACTGTTAGACAGCAATGTGCCACCTGCCAACTGGGCAAAATTGGGCTATCAGAAAGATACCAAATTTGCTGCTTTTGACTTCTTTGATGGAGTCACAAATCCAGCTAGTCTACAGCCTGCAGATGGCCTAATCAGGCAGCCCAATGAAAAAGAGCTTGCTGCCCACTCGGTTGCTAAATATGGCGCCCTTGCCCGCCAGAAAATATCCACTGGTAACTACATCACCACCCTTGGTGAAGTTACACGTGGTCACATGGGCGGCGCCAACACTATGTACGCAATTGATGCACCTCCTGAACTTTAA

>KLP2_P8_a9

ATGGAAAGATCAACTTTGATCAATTTACTTCTGTTACACAAATTTGAACACAAGATTAACACTGAAGGAATCATTGTTGTGCACGGAATTGCTGGAACTGGGAAAACCACATTGCTTAGGACTTTATTTTCTGCATACCCTAGCTTAGTTATAGGTTCACCTAGGCCTTGCTACTTAGATAAAGCTAATAAAATTTCACAAGTTTGCCTTTCTTGTTTTCCAAATACCTTGTGTGACATTGTTGACGAGTACCATCTCTTAGAAAGTTTTCCTGAACCAAAACTAGCCATTTTTGGTGACCCCTGTCAGTGCACTTACATTGAAAGGTTGAGAACACCCAACTACACATCCTTCAGAACACACCGATTTGGCAAATCCACTGCTGCTCTACTAAACAAGTTATTTGATCTTAACATTGAGTCAGTCAAAGCACAAGACGACACAGTGGAATACTTTGATCCTTTCGCAGTGGACCCCTCTGAACACATTTCTGCATCAGAAAAAGAAGTTTTGGAATTTGTAGGTGATCAAGTTGAGACTACAAGCTCTGAAGAACTAGCTGGTCTCGAGTTTAGTGAAGTTACTTTCTACTGTACCACACTTGCTGGTGCTGTTCAAGAAAATCCTGCCAAAACTTTCATTTCACTAACTAGACACACTTCAAAGCTCACAATTGGTGAACTAAATGCCAGGTCTGACTCCTAGATGCCAGGTCTGACTCCTAGAGCTGACCTTACTGACACGTATAAAATCATTGCTATAGCCCTTCTACTGTCAGCTTGCATTTACTTCCAAAACAGTCATTATCAACCAGTTGCAGGTGATAACTTGCACAGACTACCCTTCGGTGGCCAGTATCAAGACGGAACTAAGAAGATTTCTTACTTTCCGCAGCAACAATCCTACTTTCACTCAGGAAACAAGCTTAATGTCCTCATACTTATCTTCATTCTTACACTGGGTATTGTCCTCACCAATAAATTTAGTTTTAGCATTAGCCGTAATACTCACCAGCATCATTGCTACAATACACATTCTGCAACCCAAACAGGTCAATCAGTGCCAGGTCATCATTGAATGTCCTCATACTTATCTTCATTCTTACACTGGGTATTGTCCTCACCAATAAATTTAGTTTTAGCATTAGCCGTAATACTCACCAGCATCATTGCTACAATACACATTCTGCAACCCAAACAGGTCAATCAGTGCCAGGTCATCATTGACGGTGCAGCCATAGTTATAACAAATTGTCCAAACACACCCGAAGTTCTTAAAGCAATCAACTTCTCCCCTTGGAACGGGTTAAGTTTTCCTCAATTGTGAATGGAAAACCAACCTACAGCTTCTAACCCATCAGATGTACCACCAACTGCCGCTCAAGCTGGTGCCCAGAGCCCGGCCGACTTCTCAAATCCTAATACAGCTCCTTCCCTAAGTGATTTGAAGAAAATCAAATACGTGTCAACTGTCACTTCAGTTGCCACGCCTGCTGAAATTGAGGCCCTTGGCAAGATCTTTACTGCCATGGGTTTAGCAGCCAATGAGACCGGACCTGCCATGTGGGACCTCGCTCGTGCTTATGCTGATGTGCAAAGTTCAAAATCTGCACAACTTATAGGTGCCACACCATCCAACCCTGCTTTGTCTAGACGTGCACTTGCTGCACAGTTTGATCGTATCAATATCACACCCAGACAATTCTGCATGTATTTTGCAAAAATTGTTTGGAACATACTGTTAGACAGCAATGTGCCACCTGCCAACTGGGCAAAATTGGGCTATCAGAAAGATACCAAATTTGCTGCTTTTGACTTCTTTGATGGAGTCACAAATCCAGCTAGTCTACAGCCTGCAGATGGCCTAATCAGGCAGCCCAATGAAAAAGAGCTTGCTGCCCACTCGGTTGCTAAATATGGCGCCCTTGCCCGCCAGAAAATATCCACTGGTAACTACATCACCACCCTTGGTGAAGTTACACGTGGTCACATGGGCGGCGCCAACACTATGTACGCAATTGATGCACCTCCTGAACTTTAA

>KLP2_P8_a8

ATGGAAAGATCAACTTTGATCAATTTACTTCTGTTACACAAATTTGAACACAAGATTAACACTGAAGGAATCATTGTTGTGCACGGAATTGCTGGAACTGGGAAAACCACATTGCTTAGGACTTTATTTTCTGCATACCCTAGCTTAGTTATAGGTTCACCTAGGCCTTGCTACTTAGATAAAGCTAATAAAATTTCACAAGTTTGCCTTTCTTGTTTTCCAAATACCTTGTGTGACATTGTTGACGAGTACCATCTCTTAGAAAGTTTTCCTGAACCAAAACTAGCCATTTTTGGTGACCCCTGTCAGTGCACTTACATTGAAAGGTTGAGAACACCCAACTACACATCCTTCAGAACACACCGATTTGGCAAATCCACTGCTGCTCTACTAAACAAGTTATTTGATCTTAACATTGAGTCAGTCAAAGCACAAGACGACACAGTGGAATACTTTGATCCTTTCGCAGTGGACCCCTCTGAACACATTTCTGCATCAGAAAAAGAAGTTTTGGAATTTGTAGGTGATCAAGTTGAGACTACAAGCTCTGAAGAACTAGCTGGTCTCGAGTTTAGTGAAGTTACTTTCTACTGTACCACACTTGCTGGTGCTGTTCAAGAAAATCCTGCCAAAACTTTCATTTCACTAACTAGACACACTTCAAAGCTCACAATTGGTGAACTAAATGCCAGGTCTGACTCCTAGATGCCAGGTCTGACTCCTAGAGCTGACCTTACTGACACGTATAAAATCATTGCTATAGCCCTTCTACTGTCAGCTTGCATTTACTTCCAAAACAGTCATTATCAACCAGTTGCAGGTGATAACTTGCACAGACTACCCTTCGGTGGCCAGTATCAAGACGGAACTAAGAAGATTTCTTACTTTCCGCAGCAACAATCCTACTTTCACTCAGGAAACAAGCTTAATGTCCTCATACTTATCTTCATTCTTACACTGGGTATTGTCCTCACCAATAAATTTAGTTTTAGCATTAGCCGTAATACTCACCAGCATCATTGCTACAATACACATTCTGCAACCCAAACAGGTCAATCAGTGCCAGGTCATCATTGAATGTCCTCATACTTATCTTCATTCTTACACTGGGTATTGTCCTCACCAATAAATTTAGTTTTAGCATTAGCCGTAATACTCACCAGCATCATTGCTACAATACACATTCTGCAACCCAAACAGGTCAATCAGTGCCAGGTCATCATTGACGGTGCAGCCATAGTTATAACAAATTGTCCAAACACACCCGAAGTTCTTAAAGCAATCAACTTCTCCCCTTGGAACGGGTTAAGTTTTCCTCAATTGTGAATGGAAAACCAACCTACAGCTTCTAACCCATCAGATGTACCACCAACTGCCGCTCAAGCTGGTGCCCAGAGCCCGGCCGACTTCTCAAATCCTAATACAGCTCCTTCCCTAAGTGATTTGAAGAAAATCAAATACGTGTCAACTGTCACTTCAGTTGCCACGCCTGCTGAAATTGAGGCCCTTGGCAAGATCTTTACTGCCATGGGTTTAGCAGCCAATGAGACCGGACCTGCCATGTGGGACCTCGCTCGTGCTTATGCTGATGTGCAAAGTTCAAAATCTGCACAACTTATAGGTGCCACACCATCCAACCCTGCTTTGTCTAGACGTGCACTTGCTGCACAGTTTGATCGTATCAATATCACACCCAGACAATTCTGCATGTATTTTGCAAAAATTGTTTGGAACATACTGTTAGACAGCAATGTGCCACCTGCCAACTGGGCAAAATTGGGCTATCAGAAAGATACCAAATTTGCTGCTTTTGACTTCTTTGATGGAGTCACAAATCCAGCTAGTCTACAGCCTGCAGATGGCCTAATCAGGCAGCCCAATGAAAAAGAGCTTGCTGCCCACTCGGTTGCTAAATATGGCGCCCTTGCCCGCCAGAAAATATCCACTGGTAACTACATCACCACCCTTGGTGAAGTTACACGTGGTCACATGGGCGGCGCCAACACTATGTACGCAATTGATGCACCTCCTGAACTTTAA

>KLP2_P8_a7

ATGGAAAGATCAACTTTGATCAATTTACTTCTGTTACACAAATTTGAACACAAGATTAACACTGAAGGAATCATTGTTGTGCACGGAATTGCTGGAACTGGGAAAACCACATTGCTTAGGACTTTATTTTCTGCATACCCTAGCTTAGTTATAGGTTCACCTAGGCCTTGCTACTTAGATAAAGCTAATAAAATTTCACAAGTTTGCCTTTCTTGTTTTCCAAATACCTTGTGTGACATTGTTGACGAGTACCATCTCTTAGAAAGTTTTCCTGAACCAAAACTAGCCATTTTTGGTGACCCCTGTCAGTGCACTTACATTGAAAGGTTGAGAACACCCAACTACACATCCTTCAGAACACACCGATTTGGCAAATCCACTGCTGCTCTACTAAACAAGTTATTTGATCTTAACATTGAGTCAGTCAAAGCACAAGACGACACAGTGGAATACTTTGATCCTTTCGCAGTGGACCCCTCTGAACACATTTCTGCATCAGAAAAAGAAGTTTTGGAATTTGTAGGTGATCAAGTTGAGACTACAAGCTCTGAAGAACTAGCTGGTCTCGAGTTTAGTGAAGTTACTTTCTACTGTACCACACTTGCTGGTGCTGTTCAAGAAAATCCTGCCAAAACTTTCATTTCACTAACTAGACACACTTCAAAGCTCACAATTGGTGAACTAAATGCCAGGTCTGACTCCTAGATGCCAGGTCTGACTCCTAGAGCTGACCTTACTGACACGTATAAAATCATTGCTATAGCCCTTCTACTGTCAGCTTGCATTTACTTCCAAAACAGTCATTATCAACCAGTTGCAGGTGATAACTTGCACAGACTACCCTTCGGTGGCCAGTATCAAGACGGAACTAAGAAGATTTCTTACTTTCCGCAGCAACAATCCTACTTTCACTCAGGAAACAAGCTTAATGTCCTCATACTTATCTTCATTCTTACACTGGGTATTGTCCTCACCAATAAATTTAGTTTTAGCATTAGCCGTAATACTCACCAGCATCATTGCTACAATACACATTCTGCAACCCAAACAGGTCAATCAGTGCCAGGTCATCATTGAATGTCCTCATACTTATCTTCATTCTTACACTGGGTATTGTCCTCACCAATAAATTTAGTTTTAGCATTAGCCGTAATACTCACCAGCATCATTGCTACAATACACATTCTGCAACCCAAACAGGTCAATCAGTGCCAGGTCATCATTGACGGTGCAGCCATAGTTATAACAAATTGTCCAAACACACCCGAAGTTCTTAAAGCAATCAACTTCTCCCCTTGGAACGGGTTAAGTTTTCCTCAATTGTGAATGGAAAACCAACCTACAGCTTCTAACCCATCAGATGTACCACCAACTGCCGCTCAAGCTGGTGCCCAGAGCCCGGCCGACTTCTCAAATCCTAATACAGCTCCTTCCCTAAGTGATTTGAAGAAAATCAAATACGTGTCAACTGTCACTTCAGTTGCCACGCCTGCTGAAATTGAGGCCCTTGGCAAGATCTTTACTGCCATGGGTTTAGCAGCCAATGAGACCGGACCTGCCATGTGGGACCTCGCTCGTGCTTATGCTGATGTGCAAAGTTCAAAATCTGCACAACTTATAGGTGCCACACCATCCAACCCTGCTTTGTCTAGACGTGCACTTGCTGCACAGTTTGATCGTATCAATATCACACCCAGACAATTCTGCATGTATTTTGCAAAAATTGTTTGGAACATACTGTTAGACAGCAATGTGCCACCTGCCAACTGGGCAAAATTGGGCTATCAGAAAGATACCAAATTTGCTGCTTTTGACTTCTTTGATGGAGTCACAAATCCAGCTAGTCTACAGCCTGCAGATGGCCTAATCAGGCAGCCCAATGGAAAAGAGCTTGCTGCCCACTCGGTTGCTAAATATGGCGCCCTTGCCCGCCAGAAAATATCCACTGGTAACTACATCACCACCCTTGGTGAAGTTACACGTGGTCACATGGGCGGCGCCAACACTATGTACGCAATTGATGCACCTCCTGAACTTTAA

>KLP2_P8_a6

ATGGAAAGATCAACTTTGATCAATTTACTTCTGTTACACAAATTTGAACACAAGATTAACACTGAAGGAATCATTGTTGTGCACGGAATTGCTGGAACTGGGAAAACCACATTGCTTAGGACTTTATTTTCTGCATACCCTAGCTTAGTTATAGGTTCACCTAGGCCTTGCTACTTAGATAAAGCTAATAAAATTTCACAAGTTTGCCTTTCTTGTTTTCCAAATACCTTGTGTGACATTGTTGACGAGTACCATCTCTTAGAAAGTTTTCCTGAACCAAAACTAGCCATTTTTGGTGACCCCTGTCAGTGCACTTACATTGAAAGGTTGAGAACACCCAACTACACATCCTTCAGAACACACCGATTTGGCAAATCCACTGCTGCTCTACTAAACAAGTTATTTGATCTTAACATTGAGTCAGTCAAAGCACAAGACGACACAGTGGAATACTTTGATCCTTTCGCAGTGGACCCCTCTGAACACATTTCTGCATCAGAAAAAGAAGTTTTGGAATTTGTAGGTGATCAAGTTGAGACTACAAGCTCTGAAGAACTAGCTGGTCTCGAGTTTAGTGAAGTTACTTTCTACTGTACCACACTTGCTGGTGCTGTTCAAGAAAATCCTGCCAAAACTTTCATTTCACTAACTAGACACACTTCAAAGCTCACAATTGGTGAACTAAATGCCAGGTCTGACTCCTAGATGCCAGGTCTGACTCCTAGAGCTGACCTTACTGACACGTATAAAATCATTGCTATAGCCCTTCTACTGTCAGCTTGCATTTACTTCCAAAACAGTCATTATCAACCAGTTGCAGGTGATAACTTGCACAGACTACCCTTCGGTGGCCAGTATCAAGACGGAACTAAGAAGATTTCTTACTTTCCGCAGCAACAATCCTACTTTCACTCAGGAAACAAGCTTAATGTCCTCATACTTATCTTCATTCTTACACTGGGTATTGTCCTCACCAATAAATTTAGTTTTAGCATTAGCCGTAATACTCACCAGCATCATTGCTACAATACACATTCTGCAACCCAAACAGGTCAATCAGTGCCAGGTCATCATTGAATGTCCTCATACTTATCTTCATTCTTACACTGGGTATTGTCCTCACCAATAAATTTAGTTTTAGCATTAGCCGTAATACTCACCAGCATCATTGCTACAATACACATTCTGCAACCCAAACAGGTCAATCAGTGCCAGGTCATCATTGACGGTGCAGCCATAGTTATAACAAATTGTCCAAACACACCCGAAGTTCTTAAAGCAATCAACTTCTCCCCTTGGAACGGGTTAAGTTTTCCTCAATTGTGAATGGAAAACCAACCTACAGCTTCTAACCCATCAGATGTACCACCAACTGCCGCTCAAGCTGGTGCCCAGAGCCCGGCCGACTTCTCAAATCCTAATACAGCTCCTTCCCTAAGTGATTTGAAGAAAATCAAATACGTGTCAACTGTCACTTCAGTTGCCACGCCTGCTGAAATTGAGGCCCTTGGCAAGATCTTTACTGCCATGGGTTTAGCAGCCAATGAGACCGGACCTGCCATGTGGGACCTCGCTCGTGCTTATGCTGATGTGCAAAGTTCAAAATCTGCACAACTTATAGGTGCCACACCATCCAACCCTGCTTTGTCTAGACGTGCACTTGCTGCACAGTTTGATCGTATCAATATCACACCCAGACAATTCTGCATGTATTTTGCAAAAATTGTTTGGAACATACTGTTAGACAGCAATGTGCCACCTGCCAACTGGGCAAAATTGGGCTATCAGAAAGATACCAAATTTGCTGCTTTTGACTTCTTTGATGGAGTCACAAATCCAGCTAGTCTACAGCCTGCAGATGGCCTAATCAGGCAGCCCAATGAAAAAGAGCTTGCTGCCCACTCGGTTGCTAAATATGGCGCCCTTGCCCGCCAGAAAATATCCACTGGTAACTACATCACCACCCTTGGTGAAGTTACACGTGGTCACATGGGCGGCGCCAACACTATGTACGCAATTGATGCACCTCCTGAACTTTAA

>KLP2_P8_a5

ATGGAAAGATCAACTTTGATCAATTTACTTCTGTTACACAAATTTGAACACAAGATTAACACTGAAGGAATCATTGTTGTGCACGGAATTGCTGGAACTGGGAAAACCACATTGCTTAGGACTTTATTTTCTGCATACCCTAGCTTAGTTATAGGTTCACCTAGGCCTTGCTACTTAGATAAAGCTAATAAAATTTCACAAGTCTGCCTTTCTTGTTTTCCAAATACCTTGTGTGACATTGTTGACGAGTACCATCTCTTAGAAAGTTTTCCTGAACCAAAACTAGCCATTTTTGGTGACCCCTGTCAGTGCACTTACATTGAAAGGTTGAGAACACCCAACTACACATCCTTCAGAACACACCGATTTGGCAAATCCACTGCTGCTCTACTAAACAAGTTATTTGATCTTAACATTGAGTCAGTCAAAGCACAAGACGACACAGTGGAATACTTTGATCCTTTCGCAGTGGACCCCTCTGAACACATTTCTGCATCAGAAAAAGAAGTTTTGGAATTTGTAGGTGATCAAGTTGAGACTACAAGCTCTGAAGAACTAGCTGGTCTCGAGTTTAGTGAAGTTACTTTCTACTGTACCACACTTGCTGGTGCTGTTCAAGAAAATCCTGCCAAAACTTTCATTTCACTAACTAGACACACTTCAAAGCTCACAATTGGTGAACTAAATGCCAGGTCTGACTCCTAGATGCCAGGTCTGACTCCTAGAGCTGACCTTACTGACACGTATAAAATCATTGCTATAGCCCTTCTACTGTCAGCTTGCATTTACTTCCAAAACAGTCATTATCAACCAGTTGCAGGTGATAACTTGCACAGACTACCCTTCGGTGGCCAGTATCAAGACGGAACTAAGAAGATTTCTTACTTTCCGCAGCAACAATCCTACTTTCACTCAGGAAACAAGCTTAATGTCCTCATACTTATCTTCATTCTTACACTGGGTATTGTCCTCACCAATAAATTTAGTTTTAGCATTAGCCGTAATACTCACCAGCATCATTGCTACAATACACATTCTGCAACCCAAACAGGTCAATCAGTGCCAGGTCATCATTGAATGTCCTCATACTTATCTTCATTCTTACACTGGGTATTGTCCTCACCAATAAATTTAGTTTTAGCATTAGCCGTAATACTCACCAGCATCATTGCTACAATACACATTCTGCAACCCAAACAGGTCAATCAGTGCCAGGTCATCATTGACGGTGCAGCCATAGTTATAACAAATTGTCCAAACACACCCGAAGTTCTTAAAGCAATCAACTTCTCCCCTTGGAACGGGTTAAGTTTTCCTCAATTGTGAATGGAAAACCAACCTACAGCTTCTAACCCATCAGATGTACCACCAACTGCCGCTCAAGCTGGTGCCCAGAGCCCGGCCGACTTCTCAAATCCTAATACAGCTCCTTCCCTAAGTGATTTGAAGAAAATCAAATACGTGTCAACTGTCACTTCAGTTGCCACGCCTGCTGAAATTGGGGCCCTTGGCAAGATCTTTACTGCCATGGGTTTAGCAGCCAATGAGACCGGACCTGCCATGTGGGACCTCGCTCGTGCTTATGCTGATGTGCAAAGTTCAAAATCTGCACAACTTATAGGTGCCACACCATCCAACCCTGCTTTGTCTAGACGTGCACTTGCTGCACAGTTTGATCGTATCAATATCACACCCAGACAATTCTGCATGTATTTTGCAAAAATTGTTTGGAACATACTGTTAGACAGCAATGTGCCACCTGCCAACTGGGCAAAATTGGGCTATCAGAAAGATACCAAATTTGCTGCTTTTGACTTCTTTGATGGAGTCACAAATCCAGCTAGTCTACAGCCTGCAGATGGCCTAATCAGGCAGCCCAATGAAAAAGAGCTTGCTGCCCACTCGGTTGCTAAATATGGCGCCCTTGCCCGCCAGAAAATATCCACTGGTAACTACATCACCACCCTTGGTGAAGTTACACGTGGTCACATGGGCGGCGCCAACACTATGTACGCAATTGATGCACCTCCTGAACTTTAA

>KLP2_P8_a4

ATGGAAAGATCAACTTTGATCAATTTACTTCTGTTACACAAATTTGAACACAAGATTAACACTGAAGGAATCATTGTTGTGCACGGAATTGCTGGAACTGGGAAAACCACATTGCTTAGGACTTTATTTTCTGCATACCCTAGCTTAGTTATAGGTTCACCTAGGCCTTGCTACTTAGATAAAGCTAATAAAATTTCACAAGTTTGCCTTTCTTGTTTTCCAAATACCTTGTGTGACATTGTTGACGAGTACCATCTCTTAGAAAGTTTTCCTGAACCAAAACTAGCCATTTTTGGTGACCCCTGTCAGTGCACTTACATTGAAAGGTTGAGAACACCCAACTACACATCCTTCAGAACACACCGATTTGGCAAATCCACTGCTGCTCTACTAAACAAGTTATTTGATCTTAACATTGAGTCAGTCAAAGCACAAGACGACACAGTGGAATACTTTGATCCTTTCGCAGTGGACCCCTCTGAACACATTTCTGCATCAGAAAAAGAAGTTTTGGAATTTGTAGGTGATCAAGTTGAGACTACAAGCTCTGAAGAACTAGCTGGTCTCGAGTTTAGTGAAGTTACTTTCTACTGTACCACACTTGCTGGTGCTGTTCAAGAAAATCCTGCCAAAACTTTCATTTCACTAACTAGACACACTTCAAAGCTCACAATTGGTGAACTAAATGCCAGGTCTGACTCCTAGATGCCAGGTCTGACTCCTAGAGCTGACCTTACTGACACGTATAAAATCATTGCTATAGCCCTTCTACTGTCAGCTTGCATTTACTTCCAAAACAGTCATTATCAACCAGTTGCAGGTGATAACTTGCACAGACTACCCTTCGGTGGCCAGTATCAAGACGGAACTAAGAAGATTTCTTACTTTCCGCAGCAACAATCCTACTTTCACTCAGGAAACAAGCTTAATGTCCTCATACTTATCTTCATTCTTACACTGGGTATTGTCCTCACCAATAAATTTAGCTTTAGCATTAGCCGTAATACTCACCAGCATCATTGCTACAATACACATTCTGCAACCCAAACAGGTCAATCAGTGCCAGGTCATCATTGAATGTCCTCATACTTATCTTCATTCTTACACTGGGTATTGTCCTCACCAATAAATTTAGCTTTAGCATTAGCCGTAATACTCACCAGCATCATTGCTACAATACACATTCTGCAACCCAAACAGGTCAATCAGTGCCAGGTCATCATTGACGGTGCAGCCATAGTTATAACAAATTGTCCAAACACACCCGAAGTTCTTAAAGCAATCAACTTCTCCCCTTGGAACGGGTTAAGTTTTCCTCAATTGTGAATGGAAAACCAACCTACAGCTTCTAACCCATCAGATGTACCACCAACTGCCGCTCAAGCTGGTGCCCAGAGCCCGGCCGACTTCTCAAATCCTAATACAGCTCCTTCCCTAAGTGATTTGAAGAAAATCAAATACGTGTCAACTGTCACTTCAGTTGCCACGCCTGCTGAAATTGAGGCCCTTGGCAAGATCTTTACTGCCATGGGTTTAGCAGCTAATGAGACCGGACCTGCCATGTGGGACCTCGCTCGTGCTTATGCTGATGTGCAAAGTTCAAAATCTGCACAACTTATAGGTGCCACACCATCCAACCCTGCTTTGTCTAGACGTGCACTTGCTGCACAGTTTGATCGTATCAATATCACACCCAGACAATTCTGCATGTATTTTGCAAAAATTGTTTGGAACATACTGTTAGACAGCAATGTGCCACCTGCCAACTGGGCAAAATTGGGCTATCAGAAAGATACCAAATTTGCTGCTTTTGACTTCTTTGATGGAGTCACAAATCCAGCTAGTCTACAGCCTGCAGATGGCCTAATCAGGCAGCCCAATGAAAAAGAGCTTGCTGCCCACTCGGTTGCTAAATATGGCGCCCTTGCCCGCCAGAAAATATCCACTGGTAACTACATCACCACCCTTGGTGAAGTTACACGTGGTCACATGGGCGGCGCCAACACTATGTACGCAATTGATGCACCTCCTGAACTTTAA

>KLP2_P8_a3

ATGGAAAGATCAACTTTGATCAATTTACTTCTGTTACACAAATTTGAACACAAGATTAACACTGAAGGAATCATTGTTGTGCACGGAATTGCTGGAACTGGGAAAACCACATTGCTTAGGACTTTATTTTCTGCATACCCTAGCTTAGTTATAGGTTCACCTAGGCCTTGCTACTTAGATAAAGCTAATAAAATTTCACAAGTTTGCCTTTCTTGTTTTCCAAATACCTTGTGTGACATTGTTGACGAGTACCATCTCTTAGAAAGTTTTCCTGAACCAAAACTAGCCATTTTTGGTGACCCCTGTCAGTGCACTTACATTGAAAGGTTGAGAACACCCAACTACACATCCTTCAGAACACACCGATTTGGCAAATCCACTGCTGCTCTACTAAACAAGTTATTTGATCTTAACATTGAGTCAGTCAAAGCACAAGACGACACAGTGGAATACTTTGATCCTTTCGCAGTGGACCCCTCTGAACACATTTCTGCATCAGAAAAAGAAGTTTTGGAATTTGTAGGTGATCAAGTTGAGACTACAAGCTCTGAAGAACTAGCTGGTCTCGAGTTTAGTGAAGTTACTTTCTACTGTACCACACTTGCTGGTGCTGTTCAAGAAAATCCTGCCAAAACTTTCATTTCACTAACTAGACACACTTCAAAGCTCACAATTGGTGAACTAAATGCCAGGTCTGACTCCTAGATGCCAGGTCTGACTCCTAGAGCTGACCTTACTGACACGTATAAAATCATTGCTATAGTCCTTCTACTGTCAGCTTGCATTTACTTCCAAAACAGTCATTATCAACCAGTTGCAGGTGATAACTTGCACAGACTACCCTTCGGTGGCCAGTATCAAGACGGAACTAAGAAGATTTCTTACTTTCCGCAGCAACAATCCTACTTTCACTCAGGAAACAAGCTTAATGTCCTCATACTTATCTTCATTCTTACACTGGGTATTGTCCTCACCAATAAATTTAGTTTTAGCATTAGCCGTAATACTCACCAGCATCATTGCTACAATACACATTCTGCAACCCAAACAGGTCAATCAGTGCCAGGTCATCATTGAATGTCCTCATACTTATCTTCATTCTTACACTGGGTATTGTCCTCACCAATAAATTTAGTTTTAGCATTAGCCGTAATACTCACCAGCATCATTGCTACAATACACATTCTGCAACCCAAACAGGTCAATCAGTGCCAGGTCATCATTGACGGTGCAGCCATAGTTATAACAAATTGTCCAAACACACCCGAAGTTCTTAAAGCAATCAACTTCTCCCCTTGGAACGGGTTAAGTTTTCCTCAATTGTGAATGGAAAACCAACCTACAGCTTCTAACCCATCAGATGTACCACCAACTGCCGCTCAAGCTGGTGCCCAGAGCCCGGCCGACTTCTCAAATCCTAATACAGCTCCTTCCCTAAGTGATTTGAAGAAAATCAAATACGTGTCAACTGTCACTTCAGTTGCCACGCCTGCTGAAATTGAGGCCCTTGGCAAGATCTTTACTGCCATGGGTTTAGCAGCCAATGAGACCGGACCTGCCATGTGGGACCTCGCTCGTGCTTATGCTGATGTGCAAAGTTCAAAATCTGCACAACTTATAGGTGCCACACCATCCAACCCTGCTTTGTCTAGACGTGCACTTGCTGCACAGTTTGATCGTATCAATATCACACCCAGACAATTCTGCATGTATTTTGCAAAAATTGTTTGGAACATACTGTTAGACAGCAATGTGCCACCTGCCAACTGGGCAAAATTGGGCTATCAGAAAGATACCAAATTTGCTGCTTTTGACTTCTTTGATGGAGTCACAAATCCAGCTAGTCTACAGCCTGCAGATGGCCTAATCAGGCAGCCCAATGAAAAAGAGCTTGCTGCCCACTCGGTTGCTAAATATGGCGCCCTTGCCCGCCAGAAAATATCCACTGGTAACTACATCACCACCCTTGGTGAAGTTACACGTGGTCACATGGGCGGCGCCAACACTATGTACGCAATTGATGCACCTCCTGAACTTTAA

>KLP2_P8_a2

ATGGAAAGATCAACTTTGATCAATTTACTTCTGTTACACAAATTTGAACACAAGATTAACACTGAAGGAATCATTGTTGTGCACGGAATTGCTGGAACTGGGAAAACCACATTGCTTAGGACTTTATTTTCTGCATACCCTAGCTTAGTTATAGGTTCACCTAGGCCTTGCTACTTAGATAAAGCTAATAAAATTTCACAAGTTTGCCTTTCTTGTTTTCCAAATACCTTGTGTGACATTGTTGACGAGTACCATCTCTTAGAAAGTTTTCCTGAACCAAAACTAGCCATTTTTGGTGACCCCTGTCAGTGCACTTACATTGAAAGGTTGAGAACACCCAACTACACATCCTTCAGAACACACCGATTTGGCAAATCCACTGCTGCTCTACTAAACAAGTTATTTGATCTTAACATTGAGTCAGTCAAAGCACAAGACGACACAGTGGAATACTTTGATCCTTTCGCAGTGGACCCCTCTGAACACATTTCTGCATCAGAAAAAGAAGTTTTGGAATTTGTAGGTGATCAAGTTGAGACTACAAGCTCTGAAGAACTAGCTGGTCTCGAGTTTAGTGAAGTTACTTTCTACTGTACCACACTTGCTGGTGCTGTTCAAGAAAATCCTGCCAAAACTTTCATTTCACTAACTAGACACACTTCAAAGCTCACAATTGGTGAACTAAATGCCAGGTCTGACTCCCAGATGCCAGGTCTGACTCCCAGAGCTGACCTTACTGACACGTATAAAATCATTGCTATAGCCCTTCTACTGTCAGCTTGCATTTACTTCCAAAACAGTCATTATCAACCAGTTGCAGGTGATAACTTGCACAGACTACCCTTCGGTGGCCAGTATCAAGACGGAACTAAGAAGATTTCTTACTTTCCGCAGCAACAATCCTACTTTCACTCAGGAAACAAGCTTAATGTCCTCATACTTATCTTCATTCTTACACTGGGTATTGTCCTCACCAATAAATTTAGTTTTAGCATTAGCCGTAATACTCACCAGCATCATTGCTACAATACACATTCTGCAACCCAAACAGGTCAATCAGTGCCAGGTCATCATTGAATGTCCTCATACTTATCTTCATTCTTACACTGGGTATTGTCCTCACCAATAAATTTAGTTTTAGCATTAGCCGTAATACTCACCAGCATCATTGCTACAATACACATTCTGCAACCCAAACAGGTCAATCAGTGCCAGGTCATCATTGACGGTGCAGCCATAGTTATAACAAATTGTCCAAACACACCCGAAGTTCTTAAAGCAATCAACTTCTCCCCTTGGAACGGGTTAAGTTTTCCTCAATTGTGAATGGAAAACCAACCTACAGCTTCTAACCCATCAGATGTACCACCAACTGCCGCTCAAGCTGGTGCCCAGAGCCCGGCCGACTTCTCAAATCCTAATACAGCTCCTTCCCTAAGTGATTTGAAGAAAATCAAATACGTGTCAACTGTCACTTCAGTTGCCACGCCTGCTGAAATTGAGGCCCTTGGCAAGATCTTTACTGCCATGGGTTTAGCAGCCAATGAGACCGGACCTGCCATGTGGGACCTCGCTCGTGCTTATGCTGATGTGCAAAGTTCAAAATCTGCACAACTTATAGGTGCCACACCATCCAACCCTGCTTTGTCTAGACGTGCACTTGCTGCACAGTTTGATCGTATCAATATCACACCCAGACAATTCTGCATGTATTTTGCAAAAATTGTTTGGAACATACTGTTAGACAGCAATGTGCCACCTGCCAACTGGGCAAAATTGGGCTATCAGAAAGATACCAAATTTGCTGCTTTTGACTTCTTTGATGGAGCCACAAATCCAGCTAGTCTACAGCCTGCAGATGGCCTAATCAGGCAGCCCAATGAAAAAGAGCTTGCTGCCCACTCGGTTGCTAAATATGGCGCCCTTGTCCGCCAGAAAATATCCACTGGTAACTACATCACCACCCTTGGTGAAGTTACACGTGGTCACATGGGCGGCGCCAACACTATGTACGCAATTGATGCACCTCCTGAACTTTAA

>KLP2_P8_a1

ATGGAAAGATCAACTTTGATCAATTTACTTCTGTTACACAAATTTGAACACAAGATTAACACTGAAGGAATCATTGTTGTGCACGGAATTGCTGGAACTGGGAAAACCACTTTGCTTAGGACTTTATTTTCTGCATACCCTAGCTTAGTTATAGGTTCACCTAGGCCTTGCTACTTAGATAAAGCTAATAAAATTTCACAAGTTTGCCTTTCTTGTTTTCCAAATACCTTGTGTGACATTGTTGACGAGTACCATCTCTTAGAAAGTTTTCCTGAACCAAAACTAGCCATTTTTGGTGACCCCTGTCAGTGCACTTACATTGAAAGGTTGAGAACACCCAACTACACATCCTTCAGAACACACCGATTTGGCAAATCCACTGCTGCTCTACTAAACAAGTTATTTGATCTTAACATTGAGTCAGTCAAAGCACAAGACGACACAGTGGAATACTTTGATCCTTTCGCAGTGGACCCCTCTGAACACATTTCTGCATCAGAAAAAGAAGTTTTGGAATTTGTAGGTGATCAAGTTGAGACTACAAGCTCTGAAGAACTAGCTGGTCTCGAGTTTAGTGAAGTTACTTTCTACTGTACCACACTTGCTGGTGCTGTTCAAGAAAATCCTGCCAAAACTTTCATTTCACTAACTAGACACACTTCAAAGCTCACAATTGGTGAACTAAATGCCAGGTCTGACTCCTAGATGCCAGGTCTGACTCCTAGAGCTGACCTTACTGACACGTATAAAATCATTGCTATAGCCCTTCTACTGTCAGCTTGCATTTACTTCCAAAACAGTCATTATCAACCAGTTGCAGGTGATAACTTGCACAGACTACCCTTCGGTGGCCAGTATCAAGACGGAACTAAGAAGATTTCTTACTTTCCGCAGCAACAATCCTACTTTCACTCAGGAAACAAGCTTAATGTCCTCATACTTATCTTCATTCTTACACTGGGTATTGTCCTCACCAATAAATTTAGTTTTAGCATTAGCCGTAATACTCACCAGCATCATTGCTACAATACACATTCTGCAACCCAAACAGGTCAATCAGTGCCAGGTCATCATTGAATGTCCTCATACTTATCTTCATTCTTACACTGGGTATTGTCCTCACCAATAAATTTAGTTTTAGCATTAGCCGTAATACTCACCAGCATCATTGCTACAATACACATTCTGCAACCCAAACAGGTCAATCAGTGCCAGGTCATCATTGACGGTGCAGCCATAGTTATAACAAATTGTCCAAACACACCCGAAGTTCTTAAAGCAATCAACTTCTCCCCTTGGAACGGGTTAAGTTTTCCTCAATTGTGAATGGAAAACCAACCTACAGCTTCTAACCCATCAGATGTACCACCAACTGCCGCTCAAGCTGGTGCCCAGAGCCCGGCCGACTTCTCAAATCCTAATACAGCTCCTTCCCTAAGTGATTTGAAGAAAATCAAATACGTGTCAACTGTCACTTCAGTTGCCACGCCTGCTGAAATTGAGGCCCTTGGCAAGATCTTTACTGCCATGGGTTTAGCAGCCAATGAGACCGGACCTGCCATGTGGGACCTCGCTCGTGCTTATGCTGATGTGCAAAGTTCAAAATCTGCACAACTTATAGGTGCCACACCATCCAACCCTGCTTTGTCTAGACGTGCACTTGCTGCACAGTTTGATCGTATCAATATCACACCCAGACAATTCTGCATGTATTTTGCAAAAATTGTTTGGAACATACTGTTAGACAGCAATGTGCCACCTGCCAACTGGGCAAAATTGGGCTATCAGAAAGATACCAAATTTGCTGCTTTTGACTTCTTTGATGGAGTCACAAATCCAGCTAGTCTACAGCCTGCAGATGGCCTAATCAGGCAGCCCAATGAAAAAGAGCTTGCTGCCCACTCGGTTGCTAAATATGGCGCCCTTGCCCGCCAGAAAATATCCACTGGTAACTACATCACCACCCTTGGTGAAGTTACACGTGGTCACATGGGCGGCGCCAACACTATGTACGCAATTGATGCACCTCCTGAACTTTAA

>PS5_P1_consensus_sequence

ATGGAAAGATCAACTTTGATCAATTTACTTCTGTTACACAAATTTGAACACAAGATTAACACTGAAGGAATCATTGTTGTGCACGGAATTGCTGGAACTGGGAAAACCACATTGCTTAGGACTTTATTTTCTGCTTACCCTAGCTTAGTTATAGGTTCACCTAGGCCTTGTTACTTAGATAAAGCTAATAAAATTTCACAAGTTTGCCTTTCTTGTTTTCCAAATACCTTGTGTGACATTGTTGACGAGTACCATCTCTTAGAAAGTTTTCCTGAACCAAAACTAGCCATTTTTGGTGACCCCTGTCAGTGCACTTACATTGAAAGGTTGAGAACACCCAACTACACATCCTTCAGAACACACCGATTTGGCAAATCCACTGCTGCTCTACTAAACAAGTTATTTGATCTTAACATTGAGTCAGTCAAAGCACAAGACGACACAGTAGAATACTTTGATCCTTTCGCAGTGGACCCCTCTGAACACATTTCTGCTTCAGAAAAAGAAGTTTTGGAATTTGTAGGTGATCAAGTTGAGACTACAAGCTCTGAAGAACTAGCTGGTCTCGAGTTTAGTGAAGTTACTTTCTACTGTACCACACTTGCTGGTGCTGTTCAAGAAAATCCTGCCAAAACCTTCATTTCACTCACTAGACACACTTCAAAGCTCACAATTGGTGAACTAAATGCCAGGTCTGACTCCTAGATGCCAGGTCTGACTCCTAGAGCTGATCTTACTGACACGTATAAAATCATTGCTATAGCCTTTCTACTGTCAGCTTGCATTTACTTCCAAAACAGTCATTATCAACCAGTTGCAGGTGATAATTTGCACAGACTACCCTTTGGTGGTCAGTATCAAGACGGAACTAAGAAGATCTCTTACTTTCCGCAGCAACAATCCTACTTTCACTCAGGAAACAAGCTTAATGTCCTCATACTTATCTTCATTCTTACACTGGGTATTGTCCTCACCAATAAATTTAGTTTTAGCATTAGCCGTAATACTCACCAGCATCATTGCTACAACACACATTCTGCAACCCAAACAGGTCAATCAGTGCCAGGTCATCATTGAATGTCCTCATACTTATCTTCATTCTTACACTGGGTATTGTCCTCACCAATAAATTTAGTTTTAGCATTAGCCGTAATACTCACCAGCATCATTGCTACAACACACATTCTGCAACCCAAACAGGTCAATCAGTGCCAGGTCATCATTGATGGTGCAGCCATAGTCATAACAAATTGTCCAAACACACCCGAAGTTCTTAAAGCAATCAACTTCTCCCCTTGGAACGGGTTAAGTTTTCCTCAATTGTGAATGGAAAACCAACCTACAGCTTCTAACCCATCAGATGTACCACCAACTGCTGCTCAAGCTGGTGCCCAGAGCCCAGCCGACTTCTCAAATCCTAATACAGCTCCTTCCCTAAGTGATTTGAAGAAGATCAAATACGTGTCAACTGTCACTTCAGTTGCCACGCCTGCTGAAATTGAGGCCCTTGGCAAGATCTTTACTGCCATGGGTTTAGCAGCCAATGAGACCGGACCTGCCATGTGGGACCTCGCTCGTGCTTATGCTGATGTGCAAAGTTCAAAATCTGCACAACTTATAGGTGCCACACCATCCAACCCTGCTTTGTCTAGACGTGCACTTGCTGCACAGTTTGATCGTATCAATATCACACCCAGACAATTCTGCATGTATTTTGCAAAAATTGTTTGGAACATACTGTTAGACAGCAATGTGCCACCTGCCAACTGGGCAAAATTGGGCTATCAGGAAGATACCAAGTTTGCTGCTTTTGACTTCTTTGATGGAGTCACAAATCCAGCTAGTCTACAGCCTGCAGATGGCCTAATCAGGCAGCCCAATGAAAAAGAGCTTGCTGCTCACTCGGTTGCTAAATATGGTGCCCTTGCCCGCCAGAAAATATCCACTGGTAACTACATCACCACCCTTGGTGAAGTTACACGTGGTCACATGGGCGGCGCCAACACTATGTACGCAATTGATGCACCTCCTGAACTTTAA

>PS5_P8_c14

ATGGAAAGATCAACTTTGATCAATTTACTTCTGTTACACAAATTTGAACACAAGATTAACACTGAAGGAATCATTGTTGTGCACGGAATTGCTGGAACTGGGAAAACCACATTGCTTAGGACTTTATTTTCTGCTTACCCTAGCTTAGTTATAGGTTCACCTAGGCCTTGTTACTTAGATAAAGCTAATAAAATTTCACAAGTTTGCCTTTCTTGTTTTTCAAATACCTTGTGTGACATTGTTGACGAGTACCATCTCTTAGAAAGTTTTCCTGAACCAAAACTAGCCATTTTTGGTGACCCCTGTCAGTGCACTTACATTGAAAGGTTGAGAACACCCAACTACACATCCTTCAGAACACACCGATTTGGCAAATCCACTGCTGCTCTACTAAACAAGTTATTTGATCTTAACATTGAGTCAGTCAAAGCACAAGACGACACAGTAGAATACTTTGATCCTTTCGCAGTGGACCCCTCTGAACACATTTCTGCTTCAGAAAAAGAAGTTTTGGAATTTGTAGGTGATCAAGTTGAGACTACAAGCTCTGAAGAACTAGCTGGTCTCGAGTTTAGTGAAGTTACTTTCTACTGTACCACACTTGCTGGTGCTGTTCAAGAAAATCCTGCCAAAACCTTCATTTCACTCACTAGACACACTTCAAAGCTCACAATTGGTGAACTAAATGCCAGGTCTGACTCCTAGATGCCAGGTCTGACTCCTAGAGCTGATCTTACTGACACGTATAAAATCATTGCTATAGCCTTTCTACTGTCAGCTTGCATTTACTTCCAAAACAGTCATTATCAACCAGTTGCAGGTGATAATTTGCACAGACTACCCTTTGGTGGTCAGTATCAAGACGGAACTAAGAAGATCTCTTACTTTCCGCAGCAACAATCCTACTTTCACTCAGGAAACAAGCTTAATGTCCTCATACTTATCTTCATTCTTACACTGGGTATTGTCCTCACCAATAAATTTAGTTTTAGCATTAGCCGCAATACTCACCAGCATCATTGCTACAACACACATTCTGCAACCCAAACAGGTCAATCAGTGCCAGGTCATCATTGAATGTCCTCATACTTATCTTCATTCTTACACTGGGTATTGTCCTCACCAATAAATTTAGTTTTAGCATTAGCCGCAATACTCACCAGCATCATTGCTACAACACACATTCTGCAACCCAAACAGGTCAATCAGTGCCAGGTCATCATTGATGGTGCAGCCATAGTCATAACAAATTGTCCAAACACACCCGAAGTTCTTAAAGCAATCAACTTCTCCCCTTGGAACGGGTTAAGTTTTCCTCAATTGTGAATGGAAAACCAACCTACAGCTTCTAACCCATCAGATGTACCACCAACTGCTGCTCAAGCTGGTGCCCAGAGCCCAGCCGACTTCTCAAATCCTAATACAGCTCCTTCCCTAAGTGATTTGAAGAAGATCAAATACGTGTCAACTGTCACTTCAGTTGCCACGCCTGCTGAAATTGAGGCCCTTGGCAAGATCTTTACTGCCATGGGTTTAGCAGCCAATGAGACCGGACCTGCCATGTGGGACCTCGCTCGTGCTTATGCTGATGTGCAAAGTTCAAAATCTGCACAACTTATAGGTGCCACACCATCCAACCCTGCTTTGTCTAGACGTGCACTTGCTGCACAGTTTGATCGTATCAATATCACACCCAGACAATTCTGCATGTATTTTGCAAAAATTGTTTGGAACATACTGTTAGACAGCAATGTGCCACCTGCCAACTGGGCAAAATTGGGCTATCAGGAAGATACCAAGTTTGCTGCTTTTGACTTCTTTGATGGAGTCACAAATCCAGCTAGTCTACAGCCTGCAGATGGCCTAATCAGGCAGCCCAATGAAAAAGAGCTTGCTGCTCACTCGGTTGCTAAATATGGTGCCCTTGCCCGCCAGAAAATATCCACTGGTAACTACATCACCACCCTTGGTGAAGTTACACGTGGTCACATGGGCGGCGCCAACACTATGTACGCAATTGATGCACCTCCTGAACTTTAA

>PS5_P8_c13

ATGGAAAGATCAACTTTGATCAATTTACTTCTGTTACACAAATTTGAACACAAGATTAACACTGAAGGAATCATTGTTGTGCACGGAATTGCTGGAACTGGGAAAACCACATTGCTTAGGACTTTATTTTCTGCTTACCCTAGCTTAGTTATAGGTTCACCTAGGCCTTGTTACTTAGATAAAGCTAATAAAATTTCACAAGTTTGCCTTTCTTGTTTTCCAAATACCTTGTGTGACATTGTTGACGAGTACCATCTCTTAGAAAGTTTTCCTGAACCAAAACTAGCCATTTTTGGTGACCCCTGTCAGTGCACTTACATTGAAAGGTTGAGAACACCCAACTACACATCCTTCAGAACACACCGATTTGGCAAATCCACTGCTGCTCTACTAAACAAGTTATTTGATCTTAACATTGAGTCAGTCAAAGCACAAGACGACACAGTAGAATACTTTGATCCTTTCGCAGTGGACCCCTCTGAACACATTTCTGCTTCAGAAAAAGAAGTTTTGGAATTTGTAGGTGATCAAGTTGAGACTACAAGCTCTGAAGAACTAGCTGGTCTCGAGTTTAGTGAAGTTACTTTCTACTGTACCACACTTGCTGGTGCTGTTCAAGAAAATCCTGCCAAAACCTTCATTTCACTCACTAGACACACTTCAAAGCTCACAATTGGTGAACTAAATGCCAGGTCTGACTCCTAGATGCCAGGTCTGACTCCTAGAGCTGATCTTACTGACACGTATAAAATCATTGCTATAGCCTTTCTACTGTCAGCTTGCATTTACTTCCAAAACAGTCATTATCAACCAGTTGCAGGTGATAATTTGCACAGACTACCCTTTGGTGGTCAGTATCAAGACGGAACTAAGAAGATCTCTTACTTTCCGCAGCAACAATCCTACTTTCACTCAGGAAACAAGCTTAATGTCCTCATACTTATCTTCATTCTTACACTGGGTATTGTCCTCACCAATAAATTTAGTTTTAGCATTAGCCGTAATACTCACCAGCATCATTGCTACAACACACATTCTGCAACCCAAACAGGTCAATCAGTGCCAGGTCATCATTGAATGTCCTCATACTTATCTTCATTCTTACACTGGGTATTGTCCTCACCAATAAATTTAGTTTTAGCATTAGCCGTAATACTCACCAGCATCATTGCTACAACACACATTCTGCAACCCAAACAGGTCAATCAGTGCCAGGTCATCATTGATGGTGCAGCCATAGTCATAACAAATTGTCCAAACACACCCGAAGTTCTTAAAGCAATCAACTTCTCCCCTTGGAACGGGTTAAGTTTTCCTCAATTGTGAATGGAAAACCAACCTACAGCTTCTAACCCATCAGATGTACCACCAACTGCTGCTCAAGCTGGTGCCCAGAGCCCAGCCGACTTCTCAAATCCTAATACAGCTCCTTCCCTAAGTGATTTGAAGAAGATCAAATACGTGTCAACTGTCACTTCAGTTGCCACGCCTGCTGAAATTGAGGCCCTTGGCAAGATCTTTACTGCCATGGGTTTAGCAGCCAATGAGACCGGACCTGCCATGTGGGACCTCGCTCGTGCTTATGCTGATGTGCAAAGTTCAAAATCTGCACAACTTATAGGTGCCACACCATCCAACCCTGCTTTGTCTAGACGTGCACTTGCTGCACAGTTTGATCGTATCAATATCACACCCAGACAATTCTGCATGTATTTTGCAAAAATTGTTTGGAACATACTGTTAGACAGCAATGTGCCACCTGCCAACTGGGCAAAATTGGGCTATCAGGAAGATACCAAGTTTGCTGCTTTTGACTTCTTTGATGGAGTCACAAATCCAGCTAGTCTACAGCCTGCAGATGGCCTAATCAGGCAGCCCAATGAAAAAGAGCTTGCTGCTCACTCGGTTGCTAAATATGGTGCCCTTGCCCGCCAGAAAATATCCACTGGTAACTACATCACCACCCTTGGTGAAGTTACACGTGGTCACATGGGCGGCGCCAACACTATGTACGCAATTGATGCACCTCCTGAACTTTAA

>PS5_P8_c12

ATGGAAAGATCAACTTTGATCAATTTACTTCTGTTACACAAATTTGAACACAAGATTAACACTGAAGGAATCATTGTTGTGCACGGAATTGCTGGAACTGGGAAAACCACATTGCTTAGGACTTTATTTTCTGCTTACCCTAGCTTAGTTATAGGTTCACCTAGGCCTTGTTACTTAGATAAAGCTAATAAAATTTCACAAGTTTGCCTTTCTTGTTTTCCAAATACCTTGTGTGACATTGTTGACGAGTACCATCTCTTAGAAAGTTTTCCTGAACCAAAACTAGCCATTTTTGGTGACCCCTGTCAGTGCACTTACATTGAAAGGTTGAGAACACCCAACTACACATCCTTCAGAACACACCGATTTGGCAAATCCACTGCTGCTCTACTAAACAAGTTATTTGATCTTAACATTGAGTCAGTCAAAGCACAAGACGACACAGTAGAATACTTTGATCCTTTCGCAGTGGACCCCTCTGAACACATTTCTGCTTCAGAAAAAGAAGTTTTGGAATTTGTAGGTGATCAAGTTGAGACTACAAGCTCTGAAGAACTAGCTGGTCTCGAGTTTAGTGAAGTTACTTTCTACTGTACCACACTTGCTGGTGCTGTTCAAGAAAATCCTGCCAAAACCTTCATTTCACTCACTAGACACACTTCAAAGCTCACAATTGGTGAACTAAATGCCAGGTCTGACTCCTAGATGCCAGGTCTGACTCCTAGAGCTGATCTTACTGACACGTATAAAATCATTGCTATAGCCTTTCTACTGTCAGCTTGCATTTACTTCCAAAACAGTCATTATCAACCAGTTGCAGGTGATAATTTGCACAGACTACCCTTTGGTGGTCAGTATCAAGACGGAACTAAGAAGATCTCTTACTTTCCGCAGCAACAATCCTACTTTCACTCAGGAAACAAGCTTAATGTCCTCATACTTATCTTCATTCTTACACTGGGTATTGTCCTCACCAATAAATTTAGTTTTAGCATTAGCCGTAATACTCACCAGCATCATTGCTACAACACACATTCTGCAACCCAAACAGGTCAATCAGTGCCAGGTCATCATTGAATGTCCTCATACTTATCTTCATTCTTACACTGGGTATTGTCCTCACCAATAAATTTAGTTTTAGCATTAGCCGTAATACTCACCAGCATCATTGCTACAACACACATTCTGCAACCCAAACAGGTCAATCAGTGCCAGGTCATCATTGATGGTGCAGCCATAGTCATAACAAATTGTCCAAACACACCCGAAGTTCTTAAAGCAATCAACTTCTCCCCTTGGAACGGGTTAAGTTTTCCTCAATTGTGAATGGAAAACCAACCTACAGCTTCTAACCCATCAGATGTACCACCAACTGCTGCTCAAGCTGGTGCCCAGAGCCCAGCCGACTTCTCAAATCCTAATACAGCTCCTTCCCTAAGTGATTTGAAGAAGATCAAATACGTGTCAACTGTCACTTCAGTTGCCACGCCTGCTGAAATTGAGGCCCTTGGCAAGATCTTTACTGCCATGGGTTTAGCAGCCAATGAGACCGGACCTGCCATGTGGGACCTCGCTCGTGCTTATGCTGATGTGCAAAGTTCAAAATCTGCACAACTTATAGGTGCCACACCATCCAACCCTGCTTTGTCTAGACGTGCACTTGCTGCACAGTTTGATCGTATCAATATCACACCCAGACAATTCTGCATGTATTTTGCAAAAATTGTTTGGAACATACTGTTAGACAGCAATGTGCCACCTGCCAACTGGGCAAAATTGGGCTATCAGGAAGATACCAAGTTTGCTGCTTTTGACTTCTTTGATGGAGTCACAAATCCAGCTAGTCTACAGCCTGCAGATGGCCTAATCAGGCAGCCCAATGAAAAAGAGCTTGCTGCTCACTCGGTTGCTAAATATGGTGCCCTTGCCCGCCAGAAAATATCCACTGGTAACTACATCACCACCCTTGGTGAAGTTACACGTGGTCACATGGGCGGCGCCAACACTATGTACGCAATTGATGCACCTCCTGAACTTTAA

>PS5_P8_c11

ATGGAAAGATCAACTTTGATCAATTTACTTCTGTTACACAAATTTGAACACAAGATTAACACTGAAGGAATCATTGTTGTGCACGGAATTGCTGGAACTGGGAAAACCACATTGCTTAGGACTTTATTTTCTGCTTACCCTAGCTTAGTTATAGGTTCACCTAGGCCTTGTTACTTAGATAAAGCTAATAAAATTTCACAAGTTTGCCTTTCTTGTTTTCCAAATACCTTGTGTGACATTGTTGACGAGTACCATCTCTTAGAAAGTTTTCCTGAACCAAAACTAGCCATTTTTGGTGACCCCTGTCAGTGCACTTACATTGAAAGGTTGAGAACACCCAACTACACATCCTTCAGAACACACCGATTTGGCAAATCCACTGCTGCTCTACTAAACAAGTTATTTGATCTTAACATTGAGTCAGTCAAAGCACAAGACGACACAGTAGAATACTTTGATCCTTTCGCAGTGGACCCCTCTGAACACATTTCTGCTTCAGAAAAAGAAGTTTTGGAATTTGTAGGTGATCAAGTTGAGACTACAAGCTCTGAAGAACTAGCTGGTCTCGAGTTTAGTGAAGTTACTTTCTACTGTACCACACTTGCTGGTGCTGTTCAAGAAAATCCTGCCAAAACCTTCATTTCACTCACTAGACACACTTCAAAGCTCACAATTGGTGAACTAAATGCCAGGTCTGACTCCTAGATGCCAGGTCTGACTCCTAGAGCTGATCTTACTGACACGTATAAAATCATTGCTATAGCCTTTCTACTGTCAGCTTGCATTTACTTCCAAAACAGTCATTATCAACCAGTTGCAGGTGATAATTTGCACAGACTACCCTTTGGTGGTCAGTATCAAGACGGAACTAAGAAGATCTCTTACTTTCCGCAGCAACAATCCTACTTTCACTCAGGAAACAAGCTTAATGTCCTCATACTTATCTTCATTCTTACACTGGGTATTGTCCTCACCAATAAATTTAGTTTTAGCATTAGCCGTAATACTCACCAGCATCATTGCTACAACACACATTCTGCAACCCAAACAGGTCAATCAGTGCCAGGTCATCATTGAATGTCCTCATACTTATCTTCATTCTTACACTGGGTATTGTCCTCACCAATAAATTTAGTTTTAGCATTAGCCGTAATACTCACCAGCATCATTGCTACAACACACATTCTGCAACCCAAACAGGTCAATCAGTGCCAGGTCATCATTGATGGTGCAGCCATAGTCATAACAAATTGTCCAAACACACCCGAAGTTCTTAAAGCAATCAACTTCTCCCCTTGGAACGGGTTAAGTTTTCCTCAATTGTGAATGGAAAACCAACCTACAGCTTCTAACCCATCAGATGCACCACCAACTGCTGCTCAAGCTGGTGCCCAGAGCCCAGCCGACTTCTCAAATCCTAATACAGCTCCTTCCCTAAGTGATTTGAAGAAGATCAAATACGTGTCAACTGTCACTTCAGTTGCCACGCCTGCTGAAATTGAGGCCCTTGGCAAGATCTTTACTGCCATGGGTTTAGCAGCCAATGAGACCGGACCTGCCATGTGGGACCTCGCTCGTGCTTATGCTGATGTGCAAAGTTCAAAATCTGCACAACTTATAGGTGCCACACCATCCAACCCTGCTTTGTCTAGACGTGCACTTGCTGCACAGTTTGATCGTATCAATATCACACCCAGACAATTCTGCATGTATTTTGCAAAAATTGTTTGGAACATACTGTTAGACAGCAATGTGCCACCTGCCAACTGGGCAAAATTGGGCTATCAGGAAGATACCAAGTTTGCTGCTTTTGACTTCTTTGATGGAGTCACAAATCCAGCTAGTCTACAGCCTGCAGATGGCCTAATCAGGCAGCCCAATGAAAAAGAGCTTGCTGCTCACTCGGTTGCTAAATATGGTGCCCTTGCCCGCCAGAAAATATCCACTGGTAACTACATCACCACCCTTGGTGAAGTTACACGTGGTCACATGGGCGGCGCCAACACTATGTACGCAATTGATGCACCTCCTGAACTTTAA

>PS5_P8_c10

ATGGAAAGATCAACTTTGATCAATTTACTTCTGTTACACAAATTTGAACACAAGATTAACACTGAAGGAATCATTGTTGTGCACGGAATTGCTGGAACTGGGAAAACCACATTGCTTAGGACTTTATTTTCTGCTTACCCTAGCTTAGTTATAGGTTCACCTAGGCCTTGTTACTTAGATAAAGCTAATAAAATTTCACAAGTTTGCCTTTCTTGTTTTCCAAATACCTTGTGTGACATTGTTGACGAGTACCATCTCTTAGAAAGTTTTCCTGAACCAAAACTAGCCATTTTTGGTGACCCCTGTCAGTGCACTTACATTGAAAGGTTGAGAACACCCAACTACACATCCTTCAGAACACACCGATTTGGCAAATCCACTGCTGCTCTACTAAACAAGTTATTTGATCTTAACATTGAGTCAGTCAAAGCACAAGACGACACAGTAGAATACTTTGATCCTTTCGCAGTGGACCCCTCTGAACACATTTCTGCTTCAGAAAAAGAAGTTTTGGAATTTGTAGGTGATCAAGTTGAGACTACAAGCTCTGAAGAACTAGCTGGTCTCGAGTTTAGTGAAGTTACTTTCTACTGTACCACACTTGCTGGTGCTGTTCAAGAAAATCCTGCCAAAACCTTCATTTCACTCACTAGACACACTTCAAAGCTCACAATTGGTGAACTAAATGCCAGGTCTGACTCCTAGATGCCAGGTCTGACTCCTAGAGCTGATCTTACTGACACGTATAAAATCATTGCTATAGCCTTTCTACTGTCAGCTTGCATTTACTTCCAAAACAGTCATTATCAACCAGTTGCAGGTGATAATTTGCACAGACTACCCTTTGGTGGTCAGTATCAAGACGGAACTAAGAAGATCTCTTACTTTCCGCAGCAACAATCCTACTTTCACTCAGGAAACAAGCTTAATGTCCTCATACTTATCTTCATTCTTACACTGGGTATTGTCCTCACCAATAAATTTAGTTTTAGCATTAGCCGTAATACTCACCAGCATCATTGCTACAACACACATTCTGCAACCCAAACAGGTCAATCAGTGCCAGGTCATCATTGAATGTCCTCATACTTATCTTCATTCTTACACTGGGTATTGTCCTCACCAATAAATTTAGTTTTAGCATTAGCCGTAATACTCACCAGCATCATTGCTACAACACACATTCTGCAACCCAAACAGGTCAATCAGTGCCAGGTCATCATTGATGGTGCAGCCATAGTCATAACAAATTGTCCAAACACACCCGAAGTTCTTAAAGCAATCAACTTCTCCCCTTGGAACGGGTTAAGTTTTCCTCAATTGTGAATGGAAAACCAACCTACAGCTTCTAACCCATCAGATGTACCACCAACTGCTGCTCAAGCTGGTGCCCAGAGCCCAGCCGACTTCTCAAATCCTAATACAGCTCCTTCCCTAAGTGATTTGAAGAAGATCAAATACGTGTCAACTGTCACTTCAGTTGCCACGCCTGCTGAAATTGAGGCCCTTGGCAAGATCTTTACTGCCATGGGTTTAGCAGCCAATGAGACCGGACCTGCCATGTGGGACCTCGCTCGTGCTTATGCTGATGTGCAAAGTTCAAAATCTGCACAACTTATAGGTGCCACACCATCCAACCCTGCTTTGTCTAGACGTGCACTTGCTGCACAGTTTGATCGTATCAATATCACACCCAGACAATTCTGCATGTATTTTGCAAAAATTGTTTGGAACATACTGTTAGACAGCAATGTGCCACCTGCCAACTGGGCAAAATTGGGCTATCAGGAAGATACCAAGTTTGCTGCTTTTGACTTCTTTGATGGAGTCACAAATCCAGCTAGTCTACAGCCTGCAGATGGCCTAATCAGGCAGCCCAATGAAAAAGAGCTTGCTGCTCACTCGGTTGCTAAATATGGTGCCCTTGCCCGCCAGAAAATATCCACTGGTAACTACATCACCACCCTTGGTGAAGTTACACGTGGTCACATGGGCGGCGCCAACACTATGTACGCAATTGATGCACCTCCTGAACTTTAA

>PS5_P8_c9

ATGGAAAGATCAACTTTGATCAATTTACTTCTGTTACACAAATTTGAACACAAGATTAACACTGAAGGAATCATTGTTGTGCACGGAATTGCTGGAACTGGGAAAACCACATTGCTTAGGACTTTATTTTCTGCTTACCCTAGCTTAGTTATAGGTTCACCTAGGCCTTGTTACTTAGATAAAGCTAATAAAATTTCACAAGTTTGCCTTTCTTGTTTTCCAAATACCTTGTGTGACATTGTTGACGAGTACCATCTCTTAGAAAGTTTTCCTGAACCAAAACTAGCCATTTTTGGTGACCCCTGTCAGTGCACTTACATTGAAAGGTTGAGAACACCCAACTACACATCCTTCAGAACACACCGATTTGGCAAATCCACTGCTGCTCTACTAAACAAGTTATTTGATCTTAACATTGAGTCAGTCAAAGCACAAGACGACACAGTAGAATACTTTGATCCTTTCGCAGTGGACCCCTCTGAACACATTTCTGCTTCAGAAAAAGAAGTTTTGGAATTTGTAGGTGATCAAGTTGAGACTACAAGCTCTGAAGAACTAGCTGGTCTCGAGTTTAGTGAAGTTACTTTCTACTGTACCACACTTGCTGGTGCTGTTCAAGAAAATCCTGCCAAAACCTTCATTTCACTCACTAGACACACTTCAAAGCTCACAATTGGTGAACTAAATGCCAGGTCTGACTCCTAGATGCCAGGTCTGACTCCTAGAGCTGATCTTACTGACACGTATAAAATCATTGCTATAGCCTTTCTACTGTCAGCTTGCATTTACTTCCAAAACAGTCATTATCAACCAGTTGCAGGTGATAATTTGCACAGACTACCCTTTGGTGGTCAGTATCAAGACGGAACTAAGAAGATCTCTTACTTTCCGCAGCAACAATCCTACTTTCACTCAGGAAACAAGCTTAATGTCCTCATACTTATCTTCATTCTTACACTGGGTATTGTCCTCACCAATAAATTTAGTTTTAGCATTAGCCGTAATACTCACCAGCATCATTGCTACAACACACATTCTGCAACCCAAACAGGTCAATCAGTGCCAGGTCATCATTGAATGTCCTCATACTTATCTTCATTCTTACACTGGGTATTGTCCTCACCAATAAATTTAGTTTTAGCATTAGCCGTAATACTCACCAGCATCATTGCTACAACACACATTCTGCAACCCAAACAGGTCAATCAGTGCCAGGTCATCATTGATGGTGCAGCCATAGTCATAACAAATTGTCCAAACACACCCGAAGTTCTTAAAGCAATCAACTTCTCCCCTTGGAACGGGTTAAGTTTTCCTCAATTGTGAATGGAAAACCAACCTACAGCTTCTAACCCATCAGATGTACCACCAACTGCTGCTCAAGCTGGTGCCCAGAGCCCAGCCGACTTCTCAAATCCTAATACAGCTCCTTCCCTAAGTGATTTGAAGAAGATCAAATACGTGTCAACTGTCACTTCAGTTGCCACGCCTGCTGAAATTGAGGCCCTTGGCAAGATCTTTACTGCCATGGGTTTAGCAGCCAATGAGACCGGACCTGCCATGTGGGACCTCGCTCGTGCTTATGCTGATGTGCAAAGTTCAAAATCTGCACAACTTATAGGTGCCACACCATCCAACCCTGCTTTGTCTAGACGTGCACTTGCTGCACAGTTTGATCGTATCAATATCACACCCAGACAATTCTGCATGTATTTTGCAAAAATTGTTTGGAACATACTGTTAGACAGCAATGTGCCACCTGCCAACTGGGCAAAATTGGGCTATCAGGAAGATACCAAGTTTGCTGCTTTTGACTTCTTTGATGGAGTCACAAATCCAGCTAGTCTACAGCCTGCAGATGGCCTAATCAGGCAGCCCAATGAAAAAGAGCTTGCCGCTCACTCGGTTGCTAAATATGGTGCCCTTGCCCGCCAGAAAATATCCACTGGTAACTACATCACCACCCTTGGTGAAGTTACACGTGGTCACATGGGCGGCGCCAACACTATGTACGCAATTGATGCACCTCCTGAACTTTAA

>PS5_P8_c8

ATGGAAAGATCAACTTTGATCAATTTACTTCTGTTACACAAATTTGAACACAAGATTAACACTGAAGGAATCATTGTTGTGCACGGAATTGCTGGAACTGGGAAAACCACATTGCTTAGGACTTTATTTTCTGCTTACCCTAGCTTAGTTATAGGTTCACCTAGGCCTTGTTACTTAGATAAAGCTAATAAAATTTCACAAGTTTGCCTTTCTTGTTTTCCAAATACCTTGTGTGACATTGTTGACGAGTACCATCTCTTAGAAAGTTTTCCTGAACCAAAACTAGCCATTTTTGGTGACCCCTGTCAGTGCACTTACATTGAAAGGTTGAGAACACCCAACTACACATCCTTCAGAACACACCGATTTGGCAAATCCACTGCTGCTCTACTAAACAAGTTATTTGATCTTAACATTGAGTCAGTCAAAGCACAAGACGACACAGTAGAATACTTTGATCCTTTCGCAGTGGACCCCTCTGAACACATTTCTGCTTCAGAAAAAGAAGTTTTGGAATTTGTAGGTGATCAAGTTGAGACTACAAGCTCTGAAGAACTAGCTGGTCTCGAGTTTAGTGAAGTTACTTTCTACTGTACCACACTTGCTGGTGCTGTTCAAGAAAATCCTGCCAAAACCTTCATTTCACTCACTAGACACACTTCAAAGCTCACAATTGGTGAACTAAATGCCAGGTCTGACTCCTAGATGCCAGGTCTGACTCCTAGAGCTGATCTTACTGACACGTATAAAATCATTGCTATAGCCTTTCTACTGTCAGCTTGCATTTACTTCCAAAACAGTCATTATCAACCAGTTGCAGGTGATAATTTGCACAGACTACCCTTTGGTGGTCAGTATCAAGACGGAACTAAGAAGATCTCTTACTTTCCGCAGCAACAATCCTACTTTCACTCAGGAAACAAGCTTAATGTCCTCATACTTATCTTCATTCTTACACTGGGTATTGTCCTCACCAATAAATTTAGTTTTAGCATTAGCCGTAATACTCACCAGCATCATTGCTACAACACACATTCTGCAACCCAAACAGGTCAATCAGTGCCAGGTCATCATTGAATGTCCTCATACTTATCTTCATTCTTACACTGGGTATTGTCCTCACCAATAAATTTAGTTTTAGCATTAGCCGTAATACTCACCAGCATCATTGCTACAACACACATTCTGCAACCCAAACAGGTCAATCAGTGCCAGGTCATCATTGATGGTGCAGCCATAGTCATAACAAATTGTCCAAACACACCCGAAGTTCTTAAAGCAATCAACTTCTCCCCTTGGAACGGGTTAAGTTTTCCTCAATTGTGAATGGAAAACCAACCTACAGCTTCTAACCCATCAGATGTACCACCAACTGCTGCTCAAGCTGGTGCCCAGAGCCCAGCCGACTTCTCAAATCCTAATACAGCTCCTTCCCTAAGTGATTTGAAGAAGATCAAATACGTGTCAACTGTCACTTCAGTTGCCACGCCTGCTGAAATTGAGGCCCTTGGCAAGATCTTTACTGCCATGGGTTTAGCAGCCAATGAGACCGGACCTGCCATGTGGGACCTCGCTCGTGCTTATGCTGATGTGCAAAGTTCAAAATCTGCACAACTTATAGGTGCCACACCATCCAACCCTGCTTTGTCTAGACGTGCACTTGCTGCACAGTTTGATCGTATCAATATCACACCCAGACAATTCTGCATGTATTTTGCAAAAATTGTTTGGAACATACTGTTAGACAGCAATGTGCCACCTGCCAACTGGGCAAAATTGGGCTATCAGGAAGATACCAAGTTTGCTGCTTTTGACTTCTTTGATGGAGTCACAAATCCAGCTAGTCTACAGCCTGCAGATGGCCTAATCAGGCAGCCCAATGAAAAAGAGCTTGCTGCTCACTCGGTTGCTAAATATGGTGCCCTTGCCCGCCAGAAAATATCCACTGGTAACTACATCACCACCCTTGGTGAAGTTACACGTGGTCACATGGGCGGCGCCAACACTATGTACGCAATTGATGCACCTCCTGAACTTTAA

>PS5_P8_c7

ATGGAAGGATCAACTTTGATCAATTTACTTCTGTTACACAAATTTGAACACAAGATTAACACTGAAGGAATCATTGTTGTGCACGGAATTGCTGGAACTGGGAAAACCACATTGCTTAGGACTTTATTTTCTGCTTACCCTAGCTTAGTTATAGGTTCACCTAGGCCTTGTTACTTAGATAAAGCTAATAAAATTTCACAAGTTTGCCTTTCTTGTTTTCCAAATACCTTGTGTGACATTGTTGACGAGTACCATCTCTTAGAAAGTTTTCCTGAACCAAAACTAGCCATTTTTGGTGACCCCTGTCAGTGCACTTACATTGAAAGGTTGAGAACACCCAACTACACATCCTTCAGAACACACCGATTTGGCAAATCCACTGCTGCTCTACTAAACAAGTTATTTGATCTTAACATTGAGTCAGTCAAAGCACAAGACGACACAGTAGAATACTTTGATCCTTTCGCAGTGGACCCCTCTGAACACATTTCTGCTTCAGAAAAAGAAGTTTTGGAATTTGTAGGTGATCAAGTTGAGACTACAAGCTCTGAAGAACTAGCTGGTCTCGAGTTTAGTGAAGTTACTTTCTACTGTACCACACTTGCTGGTGCTGTTCAAGAAAATCCTGCCAAAACCTTCATTTCACTCACTAGACACACTTCAAAGCTCACAATTGGTGAACTAAATGCCAGGTCTGACTCCTAGATGCCAGGTCTGACTCCTAGAGCTGATCTTACTGACACGTATAAAATCATTGCTATAGCCTTTCTACTGTCAGCTTGCATTTACTTCCAAAACAGTCATTATCAACCAGTTGCAGGTGATAATTTGCACAGACTACCCTTTGGTGGTCAGTATCAAGACGGAACTAAGAAGATCTCTTACTTTCCGCAGCAACAATCCTACTTTCACTCAGGAAACAAGCTTAATGTCCTCATACTTATCTTCATTCTTACACTGGGTATTGTCCTCACCAATAAATTTAGTTTTAGCATTAGCCGTAATACTCACCAGCATCATTGCTACAACACACATTCTGCAACCCAAACAGGTCAATCAGTGCCAGGTCATCATTGAATGTCCTCATACTTATCTTCATTCTTACACTGGGTATTGTCCTCACCAATAAATTTAGTTTTAGCATTAGCCGTAATACTCACCAGCATCATTGCTACAACACACATTCTGCAACCCAAACAGGTCAATCAGTGCCAGGTCATCATTGATGGTGCAGCCATAGTCATAACAAATTGTCCAAACACACCCGAAGTTCTTAAAGCAATCAACTTCTCCCCTTGGAACGGGTTAAGTTTTCCTCAATTGTGAATGGAAAACCAACCTACAGCTTCTAACCCATCAGATGTACCACCAACTGCTGCTCAAGCTGGTGCCCAGAGCCCAGCCGACTTCTCAAATCCTAATACAGCTCCTTCCCTAAGTGATTTGAAGAAGATCAAATACGTGTCAACTGTCACTTCAGTTGCCACGCCTGCTGAAATTGAGGCCCTTGGCAAGATCTTTACTGCCATGGGTTTAGCAGCCAATGAGACCGGACCTGCCATGTGGGACCTCGCTCGTGCTTATGCTGATGTGCAAAGTTCAAAATCTGCACAACTTATAGGTGCCACACCATCCAACCCTGCTTTGTCTAGACGTGCACTTGCTGCACAGTTTGATCGTATCAATATCACACCCAGACAATTCTGCATGTATTTTGCAAAAATTGTTTGGAACATACTGTTAGACAGCAATGTGCCACCTGCCAACTGGGCAAAATTGGGCTATCAGGAAGATACCAAGTTTGCTGCTTTTGACTTCTTTGATGGAGTCACAAATCCAGCTAGTCTACAGCCTGCAGATGGCCTAATCAGGCAGCCCAATGAAAAAGAGCTTGCTGCTCACTCGGTTGCTAAATATGGTGCCCTTGCCCGCCAGAAAATATCCACTGGTAACTACATCACCACCCTTGGTGAAGTTACACGTGGTCACATGGGCGGCGCCAACACTATGTACGCAATTGATGCACCTCCTGAACTTTAA

>PS5_P8_c6

ATGGAAAGATCAACTTTGATCAATTTACTTCTGTTACACAAATTTGAACACAAGATTAACACTGAAGGAATCATTGTTGTGCACGGAATTGCTGGAACTGGGAAAACCACATTGCTTAGGACTTTATTTTCTGCTTACCCTAGCTTAGTTATAGGTTCACCTAGGCCTTGTTACTTAGATAAAGCTAATAAAATTTCACAAGTTTGCCTTTCTTGTTTTCCAAATACCTTGTGTGACATTGTTGACGAGTACCATCTCTTAGAAAGTTTTCCTGAACCAAAACTAGCCATTTTTGGTGACCCCTGTCAGTGCACTTACATTGAAAGGTTGAGAACACCCAACTACACATCCTTCAGAACACACCGATTTGGCAAATCCACTGCTGCTCTACTAAACAAGTTATTTGATCTTAACATTGAGTCAGTCAAAGCACAAGACGACACAGTAGAATACTTTGATCCTTTCGCAGTGGACCCCTCTGAACACATTTCTGCTTCAGAAAAAGAAGTTTTGGAATTTGTAGGTGATCAAGTTGAGACTACAAGCTCTGAAGAACTAGCTGGTCTCGAGTTTAGTGAAGTTACTTTCTACTGTACCACACTTGCTGGTGCTGTTCAAGAAAATCCTGCCAAAACCTTCATTTCACTCACTAGACACACTTCAAAGCTCACAATTGGTGAACTAAATGCCAGGTCTGACTCCTAGATGCCAGGTCTGACTCCTAGAGCTGATCTTACTGACACGTATAAAATCATTGCTATAGCCTTTCTACTGTCAGCTTGCATTTACTTCCAAAACAGTCATTATCAACCAGTTGCAGGTGATAATTTGCACAGACTACCCTTTGGTGGTCAGTATCAAGACGGAACTAAGAAGATCTCTTACTTTCCGCAGCAACAATCCTACTTTCACTCAGGAAACAAGCTTAATGTCCTCATACTTATCTTCATTCTTACACTGGGTATTGTCCTCACCAATAAATTTAGTTTTAGCATTAGCCGTAATACTCACCAGCATCATTGCTACAACACACATTCTGCAACCCAAACAGGTCAATCAGTGCCAGGTCATCATTGAATGTCCTCATACTTATCTTCATTCTTACACTGGGTATTGTCCTCACCAATAAATTTAGTTTTAGCATTAGCCGTAATACTCACCAGCATCATTGCTACAACACACATTCTGCAACCCAAACAGGTCAATCAGTGCCAGGTCATCATTGATGGTGCAGCCATAGTCATAACAAATTGTCCAAACACACCCGAAGTTCTTAAAGCAATCAACTTCTCCCCTTGGAACGGGTTAAGTTTTCCTCAATTGTGAATGGAAAACCAACCTACAGCTTCTAACCCATCAGATGTACCACCAACTGCTGCTCAAGCTGGTGCCCAGAGCCCAGCCGACTTCTCAAATCCTAATACAGCTCCTTCCCTAAGTGATTTGAAGAAGATCAAATACGTGTCAACTGTCACTTCAGTTGCCACGCCTGCTGAAATTGAGGCCCTTGGCAAGATCTTTACTGCCATGGGTTTAGCAGCCAATGAGACCGGACCTGCCATGTGGGACCTCGCTCGTGCTTATGCTGATGTGCAAAGTTCAAAATCTGCACAACTTATAGGTGCCGCACCATCCAACCCTGCTTTGTCTAGACGTGCACTTGCTGCACAGTTTGATCGTATCAATATCACACCCAGACAATTCTGCATGTATTTTGCAAAAATTGTTTGGAACATACTGTTAGACAGCAATGTGCCACCTGCCAACTGGGCAAAATTGGGCTATCAGGAAGATACCAAGTTTGCTGCTTTTGACTTCTTTGATGGAGTCACAAATCCAGCTAGTCTACAGCCTGCAGATGGCCTAATCAGGCAGCCCAATGAAAAAGAGCTTGCTGCTCACTCGGTTGCTAAATATGGTGCCCTTGCCCGCCAGAAAATATCCACTGGTAACTACATCACCACCCTTGGTGAAGTTACACGTGGTCACATGGGCGGCGCCAACACTATGTACGCAATTGATGCACCTCCTGAACTTTAA

>PS5_P8_c5

ATGGAAAGATCAACTTTGATCAATTTACTTCTGTTACACAAATTTGAACACAAGATTAACACTGAAGGAATCATTGTTGTGCACGGAATTGCTGGAACTGGGAAAACCACATTGCTTAGGACTTTATTTTCTGCTTACCCTAGCTTAGTTATAGGTTCACCTAGGCCTTGTTACTTAGATAAAGCTAATAAAATTTCACAAGTTTGCCTTTCTTGTTTTCCAAATACCTTGTGTGACATTGTTGACGAGTACCATCTCTTAGAAAGTTTTCCTGAACCAAAACTAGCCATTTTTGGTGACCCCTGTCAGTGCACTTACATTGAAAGGTTGAGAACACCCAACTACACATCCTTCAGAACACACCGATTTGGCAAATCCACTGCTGCTCTACTAAACAAGTTATTTGATCTTAACATTGAGTCAGTCAAAGCACAAGACGACACAGTAGAATACTTTGATCCTTTCGCAGTGGACCCCTCTGAACACATTTCTGCTTCAGAAAAAGAAGTTTTGGAATTTGTAGGTGATCAAGTTGAGACTACAAGCTCTGAAGAACTAGCTGGTCTCGAGTTTAGTGAAGTTACTTTCTACTGTACCACACTTGCTGGTGCTGTTCAAGAAAATCCTGCCAAAACCTTCATTTCACTCACTAGACACACTTCAAAGCTCACAATTGGTGAACTAAATGCCAGGTCTGACTCCTAGATGCCAGGTCTGACTCCTAGAGCTGATCTTACTGACACGTATAAAATCATTGCTATAGCCTTTCTACTGTCAGCTTGCATTTACTTCCAAAACAGTCATTATCAACCAGTTGCAGGTGATAATTTGCACAGACTACCCTTTGGTGGTCAGTATCAAGACGGAACTAAGAAGATCTCTTACTTTCCGCAGCAACAATCCTACTTTCACTCAGGAAACAAGCTTAATGTCCTCATACTTATCTTCATTCTTACACTGGGTATTGTCCTCACCAATAAATTTAGTTTTAGCATTAGCCGTAATACTCACCAGCATCATTGCTACAACACACATTCTGCAACCCAAACAGGTCAATCAGTGCCAGGTCATCATTGAATGTCCTCATACTTATCTTCATTCTTACACTGGGTATTGTCCTCACCAATAAATTTAGTTTTAGCATTAGCCGTAATACTCACCAGCATCATTGCTACAACACACATTCTGCAACCCAAACAGGTCAATCAGTGCCAGGTCATCATTGATGGTGCAGCCATAGTCATAACAAATTGTCCAAACACACCCGAAGTTCTTAAAGCAATCAACTTCTCCCCTTGGAACGGGTTAAGTTTTCCTCAATTGTGAATGGAAAACCAACCTACAGCTTCTAACCCATCAGATGTACCACCAACTGCTGCTCAAGCTGGTGCCCAGAGCCCAGCCGACTTCTCAAATCCTAATACAGCTCCTTCCCTAAGTGATTTGAAGAAGATCAAATACGTGTCAACTGTCACTTCAGTTGCCACGCCTGCTGAAATTGAGGCCCTTGGCAAGATCTTTACTGCCATGGGTTTAGCAGCCAATGAGACCGGACCTGCCATGTGGGACCTCGCTCGTGCTTATGCTGATGTGCAAAGTTCAAAATCTGCACAACTTATAGGTGCCACACCATCCAACCCTGCTTTGTCTAGACGTGCACTTGCTGCACAGTTTGATCGTATCAATATCACACCCAGACAATTCTGCATGTATTTTGCAAAAATTGTTTGGAACATACTGTTAGACAGCAATGTGCCACCTGCCAACTGGGCAAAATTGGGCTATCAGGAAGATACCAAGTTTGCTGCTTTTGACTTCTTTGATGGAGTCACAAATCCAGCTAGTCTACAGCCTGCAGATGGCCTAATCAGGCAGCCCAATGAAAAAGAGCTTGCTGCTCACTCGGTTGCTAAATATGGTGCCCTTGCCCGCCAGAAAATATCCACTGGTAACTACATCACCACCCTTGGTGAAGTTACACGTGGTCACATGGGCGGCGCCAACACTATGTACGCAATTGATGCACCTCCTGAACTTTAA

>PS5_P8_c4

ATGGAAAGATCAACTTTGATCAATTTACTTCTGTTACACAAATTTGAACACAAGATTAACACTGAAGGAATCATTGTTGTGCACGGAATTGCTGGAACTGGGAAAACCACATTGCTTAGGACTTTATTTTCTGCTTACCCTAGCTTAGTTATAGGTTCACCTAGGCCTTGTTACTTAGATAAAGCTAATAAAATTTCACAAGTTTGCCTTTCTTGTTTTCCAAATACCTTGTGTGACATTGTTGACGAGTACCATCTCTTAGAAAGTTTTCCTGAACCAAAACTAGCCATTTTTGGTGACCCCTGTCAGTGCACTTACATTGAAAGGTTGAGAACACCCAACTACACATCCTTCAGAACACACCGATTTGGCAAATCCACTGCTGCTCTACTAAACAAGTTATTTGATCTTAACATTGAGTCAGTCAAAGCACAAGACGACACAGTAGAATACTTTGATCCTTTCGCAGTGGACCCCTCTGAACACATTTCTGCTTCAGAAAAAGAAGCTTTGGAATTTGTAGGTGATCAAGTTGAGACTACAAGCTCTGAAGAACTAGCTGGTCTCGAGTTTAGTGAAGTTACTTTCTACTGTACCACACTTGCTGGTGCTGTTCAAGAAAATCCTGCCAAAACCTTCATTTCACTCACTAGACACACTTCAAAGCTCACAATTGGTGAACTAAATGCCAGGTCTGACTCCTAGATGCCAGGTCTGACTCCTAGAGCTGATCTTACTGACACGTATAAAATCATTGCTATAGCCTTTCTACTGTCAGCTTGCATTTACTTCCAAAACAGTCATTATCAACCAGTTGCAGGTGATAATTTGCACAGACTACCCTTTGGTGGTCAGTATCAAGACGGAACTAAGAAGATCTCTTACTTTCCGCAGCAACAATCCTACTTTCACTCAGGAAACAAGCTTAATGTCCTCATACTTATCTTCATTCTTACACTGGGTATTGTCCTCACCAATAAATTTAGTTTTAGCATTAGCCGTAATACTCACCAGCATCATTGCTACAACACACATTCTGCAACCCAAACAGGTCAATCAGTGCCAGGTCATCATTGAATGTCCTCATACTTATCTTCATTCTTACACTGGGTATTGTCCTCACCAATAAATTTAGTTTTAGCATTAGCCGTAATACTCACCAGCATCATTGCTACAACACACATTCTGCAACCCAAACAGGTCAATCAGTGCCAGGTCATCATTGATGGTGCAGCCATAGTCATAACAAATTGTCCAAACACACCCGAAGTTCTTAAAGCAATCAACTTCTCCCCTTGGAACGGGTTAAGTTTTCCTCAATTGTGAATGGAAAACCAACCTACAGCTTCTAACCCATCAGATGTACCACCAACTGCTGCTCAAGCTGGTGCCCAGAGCCCAGCCGACTTCTCAAATCCTAATACAGCTCCTTCCCTAAGTGATTTGAAGAAGATCAAATACGTGTCAACTGTCACTTCAGTTGCCACGCCTGCTGAAATTGAGGCCCTTGGCAAGATCTTTACTGCCATGGGTTTAGCAGCCAATGAGACCGGACCTGCCATGTGGGACCTCGCTCGTGCTTATGCTGATGTGCAAAGTTCAAAATCTGCACAACTTATAGGTGCCACACCATCCAACCCTGCTTTGTCTAGACGTGCACTTGCTGCACAGTTTGATCGTATCAATATCACACCCAGACAATTCTGCATGTATTTTGCAAAAATTGTTTGGAACATACTGTTAGACAGCAATGTGCCACCTGCCAACTGGGCAAAATTGGGCTATCAGGAAGATACCAAGTTTGCTGCTTTTGACTTCTTTGATGGAGTCACAAATCCAGCTAGTCTACAGCCTGCAGATGGCCTAATCAGGCAGCCCAATGAAAAAGAGCTTGCTGCTCACTCGGTTGCTAAATATGGTGCCCTTGCCCGCCAGAAAATATCCACTGGTAACTACATCACCACCCTTGGTGAAGTTACACGTGGTCACATGGGCGGCGCCAACACTATGTACGCAATTGATGCACCTCCTGAACTTTAA

>PS5_P8_c3

ATGGAAAGATCAACTTTGATCAATTTACTTCTGTTACACAAATTTGAACACAAGATTAACACTGAAGGAATCATTGTTGTGCACGGAATTGCTGGAACTGGGAAAACCACATTGCTTAGGACTTTATTTTCTGCTTACCCTAGCTTAGTTATAGGTTCACCTAGGCCTTGTTACTTAGATAAAGCTAATAAAATTTCACAAGTTTGCCTTTCTTGTTTTCCAAATACCTTGTGTGACATTGTTGACGAGTACCATCTCTTAGAAAGTTTTCCTGAACCAAAACTAGCCATTTTTGGTGACCCCTGTCAGTGCACTTACATTGAAAGGTTGAGAACACCCAACTACACATCCTTCAGAACACACCGATTTGGCAAATCCACTGCTGCTCTACTAAACAAGTTATTTGATCTTAACATTGAGTCAGTCAAAGCACAAGACGACACAGTAGAATACTTTGATCCTTTCGCAGTGGACCCCTCTGAACACATTTCTGCTTCAGAAAAAGAAGTTTTGGAATTTGTAGGTGATCAAGTTGAGACTACAAGCTCTGAAGAACTAGCTGGTCTCGAGTTTAGTGAAGTTACTTTCTACTGTACCACACTTGCTGGTGCTGTTCAAGAAAATCCTGCCAAAACCTTCATTTCACTCACTAGACACACTTCAAAGCTCACAATTGGTGAACTAAATGCCAGGTCTGACTCCTAGATGCCAGGTCTGACTCCTAGAGCTGATCTTACTGACACGTATAAAATCATTGCTATAGCCTTTCTACTGTCAGCTTGCATTTACTTCCAAAACAGTCATTATCAACCAGTTGCAGGTGATAATTTGCACAGACTACCCTTTGGTGGTCAGTATCAAGACGGAACTAAGAAGATCTCTTACTTTCCGCAGCAACAATCCTACTTTCACTCAGGAAACAAGCTTAATGTCCTCATACTTATCTTCATTCTTACACTGGGTATTGTCCTCACCAATAAATTTAGTTTTAGCATTAGCCGTAATACTCACCAGCATCATTGCTACAACACACATTCTGCAACCCAAACAGGTCAATCAGTGCCAGGTCATCATTGAATGTCCTCATACTTATCTTCATTCTTACACTGGGTATTGTCCTCACCAATAAATTTAGTTTTAGCATTAGCCGTAATACTCACCAGCATCATTGCTACAACACACATTCTGCAACCCAAACAGGTCAATCAGTGCCAGGTCATCATTGATGGTGCAGCCATAGTCATAACAAATTGTCCAAACACACCCGAAGTTCTTAAAGCAATCAACTTCTCCCCTTGGAACGGGTTAAGTTTTCCTCAATTGTGAATGGAAAACCAACCTACAGCTTCTAACCCATCAGATGTACCACCAACTGCTGCTCAAGCTGGTGCCCAGAGCCCAGCCGACTTCTCAAATCCTAATACAGCTCCTTCCCTAAGTGATTTGAAGAAGATCAAATACGTGTCAACTGTCACTTCAGTTGCCACGCCTGCTGAAATTGAGGCCCTTGGCAAGATCTTTACTGCCATGGGTTTAGCAGCCAATGAGACCGGACCTGCCATGTGGGACCTCGCTCGTGCTTATGCTGATGTGCAAAGTTCAAAATCTGCACAACTTATAGGTGCCACACCATCCAACCCTGCTTTGTCTAGACGTGCACTTGCTGCACAGTTTGATCGTATCAATATCACACCCAGACAATTCTGCATGTATTTTGCAAAAATTGTTTGGAACATACTGTTAGACAGCAATGTGCCACCTGCCAACTGGGCAAAATTGGGCTATCAGGAAGATACCAAGTTTGCTGCTTTTGACTTCTTTGATGGAGTCACAAATCCAGCTAGTCTACAGCCTGCAGATGGCCTAATCAGGCAGCCCAATGAAAAAGAGCTTGCTGCTCACTCGGTTGCTAAATATGGTGCCCTTGCCCGCCAGAAAATATCCACTGGTAACTACATCACCACCCTTGGTGAAGTTACACGTGGTCACATGGGCGGCGCCAACACTATGTACGCAATTGATGCACCTCCTGAACTTTAA

>PS5_P8_c2

ATGGAAAGATCAACTTTGATCAATTTACTTCTGTTACACAAATTTGAACACAAGATTAACACTGAAGGAATCATTGTTGTGCACGGAATTGCTGGAACTGGGAAAACCACATTGCTTAGGACTTTATTTTCTGCTTACCCTAGCTTAGTTATAGGTTCACCTAGGCCTTGTTACTTAGATAAAGCTAATAAAATTTCACAAGTTTGCCTTTCTTGTTTTCCAAATACCTTGTGTGACATTGTTGACGAGTACCATCTCTTAGAAAGTTTTCCTGAACCAAAACTAGCCATTTTTGGTGACCCCTGTCAGTGCACTTACATTGAAAGGTTGAGAACACCCAACTACACATCCTTCAGAACACACCGATTTGGCAAATCCACTGCTGCTCTACTAAACAAGTTATTTGATCTTAACATTGAGTCAGTCAAAGCACAAGACGACACAGTAGAATACTTTGATCCTTTCGCAGTGGACCCCTCTGAACACATTTCTGCTTCAGAAAAAGAAGTTTTGGAATTTGTAGGTGATCAAGTTGAGACTACAAGCTCTGAAGAACTAGCTGGTCTCGAGTTTAGTGAAGTTACTTTCTACTGTACCACACTTGCTGGTGCTGTTCAAGAAAATCCTGCCAAAACCTTCATTTCACTCACTAGACACACTTCAAAGCTCACAATTGGTGAACTAAATGCCAGGTCTGACTCCTAGATGCCAGGTCTGACTCCTAGAGCTGATCTTACTGACACGTATAAAATCATTGCTATAGCCTTTCTACTGTCAGCTTGCATTTACTTCCAAAACAGTCATTATCAACCAGTTGCAGGTGATAATTTGCACAGACTACCCTTTGGTGGTCAGTATCAAGACGGAACTAAGAAGATCTCTTACTTTCCGCAGCAACAATCCTACTTTCACTCAGGAAACAAGCTTAATGTCCTCATACTTATCTTCATTCTTACACTGGGTATTGTCCTCACCAATAAATTTAGTTTTAGCATTAGCCGTAATACTCACCAGCATCATTGCTACAACACACATTCTGCAACCCAAACAGGTCAATCAGTGCCAGGTCATCATTGAATGTCCTCATACTTATCTTCATTCTTACACTGGGTATTGTCCTCACCAATAAATTTAGTTTTAGCATTAGCCGTAATACTCACCAGCATCATTGCTACAACACACATTCTGCAACCCAAACAGGTCAATCAGTGCCAGGTCATCATTGATGGTGCAGCCATAGTCATAACAAATTGTCCAAACACACCCGAAGTTCTTAAAGCAATCAACTTCTCCCCTTGGAACGGGTTAAGTTTTCCTCAATTGTGAATGGAAAACCAACCTACAGCTTCTAACCCATCAGATGTACCACCAACTGCTGCTCAAGCTGGTGCCCAGAGCCCAGCCGACTTCTCAAATCCTAATACAGCTCCTTCCCTAAGTGATTTGAAGAAGATCAAATACGTGTCAACTGTCACTTCAGTTGCCACGCCTGCTGAAATTGAGGCCCTTGGCAAGATCTTTACTGCCATGGGTTTAGCAGCCAATGAGACCGGACCTGCCATGTGGGACCTCGCTCGTGCTTATGCTGATGTGCAAAGTTCAAAATCTGCACAACTTATAGGTGCCACACCATCCAACCCTGCTTTGTCTAGACGTGCACTTGCTGCACAGTTTGATCGTATCAATATCACACCCAGACAATTCTGCATGTATTTTGCAAAAATTGTTTGGAACATACTGTTAGACAGCAATGTGCCACCTGCCAACTGGGCAAAATTGGGCTATCAGGAAGATACCAAGTTTGCTGCTTTTGACTTCTTTGATGGAGTCACAAATCCAGCTAGTCTACAGCCTGCAGATGGCCTAATCAGGCAGCCCAATGAAAAAGAGCTTGCTGCTCACTCGGTTGCTAAATATGGTGCCCTTGCCCGCCAGAAAATATCCACTGGTAACTACATCACCACCCTTGGTGAAGTTACACGTGGTCACATGGGCGGCGCCAACACTATGTACGCAATTGATGCACCTCCTGAACTTTAA

>PS5_P8_c1

ATGGAAAGATCAACTTTGATCAATTTACTTCTGTTACACAAATTTGAACACAAGATTAACACTGAAGGAATCATTGTTGTGCACGGAATTGCTGGAACTGGGAAAACCACATTGCTTAGGACTTTATTTTCTGCTTACCCTAGCTTAGTTATAGGTTCACCTAGGCCTTGTTACTTAGATAAAGCTAATAAAATTTCACAAGTTTGCCTTTCTTGTTTTCCAAATACCTTGTGTGACATTGTTGACGAGTACCATCTCTTAGAAAGTTTTCCTGAACCAAAACTAGCCATTTTTGGTGACCCCTGTCAGTGCACTTACATTGAAAGGTTGAGAACACCCAACTACACATCCTTCAGAACACACCGATTTGGCAAATCCACTGCTGCTCTACTAAACAAGTTATTTGATCTTAACATTGAGTCAGTCAAAGCACAAGACGACACAGTAGAATACTTTGATCCTTTCGCAGTGGACCCCTCTGAACACATTTCTGCTTCAGAAAAAGAAGTTTTGGAATTTGTAGGTGATCAAGTTGAGACTACAAGCTCTGAAGAACTAGCTGGTCTCGAGTTTAGTGAAGTTACTTTCTACTGTACCACACTTGCTGGTGCTGTTCAAGAAAATCCTGCCAAAACCTTCATTTCACTCACTAGACACACTTCAAAGCTCACAATTGGTGAACTAAATGCCAGGTCTGACTCCTAGATGCCAGGTCTGACTCCTAGAGCTGATCTTACTGACACGTATAAAATCATTGCTATAGCCTTTCTACTGTCAGCTTGCATTTACTTCCAAAACAGTCATTATCAACCAGTTGCAGGTGATAATTTGCACAGACTACCCTTTGGTGGTCAGTATCAAGACGGAACTAAGAAGATCTCTTACTTTCCGCAGCAACAATCCTACTTTCACTCAGGAAACAAGCTTAATGTCCTCATACTTATCTTCATTCTTACACTGGGTATTGTCCTCACCAATAAATTTAGTTTTAGCATTAGCCGTAATACTCACCAGCATCATTGCTACAACACACATTCTGCAACCCAAACAGGTCAATCAGTGCCAGGTCATCATTGAATGTCCTCATACTTATCTTCATTCTTACACTGGGTATTGTCCTCACCAATAAATTTAGTTTTAGCATTAGCCGTAATACTCACCAGCATCATTGCTACAACACACATTCTGCAACCCAAACAGGTCAATCAGTGCCAGGTCATCATTGATGGTGCAGCCATAGTCATAACAAATTGTCCAAACACACCCGAAGTTCTTAAAGCAATCAACTTCTCCCCTTGGAACGGGTTAAGTTTTCCTCAATTGTGAATGGAAAACCAACCTACAGCTTCTAACCCATCAGATGTACCACCAACTGCTGCTCAAGCTGGTGCCCAGAGCCCAGCCGACTTCTCAAATCCTAATACAGCTCCTTCCCTAAGTGATTTGAAGAAGATCAAATACGTGTCAACTGTCACTTCAGTTGCCACGCCTGCTGAAATTGAGGCCCTTGGCAAGATCTTTACTGCCATGGGTTTAGCAGCCAATGAGACCGGACCTGCCATGTGGGACCTCGCTCGTGCTTATGCTGATGTGCAAAGTTCAAAATCTGCACAACTTATAGGTGCCACACCATCCAACCCTGCTTTGTCTAGACGTGCACTTGCTGCACAGTTTGATCGTATCAATATCACACCCAGACAATTCTGCATGTATTTTGCAAAAATTGTTTGGAACATACTGTTAGACAGCAATGTGCCACCTGCCAACTGGGCAAAATTGGGCTATCAGGAAGATACCAAGTTTGCTGCTTTTGACTTCTTTGATGGAGTCACAAATCCAGCTAGTCTACAGCCTGCAGATGGCCTAATCAGGCAGCCCAATGAAAAAGAGCTTGCTGCTCACTCGGTTGCTAAATATGGTGCCCTTGCCCGCCAGAAAATATCCACTGGTAACTACATCACCACCCTTGGTAAAGTTACACGTGGTCACATGGGCGGCGCCAACACTATGTACGCAATTGATGCACCTCCTGAACTTTAA

>PS5_P8_b14

ATGGAAAGATCAACTTTGATCAATTTACTTCTGTTACACAAATTTGAACACAAGATTAACACTGAAGGAATCATTGTTGTGCACGGAATTGCTGGAACTGGGAAAACCACATTGCTTAGGACTTTATTTTCTGCTTACCCTAGCTTAGTTATAGGTTCACCTAGGCCTTGTTACTTAGATAAAGCTAATAAAATTTCACAAGTTTGCCTTTCTTGTTTTCCAAATACCTTGTGTGACATTGTTGACGAGTACCATCTCTTAGAAAGTTTTCCTGAACCAAAACTAGCCATTTTTGGTGACCCCTGTCAGTGCACTTACATTGAAAGGTTGAGAACACCCAACTACACATCCTTCAGAACACACCGATTTGGCAAATCCACTGCTGCTCTACTAAACAAGTTATTTGATCTTAACATTGAGTCAGTCAAAGCACAAGACGACACAGTAGAATACTTTGATCCTTTCGCAGTGGACCCCTCTGAACACATTTCTGCTTCAGAAAAAGAAGTTTTGGAATTTGTAGGTGATCAAGTTGAGACTACAAGCTCTGAAGAACTAGCTGGTCTCGAGTTTAGTGAAGTTACTTTCTACTGTACCACACTTGCTGGTGCTGTTCAAGAAAATCCTGCCAAAACCTTCATTTCACTCACTAGACACACTTCAAAGCTCACAATTGGTGAACTAAATGCCAGGTCTGACTCCTAGATGCCAGGTCTGACTCCTAGAGCTGATCTTACTGACACGTATAAAATCATTGCTATAGCCTTTCTACTGTCAGCTTGCATTTACTTCCAAAACAGTCATTATCAACCAGTTGCAGGTGATAATTTGCACAGACTACCCTTTGGTGGTCAGTATCAAGACGGAACTAAGAAGATCTCTTACTTTCCGCAGCAACAATCCTACTTTCACTCAGGAAACAAGCTTAATGTCCTCATACTTATCTTCATTCTTACACTGGGTATTGTCCTCACCAATAAATTTAGTTTTAGCATTAGCCGTAATACCCACCAGCATCATTGCTACAACACACATTCTGCAACCCAAACAGGTCAATCAGTGCCAGGTCATCATTGAATGTCCTCATACTTATCTTCATTCTTACACTGGGTATTGTCCTCACCAATAAATTTAGTTTTAGCATTAGCCGTAATACCCACCAGCATCATTGCTACAACACACATTCTGCAACCCAAACAGGTCAATCAGTGCCAGGTCATCATTGATGGTGCAGCCATAGTCATAACAAATTGTCCAAACACACCCGAAGTTCTTAAAGCAATCAACTTCTCCCCTTGGAACGGGTTAAGTTTTCCTCAATTGTGAATGGAAAACCAACCTACAGCTTCTAACCCATCAGATGTACCACCAACTGCTGCTCAAGCTGGTGCCCAGAGCCCAGCCGACTTCTCAAATCCTAATACAGCTCCTTCCCTAAGTGATTTGAAGAAGATCAAATACGTGTCAACTGTCACTTCAGTTGCCACGCCTGCTGAAATTGAGGCCCTTGGCAAGATCTTTACTGCCATGGGTTTAGCAGCCAATGAGACCGGACCTGCCATGTGGGACCTCGCTCGTGCTTATGCTGATGTGCAAAGTTCAAAATCTGCACAACTTATAGGTGCCACACCATCCAACCCTGCTTTGTCTAGACGTGCACTTGCTGCACAGTTTGATCGTATCAATATCACACCCAGACAATTCTGCATGTATTTTGCAAAAATTGTTTGGAACATACTGTTAGACAGCAATGTGCCACCTGCCAACTGGGCAAAATTGGGCTATCAGGAAGATACCAAGTTTGCTGCTTTTGACTTCTTTGACGGAGTCACAAATCCAGCTAGTCTACAGCCTGCAGATGGCCTAATCAGGCAGCCCAATGAAAAAGAGCTTGCTGCTCACTCGGTTGCTAAATATGGTGCCCTTGCCCGCCAGAAAATATCCACTGGTAACTACATCACCACCCTTGGTGAAGTTACACGTGGTCACATGGGCGGCGCCAACACTATGTACGCAATTGATGCACCTCCTGAACTTTAA

>PS5_P8_b13

ATGGAAAGATCAACTTTGATCAATTTACTTCTGTTACACAAATTTGAACACAAGATTAACACTGAAGGAATCATTGTTGTGCACGGAATTGCTGGAACTGGGAAAACCACATTGCTTAGGACTTTATTTTCTGCTTACCCTAGCTTAGTTATAGGTTCACCTAGGCCTTGTTACTTAGATAAAGCTAATAAAATTTCACAAGTTTGCCTTTCTTGTTTTCCAAATACCTTGTGTGACATTGTTGACGAGTACCATCTCTTAGAAAGTTTTCCTGAACCAAAACTAGCCATTTTTGGTGACCCCTGTCAGTGCACTTACATTGAAAGGTTGAGAACACCCAACTACACATCCTTCAGAACACACCGATTTGGCAAATCCACTGCTGCTCTACTAAACAAGTTATTTGATCTTAACATTGAGTCAGTCAAAGCACAAGACGACACAGTAGAATACTTTGATCCTTTCGCAGTGGACCCCTCTGAACACATTTCCGCTTCAGAAAAAGAAGTTTTGGAATTTGTAGGTGATCAAGTTGAGACTACAAGCTCTGAAGAACTAGCTGGTCTCGAGTTTAGTGAAGTTACTTTCTACTGTACCACACTTGCTGGTGCTGTTCAAGAAAATCCTGCCAAAACCTTCATTTCACTCACTAGACACACCTCAAAGCTCACAATTGGTGAACTAAATGCCAGGTCTGACTCCTAGATGCCAGGTCTGACTCCTAGAGCTGATCTTACTGACACGTATAAAATCATTGCTATAGCCTTTCTACTGTCAGCTTGCATTTACTTCCAAAACAGTCATTATCAACCAGTTGCAGGTGATAATTTGCACAGACTACCCTTTGGTGGTCAGTATCAAGACGGAACTAAGAAGATCTCTTACTTTCCGCAGCAACAATCCTACTTTCACTCAGGAAACAAGCTTAATGTCCTCATACTTATCTTCATTCTTACACTGGGTATTGTCCTCACCAATAAATTTAGTTTTAGCATTAGCCGTAATACTCACCAGCATCATTGCTACAACACACATTCTGCAACCCAAACAGGTCAATCAGTGCCAGGTCATCATTGAATGTCCTCATACTTATCTTCATTCTTACACTGGGTATTGTCCTCACCAATAAATTTAGTTTTAGCATTAGCCGTAATACTCACCAGCATCATTGCTACAACACACATTCTGCAACCCAAACAGGTCAATCAGTGCCAGGTCATCATTGATGGTGCAGCCATAGTCATAACAAATTGTCCAAACACACCCGAAGTTCTTAAAGCAATCAACTTCTCCCCTTGGAACGGGTTAAGTTTTCCTCAATTGTGAATGGAAAACCAACCTACAGCTTCTAACCCATCAGATGTACCACCAACTGCTGCTCAAGCTGGTGCCCAGAGCCCAGCCGACTTCTCAAATCCTAATACAGCTCCTTCCCTAAGTGATTTGAAGAAGATCAAATACGTGTCAACTGTCACTTCAGTTGCCACGCCTGCTGAAATTGAGGCCCTTGGCAAGATCTTTACTGCCATGGGTTTAGCAGCCAATGAGACCGGACCTGCCATGTGGGACCTCGCTCGTGCTTATGCTGATGTGCAAAGTTCAAAATCTGCACAACTTATAGGTGCCACACCATCCAACCCTGCTTTGTCTAGACGTGCACTTGCTGCACAGTTTGATCGTATCAATATCACACCCAGACAATTCTGCATGTATTTTGCAAAAATTGTTTGGAACATACTGTTAGACAGCAATGTGCCACCTGCCAACTGGGCAAAATTGGGCTATCAGGAAGATACCAAGTTTGCTGCTTTTGACTTCTTTGACGGAGTCACAAATCCAGCTAGTCTACAGCCTGCAGATGGCCTAATCAGGCAGCCCAATGAAAAAGAGCTTGCTGCTCACTCGGTTGCTAAATATGGTGCCCTTGCCCGCCAGAAAATATCCACTGGTAACTACATCACCACCCTTGGTGAAGTTACACGTGGTCACATGGGCGGCGCCAACACTATGTACGCAATTGATGCACCTCCTGAACTTTAA

>PS5_P8_b12

ATGGAAAGATCAACTTTGATCAATTTACTTCTGTTACACAAATTTGAACACAAGATTAACACTGAAGGAATCATTGTTGTGCACGGAATTGCTGGAACTGGGAAAACCACATTGCTTAGGACTTTATTTTCTGCTTACCCTAGCTTAGTTATAGGTTCACCTAGGCCTTGTTACTTAGATAAAGCTAATAAAATTTCACAAGTTTGCCTTTCTTGTTTTCCAAATACCTTGTGTGACATTGTTGACGAGTACCATCTCTTAGAAAGTTTTCCTGAACCAAAACTAGCCATTTTTGGTGACCCCTGTCAGTGCACTTACATTGAAAGGTTGAGAACACCCAACTACACATCCTTCAGAACACACCGATTTGGCAAATCCACTGCTGCTCTACTAAACAAGTTATTTGATCTTAACATTGAGTCAGTCAAAGCACAAGACGACACAGTAGAATACTTTGATCCTTTCGCAGTGGACCCCTCTGAACACATTTCTGCTTCAGAAAAAGAAGTTTTGGAATTTGTAGGTGATCAAGTTGAGACTACAAGCTCTGAAGAACTAGCTGGTCTCGAGTTTAGTGAAGTTACTTTCTACTGTACCACACTTGCTGGTGCTGTTCAAGAAAATCCTGCCAAAACCTTCATTTCACTCACTAGACACACTTCAAAGCTCACAATTGGTGAACTAAATGCCAGGTCTGACTCCTAGATGCCAGGTCTGACTCCTAGAGCTGATCTTACTGACACGTATAAAATCATTGCTATAGCCTTTCTACTGTCAGCTTGCATTTACTTCCAAAACAGTCATTATCAACCAGTTGCAGGTGATAATTTGCACAGACTACCCTTTGGTGGTCAGTATCAAGACGGAACTAAGAAGATCTCTTACTTTCCGCAGCAACAATCCTACTTTCACTCAGGAAACAAGCTTAATGTCCTCATACTTATCTTCATTCTTACACTGGGTATTGTCCTCACCAATAAATTTAGTTTTAGCATTAGCCGTAATACTCACCAGCATCATTGCTACAACACACATTCTGCAACCCAAACAGGTCAATCAGTGCCAGGTCATCATTGAATGTCCTCATACTTATCTTCATTCTTACACTGGGTATTGTCCTCACCAATAAATTTAGTTTTAGCATTAGCCGTAATACTCACCAGCATCATTGCTACAACACACATTCTGCAACCCAAACAGGTCAATCAGTGCCAGGTCATCATTGATGGTGCAGCCATAGTCATAACAAATTGTCCAAACACACCCGAAGTTCTTAAAGCAATCAACTTCTCCCCTTGGAACGGGTTAAGCTTTCCTCAATTGTGAATGGAAAACCAACCTACAGCTTCTAACCCATCAGATGTACCACCAACTGCTGCTCAAGCTGGTGCCCAGAGCCCAGCCGACTTCTCAAATCCTAATACAGCTCCTTCCCTAAGTGATTTGAAGAAGATCAAATACGTGTCAACTGTCACTTCAGTTGCCACGCCTGCTGAAATTGAGGCCCTTGGCAAGATCTTTACTGCCATGGGTTTAGCAGCCAATGAGACCGGACCTGCCATGTGGGACCTCGCTCGTGCTTATGCTGATGTGCAAAGTTCAAAATCTGCACAACTTATAGGTGCCACACCATCCAACCCTGCTTTGTCTAGACGTGCACTTGCTGCACAGTTTGATCGTATCAATATCACACCCAGACAATTCTGCATGTATTTTGCAAAAATTGTTTGGAACATACTGTTAGACAGCAATGTGCCACCTGCCAACTGGGCAAAATTGGGCTATCAGGAAGATACCAAGTTTGCTGCTTTTGACTTCTTTGACGGAGTCACAAATCCAGCTAGTCTACAGCCTGCAGATGGCCTAATCAGGCAGCCCAATGAAAAAGAGCTTGCTGCTCACTCGGTTGCTAAATATGGTGCCCTTGCCCGCCAGAAAATATCCACTGGTAACTACATCACCACCCTTGGTGAAGTTACACGTGGTCACATGGGCGGCGCCAACACTATGTACGCAATTGATGCACCTCCTGAACTTTAA

>PS5_P8_b11

ATGGAAAGATCAACTTTGATCAATTTACTTCTGTTACACAAATTTGAACACAAGATTAACACTGAAGGAATCATTGTTGTGCACGGAATTGCTGGAACTGGGAAAACCACATTGCTTAGGACTTTATTTTCTGCTTACCCTAGCTTAGTTATAGGTTCACCTAGGCCTTGTTACTTAGATAAAGCTAATAAAATTTCACAAGTTTGCCTTTCTTGTTTTCCAAATACCTTGTGTGACATTGTTGACGAGTACCATCTCTTAGAAAGTTTTCCTGAACCAAAACTAGCCATTTTTGGTGACCCCTGTCAGTGCACTTACATTGAAAGGTTGAGAACACCCAACTACACATCCTTCAGAACACACCGATTTGGCAAATCCACTGCTGCTCTACTAAACAAGTTATTTGATCTTAACATTGAGTCAGTCAAAGCACAAGACGACACAGTAGAATACTTTGATCCTTTCGCAGTGGACCCCTCTGAACACATTTCTGCTTCAGAAAAAGAAGTTTTGGAATTTGTAGGTGATCAAGTTGAGACTACAAGCTCTGAAGAACTAGCTGGTCTCGAGTTTAGTGAAGTTACTTTCTACTGTACCACACTTGCTGGTGCTGTTCAAGAAAATCCTGCCAAAACCTTCATTTCACTCACTAGACACACTTCAAAGCTCACAATTGGTGAACTAAATGCCAGGTCTGACTCCTAGATGCCAGGTCTGACTCCTAGAGCTGATCTTACTGACACGTATAAAATCATTGCTATAGCCTTTCTACTGTCAGCTTGCATTTACTTCCAAAACAGTCATTATCAACCAGTTGCAGGTGATAATTTGCACAGACTACCCTTTGGTGGTCAGTATCAAGACGGAACTAAGAAGGTCTCTTACTTTCCGCAGCAACAATCCTACTTTCACTCAGGAAACAAGCTTAATGTCCTCATACTTATCTTCATTCTTACACTGGGTATTGTCCTCACCAATAAATTTAGTTTTAGCATTAGCCGTAATACTCACCAGCATCATTGCTACAACACACATTCTGCAACCCAAACAGGTCAATCAGTGCCAGGTCATCATTGAATGTCCTCATACTTATCTTCATTCTTACACTGGGTATTGTCCTCACCAATAAATTTAGTTTTAGCATTAGCCGTAATACTCACCAGCATCATTGCTACAACACACATTCTGCAACCCAAACAGGTCAATCAGTGCCAGGTCATCATTGATGGTGCAGCCATAGTCATAACAAATTGTCCAAACACACCCGAAGTTCTTAAAGCAATCAACTTCTCCCCTTGGAACGGGTTAAGTTTTCCTCAATTGTGAATGGAAAACCAACCTACAGCTTCTAACCCATCAGATGTACCACCAACTGCTGCTCAAGCTGGTGCCCAGAGCCCAGCCGGCTTCTCAAATCCTAATACAGCTCCTTCCCTAAGTGATTTGAAGAAGATCAAATACGTGTCAACTGTCACTTCAGTTGCCACGCCTGCTGAAATTGAGGCCCTTGGCAAGATCTTTACTGCCATGGGTTTAGCAGCCAATGAGACCGGACCTGCCATGTGGGACCTCGCTCGTGCTTATGCTGATGTGCAAAGTTCAAAATCTGCACAACTTATAGGTGCCACACCATCCAACCCTGCTTTGTCTAGACGTGCACTTGCTGCACAGTTTGATCGTATCAATATCACACCCAGACAATTCTGCATGTATTTTGCAAAAATTGTTTGGAACATACTGTTAGACAGCAATGTGCCACCTGCCAACTGGGCAAAATTGGGCTATCAGGAAGATACCAAGTTTGCTGCTTTTGACTTCTTTGACGGAGTCACAAATCCAGCTAGTCTACAGCCTGCAGATGGCCTAATCAGGCAGCCCAATGAAAAAGAGCTTGCTGCTCACTCGGTTGCTAAATATGGTGCCCTTGCCCGCCAGAAAATATCCACTGGTAACTACATCACCACCCTTGGTGAAGTTACACGTGGTCACATGGGCGGCGCCAACACTATGTACGCAATTGATGCACCTCCTGAACTTTAA

>PS5_P8_b10

ATGGAAAGATCAACTTTGATCAATTTACTTCTGTTACACAAATTTGAACACAAGATTAACACTGAAGGAATCATTGTTGCGCACGGAATTGCTGGAACTGGAAAAACCACATTGCTTAGGACTTTATTTTCTGCTTACCCTAGCTTAGTTATAGGTTCACCTAGGCCTTGTTACTTAGATAAAGCTAATAAAATTTCACAAGTTTGCCTTTCTTGTTTTCCAAATACCTTGTGTGACATTGTTGACGAGTACCATCTCTTAGAAAGTTTTCCTGAACCAAAACTAGCCATTTTTGGTGACCCCTGTCAGTGCACTTACATTGAAAGGTTGAGAACACCCAACTACACATCCTTCAGAACACACCGATTTGGCAAATCCACTGCTGCTCTACTAAACAAGTTATTTGATCTTAACATTGAGTCAGTCAAAGCACAAGACGACACAGTAGAATACTTTGATCCTTTCGCAGTGGACCCCTCTGAACACATTTCTGCTTCAGAAAAAGAAGTTTTGGAATTTGTAGGTGATCAAGTTGAGACTACAAGCTCTGAAGAACTAGCTGGTCTCGAGTTTAGTGAAGTTACTTTCTACTGTACCACACTTGCTGGTGCTGTTCAAGAAAATCCTGCCAAAACCTTCATTTCACTCACTAGACACACTTCAAAGCTCACAATTGGTGAACTAAATGCCAGGTCTGACTCCTAGATGCCAGGTCTGACTCCTAGAGCTGATCTTACTGACACGTATAAAATCATTGCTATAGCCTTTCTACTGTCAGCTTGCATTTACTTCCAAAACAGTCATTATCAACCAGTTGCAGGTGATAATTTGCACAGACTACCCTTTGGTGGTCAGTATCAAGACGGAACTAAGAAGATCTCTTACTTTCCGCAGCAACAATCCTACTTTCACTCAGGAAACAAGCTTAATGTCCTCATACTTATCTTCATTCTTACACTGGGTATTGTCCTCACCAATAAATTTAGTTTTAGCATTAGCCGTAATACTCACCAGCATCATTGCTACAACACACATTCTGCAACCCAAACAGGTCAATCAGTGCCAGGTCATCATTGAATGTCCTCATACTTATCTTCATTCTTACACTGGGTATTGTCCTCACCAATAAATTTAGTTTTAGCATTAGCCGTAATACTCACCAGCATCATTGCTACAACACACATTCTGCAACCCAAACAGGTCAATCAGTGCCAGGTCATCATTGATGGTGCAGCCATAGTCATAACAAATTGTCCAAACACACCCGAAGTTCTTAAAGCAATCAACTTCTCCCCTTGGAACGGGTTAAGTTTTCCTCAATTGTGAATGGAAAACCAACCTACAGCTTCTAACCCATCAGATGTACCACCAACTGCTGCTCAAGCTGGTGCCCAGAGCCCAGCCGACTTCTCAAATCCTAATACAGCTCCTTCCCTAAGTGATTTGAAGAAGATCAAATACGTGTCAACTGTCACTTCAGTTGCCACGCCTGCTGAAATTGAGGCCCTTGGCAAGATCTTTACTGCCATGGGTTTAGCAGCCAATGAGACCGGACCTGCCATGTGGGACCTCGCTCGTGCTTATGCTGATGTGCAAAGTTCAAAATCTGCACAACTTATAGGTGCCACACCATCCAACCCTGCTTTGTCTAGACGTGCACTTGCTGCACAGTTTGATCGTATCAATATCACACCCAGACAATTCTGCATGTATTTTGCAAAAATTGTTTGGAACATACTGTTAGACAGCAATGTGCCACCTGCCAACTGGGCAAAATTGGGCTATCAGGAAGATACCAAGTTTGCTGCTTTTGACTTCTTTGATGGAGTCACAAATCCAGCTAGTCTACAGCCTGCAGATGGCCTAATCAGGCAGCCCAATGAAAAAGAGCTTGCTGCTCACTCGGTTGCTAAATATGGTGCCCTTGCCCGCCAGAAAATATCCACTGGTAACTACATCACCACCCTTGGTGAAGTTACACGTGGTCACATGGGCGGCGCCAACACTATGTACGCAATTGATGCACCTCCTGAACTTTAA

>PS5_P8_b9

ATGGAAAGATCAACTTTGATCAATTTACTTCTGTTACACAAATTTGAACACAAGATTAACACTGAAGGAATCATTGTTGTGCACGGAATTGCTGGAACTGGGAAAACCACATTGCTTAGGACTTTATTTTCTGCTTACCCTAGCTTAGTTATAGGTTCACCTAGGCCTTGTTACTTAGATAAAGCTAATAAAATTTCACAAGTTTGCCTTTCTTGTTTTCCAAATACCTTGTGTGACATTGTTGACGAGTACCATCTCTTAGAAAGTTTTCCTGAACCAAAACTAGCCATTTTTGGTGACCCCTGTCAGTGCACTTACATTGAAAGGTTGAGAACACCCAACTACACATCCTTCAGAACACACCGATTTGGCAAATCCACTGCTGCTCTACTAAACAAGTTATTTGATCTTAACATTGAGTCAGTCAAAGCACAAGACGACACAGTAGAATACTTTGATCCTTTCGCAGTGGACCCCTCTGAACACATTTCTGCTTCAGAAAAAGAAGTTTTGGAATTTGTAGGTGATCAAGTTGAGACTACAAGCTCTGAAGAACTAGCTGGTCTCGAGTTTAGTGAAGTTACTTTCTACTGTACCACACTTGCTGGTGCTGTTCAAGAAAATCCTGCCAAAACCTTCATTTCACTCACTAGACACACTTCAAAGCTCACAATTGGTGAACTAAATGCCAGGTCTGACTCCTAGATGCCAGGTCTGACTCCTAGAGCTGATCTTACTGACACGTATAAAATCATTGCTATAGCCTTTCTACTGTCAGCTTGCATTTACTTCCAAAACAGTCATTATCAACCAGTTGCAGGTGATAATTTGCACAGACTACCCTTTGGTGGTCAGTATCAAGACGGAACTAAGAAGATCTCTTACTTTCCGCAGCAACAATCCTACTTTCACTCAGGAAACAAGCTTAATGTCCTCATACTTATCTTCATTCTTACACTGGGTATTGTCCTCACCAATAAATTTAGTTTTAGCATTAGCCGTAATACTCACCAGCATCATTGCTACAACACACATTCTGCAACCCAAACAGGTCAATCAGTGCCAGGTCATCATTGAATGTCCTCATACTTATCTTCATTCTTACACTGGGTATTGTCCTCACCAATAAATTTAGTTTTAGCATTAGCCGTAATACTCACCAGCATCATTGCTACAACACACATTCTGCAACCCAAACAGGTCAATCAGTGCCAGGTCATCATTGATGGTGCAGCCATAGTCATAACAAATTGTCCAAACACACCCGAAGTTCTTAAAGCAATCAACTTCTCCCCTTGGAACGGGTTAAGTTTTCCTCAATTGTGAATGGAAAACCAACCTACAGCTTCTAACCCATCAGATGTACCACCAACTGCTGCTCAAGCTGGTGCCCAGAGCCCAGCCGACTTCTCAAATCCTAATACAGCTCCTTCCCTAAGTGATTTGAAGAAGATCAAATACGTGTCAACTGTCACTTCAGTTGCCACGCCTGCTGAAATTGAGGCCCTTGGCAAGATCTTTACTGCCATGGGTTTAGCAGCCAATGAGACCGGACCTGCCATGTGGGACCTCGCTCGTGCTTATGCTGATGTGCAAAGTTCAAAATCTGCACAACTTATAGGTGCCACACCATCCAACCCTGCTTTGTCTAGACGTGCACTTGCTGCACAGTTTGATCGTATCAATATCACACCCAGACAATTCTGCATGTATTTTGCAAAAATTGTTTGGAACATACTGTTAGACAGCAATGTGCCACCTGCCAACTGGGCAAAATTGGGCTATCAGGAAGATACCAAGTTTGCTGCTTTTGACTTCTTTGATGGAGTCACAAATCCAGCTAGTCTACAGCCTGCAGATGGCCTAATCAGGCAGCCCAATGAAAAAGAGCTTGCTGCTCACTCGGTTGCTAAATATGGTGCCCTTGCCCGCCAGAAAATATCCACTGGTAACTACATCACCACCCTTGGTGAAGTTACACGTGGTCACATGGGCGGCGCCAACACTATGTACGCAATTGATGCACCTCCTGAACTTTAA

>PS5_P8_b8

ATGGAAAGATCAACTTTGATCAATTTACTTCTGTTACACAAATTTGAACACAAGATTAACACTGAAGGAATCATTGTTGTGCACGGAATTGCTGGAACTGGGAAAACCACATTGCTTAGGACTTTATTTTCTGCTTACCCTAGCTTAGTTATAGGTTCACCTAGGCCTTGTTACTTAGATAAAGCTAATAAAATTTCACAAGTTTGCCTTTCTTGTTTTCCAAATACCTTGTGTGACATTGTTGACGAGTACCATCTCTTAGAAAGTTTTCCTGAACCAAAACTAGCCATTTTTGGTGACCCCTGTCAGTGCACTTACATTGAAAGGTTGAGAACACCCAACTACACATCCTTCAGAACACACCGATTTGGCAAATCCACTGCTGCTCTACTAAACAAGTTATTTGATCTTAACATTGAGTCAGTCAAAGCACAAGACGACACAGTAGAATACTTTGATCCTTTCGCAGTGGACCCCTCTGAACACATTTCTGCTTCAGAAAAAGAAGTTTTGGAATTTGTAGGTGATCAAGTTGAGACTACAAGCTCTGAAGAACTAGCTGGTCTCGAGTTTAGTGAAGTTACTTTCTACTGTACCACACTTGCTGGTGCTGTTCAAGAAAATCCTGCCAAAACCTTCATTTCACTCACTAGACACACTTCAAAGCTCACAATTGGTGAACTAAATGCCAGGTCTGACTCCTAGATGCCAGGTCTGACTCCTAGAGCTGATCTTACTGACACGTATAAAATCATTGCTATAGCCTTTCTACTGTCAGCTTGCATTTACTTCCAAAACAGTCATTATCAACCAGTTGCAGGTGATAATTTGCACAGACTACCCTTTGGTGGTCAGTATCAAGACGGAACTAAGAAGATCTCTTACTTTCCGCAGCAACAATCCTACTTTCACTCAGGAAACAAGCTTAATGTCCTCATACTTATCTTCATTCTTACACTGGGTATTGTCCTCACCAATAAATTTAGTTTTAGCATTAGCCGTAATACTCACCAGCATCATTGCTACAACACACATTCTGCAACCCAAACAGGTCAATCAGTGCCAGGTCATCATTGAATGTCCTCATACTTATCTTCATTCTTACACTGGGTATTGTCCTCACCAATAAATTTAGTTTTAGCATTAGCCGTAATACTCACCAGCATCATTGCTACAACACACATTCTGCAACCCAAACAGGTCAATCAGTGCCAGGTCATCATTGATGGTGCAGCCATAGTCATAACAAATTGTCCAAACACACCCGAAGTTCTTAAAGCAATCAACTTCTCCCCTTGGAACGGGTTAAGTTTTCCTCAATTGTGAATGGAAAACCAACCTACAGCTTCTAACCCATCAGATGTACCACCAACTGCTGCTCAAGCTGGTGCCCAGAGCCCAGCCGACTTCTCAAATCCTAATACAGCTCCTTCCCTAAGTGATTTGAAGAAGATCAAATACGTGTCAACTGTCACTTCAGTTGCCACGCCTGCTGAAATTGAGGCCCTTGGCAAGATCTTTACTGCCATGGGTTTAGCAGCCAATGAGACCGGACCTGCCATGTGGGACCTCGCTCGTGCTTATGCTGATGTGCAAAGTTCAAAATCTGCACAACTTATAGGTGCCACACCATCCAACCCTGCTTTGTCTAGACGTGCACTTGCTGCACAGTTTGATCGTATCAATATCACACCCAGACAATTCTGCATGTATTTTGCAAAAATTGTTTGGAACATACTGTTAGACAGCAATGTGCCACCTGCCAACTGGGCAAAATTGGGCTATCAGGAAGATACCAAGTTTGCTGCTTTTGACTTCTTTGATGGAGTCACAAATCCAGCTAGTCTACAGCCTGCAGATGGCCTAATCAGGCAGCCCAATGAAAAAGAGCTTGCTGCTCACTCGGTTGCTAAATATGGTGCCCTTGCCCGCCAGAAAATATCCACTGGTAACTACATCACCACCCTTGGTGAAGTTACACGTGGTCACATGGGCGGCGCCAACACTATGTACGCAATTGATGCACCTCCTGAACTTTAA

>PS5_P8_b7

ATGGAAAGATCAACTTTGATCAATTTACTTCTGTTACACAAATTTGAACACAAGATTAACACTGAAGGAATCATTGTTGTGCACGGAATTGCTGGAACTGGGAAAACCACATTGCTTAGGACTTTATTTTCTGCTTACCCTAGCTTAGTTATAGGTTCACCTAGGCCTTGTTACTTAGATAAAGCTAATAAAATTTCACAAGTTTGCCTTTCTTGTTTTCCAAATACCTTGTGTGACATTGTTGACGAGTACCATCTCTTAGAAAGTTTTCCTGAACCAAAACTAGCCATTTTTGGTGACCCCTGTCAGTGCACTTACATTGAAAGGTTGAGAACACCCAACTACACATCCTTCAGAACACACCGATTTGGCAAATCCACTGCTGCTCTACTAAACAAGTTATTTGATCTTAACATTGAGTCAGTCAAAGCACAAGACGACACAGTAGAATACTTTGATCCTTTCGCAGTGGACCCCTCTGAACACATTTCTGCTTCAGAAAAAGAAGTTTTGGAATTTGTAGGTGATCAAGTTGAGACTACAAGCTCTGAAGAACTAGCTGGTCTCGAGTTTAGTGAAGTTACTTTCTACTGTACCACACTTGCTGGTGCTGTTCAAGAAAATCCTGCCAAAACCTTCATTTCACTCACTAGACACACTTCAAAGCTCACAATTGGTGAACTAAATGCCAGGTCTGACTCCTAGATGCCAGGTCTGACTCCTAGAGCTGATCTTACTGACACGTATAAAATCATTGCTATAGCCTTTCTACTGTCAGCTTGCATTTACTTCCAAAACAGTCATTATCAACCAGTTGCAGGTGATAATTTGCACAGACTACCCTTTGGTGGTCAGTATCAAGACGGAACTAAGAAGATCTCTTACTTTCCGCAGCAACAATCCTACTTTCACTCAGGAAACAAGCTTAATGTCCTCATACTTATCTTCATTCTTACACTGGGTATTGTCCTCACCAATAAATTTAGTTTTAGCATTAGCCGTAATACTCACCAGCATCATTGCTACAACACACATTCTGCAACCCAAACAGGTCAATCAGTGCCAGGTCATCATTGAATGTCCTCATACTTATCTTCATTCTTACACTGGGTATTGTCCTCACCAATAAATTTAGTTTTAGCATTAGCCGTAATACTCACCAGCATCATTGCTACAACACACATTCTGCAACCCAAACAGGTCAATCAGTGCCAGGTCATCATTGATGGTGCAGCCATAGTCATAACAAATTGTCCAAACACACCCGAAGTTCTTAAAGCAATCAACTTCTCCCCTTGGAACGGGTTAAGTTTTCCTCAATTGTGAATGGAAAACCAACCTACAGCTTCTAACCCATCAGATGTACCACCAACTGCTGCTCAAGCTGGTGCCCAGAGCCCAGCCGACTTCTCAAATCCTAATACAGCTCCTTCCCTAAGTGATTTGAAGAAGATCAAATACGTGTCAACTGTCACTTCAGTTGCCACGCCTGCTGAAATTGAGGCCCTTGGCAAGATCTTTACTGCCATGGGTTTAGCAGCCAATGAGACCGGACCTGCCATGTGGGACCTCGCTCGTGCTTATGCTGATGTGCAAAGTTCAAAATCTGCACAACTTATAGGTGCCACACCATCCAACCCTGCTTTGTCTAGACGTGCACTTGCTGCACAGTTTGATCGTATCAATATCACACCCAGACAATTCTGCATGTATTTTGCAAAAATTGTTTGGAACATACTGTTAGACAGCAATGTGCCACCTGCCAACTGGGCAAAATTGGGCTATCAGGAAGATACCAAGTTTGCTGCTTTTGACTTCTTTGATGGAGTCACAAATCCAGCTAGTCTACAGCCTGCAGATGGCCTAATCAGGCAGCCCAATGAAAAAGAGCTTGCTGCTCACTCGGTTGCTAAATATGGTGCCCTTGCCCGCCAGAAAATATCCACTGGTAACTACATCACCACCCTTGGTGAAGTTACACGTGGTCACATGGGCGGCGCCAACACTATGTACGCAATTGATGCACCTCCTGAACTTTAA

>PS5_P8_b6

ATGGAAAGATCAACTTTGATCAATTTACTTCTGTTACACAAATTTGAACACAAGATTAACACTGAAGGAATCATTGTTGTGCACGGAATTGCTGGAACTGGGAAAACCACATTGCTTAGGACTTTATTTTCTGCTTACCCTAGCTTAGTTATAGGTTCACCTAGGCCTTGTTACTTAGATAAAGCTAATAAAATTTCACAAGTTTGCCTTTCTTGTTTTCCAAATACCTTGTGTGACATTGTTGACGAGTACCATCTCTTAGAAAGTTTTCCTGAACCAAAACTAGCCATTTTTGGTGACCCCTGTCAGTGCACTTACATTGAAAGGTTGAGAACACCCAACTACACATCCTTCAGAACACACCGATTTGGCAAATCCACTGCTGCTCTACTAAACAAGTTATTTGATCTTAACATTGAGTCAGTCAAAGCACAAGACGACACAGTAGAATACTTTGATCCTTTCGCAGTGGACCCCTCTGAACACATTTCTGCTTCAGAAAAAGAAGTTTTGGAATTTGTAGGTGATCAAGTTGAGACTACAAGCTCTGAAGAACTAGCTGGTCTCGAGTTTAGTGAAGTTACTTTCTACTGTACCACACTTGCTGGTGCTGTTCAAGAAAATCCTGCCAAAACCTTCATTTCACTCACTAGACACACTTCAAAGCTCACAATTGGTGAACTAAATGCCAGGTCTGACTCCTAGATGCCAGGTCTGACTCCTAGAGCTGATCTTACTGACACGTATAAAATCATTGCTATAGCCTTTCTACTGTCAGCTTGCATTTACTTCCAAAACAGTCATTATCAACCAGTTGCAGGTGATAATTTGCACAGACTACCCTTTGGTGGTCAGTATCAAGACGGAACTAAGAAGATCTCTTACTTTCCGCAGCAACAATCCTACTTTCACTCAGGAAACAAGCTTAATGTCCTCATACTTATCTTCATTCTTACACTGGGTATTGTCCTCACCAATAAATTTAGTTTTAGCATTAGCCGTAATACTCACCAGCATCATTGCTACAACACGCATTCTGCAACCCAAACAGGTCAATCAGTGCCAGGTCATCATTGAATGTCCTCATACTTATCTTCATTCTTACACTGGGTATTGTCCTCACCAATAAATTTAGTTTTAGCATTAGCCGTAATACTCACCAGCATCATTGCTACAACACGCATTCTGCAACCCAAACAGGTCAATCAGTGCCAGGTCATCATTGATGGTGCAGCCATAGTCATAACAAATTGTCCAAACACACCCGAAGTTCTTAAAGCAATCAACTTCTCCCCTTGGAACGGGTTAAGTTTTCCTCAATTGTGAATGGAAAACCAACCTACAGCTTCTAACCCATCAGATGTACCACCAACTGCTGCTCAAGCTGGTGCCCAGAGCCCAGCCGACTTCTCAAATCCTAATACAGCTCCTTCCCTAAGTGATTTGAAGAAGATCAAATACGTGTCAACTGTCACTTCAGTTGCCACGCCTGCTGAAATTGAGGCCCTTGGCAAGATCTTTACTGCCATGGGTTTAGCAGCCAATGAGACCGGACCTGCCATGTGGGACCTCGCTCGTGCTTATGCTGATGTGCAAAGTTCAAAATCTGCACAACTTATAGGTGCCACACCATCCAACCCTGCTTTGTCTAGACGTGCACTTGCTGCACATTTTGATCGTATCAATATCACACCCAGACAATTCTGCATGTATTTTGCAAAAATTGTTTGGAACATACTGTTAGACAGCAATGTGCCACCTGCCAACTGGGCAAAATTGGGCTATCAGGAAGATACCAAGTTTGCTGCTTTTGACTTCTTTGATGGAGTCACAAATCCAGCTAGTCTACAGCCTGCAGATGGCCTAATCAGGCAGCCCAATGAAAAAGAGCTTGCTGCTCACTCGGTTGCTAAATATGGTGCCCTTGCCCGCCAGAAAATATCCACTGGTAACTACATCACCACCCTTGGTGAAGTTACACGTGGTCACATGGGCGGCGCCAACACTATGTACGCAATTGATGCACCTCCTGAACTTTAA

>PS5_P8_b5

ATGGAAAGATCAACTTTGATCAATTTACTTCTGTTACACAAATTTGAACACAAGATTAACACTGAAGGAATCATTGTTGTGCACGGAATTGCTGGAACTGGGAAAACCACATTGCTTAGGACTTTATTTTCTGCTTACCCTAGCTTAGTTATAGGTTCACCTAGGCCTTGTTACTTAGATAAAGCTAATAAAATTTCACAAGTTTGCCTTTCTTGTTTTCCAAATACCTTGTGTGACATTGTTGACGAGTACCATCTCTTAGAAAGTTTTCCTGAACCAAAACTAGCCATTTTTGGTGACCCCTGTCAGTGCACTTACATTGAAAGGTTGAGAACACCCAACTACACATCCTTCAGAACACACCGATTTGGCAAATCCACTGCTGCTCTACTAAACAAGTTATTTGATCTTAACATTGAGTCAGTCAAAGCACAAGACGACACAGTAGAATACTTTGATCCTTTCGCAGTGGACCCCTCTGAACACATTTCTGCTTCAGAAAAAGAAGTTTTGGAATTTGTAGGTGATCAAGTTGAGACTACAAGCTCTGAAGAACTAGCTGGTCTCGAGTTTAGTGAAGTTACTTTCTACTGTACCACACTTGCTGGTGCTGTTCAAGAAAATCCTGCCAAAACCTTCATTTCACTCACTAGACACACTTCAAAGCTCACAATTGGTGAACTAAATGCCAGGTCTGACTCCTAGATGCCAGGTCTGACTCCTAGAGCTGATCTTACTGACACGTATAAAATCATTGCTATAGCCTTTCTACTGTCAGCTTGCATTTACTTCCAAAACAGTCATTATCAACCAGTTGCAGGTGATAATTTGCACAGACTACCCTTTGGTGGTCAGTATCAAGACGGAACTAAGAAGATCTCTTACTTTCCGCAGCAACAATCCTACTTTCACTCAGGAAACAAGCTTAATGTCCTCATACTTATCTTCATTCTTACACTGGGTATTGTCCTCACCAATAAATTTAGTTTTAGCATTAGCCGTAATACTCACCAGCATCATTGCTACAACACACATTCTGCAACCCAAACAGGTCAATCAGTGCCAGGTCATCATTGAATGTCCTCATACTTATCTTCATTCTTACACTGGGTATTGTCCTCACCAATAAATTTAGTTTTAGCATTAGCCGTAATACTCACCAGCATCATTGCTACAACACACATTCTGCAACCCAAACAGGTCAATCAGTGCCAGGTCATCATTGATGGTGCAGCCATAGTCATAACAAATTGTCCAAACACACCCGAAGTTCTTAAAGCAATCAACTTCTCCCCTTGGAACGGGTTAAGTTTTCCTCAATTGTGAATGGAAAACCAACCTACAGCTTCTAACCCATCAGATGTACCACCAACTGCTGCTCAAGCTGGTGCCCAGAGCCCAGCCGACTTCTCAAATCCTAATACAGCTCCTTCCCTAAGTGATTTGAAGAAGATCAAATACGTGTCAACTGTCACTTCAGTTGCCACGCCTGCTGAAATTGAGGCCCTTGGCAAGATCTTTACTGCCATGGGTTTAGCAGCCAATGAGACCGGACCTGCCATGTGGGACCTCGCTCGTGCTTATGCTGATGTGCAAAGTTCAAAATCTGCACAACTTATAGGTGCCACACCATCCAACCCTGCTTTGTCTAGACGTGCACTTGCTGCACAGTTTGATCGTATCAATATCACACCCAGACAATTCTGCATGTATTTTGCAAAAATTGTTTGGAACATACTGTTAGACAGCAATGTGCCACCTGCCAACTGGGCAAAATTGGGCTATCAGGAAGATACCAAGTTTGCTGCTTTTGACTTCTTTGACGGAGTCACAAATCCAGCTAGTCTACAGCCTGCAGATGGCCTAATCAGGCAGCCCAATGAAAAAGAGCTTGCTGCTCACTCGGTTGCTAAATATGGTGCCCTTGCCCGCCAGAAAATATCCACTGGTAACTACATCACCACCCTTGGTGAAGTTACACGTGGTCACATGGGCGGCGCCAACACTATGTACGCAATTGATGCACCTCCTGAACTTTAA

>PS5_P8_b4

ATGGAAAGATCAACTTTGATCAATTTACTTCTGTTACACAAATTTGAACACAAGATTAACACTGAAGGAATCATTGTTGCGCACGGAATTGCTGGAACTGGAAAAACCACATTGCTTAGGACTTTATTTTCTGCTTACCCTAGCTTAGTTATAGGTTCACCTAGGCCTTGTTACTTAGATAAAGCTAATAAAATTTCACAAGTTTGCCTTTCTTGTTTTCCAAATACCTTGTGTGACATTGTTGACGAGTACCATCTCTTAGAAAGTTTTCCTGAACCAAAACTAGCCATTTTTGGTGACCCCTGTCAGTGCACTTACATTGAAAGGTTGAGAACACCCAACTACACATCCTTCAGAACACACCGATTTGGCAAATCCACTGCTGCTCTACTAAACAAGTTATTTGATCTTAACATTGAGTCAGTCAAAGCACAAGACGACACAGTAGAATACTTTGATCCTTTCGCAGTGGACCCCTCTGAACACATTTCTGCTTCAGAAAAAGAAGTTTTGGAATTTGTAGGTGATCAAGTTGAGACTACAAGCTCTGAAGAACTAGCTGGTCTCGAGTTTAGTGAAGTTACTTTCTACTGTACCACACTTGCTGGTGCTGTTCAAGAAAATCCTGCCAAAACCTTCATTTCACTCACTAGACACACTTCAAAGCTCACAATTGGTGAACTAAATGCCAGGTCTGACTCCTAGATGCCAGGTCTGACTCCTAGAGCTGATCTTACTGACACGTATAAAATCATTGCTATAGCCTTTCTACTGTCAGCTTGCATTTACTTCCAAAACAGTCATTATCAACCAGTTGCAGGTGATAATTTGCACAGACTACCCTTTGGTGGTCAGTATCAAGACGGAACTAAGAAGATCTCTTACTTTCCGCAGCAACAATCCTACTTTCACTCAGGAAACAAGCTTAATGTCCTCATACTTATCTTCATTCTTACACTGGGTATTGTCCTCACCAATAAATTTAGTTTTAGCATTAGCCGTAATACTCACCAGCATCATTGCTACAACACACATTCTGCAACCCAAACAGGTCAATCAGTGCCAGGTCATCATTGAATGTCCTCATACTTATCTTCATTCTTACACTGGGTATTGTCCTCACCAATAAATTTAGTTTTAGCATTAGCCGTAATACTCACCAGCATCATTGCTACAACACACATTCTGCAACCCAAACAGGTCAATCAGTGCCAGGTCATCATTGATGGTGCAGCCATAGTCATAACAAATTGTCCAAACACACCCGAAGTTCTTAAAGCAATCAACTTCTCCCCTTGGAACGGGTTAAGTTTTCCTCAATTGTGAATGGAAAACCAACCTACAGCTTCTAACCCATCAGATGTACCACCAACTGCTGCTCAAGCTGGTGCCCAGAGCCCAGCCGACTTCTCAAATCCTAATACAGCTCCTTCCCTAAGTGATTTGAAGAAGATCAAATACGTGTCAACTGTCACTTCAGTTGCCACGCCTGCTGAAATTGAGGCCCTTGGCAAGATCTTTACTGCCATGGGTTTAGCAGCCAATGAGACCGGACCTGCCATGTGGGACCTCGCTCGTGCTTATGCTGATGTGCAAAGTTCAAAATCTGCACAACTTATAGGTGCCACACCATCCAACCCTGCTTTGTCTAGACGTGCACTTGCTGCACAGTTTGATCGTATCAATATCACACCCAGACAATTCTGCATGTATTTTGCAAAAATTGTTTGGAACATACTGTTAGACAGCAATGTGCCACCTGCCAACTGGGCAAAATTGGGCTATCAGGAAGATACCAAGTTTGCTGCTTTTGACTTCTTTGATGGAGTCACAAATCCAGCTAGTCTACAGCCTGCAGATGGCCTAATCAGGCAGCCCAATGAAAAAGAGCTTGCTGCTCACTCGGTTGCTAAATATGGTGCCCTTGCCCGCCAGAAAATATCCACTGGTAACTACATCACCACCCTTGGTGAAGTTACACGTGGTCACATGGGCGGCGCCAACACTATGTACGCAATTGATGCACCTCCTGAACTTTAA

>PS5_P8_b3

ATGGAAAGATCAACTTTGATCAATTTACTTCTGTTACACAAATTTGAACACAAGATTAACACTGAAGGAATCATTGTTGTGCACGGAATTGCTGGAACTGGGAAAACCACATTGCTTAGGACTTTATTTTCTGCTTACCCTAGCTTAGTTATAGGTTCACCTAGGCCTTGTTACTTAGATAAAGCTAATAAAATTTCACAAGTTTGCCTTTCTTGTTTTCCAAATACCTTGTGTGACATTGTTGACGAGTACCATCTCTTAGAAAGTTTTCCTGAACCAAAACTAGCCATTTTTGGTGACCCCTGTCAGTGCACTTACATTGAAAGGTTGAGAACACCCAACTACACATCCTTCAGAGCACACCGATTTGGCAAATCCACTGCTGCTCTACTAAACAAGTTATTTGATCTTAACATTGAGTCAGTCAAAGCACAAGACGACACAGTAGAATACTTTGATCCTTTCGCAGTGGACCCCTCTGAACACATTTCTGCTTCAGAAAAAGAAGTTTTGGAATTTGTAGGTGATCAAGTTGAGACTACAAGCTCTGAAGAACTAGCTGGTCTCGAGTTTAGTGAAGTTACTTTCTACTGTACCACACTTGCTGGTGCTGTTCAAGAAAATCCTGCCAAAACCTTCATTTCACTCACTAGACACACTTCAAAGCTCACAATTGGTGAACTAAATGCCAGGTCTGACTCCTAGATGCCAGGTCTGACTCCTAGAGCTGATCTTACTGACACGTATAAAATCATTGCTATAGCCTTTCTACTGTCAGCTTGCATTTACTTCCAAAACAGTCATTATCAACCAGTTGCAGGTGATAATTTGCACAGACTACCCTTTGGTGGTCAGTATCAAGACGGAACTAAGAAGATCTCTTACTTTCCGCAGCAACAATCCTACTTTCACTCAGGAAACAAGCTTAATGTCCTCATACTTATCTTCATTCTTACACTGGGTATTGTCCTCACCAATAAATTTAGTTTTAGCATTAGCCGTAATACTCACCAGCATCATTGCTACAACACACATTCTGCAACCCAAACAGGTCAATCAGTGCCAGGTCATCATTGAATGTCCTCATACTTATCTTCATTCTTACACTGGGTATTGTCCTCACCAATAAATTTAGTTTTAGCATTAGCCGTAATACTCACCAGCATCATTGCTACAACACACATTCTGCAACCCAAACAGGTCAATCAGTGCCAGGTCATCATTGATGGTGCAGCCATAGTCATAACAAATTGTCCAAACACACCCGAAGTTCTTAAAGCAATCAACTTCTCCCCTTGGAACGGGTTAAGTTTTCCTCAATTGTGAATGGAAAACCAACCTACAGCTTCTAACCCATCAGATGTACCACCAACTGCTGCTCAAGCTGGTGCCCAGAGCCCAGCCGACTTCTCAAATCCTAATACAGCTCCTTCCCTAAGTGATTTGAAGAAGATCAAATACGTGTCAACTGTCACTTCAGTTGCCACGCCTGCTGAAATTGAGGCCCTTGGCAAGATCTTTACTGCCATGGGTTTAGCAGCCAATGAGACCGGACCTGCCATGTGGGACCTCGCTCGTGCTTATGCTGATGTGCAAAGTTCAAAATCTGCACAACTTATAGGTGCCACACCATCCAACCCTGCTTTGTCTAGACGTGCACTTGCTGCACAGTTTGATCGTATCAATATCACACCCAGACAATTCTGCATGTATTTTGCAAAAATTGTTTGGAACATACTGTTAGACAGCAATGTGCCACCTGCCAACTGGGCAAAATTGGGCTATCAGGAAGATACCAAGTTTGCTGCTTTTGACTTCTTTGATGGAGTCACAAATCCAGCTAGTCTACAGCCTGCAGATGGCCTAATCAGGCAGCCCAATGAAAAAGAGCTTGCTGCTCACTCGGTTGCTAAATATGGTGCCCTTGCCCGCCAGAAAATATCCACTGGTAACTACATCACCACCCTTGGTGAAGTTACACGTGGTCACATGGGCGGCGCCAACACTATGTACGCAATTGATGCACCTCCTGAACTTTAA

>PS5_P8_b2

ATGGAAAGATCAACTTTGATCAATTTACTTCTGTTACACAAATTTGAACACAAGATTAACACTGAAGGAATCATTGTTGTGCACGGAATTGCTGGAACTGGGAAAACCACATTGCTTAGGACTTTATTTTCTGCTTACCCTAGCTTAGTTATAGGTTCACCTAGGCCTTGTTACTTAGATAAAGCTAATAAAATTTCACAAGTTTGCCTTTCTTGTTTTCCAAATACCTTGTGTGACATTGTTGACGAGTACCATCTCTTAGAAAGTTTTCCTGAACCAAAACTAGCCATTTTTGGTGACCCCTGTCAGTGCACTTACATTGAAAGGTTGAGAACACCCAACTACACATCCTTCAGAACACACCGATTTGGCAAATCCACTGCTGCTCTACTAAACAAGTTATTTGATCTTAACATTGAGTCAGTCAAAGCACAAGACGACACAGTAGAATACTTTGATCCTTTCGCAGTGGACCCCTCTGAACACATTTCTGCTTCAGAAAAAGAAGTTTTGGAATTTGTAGGTGATCAAGTTGAGACTACAAGCTCTGAAGAACTAGCTGGTCTCGAGTTTAGTGAAGTTACTTTCTACTGTACCACACTTGCTGGTGCTGTTCAAGAAAATCCTGCCAAAACCTTCATTTCACTCACTAGACACACTTCAAAGCTCACAATTGGTGAACTAAATGCCAGGTCTGACTCCTAGATGCCAGGTCTGACTCCTAGAGCTGATCTTACTGACACGTATAAAATCATTGCTATAGCCTTTCTACTGTCAGCTTGCATTTACTTCCAAAACAGTCATTATCAACCAGTTGCAGGTGATAATTTGCACAGACTACCCTTTGGTGGTCAGTATCAAGACGGAACTAAGAAGATCTCTTACTTTCCGCAGCAACAATCCTACTTTCACTCAGGAAACAAGCTTAATGTCCTCATACTTATCTTCATTCTTACACTGGGTATTGTCCTCACCAATAAATTTAGTTTTAGCATTAGCCGTAATACTCACCAGCATCATTGCTACAACACACATTCTGCAACCCAAACAGGTCAATCAGTGCCAGGTCATCATTGAATGTCCTCATACTTATCTTCATTCTTACACTGGGTATTGTCCTCACCAATAAATTTAGTTTTAGCATTAGCCGTAATACTCACCAGCATCATTGCTACAACACACATTCTGCAACCCAAACAGGTCAATCAGTGCCAGGTCATCATTGATGGTGCAGCCATAGTCATAACAAATTGTCCAAACACACCCGAAGTTCTTAAAGCAATCAACTTCTCCCCTTGGAACGGGTTAAGTTTTCCTCAATTGTGAATGGAAAACCAACCTACAGCTTCTAACCCATCAGATGTACCACCAACTGCTGCTCAAGCTGGTGCCCAGAGCCCAGCCGACTTCTCAAATCCTAATACAGCTCCTTCCCTAAGTGATTTGAAGAAGATCAAATACGTGTCAACTGTCACTTCAGTTGCCACGCCTGCTGAAATTGAGGCCCTTGGCAAGATCTTTACTGCCATGGGTTTAGCAGCCAATGAGACCGGACCTGCCATGTGGGACCTCGCTCGTGCTTATGCTGATGTGCAAAGTTCAAAATCTGCACAACTTATAGGTGCCACACCATCCAACCCTGCTTTGTCTAGACGTGCACTTGCTGCACAGTTTGATCGTATCAATACCACACCCAGACAATTCTGCATGTATTTTGCAAAAATTGTTTGGAACATACTGTTAGACAGCAATGTGCCACCTGCCAACTGGGCAAAATTGGGCTATCAGGAAGATACCAAGTTTGCTGCTTTTGACTTCTTTGATGGAGTCACAAATCCAGCTAGTCTACAGCCTGCAGATGGCCTAATCAGGCAGCCCAATGAAAAAGAGCTTGCTGCTCACTCGGTTGCTAAATATGGTGCCCTTGCCCGCCAGAAAATATCCACTGGTAACTACATCACCACCCTTGGTGAAGTTACACGTGGTCACATGGGCGGCGCCAACACTATGTACGCAATTGATGCACCTCCTGAACTTTAA

>PS5_P8_b1

ATGGAAAGATCAACTTTGATCAATTTACTTCTGTTACACAAATTTGAACACAAGATTAACACTGAAGGAATCATTGTTGTGCACGGAATTGCTGGAACTGGGAAAACCACATTGCTTAGGACTTTATTTTCTGCTTACCCTAGCTTAGTTATAGGTTCACCTAGGCCTTGTTACTTAGATAAAGCTAATAAAATTTCACAAGTTTGCCTTTCTTGTTTTCCAAATACCTTGTGTGACATTGTTGACGAGTACCATCTCTTAGAAAGTTTTCCTGAACCAAAACTAGCCATTTTTGGTGACCCCTGTCAGTGCACTTACATTGAAAGGTTGAGAACACCCAACTACACATCCTTCAGAACACACCGATTTGGCAAATCCACTGCTGCTCTACTAAACAAGTTATTTGATCTTAACATTGAGTCAGTCAAAGCACAAGACGACACAGTAGAATACTTTGATCCTTTCGCAGTGGACCCCTCTGAACACATTTCTGCTTCAGAAAAAGAAGTTTTGGAATTTGTAGGTGATCAAGTTGAGACTACAAGCTCTGAAGAACTAGCTGGTCTCGAGTTTAGTGAAGTTACTTTCTACTGTACCACACTTGCTGGTGCTGTTCAAAAAAATCCTGCCAAAACCTTCATTTCACTCACTAGACACACTTCAAAGCTCACAATTGGTGAACTAAATGCCAGGTCTGACTCCTAGATGCCAGGTCTGACTCCTAGAGCTGATCTTACTGACACGTATAAAATCATTGCTATAGCCTTTCTACTGTCAGCTTGCATTTACTTCCAAAACAGTCATTATCAACCAGTTGCAGGTGATAATTTGCACAGACTACCCTTTGGTGGTCAGTATCAAGACGGAACTAAGAAGATCTCTTACTTTCCGCAGCAACAATCCTACTTTCACTCAGGAAACAAGCTTAATGTCCTCATACTTATCTTCATTCTTACACTGGGTATTGTCCTCACCAATAAATTTAGTTTTAGCATTAGCCGTAATACTCACCAGCATCATTGCTACAACACACATTCTGCAACCCAAACAGGTCAATCAGTGCCAGGTCATCATTGAATGTCCTCATACTTATCTTCATTCTTACACTGGGTATTGTCCTCACCAATAAATTTAGTTTTAGCATTAGCCGTAATACTCACCAGCATCATTGCTACAACACACATTCTGCAACCCAAACAGGTCAATCAGTGCCAGGTCATCATTGATGGTGCAGCCATAGTCATAACAAATTGTCCAAACACACCCGAAGTTCTTAAAGCAATCAACTTCTCCCCTTGGAACGGGTTAAGTTTTCCTCAATTGTGAATGGAAAACCAACCTACAGCTTCTAACCCATCAGATGTACCACCAACTGCTGCTCAAGCTGGTGCCCAGAGCCCAGCCGACTTCTCAAATCCTAATACAGCTCCTTCCCTAAGTGATTTGAAGAAGATCAAATACGTGTCAACTGTCACTTCAGTTGCCACGCCTGCTGAAATTGAGGCCCTTGGCAAGATCTTTACTGCCATGGGTTTAGCAGCCAATGAGACCGGACCTGCCATGTGGGACCTCGCTCGTGCTTATGCTGATGTGCAAAGTTCAAAATCTGCACAACTTATAGGTGCCACACCATCCAACCCTGCTTTGTCTAGACGTGCACTTGCTGCACAGTTTGATCGTATCAATATCACACCCAGACAATTCTGCATGTATTTTGCAAAAATTGTTTGGAACATACTGTTAGACAGCAATGTGCCACCTGCCAACTGGGCAAAATTGGGCTATCAGGAAGATACCAAGTTTGCTGCTTTTGACTTCTTTGATGGAGTCACAAATCCAGCTAGTCTACAGCCTGCAGATGGCCTAATCAGGCAGCCCAATGAAAAAGAGCTTGCTGCTCACTCGGTTGCTAAATATGGTGCCCTTGCCCGCCAGAAAATATCCACTGGTAACTACATCACCACCCTTGGTGAAGTTACACGTGGTCACATGGGCGGCGCCAACACTATGTACGCAATTGATGCACCTCCTGAACTTTAA

>PS5_P8_a14

ATGGAAAGATCAACTTTGATCAATTTACTTCTGTTACACAAATTTGAACACAAGATTAACACTGAAGGAATCATTGTTGTGCACGGAATTGCTGGAACTGGGAAAACCACATTGCTTAGGACTTTATTTTCTGCTTACCCTAGCTTAGTTATAGGTTCACCTAGGCCTTGTTACTTAGATAAAGCTAATAAAATTTCACAAGTTTGCCTTTCTTGTTTTCCAAATACCTTGTGTGACATTGTTGACGAGTACCATCTCTTAGAAAGTTTTCCTGAACCAAAACTAGCCATTTTTGGTGACCCCTGTCAGTGCACTTACATTGAAAGGTTGAGAACACCCAACTACACATCCTTCAGAACACACCGATTTGGCAAATCCACTGCTGCTCTACTAAACAAGTTATTTGATCTTAACATTGAGTCAGTCAAAGCACAAGACGACACAGTAGAATACTTTGATCCTTTCGCAGTGGACCCCTCTGAACACATTTCTGCTTCAGAAAAAGAAGTTTTGGAATTTGTAGGTGATCAAGTTGAGACTACAAGCTCTGAAGAACTAGCTGGTCTCGAGTTTAGTGAAGTTACTTTCTACTGTACCACACTTGCTGGTGCTGTTCAAGAAAATCCTGCCAAAACCTTCATTTCACTCACTAGACACACTTCAAAGCTCACAATTGGTGAACTAAATGCCAGGTCTGACTCCTAGATGCCAGGTCTGACTCCTAGAGCTGATCTTACTGACACGTATAAAATCATTGCTATAGCCTTTCTACTGTCAGCTTGCATTTACTTCCAAAACAGTCATTATCAACCAGTTGCAGGTGATAATTTGCACAGACTACCCTTTGGTGGTCAGTATCAAGACGGAACTAAGAAGATCTCTTACTTTCCGCAGCAACAATCCTACTTTCACTCAGGAAACAAGCTTAATGTCCTCATACTTATCTTCATTCTTACACTGGGTATTGTCCTCACCAATAAATTTAGTTTTAGCATTAGCCGTAATACTCACCAGCATCATTGCTACAACACACATTCTGCAACCCAAACAGGTCAATCAGTGCCAGGTCATCATTGAATGTCCTCATACTTATCTTCATTCTTACACTGGGTATTGTCCTCACCAATAAATTTAGTTTTAGCATTAGCCGTAATACTCACCAGCATCATTGCTACAACACACATTCTGCAACCCAAACAGGTCAATCAGTGCCAGGTCATCATTGATGGTGCAGCCATAGTCATAACAAATTGTCCAAACACACCCGAAGTTCTTAAAGCAATCAACTTCTCCCCTTGGAACGGGTTAAGTTTTCCTCAATTGTGAATGGAAAACCAACCTACAGCTTCTAACCCATCAGATGTACCACCAACTGCTGCTCAAGCTGGTGCCCAGAGCCCAGCCGACTTCTCAAATCCTAATACAGCTCCTTCCCTAAGTGATTTGAAGAAGATCAAATACGTGTCAACTGTCACTTCAGTTGCCACGCCTGCTGAAATTGAGGCCCTTGGCAAGATCTTTACTGCCATGGGTTTAGCAGCCAATGAGACCGGACCTGCCATGTGGGACCTCGCTCGTGCTTATGCTGATGTGCAAAGTTCAAAATCTGCACAACTTATAGGTGCCACACCATCCAACCCTGCTTTGTCTAGACGTGCACTTGCTGCACAGTTTGATCGTATCAATATCACACCCAGACAATTCTGCATGTATTTTGCAAAAATTGTTTGGAACATACTGTTAGACAGCAATGTGCCACCTGCCAACTGGGCAAAATTGGGCTATCAGGAAGATACAAAGTTTGCTGCTTTTGACTTCTTTGATGGAGTCACAAATCCAGCTAGTCTACAGCCTGCAGATGGCCTAATCAGGCAGCCCAATGAAAAAGAGCTTGCTGCTCACTCGGTTGCTAAATATGGTGCCCTTGCCCGCCAGAAAATATCCACTGGTAACTACATCACCACCCTTGGTGAAGTTACACGTGGTCACATGGGCGGCGCCAACACTATGTACGCAATTGATGCACCTCCTGAACTTTAA

>PS5_P8_a13

ATGGAAAGATCAACTTTGATCAATTTACTTCTGTTACACAAATTTGAACACAAGATTAACACTGAAGGAATCATTGTTGTGCACGGAATTGCTGGAACTGGGAAAACCACATTGCTTAGGACTTTATTTTCTGCTTACCCTAGCTTAGTTATAGGTTCACCTAGGCCTTGTTACTTAGATAAAGCTAATAAAATTTCACAAGTTTGCCTTTCTTGTTTTCCAAATACCTTGTGTGACATTGTTGACGAGTACCATCTCTTAGAAAGTTTTCCTGAACCAAAACTAGCCATTTTTGGTGACCCCTGTCAGTGCACTTACATTGAAAGGTTGAGAACACCCAACTACACATCCTTCAGAACACACCGATTTGGCAAATCCACTGCTGCTCTACTAAACAAGTTATTTGATCTTAACATTGAGTCAGTCAAAGCACAAGACGACACAGTAGAATACTTTGATCCTTTCGCAGTGGACCCCTCTGAACACATTTCTGCTTCAGAAAAAGAAGTTTTGGAATTTGTAGGTGATCAAGTTGAGACTACAAGCTCTGAAGAACTAGCTGGTCTCGAGTTTAGTGAAGTTACTTTCTACTGTACCACACTTGCTGGTGCTGTTCAAGAAAATCCTGCCAAAACCTTCATTTCACTCACTAGACACACTTCAAAGCTCACAATTGGTGAACTAAATGCCAGGTCTGACTCCTAGATGCCAGGTCTGACTCCTAGAGCTGATCTTACTGACACGTATAAAATCATTGCTATAGCCTTTCTACTGTCAGCTTGCATTTACTTCCAAAACAGTCATTATCAACCAGTTGCAGGTGATAATTTGCACAGACTACCCTTTGGTGGTCAGTATCAAGACGGAACTAAGAAGATCTCTTACTTTCCGCAGCAACAATCCTACTTTCACTCAGGAAACAAGCTTAATGTCCTCATACTTATCTTCATTCTTACACTGGGTATTGTCCTCACCAATAAATTTAGTTTTAGCATTAGCCGTAATACTCACCAGCATCATTGCTACAACACACATTCTGCAACCCAAACAGGTCAATCAGTGCCAGGTCATCATTGAATGTCCTCATACTTATCTTCATTCTTACACTGGGTATTGTCCTCACCAATAAATTTAGTTTTAGCATTAGCCGTAATACTCACCAGCATCATTGCTACAACACACATTCTGCAACCCAAACAGGTCAATCAGTGCCAGGTCATCATTGATGGTGCAGCCATAGTCATAACAAATTGTCCAAACACACCCGAAGTTCTTAAAGCAATCAACTTCTCCCCTTGGAACGGGTTAAGTTTTCCTCAATTGTGAATGGAAAACCAACCTACAGCTTCTAACCCATCAGATGTACCACCAACTGCTGCTCAAGCTGGTGCCCAGAGCCCAGCCGACTTCTCAAATCCTAATACAGCTCCTTCCCTAAGTGATTTGAAGAAGATCAAATACGTGTCAACTGTCACTTCAGTTGCCACGCCTGCTGAAATTGAGGCCCTTGGCAAGATCTTTACTGCCATGGGTTTAGCAGCCAATGAGACCGGACCTGCCATGTGGGACCTCGCTCGTGCTTATGCTGATGTGCAAAGTTCAAAATCTGCACAACTTATAGGTGCCACACCATCCAACCCTGCTTTGTCTAGACGTGCACTTGCTGCACAGTTTGATCGTATCAATATCACACCCAGACAATTCTGCATGTATTTTGCAAAAATTGTTTGGAACATACTGTTAGACAGCAATGTGCCACCTGCCAACTGGGCAAAATTGGGCTATCAGGAAGATACAAAGTTTGCTGCTTTTGACTTCTTTGATGGAGTCACAAATCCAGCTAGTCTACAGCCTGCAGATGGCCTAATCAGGCAGCCCAATGAAAAAGAGCTTGCTGCTCACTCGGTTGCTAAATATGGTGCCCTTGCCCGCCAGAGAATATCCACTGGTAACTACATCACCACCCTTGGTGAAGTTACACGTGGTCACATGGGCGGCGCCAACACTATGTACGCAATTGATGCACCTCCTGAACTTTAA

>PS5_P8_a12

ATGGAAAGATCAACTTTGATCAATTTACTTCTGTTACACAAATTTGAACACAAGATTAACACTGAAGGAATCATTGTTGTGCACGGAATTGCTGGAACTGGGAAAACCACATTGCTTAGGACTTTATTTTCTGCTTACCCTAGCTTAGTTATAGGTTCACCTAGGCCTTGTTACTTAGATAAAGCTAATAAAATTTCACAAGTTTGCCTTTCTTGTTTTCCAAATACCTTGTGTGACATTGTTGACGAGTACCATCTCTTAGAAAGTTTTCCTGAACCAAAACTAGCCATTTTTGGTGACCCCTGTCAGTGCACTTACATTGAAAGGTTGAGAACACCCAACTACACATCCTTCAGAACACACCGATTTGGCAAATCCACTGCTGCTCTACTAAACAAGTTATTTGATCTTAACATTGAGTCAGTCAAAGCACAAGACGACACAGTAGAATACTTTGATCCTTTCGCAGTGGACCCCTCTGAACACATTTCTGCTTCAGAAAAAGAAGTTTTGGAATTTGTAGGTGATCAAGTTGAGACTACAAGCTCTGAAGAACTAGCTGGTCTCGAGTTTAGTGAAGTTACTTTCTACTGTACCACACTTGCTGGTGCTGTTCAAGAAAATCCTGCCAAAACCTTCATTTCACTCACTAGACACACTTCAAAGCTCACAATTGGTGAACTAAATGCCAGGTCTGACTCCTAGATGCCAGGTCTGACTCCTAGAGCTGATCTTACTGACACGTATAAAATCATTGCTATAGCCTTTCTACTGTCAGCTTGCATTTACTTCCAAAACAGTCATTATCAACCAGTTGCAGGTGATAATTTGCACAGACTACCCTTTGGTGGTCAGTATCAAGACGGAACTAAGAAGATCTCTTACTTTCCGCAGCAACAATCCTACTTTCACTCAGGAAACAAGCTTAATGTCCTCATACTTATCTTCATTCTTACACTGGGTATTGTCCTCACCAATAAATTTAGTTTTAGCATTAGCCGTAATACTCACCAGCATCATTGCTACAACACACATTCTGCAACCCAAACAGGTCAATCAGTGCCAGGTCATCATTGAATGTCCTCATACTTATCTTCATTCTTACACTGGGTATTGTCCTCACCAATAAATTTAGTTTTAGCATTAGCCGTAATACTCACCAGCATCATTGCTACAACACACATTCTGCAACCCAAACAGGTCAATCAGTGCCAGGTCATCATTGATGGTGCAGCCATAGTCATAACAAATTGTCCAAACACACCCGAAGTTCTTAAAGCAATCAACTTCTCCCCTTGGAACGGGTTAAGTTTTCCTCAATTGTGAATGGAAAACCAACCTACAGCTTCTAACCCATCAGATGTACCACCAACTGCTGCTCAAGCTGGTGCCCAGAGCCCAGCCGACTTCTCAAATCCTAATACAGCTCCTTCCCTAAGTGATTTGAAGAAGATCAAATACGTGTCAACTGTCACTTCAGTTGCCACGCCTGCTGAAATTGAGGCCCTTGGCAAGATCTTTACTGCCATGGGTTTAGCAGCCAATGAGACCGGACCTGCCATGTGGGACCTCGCTCGTGCTTATGCTGATGTGCAAAGTTCAAAATCTGCACAACTTATAGGTGCCACACCATCCAACCCTGCTTTGTCTAGACGTGCACTTGCTGCACAGTTTGATCGTATCAATATCACACCCAGACAATTCCGCATGTATTTTGCAAAAATTGTTTGGAACATACTGTTAGACAGCAATGTGCCACCTGCCAACTGGGCAAAATTGGGCTATCAGGAAGATACAAAGTTTGCTGCTTTTGACTTCTTTGATGGAGTCACAAATCCAGCTAGTCTACAGCCTGCAGATGGCCTAATCAGGCAGCCCAATGAAAAAGAGCTTGCTGCTCACTCGGTTGCTAAATATGGTGCCCTTGCCCGCCAGAAAATATCCACTGGTAACTACATCACCACCCTTGGTGAAGTTACACGTGGTCACATGGGCGGCGCCAACACTATGTACGCAATTGATGCACCTCCTGAACTTTAA

>PS5_P8_a11

ATGGAAAGATCAACTTTGATCAATTTACTTCTGTTACACAAATTTGAACACAAGATTAACACTGAAGGAATCATTGTTGTGCACGGAATTGCTGGAACTGGGAAAACCACATTGCTTAGGACTTTATTTTCTGCTTACCCTAGCTTAGTTATAGGTTCACCTAGGCCTTGTTACTTAGATAAAGCTAATAAAATTTCACAAGTTTGCCTTTCTTGTTTTCCAAATACCTTGTGTGACATTGTTGACGAGTACCATCTCTTAGAAAGTTTTCCTGAACCAAAACTAGCCATTTTTGGTGACCCCTGTCAGTGCACTTACATTGAAAGGTTGAGAACACCCAACTACACATCCTTCAGAACACACCGATTTGGCAAATCCACTGCTGCTCTACTAAACAAGTTATTTGATCTTAACATTGAGTCAGTCAAAGCACAAGACGACACAGTAGAATACTTTGATCCTTTCGCAGTGGACCCCTCTGAACACATTTCTGCTTCAGAAAAAGAAGTTTTGGAATTTGTAGGTGATCAAGTTGAGACTACAAGCTCTGAAGAACTAGCCGGTCTCGAGTTTAGTGAAGTTACTTTCTACTGTACCACACTTGCTGGTGCTGTTCAAGAAAATCCTGCCAAAACCTTCATTTCACTCACTAGACACACTTCAAAGCTCACAATTGGTGAGCTAAATGCCAGGTCTGACTCCTAGATGCCAGGTCTGACTCCTAGAGCTGATCTTACTGACACGTATAAAATCATTGCTATAGCCTTTCTACTGTCAGCTTGCATTTACTTCCAAAACAGTCATTATCAACCAGTTGCAGGTGATAATTTGCACAGACTACCCTTTGGTGGTCAGTATCAAGACGGAACTAAGAAGATCTCTTACTTTCCGCAGCAACAATCCTACTTTCACTCAGGAAACAAGCTTAATGTCCTCATACTTATCTTCATTCTTACACTGGGTATTGTCCTCACCAATAAATTTAGTTTTAGCATTAGCCGTAATACTCACCAGCATCATTGCTACAACACACATTCTGCAACCCAAACAGGTCAATCAGTGCCAGGTCATCATTGAATGTCCTCATACTTATCTTCATTCTTACACTGGGTATTGTCCTCACCAATAAATTTAGTTTTAGCATTAGCCGTAATACTCACCAGCATCATTGCTACAACACACATTCTGCAACCCAAACAGGTCAATCAGTGCCAGGTCATCATTGATGGTGCAGCCATAGTCATAACAAATTGTCCAAACACACCCGAAGTTCTTAAAGCAATCAACTTCTCCCCTTGGAACGGGTTAAGTTTTCCTCAATTGTGAATGGAAAACCAACCTACAGCTTCTAACCCATCAGATGTACCACCAACTGCTGCTCAAGCTGGTGCCCAGAGCCCAGCCGACTTCTCAAATCCTAATACAGCTCCTTCCCTAAGTGATTTGAAGAAGATCAAATACGTGTCAACTGTCACTTCAGTTGCCACGCCTGCTGAAATTGAGGCCCTTGGCAAGATCTTTACTGCCATGGGTTTAGCAGCCAATGAGACCGGACCTGCCATGTGGGACCTCGCTCGTGCTTATGCTGATGTGCAAAGTTCAAAATCTGCACAACTTATAGGTGCCACACCATCCAACCCTGCTTTGTCTAGACGTGCACTTGCTGCACAGTTTGATCGTATCAATATCACACCCAGACAATTCTGCATGTATTTTGCAAAAATTGTTTGGAACATACTGTTAGACAGCAATGTGCCACCTGCCAACTGGGCAAAATTGGGCTATCAGGAAGATACAAAGTTTGCTGCTTTTGACTTCTTTGATGGAGTCACAAATCCAGCTAGTCTACAGCCTGCAGATGGCCTAATCAGGCAGCCCAATGAAAAAGAGCTTGCTGCTCACTCGGTTGCTAAATATGGTGCCCTTGCCCGCCAGAAAATATCCACTGGTAACTACATCACCACCCTTGGTGAAGTTACACGTGGTCACATGGGCGGCGCCAACACTATGTACGCAATTGATGCACCTCCTGAACTTTAA

>PS5_P8_a10

ATGGAAAGATCAACTTTGATCAATTTACTTCTGTTACACAAATTTGAACACAAGATTAACACTGAAGGAATCATTGTTGTGCACGGAATTGCTGGAACTGGGAAAACCACATTGCTTAGGACTTTATTTTCTGCTTACCCTAGCTTAGTTATAGGTTCACCTAGGCCTTGTTACTTAGATAAAGCTAATAAAATTTCACAAGTTTGCCTTTCTTGTTTTCCAAATACCTTGTGTGACATTGTTGACGAGTACCATCTCTTAGAAAGTTTTCCTGAACCAAAACTAGCCATTTTTGGTGACCCCTGTCAGTGCACTTACATTGAAAGGTTGAGAACACCCAACTACACATCCTTCAGAACACACCGATTTGGCAAATCCACTGCTGCTCTACTAAACAAGTTATTTGATCTTAACATTGAGTCAGTCAAAGCACAAGACGACACAGTAGAATACTTTGATCCTTTCGCAGTGGACCCCTCTGAACACATTTCTGCTTCAGAAAAAGAAGTTTTGGAATTTGTAGGTGATCAAGTTGAGACTACAAGCTCTGAAGAACTAGCTGGTCTCGAGTTTAGTGAAGTTACTTTCTACTGTACCACACTTGCTGGTGCTGTTCAAGAAAATCCTGCCAAAACCTTCATTTCACTCACTAGACACACTTCAAAGCTCACAATTGGTGAACTAAATGCCAGGTCTGACTCCTAGATGCCAGGTCTGACTCCTAGAGCTGATCTTACTGACACGTATAAAATCATTGCTATAGCCTTTCTACTGTCAGCTTGCATTTACTTCCAAAACAGTCATTATCAACCAGTTGCAGGTGATAATTTGCACAGACTACCCTTTGGTGGTCAGTATCAAGACGGAACTAAGAAGATCTCTTACTTTCCGCAGCAACAATCCTACTTTCACTCAGGAAACAAGCTTAATGTCCTCATACTTATCTTCATTCTTACACTGGGTATTGTCCTCACCAATAAATTTAGTTTTAGCATTAGCCGTAATACTCACCAGCATCATTGCTACAACACACATTCTGCAACCCAAACAGGTCAATCAGTGCCAGGTCATCATTGAATGTCCTCATACTTATCTTCATTCTTACACTGGGTATTGTCCTCACCAATAAATTTAGTTTTAGCATTAGCCGTAATACTCACCAGCATCATTGCTACAACACACATTCTGCAACCCAAACAGGTCAATCAGTGCCAGGTCATCATTGATGGTGCAGCCATGGTCATAACAAATTGTCCAAACACACCCGAAGTTCTTAAAGCAATCAACTTCTCCCCTTGGAACGGGTTAAGTTTTCCTCAATTGTGAATGGAAAACCAACCTACAGCTTCTAACCCATCAGATGTACCACCAACTGCTGCTCAAGCTGGTGCCCAGAGCCCAGCCGACTTCTCAAATCCTAATACAGCTCCTTCCCTAAGTGATTTGAAGAAGATCAAATACGTGTCAACTGTCACTTCAGTTGCCACGCCTGCTGAAATTGAGGCCCTTGGCAAGATCTTTACTGCCATGGGTTTAGCAGCCAATGAGACCGGACCTGCCATGTGGGACCTCGCTCGTGCTTATGCTGATGTGCAAAGTTCAAAATCTGCACAACTTATAGGTGCCACACCATCCAACCCTGCTTTGTCTAGACGTGCACTTGCTGCACAGTTTGATCGTATCAATATCACACCCAGACAATTCTGCATGTATTTTGCAAAAATTGTTTGGAACATACTGTTAGACAGCAATGTGCCACCTGCCAACTGGGCAAAATTGGGCTATCAGGAAGATACAAAGTTTGCTGCTTTTGACTTCTTTGATGGAGTCACAAATCCAGCTAGTCTACAGCCTGCAGATGGCCTAATCAGGCAGCCCAATGAAAAAGAGCTTGCTGCTCACTCGGTTGCTAAATATGGTGCCCTTGCCCGCCAGAAAATATCCACTGGTAACTACATCACCACCCTTGGTGAAGTTACACGTGGTCACATGGGCGGCGCCAACACTATGTACGCAATTGATGCACCTCCTGAACTTTAA

>PS5_P8_a9

ATGGAAAGATCAACTTTGATCAATTTACTTCTGTTACACAAATTTGAACACAAGATTAACACTGAAGGAATCATTGTTGTGCACGGAATTGCTGGAACTGGGAAAACCACATTGCTTAGGACTTTATTTTCTGCTTACCCTAGCTTAGTTATAGGTTCACCTAGGCCTTGTTACTTAGATAAAGCTAATAAAATTTCACAAGTTTGCCTTTCTTGTTTTCCAAATACCTTGTGTGACATTGTTGACGAGTACCATCTCTTAGAAAGTTTTCCTGAACCAAAACTAGCCATTTTTGGTGACCCCTGTCAGTGCACTTACATTGAAAGGTTGAGAACACCCAACTACACATCCTTCAGAACACACCGATTTGGCAAATCCACTGCTGCTCTACTAAACAAGTTATTTGATCTTAACATTGAGTCAGTCAAAGCACAAGACGACACAGTAGAATACTTTGATCCTTTCGCAGTGGACCCCTCTGAACACATTTCTGCTTCAGAAAAAGAAGTTTTGGAATTTGTAGGTGATCAAGTTGAGACTACAAGCTCTGAAGAACTAGCTGGTCTCGAGTTTAGTGAAGTTACTTTCTACTGTACCACACTTGCTGGTGCTGTTCAAGAAAATCCTGCCAAAACCTTCATTTCACTCACTAGACACACTTCAAAGCTCACAATTGGTGAACTAAATGCCAGGTCTGACTCCTAGATGCCAGGTCTGACTCCTAGAGCTGATCTTACTGACACGTATAAAATCATTGCTATAGCCTTTCTACTGTCAGCTTGCATTTACTTCCAAAACAGTCATTATCAACCAGTTGCAGGTGATAATTTGCACAGACTACCCTTTGGTGGTCAGTATCAAGACGGAACTAAGAAGATCTCTTACTTTCCGCAGCAACAATCCTACTTTCACTCAGGAAACAAGCTTAATGTCCTCATACTTATCTTCATTCTTACACTGGGTATTGTCCTCACCAATAAATTTAGTTTTAGCATTAGCCGTAATACTCACCAGCATCATTGCTACAACACACATTCTGCAACCCAAACAGGTCAATCAGTGCCAGGTCATCATTGAATGTCCTCATACTTATCTTCATTCTTACACTGGGTATTGTCCTCACCAATAAATTTAGTTTTAGCATTAGCCGTAATACTCACCAGCATCATTGCTACAACACACATTCTGCAACCCAAACAGGTCAATCAGTGCCAGGTCATCATTGATGGTGCAGCCATAGTCATAACAAATTGTCCAAACACACCCGAAGTTCTTAAAGCAATCAACTTCTCCCCTTGGAACGGGTTAAGTTTTCCTCAATTGTGAATGGAAAACCAACCTACAGCTTCTAACCCATCAGATGTACCACCAACTGCTGCTCAAGCTGGTGCCCAGAGCCCAGCCGACTTCTCAAATCCTAATACAGCTCCTTCCCTAAGTGATTTGAAGAAGATCAAATACGTGTCAACTGTCACTTCAGTTGCCACGCCTGCTGAAATTGAGGCCCTTGGCAAGATCTTTACTGCCATGGGTTTAGCAGCCAATGAGACCGGACCTGCCATGTGGGACCTCGCTCGTGCTTATGCTGATGTGCAAAGTTCAAAATCTGCACAACTTATAGGTGCCACACCATCCAACCCTGCTTTGTCTAGACGTGCACTTGCTGCACAGTTTGATCGTATCAATATCACACCCAGACAATTCTGCATGTATTTTGCAAAAATTGTTTGGAACATACTGTTAGACAGCAATGTGCCACCTGCCAACTGGGCAAAATTGGGCTATCAGGAAGATACAAAGTTTGCTGCTTTTGACTTCTTTGATGGAGTCACAAATCCAGCTAGTCTACGGCCTGCAGATGGCCTAATCAGGCAGCCCAATGAAAAAGAGCTTGCTGCTCACTCGGTTGCTAAATATGGTGCCCTTGCCCGCCAGAAAATATCCACTGGTAACTACATCACCACCCTTGGTGAAGTTACACGTGGTCACATGGGCGGCGCCAACACTATGTACGCAATTGATGCACCTCCTGAACTTTAA

>PS5_P8_a8

ATGGAAAGATCAACTTTGATCAATTTACTTCTGTTACACAAATTTGAACACAAGATTAACACTGAAGGAATCATTGTTGTGCACGGAATTGCTGGAACTGGGAAAACCACATTGCTTAGGACTTTATTTTCTGCTTACCCTAGCTTAGTTATAGGTTCACCTAGGCCTTGTTACTTAGATAAAGCTAATAAAATTTCACAAGTTTGCCTTTCTTGTTTTCCAAATACCTTGTGTGACATTGTTGACGAGTACCATCTCTTAGAAAGTTTTCCTGAGCCAAAACTAGCCATTTTTGGTGACCCCTGTCAGTGCACTTACATTGAAAGGTTGAGAACACCCAACTACACATCCTTCAGAACACACCGATTTGGCAAATCCACTGCTGCTCTACTAAACAAGTTATTTGATCTTAACATTGAGTCAGTCAAAGCACAAGACGACACAGTAGAATACTTTGATCCTTTCGCAGTGGACCCCTCTGAACACATTTCTGCTTCAGAAAAAGAAGTTTTGGAATTTGTAGGTGATCAAGTTGAGACTACAAGCTCTGAAGAACTAGCTGGTCTCGAGTTTAGTGAAGTTACTTTCTACTGTACCACACTTGCTGGTGCTGTTCAAGAAAATCCTGCCAAAACCTTCATTTCACTCACTAGACACACTTCAAAGCTCACAATTGGTGAACTAAATGCCAGGTCTGACTCCTAGATGCCAGGTCTGACTCCTAGAGCTGATCTTACTGACACGTATAAAATCATTGCTATAGCCTTTCTACTGTCAGCTTGCATTTACTTCCAAAACAGTCATTATCAACCAGTTGCAGGTGATAATTTGCACAGACTACCCTTTGGTGGTCAGTATCAAGACGGAACTAAGAAGATCTCTTACTTTCCGCAGCAACAATCCTACTTTCACTCAGGAAACAAGCTTAATGTCCTCATACTTATCTTCATTCTTACACTGGGTATTGTCCTCACCAATAAATTTAGTTTTAGCATTAGCCGTAATACTCACCAGCATCATTGCTACAACACACATTCTGCAACCCAAACAGGTCAATCAGTGCCAGGTCATCATTGAATGTCCTCATACTTATCTTCATTCTTACACTGGGTATTGTCCTCACCAATAAATTTAGTTTTAGCATTAGCCGTAATACTCACCAGCATCATTGCTACAACACACATTCTGCAACCCAAACAGGTCAATCAGTGCCAGGTCATCATTGATGGTGCAGCCATAGTCATAACAAATTGTCCAAACACACCCGAAGTTCTTAAAGCAATCAACTTCTCCCCTTGGAACGGGTTAAGTTTTCCTCAATTGTGAATGGAAAACCAACCTACAGCTTCTAACCCATCAGATGTACCACCAACTGCTGCTCAAGCTGGTGCCCAGAGCCCAGCCGACTTCTCAAATCCTAATACAGCTCCTTCCCTAAGTGATTTGAAGAAGATCAAATACGTGTCAACTGTCACTTCAGTTGCCACGCCTGCTGAAATTGAGGCCCTTGGCAAGATCTTTACTGCCATGGGTTTAGCAGCCAATGAGACCGGACCTGCCATGTGGGACCTCGCTCGTGCTTATGCTGATGTGCAAAGTTCAAAATCTGCACAACTTATAGGTGCCACACCATCCAACCCTGCTTTGTCTAGACGTGCACTTGCTGCACAGTTTGATCGTATCAATATCACACCCAGACAATTCTGCATGTATTTTGCAAAAATTGTTTGGAACATACTGTTAGACAGCAATGTGCCACCTGCCAACTGGGCAAAATTGGGCTATCAGGAAGATACAAAGTTTGCTGCTTTTGACTTCTTTGATGGAGTCACAAATCCAGCTAGTCTACAGCCTGCAGATGGCCTAATCAGGCAGCCCAATGAAAAAGAGCTTGCTGCTCACTCGGTTGCTAAATATGGTGCCCTTGCCCGCCAGAAAATATCCACTGGTAACTACATCACCACCCTTGGTGAAGTTACACGTGGTCACATGGGCGGCGCCAACACTATGTACGCAATTGATGCACCTCCTGAACTTTAA

>PS5_P8_a7

ATGGAAAGATCAACTTTGATCAATTTACTTCTGTTACACAAATTTGAACACAAGATTAACACTGAAGGAATCATTGTTGTGCACGGAATTGCTGGAACTGGGAAAACCACATTGCTTAGGACTTTATTTTCTGCTTACCCTAGCTTAGTTATAGGTTCACCTAGGCCTTGTTACTTAGATAAAGCTAATAAAATTTCACAAGTTTGCCTTTCTTGTTTTCCAAATACCTTGTGTGACATTGTTGACGAGTACCATCTCTTAGAAAGTTTTCCTGAACCAAAACTAGCCATTTTTGGTGACCCCTGTCAGTGCACTTACATTGAAAGGTTGAGAACACCCAACTACACATCCTTCAGAACACACCGATTTGGCAAATCCACTGCTGCTCTACTAAACAAGTTATTTGATCTTAACATTGAGTCAGTCAAAGCACAAGACGACACAGTAGAATACTTTGATCCTTTCGCAGTGGACCCCTCTGAACACATTTCTGCTTCAGAAAAAGAAGTTTTGGAATTTGTAGGTGATCAAGTTGAGACTACAAGCTCTGAAGAACTAGCTGGTCTCGAGTTTAGTGAAGTTACTTTCTACTGTACCACACTTGCTGGTGCTGTTCAAGAAAATCCTGCCAAAACCTTCATTTCACTCACTAGACACACTTCAAAGCTCACAATTGGTGAACTAAATGCCAGGTCTGACTCCTAGATGCCAGGTCTGACTCCTAGAGCTGATCTTACTGACACGTATAAAATCATTGCTATAGCCTTTCTACTGTCAGCTTGCATTTACTTCCAAAACAGTCATTATCAACCAGTTGCAGGTGATAATTTGCACAGACTACCCTTTGGTGGTCAGTATCAAGACGGAACTAAGAAGATCTCTTACTTTCCGCAGCAACAATCCTACTTTCACTCAGGAAACAAGCTTAATGTCCTCATACTTATCTTCATTCTTACACTGGGTATTGTCCTCACCAATAAATTTAGTTTTAGCATTAGCCGTAATACTCACCAGCATCATTGCTACAACACACATTCTGCAACCCAAACAGGTCAATCAGTGCCAGGTCATCATTGAATGTCCTCATACTTATCTTCATTCTTACACTGGGTATTGTCCTCACCAATAAATTTAGTTTTAGCATTAGCCGTAATACTCACCAGCATCATTGCTACAACACACATTCTGCAACCCAAACAGGTCAATCAGTGCCAGGTCATCATTGATGGTGCAGCCATAGTCATAACAAATTGTCCAAACACACCCGAAGTTCTTAAAGCAATCAACTTCTCCCCTTGGAACGGGTTAAGTTTTCCTCAATTGTGAATGGAAAACCAACCTACAGCTTCTAACCCATCAGATGTACCACCAACTGCTGCTCAAGCTGGTGCCCAGAGCCCAGCCGACTTCTCAAATCCTAATACAGCTCCTTCCTTAAGTGATTTGAAGAAGATCAAATACGTGTCAACTGTCACTTCAGTTGCCACGCCTGCTGAAATTGAGGCCCTTGGCAAGATCTTTACTGCCATGGGTTTAGCAGCCAATGAGACCGGACCTGCCATGTGGGACCTCGCTCGTGCTTATGCTGATGTGCAAAGTTCAAAATCTGCACAACTTATAGGTGCCACACCATCCAACCCTGCTTTGTCTAGACGTGCACTTGCTGCACAGTTTGATCGTATCAATATCACACCCAGACAATTCTGCATGTATTTTGCAAAAATTGTTTGGAACATACTGTTAGACAGCAATGTGCCACCTGCCAACTGGGCAAAATTGGGCTATCAGGAAGATACAAAGTTTGCTGCTTTTGACTCCTTTGATGGAGTCACAAATCCAGCTAGTCTACAGCCTGCAGATGGCCTAATCAGGCAGCCCAATGAAAAAGAGCTTGCTGCTCACTCGGTTGCTAAATATGGTGCCCTTGCCCGCCAGAAAATATCCACTGGTAACTACATCACCACCCTTGGTGAAGTTACACGTGGTCACATGGGCGGCGCCAACACTATGTACGCAATTGATGCACCTCCTGAACTTTAA

>PS5_P8_a6

ATGGAAAGATCAACTTTGATCAATTTACTTCTGTTACACAAATTTGAACACAAGATTAACACTGAAGGAATCATTGTTGTGCACGGAATTGCTGGAACTGGGAAAACCACATTGCTTAGGACTTTATTTTCTGCTTACCCTAGCTTAGTTATAGGTTCACCTAGGCCTTGTTACTTAGATAAAGCTAATAAAATTTCACAAGTTTGCCTTTCTTGTTTTCCAAATACCTTGTGTGACATTGTTGACGAGTACCATCTCTTAGAAAGTTTTCCTGAACCAAAACTAGCCATTTTTGGTGACCCCTGTCAGTGCACTTACATTGAAAGGTTGAGAACACCCAACTACACATCCTTCAGAACACACCGATTTGGCAAATCCACTGCTGCTCTACTAAACAAGTTATTTGATCTTAACATTGAGTCAGTCAAAGCACAAGACGACACAGTAGAATACTTTGATCCTTTCGCAGTGGACCCCTCTGAACACATTTCTGCTTCAGAAAAAGAAGTTTTGGAATTTGTAGGTGATCAAGTTGAGACTACAAGCTCTGAAGAACTAGCTGGTCTCGAGTTTAGTGAAGTTACTTTCTACTGTACCACACTTGCTGGTGCTGTTCAAGAAAATCCTGCCAAAACCTTCATTTCACTCACTAGACACACTTCAAAGCTCACAATTGGTGAACTAAATGCCAGGTCTGACTCCTAGATGCCAGGTCTGACTCCTAGAGCTGATCTTACTGACACGTATAAAATCATTGCTATAGCCTTTCTACTGTCAGCTTGCATTTACTTCCAAAACAGTCATTATCAACCAGTTGCAGGTGATAATTTGCACAGACTACCCTTTGGTGGTCAGTATCAAGACGGAACTAAGAAGATCTCTTACTTTCCGCAGCAACAATCCTACTTTCACTCAGGAAACAAGCTTAATGTCCTCATACTTATCTTCATTCTTACACTGGGTATTGTCCTCACCAATAAATTTAGTTTTAGCATTAGCCGTAATACTCACCAGCATCATTGCTACAACACACATTCTGCAACCCAAACAGGTCAATCAGTGCCAGGTCATCATTGAATGTCCTCATACTTATCTTCATTCTTACACTGGGTATTGTCCTCACCAATAAATTTAGTTTTAGCATTAGCCGTAATACTCACCAGCATCATTGCTACAACACACATTCTGCAACCCAAACAGGTCAATCAGTGCCAGGTCATCATTGATGGTGCAGCCATAGTCATAACAAATTGTCCAAACACACCCGAAGTTCTTAAAGCAATCAACTTCTCCCCTTGGAACGGGTTAAGTTTTCCTCAATTGTGAATGGAAAACCAACCTACAGCTTCTAACCCATCAGATGTACCACCAACTGCTGCTCAAGCTGGTGCCCAGAGCCCAGCCGACTTCTCAAATCCTAATACAGCTCCTTCCCTAAGTGATTTGAAGAAGATCAAATACGTGTCAACTGTCACTTCAGTTGCCACGCCTGCTGAAATTGAGGCCCTTGGCAAGATCTTTACTGCCATGGGTTTAGCAGCCAATGAGACCGGACCTGCCATGTGGGACCTCGCTCGTGCTTATGCTGATGTGCAAAGTTCAAAATCTGCACAACTTATAGGTGCCACACCATCCAACCCTGCTTTGTCTAGACGTGCACTTGCTGCACAGTTTGATCGTATCAATATCACACCCAGACAATTCTGCATGTATTTTGCAAAAATTGTTTGGAACATACTGTTAGACAGCAATGTGCCACCTGCCAACTGGGCAAAATTGGGCTATCAGGAAGATACAAAGTTTGCTGCTTTTGACTTCTTTGATGGAGTCACAAATCCAGCTAGTCTACAGCCTGCAGATGGCCTAATCAGGCAGCCCAATGAAAAAGAGCTTGCTGCTCACTCGGTTGCTAAATATGGTGCCCTTGCCCGCCAGAAAATATCCACTGGTAACTACATCACCACCCTTGGTGAAGTTACACGTGGTCACATGGGCGGCGCCAACACTATGTACGCAATTGATGCACCTCCTGAACTTTAA

>PS5_P8_a5

ATGGAAAGATCAACTTTGATCAATTTACTTCCGTTACACAAATTTGAACACAAGATTAACACTGAAGGAATCATTGTTGTGCACGGAATTGCTGGAACTGGGAAAACCACATTGCTTAGGACTTTATTTTCTGCTTACCCTAGCTTAGTTATAGGTTCACCTAGGCCTTGTTACTTAGATAAAGCTAATAAAATTTCACAAGTTTGCCTTTCTTGTTTTCCAAATACCTTGTGTGACATTGTTGACGAGTACCATCTCTTAGAAAGTTTTCCTGAACCAAAACTAGCCATTTTTGGTGACCCCTGTCAGTGCACTTACATTGAAAGGTTGAGAACACCCAACTACACATCCTTCAGAACACACCGATTTGGCAAATCCACTGCTGCTCTACTAAACAAGTTATTTGATCTTAACATTGAGTCAGTCAAAGCACAAGACGACACAGTAGAATACTTTGATCCTTTCGCAGTGGACCCCTCTGAACACATTTCTGCTTCAGAAAAAGAAGTTTTGGAATTTGTAGGTGATCAAGTTGAGACTACAAGCTCTGAAGAACTAGCTGGTCTCGAGTTTAGTGAAGTTACTTTCTACTGTACCACACTTGCTGGTGCTGTTCAAGAAAATCCTGCCAAAACCTTCATTTCACTCACTAGACACACTTCAAAGCTCACAATTGGTGAACTAAATGCCAGGTCTGACTCCTAGATGCCAGGTCTGACTCCTAGAGCTGATCTTACTGACACGTATAAAATCATTGCTATAGCCTTTCTACTGTCAGCTTGCATTTACTTCCAAAACAGTCATTATCAACCAGTTGCAGGTGATAATTTGCACAGACTACCCTTTGGTGGTCAGTATCAAGACGGAACTAAGAAGATCTCTTACTTTCCGCAGCAACAATCCTACTTTCACTCAGGAAACAAGCTTAATGTCCTCATACTTATCTTCATTCTTACACTGGGTATTGTCCTCACCAATAAATTTAGTTTTAGCATTAGCCGTAATACTCACCAGCATCATTGCTACAACACACATTCTGCAACCCAAACAGGTCAATCAGTGCCAGGTCATCATTGAATGTCCTCATACTTATCTTCATTCTTACACTGGGTATTGTCCTCACCAATAAATTTAGTTTTAGCATTAGCCGTAATACTCACCAGCATCATTGCTACAACACACATTCTGCAACCCAAACAGGTCAATCAGTGCCAGGTCATCATTGATGGTGCAGCCATAGTCATAACAAATTGTCCAAACACACCCGAAGTTCTTAAAGCAATCAACTTCTCCCCTTGGAACGGGTTAAGTTTTCCTCAATTGTGAATGGAAAACCAACCTACAGCTTCTAACCCATCAGATGTACCACCAACTGCTGCTCAAGCTGGTGCCCAGAGCCCAGCCGACTTCTCAAATCCTAATACAGCTCCTTCCCTAAGTGATTTGAAGAAGATCAAATACGTGTCAACTGTCACTTCAGTTGCCACGCCTGCTGAAATTGAGGCCCTTGGCAAGATCTTTACTGCCATGGGTTTAGCAGCCAATGAGACCGGACCTGCCATGTGGGACCTCGCTCGTGCTTATGCTGATGTGCAAAGTTCAAAATCTGCACAACTTATAGGTGCCACACCATCCAACCCTGCTTTGTCTAGACGTGCACTTGCTGCACAGTTTGATCGTATCAATATCACACCCAGACAATTCTGCATGTATTTTGCAAAAATTGTTTGGAACATACTGTTAGACAGCAATGTGCCACCTGCCAACTGGGCAAAATTGGGCTATCAGGAAGATACAAAGTTTGCTGCTTTTGACTTCTTTGATGGAGTCACAAATCCAGCTAGTCTACAGCCTGCAGATGGCCTAATCAGGCAGCCCAATGAAAAAGAGCTTGCTGCTCACTCGGTTGCTAAATATGGTGCCCTTGCCCGCCAGAAAATATCCACTGGTAACTACATCACCACCCTTGGTGAAGTTACACGTGGTCACATGGGCGGCGCCAACACTATGTACGCAATTGATGCACCTCCTGAACTTTAA

>PS5_P8_a4

ATGGAAAGATCAACTTTGATCAATTTACTTCTGTTACACAAATTTGAACACAAGATTAACACTGAAGGAATCATTGTTGTGCACGGAATTGCTGGAACTGGGAAAACCACATTGCTTAGGACTTTATTTTCTGCTTACCCTAGCTTAGTTATAGGTTCACCTAGGCCTTGTTACTTAGATAAAGCTAATAAAATTTCACAAGTTTGCCTTTCTTGTTTTCCAAATACCTTGTGTGACATTGTTGACGAGTACCATCTCTTAGAAAGTTTTCCTGAACCAAAACTAGCCATTTTTGGTGACCCCTGTCAGTGCACTTACATTGAAAGGTTGAGAACACCCAACTACACATCCTTCAGAACACACCGATTTGGCAAATCCACTGCTGCTCTACTAAACAAGTTATTTGATCTTAACATTGAGTCAGTCAAAGCACAAGACGACACAGTAGAATACTTTGATCCTTTCGCAGTGGACCCCTCTGAACACATTTCTGCTTCAGAAAAAGAAGTTTTGGAATTTGTAGGTGATCAAGTTGAGACTACAAGCTCTGAAGAACTAGCTGGTCTCGAGTTTAGTGAAGTTACTTTCTACTGTACCACACTTGCTGGTGCTGTTCAAGAAAATCCTGCCAAAACCTTCATTTCACTCACTAGACACACTTCAAAGCTCACAATTGGTGAACTAAATGCCAGGTCTGACTCCTAGATGCCAGGTCTGACTCCTAGAGCTGATCTTACTGACACGTATAAAATCATTGCTATAGCCTTTCTACTGTCAGCTTGCATTTACTTCCAAAACAGTCATTATCAACCAGTTGCAGGTGATAATTTGCACAGACTACCCTTTGGTGGTCAGTATCAAGACGGAACTAAGAAGATCTCTTACTTTCCGCAGCAACAATCCTACTTTCACTCAGGAAACAAGCTTAATGTCCTCATACTTATCTTCATTCTTACACTGGGTATTGTCCTCACCAATAAATTTAGTTTTAGCATTAGCCGTAATACTCACCAGCATCATTGCTACAACACACATTCTGCAACCCAAACAGGTCAATCAGTGCCAGGTCATCATTGAATGTCCTCATACTTATCTTCATTCTTACACTGGGTATTGTCCTCACCAATAAATTTAGTTTTAGCATTAGCCGTAATACTCACCAGCATCATTGCTACAACACACATTCTGCAACCCAAACAGGTCAATCAGTGCCAGGTCATCATTGATGGTGCAGCCATAGTCATAACAAATTGTCCAAACACACCCGAAGTTCTTAAAGCAATCAACTTCTCCCCTTGGAACGGGTTAAGTTTTCCTCAATTGTGAATGGAAAACCAACCTACAGCTTCTAACCCATCAGATGTACCACCAACTGCTGCTCAAGCTGGTGCCCAGAGCCCAGCCGACTTCTCAAATCCTAATACAGCTCCTTCCCTAAGTGATTTGAAGAAGATCAAATACGTGTCAACTGTCACTTCAGTTGCCACGCCTGCTGAAATTGAGGCCCTTGGCAAGATCTTTACTGCCATGGGTTTAGCAGCCAATGAGACCGGACCTGCCATGTGGGACCTCGCTCGTGCTTATGCTGATGTGCAAAGTTCAAAATCTGCACAACTTATAGGTGCCACACCATCCAACCCTGCTTTGTCTAGACGTGCACTTGCTGCACAGTTTGATCGTATCAATATCACACCCAGACAATTCTGCATGTATTTTGCAAAAATTGTTTGGAACATACTGTTAGACAGCAATGTGCCACCTGCCAACTGGGCAAAATTGGGCTATCAGGAAGATACAAAGTTTGCTGCTTTTGACTTCTTTGATGGAGTCACAAATCCAGCTAGTCTACAGCCTGCAGATGGCCTAATCAGGCAGCCCAATGAAAAAGAGCTTGCTGCTCACTCGGTTGCTAAATATGGTGCCCTTGCCCGCCAGAAAATATCCACTGGTAACTACATCACCACCCTTGGTGAAGTTACACGTGGTCACATGGGCGGCGCCAACACTATGTACGCAATTGATGCACCTCCTGAACTTTAA

>PS5_P8_a3

ATGGAAAGATCAACTTTGATCAATTTACTTCCGTTACACAAATTTGAACACAAGATTAACACTGAAGGAATCATTGTTGTGCACGGAATTGCTGGAACTGGGAAAACCACATTGCTTAGGACTTTATTTTCTGCTTACCCTAGCTTAGTTATAGGTTCACCTAGGCCTTGTTACTTAGATAAAGCTAATAAAATTTCACAAGTTTGCCTTTCTTGTTTTCCAAATACCTTGTGTGACATTGTTGACGAGTACCATCTCTTAGAAAGTTTTCCTGAACCAAAACTAGCCATTTTTGGTGACCCCTGTCAGTGCACTTACATTGAAAGGTTGAGAACACCCAACTACACATCCTTCAGAACACACCGATTTGGCAAATCCACTGCTGCTCTACTAAACAAGTTATTTGATCTTAACATTGAGTCAGTCAAAGCACAAGACGACACAGTAGAATACTTTGATCCTTTCGCAGTGGACCCCTCTGAACACATTTCTGCTTCAGAAAAAGAAGTTTTGGAATTTGTAGGTGATCAAGTTGAGACTACAAGCTCTGAAGAACTAGCTGGTCTCGAGTTTAGTGAAGTTACTTTCTACTGTACCACACTTGCTGGTGCTGTTCAAGAAAATCCTGCCAAAACCTTCATTTCACTCACTAGACACACTTCAAAGCTCACAATTGGTGAACTAAATGCCAGGTCTGACTCCTAGATGCCAGGTCTGACTCCTAGAGCTGATCTTACTGACACGTATAAAATCATTGCTATAGCCTTTCTACTGTCAGCTTGCATTTACTTCCAAAACAGTCATTATCAACCAGTTGCAGGTGATAATTTGCACAGACTACCCTTTGGTGGTCAGTATCAAGACGGAACTAAGAAGATCTCTTACTTTCCGCAGCAACAATCCTACTTTCACTCAGGAAACAAGCTTAATGTCCTCATACTTATCTTCATTCTTACACTGGGTATTGTCCTCACCAATAAATTTAGTTTTAGCATTAGCCGTAATACTCACCAGCATCATTGCTACAACACACATTCTGCAACCCAAACAGGTCAATCAGTGCCAGGTCATCATTGAATGTCCTCATACTTATCTTCATTCTTACACTGGGTATTGTCCTCACCAATAAATTTAGTTTTAGCATTAGCCGTAATACTCACCAGCATCATTGCTACAACACACATTCTGCAACCCAAACAGGTCAATCAGTGCCAGGTCATCATTGATGGTGCAGCCATAGTCATAACAAATTGTCCAAACACACCCGAAGTTCTTAAAGCAATCAACTTCTCCCCTTGGAACGGGTTAAGTTTTCCTCAATTGTGAATGGAAAACCAACCTACAGCTTCTAACCCATCAGATGTACCACCAACTGCTGCTCAAGCTGGTGCCCAGAGCCCAGCCGACTTCTCAAATCCTAATACAGCTCCTTCCCTAAGTGATTTGAAGAAGATCAAATACGTGTCAACTGTCACTTCAGTTGCCACGCCTGCTGAAATTGAGGCCCTTGGCAAGATCTTTACTGCCATGGGTTTAGCAGCCAATGAGACCGGACCTGCCATGTGGGACCTCGCTCGTGCTTATGCTGATGTGCAAAGTTCAAAATCTGCACAACTTATAGGTGCCACACCATCCAACCCTGCTTTGTCTAGACGTGCACTTGCTGCACAGTTTGATCGTATCAATATCACACCCAGACAATTCTGCATGTATTTTGCAAAAATTGTTTGGAACATACTGTTAGACAGCAATGTGCCACCTGCCAACTGGGCAAAATTGGGCTATCAGGAAGATACAAAGTTTGCTGCTTTTGACTTCTTTGATGGAGTCACAAATCCAGCTAGTCTACAGCCTGCAGATGGCCTAATCAGGCAGCCCAATGAAAAAGAGCTTGCTGCTCACTCGGTTGCTAAATATGGTGCCCTTGCCCGCCAGAAAATATCCACTGGTAACTACATCACCACCCTTGGTGAAGTTACACGTGGTCACATGGGCGGCGCCAACACTATGTACGCAATTGATGCACCTCCTGAACTTTAA

>PS5_P8_a2

ATGGAAAGATCAACTTTGATCAATTTACTTCTGTTACACAAATTTGAACACAAGATTAACACTGAAGGAATCATTGTTGTGCACGGAATTGCTGGAACTGGGAAAACCACATTGCTTAGGACTTTATTTTCTGCTTACCCTAGCTTAGTTATAGGTTCACCTAGGCCTTGTTACTTAGATAAAGCTAATAAAATTTCACAAGTTTGCCTTTCTTGTTTTCCAAATACCTTGTGTGACATTGTTGACGAGTACCATCTCTTAGAAAGTTTTCCTGAACCAAAACTAGCCATTTTTGGTGACCCCTGTCAGTGCACTTACATTGAAAGGTTGAGAACACCCAACTACACATCCTTCAGAACACACCGATTTGGCAAATCCACTGCTGCTCTACTAAACAAGTTATTTGATCTTAACATTGAGTCAGTCAAAGCACAAGACGACACAGTAGAATACTTTGATCCTTTCGCAGTGGACCCCTCTGAACACATTTCTGCTTCAGAAAAAGAAGTTTTGGAATTTGTAGGTGATCAAGTTGAGACTACAAGCTCTGAAGAACTAGCTGGTCTCGAGTTTAGTGAAGTTACTTTCTACTGTACCACACTTGCTGGTGCTGTTCAAGAAAATCCTGCCAAAACCTTCATTTCACTCACTAGACACACTTCAAAGCTCACAATTGGTGAACTAAATGCCAGGTCTGACTCCTAGATGCCAGGTCTGACTCCTAGAGCTGATCTTACTGACACGTATAAAATCATTGCTATAGCCTTTCTACTGTCAGCTTGCATTTACTTCCAAAACAGTCATTATCAACCAGTTGCAGGTGATAATTTGCACAGACTACCCTTTGGTGGTCAGTATCAAGACGGAACTAAGAAGATCTCTTACTTTCCGCAGCAACAATCCTACTTTCACTCAGGAAACAAGCTTAATGTCCTCATACTTATCTTCATTCTTACACTGGGTATTGTCCTCACCAATAAATTTAGTTTTAGCATTAGCCGTAATACTCACCAGCATCATTGCTACAACACACATTCTGCAACCCAAACAGGTCAATCAGTGCCAGGTCATCATTGAATGTCCTCATACTTATCTTCATTCTTACACTGGGTATTGTCCTCACCAATAAATTTAGTTTTAGCATTAGCCGTAATACTCACCAGCATCATTGCTACAACACACATTCTGCAACCCAAACAGGTCAATCAGTGCCAGGTCATCATTGATGGTGCAGCCATAGTCATAACAAATTGTCCAAACACACCCGAAGTTCTTAAAGCAATCAACTTCTCCCCTTGGAACGGGTTAAGTTTTCCTCAATTGTGAATGGAAAACCAACCTACAGCTTCTAACCCATCAGATGTACCACCAACTGCTGCTCAAGCTGGTGCCCAGAGCCCAGCCGACTTCTCAAATCCTAATACAGCTCCTTCCCTAAGTGATTTGAAGAAGATCAAATACGTGTCAACTGTCACTTCAGTTGCCACGCCTGCTGAAATTGAGGCCCTTGGCAAGATCTTTACTGCCATGGGTTTAGCAGCCAATGAGACCGGACCTGCCATGTGGGACCTCGCTCGTGCTTATGCTGATGTGCAAAGTTCAAAATCTGCACAACTTATAGGTGCCACACCATCCAACCCTGCTTTGTCTAGACGTGCACTTGCTGCACAGTTTGATCGTATCAATATCACACCCAGACAATTCTGCATGTATTTTGCAAAAATTGTTTGGAACATACTGTTAGACAGCAATGTGCCACCTGCCAACTGGGCAAAATTGGGCTATCAGGAAGATACAAAGTTTGCTGCTTTTGACTTCTTTGATGGAGTCACAAATCCAGCTAGTCTACAGCCTGCAGATGGCCTAATCAGGCAGCCCAATGAAAAAGAGCTTGCTGCTCACTCGGTTGCTAAATATGGTGCCCTTGCCCGCCAGAAAATATCCACTGGTAACTACATCACCACCCTTGGTGAAGTTACACGTGGTCACATGGGCGGCGCCAACACTATGTACGCAATTGATGCACCTCCTGAACTTTAA

>PS5_P8_a1

ATGGAAAGATCAACTTTGATCAATTTACTTCTGTTACACAAATTTGAACACAAGATTAACACTGAAGGAATCATTGTTGTGCACGGAATTGCTGGAACTGGGAAAACCACATTGCTTAGGACTTTATTTTCTGCTTACCCTAGCTTAGTTATAGGTTCACCTAGGCCTTGTTACTTAGATAAAGCTAATAAAATTTCACAAGTTTGCCTTTCTTGTTTTCCAAATACCTTGTGTGACATTGTTGACGAGTACCATCTCTTAGAAAGTTTTCCTGAACCAAAACTAGCCATTTTTGGTGACCCCTGTCAGTGCACTTACATTGAAAGGTTGAGAACACCCAACTACACATCCTTCAGAACACACCGATTTGGCAAACCCACTGCTGCTCTACTAAACAAGTTATTTGATCTTAACATTGAGTCAGTCAAAGCACAAGACGACACAGTAGAATACTTTGATCCTTTCGCAGTGGACCCCTCTGAACACATTTCTGCTTCAGAAAAAGAAGTTTTGGAATTTGTAGGTGATCAAGTTGAGACTACAAGCTCTGAAGAACTAGCTGGTCTCGAGTTTAGTGAAGTTACTTTCTACTGTACCACACTTGCTGGTGCTGTTCAAGAAAATCCTGCCAAAACCTTCATTTCACTCACTAGACACACTTCAAAGCTCACAATTGGTGAACTAAATGCCAGGTCTGACTCCTAGATGCCAGGTCTGACTCCTAGAGCTGATCTTACTGACACGTATAAAATCATTGCTATAGCCTTTCTACTGTCAGCTTGCATTTACTTCCAAAACAGTCATTATCAACCAGTTGCAGGTGATAATTTGCACAGACTACCCTTTGGTGGTCAGTATCAAGACGGAACTAAGAAGATCTCTTACTTTCCGCAGCAACAATCCTACTTTCACTCAGGAAACAAGCTTAATGTCCTCATACTTATCTTCATTCTTACACTGGGTATTGTCCTCACCAATAAATTTAGTTTTAGCATTAGCCGTAATACTCACCAGCATCATTGCTACAACACACATTCTGCAACCCAAACAGGTCAATCAGTGCCAGGTCATCATTGAATGTCCTCATACTTATCTTCATTCTTACACTGGGTATTGTCCTCACCAATAAATTTAGTTTTAGCATTAGCCGTAATACTCACCAGCATCATTGCTACAACACACATTCTGCAACCCAAACAGGTCAATCAGTGCCAGGTCATCATTGATGGTGCAGCCATAGTCATAACAAATTGTCCAAACACACCCGAAGTTCTTAAAGCAATCAACTTCTCCCCTTGGAACGGGTTAAGTTTTCCTCAATTGTGAATGGAAAACCAACCTACAGCTTCTAACCCATCAGATGTACCACCAACTGCTGCTCAAGCTGGTGCCCAGAGCCCAGCCGACTTCTCAAATCCTAATACAGCTCCTTCCCTAAGTGATTTGAAGAAGATCAAATACGTGTCAACTGTCACTTCAGTTGCCACGCCTGCTGAAATTGAGGCCCTTGGCAAGATCTTTACTGCCATGGGTTTAGCAGCCAATGAGACCGGACCTGCCATGTGGGACCTCGCTCGTGCTTATGCTGATGTGCAAAGTTCAAAATCTGCACAACTTATAGGTGCCACACCATCCAACCCTGCTTTGTCTAGACGTGCACTTGCTGCACAGTTTGATCGTATCAATATCACACCCAGACAATTCTGCATGTATTTTGCAAAAATTGTTTGGAACATACTGTTAGACAGCAATGTGCCACCTGCCAACTGGGCAAAATTGGGCTATCAGGAAGATACAAAGTTTGCTGCTTTTGACTTCTTTGATGGAGTCACAAATCCAGCTAGTCTACAGCCTGCAGATGGCCTAATCAGGCAGCCCAATGAAAAAGAGCTTGCTGCTCACTCGGTTGCTAAATATGGTGCCCTTGCCCGCCAGAAAATATCCACTGGTAACTACATCACCACCCTTGGTGAAGTTACACGTGGTCACATGGGCGGCGCCAACACTATGTACGCAATTGATGCACCTCCTGAACTTTAA

>Sp13_P1_consensus_sequence

ATGGAAAGATCAACTCTGATTAATTTACTTCAATTGCACCACTTCGAGCCAAAACTCAGTGTTGAAGGAATCATAGTTGTGCACGGAATTGCAGGCACTGGGAAAACCACTTTACTTAGGACTTTATTTTCTGCTTACCCTAGCTTAGTTATAGGTTCACCTAGGCCTTGCTATTTAGATAAACAAAACAAAATTTCACAAGTTTGCTTATCTTGCTTTCCCAATACCCATTGTGATATTGTCGATGAGTATCATTTGCTAGAAAGTTTTCTAGAACCAAAATTGGCTATCTTTGGTGACCCCTGTCAATGCACATACATTGAGAGACTTAGAGTCCCACATTACACTTCCTTCAGAACTCATAGATTTGGAAAGTCAACTGCTGAGATTTTGAACAAACTGTTTGACCTTAATATAGTCTCAGTTAAGAAAGAAGACGACATCGTTGAATTCTTTAACCCTTTTGAAGTTGACCCCACTGAGCATATCTCTGCCTCTGAAGAAGAAGTCTTGGACTTTGTTTCTGACCAAGTGGTGACCACTAGCTCAGAGGAACTAGCAGGACTTGAGTTTGCAGAAACAACTTTCTACTGCACAACATTGGCCGCAGCTGTTGCTGAAAATCCTGCTAAGACTTTCATCTCTCTGACTAGACACACCCACAAACTCACCATTGGGGAACTAAATGCCAGGTCTAACTCCTAGATGCCAGGTCTAACTCCTAGAGCTGACCTCACTGACACATACAAAATCATTGCCATTGCTTTCTTGTTGTCAGCTTGCATTTACTTCCAAAATAGCCACTACCAACCTGTTGCTGGAGACAACTTGCACCGTTTGCCTTTTGGTGGCCAATATCAAGACGGCACCAAAAAGATATCTTATTTTCCACAACAGCAGTCATACTTTCATTCTGGAAACAAATTAAATGTCCTCATACTTATCTTCATTCTCACGTTGGGTATTGTCCTCACCAATAAATTTAGTTTTAGCTTTAGTCGTACTACTCACCAGCATTCTTGCTATAACACACATTCAGCAACCAACAATACACAACCATTGTCAGGTCATCATTGAATGTCCTCATACTTATCTTCATTCTCACGTTGGGTATTGTCCTCACCAATAAATTTAGTTTTAGCTTTAGTCGTACTACTCACCAGCATTCTTGCTATAACACACATTCAGCAACCAACAATACACAACCATTGTCAGGTCATCATTGACGGTGCTGCAATAGTCATAACAAATTGTGAGAACACACCAGAAGTGCTTAAAGCAATAAACTTCTCCCCTTGGAACGGGTTAAGTTTTCCTAAATTTGAAAATTAAATGCCTGACACAACACCTGTTGCTGCCACTTCAAGTGCACCACCCACAGCCAAAGATGCTGGTGCCAAAGCTCCTTCTGACTTCTCAAATCCCAATACAGCTCCTAGTCTCAGTGATTTGAAGAAAGTCAAGTATGTCTCCACCGTGACCTCCGTGGCCACACCAGCTGAAATTGAAGCCCTAGGCAAAATCTTCACCGCTATGGGCCTTGCCGCCAATGAGACTGGTCCGGCCATGTGGGATCTAGCTCGTGCATATGCTGATGTGCAGAGTTCTAAATCGGCACAGCTGATTGGAGCTACCCCTTCCAACCCTGCACTATCACGCCGAGCCCTTGCTGCTCAGTTTGATCGAATCAATATAACCCCCAGGCAATTTTGCATGTACTTTGCCAAAGTTGTTTGGAACATACTTCTCGACAGCAACATTCCACCAGCAAATTGGGCCAAACTTGGTTACCAAGAAGATACAAAATTTGCTGCATTTGACTTCTTCGATGGAGTCACCAACCCTGCCAGCCTGCAGCCTGCTGATGGTCTTATCAGGCAGCCAAATGAGAAAGAACTAGCTGCTCACTCCGTAGCTAAGTACGGCGCCTTGGCTAGGCAAAAGATCTCCACAGGTAATTATATTACCACACTTGGAGAAGTCACACGTGGACACATGGGAGGAGCTAACACCATGTACGCGATAGACGCACCCCCTGAACTTTAA

>Sp13_P8_c14

ATGGAAAGATCAACTCTGATTAATTTACTTCAATTGCACCACTTCGAGCCAAAACTCAGTGTTGAAGGAATCATAGTTGTGCACGGAATTGCAGGCACTGGGAAAACCACTTTACTTAGGACTTTATTTTCTGCTTACCCTAGCTTAGTTATAGGTTCACCTAGGCCTTGCTATTTAGATAAACAAAACAAAATTTCACAAGTTTGCTTATCTTGCTTTCCCAATACCCATTGTGATATTGTCGATGAGTATCATTTGCTAGAAAGTTTTCTAGAACCAAAATTGGCTATCTTTGGTGACCCCTGTCAATGCACATACATTGAGAGACTTAGAGTCCCACATTACACTTCCTTCAGAACTCATAGATTTGGAAAGTCAACTGCTGAGATTTTGAACAAACTGTTTGACCTTAATATAGTCTCAGTTAAGAAAGAAGACGACATCGTTGAATTCTTTAACCCTTTTGAAGTTGACCCCACTGAGCATATCTCTGCCTCTGAAGAAGAAGTCTTGGACTTTGTTTCTGACCAAGTGGTGACCACTAGCTCAGAGGAACTAGCAGGACTTGAGTTTGCAGAAACAACTTTCTACTGCACAACATTGGCCGCAGCTGTTGCTGAAAATCCTGCTAAGACTTTCATCTCTCTGACTAGACACACCCACAAACTCACCATTGGGGAACTAAATGCCAGGTCTAACTCCTAGATGCCAGGTCTAACTCCTAGAGCTGACCTCACTGACACATACAAAATCATTGCCATTGCTTTCTTGTTGTCAGCTTGCATTTACTTCCAAAATAGCCACTACCAACCTGTTGCTGGAGACAACTTGCACCGTTTGCCTTTTGGTGGCCAATATCAAGACGGCACCAAAAAGATATCTTATTTTCCACAACAGCAGTCATACTTTCATTCTGGAAACAAATTAAATGTCCTCATACTTATCTTCATTCTCACGTTGGGTATTGTCCTCACCAATAAATTTAGTTTTAGCTTTAGTCGTACTACTCACCAGCATTCTTGCTATAACACACATTCAGCAACCAACAATACACAACCATTGTCAGGTCATCATTGAATGTCCTCATACTTATCTTCATTCTCACGTTGGGTATTGTCCTCACCAATAAATTTAGTTTTAGCTTTAGTCGTACTACTCACCAGCATTCTTGCTATAACACACATTCAGCAACCAACAATACACAACCATTGTCAGGTCATCATTGACGGTGCTGCAATAGTCATAACAAATTGTGAGAACACACCAGAAGTGCTTAAAGCAATAAACTTCTCCCCTTGGAACGGGTTAAGTTTTCCTAAATTTGAAAATTAAATGCCTGACACAACACCTGTTGCTGCCACTTCAAGTGCACCACCCACAGCCAAAGATGCTGGTGCCAAAGCTCCTTCTGACTTCTCAAATCCCAATACAGCTCCTAGTCTCAGTGATTTGAAGAAAGTCAAGTATGTCTCCACCGTGACCTCCGTGGCCACACCAGCTGAAATTGAAGCCCTAGGCAAAATMTTCACCGCTATGGGCCTTGCCGCCAATGAGACTGGTCCGGCCATGTGGGATCTAGCTCGTGCATATGCTGATGTGCAGAGTTCTAAATCGGCACAGCTGATTGGAGCTACCCCTTCCAACCCTGCACTATCACGCCGAGCCCTTGCTGCTCAGTTTGATCGAATCAATATAACCCCCAGGCAATTTTGCATGTACTTTGCCAAAGTTGTTTGGAACATACTTCTCGACAGCAACATTCCACCAGCAAATTGGGCCAAACTTGGTTACCAAGAAGATACAAAATTTGCTGCATTTGACTTCTTCGATGGAGTCACCAACCCTGCCAGCCTGCAGCCTGCTGATGGTCTTATCAGGCAGCCAAATGAGAAAGAACTAGCTGCTCACTCCGTAGCTAAGTACGGCGCCTTGGCTAGGCAAAAGATCTCCACAGGTAATTATATTACCACACTTGGAGAAGTCACACGTGGACACATGGGAGGAGCTAACACCATGTACGCGATAGACGCACCCCCTGAACTTTAA

>Sp13_P8_c13

ATGGAAAGATCAACTCTGATTAATTTACTTCAATTGCACCACTTCGAGCCAAAACTCAGTGTTGAAGGAATCATAGTTGTGCACGGAATTGCAGGCACTGGGAAAACCACTTTACTTAGGACTTTATTTTCTGCTTACCCTAGCTTAGTTATAGGTTCACCTAGGCCTTGCTATTTAGATAAACAAAACAAAATTTCACAAGTTTGCTTATCTTGCTTTCCCAATACCCATTGTGATATTGTCGATGAGTATCATTTGCTAGAAAGTTTTCTAGAACCAAAATTGGCTATCTTTGGTGACCCCTGTCAATGCACATACATTGAGAGACTTAGAGTCCCACATTACACTTCCTTCAGAACTCATAGATTTGGAAAGTCAACTGCTGAGATTTTGAACAAACTGTTTGACCTTAATATAGTCTCAGTTAAGAAAGAAGACGACATCGTTGAATTCTTTAACCCTTTTGAAGTTGACCCCACTGAGCATATCTCTGCCTCTGAAGAAGAAGTCTTGGACTTTGTTTCTGACCAAGTGGTGACCACTAGCTCAGAGGAACTAGCAGGACTTGAGTTTGCAGAAACAACTTTCTACTGCACAACATTGGCCGCAGCTGTTGCTGAAAATCCTGCTAAGACTTTCATCTCTCTGACTAGACACACCCACAAACTCACCATTGGGGAACTAAATGCCAGGTCTAACTCCTAGATGCCAGGTCTAACTCCTAGAGCTGACCTCACTGACACATACAAAATCATTGCCATTGCTTTCTTGTTGTCAGCTTGCATTTACTTCCAAAATAGCCACTACCAACCTGTTGCTGGAGACAACTTGCACCGTTTGCCTTTTGGTGGCCAATATCAAGACGGCACCAAAAAGATATCTTATTTTCCACAACAGCAGTCATACTTTCATTCTGGAAACAAATTAAATGTCCTCATACTTATCTTCATTCTCACGTTGGGTATTGTCCTCACCAATAAATTTAGTTTTAGCTTTAGTCGTACTACTCACCAGCATTCTTGCTATAACACACATTCAGCAACCAACAATACACAACCATTGTCAGGTCATCATTGAATGTCCTCATACTTATCTTCATTCTCACGTTGGGTATTGTCCTCACCAATAAATTTAGTTTTAGCTTTAGTCGTACTACTCACCAGCATTCTTGCTATAACACACATTCAGCAACCAACAATACACAACCATTGTCAGGTCATCATTGACGGTGCTGCAATAGTCATAACAAATTGTGAGAACACACCAGAAGTGCTTAAAGCAATAAACTTCTCCCCTTGGAACGGGTTAAGTTTTCCTAAATTTGAAAATTAAATGCCTGACACAACACCTGTTGCTGCCACTTCAAGTGCACCACCCGCAGCCAAAGATGCTGGTGCCAAAGCTCCTTCTGACTTCTCAAATCCCAATACAGCTCCTAGTCTCAGTGATTTGAAGAAAGTCAAGTATGTCTCCACCGTGACCTCCGTGGCCACACCAGCTGAAATTGAAGCCCTAGGCAAAATCTTCACCGCTATGGGCCTTGCCGCCAATGAGACTGGTCCGGCCATGTGGGATCTAGCTCGTGCATATGCTGATGTGCAGAGTTCTAAATCGGCACAGCTGATTGGAGCTACCCCTTCCAACCCTGCACTATCACGCCGAGCCCTTGCTGCTCAGTTTGATCGAATCAATATAACCCCCAGGCAATTTTGCATGTACTTTGCCAAAGTTGTTTGGAACATACTTCTCGACAGCAACATTCCACCAGCAAATTGGGCCAAACTTGGTTACCAAGAAGATACAAAATTTGCTGCATTTGACTTCTTCGATGGAGTCACCAACCCTGCCAGCCTGCAGCCTGCTGATGGTCTTATCAGGCAGCCAAATGAGAAAGAACTAGCTGCTCACTCCGTAGCTAAGTACGGCGCCTTGGCTAGGCAAAAGATCTCCACAGGTAATTATATTACCACACTTGGAGAAGTCACACGTGGACACATGGGAGGAGCTAACACCATGTACGCGATAGACGCACCCCCTGAACTTTAA

>Sp13_P8_c12

ATGGAAAGATCAACTCTGATTAATTTACTTCAATTGCACCACTTCGAGCCAAAACTCAGTGTTGAAGGAATCATAGTTGTGCACGGAATTGCAGGCACTGGGAAAACCACTTTACTTAGGACTTTATTTTCTGCTTACCCTAGCTTAGTTATAGGTTCACCTAGGCCTTGCTATTTAGATAAACAAAACAAAATTTCACAAGTTTGCTTATCTTGCTTTCCCAATACCCATTGTGATATTGTCGATGAGTATCATTTGCTAGAAAGTTTTCTAGAACCAAAATTGGCTATCTTTGGTGACCCCTGTCAATGCACATACATTGAGAGACTTAGAGTCCCACATTACACTTCCTTCAGAACTCATAGATTTGGAAAGTCAACTGCTGAGATTTTGAACAAACTGTTTGACCTTAATATAGTCTCAGTTAAGAAAGAAGACGACATCGTTGAATTCTTTAACCCTTTTGAAGTTGACCCCACTGAGCATATCTCTGCCTCTGAAGAAGAAGTCTTGGACTTTGTTTCTGACCAAGTGGTGACCACTAGCTCAGAGGAACTAGCAGGACTTGAGTTTGCAGAAACAACTTTCTACTGCACAACATTGGCCGCAGCTGTTGCTGAAAATCCTGCTAAGACTTTCATCTCTCTGACTAGACACACCCACAAACTCACCATTGGGGAACTAAATGCCAGGTCTAACTCCTAGATGCCAGGTCTAACTCCTAGAGCTGACCTCACTGACACATACAAAATCATTGCCATTGCTTTCTTGTTGTCAGCTTGCATTTACTTCCAAAATAGCCACTACCAACCTGTTGCTGGAGACAACTTGCACCGCTTGCCTTTTGGTGGCCAATATCAAGACGGCACCAAAAAGATATCTTATTTTCCACAACAGCAGTCATACTTTCATTCTGGAAACAAATTAAATGTCCTCATACTTATCTTCATTCTCACGTTGGGTATTGTCCTCACCAATAAATTTAGTTTTAGCTTTAGTCGTACTACTCACCAGCATTCTTGCTATAACACACATTCAGCAACCAACAATACACAACCATTGTCAGGTCATCATTGAATGTCCTCATACTTATCTTCATTCTCACGTTGGGTATTGTCCTCACCAATAAATTTAGTTTTAGCTTTAGTCGTACTACTCACCAGCATTCTTGCTATAACACACATTCAGCAACCAACAATACACAACCATTGTCAGGTCATCATTGACGGTGCTGCAATAGTCATAACAAATTGTGAGAACACACCAGAAGTGCTTAAAGCAATAAACTTCTCCCCTTGGAACGGGTTAAGTTTTCCTAAATTTGAAAATTAAATGCCTGACACAACACCTGTTGCTGCCACTTCAAGTGCACCACCCACAGCCAAAGATGCTGGTGCCAAAGCTCCTTCTGACTTCTCAAATCCCAATACAGCTCCTAGTCTCAGTGATTTGAAGAAAGTCAAGTATGTCTCCACCGTGACCTCCGTGGCCACACCAGCTGAAATTGAAGCCCTAGGCAAAATCTTCACCGCTATGGGCCTTGCCGCCAATGAGACTGGTCCGGCCATGTGGGATCTAGCTCGTGCATATGCTGATGTGCAGAGTTCTAAATCGGCACAGCTGATTGGAGCTACCCCTTCCAACCCTGCACTATCACGCCGAGCCCTTGCTGCTCAGTTTGATCGAATCAATATAACCCCCAGGCAATTTTGCATGTACTTTGCCAAAGTTGTTTGGAACATACTTCTCGACAGCAACATTCCACCAGCAAATTGGGCCAAACTTGGTTACCAAGAAGATACAAAATTTGCTGCATTTGACTTCTTCGATGGAGTCACCAACCCTGCCAGCCTGCAGCCTGCTGATGGTCTTATCAGGCAGCCAAATGAGAAAGAACTAGCTGCTCACTCCGTAGCTAAGTACGGCGCCTTGGCTAGGCAAAAGATCTCCACAGGTAATTATATTACCACACTTGGAGAAGTCACACGTGGACACATGGGAGGAGCTAACACCATGTACGCGATAGACGCACCCCCTGAACTTTAA

>Sp13_P8_c11

ATGGAAAGATCAACTCTGATTAATTTACTTCAATTGCACCACTTCGAGCCAAAACTCAGTGTTGAAGGAATCATAGTTGTGCACGGAATTGCAGGCACTGGGAAAACCACTTTACTTAGGACTTTATTTTCTGCTTACCCTAGCTTAGTTATAGGTTCACCTAGGCCTTGCTATTTAGATAAACAAAACAAAATTTCACAAGTTTGCTTATCTTGCTTTCCCAATACCCATTGTGATATTGTCGATGAGTATCATTTGCTAGAAAGTTTTCTAGAACCAAAATTGGCTATCTTTGGTGACCCCTGTCAATGCACATACATTGAGAGACTTAGAGTCCCACATTACACTTCCTTCAGAACTCATAGATTTGGAAAGTCAACTGCTGAGATTTTGAACAAACTGTTTGACCTTAATATAGTCTCAGTTAAGAAAGAAGACGACATCGTTGAATTCTTTAACCCTTTTGAAGTTGACCCCACTGAGCATATCTCTGCCTCTGAAGAAGAAGTCTTGGACTTTGTTTCTGACCAAGTGGTGACCACTAGCTCAGAGGAACTAGCAGGACTTGAGTTTGCAGAAACAACTTTCTACTGCACAACATTGGCCGCAGCTGTTGCTGAAAATCCTGCTAAGACTTTCATCTCTCTGACTAGACACACCCACAAACTCACCATTGGGGAACTAAATGCCAGGTCTAACTCCTAGATGCCAGGTCTAACTCCTAGAGCTGACCTCACTGACACATACAAAATCATTGCCATTGCTTTCTTGTTGTCAGCTTGCATTTACTTCCAAAATAGCCACTACCAACCTGTTGCTGGAGACAACTTGCACCGTTTGCCTTTTGGTGGCCAATATCAAGACGGCACCAAAAAGATATCTTATTTTCCACAACAGCAGTCATACTTTCATTCTGGAAACAAATTAAATGTCCTCATACTTATCTTCATTCTCACGTTGGGTATTGTCCTCACCAATAAATTTAGTTTTAGCTTTAGTCGTACTACTCACCAGCATTCTTGCTATAACACACATTCAGCAACCAACAATACACAACCATTGTCAGGTCATCATTGAATGTCCTCATACTTATCTTCATTCTCACGTTGGGTATTGTCCTCACCAATAAATTTAGTTTTAGCTTTAGTCGTACTACTCACCAGCATTCTTGCTATAACACACATTCAGCAACCAACAATACACAACCATTGTCAGGTCATCATTGACGGTGCTGCAATAGTCATAACAAATTGTGAGAACACACCAGAAGTGCTTAAAGCAATAAACTTCTCCCCTTGGAACGGGTTAAGTTTTCCTAAATTTGAAAATTAAATGCCTGACACAACACCTGTTGCTGCCACTTCAAGTGCACCACCCACAGCCAAAGATGCTGGTGCCAAAGCTCCTTCTGACTTCTCAAATCCCAATACAGCTCCTAGTCTCAGTGATTTGAAGAAGGTCAAGTATGTCTCCACCGTGACCTCCGTGGCCACACCAGCTGAAATTGAAGCCCTAGGCAAAATCTTCACCGCTATGGGCCTTGCCGCCAATGAGACTGGTCCGGCCATGTGGGATCTAGCTCGTGCATATGCTGATGTGCAGAGTTCTAAATCGGCACAGCTGATTGGAGCTACCCCTTCCAACCCTGCACTATCACGCCGAGCCCTTGCTGCTCAGTTTGATCGAATCAATATAACCCCCAGGCAATTTTGCATGTACTTTGCCAAAGTTGTTTGGAACATACTTCTCGACAGCAACATTCCACCAGCAAATTGGGCCAAACTTGGTTACCAAGAAGATACAAAATTTGCTGCATTTGACTTCTTCGATGGAGTCACCAACCCTGCCAGCCTGCAGCCTGCTGATGGTCTTATCAGGCAGCCAAATGAGAAAGAACTAGCTGCTCACTCCGTAGCTAAGTACGGCGCCTTGGCTAGGCAAAAGATCTCCACAGGTAATTATATTACCACACTTGGAGAAGTCACACGTGGACACATGGGAGGAGCTAACACCATGTACGCGATAGACGCACCCCCTGAACTTTAA

>Sp13_P8_c10

ATGGAAAGATCAACTCTGATTAATTTACTTCAATTGCACCACTTCGAGCCAAAACTCAGTGTTGAAGGAATCATAGTTGTGCACGGAATTGCAGGCACTGGGAAAACCACTTTACTTAGGACTTTATTTTCTGCTTACCCTAGCTTAGTTATAGGTTCACCTAGGCCTTGCTATTTAGATAAACAAAACAAAATTTCACAAGTTTGCTTATCTTGCTTTCCCAATACCCATTGTGATATTGTCGATGAGTATCATTTGCTAGAAAGTTTTCTAGAACCAAAATTGGCTATCTTTGGTGACCCCTGTCAATGCACATACATTGAGAGACTTAGAGTCCCACATTACACTTCCTTCAGAACTCATAGATTTGGAAAGTCAACTGCTGAGATTTTGAACAAACTGTTTGACCTTAATATAGTCTCAGTTAAGAAAGAAGACGACATCGTTGAATTCTTTAACCCTTTTGAAGTTGACCCCACTGAGCATATCTCTGCCTCTGAAGAAGAAGTCTTGGACTTTGTTTCTGACCAAGTGGTGACCACTAGCTCAGAGGAACTAGCAGGACTTGAGTTTGCAGAAACAACTTTCTACTGCACAACATTGGCCGCAGCTGTTGCTGAAAATCCTGCTAAGACTTTCATCTCTCTGACTAGACACACCCACAAACTCACCATTGGGGAACTAAATGCCAGGTCTAACTCCTAGATGCCAGGTCTAACTCCTAGAGCTGACCTCACTGACACATACAAAATCATTGCCATTGCTTTCTTGTTGTCAGCTTGCATTTACTTCCAAAATAGCCACTACCAACCTGTTGCTGGAGACAACTTGCACCGTTTGCCTTTTGGTGGCCAATATCAAGACGGCACCAAAAAGATATCTTATTTTCCACAACAGCAGTCATACTTTCATTCTGGAAACAAATTAAATGTCCTCATACTTATCTTCATTCTCACGTTGGGTATTGTCCTCACCAATAAATTTAGTTTTAGCTTTAGTCGTACTACTCACCAGCATTCTTGCTATAACACACATTCAGCAACCAACAATACACAACCATTGTCAGGTCATCATTGAATGTCCTCATACTTATCTTCATTCTCACGTTGGGTATTGTCCTCACCAATAAATTTAGTTTTAGCTTTAGTCGTACTACTCACCAGCATTCTTGCTATAACACACATTCAGCAACCAACAATACACAACCATTGTCAGGTCATCATTGACGGTGCTGCAATAGTCATAACAAATTGTGAGAACACACCAGAAGTGCTTAAAGCAATAAACTTCTCCCCTTGGAACGGGTTAAGTTTTCCTAAATTTGAAAATTAAATGCCTGACACAACACCTGTTGCTGCCACTTCAAGTGCACCACCCACAGCCAAAGATGCTGGTGCCAAAGCTCCTTCTGACTTCTCAAATCCCAATACAGCTCCTAGTCTCAGTGATTTGAAGAAAGTCAAGTATGTCTCCACCGTGACCTCCGTGGCCACACCAGCTGAAATTGAAGCCCTAGGCAAAATCTTCACCGCTATGGGCCTTGCCGCCAATGAGACTGGTCCGGCCATGTGGGATCTAGCTCGTGCATATGCTGATGTGCAGAGTTCTAAATCGGCACAGCTGATTGGAGCTACCCCTTCCAACCCTGCACTATCACGCCGAGCCCTTGCTGCTCAGTTTGATCGAATCAATATAACCCCCAGGCAATTTTGCATGTACTTTGCCAAAGTTGTTTGGAACATACTTCTCGACAGCAACATTCCACCAGCAAATTGGGCCAAACTTGGTTACCAAGAAGATACAAAATTTGCTGCATTTGACTTCTTCGATGGAGTCACCAACCCTGCCAGCCTGCAGCCTGCTGATGGTCTTATCAGGCAGCCAAATGAGAAAGAACTAGCTGCTCACTCCGTAGCTAAGTACGGCGCCTTGGCTAGGCAAAAGATCTCCACAGGTAATTATATTACCACACTTGGAGAAGTCACACGTGGACACATGGGAGGAGCTAACACCATGTACGCGATAGACGCACCCCCTGAACTTTAA

>Sp13_P8_c9

ATGGAAAGATCAACTCTGATTAATTTACTTCAATTGCACCACTTCGAGCCAAAACTCAGTGTTGAAGGAATCATAGTTGTGCACGGAATTGCAGGCACTGGGAAAACCACTTTACTTAGGACTTTATTTTCTGCTTACCCTAGCTTAGTTATAGGTTCACCTAGGCCTTGCTATTTAGATAAACAAAACAAAATTTCACAAGTTTGCTTATCTTGCTTTCCCAATACCCATTGTGATATTGTCGATGAGTATCATTTGCTAGAAAGTTTTCTAGAACCAAAATTGGCTATCTTTGGTGACCCCTGTCAATGCACATACATTGAGAGACTTAGAGTCCCACATTACACTTCCTTCAGAACTCATAGATTTGGAAAGTCAACTGCTGAGATTTTGAACAAACTGTTTGACCTTAATATAGTCTCAGTTAAGAAAGAAGACGACATCGTTGAATTCTTTAACCCTTTTGAAGTTGACCCCACTGAGCATATCTCTGCCTCTGAAGAAGAAGTCTTGGACTTTGTTTCTGACCAAGTGGTGACCACTAGCTCAGAGGAACTAGCAGGACTTGAGTTTGCAGAAACAACTTTCTACTGCACAACATTGGCCGCAGCTGTTGCTGAAAATCCTGCTAAGACTTTCATCTCTCTGACTAGACACACCCACAAACTCACCATTGGGGAACTAAATGCCAGGTCTAACTCCTAGATGCCAGGTCTAACTCCTAGAGCTGACCTCACTGACACATACAAAATCATTGCCATTGCTTTCTTGTTGTCAGCTTGCATTTACTTCCAAAATAGCCACTACCAACCTGTTGCTGGAGACAACTTGCACCGTTTGCCTTTTGGTGGCCAATATCAAGACGGCACCAAAAAGATATCTTATTTTCCACAACAGCAGTCATACTTTCATTCTGGAAACAAATTAAATGTCCTCATACTTATCTTCATTCTCACGTTGGGTATTGTCCTCACCAATAAATTTAGTTTTAGCTTTAGTCGTACTACTCACCAGCATTCTTGCTATAACACACATTCAGCAACCAACAATACACAACCATTGTCAGGTCATCATTGAATGTCCTCATACTTATCTTCATTCTCACGTTGGGTATTGTCCTCACCAATAAATTTAGTTTTAGCTTTAGTCGTACTACTCACCAGCATTCTTGCTATAACACACATTCAGCAACCAACAATACACAACCATTGTCAGGTCATCATTGACGGTGCTGCAATAGTCATAACAAATTGTGAGAACACACCAGAAGTGCTTAAAGCAATAAACTTCTCCCCTTGGAACGGGTTAAGTTTTCCTAAATTTGAAAATTAAATGCCTGACACAACACCTGTTGCTGCCACTTCAAGTGCACCACCCACAGCCAAAGATGCTGGTGCCAAAGCTCCTTCTGACTTCTCAAATCCCAATACAGCTCCTAGTCTCAGTGATTTGAAGAAAGTCAAGTATGTCTCCACCGTGACCTCCGTGGCCACACCAGCTGAAATTGAAGCCCTAGGCAAAATCTTCACCGCTATGGGCCTTGCCGCCAATGAGACTGGTCCGGCCATGTGGGATCTAGCTCGTGCATATGCTGATGTGCAGAGTTCTAAATCGGCACAGCTGATTGGAGCTACCCCTTCCAACCCTGCACTATCACGCCGAGCCCTTGCTGCTCAGTTTGATCGAATCAATATAACCCCCAGGCAATTTTGCATGTACTTTGCCAAAGTTGTTTGGAACATACTTCTCGACAGCAACATTCCACCAGCAAATTGGGCCAAACTTGGTTACCAAGAAGATACAAAATTTGCTGCATTTGACTTCTTCGATGGAGTCACCAACCCTGCCAGCCTGCAGCCTGCTGATGGTCTTATCAGGCAGCCAAATGAGAAAGAACTAGCTGCTCACTCCGTAGCTAAGTACGGCGCCTTGGCTAGGCAAAAGATCTCCACAGGTAATTATATTACCACACTTGGAGAAGTCACACGTGGACACATGGGAGGAGCTAACACCATGTACGCGATAGACGCACCCCCTGAACTTTAA

>Sp13_P8_c8

ATGGAAAGATCAACTCTGATTAATTTACTTCAATTGCACCACTTCGAGCCAAAACTCAGTGTTGAAGGAATCATAGTTGTGCACGGAATTGCAGGCACTGGGAAAACCACTTTACTTAGGACTTTATTTTCTGCTTACCCTAGCTTAGTTATAGGTTCACCTAGGCCTTGCTATTTAGATAAACAAAACAAAATTTCACAAGTTTGCTTATCTTGCTTTCCCAATACCCATTGTGATATTGTCGATGAGTATCATTTGCTAGAAAGTTTTCTAGAACCAAAATTGGCTATCTTTGGTGACCCCTGTCAATGCACATACATTGAGAGACTTAGAGTCCCACATTACACTTCCTTCAGAACTCATAGATTTGGAAAGTCAACTGCTGAGATTTTGAACAAACTGTTTGACCTTAATATAGTCTCAGTTAAGAAAGAAGACGACATCGTTGAATTCTTTAACCCTTTTGAAGTTGACCCCACTGAGCATATCTCTGCCTCTGAAGAAGAAGTCTTGGACTTTGTTTCTGACCAAGTGGTGACCACTAGCTCAGAGGAACTAGCAGGACTTGAGTTTGCAGAAACAACTTTCTACTGCACAACATTGGCCGCAGCTGTTGCTGAAAATCCTGCTAAGACTTTCATCTCTCTGACTAGACACACCCACAAACTCACCATTGGGGAACTAAATGCCAGGTCTAACTCCTAGATGCCAGGTCTAACTCCTAGAGCTGACCTCACTGACACATACAAAATCATTGCCATTGCTTTCTTGTTGTCAGCTTGCATTTACTTCCAAAATAGCCACTACCAACCTGTTGCTGGAGACAACTTGCACCGTTTGCCTTTTGGTGGCCAATATCAAGACGGCACCAAAAAGATATCTTATTTTCCACAACAGCAGTCATACTTTCATTCTGGAAACAAATTAAATGTCCTCATACTTATCTTCATTCTCACGTTGGGTATTGTCCTCACCAATAAATTTAGTTTTAGCTTTAGTCGTACTACTCACCAGCATTCTTGCTATAACACACATTCAGCAACCAACAATACACAACCATTGTCAGGTCATCATTGAATGTCCTCATACTTATCTTCATTCTCACGTTGGGTATTGTCCTCACCAATAAATTTAGTTTTAGCTTTAGTCGTACTACTCACCAGCATTCTTGCTATAACACACATTCAGCAACCAACAATACACAACCATTGTCAGGTCATCATTGACGGTGCTGCAATAGTCATAACAAATTGTGAGAACACACCAGAAGTGCTTAAAGCAATAAACTTCTCCCCTTGGAACGGGTTAAGTTTTCCTAAATTTGAAAATTAAATGCCTGACACAACACCTGTTGCTGCCACTTCAAGTGCACCACCCACAGCCAAAGATGCTGGTGCCAAAGCTCCTTCTGACTTCTCAAATCCCAATACAGCTCCTAGTCTCAGTGATTTGAAGAAAGTCAAGTATGTCTCCACCGTGACCTCCGTGGCCACACCAGCTGAAATTGAAGCCCTAGGCAAAATCTTCACCGCTATGGGCCTTGCCGCCAATGAGACTGGTCCGGCCATGTGGGATCTAGCTCGTGCATATGCTGATGTGCAGAGTTCTAAATCGGCACAGCTGATTGGAGCTACCCCTTCCAACCCTGCACTATCACGCCGAGCCCTTGCTGCTCAGTTTGATCGAATCAATATAACCCCCAGGCAATTTTGCATGTACTTTGCCAAAGTTGTTTGGAACATACTTCTCGACAGCAACATTCCACCAGCAAATTGGGCCAAACTTGGTTACCAAGAAGATACAAAATTTGCTGCATTTGACTTCTTCGATGGAGTCACCAACCCTGCCAGCCTGCAGCCTGCTGATGGTCTTATCAGGCAGCCAAATGAGAAAGAACTAGCTGCTCACTCCGTAGCTAAGTACGGCGCCTTGGCTAGGCAAAAGATCTCCACAGGTAATTATATTACCACACTTGGAGAAGTCACACGTGGACACATGGGAGGAGCTAACACCATGTACGCGATAGACGCACCCCCTGAACTTTAA

>Sp13_P8_c7

ATGGAAAGATCAACTCTGATTAATTTACTTCAATTGCACCACTTCGAGCCAAAACTCAGTGTTGAAGGAATCATAGTTGTGCACGGAATTGCAGGCACTGGGAAAACCACTTTACTTAGGACTTTATTTTCTGCTTACCCTAGCTTAGTTATAGGTTCACCTAGGCCTTGCTATTTAGATAAACAAAACAAAATTTCACAAGTTTGCTTATCTTGCTTTCCCAATACCCATTGTGATATTGTCGATGAGTATCATTTGCTAGAAAGTTTTCTAGAACCAAAATTGGCTATCTTTGGTGACCCCTGTCAATGCACATACATTGAGAGACTTAGAGTCCCACATTACACTTCCTTCAGAACTCATAGATTTGGAAAGTCAACTGCTGAGATTTTGAACAAACTGTTTGACCTTAATATAGTCTCAGTTAAGAAAGAAGACGACATCGTTGAATTCTTTAACCCTTTTGAAGTTGACCCCACTGAGCATATCTCTGCCTCTGAAGAAGAAGTCTTGGACTTTGTTTCTGACCAAGTGGTGACCACTAGCTCAGAGGAACTAGCAGGACTTGAGTTTGCAGAAACAACTTTCTACTGCACAACATTGGCCGCAGCTGTTGCTGAAAATCCTGCTAAGACTTTCATCTCTCTGACTAGACACACCCACAAACTCACCATTGGGGAACTAAATGCCAGGTCTAACTCCTAGATGCCAGGTCTAACTCCTAGAGCTGACCTCACTGACACATACAAAATCATTGCCATTGCTTTCTTGTTGTCAGCTTGCATTTACTTCCAAAATAGCCACTACCAACCTGTTGCTGGAGACAACTTGCACCGTTTGCCTTTTGGTGGCCAATATCAAGACGGCACCAAAAAGATATCTTATTTTCCACAACAGCAGTCATACTTTCATTCTGGAAACAAATTAAATGTCCTCATACTTATCTTCATTCTCACGTTGGGTATTGTCCTCACCAATAAATTTAGTTTTAGCTTTAGTCGTACTACTCACCAGCATTCTTGCTATAACACACATTCAGCAACCAACAATACACAACCATTGTCAGGTCATCATTGAATGTCCTCATACTTATCTTCATTCTCACGTTGGGTATTGTCCTCACCAATAAATTTAGTTTTAGCTTTAGTCGTACTACTCACCAGCATTCTTGCTATAACACACATTCAGCAACCAACAATACACAACCATTGTCAGGTCATCATTGACGGTGCTGCAATAGTCATAACAAATTGTGAGAACACACCAGAAGTGCTTAAAGCAATAAACTTCTCCCCTTGGAACGGGTTAAGTTTTCCTAAATTTGAAAATTAAATGCCTGACACAACACCTGTTGCTGCCACTTCAAGTGCACCACCCACAGCCAAAGATGCTGGTGCCAAAGCTCCTTCTGACTTCTCAAATCCCAATACAGCTCCTAGTCTCAGTGATTTGAAGAAAGTCAAGTATGTCTCCACCGTGACCTCCGTGGCCACACCAGCTGAAATTGAAGCCCTAGGCAAAATCTTCACCGCTATGGGCCTTGCCGCCAATGAGACTGGTCCGGCCATGTGGGATCTAGCTCGTGCATATGCTGATGTGCAGAGTTCTAAATCGGCACAGCTGATTGGAGCTACCCCTTCCAACCCTGCACTATCACGCCGAGCCCTTGCTGCTCAGTTTGATCGAATCAATATAACCCCCAGGCAATTTTGCATGTACTTTGCCAAAGTTGTTTGGAACATACTTCTCGACAGCAACATTCCACCAGCAAATTGGGCCAAACTTGGTTACCAAGAAGATACAAAATTTGCTGCATTTGACTTCTTCGATGGAGTCACCAACCCTGCCAGCCTGCAGCCTGCTGATGGTCTTATCAGGCAGCCAAATGAGAAAGAACTAGCTGCTCACTCCGTAGCTAAGTACGGCGCCTTGGCTAGGCAAAAGATCTCCACAGGTAATTATATTACCACACTTGGAGAAGTCACACGTGGACACATGGGAGGAGCTAACACCATGTACGCGATAGACGCACCCCCTGAACTTTAA

>Sp13_P8_c6

ATGGAAAGATCAACTCTGATTAATTTACTTCAATTGCACCACTTCGAGCCAAAACTCAGTGTTGAAGGAATCATAGTTGTGCACGGAATTGCAGGCACTGGGAAAACCACTTTACTTAGGACTTTATTTTCTGCTTACCCTAGCTTAGTTATAGGTTCACCTAGGCCTTGCTATTTAGATAAACAAAACAAAATTTCACAAGTTTGCTTATCTTGCTTTCCCAATACCCATTGTGATATTGTCGATGAGTATCATTTGCTAGAAAGTTTTCTAGAACCAAAATTGGCTATCTTTGGTGACCCCTGTCAATGCACATACATTGAGAGACTTAGAGTCCCACATTACACTTCCTTCAGAACTCATAGATTTGGAAAGTCAACTGCTGAGATTTTGAACAAACTGTTTGACCTTAATATAGTCTCAGTTAAGAAAGAAGACGACATCGTTGAATTCTTTAACCCTTTTGAAGTTGACCCCACTGAGCATATCTCTGCCTCTGAAGAAGAAGTCTTGGACTTTGTTTCTGACCAAGTGGTGACCACTAGCTCAGAGGAACTAGCAGGACTTGAGTTTGCAGAAACAACTTTCTACTGCACAACATTGGCCGCAGCTGTTGCTGAAAATCCTGCTAAGACTTTCATCTCTCTGACTAGACACACCCACAAACTCACCATTGGGGAACTAAATGCCAGGTCTAACTCCTAGATGCCAGGTCTAACTCCTAGAGCTGACCTCACTGACACATACAAAATCATTGCCATTGCTTTCTTGTTGTCAGCTTGCATTTACTTCCAAAATAGCCACTACCAACCTGTTGCTGGAGACAACTTGCACCGTTTGCCTTTTGGTGGCCAATATCAAGACGGCACCAAAAAGATATCTTATTTTCCACAACAGCAGTCATACTTTCATTCTGGAAACAAATTAAATGTCCTCATACTTATCTTCATTCTCACGTTGGGTATTGTCCTCACCAATAAATTTAGTTTTAGCTTTAGTCGTACTACTCACCAGCATTCTTGCTATAACACACATTCAGCAACCAACAATACACAACCATTGTCAGGTCATCATTGAATGTCCTCATACTTATCTTCATTCTCACGTTGGGTATTGTCCTCACCAATAAATTTAGTTTTAGCTTTAGTCGTACTACTCACCAGCATTCTTGCTATAACACACATTCAGCAACCAACAATACACAACCATTGTCAGGTCATCATTGACGGTGCTGCAATAGTCATAACAAATTGTGAGAACACACCAGAAGTGCTTAAAGCAATAAACTTCTCCCCTTGGAACGGGTTAAGTTTTCCTAAATTTGAAAATTAAATGCCTGACACAACACCTGTTGCTGCCACTTCAAGTGCACCACCCACAGCCAAAGATGCTGGTGCCAAAGCTCCTTCTGACTTCTCAAATCCCAATACAGCTCCTAGTCTCAGTGATTTGAAGAAAGTCAAGTATGTCTCCACCGTGACCTCCGTGGCCACACCAGCTGAAATTGAAGCCCTAGGCAAAATCTTCACCGCTATGGGCCTTGCCGCCAATGAGACTGGTCCGGCCATGTGGGATCTAGCTCGTGCATATGCTGATGTGCAGAGTTCTAAATCGGCACAGCTGATTGGAGCTACCCCTTCCAACCCTGCACTATCACGCCGAGCCCTTGCTGCTCAGTTTGATCGAATCAATATAACCCCCAGGCAATTTTGCATGTACTTTGCCAAAGTTGTTTGGAACATACTTCTCGACAGCAACATTCCACCAGCAAATTGGGCCAAACTTGGTTACCAAGAAGATACAAAATTTGCTGCATTTGACTTCTTCGATGGAGTCACCAACCCTGCCAGCCTGCAGCCTGCTGATGGTCTTATCAGGCAGCCAAATGAGAAAGAACTAGCTGCTCACTCCGTAGCTAAGTACGGCGCCTTGGCTAGGCAAAAGATCTCCACAGGTAATTATATTACCACACTTGGAGAAGTCACACGTGGACACATGGGAGGAGCTAACACCATGTACGCGATAGACGCACCCCCTGAACTTTAA

>Sp13_P8_c5

ATGGAAAGATCAACTCTGATTAATTTACTTCAATTGCACCACTTCGAGCCAAAACTCAGTGTTGAAGGAATCATAGTTGTGCACGGAATTGCAGGCACTGGGAAAACCACTTTACTTAGGACTTTATTTTCTGCTTACCCTAGCTTAGTTATAGGTTCACCTAGGCCTTGCTATTTAGATAAACAAAACAAAATTTCACAAGTTTGCTTATCTTGCTTTCCCAATACCCATTGTGATATTGTCGATGAGTATCATTTGCTAGAAAGTTTTCTAGAACCAAAATTGGCTATCTTTGGTGACCCCTGTCAATGCACATACATTGAGAGACTTAGAGTCCCACATTACACTTCCTTCAGAACTCATAGATTTGGAAAGTCAACTGCTGAGATTTTGAACAAACTGTTTGACCTTAATATAGTCTCAGTTAAGAAAGAAGACGGCATCGTTGAATTCTTTAACCCTTTTGAAGTTGACCCCACTGAGCATATCTCTGCCTCTGAAGAAGAAGTCTTGGACTTTGTTTCTGACCAAGTGGTGACCACTAGCTCAGAGGAACTAGCAGGACTTGAGTTTGCAGAAACAACTTTCTACTGCACAACATTGGCCGCAGCTGTTGCTGAAAATCCTGCTAAGACTTTCATCTCTCTGACTAGACACACCCACAAACTCACCATTGGGGAACTAAATGCCAGGTCTAACTCCTAGATGCCAGGTCTAACTCCTAGAGCTGACCTCACTGACACATACAAAATCATTGCCATTGCTTTCTTGTTGTCAGCTTGCATTTACTTCCAAAATAGCCACTACCAACCTGTTGCTGGAGACAACTTGCACCGTTTGCCTTTTGGTGGCCAATATCAAGACGGCACCAAAAAGATATCTTATTTTCCACAACAGCAGTCATACTTTCATTCTGGAAACAAATTAAATGTCCTCATACTTATCTTCATTCTCACGTTGGGTATTGTCCTCACCAATAAATTTAGTTTTAGCTTTAGTCGTACTACTCACCAGCATTCTTGCTATAACACACATTCAGCAACCAACAATACACAACCATTGTCAGGTCATCATTGAATGTCCTCATACTTATCTTCATTCTCACGTTGGGTATTGTCCTCACCAATAAATTTAGTTTTAGCTTTAGTCGTACTACTCACCAGCATTCTTGCTATAACACACATTCAGCAACCAACAATACACAACCATTGTCAGGTCATCATTGACGGTGCTGCAATAGTCATAACAAATTGTGAGAACACACCAGAAGTGCTTAAAGCAATAAACTTCTCCCCTTGGAACGGGTTAAGTTTTCCTAAATTTGAAAATTAAATGCCTGACACAACACCTGTTGCTGCCACTTCAAGTGCACCACCCACAGCCAAAGATGCTGGTGCCAAAGCTCCTTCTGACTTCTCAAATCCCAATACAGCTCCTAGTCTCAGTGATTTGAAGAAAGTCAAGTATGTCTCCACCGTGACCTCCGTGGCCACACCAGCTGAAATTGAAGCCCTAGGCAAAATCTTCACCGCTATGGGCCTTGCCGCCAATGAGACTGGTCCGGCCATGTGGGATCTAGCTCGTGCATATGCTGATGTGCAGAGTTCTAAATCGGCACAGCTGATTGGAGCTACCCCTTCCAACCCTGCACTATCACGCCGAGCCCTTGCTGCTCAGTTTGATCGAATCAATATAACCCCCAGGCAATTTTGCATGTACTTTGCCAAAGTTGTTTGGAACATACTTCTCGACAGCAACATTCCACCAGCAAATTGGGCCAAACTTGGTTACCAAGAAGATACAAAATTTGCTGCATTTGACTTCTTCGATGGAGTCACCAACCCTGCCAGCCTGCAGCCTGCTGATGGTCTTATCAGGCAGCCAAATGAGAAAGAACTAGCTGCTCACTCCGTAGCTAAGTACGGCGCCTTGGCTAGGCAAAAGATCTCCACAGGTAATTATATTACCACACTTGGAGAAGTCACACGTGGACACATGGGAGGAGCTAACACCATGTACGCGATAGACGCACCCCCTGAACTTTAA

>Sp13_P8_c4

ATGGAAAGATCAACTCTGATTAATTTACTTCAATTGCACCACTTCGAGCCAAAACTCAGTGTTGAAGGAATCATAGTTGTGCACGGAATTGCAGGCACTGGGAAAACCACTTTACTTAGGACTTTATTTTCTGCTTACCCTAGCTTAGTTATAGGTTCACCTAGGCCTTGCTATTTAGATAAACAAAACAAAATTTCACAAGTTTGCTTATCTTGCTTTCCCAATACCCATTGTGATATTGTCGATGAGTATCATTTGCTAGAAAGTTTTCTAGAACCAAAATTGGCTATCTTTGGTGACCCCTGTCAATGCACATACATTGAGAGACTTAGAGTCCCACATTACACTTCCTTCAGAACTCATAGATTTGGAAAGTCAACTGCTGAGATTTTGAACAAACTGTTTGACCTTAATATAGTCTCAGTTAAGAAAGAAGACGACATCGTTGAATTCTTTAACCCTTTTGAAGTTGACCCCACTGAGCATATCTCTGCCTCTGAAGAAGAAGTCTTGGACTTTGTTTCTGACCAAGTGGTGACCACTAGCTCAGAGGAACTAGCAGGACTTGAGTTTGCAGAAACAACTTTCTACTGCACAACATTGGCCGCAGCTGTTGCTGAAAATCCTGCTAAGACTTTCATCTCTCTGACTAGACACACCCACAAACTCACCATTGGGGAACTAAATGCCAGGTCTAACTCCTAGATGCCAGGTCTAACTCCTAGAGCTGACCTCACTGACACATACAAAATCATTGCCATTGCTTTCTTGTTGTCAGCTTGCATTTACTTCCAAAATAGCCACTACCAACCTGTTGCTGGAGACAACTTGCACCGTTTGCCTTTTGGTGGCCAATATCAAGACGGCACCAAAAAGATATCTTATTTTCCACAACAGCAGTCATACTTTCATTCTGGAAACAAATTAAATGTCCTCATACTTATCTTCATTCTCACGTTGGGTATTGTCCTCACCAATAAATTTAGTTTTAGCTTTAGTCGTACTACTCACCAGCATTCTTGCTATAACACACATTCAGCAACCAACAATACACAACCATTGTCAGGTCATCATTGAATGTCCTCATACTTATCTTCATTCTCACGTTGGGTATTGTCCTCACCAATAAATTTAGTTTTAGCTTTAGTCGTACTACTCACCAGCATTCTTGCTATAACACACATTCAGCAACCAACAATACACAACCATTGTCAGGTCATCATTGACGGTGCTGCAATAGTCATAACAAATTGTGAGAACACACCAGAAGTGCTTAAAGCAATAAACTTCTCCCCTTGGAACGGGTTAAGTTTTCCTAAATTTGAAAATTAAATGCCTGACACAACACCTGTTGCTGCCACTTCAAGTGCACCACCCACAGCCAAAGATGCTGGTGCCAAAGCTCCTTCTGACTTCTCAAATCCCAATACAGCTCCTAGTCTCAGTGATTTGAAGAAAGTCAAGTATGTCTCCACCGTGACCTCCGTGGCCACACCAGCTGAAATTGAAGCCCTAGGCAAAATCTTCACCGCTATGGGCCTTGCCGCCAATGAGACTGGTCCGGCCATGTGGGATCTAGCTCGTGCATATGCTGATGTGCAGAGTTCTAAATCGGCACAGCTGATTGGAGCTACCCCTTCCAACCCTGCACTATCACGCCGAGCCCTTGCTGCTCAGTTTGATCGAATCAATATAACCCCCAGGCAATTTTGCATGTACTTTGCCAAAGTTGTTTGGAACATACTTCTCGACAGCAACATTCCACCAGCAAATTGGGCCAAACTTGGTTACCAAGAAGATACAAAATTTGCTGCATTTGACTTCTTCGATGGAGTCACCAACCCTGCCAGCCTGCAGCCTGCTGATGGTCTTATCAGGCAGCCAAATGAGAAAGAACTAGCTGCTCACTCCGTAGCTAAGTACGGCGCCTTGGCTAGGCAAAAGATCTCCACAGGTAATTATATTACCACACTTGGAGAAGTCACACGTGGACACATGGGAGGAGCTAACACCATGTACGCGATAGACGCACCCCCTGAACTTTAA

>Sp13_P8_c3

ATGGAAAGATCAACTCTGATTAATTTACTTCAATTGCACCACTTCGAGCCAAAACTCAGTGTTGAAGGAATCATAGTTGTGCACGGAATTGCAGGCACTGGGAAAACCACTTTACTTAGGACTTTATTTTCTGCTTACCCTAGCTTAGTTATAGGTTCACCTAGGCCTTGCTATTTAGATAAACAAAACAAAATTTCACAAGTTTGCTTATCTTGCTTTCCCAATACCCATTGTGATATTGTCGATGAGTATCATTTGCTAGAAAGTTTTCTAGAACCAAAATTGGCTATCTTTGGTGACCCCTGTCAATGCACATACATTGAGAGACTTAGAGTCCCACATTACACTTCCTTCAGAACTCATAGATTTGGAAAGTCAACTGCTGAGATTTTGAACAAACTGTTTGACCTTAATATAGTCTCAGTTAAGAAAGAAGACGACATCGTTGAATTCTTTAACCCTTTTGAAGTTGACCCCACTGAGCATATCTCTGCCTCTGAAGAAGAAGTCTTGGACTTTGTTTCTGACCAAGTGGTGACCACTAGCTCAGAGGAACTAGCAGGACTTGAGTTTGCAGAAACAACTTTCTACTGCACAACATTGGCCGCAGCTGTTGCTGAAAATCCTGCTAAGACTTTCATCTCTCTGACTAGACACACCCACAAACTCACCATTGGGGAACTAAATGCCAGGTCTAACTCCTAGATGCCAGGTCTAACTCCTAGAGCTGACCTCACTGACACATACAAAATCATTGCCATTGCTTTCTTGTTGTCAGCTTGCATTTACTTCCAAAATAGCCACTACCAACCTGTTGCTGGAGACAACTTGCACCGTTTGCCTTTTGGTGGCCAATATCAAGACGGCACCAAAAAGATATCTTATTTTCCACAACAGCAGTCATACTTTCATTCTGGAAACAAATTAAATGTCCTCATACTTATCTTCATTCTCACGTTGGGTATTGTCCTCACCAATAAATTTAGTTTTAGCTTTAGTCGTACTACTCACCAGCATTCTTGCTATAACACACATTCAGCAACCAACAATACACAACCATTGTCAGGTCATCATTGAATGTCCTCATACTTATCTTCATTCTCACGTTGGGTATTGTCCTCACCAATAAATTTAGTTTTAGCTTTAGTCGTACTACTCACCAGCATTCTTGCTATAACACACATTCAGCAACCAACAATACACAACCATTGTCAGGTCATCATTGACGGTGCTGCAATAGTCATAACAAATTGTGAGAACACACCAGAAGTGCTTAAAGCAATAAACTTCTCCCCTTGGAACGGGTTAAGTTTTCCTAAATTTGAAAATTAAATGCCTGACACAACACCTGTTGCTGCCACTTCAAGTGCACCACCCACAGCCAAAGATGCTGGTGCCAAAGCTCCTTCTGACTTCTCAAATCCCAATACAGCTCCTAGTCTCAGTGATTTGAAGAAAGTCAAGTATGTCTCCACCGTGACCTCCGTGGCCACACCAGCTGAAATTGAAGCCCTAGGCAAAATCTTCACCGCTATGGGCCTTGCCGCCAATGAGACTGGTCCGGCCATGTGGGATCTAGCTCGTGCATATGCTGATGTGCAGAGTTCTAAATCGGCACAGCTGATTGGAGCTACCCCTTCCAACCCTGCACTATCACGCCGAGCCCTTGCTGCTCAGTTTGATCGAATCAATATAACCCCCAGGCAATTTTGCATGTACTTTGCCAAAGTTGTTTGGAACATACTTCTCGACAGCAACATTCCACCAGCAAATTGGGCCAAACTTGGTTACCAAGAAGATACAAAATTTGCTGCATTTGACTTCTTCGATGGAGTCACCAACCCTGCCAGCCTGCAGCCTGCTGATGGTCTTATCAGGCAGCCAAATGAGAAAGAACTAGCTGCTCACTCCGTAGCTAAGTACGGCGCCTTGGCTAGGCAAAAGATCTCCACAGGTAATTATATTACCACACTTGGAGAAGTCACACGTGGACACATGGGAGGAGCTAACACCATGTACGCGATAGACGCACCCCCTGAACTTTAA

>Sp13_P8_c2

ATGGAAAGATCAACTCTGATTAATTTACTTCAATTGCACCACTTCGAGCCAAAACTCAGTGTTGAAGGAATCATAGTTGTGCACGGAATTGCAGGCACTGGGAAAACCACTTTACTTAGGACTTTATTTTCTGCTTACCCTAGCTTAGTTATAGGTTCACCTAGGCCTTGCTATTTAGATAAACAAAACAAAATTTCACAAGTTTGCTTATCTTGCTTTCCCAATACCCATTGTGATATTGTCGATGAGTATCATTTGCTAGAAAGTTTTCTAGAACCAAAATTGGCTATCTTTGGTGACCCCTGTCAATGCACATACATTGAGAGACTTAGAGTCCCACATTACACTTCCTTCAGAACTCATAGATTTGGAAAGTCAACTGCTGAGATTTTGAACAAACTGTTTGACCTTAATATAGTCTCAGTTAAGAAAGAAGACGACATCGTTGAATTCTTTAACCCTTTTGAAGTTGACCCCACTGAGCATATCTCTGCCTCTGAAGAAGAAGTCTTGGACTTTGTTTCTGACCAAGTGGTGACCACTAGCTCAGAGGAACTAGCAGGACTTGAGTTTGCAGAAACAACTTTCTACTGCACAACATTGGCCGCAGCTGTTGCTGAAAATCCTGCTAAGACTTTCATCTCTCTGACTAGACACACCCACAAACTCACCATTGGGGAACTAAATGCCAGGTCTAACTCCTAGATGCCAGGTCTAACTCCTAGAGCTGACCTCACTGACACATACAAAATCATTGCCATTGCTTTCTTGTTGTCAGCTTGCATTTACTTCCAAAATAGCCACTACCAACCTGTTGCTGGAGACAACTTGCACCGTTTGCCTTTTGGTGGCCAATATCAAGACGGCACCAAAAAGATATCTTATTTTCCACAACAGCAGTCATACTTTCATTCTGGAAACAAATTAAATGTCCTCATACTTATCTTCATTCTCACGTTGGGTATTGTCCTCACCAATAAATTTAGTTTTAGCTTTAGTCGTACTACTCACCAGCATTCTTGCTATAACACACATTCAGCAACCAACAATACACAACCATTGTCAGGTCATCATTGAATGTCCTCATACTTATCTTCATTCTCACGTTGGGTATTGTCCTCACCAATAAATTTAGTTTTAGCTTTAGTCGTACTACTCACCAGCATTCTTGCTATAACACACATTCAGCAACCAACAATACACAACCATTGTCAGGTCATCATTGACGGTGCTGCAATAGTCATAACAAATTGTGAGAACACACCAGAAGTGCTTAAAGCAATAAACTTCTCCCCTTGGAACGGGTTAAGTTTTCCTAAATTTGAAAATTAAATGCCTGACACAACACCTGTTGCTGCCACTTCAAGTGCACCACCCACAGCCAAAGATGCTGGTGCCAAAGCTCCTTCTGACTTCTCAAATCCCAATACAGCTCCTAGTCTCAGTGATTTGAAGAAAGTCAAGTATGTCTCCACCGTGACCTCCGTGGCCACACCAGCTGAAATTGAAGCCCTAGGCAAAATCTTCACCGCTATGGGCCTTGCCGCCAATGAGACTGGTCCGGCCATGTGGGATCTAGCTCGTGCATATGCTGATGTGCAGAGTTCTAAATCGGCACAGCTGATTGGAGCTACCCCTTCCAACCCTGCACTATCACGCCGAGCCCTTGCTGCTCAGTTTGATCGAATCAATATAACCCCCAGGCAATTTTGCATGTACTTTGCCAAAGTTGTTTGGAACATACTTCTCGACAGCAACATTCCACCAGCAAATTGGGCCAAACTTGGTTACCAAGAAGATACAAAATTTGCTGCATTTGACTTCTTCGATGGAGTCACCAACCCTGCCAGCCTGCAGCCTGCTGATGGTCTTATCAGGCAGCCAAATGAGAAAGAACTAGCTGCTCACTCCGTAGCTAAGTACGGCGCCTTGGCTAGGCAAAAGATCTCCACAGGTAATTATATTACCACACTTGGAGAAGTCACACGTGGACACATGGGAGGAGCTAACACCATGTACGCGATAGACGCACCCCCTGAACTTTAA

>Sp13_P8_c1

ATGGAAAGATCAACTCTGATTAATTTACTTCAATTGCACCACTTCGAGCCAAAACTCAGTGTTGAAGGAATCATAGTTGTGCACGGAATTGCAGGCACTGGGAAAACCACTTTACTTAGGACTTTATTTTCTGCTTACCCTAGCTTAGTTATAGGTTCACCTAGGCCTTGCTATTTAGATAAACAAAACAAAATTTCACAAGTTTGCTTATCTTGCTTTCCCAATACCCATTGTGATATTGTCGATGAGTATCATTTGCTAGAAAGTTTTCTAGAACCAAAATTGGCTATCTTTGGTGACCCCTGTCAATGCACATACATTGAGAGACTTAGAGTCCCACATTACACTTCCTTCAGAACTCATAGATTTGGAAAGTCAACTGCTGAGATTTTGAACAAACTGTTTGACCTTAATATAGTCTCAGTTAAGAAAGAAGACGACATCGTTGAATTCTTTAACCCTTTTGAAGTTGACCCCACTGAGCATATCTCTGCCTCTGAAGAAGAAGTCTTGGACTTTGTTTCTGACCAAGTGGTGACCACTAGCTCAGAGGAACTAGCAGGACTTGAGTTTGCAGAAACAACTTTCTACTGCACAACATTGGCCGCAGCTGTTGCTGAAAATCCTGCTAAGACTTTCATCTCTCTGACTAGACACACCCACAAACTCACCATTGGGGAACTAAATGCCAGGTCTAACTCCTAGATGCCAGGTCTAACTCCTAGAGCTGACCTCACTGACACATACAAAATCATTGCCATTGCTTTCTTGTTGTCAGCTTGCATTTACTTCCAAAATAGCCACTACCAACCTGTTGCTGGAGACAACTTGCACCGTTTGCCTTTTGGTGGCCAATATCAAGACGGCACCAAAAAGATATCTTATTTTCCACAACAGCAGTCATACTTTCATTCTGGAAACAAATTAAATGTCCTCATACTTATCTTCATTCTCACGTTGGGTATTGTCCTCACCAATAAATTTAGTTTTAGCTTTAGTCGTACTACTCACCAGCATTCTTGCTATAACACACATTCAGCAACCAACAATACACAACCATTGTCAGGTCATCATTGAATGTCCTCATACTTATCTTCATTCTCACGTTGGGTATTGTCCTCACCAATAAATTTAGTTTTAGCTTTAGTCGTACTACTCACCAGCATTCTTGCTATAACACACATTCAGCAACCAACAATACACAACCATTGTCAGGTCATCATTGACGGTGCTGCAATAGTCATAACAAATTGTGAGAACACACCAGAAGTGCTTAAAGCAATAAACTTCTCCCCTTGGAACGGGTTAAGTTTTCCTAAATTTGAAAATTAAATGCCTGACACAACACCTGTTGCTGCCACTTCAAGTGCACCACCCACAGCCAAAGATGCTGGTGCCAAAGCTCCTTCTGACTTCTCAAATCCCAATACAGCTCCTAGTCTCAGTGATTTGAAGAAAGTCAAGTATGTCTCCACCGTGACCTCCGTGGCCACACCAGCTGAAATTGAAGCCCTAGGCAAAATCTTCACCGCTATGGGCCTTGCCGCCAATGAGACTGGTCCGGCCATGTGGGATCTAGCTCGTGCATATGCTGATGTGCAGAGTTCTAAATCGGCACAGCTGATTGGAGCTACCCCTTCCAACCCTGCACTATCACGCCGAGCCCTTGCTGCTCAGTTTGATCGAATCAATATAACCCCCAGGCAATTTTGCATGTACTTTGCCAAAGTTGTTTGGAACATACTTCTCGACAGCAACATTCCACCAGCAAATTGGGCCAAACTTGGTTACCAAGAAGATACAAAATTTACTGCATTTGACTTCTTCGGTGGAGTCACCAACCCTGCCAGCCTGCAGCCTGCTGATGGTCTTATCAGGCAGCCAAATGAGAAAGAACTAGCTGCTCACTCCGTAGCTAAGTACGGCGCCTTGGCTAGGCAAAAGATCTCCACAGGTAATTATATTACCACACTTGGAGAAGTCACACGTGGACACATGGGAGGAGCTAACACCATGTACGCGATAGACGCACCCCCTGAACTTTAA

>Sp13_P8_b14

ATGGAAAGATCAACTCTGATTAATTTACTTCAATTGCACCACTTCGAGCCAAAACTCAGTGTTGAAGGAATCATAGTTGTGCACGGAATTGCAGGCACTGGGAAAACCACTTTACTTAGGACTTTATTTTCTGCTTACCCTAGCTTAGTTATAGGTTCACCTAGGCCTTGCTATTTAGATAAACAAAACAAAATTTCACAAGTTTGCTTATCTTGCTTTCCCAATACCCATTGTGATATTGTCGATGAGTATCATTTGCTAGAAAGTTTTCTAGAACCAAAATTGGCTATCTTTGGTGACCCCTGTCAATGCACATACATTGAGAGACTTAGAGTCCCACATTACACTTCCTTCAGAACTCATAGATTTGGAAAGTCAACTGCTGAGATTTTGAACAAACTGTTTGACCTTAATATAGTCTCAGTTAAGAAAGAAGACGACATCGTTGAATTCTTTAACCCGTTTGAAGTTGACCCCACTGAGCATATCTCTGCCTCTGAAGAAGAAGTCTTGGACTTTGTTTCTGACCAAGTGGTGACCACTAGCTCAGAGGAACTAGCAGGACTTGAGTTTGCAGAAACAACTTTCTACTGCACAACATTGGCCGCAGCTGTTGCTGAAAATCCTGCTAAGACTTTCATCTCTCTGACTAGACACACCCACAAACTCACCATTGGGGAACTAAATGCCAGGTCTAACTCCTAGATGCCAGGTCTAACTCCTAGAGCTGACCTCACTGACACATACAAAATCATTGCCATTGCTTTCTTGTTGTCAGCTTGCATTTACTTCCAAAATAGCCACTACCAACCTGTTGCTGGAGACAACTTGCACCGTTTGCCTTTTGGTGGCCAATATCAAGACGGCACCAAAAAGATATCTTATTTTCCACAACAGCAGTCATACTTTCATTCTGGAAACAAATTAAATGTCCTCATACTTATCTTCATTCTCACGTTGGGTATTGTCCTCACCAATAAATTTAGTTTTAGCTTTAGTCGTACTACTCACCAGCATTCTTGCTATAACACACATTCAGCAACCAACAATACACAACCATTGTCAGGTCATCATTGAATGTCCTCATACTTATCTTCATTCTCACGTTGGGTATTGTCCTCACCAATAAATTTAGTTTTAGCTTTAGTCGTACTACTCACCAGCATTCTTGCTATAACACACATTCAGCAACCAACAATACACAACCATTGTCAGGTCATCATTGACGGTGCTGCAATAGTCATAACAAATTGTGAGAACACACCAGAAGTGCTTAAAGCAATAAACTTCTCCCCTTGGAACGGGTTAAGTTTTCCTAAATTTGAAAATTAAATGCCTGACACAACACCTGTTGCTGCCACTTCAAGTGCACCACCCACAGCCAAAGATGCTGGTGCCAAAGCTCCTTCTGACTTCTCAAATCCCAATACAGCTCCTAGTCTCAGTGATTTGAAGAAAGTCAAGTATGTCTCCACCGTGACCTCCGTGGCCACACCAGCTGAAATTGAAGCCCTAGGCAAAATCTTCACCGCTATGGGCCTTGCCGCCAATGAGACTGGTCCGGCCATGTGGGATCTAGCTCGTGCATATGCTGATGTGCAGAGTTCTAAATCGGCACAGCTGATTGGAGCTACCCCTTCCAACCCTGCACTATCACGCCGAGCCCTTGCTGCTCAGTTTGATCGAATCAATATAACCCCCAGGCAATTTTGCATGTACTTTGCCAAAGTTGTTTGGAACATACTTCTCGACAGCAACATTCCACCAGCAAATTGGGCCAAACTTGGTTACCAAGAAGATACAAAATTTGCTGCATTTGACTTCTTCGATGGAGTCACCAACCCTGCCAGCCTGCAGCCTGCTGATGGTCTTATCAGGCAGCCAAATGAGAAAGAACTAGCTGCTCACTCCGTAGCTAAGTACGGCGCCTTGGCTAGACAAAAGATCTCCACAGGTAATTATATTACCACACTTGGAGAAGTCACACGTGGACACATGGGAGGAGCTAACACCATGTACGCGATAGACGCACCCCCTGAACTTTAA

>Sp13_P8_b13

ATGGAAAGATCAACTCTGATTAATTTACTTCAATTGCACCACTTCGAGCCAAAACTCAGTGTTGAAGGAATCATAGTTGTGCACGGAATTGCAGGCACTGGGAAAACCACTTTACTTAGGACTTTATTTTCTGCTTACCCTAGCTTAGTTATAGGTTCACCTAGGCCTTGCTATTTAGATAAACAAAACAAAATTTCACAAGTTTGCTTATCTTGCTTTCCCAATACCCATTGTGATATTGTCGATGAGTATCATTTGCTAGAAAGTTTTCTAGAACCAAAATTGGCTATCTTTGGTGACCCCTGTCAATGCACATACATTGAGAGACTTAGAGTCCCACATTACACTTCCTTCAGAACTCATAGATTTGGAAAGTCAACTGCTGAGATTTTGAACAAACTGTTTGACCTTAATATAGTCTCAGTTAAGAAAGAAGACGACATCGTTGAATTCTTTAACCCGTTTGAAGTTGACCCCACTGAGCATATCTCTGCCTCTGAAGAAGAAGTCTTGGACTTTGTTTCTGACCAAGTGGTGACCACTAGCTCAGAGGAACTAGCAGGACTTGAGTTTGCAGAAACAACTTTCTACTGCACAACATTGGCCGCAGCTGTTGCTGAAAATCCTGCTAAGACTTTCATCTCTCTGACTAGACACACCCACAAACTCACCATTGGGGAACTAAATGCCAGGTCTAACTCCTAGATGCCAGGTCTAACTCCTAGAGCTGACCTCACTGACACATACAAAATCATTGCCATTGCTTTCTTGTTGTCAGCTTGCATTTACTTCCAAAATAGCCACTACCAACCTGTTGCTGGAGACAACTTGCACCGTTTGCCTTTTGGTGGCCAATATCAAGACGGCACCAAAAAGATATCTTATTTTCCACAACAGCAGTCATACTTTCATTCTGGAAACAAATTAAATGTCCTCATACTTATCTTCATTCTCACGTTGGGTATTGTCCTCACCAATAAATTTAGTTTTAGCTTTAGTCGTACTACTCACCAGCATTCTTGCTATAACACACATTCAGCAACCAACAATACACAACCATTGTCAGGTCATCATTGAATGTCCTCATACTTATCTTCATTCTCACGTTGGGTATTGTCCTCACCAATAAATTTAGTTTTAGCTTTAGTCGTACTACTCACCAGCATTCTTGCTATAACACACATTCAGCAACCAACAATACACAACCATTGTCAGGTCATCATTGACGGTGCTGCAATAGTCATAACAAATTGTGAGAACACACCAGAAGTGCTTAAAGCAATAAACTTCTCCCCTTGGAACGGGTTAAGTTTTCCTAAATTTGAAAATTAAATGCCTGACACAACACCTGTTGCTGCCACTTCAAGTGCACCACCCACAGCCAAAGATGCTGGTGCCAAAGCTCCTTCTGACTTCTCAAATCCCAATACAGCTCCTAGTCTCAGTGATTTGAAGAAAGTCAAGTATGTCTCCACCGTGACCTCCGTGGCCACACCAGCTGAAATTGAAGCCCTAGGCAAAATCTTCACCGCTATGGGCCTTGCCGCCAATGAGACTGGTCCGGCCATGTGGGATCTAGCTCGTGCATATGCTGATGTGCAGAGTTCTAAATCGGCACAGCTGATTGGAGCTACCCCTTCCAACCCTGCACTATCACGCCGAGCCCTTGCTGCTCAGTTTGATCGAATCAATATAACCCCCAGGCAATTTTGCATGTACTTTGCCAAAGTTGTTTGGAACATACTTCTCGACAGCAACATTCCACCAGCAAATTGGGCCAAACTTGGTTACCAAGAAGATACAAAATTTGCTGCATTTGACTTCTTCGATGGAGTCACCAACCCTGCCAGCCTGCAGCCTGCTGATGGTCTTATCAGGCAGCCAAATGAGAAAGAACTAGCTGCTCACTCCGTAGCTAAGTACGGCGCCTTGGCTAGGCAAAAGATCTCCACAGGTAATTATATTACCACACTTGGAGAAGTCACACGTGGACACATGGGAGGAGCTAACACCATGTACGCGATAGACGCACCCCCTGAACTTTAA

>Sp13_P8_b12

ATGGAAAGATCAACTCTGATTAATTTACTTCAATTGCACCACTTCGAGCCAAAACTCAGTGTTGAAGGAATCATAGTTGTGCACGGAATTGCAGGCACTGGGAAAACCACTTTACTTAGGACTTTATTTTCTGCTTACCCTAGCTTAGTTATAGGTTCACCTAGGCCTTGCTATTTAGATAAACAAAACAAAATTTCACAAGTTTGCTTATCTTGCTTTCCCAATACCCATTGTGATATTGTCGATGAGTATCATTTGCTAGAAAGTTTTCTAGAACCAAAATTGGCTATCTTTGGTGACCCCTGTCAATGCACATACATTGAGAGACTTAGAGTCCCACATTACACTTCCTTCAGAACTCATAGATTTGGAAAGTCAACTGCTGAGATTTTGAACAAACTGTTTGACCTTAATATAGTCTCAGTTAAGAAAGAAGACGACATTGTTGAATTCTTTAACCCTTTTGAAGTTGACCCCACTGAGCATATCTCTGCCTCTGAAGAAGAAGTCTTGGACTTTGTTTCTGACCAAGTGGTGACCACTAGCTCAGAGGAACTAGCAGGACTTGAGTTTGCAGAAACAACTTTCTACTGCACAACATTGGCCGCAGCTGTTGCTGAAAATCCTGCTAAGACTTTCATCTCTCTGACTAGACACACCCACAAACTCACCATTGGGGAACTAAATGCCAGGTCTAACTCCTAGATGCCAGGTCTAACTCCTAGAGCTGACCTCACTGACACATACAAAATCATTGCCATTGCTTTCTTGTTGTCAGCTTGCATTTACTTCCAAAATAGCCACTACCAACCTGTTGCTGGAGACAACTTGCACCGTTTGCCTTTTGGTGGCCAATATCAAGACGGCACCAAAAAGATATCTTATTTTCCACAACAGCAGTCATACTTTCATTCTGGAAACAAATTAAATGTCCTCATACTTATCTTCATTCTCACGTTGGGTATTGTCCTCACCAATAAATTTAGTTTTAGCTTTAGTCGTACTACTCACCAGCATTCTTGCTATAACACACATTCAGCAACCAACAATACACAACCATTGTCAGGTCATCATTGAATGTCCTCATACTTATCTTCATTCTCACGTTGGGTATTGTCCTCACCAATAAATTTAGTTTTAGCTTTAGTCGTACTACTCACCAGCATTCTTGCTATAACACACATTCAGCAACCAACAATACACAACCATTGTCAGGTCATCATTGACGGTGCTGCAATAGTCATAACAAATTGTGAGAACACACCAGAAGTGCTTAAAGCAATAAACTTCTCCCCTTGGAACGGGTTAAGTTTTCCTAAATTTGAAAATTAAATGCCTGACACAACACCTGTTGCTGCCACTTCAAGTGCACCACCCACAGCCAAAGATGCTGGTGCCAAAGCTCCTTCTGACTTCTCAAATCCCAATACAGCTCCTAGTCTCAGTGATTTGAAGAAAGTCAAGTATGTCTCCACCGTGACCTCCGTGGCCACACCAGCTGAAATTGAAGCCCTAGGCAAAATCTTCACCGCTATGGGCCTTGCCGCCAATGAGACTGGTCCGGCCATGTGGGATCTAGCTCGTGCATATGCTGATGTGCAGAGTTCTAAATCGGCACAGCTGATTGGAGCTACCCCTTCCAACCCTGCACTATCACGCCGAGCCCTTGCTGCTCAGTTTGATCGAATCAATATAACCCCCAGGCAATTTTGCATGTACTTTGCCAAAGTTGTTTGGAACATACTTCTCGACAGCAACATTCCACCAGCAAATTGGGCCAAACTTGGTTACCAAGAAGATACAAAATTTGCTGCATTTGACTTCTTCGATGGAGTCACCAACCCTGCCAGCCTGCAGCCTGCTGATGGTCTTATCAGGCAGCCAAATGAGAAAGAACTAGCTGCTCACTCCGTAGCTAAGTACGGCGCCTTGGCTAGGCAAAAGATCTCCACAGGTAATTATATTACCACACTTGGAGAAGTCACACGTGGACACATGGGAGGAGCTAACACCATGTACGCGATAGACGCACCCCCTGAACTTTAA

>Sp13_P8_b11

ATGGAAAGATCAACTCTGATTAATTTACTTCAATTGCACCACTTCGAGCCAAAACTCAGTGTTGAAGGAATCATAGTTGTGCACGGAATTGCAGGCACTGGGAAAACCACTTTACTTAGGACTTTATTTTCTGCTTACCCTAGCTTAGTTATAGGTTCACCTAGGCCTTGCTATTTAGATAAACAAAACAAAATTTCACAAGTTTGCTTATCTTGCTTTCCCAATACCCATTGTGATATTGTCGATGAGTATCATTTGCTAGAAAGTTTTCTAGAACCAAAATTGGCTATCTTTGGTGACCCCTGTCAATGCACATACATTGAGAGACTTAGAGTCCCACATTACACTTCCTTCAGAACTCATAGATTTGGAAAGTCAACTGCTGAGATTTTGAACAAACTGTTTGACCTTAATATAGTCTCAGTTAAGAAAGAAGACGACATCGTTGAATTCTTTAACCCTTTTGAAGTTGACCCCACTGAGCATATCTCTGCCTCTGAAGAAGAAGTCTTGGACTTTGTTTCTGACCAAGTGGTGACCACTAGCTCAGAGGAACTAGCAGGACTTGAGTTTGCAGAAACAACTTTCTACTGCACAACATTGGCCGCAGCTGTTGCTGAAAATCCTGCTAAGACTTTCATCTCTCTGACTAGACACACCCACAAACTCACCATTGGGGAACTAAATGCCAGGTCTAACTCCTAGATGCCAGGTCTAACTCCTAGAGCTGACCTCACTGACACATACAAAATCATTGCCATTGCTTTCTTGTTGTCAGCTTGCATTTACTTCCAAAATAGCCACTACCAACCTGTTGCTGGAGACAACTTGCACCGTTTGCCTTTTGGTGGCCAATATCAAGACGGCACCAAAAAGATATCTTATTTTCCACAACAGCAGTCATACTTTCATTCTGGAAACAAATTAAATGTCCTCATACTTATCTTCATTCTCACGTTGGGTATTGTCCTCACCAATAAATTTAGTTTTAGCTTTAGTCGTACTACTCACCAGCATTCTTGCTATAACACACATTCAGCAACCAACAATACACAACCATTGTCAGGTCATCATTGAATGTCCTCATACTTATCTTCATTCTCACGTTGGGTATTGTCCTCACCAATAAATTTAGTTTTAGCTTTAGTCGTACTACTCACCAGCATTCTTGCTATAACACACATTCAGCAACCAACAATACACAACCATTGTCAGGTCATCATTGACGGTGCTGCAATAGTCATAACAAATTGTGAGAACACACCAGAAGTGCTTAAAGCAATAAACTTCTCCCCTTGGAACGGGTTAAGTTTTCCTAAATTTGAAAATTAAATGCCTGACACAACACCTGTTGCTGCCACTTCAAGTGCACCACCCACAGCCAAAGATGCTGGTGCCAAAGCTCCTTCTGACTTCTCAAATCCCAATACAGCTCCTAGTCTCAGTGATTTGAAGAAAGTCAAGTATGTCTCCACCGTGACCTCCGTGGCCACACCAGCTGAAATTGAAGCCCTAGGCAAAATCTTCACCGCTATGGGCCTTGCCGCCAATGAGACTGGTCCGGCCATGTGGGATCTAGCTCGTGCATATGCTGATGTGCAGAGTTCTAAATCGGCACAGCTGATTGGAGCTACCCCTTCCAACCCTGCACTATCACGCCGAGCCCTTGCTGCTCAGTTTGATCGAATCAATATAACCCCCAGGCAATTTTGCATGTACTTTGCCAAAGTTGTTTGGAACATACTTCTCGACAGCAACATTCCACCAGCAAATTGGGCCAAACTTGGTTACCAAGAAGATACAAAATTTGCTGCATTTGACTTCTTCGATGGAGTCACCAACCCTGCCAGCCTGCAGCCTGCTGATGGTCTTATCAGGCAGCCAAATGAGAAAGAACTAGCTGCTCACTCCGTAGCTAAGTACGGCGCCTTGGCTAGGCAAAAGATCTCCACAGGTAATTATATTACCACACTTGGAGAAGTCACACGTGGACACATGGGAGGAGCTAACACCATGTACGCGATAGACGCACCCCCTGAACTTTAA

>Sp13_P8_b10

ATGGAAAGATCAACTCTGATTAATTTACTTCAATTGCACCACTTCGAGCCAAAACTCAGTGTTGAAGGAATCATAGTTGTGCACGGAATTGCAGGCACTGGGAAAACCACTTTACTTAGGACTTTATTTTCTGCTTACCCTAGCTTAGTTATAGGTTCACCTAGGCCTTGCTATTTAGATAAACAAAACAAAATTTCACAAGTTTGCTTATCTTGCTTTCCCAATACCCATTGTGATATTGTCGATGAGTATCATTTGCTAGAAAGTTTTCTAGAACCAAAATTGGCTATCTTTGGTGACCCCTGTCAATGCACATACATTGAGAGACTTAGAGTCCCACATTACACTTCCTTCAGAACTCATAGATTTGGAAAGTCAACTGCTGAGATTTTGAACAAACTGTTTGACCTTAATATAGTCTCAGTTAAGAAAGAAGACGACATCGTTGAATTCTTTAACCCTTTTGAAGTTGACCCCACTGAGCATATCTCTGCCTCTGAAGAAGAAGTCTTGGACTTTGTTTCTGACCAAGTGGTGACCACTAGCTCAGAGGAACTAGCAGGACTTGAGTTTGCAGAAACAACTTTCTACTGCACAACATTGGCCGCAGCTGTTGCTGAAAATCCTGCTAAGACTTTCATCTCTCTGACTAGACACACCCACAAACTCACCATTGGGGAACTAAATGCCAGGTCTAACTCCTAGATGCCAGGTCTAACTCCTAGAGCTGACCTCACTGACACATACAAAATCATTGCCATTGCTTTCTTGTTGTCAGCTTGCATTTACTTCCAAAATAGCCACTACCAACCTGTTGCTGGAGACAACTTGCACCGTTTGCCTTTTGGTGGCCAATATCAAGACGGCACCAAAAAGATATCTTATTTTCCACAACAGCAGTCATACTTTCATTCTGGAAACAAATTAAATGTCCTCATACTTATCTTCATTCTCACGTTGGGTATTGTCCTCACCAATAAATTTAGTTTTAGCTTTAGTCGTACTACTCACCAGCATTCTTGCTATAACACACATTCAGCAACCAACAATACACAACCATTGTCAGGTCATCATTGAATGTCCTCATACTTATCTTCATTCTCACGTTGGGTATTGTCCTCACCAATAAATTTAGTTTTAGCTTTAGTCGTACTACTCACCAGCATTCTTGCTATAACACACATTCAGCAACCAACAATACACAACCATTGTCAGGTCATCATTGACGGTGCTGCAATAGTCATAACAAATTGTGAGAACACACCAGAAGTGCTTAAAGCAATAAACTTCTCCCCTTGGAACGGGTTAAGTTTTCCTAAATTTGAAAATTAAATGCCTGACACAACACCTGTTGCTGCCACTTCAAGTGCACCACCCACAGCCAAAGATGCTGGTGCCAAAGCTCCTTCTGACTTCTCAAATCCCAATACAGCTCCTAGTCTCAGTGATTTGAAGAAAGTCAAGTATGTCTCCACCGTGACCTCCGTGGCCACACCAGCTGAAATTGAAGCCCTAGGCAAAATCTTCACCGCTATGGGCCTTGCCGCCAATGAGACTGGTCCGGCCATGTGGGATCTAGCTCGTGCATATGCTGATGTGCAGAGTTCTAAATCGGCACAGCTGATTGGAGCTACCCCTTCCAACCCTGCACTATCACGCCGAGCCCTTGCTGCTCAGTTTGATCGAATCAATATAACCCCCAGGCAATTTTGCATGTACTTTGCCAAAGTTGTTTGGAACATACTTCTCGACAGCAACATTCCACCAGCAAATTGGGCCAAACTTGGTTACCAAGAAGATACAAAATTTGCTGCATTTGACTTCTTCGATGGAGTCACCAACCCTGCCAGCCTGCAGCCTGCTGATGGTCTTATCAGGCAGCCAAATGAGAAAGAACTAGCTGCTCACTCCGTAGCTAAGTACGGCGCCTTGGCTAGGCAAAAGATCTCCACAGGTAATTATATTACCACACTTGGAGAAGTCACACGTGGACACATGGGAGGAGCTAACACCATGTACGCGATAGACGCACCCCCTGAACTTTAA

>Sp13_P8_b9

ATGGAAAGATCAACTCTGATTAATTTACTTCAATTGCACCACTTCGAGCCAAAACTCAGTGTTGAAGGAATCATAGTTGTGCACGGAATTGCAGGCACTGGGAAAACCACTTTACTTAGGACTTTATTTTCTGCTTACCCTAGCTTAGTTATAGGTTCACCTAGGCCTTGCTATTTAGATAAACAAAACAAAATTTCACAAGTTTGCTTATCTTGCTTTCCCAATACCCATTGTGATATTGTCGATGAGTATCATTTGCTAGAAAGTTTTCTAGAACCAAAATTGGCTATCTTTGGTGACCCCTGTCAATGCACATACATTGAGAGACTTAGAGTCCCACATTACACTTCCTTCAGAACTCATAGATTTGGAAAGTCAACTGCTGAGATTTTGAACAAACTGTTTGACCTTAATATAGTCTCAGTTAAGAAAGAAGACGACATCGTTGAATTCTTTAACCCTTTTGAAGTTGACCCCACTGAGCATATCTCTGCCTCTGAAGAAGAAGTCTTGGACTTTGTTTCTGACCAAGTGGTGACCACTAGCTCAGAGGAACTAGCAGGACTTGAGTTTGCAGAAACAACTTTCTACTGCACAACATTGGCCGCAGCTGTTGCTGAAAATCCTGCTAAGACTTTCATCTCTCTGACTAGACACACCCACAAACTCACCATTGGGGAACTAAATGCCAGGTCTAACTCCTAGATGCCAGGTCTAACTCCTAGAGCTGACCTCACTGACACATACAAAATCATTGCCATTGCTTTCTTGTTGTCAGCTTGCATTTACTTCCAAAATAGCCACTACCAACCTGTTGCTGGAGACAACTTGCACCGTTTGCCTTTTGGTGGCCAATATCAAGACGGCACCAAAAAGATATCTTATTTTCCACAACAGCAGTCATACTTTCATTCTGGAAACAAATTAAATGTCCTCATACTTATCTTCATTCTCACGTTGGGTATTGTCCTCACCAATAAATTTAGTTTTAGCTTTAGTCGTACTACTCACCAGCATTCTTGCTATAACACACATTCAGCAACCAACAATACACAACCATTGTCAGGTCATCATTGAATGTCCTCATACTTATCTTCATTCTCACGTTGGGTATTGTCCTCACCAATAAATTTAGTTTTAGCTTTAGTCGTACTACTCACCAGCATTCTTGCTATAACACACATTCAGCAACCAACAATACACAACCATTGTCAGGTCATCATTGACGGTGCTGCAATAGTCATAACAAATTGTGAGAACACACCAGAAGTGCTTAAAGCAATAAACTTCTCCCCTTGGAACGGGTTAAGTTTTCCTAAATTTGAAAATTAAATGCCTGACACAACACCTGTTGCTGCCACTTCAAGTGCACCACCCACAGCCAAAGATGCTGGTGCCAAAGCTCCTTCTGACTTCTCAAATCCCAATACAGCTCCTAGTCTCAGTGATTTGAAGAAAGTCAAGTATGTCTCCACCGTGACCTCCGTGGCCACACCAGCTGAAATTGAAGCCCTAGGCAAAATCTTCACCGCTATGGGCCTTGCCGCCAATGAGACTGGTCCGGCCATGTGGGATCTAGCTCGTGCATATGCTGATGTGCAGAGTTCTAAATCGGCACAGCTGATTGGAGCTACCCCTTCCAACCCTGCACTATCACGCCGAGCCCTTGCTGCTCAGTTTGATCGAATCAATATAACCCCCAGGCAATTTTGCATGTACTTTGCCAAAGTTGTTTGGAACATACTTCTCGACAGCAACATTCCACCAGCAAATTGGGCCAAACTTGGTTACCAAGAAGATACAAAATTTGCTGCATTTGACTTCTTCGATGGAGTCACCAACCCTGCCAGCCTGCAGCCTGCTGATGGTCTTATCAGGCAGCCAAATGAGAAAGAACTAGCTGCTCACTCCGTAGCTAAGTACGGCGCCTTGGCTAGGCAAAAGATCTCCACAGGTAATTATATTACCACACTTGGAGAAGTCACACGTGGACACATGGGAGGAGCTAACACCATGTACGCGATAGACGCACCCCCTGAACTTTAA

>Sp13_P8_b8

ATGGAAAGATCAACTCTGATTAATTTACTTCAATTGCACCACTTCGAGCCAAAACTCAGTGTTGAAGGAATCATAGTTGTGCACGGAATTGCAGGCACTGGGAAAACCACTTTACTTAGGACTTTATTTTCTGCTTACCCTAGCTTAGTTATAGGTTCACCTAGGCCTTGCTATTTAGATAAACAAAACAAAATTTCACAAGTTTGCTTATCTTGCTTTCCCAATACCCATTGTGATATTGTCGATGAGTATCATTTGCTAGAAAGTTTTCTAGAACCAAAATTGGCTATCTTTGGTGACCCCTGTCAATGCACATACATTGAGAGACTTAGAGTCCCACATTACACTTCCTTCAGAACTCATAGATTTGGAAAGTCAACTGCCGAGATTTTGAACAAACTGTTTGACCTTAATATAGTCTCAGTTAAGAAAGAAGACGACATCGTTGAATTCTTTAACCCTTTTGAAGTTGACCCCACTGAGCATATCTCTGCCTCTGAAGAAGAAGTCTTGGACTTTGTTTCTGACCAAGTGGTGACCACTAGCTCAGAGGAACTAGCAGGACTTGAGTTTGCAGAAACAACTTTCTACTGCACAACATTGGCCGCAGCTGTTGCTGAAAATCCTGCTAAGACTTTCATCTCTCTGACTAGACACACCCACAAACTCACCATTGGGGAACTAAATGCCAGGTCTAACTCCTAGATGCCAGGTCTAACTCCTAGAGCTGACCTCACTGACACATACAAAATCATTGCCATTGCTTTCTTGTTGTCAGCTTGCATTTACTTCCAAAATAGCCACTACCAACCTGTTGCTGGAGACAACTTGCACCGTTTGCCTTTTGGTGGCCAATATCAAGACGGCACCAAAAAGATATCTTATTTTCCACAACAGCAGTCATACTTTCATTCTGGAAACAAATTAAATGTCCTCATACTTATCTTCATTCTCACGTTGGGTATTGTCCTCACCAATAAATTTAGTTTTAGCTTTAGTCGTACTACTCACCAGCATTCTTGCTATAACACACATTCAGCAACCAACAATACACAGCCATTGTCAGGTCATCATTGAATGTCCTCATACTTATCTTCATTCTCACGTTGGGTATTGTCCTCACCAATAAATTTAGTTTTAGCTTTAGTCGTACTACTCACCAGCATTCTTGCTATAACACACATTCAGCAACCAACAATACACAGCCATTGTCAGGTCATCATTGACGGTGCTGCAATAGTCATAACAAATTGTGAGAACACACCAGAAGTGCTTAAAGCAATAAACTTCTCCCCTTGGAACGGGTTAAGTTTTCCTAAATTTGAAAATTAAATGCCTGACACAACACCTGTTGCTGCCACTTCAAGTGCACCACCCACAGCCAAAGATGCTGGTGCCAAAGCTCCTTCTGACTTCTCAAATCCCAATACAGCTCCTAGTCTCAGTGATTTGAAGAAAGTCAAGTATGTCTCCACCGTGACCTCCGTGGCCACACCAGCTGAAATTGAAGCCCTAGGCAAAATCTTCACCGCTATGGGCCTTGCCGCCAATGAGACTGGTCCGGCCATGTGGGATCTAGCTCGTGCATATGCTGATGTGCAGAGTTCTAAATCGGCACAGCTGATTGGAGCTACCCCTTCCAACCCTGCACTATCACGCCGAGCCCTTGCTGCTCAGTTTGATCGAATCAATATAACCCCCAGGCAATTTTGCATGTACTTTGCCAAAGTTGTTTGGAACATACTTCTCGACAGCAACATTCCACCAGCAAATTGGGCCAAACTTGGTTACCAAGAAGATACAAAATTTGCTGCATTTGACTTCTTCGATGGAGTCACCAACCCTGCCAGCCTGCAGCCTGCTGATGGTCTTATCAGGCAGCCAAATGAGAAAGAACTAGCTGCTCACTCCGTAGCTAAGTACGGCGCCTTGGCTAGGCAAAAGGCCTCCACAGGTAATTATATTACCACACTTGGAGAAGTCACACGTGGACACATGGGAGGAGCTAACACCATGTACGCGATAGACGCACCCCCTGAACTTTAA

>Sp13_P8_b7

ATGGAAAGATCAACTCTGATTAATTTACTTCAATTGCACCACTTCGAGCCAAAACTCAGTGTTGAAGGAATCATAGTTGTGCACGGAATTGCAGGCACTGGGAAAACCACTTTACTTAGGACTTTATTTTCTGCTTACCCTAGCTTAGTTATAGGTTCACCTAGGCCTTGCTATTTAGATAAACAAAACAAAATTTCACAAGTTTGCTTATCTTGCTTTCCCAATACCCATTGTGATATTGTCGATGAGTATCATTTGCTAGAAAGTTTTCTAGAACCAAAATTGGCTATCTTTGGTGACCCCTGTCAATGCACATACATTGAGAGACTTAGAGTCCCACATTACACTTCCTTCAGAACTCATAGATTTGGAAAGTCAACTGCTGAGATTTTGAACAAACTGTTTGACCTTAATATAGTCTCAGTTAAGAAAGAAGACGACATCGTTGAATTCTTTAACCCTTTTGAAGTTGACCCCACTGAGCATATCTCTGCCTCTGAAGAAGAAGTCTTGGACTTTGTTTCTGACCAAGTGGTGACCACTAGCTCAGAGGAACTAGCAGGACTTGAGTTTGCAGAAACAACTTTCTACTGCACAACATTGGCCGCAGCTGTTGCTGAAAATCCTGCTAAGACTTTCATCTCTCTGACTAGACACACCCACAAACTCACCATTGGGGAACTAAATGCCAGGTCTAACTCCTAGATGCCAGGTCTAACTCCTAGAGCTGACCTCACTGACACATACAAAATCATTGCCATTGCTTTCTTGTTGTCAGCTTGCATTTACTTCCAAAATAGCCACTACCAACCTGTTGCTGGAGACAACTTGCACCGTTTGCCTTTTGGTGGCCAATATCAAGACGGCACCAAAAAGATATCTTATTTTCCACAACAGCAGTCATACTTTCATTCTGGAAACAAATTAAATGTCCTCATACTTATCTTCATTCTCACGTTGGGTATTGTCCTCACCAATAAATTTAGTTTTAGCTTTAGTCGTACTACTCACCAGCATTCTTGCTATAACACACATTCAGCAACCAACAATACACAACCATTGTCAGGTCATCATTGAATGTCCTCATACTTATCTTCATTCTCACGTTGGGTATTGTCCTCACCAATAAATTTAGTTTTAGCTTTAGTCGTACTACTCACCAGCATTCTTGCTATAACACACATTCAGCAACCAACAATACACAACCATTGTCAGGTCATCATTGACGGTGCTGCAATAGTCATAACAAATTGTGAGAACACACCAGAAGTGCTTAAAGCAATAAACTTCTCCCCTTGGAACGGGTTAAGTTTTCCTAAATTTGAAAATTAAATGCCTGACACAACACCTGTTGCTGCCACTTCAAGTGCACCACCCACAGCCAAAGATGCTGGTGCCAAAGCTCCTTCTGACTTCTCAAATCCCAATACAGCTCCTAGTCTCAGTGATTTGAAGAAAGTCAAGTATGTCTCCACCGTGACCTCCGTGGCCACACCAGCTGAAATTGAAGCCCTAGGCAAAATCTTCACCGCTATGGGCCTTGCCGCCAATGAGACTGGTCCGGCCATGTGGGATCTAGCTCGTGCATATGCTGATGTGCAGAGTTCTAAATCGGCACAGCTGATTGGAGCTACCCCTTCCAACCCTGCACTATCACGCCGAGCCCTTGCTGCTCAGTTTGATCGAATCAATATAACCCCCAGGCAATTTTGCATGTACTTTGCCAAAGTTGTTTGGAACATACTTCTCGACAGCAACATTCCACCAGCAAATTGGGCCAAACTTGGTTACCAAGAAGATACAAAATTTGCTGCATTTGACTTCTTCGATGGAGTCACCAACCCTGCCAGCCTGCAGCCTGCTGATGGTCTTATCAGGCAGCCAAATGAGAAAGAACTAGCTGCTCACTCCGTAGCTAAGTACGGCGCCTTGGCTAGGCAAAAGATCTCCACAGGTAATTATATTACCACACTTGGAGAAGTCACACGTGGACACATGGGAGGAGCTAACACCATGTACGCGATAGACGCACCCCCTGAACTTTAA

>Sp13_P8_b6

ATGGAAAGATCAACTCTGATTAATTTACTTCAATTGCACCACTTCGAGCCAAAACTCAGTGTTGAAGGAATCATAGTTGTGCACGGAATTGCAGGCACTGGGAAAACCACTTTACTTAGGACTTTATTTTCTGCTTACCCTAGCTTAGTTATAGGTTCACCTAGGCCTTGCTATTTAGATAAACAAAACAAAATTTCACAAGTTTGCTTATCTTGCTTTCCCAATACCCATTGTGATATTGTCGATGAGTATCATTTGCTAGAAAGTTTTCTAGAACCAAAATTGGCTATCTTTGGTGACCCCTGTCAATGCACATACATTGAGAGACTTAGAGTCCCACATTACACTTCCTTCAGAACTCATAGATTTGGAAAGTCAACTGCTGAGATTTTGAACAAACTGTTTGACCTTAATATAGTCTCAGTTAAGAAAGAAGACGACATCGTTGAATTCTTTAACCCTTTTGAAGTTGACCCCACTGAGCATATCTCTGCCTCTGAAGAAGAAGTCTTGGACTTTGTTTCTGACCAAGTGGTGACCACTAGCTCAGAGGAACTAGCAGGACTTGAGTTTGCAGAAACAACTTTCTACTGCACAACATTGGCCGCAGCTGTTGCTGAAAATCCTGCTAAGACTTTCATCTCTCTGACTAGACACACCCACAAACTCACCATTGGGGAACTAAATGCCAGGTCTAACTCCTAGATGCCAGGTCTAACTCCTAGAGCTGACCTCACTGACACATACAAAATCATTGCCATTGCTTTCTTGTTGTCAGCTTGCATTTACTTCCAAAATAGCCACTACCAACCTGTTGCTGGAGACAACTTGCACCGTTTGCCTTTTGGTGGCCAATATCAAGACGGCACCAAAAAGATATCTTATTTTCCACAACAGCAGTCATACTTTCATTCTGGAAACAAATTAAATGTCCTCATACTTATCTTCATTCTCACGTTGGGTATTGTCCTCACCAATAAATTTAGTTTTAGCTTTAGTCGTACTACTCACCAGCATTCTTGCTATAACACACATTCAGCAACCAACAATACACAACCATTGTCAGGTCATCATTGAATGTCCTCATACTTATCTTCATTCTCACGTTGGGTATTGTCCTCACCAATAAATTTAGTTTTAGCTTTAGTCGTACTACTCACCAGCATTCTTGCTATAACACACATTCAGCAACCAACAATACACAACCATTGTCAGGTCATCATTGACGGTGCTGCAATAGTCATAACAAATTGTGAGAACACACCAGAAGTGCTTAAAGCAATAAACTTCTCCCCTTGGAACGGGTTAAGTTTTCCTAAATTTGAAAATTAAATGCCTGACACAACACCTGTTGCTGCCACTTCAAGTGCACCACCCACAGCCAAAGATGCTGGTGCCAAAGCTCCTTCTGACTTCTCAAATCCCAATACAGCTCCTAGTCTCAGCGATTTGAAGAAAGTCAAGTATGTCTCCACCGTGACCTCCGTGGCCACACCAGCTGAAATTGAAGCCCTAGGCAAAATCTTCACCGCTATGGGCCTTGCCGCCAATGAGACTGGTCCGGCCATGTGGGATCTAGCTCGTGCATATGCTGATGTGCAGAGTTCTAAATCGGCACAGCTGATTGGAGCTACCCCTTCCAACCCTGCACTATCACGCCGAGCCCTTGCTGCTCAGTTTGATCGAATCAATATAACCCCCAGGCAATTTTGCATGTACTTTGCCAAAGTTGTTTGGAACATACTTCTCGACAGCAACATTCCACCAGCAAATTGGGCCAAACTTGGTTACCAAGAAGATACAAAATTTGCTGCATTTGACTTCTTCGATGGAGTCACCAACCCTGCCAGCCTGCAGCCTGCTGATGGTCTTATCAGGCAGCCAAATGAGAAAGAACTAGCTGCTCACTCCGTAGCTAAGTACGGCGCCTTGGCTAGGCAAAAGATCTCCACAGGTAATTATATTACCACACTTGGAGAAGTCACACGTGGACACATGGGAGGAGCTAACACCATGTACGCGATAGACGCACCCCCTGAACTTTAA

>Sp13_P8_b5

ATGGAAAGATCAACTCTGATTAATTTACTTCAATTGCACCACTTCGAGCCAAAACTCAGTGTTGAAGGAATCATAGTTGTGCACGGAATTGCAGGCACTGGGAAAACCACTTTACTTAGGACTTTATTTTCTGCTTACCCTAGCTTAGTTATAGGTTCACCTAGGCCTTGCTATTTAGATAAACAAAACAAAATTTCACAAGTTTGCTTATCTTGCTTTCCCAATACCCATTGTGATATTGTCGATGAGTATCATTTGCTAGAAAGTTTTCTAGAACCAAAATTGGCTATCTTTGGTGACCCCTGTCAATGCACATACATTGAGAGACTTAGAGTCCCACATTACACTTCCTTCAGAACTCATAGATTTGGAAAGTCAACTGCTGAGATTTTGAACAAACTGTTTGACCTTAATATAGTCTCAGTTAAGAAAGAAGACGACATCGTTGAATTCTTTAACCCTTTTGAAGTTGACCCCACTGAGCATATCTCTGCCTCTGAAGAAGAAGTCTTGGACTTTGTTTCTGACCAAGTGGTGACCACTAGCTCAGAGGAACTAGCAGGACTTGAGTTTGCAGAAACAACTTTCTACTGCACAACATTGGCCGCAGCTGTTGCTGAAAATCCTGCTAAGACTTTCATCTCTCTGACTAGACACACCCACAAACTCACCATTGGGGAACTAAATGCCAGGTCTAACTCCTAGATGCCAGGTCTAACTCCTAGAGCTGACCTCACTGACACATACAAAATCATTGCCATTGCTTTCTTGTTGTCAGCTTGCATTTACTTCCAAAATAGCCACTACCAACCTGTTGCTGGAGACAACTTGCACCGTTTGCCTTTTGGTGGCCAATATCAAGACGGCACCAAAAAGATATCTTATTTTCCACAACAGCAGTCATACTTTCATTCTGGAAACAAATTAAATGTCCTCATACTTATCTTCATTCTCACGTTGGGTATTGTCCTCACCAATAAATTTAGTTTTAGCTTTAGTCGTACTACTCACCAGCATTCTTGCTATAACACACATTCAGCAACCAACAATACACAACCATTGTCAGGTCATCATTGAATGTCCTCATACTTATCTTCATTCTCACGTTGGGTATTGTCCTCACCAATAAATTTAGTTTTAGCTTTAGTCGTACTACTCACCAGCATTCTTGCTATAACACACATTCAGCAACCAACAATACACAACCATTGTCAGGTCATCATTGACGGTGCTGCAATAGTCATAACAAATTGTGAGAACACACCAGAAGTGCTTAAAGCAATAAACTTCTCCCCTTGGAACGGGTTAAGTTTTCCTAAATTTGAAAATTAAATGCCTGACACAACACCTGTTGCTGCCACTTCAAGTGCACCACCCACAGCCAAAGATGCTGGTGCCAAAGCTCCTTCTGACTTCTCAAATCCCAATACAGCTCCTAGTCTCAGTGATTTGAAGAAAGTCAAGTATGTCTCCACCGTGACCTCCGTGGCCACACCAGCTGAAATTGAAGCCCTAGGCAAAATCTTCACCGCTATGGGCCTTGCCGCCAATGAGACTGGTCCGGCCATGTGGGATCTAGCTCGTGCATATGCTGATGTGCAGAGTTCTAAATCGGCACAGCTGATTGGAGCTACCCCTTCCAACCCTGCACTATCACGCCGAGCCCTTGCTGCTCAGTTTGATCGAATCAATATAACCCCCAGGCAATTTTGCATGTACTTTGCCAAAGTTGTTTGGAACATACTTCTCGACAGCAACATTCCACCAGCAAATTGGGCCAAACTTGGTTACCAAGAAGATACAAAATTTGCTGCATTTGACTTCTTCGATGGAGTCACCAACCCTGCCAGCCTGCAGCCTGCTGATGGTCTTATCAGGCAGCCAAATGAGAAAGAACTAGCTGCTCACTCCGTAGCTAAGTACGGCGCCTTGGCTAGGCAAAAGATCTCCACAGGTAATTATATTACCACACTTGGAGAAGTCACACGTGGACACATGGGAGGAGCTAACACCATGTACGCGATAGACGCACCCCCTGAACCTTAA

>Sp13_P8_b4

ATGGAAAGATCAACTCTGATTAATTTACTTCAATTGCACCACTTCGAGCCAAAACTCAGTGTTGAAGGAATCATAGTTGTGCACGGAATTGCAGGCACTGGGAAAACCACTTTACTTAGGACTTTATTTTCTGCTTACCCTAGCTTAGTTATAGGTTCACCTAGGCCTTGCTATTTAGATAAACAAAACAAAATTTCACAAGTTTGCTTATCTTGCTTTCCCAATACCCATTGTGATATTGTCGATGAGTATCATTTGCTAGAAAGTTTTCTAGAACCAAAATTGGCTATCTTTGGTGACCCCTGTCAATGCACATACATTGAGAGACTTAGAGTCCCACATTACACTTCCTTCAGAACTCATAGATTTGGAAAGTCAACTGCTGAGATTTTGAACAAACTGTTTGACCTTAATATAGTCTCAGTTAAGAAAGAAGACGACATCGTTGAATTCTTTAACCCGTTTGAAGTTGACCCCACTGAGCATATCTCTGCCTCTGAAGAAGAAGTCTTGGACTTTGTTTCTGACCAAGTGGTGACCACTAGCTCAGAGGAACTAGCAGGACTTGAGTTTGCAGAAACAACTTTCTACTGCACAACATTGGCCGCAGCTGTTGCTGAAAATCCTGCTAAGACTTTCATCTCTCTGACTAGACACACCCACAAACTCACCATTGGGGAACTAAATGCCAGGTCTAACTCCTAGATGCCAGGTCTAACTCCTAGAGCTGACCTCACTGACACATACAAAATCATTGCCATTGCTTTCTTGTTGTCAGCTTGCATTTACTTCCAAAATAGCCACTACCAACCTGTTGCTGGAGACAACTTGCACCGTTTGCCTTTTGGTGGCCAATATCAAGACGGCACCAAAAAGATATCTTATTTTCCACAACAGCAGTCATACTTTCATTCTGGAAACAAATTAAATGTCCTCATACTTATCTTCATTCTCACGTTGGGTATTGTCCTCACCAATAAATTTAGTTTTAGCTTTAGTCGTACTACTCACCAGCATTCTTGCTATAACACACATTCAGCAACCAACAATACACAACCATTGTCAGGTCATCATTGAATGTCCTCATACTTATCTTCATTCTCACGTTGGGTATTGTCCTCACCAATAAATTTAGTTTTAGCTTTAGTCGTACTACTCACCAGCATTCTTGCTATAACACACATTCAGCAACCAACAATACACAACCATTGTCAGGTCATCATTGACGGTGCTGCAATAGTCATAACAAATTGTGAGAACACACCAGAAGTGCTTAAAGCAATAAACTTCTCCCCTTGGAACGGGTTAAGTTTTCCTAAATTTGAAAATTAAATGCCTGACACAACACCTGTTGCTGCCACTTCAAGTGCACCACCCACAGCCAAAGATGCTGGTGCCAAAGCTCCTTCTGACTTCTCAAATCCCAATACAGCTCCTAGTCTCAGTGATTTGAAGAAAGTCAAGTATGTCTCCACCGTGACCTCCGTGGCCACACCAGCTGAAATTGAAGCCCTAGGCAAAATCTTCACCGCTATGGGCCTTGCCGCCAATGAGACTGGTCCGGCCATGTGGGATCTAGCTCGTGCATATGCTGATGTGCAGAGTTCTAAATCGGCACAGCTGATTGGAGCTACCCCTTCCAACCCTGCACTATCACGCCGAGCCCTTGCTGCTCAGTTTGATCGAATCAATATAACCCCCAGGCAATTTTGCATGTACTTTGCCAAAGTTGTTTGGAACATACTTCTCGACAGCAACATTCCACCAGCAAATTGGGCCAAACTTGGTTACCAAGAAGATACAAAATTTGCTGCATTTGACTTCTTCGATGGAGTCACCAACCCTGCCAGCCTGCAGCCTGCTGATGGTCTTATCAGGCAGCCAAATGAGAAAGAACTAGCTGCTCACTCCGTAGCTAAGTACGGCGCCTTGGCTAGGCAAAAGATCTCCACAGGTAATTATATTACCACACTTGGAGAAGTCACACGTGGACACATGGGAGGAGCTAACACCATGTACGCGATAGACGCACCCCCTGAACTTTAA

>Sp13_P8_b3

ATGGAAAGATCAACTCTGATTAATTTACTTCAATTGCACCACTTCGAGCCAAAACTCAGTGTTGAAGGAATCATAGTTGTGCACGGAATTGCAGGCACTGGAAAAACCACTTTACTTAGGACTTTATTTTCTGCTTACCCTAGCTTAGTTATAGGTTCACCTAGGCCTTGCTATTTAGATAAACAAAACAAAATTTCACAAGTTTGCTTATCTTGCTTTCCCAATACCCATTGTGATATTGTCGATGAGTATCATTTGCTAGAAAGTTTTCTAGAACCAAAATTGGCTATCTTTGGTGACCCCTGTCAATGCACATACATTGAGAGACTTAGAGTCCCACATTACACTTCCTTCAGAACTCATAGATTTGGAAAGTCAACTGCTGAGATTTTGAACAAACTGTTTGACCTTAATATAGTCTCAGTTAAGAAAGAAGACGACATCGTTGAATTCTTTAACCCTTTTGAAGTTGACCCCACTGAGCATATCTCTGCCTCTGAAGAAGAAGTCTTGGACTTTGTTTCTGACCAAGTGGTGACCACTAGCTCAGAGGAACTAGCAGGACTTGAGTTTGCAGAAACAACTTTCTACTGCACAACATTGGCCGCAGCTGTTGCTGAAAATCCTGCTAAGACTTTCATCTCTCTGACTAGACACACCCACAAACTCACCATTGGGGAACTAAATGCCAGGTCTAACTCCTAGATGCCAGGTCTAACTCCTAGAGCTGACCTCACTGACACATACAAAATCATTGCCATTGCTTTCTTGTTGTCAGCTTGCATTTACTTCCAAAATAGCCACTACCAACCTGTTGCTGGAGACAACTTGCACCGTTTGCCTTTTGGTGGCCAATATCAAGACGGCACCAAAAAGATATCTTATTTTCCACAACAGCAGTCATACTTTCATTCTGGAAACAAATTAAATGTCCTCATACTTATCTTCATTCTCACGTTGGGTATTGTCCTCACCAATAAATTTAGTTTTAGCTTTAGTCGTACTACTCACCAGCATTCTTGCTATAACACACATTCAGCAACCAACAATACACAACCATTGTCAGGTCATCATTGAATGTCCTCATACTTATCTTCATTCTCACGTTGGGTATTGTCCTCACCAATAAATTTAGTTTTAGCTTTAGTCGTACTACTCACCAGCATTCTTGCTATAACACACATTCAGCAACCAACAATACACAACCATTGTCAGGTCATCATTGACGGTGCTGCAATAGTCATAACAAATTGTGAGAACACACCAGAAGTGCTTAAAGCAATAAACTTCTCCCCTTGGAACGGGTTAAGTTTTCCTAAATTTGAAAATTAAATGCCTGACACAACACCTGTTGCTGCCACTTCAAGTGCACCACCCACAGCCAAAGATGCTGGTGCCAAAGCTCCTTCTGACTTCTCAAATCCCAATACAGCTCCTAGTCTCAGTGATTTGAAGAAAGTCAAGTATGTCTCCACCGTGACCTCCGTGGCCACACCAGCTGAAATTGAAGCCCTAGGCAAAATCTTCACCGCTATGGGCCTTGCCGCCAATGAGACTGGTCCGGCCATGTGGGATCTAGCTCGTGCATATGCTGATGTGCAGAGTTCTAAATCGGCACAGCTGATTGGAGCTACCCCTTCCAACCCTGCACTATCACGCCGAGCCCTTGCTGCTCAGTTTGATCGAATCAATATAACCCCCAGGCAATTTTGCATGTACTTTGCCAAAGTTGTTTGGAACATACTTCTCGACAGCAACATTCCACCAGCAAATTGGGCCAAACTTGGTTACCAAGAAGATACAAAATTTGCTGCATTTGACTTCTTCGATGGAGTCACCAACCCTGCCAGCCTGCAGCCTGCTGATGGTCTTATCAGGCAGCCAAATGAGAAAGAACTAGCTGCTCACTCCGTAGCTAAGTACGGCGCCTTGGCTAGGCAAAAGATCTCCACAGGTAATTATATTACCACACTTGGAGAAGTCACACGTGGACACATGGGAGGAGCTAACACCATGTACGCGATAGACGCACCCCCTGAACTTTAA

>Sp13_P8_b2

ATGGAAAGATCAACTCTGATTAATTTACTTCAATTGCACCACTTCGAGCCAAAACTCAGTGTTGAAGGAATCATAGTTGTGCACGGAATTGCAGGCACTGGGAAAACCACTTTACTTAGGACTTTATTTTCTGCTTACCCTAGCTTAGTTATAGGTTCACCTAGGCCTTGCTATTTAGATAAACAAAACAAAATTTCACAAGTTTGCTTATCTTGCTTTCCCAATACCCATTGTGATATTGTCGATGAGTATCATTTGCTAGAAAGTTTTCTAGAACCAAAATTGACTATCTTTGGTGACCCCTGTCAATGCACATACATTGAGAGACTTAGAGTCCCACATTACACTTCCTTCAGAACTCATAGATTTGGAAAGTCAACTGCTGAGATTTTGAACAAACTGTTTGACCTTAATATAGTCTCAGTTAAGAAAGAAGACGACATCGTTGAATTCTTTAACCCGTTTGAAGTTGACCCCACTGAGCATATCTCTGCCTCTGAAGAAGAAGTCTTGGACTTTGTTTCTGACCAAGTGGTGACCACTAGCTCAGAGGAACTAGCAGGACTTGAGTTTGCAGAAACAACTTTCTACTGCACAACATTGGCCGCAGCTGTTGCTGAAAATCCTGCTAAGACTTTCATCTCTCTGACTAGACACACCCACAAACTCACCATTGGGGAACTAAATGCCAGGTCTAACTCCTAGATGCCAGGTCTAACTCCTAGAGCTGACCTCACTGACACATACAAAATCATTGCCATTGCTTTCTTGTTGTCAGCTTGCATTTACTTCCAAAATAGCCACTACCAACCTGTTGCTGGAGACAACTTGCACCGTTTGCCTTTTGGTGGCCAATATCAAGACGGCACCAAAAAGATATCTTATTTTCCACAACAGCAGTCATACTTTCATTCTGGAAACAAATTAAATGTCCTCATACTTATCTTCATTCTCACGTTGGGTATTGTCCTCACCAATAAATTTAGTTTTAGCTTTAGTCGTACTACTCACCAGCATTCTTGCTATAACACACATTCAGCAACCAACAATACACAACCATTGTCAGGTCATCATTGAATGTCCTCATACTTATCTTCATTCTCACGTTGGGTATTGTCCTCACCAATAAATTTAGTTTTAGCTTTAGTCGTACTACTCACCAGCATTCTTGCTATAACACACATTCAGCAACCAACAATACACAACCATTGTCAGGTCATCATTGACGGTGCTGCAATAGTCATAACAAATTGTGAGAACACACCAGAAGTGCTTAAAGCAATAAACTTCTCCCCTTGGAACGGGTTAAGTTTTCCTAAATTTGAAAATTAAATGCCTGACACAACACCTGTTGCTGCCACTTCAAGTGCACCACCCACAGCCAAAGATGCTGGTGCCAAAGCTCCTTCTGACTTCTCAAATCCCAATACAGCTCCTAGTCTCAGTGATTTGAAGAAAGTCAAGTATGTCTCCACCGTGACCTCCGTGGCCACACCAGCTGAAATTGAAGCCCTAGGCAAAATCTTCACCGCTATGGGCCTTGCCGCCAATGAGACTGGTCCGGCCATGTGGGATCTAGCTCGTGCATATGCTGATGTGCAGAGTTCTAAATCGGCACAGCTGATTGGAGCTACCCCTTCCAACCCTGCACTATCACGCCGAGCCCTTGCTGCTCAGTTTGATCGAATCAATATAACCCCCAGGCAATTTTGCATGTACTTTGCCAAAGTTGTTTGGAACATACTTCTCGACAGCAACATTCCACCAGCAAATTGGGCCAAACTTGGTTACCAAGAAGATACAAAATTTGCTGCATTTGACTTCTTCGATGGAGTCACCAACCCTGCCAGCCTGCAGCCTGCTGATGGTCTTATCAGGCAGCCAAATGAGAAAGAACTAGCTGCTCACTCCGTAGCTAAGTACGGCGCCTTGGCTAGGCAAAAGATCTCCACAGGTAATTATATTACCACACTTGGAGAAGTCACACGTGGACACATGGGAGGAGCTAACACCATGTACGCGATAGACGCACCCCCTGAACTTTAA

>Sp13_P8_b1

ATGGAAAGATCAACTCTGATTAATTTACTTCAATTGCACCACTTCGAGCCAAAACTCAGTGTTGAAGGAATCATAGTTGTGCACGGAATTGCAGGCACTGGGAAAACCACTTTACTTAGGACTTTATTTTCTGCTTACCCTAGCTTAGTTATAGGTTCACCTAGGCCTTGCTATTTAGATAAACAAAACAAAATTTCACAAGTTTGCTTATCTTGCTTTCCCAATACCCATTGTGATATTGTCGATGAGTATCATTTGCTAGAAAGTTTTCTAGAACCAAAATTGGCTATCTTTGGTGACCCCTGTCAATGCACATACATTGAGAGACTTAGAGTCCCACATTACACTTCCTTCAGAACTCATAGATTTGGAAAGTCAACTGCTGAGATTTTGAACAAACTGTTTGACCTTAATATAGTCTCAGTTAAGAAAGAAGACGACATCGTTGAATTCTTTAACCCGTTTGAAGTTGACCCCACTGAGCATATCTCTGCCTCTGAAGAAGAAGTCTTGGACTTTGTTTCTGACCAAGTGGTGACCACTAGCTCAGAGGAACTAGCAGGACTTGAGTTTGCAGAAACAACTTTCTACTGCACAACATTGGCCGCAGCTGTTGCTGAAAATCCTGCTAAGACTTTCATCTCTCTGACTAGACACACCCACAAACTCACCATTGGGGAACTAAATGCCAGGTCTAACTCCTAGATGCCAGGTCTAACTCCTAGAGCTGACCTCACTGACACATACAAAATCATTGCCATTGCTTTCTTGTTGTCAGCTTGCATTTACTTCCAAAATAGCCACTACCAACCTGTTGCTGGAGACAACTTGCACCGTTTGCCTTTTGGTGGCCAATATCAAGACGGCACCAAAAAGATATCTTATTTTCCACAACAGCAGTCATACTTTCATTCTGGAAACAAATTAAATGTCCTCATACTTATCTTCATTCTCACGTTGGGTATTGTCCTCACCAATAAATTTAGTTTTAGCTTTAGTCGTACTACTCACCAGCATTCTTGCTATAACACACATTCAGCAACCAACAATACACAACCATTGTCAGGTCATCATTGAATGTCCTCATACTTATCTTCATTCTCACGTTGGGTATTGTCCTCACCAATAAATTTAGTTTTAGCTTTAGTCGTACTACTCACCAGCATTCTTGCTATAACACACATTCAGCAACCAACAATACACAACCATTGTCAGGTCATCATTGACGGTGCTGCAATAGTCATAACAAATTGTGAGAACACACCAGAAGTGCTTAAAGCAATAAACTTCTCCCCTTGGAACGGGTTAAGTTTTCCTAAATTTGAAAATTAAATGCCTGACACAACACCTGTTGCTGCCACTTCAAGTGCACCACCCACAGCCAAAGATGCTGGTGCCAAAGCTCCTTCTGACTTCTCAAATCCCAATACAGCTCCTAGTCTCAGTGATTTGAAGAAAGTCAAGTATGTCTCCACCGTGACCTCCGTGGCCACACCAGCTGAAATTGAAGCCCTAGGCAAAATCTTCACCGCTATGGGCCTTGCCGCCAATGAGACTGGTCCGGCCATGTGGGATCTAGCTCGTGCATATGCTGATGTGCAGAGTTCTAAATCGGCACAGCTGATTGGAGCTACCCCTTCCAACCCTGCACTATCACGCCGAGCCCTTGCTGCTCAGTTTGATCGAATCAATATAACCCCCAGGCAATTTTGCATGTACTTTGCCAAAGTTGTTTGGAACATACTTCTCGACAGCAACATTCCACCAGCAAATTGGGCCAAACTTGGTTACCAAGAAGATACAAAATTTGCTGCATTTGACTTCTTCGATGGAGTCACCAACCCTGCCAGCCTGCAGCCTGCTGATGGTCTTATCAGGCAGCCAAATGAGAAAGAACTAGCTGCTCACTCCGTAGCTAAGTACGGCGCCTTGGCTAGGCAAAAGATCTCCACAGGTAATTATATTACCACACTTGGAGAAGTCACACGTGGACACATGGGAGGAGCTAACACCATGTACGCGATAGACGCACCCCCTGAACTTTAA

>Sp13_P8_a14

ATGGAAAGATCAACTCTGATTAATTTACTTCAATTGCACCACTTCGAGCCAAAACTCAGTGTTGAAGGAATCATAGTTGTGCACGGAATTGCAGGCACTGGGAAAACCACTTTACTTAGGACTTTATTTTCTGCTTACCCTAGCTTAGTTATAGGTTCACCTAGGCCTTGCTATTTAGATAAACAAAACAAAATTTCACAAGTTTGCTTATCTTGCTTTCCCAATACCCATTGTGATATTGTCGATGAGTATCATTTGCTAGAAAGTTTTCTAGAACCAAAATTGGCTATCTTTGGTGACCCCTGTCAATGCACATACATTGAGAGACTTAGAGTCCCACATTACACTTCCTTCAGAACTCATAGATTTGGAAAGTCAACTGCTGAGATTTTGAACAAACTGTTTGACCTTAATATAGTCTCAGTTAAGAAAGAAGACGACATCGTTGAATTCTTTAACCCTTTTGAAGTTGACCCCACTGAGCATATCTCTGCCTCTGAAGAAGAAGTCTTGGACTTTGTTTCTGACCAAGTGGTGACCACTAGCTCAGAGGAACTAGCAGGACTTGAGTTTGCAGAAACAACTTTCTACTGCACAACATTGGCCGCAGCTGTTGCTGAAAATCCTGCTAAGACTTTCATCTCTCTGACTAGACACACCCACAAACTCACCATTGGGGAACTAAATGCCAGGTCTAACTCCTAGATGCCAGGTCTAACTCCTAGAGCTGACCTCACTGACACATACAAAATCATTGCCATTGCTTTCTTGTTGTCAGCTTGCATTTACTTCCAAAATAGCCACTACCAACCTGTTGCTGGAGACAACTTGCACCGTTTGCCTTTTGGTGGCCAATATCAAGACGGCACCAAAAAGATATCTTATTTTCCACAACAGCAGTCATACTTTCATTCTGGAAACAAATTAAATGTCCTCATACTTATCTTCATTCTCACGTTGGGTATTGTCCTCACCAATAAATTTAGTTTTAGCTTTAGTCGTACTACTCACCAGCATTCTTGCTATAACACACATTCAGCAACCAACAATACACAACCATTGTCAGGTCATCATTGAATGTCCTCATACTTATCTTCATTCTCACGTTGGGTATTGTCCTCACCAATAAATTTAGTTTTAGCTTTAGTCGTACTACTCACCAGCATTCTTGCTATAACACACATTCAGCAACCAACAATACACAACCATTGTCAGGTCATCATTGACGGTGCTGCAATAGTCATAACAAATTGTGAGAACACACCAGAAGTGCTTAAAGCAATAAACTTCTCCCCTTGGAACGGGTTAAGTTTTCCTAAATTTGAAAATTAAATGCCTGACACAACACCTGTTGCTGCCACTTCAAGTGCACCACCCACAGCCAAAGATGCTGGTGCCAAAGCTCCTTCTGACTTCTCAAATCCCAATACAGCTCCTAGTCTCAGTGATTTGAAGAAAGTCAAGTATGTCTCCACCGTGACCTCCGTGGCCACACCAGCTGAAATTGAAGCCCTAGGCAAAATCTTCACCGCTATGGGCCTTGCCGCCAATGAGACTGGTCCGGCCATGTGGGATCTAGCTCGTGCATATGCTGATGTGCAGAGTTCTAAATCGACACAGCTGATTGGAGCTACCCCTTCCAACCCTGCACTATCACGCCGAGCCCTTGCTGCTCAGTTTGATCGAATCAATATAACCCCCAGGCAATTTTGCATGTACTTTGCCAAAGTTGTTTGGAACATACTTCTCGACAGCAACATTCCACCAGCAAATTGGGCCAAACTTGGTTACCAAGAAGATACAAAATTTGCTGCATTTGACTTCTTCGATGGAGTCACCAACCCTGCCAGCCTGCAGCCTGCTGATGGTCTTATCAGGCAGCCAAATGAGAAAGAACTAGCTGCTCACTCCGTAGCTAAGTACGGCGCCTTGGCTAGGCAAAAGATCTCCACAGGTAATTATATTACCACACTTGGAGAAGTCACACGTGGACACATGGGAGGAGCTAACACCATGTACGCGATAGACGCACCCCCTGAACTTTAA

>Sp13_P8_a13

ATGGAAAGATCAACTCTGATTAATTTACTTCAATTGCACCACTTCGAGCCAAAACTCAGTGTTGAAGGAATCATAGTTGTGCACGGAATTGCAGGCACTGGGAAAACCACTTTACTTAGGACTTTATTTTCTGCTTACCCTAGCTTAGTTATAGGTTCACCTAGGCCTTGCTATTTAGATAAACAAAACAAAATTTCACAAGTTTGCTTATCTTGCTTTCCCAATACCCATTGTGATATTGTCGATGAGTATCATTTGCTAGAAAGCTTTCTAGAACCAAAATTGGCTATCTTTGGTGACCCCTGTCAATGCACATACATTGAGAGACTTAGAGTCCCACATTACACTTCCTTCAGAACTCATAGATTTGGAAAGTCAACTGCTGAGATTTTGAACAAACTGTTTGACCTTAATATAGTCTCAGTTAAGAAAGAAGACGACATCGTTGAATTCTTTAACCCTTTTGAAGTTGACCCCACTGAGCATATCTCTGCCTCTGAAGAAGAAGTCTTGGACTTTGTTTCTGACCAAGTGGTGACCACTAGCTCAGAGGAACTAGCAGGACTTGAGTTTGCAGAAACAACTTTCTACTGCACAACATTGGCCGCAGCTGTTGCTGAAAATCCTGCTAAGACTTTCATCTCTCTGACTAGACACACCCACAAACTCACCATTGGGGAACTAAATGCCAGGTCTAACTCCTAGATGCCAGGTCTAACTCCTAGAGCTGACCTCACTGACACATACAAAATCATTGCCATTGCTTTCTTGTTGTCAGCTTGCATTTACTTCCAAAATAGCCACTACCAACCTGTTGCTGGAGACAACTTGCACCGTTTGCCTTTTGGTGGCCAATATCAAGACGGCACCAAAAAGATATCTTATTTTCCACAACAGCAGTCATACTTTCATTCTGGAAACAAATTAAATGTCCTCATACTTATCTTCATTCTCACGTTGGGTATTGTCCTCACCAATAAATTTAGTTTTAGCTTTAGTCGTACTACTCACCAGCATTCTTGCTATAACACACATTCAGCAACCAACAATACACAACCATTGTCAGGTCATCATTGAATGTCCTCATACTTATCTTCATTCTCACGTTGGGTATTGTCCTCACCAATAAATTTAGTTTTAGCTTTAGTCGTACTACTCACCAGCATTCTTGCTATAACACACATTCAGCAACCAACAATACACAACCATTGTCAGGTCATCATTGACGGTGCTGCAATAGTCATAACAAATTGTGAGAACACACCAGAAGTGCTTAAAGCAATAAACTTCTCCCCTTGGAACGGGTTAAGTTTTCCTAAATTTGAAAATTAAATGCCTGACACAACACCTGTTGCTGCCACTTCAAGTGCACCACCCACAGCCAAAGATGCTGGTGCCAAAGCTCCTTCTGACTTCTCAAATCCCAATACAGCTCCTAGTCTCAGTGATTTGAAGAAAGTCAAGTATGTCTCCACCGTGACCTCCGTGGCCACACCAGCTGAAATTGAAGCCCTAGGCAAAATCTTCACCGCTATGGGCCTTGCCGCCAATGAGACTGGTCCGGCCATGTGGGATCTAGCTCGTGCATATGCTGATGTGCAGAGTTCTAAATCGGCACAGCTGATTGGAGCTACCCCTTCCAACCCTGCACTATCACGCCGAGCCCTTGCTGCTCAGTTTGATCGAATCAATATAACCCCCAGGCAATTTTGCATGTACTTTGCCAAAGTTGTTTGGAACATACTTCTCGACAGCAACATTCCACCAGCAAATTGGGCCAAACTTGGTTACCAAGAAGATACAAAATTTGCTGCATTTGACTTCTTCGATGGAGTCACCAACCCTGCCAGCCTGCAGCCTGCTGATGGTCTTATCAGGCAGCCAAATGAGAAAGAACTAGCTGCTCACTCCGTAGCTAAGTACGGCGCCTTGGCTAGGCAAAAGATCTCCACAGGTAATTATATTACCACACTTGGAGAAGTCACACGTGGACACATGGGAGGAGCTAACACCATGTACGCGATAGACGCACCCCCTGAACTTTAA

>Sp13_P8_a12

ATGGAAAGATCAACTCTGATTAATTTACTTCAATTGCACCACTTCGAGCCAAAACTCAGTGTTGAAGGAATCATAGTTGTGCACGGAATTGCAGGCACTGGGAAAACCACTTTACTTAGGACTTTATTTTCTGCTTACCCTAGCTTAGTTATAGGTTCACCTAGGCCTTGCTATTTAGATAAACAAAACAAAATTTCACAAGTTTGCTTATCTTGCTTTCCCAATACCCATTGTGATATTGTCGATGAGTATCATTTGCTAGAAAGCTTTCTAGAACCAAAATTGGCTATCTTTGGTGACCCCTGTCAATGCACATACATTGAGAGACTTAGAGTCCCACATTACACTTCCTTCAGAACTCATAGATTTGGAAAGTCAACTGCTGAGATTTTGAACAAACTGTTTGACCTTAATATAGTCTCAGTTAAGAAAGAAGACGACATCGTTGAATTCTTTAACCCTTTTGAAGTTGACCCCACTGAGCATATCTCTGCCTCTGAAGAAGAAGTCTTGGACTTTGTTTCTGACCAAGTGGTGACCACTAGCTCAGAGGAACTAGCAGGACTTGAGTTTGCAGAAACAACTTTCTACTGCACAACATTGGCCGCAGCTGTTGCTGAAAATCCTGCTAAGACTTTCATCTCTCTGACTAGACACACCCACAAACTCACCATTGGGGAACTAAATGCCAGGTCTAACTCCTAGATGCCAGGTCTAACTCCTAGAGCTGACCTCACTGACACATACAAAATCATTGCCATTGCTTTCTTGTTGTCAGCTTGCATTTACTTCCAAAATAGCCACTACCAACCTGTTGCTGGAGACAACTTGCACCGTTTGCCTTTTGGTGGCCAATATCAAGACGGCACCAAAAAGATATCTTATTTTCCACAACAGCAGTCATACTTTCATTCTGGAAACAAATTAAATGTCCTCATACTTATCTTCATTCTCACGTTGGGTATTGTCCTCACCAATAAATTTAGTTTTAGCTTTAGTCGTACTACTCACCAGCATTCTTGCTATAACACACATTCAGCAACCAACAATACACAACCATTGTCAGGTCATCATTGAATGTCCTCATACTTATCTTCATTCTCACGTTGGGTATTGTCCTCACCAATAAATTTAGTTTTAGCTTTAGTCGTACTACTCACCAGCATTCTTGCTATAACACACATTCAGCAACCAACAATACACAACCATTGTCAGGTCATCATTGACGGTGCTGCAATAGTCATAACAAATTGTGAGAACACACCAGAAGTGCTTAAAGCAATAAACTTCTCCCCTTGGAACGGGTTAAGTTTTCCTAAATTTGAAAATTAAATGCCTGACACAACACCTGTTGCTGCCACTTCAAGTGCACCACCCACAGCCAAAGATGCTGGTGCCAAAGCTCCTTCTGACTTCTCAAATCCCAATACAGCTCCTAGTCTCAGTGATTTGAAGAAAGTCAAGTATGTCTCCACCGTGACCTCCGTGGCCACACCAGCTGAAATTGAAGCCCTAGGCAAAATCTTCACCGCTATGGGCCTTGCCGCCAATGAGACTGGTCCGGCCATGTGGGATCTAGCTCGTGCATATGCTGATGTGCAGAGTTCTAAATCGGCACAGCTGATTGGAGCTACCCCTTCCAACCCTGCACTATCACGCCGAGCCCTTGCTGCTCAGTTTGATCGAATCAATATAACCCCCAGGCAATTTTGCATGTATTTTGCCAAAGTTGTTTGGAACATACTTCTCGACAGCAACATTCCACCAGCAAATTGGGCCAAACTTGGTTACCAAGAAGATACAAAATTTGCTGCATTTGACTTCTTCGATGGAGTCACCAACCCTGCCAGCCTGCAGCCTGCTGATGGTCTTATCAGGCAGCCAAATGAGAAAGAACTAGCTGCTCACTCCGTAGCTAAGTACGGCGCCTTGGCTAGGCAAAAGATCTCCACAGGTAATTATATTACCACACTTGGAGAAGTCACACGTGGACACATGGGAGGAGCTAACACCATGTACGCGATAGACGCACCCCCTGAACTTTAA

>Sp13_P8_a11

ATGGAAAGATCAACTCTGATTAATTTACTTCAATTGCACCACTTCGAGCCAAAACTCAGTGTTGAAGGAATCATAGTTGTGCACGGAATTGCAGGCACTGGGAAAACCACTTTACTTAGGACTTTATTTTCTGCTTACCCTAGCTTAGTTATAGGTTCACCTAGGCCTTGCTATTTAGATAAACAAAACAAAATTTCACAAGTTTGCTTATCTTGCTTTCCCAATACCCATTGTGATATTGTCGATGAGTATCATTTGCTAGAAAGCTTTCTAGAACCAAAATTGGCTATCTTTGGTGACCCCTGTCAATGCACATACATTGAGAGACTTAGAGTCCCACATTACACTTCCTTCAGAACTCATAGATTTGGAAAGTCAACTGCTGAGATTTTGAACAAACTGTTTGACCTTAATATAGTCTCAGTTAAGAAAGAAGACGACATCGTTGAATTCTTTAACCCTTTTGAAGTTGACCCCACTGAGCATATCTCTGCCTCTGAAGAAGAAGTCTTGGACTTTGTTTCTGACCAAGTGGTGACCACTAGCTCAGAGGAACTAGCAGGACTTGAGTTTGCAGAAACAACTTTCTACTGCACAACATTGGCCGCAGCTGTTGCTGAAAATCCTGCTAAGACTTTCATCTCTCTGACTAGACACACCCACAAACTCACCATTGGGGAACTAAATGCCAGGTCTAACTCCTAGATGCCAGGTCTAACTCCTAGAGCTGACCTCACTGACACATACAAAATCATTGCCATTGCTTTCTTGTTGTCAGCTTGCATTTACTTCCAAAATAGCCACTACCAACCTGTTGCTGGAGACAACTTGCACCGTTTGCCTTTTGGTGGCCAATATCAAGACGGCACCAAAAAGATATCTTATTTTTCACAACAGCAGTCATACTTTCATTCTGGAAACAAATTAAATGTCCTCATACTTATCTTCATTCTCACGTTGGGTATTGTCCTCACCAATAAATTTAGTTTTAGCTTTAGTCGTACTACTCACCAGCATTCTTGCTATAACACACATTCAGCAACCAACAATACACAACCATTGTCAGGTCATCATTGAATGTCCTCATACTTATCTTCATTCTCACGTTGGGTATTGTCCTCACCAATAAATTTAGTTTTAGCTTTAGTCGTACTACTCACCAGCATTCTTGCTATAACACACATTCAGCAACCAACAATACACAACCATTGTCAGGTCATCATTGACGGTGCTGCAATAGTCATAACAAATTGTGAGAACACACCAGAAGTGCTTAAAGCAATAAACTTCTCCCCTTGGAACGGGTTAAGTTTTCCTAAATTTGAAAATTAAATGCCTGACACAACACCTGTTGCTGCCACTTCAAGTGCACCACCCACAGCCAAAGATGCTGGTGCCAAAGCTCCTTCTGACTTCTCAAATCCCAATACAGCTCCTAGTCTCAGTGATTTGAAGAAAGTCAAGTATGTCTCCACCGTGACCTCCGTGGCCACACCAGCTGAAATTGAAGCCCTAGGCAAAATCTTCACCGCTATGGGCCTTGCCGCCAATGAGACTGGTCCGGCCATGTGGGATCTAGCTCGTGCATATGCTGATGTGCAGAGTTCTAAATCGGCACAGCTGATTGGAGCTACCCCTTCCAACCCTGCACTATCACGCCGAGCCCTTGCTGCTCAGTTTGATCGAATCAATATAACCCCCAGGCAATTTTGCATGTACTTTGCCAAAGTTGTTTGGAACATACTTCTCGACAGCAACATTCCACCAGCAAATTGGGCCAAACTTGGTTACCAAGAAGATACAAAATTTGCTGCATTTGACTTCTTCGATGGAGTCACCAACCCTGCCAGCCTGCAGCCTGCTGATGGTCTTATCAGGCAGCCAAATGAGAAAGAACTAGCTGCTCACTCCGTAGCTAAGTACGGCGCCTTGGCTAGGCAAAAGATCTCCACAGGTAATTATATTACCACACTTGGAGAAGTCACACGTGGACACATGGGAGGAGCTAACACCATGTACGCGATAGACGCACCCCCTGAACTTTAA

>Sp13_P8_a10

ATGGAAAGATCAACTCTGATTAATTTACTTCAATTGCACCACTTCGAGCCAAAACTCAGTGTTGAAGGAATCATAGTTGTGCACGGAATTGCAGGCACTGGGAAAACCACTTTACTTAGGACTTTATTTTCTGCTTACCCTAGCTTAGTTATAGGTTCACCTAGGCCTTGCTATTTAGATAAACAAAACAAAATTTCACAAGTTTGCTTATCTTGCTTTCCCAATACCCATTGTGATATTGTCGATGAGTATCATTTGCTAGAAAGTTTTCTAGAACCAAAATTGGCTATCTTTGGTGACCCCTGTCAATGCACATACATTGAGAGACTTAGAGTCCCACATTACACTTCCTTCAGAACTCATAGATTTGGAAAGTCAACTGCTGAGATTTTGAACAAACTGTTTGACCTTAATATAGTCTCAGTTAAGAAAGAAGACGACATCGTTGAATTCTTTAACCCTTTTGAAGTTGACCCCACTGAGCATATCTCTGCCTCTGAAGAAGAAGTCTTGGACTTTGTTTCTGACCAAGTGGTGACCACTAGCTCAGAGGAACTAGCAGGACTTGAGTTTGCAGAAACAACTTTCTACTGCACAACATTGGCCGCAGCTGTTGCTGAAAATCCTGCTAAGACTTTCATCTCTCTGACTAGACACACCCACAAACTCACCATTGGGGAACTAAATGCCAGGTCTAACTCCTAGATGCCAGGTCTAACTCCTAGAGCTGACCTCACTGACACATACAAAATCATTGCCATTGCTTTCTTGTTGTCAGCTTGCATTTACTTCCAAAATAGCCACTACCAACCTGTTGCTGGAGACAACTTGCACCGTTTGCCTTTTGGTGGCCAATATCAAGACGGCACCAAAAAGATATCTTATTTTCCACAACAGCAGTCATACTTTCATTCTGGAAACAAATTAAATGTCCTCATACTTATCTTCATTCTCACGTTGGGTATTGTCCTCACCAATAAATTTAGTTTTAGCTTTAGTCGTACTACTCACCAGCATTCTTGCTATAACACACATTCAGCAACCAACAATACACAACCATTGTCAGGTCATCATTGAATGTCCTCATACTTATCTTCATTCTCACGTTGGGTATTGTCCTCACCAATAAATTTAGTTTTAGCTTTAGTCGTACTACTCACCAGCATTCTTGCTATAACACACATTCAGCAACCAACAATACACAACCATTGTCAGGTCATCATTGACGGTGCTGCAATAGTCATAACAAATTGTGAGAACACACCAGAAGTGCTTAAAGCAATAAACTTCTCCCCTTGGAACGGGTTAAGTTTTCCTAAATTTGAAAATTAAATGCCTGACACAACACCTGTTGCTGCCACTTCAAGTGCACCACCCACAGCCAAAGATGCTGGTGCCAAAGCTCCTTCTGACTTCTCAAATCCCAATACAGCTCCTAGTCTCAGTGATTTGAAGAAAGTCAAGTATGTCTCCACCGTGACCTCCGTGGCCACACCAGCTGAAATTGAAGCCCTAGGCAAAATCTTCACCGCTATGGGCCTTGCCGCCAATGAGACTGGTCCGGCCATGTGGGATCTAGCTCGTGCATATGCTGATGTGCAGAGTTCTAAATCGGCACAGCTGATTGGAGCTACCCCTTCCAACCCTGCACTATCACGCCGAGCCCTTGCTGCTCAGTTTGATCGAATCAATATAACCCCCAGGCAATTTTGCATGTATTTTGCCAAAGTTGTTTGGAACATACTTCTCGACAGCAACATTCCACCAGCAAATTGGGCCAAACTTGGTTACCAAGAAGATACAAAATTTGCTGCATTTGACTTCTTCGATGGAGTCACCAACCCTGCCAGCCTGCAGCCTGCTGATGGTCTTATCAGGCAGCCAAATGAGAAAGAACTAGCTGCTCACTCCGTAGCTAAGTACGGCGCCTTGGCTAGGCAAAAGATCTCCACAGGTAATTATATTACCACACTTGGAGAAGTCACACGTGGACACATGGGAGGAGCTAACACCATGTACGCGATAGACGCACCCCCTGAACTTTAA

>Sp13_P8_a9

ATGGAAAGATCAACTCTGATTAATTTACTTCAATTGCACCACTTCGAGCCAAAACTCAGTGTTGAAGGAATCATAGTTGTGCACGGAATTGCAGGCACTGGGAAAACCACTTTACTTAGGACTTTATTTTCTGCTTACCCTAGCTTAGTTATAGGTTCACCTAGGCCTTGCTATTTAGATAAACAAAACAAAATTTCACAAGTTTGCTTATCTTGCTTTCCCAATACCCATTGTGATATTGTCGATGAGTATCATTTGCTAGAAAGCTTTCTAGAACCAAAATTGGCTATCTTTGGTGACCCCTGTCAATGCACATACATTGAGAGACTTAGAGTCCCACATTACACTTCCTTCAGAACTCATAGATTTGGAAAGTCAACTGCTGAGATTTTGAACAAACTGTTTGACCTTAATATAGTCTCAGTTAAGAAAGAAGACGACATCGTTGAATTCTTTAACCCTTTTGAAGTTGACCCCACTGAGCATATCTCTGCCTCTGAAGAAGAAGTCTTGGACTTTGTTTCTGACCAAGTGGTGACCACTAGCTCAGAGGAACTAGCAGGACTTGAGTTTGCAGAAACAACTTTCTACTGCACAACATTGGCCGCAGCTGTTGCTGAAAATCCTGCTAAGACTTTCATCTCTCTGACTAGACACACCCACAAACTCACCATTGGGGAACTAAATGCCAGGTCTAACTCCTAGATGCCAGGTCTAACTCCTAGAGCTGACCTCACTGACACATACAAAATCATTGCCATTGCTTTCTTGTTGTCAGCTTGCATTTACTTCCAAAATAGCCACTACCAACCTGTTGCTGGAGACAACTTGCACCGTTTGCCTTTTGGTGGCCAATATCAAGACGGCACCAAAAAGATATCTTATTTTCCACAACAGCAGTCATACTTTCATTCTGGAAACAAATTAAATGTCCTCATACTTATCTTCATTCTCACGTTGGGTATTGTCCTCACCAATAAATTTAGTTTTAGCTTTAGTCGTACTACTCACCAGCATTCTTGCTATAACACACATTCAGCAACCAACAATACACAACCATTGTCAGGTCATCATTGAATGTCCTCATACTTATCTTCATTCTCACGTTGGGTATTGTCCTCACCAATAAATTTAGTTTTAGCTTTAGTCGTACTACTCACCAGCATTCTTGCTATAACACACATTCAGCAACCAACAATACACAACCATTGTCAGGTCATCATTGACGGTGCTGCAATAGTCATAACAAATTGTGAGAACACACCAGAAGTGCTTAAAGCAATAAACTTCTCCCCTTGGAACGGGTTAAGTTTTCCTAAATTTGAAAATTAAATGCCTGACACAACACCTGTTGCTGCCACTTCAAGTGCACCACCCACAGCCAAAGATGCTGGTGCCAAAGCTCCTTCTGACTTCTCAAATCCCAATACAGCTCCTAGTCTCAGTGATTTGAAGAAAGTCAAGTATGTCTCCACCGTGACCTCCGTGGCCACACCAGCTGAAATTGAAGCCCTAGGCAAAATCTTCACCGCTATGGGCCTTGCCGCCAATGAGACTGGTCCGGCCATGTGGGATCTAGCTCGTGCATATGCTGATGTGCAGAGTTCTAAATCGGCACAGCTGATTGGAGCTACCCCTTCCAACCCTGCACTATCACGCCGAGCCCTTGCTGCTCAGTTTGATCGAATCAATATAACCCCCAGGCAATTTTGCATGTACTTTGCCAAAGTTGTTTGGAACATACTTCTCGACAGCAACATTCCACCAGCAAATTGGGCCAAACTTGGTTACCAAGAAGATACAAAATTTGCTGCATTTGACTTCTTCGATGGAGTCACCAACCCTGCCAGCCTGCAGCCTGCTGATGGTCTTATCAGGCAGCCAAATGAGAAAGAACTAGCTGCTCACTCCGTAGCTAAGTACGGCGCCTTGGCTAGGCAAAAGATCTCCACAGGTAATTATATTACCACACTTGGAGAAGTCACACGTGGACACATGGGAGGAGCTAACACCATGTACGCGATAGACGCACCCCCTGAACTTTAA

>Sp13_P8_a8

ATGGAAAGATCAACTCTGATTAATTTACTTCAATTGCACCACTTCGAGCCAAAACTCAGTGTTGAAGGAATCATAGTTGTGCACGGAATTGCAGGCACTGGGAAAACCACTTTACTTAGGACTTTATTTTCTGCTTACCCTAGCTTAGTTATAGGTTCACCTAGGCCTTGCTATTTAGATAAACAAAACAAAATTTCACAAGTTTGCTTATCTTGCTTTCCCAATACCCATTGTGATATTGTCGATGAGTATCATTTGCTAGAAAGCTTTCTAGAACCAAAATTGGCTATCTTTGGTGACCCCTGTCAATGCACATACATTGAGAGACTTAGAGTCCCACATTACACTTCCTTCAGAACTCATAGATTTGGAAAGTCAACTGCTGAGATTTTGAACAAACTGTTTGACCTTAATATAGTCTCAGTTAAGAAAGAAGACGACATCGTTGAATTCTTTAACCCTTTTGAAGTTGACCCCACTGAGCATATCTCTGCCTCTGAAGAAGAAGTCTTGGACTTTGTTTCTGACCAAGTGGTGACCACTAGCTCAGAGGAACTAGCAGGACTTGAGTTTGCAGAAACAACTTTCTACTGCACAACATTGGCCGCAGCTGTTGCTGAAAATCCTGCTAAGACTTTCATCTCTCTGACTAGACACACCCACAAACTCACCATTGGGGAACTAAATGCCAGGTCTAACTCCTAGATGCCAGGTCTAACTCCTAGAGCTGACCTCACTGACACATACAAAATCATTGCCATTGCTTTCTTGTTGTCAGCTTGCATTTACTTCCAAAATAGCCACTACCAACCTGTTGCTGGAGACAACTTGCACCGTTTGCCTTTTGGTGGCCAATATCAAGACGGCACCAAAAAGATATCTTATTTTCCACAACAGCAGTCATACTTTCATTCTGGAAACAAATTAAATGTCCTCATACTTATCTTCATTCTCACGTTGGGTATTGTCCTCACCAATAAATTTAGTTTTAGCTTTAGTCGTACTACTCACCAGCATTCTTGCTATAACACACATTCAGCAACCAACAATACACAACCATTGTCAGGTCATCATTGAATGTCCTCATACTTATCTTCATTCTCACGTTGGGTATTGTCCTCACCAATAAATTTAGTTTTAGCTTTAGTCGTACTACTCACCAGCATTCTTGCTATAACACACATTCAGCAACCAACAATACACAACCATTGTCAGGTCATCATTGACGGTGCTGCAATAGTCATAACAAATTGTGAGAACACACCAGAAGTGCTTAAAGCAATAAACTTCTCCCCTTGGAACGGGTTAAGTTTTCCTAAATTTGAAAATTAAATGCCTGACACAACACCTGTTGCTGCCACTTCAAGTGCACCACCCACAGCCAAAGATGCTGGTGCCAAAGCTCCTTCTGACTTCTCAAATCCCAATACAGCTCCTAGTCTCAGTGATTTGAAGAAAGTCAAGTATGTCTCCACCGTGACCTCCGTGGCCACACCAGCTGAAATTGAAGCCCTAGGCAAAATCTTCACCGCTATGGGCCTTGCCGCCAATGAGACTGGTCCGGCCATGTGGGATCTAGCTCGTGCATATGCTGATGTGCAGAGTTCTAAATCGGCACAGCTGATTGGAGCTACCCCTTCCAACCCTGCACTATCACGCCGAGCCCTTGCTGCTCAGTTTGATCGAATCAATATAACCCCCAGGCAATTTTGCATGTATTTTGCCAAAGTTGTTTGGAACATACTTCTCGACAGCAACATTCCACCAGCAAATTGGGCCAAACTTGGTTACCAAGAAGATACAAAATTTGCTGCATTTGACTTCTTCGATGGAGTCACCAACCCTGCCAGCCTGCAGCCTGCTGATGGTCTTATCAGGCAGCCAAATGAGAAAGAACTAGCTGCTCACTCCGTAGCTAAGTACGGCGCCTTGGCTAGGCAAAAGATCTCCACAGGTAATTATATTACCACACTTGGAGAAGTCACACGTGGACACATGGGAGGAGCTAACACCATGTACGCGATAGACGCACCCCCTGAACTTTAA

>Sp13_P8_a7

ATGGAAAGATCAACTCTGACTAATTTACTTCAATTGCACCACTTCGAGCCAAAACTCAGTGTTGAAGGAATCATAGTTGTGCACGGAATTGCAGGCACTGGGAAAACCACTTTACTTAGGACTTTATTTTCTGCTTACCCTAGCTTAGTTATAGGTTCACCTAGGCCTTGCTATTTAGATAAACAAAACAAAATTTCACAAGTTTGCTTATCTTGCTTTCCCAATACCCATTGTGATATTGTCGATGAGTATCATTTGCTAGAAAGCTTTCTAGAACCAAAATTGGCTATCTTTGGTGACCCCTGTCAATGCACATACATTGAGAGACTTAGAGTCCCACATTACACTTCCTTCAGAACTCATAGATTTGGAAAGTCAACTGCTGAGATTTTGAACAAACTGTTTGACCTTAATATAGTCTCAGTTAAGAAAGAAGACGACATCGTTGAATTCTTTAACCCTTTTGAAGTTGACCCCACTGAGCATATCTCTGCCTCTGAAGAAGAAGTCTTGGACTTTGTTTCTGACCAAGTGGTGACCACTAGCTCAGAGGAACTAGCAGGACTTGAGTTTGCAGAAACAACTTTCTACTGCACAACATTGGCCGCAGCTGTTGCTGAAAATCCTGCTAAGACTTTCATCTCTCTGACTAGACACACCCACAAACTCACCATTGGGGAACTAAATGCCAGGTCTAACTCCTAGATGCCAGGTCTAACTCCTAGAGCTGACCTCACTGACACATACAAAATCATTGCCATTGCTTTCTTGTTGTCAGCTTGCATTTACTTCCAAAATAGCCACTACCAACCTGTTGCTGGAGACAACTTGCACCGTTTGCCTTTTGGTGGCCAATATCAAGACGGCACCAAAAAGATATCTTATTTTCCACAACAGCAGTCATACTTTCATTCTGGAAACAAATTAAATGTCCTCATACTTATCTTCATTCTCACGTTGGGTATTGTCCTCACCAATAAATTTAGTTTTAGCTTTAGCCGTACTACTCACCAGCATTCTTGCTATAACACACATTCAGCAACCAACAATACACAACCATTGTCAGGTCATCATTGAATGTCCTCATACTTATCTTCATTCTCACGTTGGGTATTGTCCTCACCAATAAATTTAGTTTTAGCTTTAGCCGTACTACTCACCAGCATTCTTGCTATAACACACATTCAGCAACCAACAATACACAACCATTGTCAGGTCATCATTGACGGTGCTGCAATAGTCATAACAAATTGTGAGAACACACCAGAAGTGCTTAAAGCAATAAACTTCTCCCCTTGGAACGGGTTAAGTTTTCCTAAATTTGAAAATTAAATGCCTGACACAACACCTGTTGCTGCCACTTCAAGTGCACCACCCACAGCCAAAGATGCTGGTGCCAAAGCTCCTTCTGACTTCTCAAATCCCAATACAGCTCCTAGTCCCAGTGATTTGAAGAAAGTCAAGTATGTCTCCACCGTGACCTCCGTGGCCACACCAGCTGAAATTGAAGCCCTAGGCAAAATCTTCACCGCTATGGGCCTTGCCGCCAATGAGACTGGTCCGGCCATGTGGGATCTAGCTCGTGCATATGCTGATGTGCAGAGTTCTAAATCGGCACAGCTGATTGGAGCTACCCCTTCCAACCCTGCACTATCACGCCGAGCCCTTGCTGCTCAGTTTGATCGAATCAATATAACCCCCAGGCAATTTTGCATGTACTTTGCCAAAGTTGTTTGGAACATACTTCTCGACAGCAACATTCCACCAGCAAATTGGGCCAAACTTGGTTACCAAGAAGATACAAAATTTGCTGCATTTGACTTCTTCGATGGAGTCACCAACCCTGCCAGCCTGCAGCCTGCTGATGGTCTTATCAGGCAGCCAAATGAGAAAGAACTAGCTGCTCACTCCGTAGCTAAGTACGGCGCCTTGGCTAGGCAAAAGATCTCCACAGGTAATTATATTACCACACTTGGAGAAGTCACACGTGGACACATGGGAGGAGCTAACACCATGTACGCGATAGACGCACCCCCTGAACTTTAA

>Sp13_P8_a6

ATGGAAAGATCAACTCTGACTAATTTACTTCAATTGCACCACTTCGAGCCAAAACTCAGTGTTGAAGGAATCATAGTTGTGCACGGAATTGCAGGCACTGGGAAAACCACTTTACTTAGGACTTTATTTTCTGCTTACCCTAGCTTAGTTATAGGTTCACCTAGGCCTTGCTATTTAGATAAACAAAACAAAATTTCACAAGTTTGCTTATCTTGCTTTCCCAATACCCATTGTGATATTGTCGATGAGTATCATTTGCTAGAAAGTTTTCTAGAACCAAAATTGGCTATCTTTGGTGACCCCTGTCAATGCACATACATTGAGAGACTTAGAGTCCCACATTACACTTCCTTCAGAACTCATAGATTTGGAAAGTCAACTGCTGAGATTTTGAACAAACTGTTTGACCTTAATATAGTCTCAGTTAAGAAAGAAGACGACATCGTTGAATTCTTTAACCCTTTTGAAGTTGACCCCACTGAGCATATCTCTGCCTCTGAAGAAGAAGTCTTGGACTTTGTTTCTGACCAAGTGGTGACCACTAGCTCAGAGGAACTAGCAGGACTTGAGTTTGCAGAAACAACTTTCTACTGCACAACATTGGCCGCAGCTGTTGCTGAAAATCCTGCTAAGACTTTCATCTCTCTGACTAGACACACCCACAAACTCACCATTGGGGAACTAAATGCCAGGTCTAACTCCTAGATGCCAGGTCTAACTCCTAGAGCTGACCTCACTGACACATACAAAATCATTGCCATTGCTTTCTTGTTGTCAGCTTGCATTTACTTCCAAAATAGCCACTACCAACCTGTTGCTGGAGACAACTTGCACCGTTTGCCTTTTGGTGGCCAATATCAAGACGGCACCAAAAAGATATCTTATTTTCCACAACAGCAGTCATACTTTCATTCTGGAAACAAATTAAATGTCCTCATACTTATCTTCATTCTCACGTTGGGTATTGTCCTCACCAATAAATTTAGTTTTAGCTTTAGTCGTACTACTCACCAGCATTCTTGCTATAACACACATTCAGCAACCAACAATACACAACCATTGTCAGGTCATCATTGAATGTCCTCATACTTATCTTCATTCTCACGTTGGGTATTGTCCTCACCAATAAATTTAGTTTTAGCTTTAGTCGTACTACTCACCAGCATTCTTGCTATAACACACATTCAGCAACCAACAATACACAACCATTGTCAGGTCATCATTGACGGTGCTGCAATAGTCATAACAAATTGTGAGAACACACCAGAAGTGCTTAAAGCAATAAACTTCTCCCCTTGGAACGGGTTAAGTTTTCCTAAATTTGAAAATTAAATGCCTGACACAACACCTGTTGCTGCCACTTCAAGTGCACCACCCACAGCCAAAGATGCTGGTGCCAAAGCTCCTTCTGACTTCTCAAATCCCAATACAGCTCCTAGTCTCAGTGATTTGAAGAAAGTCAAGTATGTCTCCACCGTGACCTCCGTGGCCACACCAGCTGAAATTGAAGCCCTAGGCAAAATCTTCACCGCTATGGGCCTTGCCGCCAATGAGACTGGTCCGGCCATGTGGGATCTAGCTCGTGCATATGCTGATGTGCAGAGTTCTAAATCGGCACAGCTGATTGGAGCTACCCCTTCCAACCCTGCACTATCACGCCGAGCCCTTGCTGCTCAGTTTGATCGAATCAATATAACCCCCAGGCAATTTTGCATGTACTTTGCCAAAGTTGTTTGGAACATACTTCTCGACAGCAACATTCCACCAGCAAATTGGGCCAAACTTGGTTACCAAGAAGATACAAAATTTGCTGCATTTGACTTCTTCGATGGAGTCACCAACCCTGCCAGCCTGCAGCCTGCTGATGGTCTTATCAGGCAGCCAAATGAGAAAGAACTAGCTGCTCACTCCGTAGCTAAGTACGGCGCCTTGGCTAGGCAAAAGATCTCCACAGGTAATTATATTACCACACTTGGAGAAGTCACACGTGGACACATGGGAGGAGCTAACACCATGTACGCGATAGACGCACCCCCTGAACTTTAA

>Sp13_P8_a5

ATGGAAAGATCAACTCTGATTAATTTACTTCAATTGCACCACTTCGAGCCAAAACTCAGTGTTGAAGGAATCATAGTTGTGCACGGAATTGCAGGCACTGGGGAAACCACTTTACTTAGGACTTTATTTTCTGCTTACCCTAGCTTAGTTATAGGTTCACCTAGGCCTTGCTATTTAGATAAACAAAACAAAATTTCACAAGTTTGCTTATCTTGCTTTCCCAATACCCATTGTGATATTGTCGATGAGTATCATTTGCTAGAAAGCTTTCTAGAACCAAAATTGGCTATCTTTGGTGACCCCTGTCAATGCACATACATTGAGAGACTTAGAGTCCCACATTACACTTCCTTCAGAACTCATAGATTTGGAAAGTCAACTGCTGAGATTTTGAACAAACTGTTTGACCTTAATATAGTCTCAGTTAAGAAAGAAGACGACATCGTTGAATTCTTTAACCCTTTTGAAGTTGACCCCACTGAGCATATCTCTGCCTCTGAAGAAGAAGTCTTGGACTTTGTTTCTGACCAAGTGGTGACCACTAGCTCAGAGGAACTAGCAGGACTTGAGTTTGCAGAAACAACTTTCTACTGCACAACATTGGCCGCAGCTGTTGCTGAAAATCCTGCTAAGACTTTCATCTCTCTGACTAGACACACCCACAAACTCACCATTGGGGAACTAAATGCCAGGTCTAACTCCTAGATGCCAGGTCTAACTCCTAGAGCTGACCTCACTGACACATACAAAATCATTGCCATTGCTTTCTTGTTGTCAGCTTGCATTTACTTCCAAAATAGCCACTACCAACCTGTTGCTGGAGACAACTTGCACCGTTTGCCTTTTGGTGGCCAATATCAAGACGGCACCAAAAAGATATCTTATTTTCCACAACAGCAGTCATACTTTCATTCTGGAAACAAATTAAATGTCCTCATACTTATCTTCATTCTCACGTTGGGTATTGTCCTCACCAATAAATTTAGTTTTAGCTTTAGTCGTACTACTCACCAGCATTCTTGCTATAACACACATTCAGCAACCAACAATACACAACCATTGTCAGGTCATCATTGAATGTCCTCATACTTATCTTCATTCTCACGTTGGGTATTGTCCTCACCAATAAATTTAGTTTTAGCTTTAGTCGTACTACTCACCAGCATTCTTGCTATAACACACATTCAGCAACCAACAATACACAACCATTGTCAGGTCATCATTGACGGTGCTGCAATAGTCATAGCAAATTGTGAGAACACACCAGAAGTGCTTAAAGCAATAAACTTCTCCCCTTGGAACGGGTTAAGTTTTCCTAAATTTGAAAATTAAATGCCTGACACAACACCTGTTGCTGCCACTTCAAGTGCACCACCCACAGCCAAAGATGCTGGTGCCAAAGCTCCTTCTGACTTCTCAAATCCCAATACGGCTCCTAGTCTCAGTGATTTGAAGAAAGTCAAGTATGTCTCCACCGTGACCTCCGTGGCCACACCAGCTGAAATTGAAGCCCTAGGCAAAATCTTCACCGCTATGGGCCTTGCCGCCAATGAGACTGGTCCGGCCATGTGGGATCTAGCTCGTGCATATGCTGATGTGCAGAGTTCTAAATCGGCACAGCTGATTGGAGCTACCCCTTCCAACCCTGCACTATCACGCCGAGCCCTTGCTGCTCAGTTTGATCGAATCAATATAACCCCCAGGCAATTTTGCATGTATTTTGCCAAAGTTGTTTGGAACATACTTCTCGACAGCAACATTCCACCAGCAAATTGGGCCAAACTTGGTTACCAAGAAGATACAAAATTTGCTGCATTTGACTTCTTCGATGGAGTCACCAACCCTGCCAGCCTGCAGCCTGCTGATGGTCTTATCAGGCAGCCAAATGAGAAAGAACTAGCTGCTCACTCCGTAGCTAAGTACGGCGCCTTGGCTAGGCAAAAGATCTCCACAGGTAATTATATTACCACACTTGGAGAAGTCACACGTGGACACATGGGAGGAGCTAACACCATGTACGCGATAGACGCACCCCCTGAACTTTAA

>Sp13_P8_a4

ATGGAAAGATCAACTCTGATTAATTTACTTCAATTGCACCACTTCGAGCCAAAACTCAGTGTTGAAGGAATCATAGTTGTGCACGGAATTGCAGGCACTGGGAAAACCACTTTACTTAGGACTTTATTTTCTGCTTACCCTAGCTTAGTTATAGGTTCACCTAGGCCTTGCTATTTAGATAAACAAAACAAAATTTCACAAGTTTGCTTATCTTGCTTTCCCAATACCCATTGTGATATTGTCGATGAGTATCATTTGCTAGAAAGCTTTCTAGAACCAAAATTGGCTATCTTTGGTGACCCCTGTCAATGCACATACATTGAGAGACTTAGAGTCCCACATTACACTTCCTTCAGAACTCATAGATTTGGAAAGTCAACTGCTGAGATTTTGAACAAACTGTTTGACCTTAATATAGTCTCAGTTAAGAAAGAAGACGACATCGTTGAATTCTTTAACCCTTTTGAAGTTGACCCCACTGAGCATATCTCTGCCTCTGAAGAAGAAGTCTTGGACTTTGTTTCTGACCAAGTGGTGACCACTAGCTCAGAGGAACTAGCAGGACTTGAGTTTGCAGAAACAACTTTCTACTGCACAACATTGGCCGCAGCTGTTGCTGAAAATCCTGCTAAGACTTTCATCTCTCTGACTAGACACACCCACAAACTCACCATTGGGGAACTAAATGCCAGGTCTAACTCCTAGATGCCAGGTCTAACTCCTAGAGCTGACCTCACTGACACATACAAAATCATTGCCATTGCTTTCTTGTTGTCAGCTTGCATTTACTTCCAAAATAGCCACTACCAACCTGTTGCTGGAGACAACTTGCACCGTTTGCCTTTTGGTGGCCAATATCAAGACGGCACCAAAAAGATATCTTATTTTCCACAACAGCAGTCATACTTTCATTCTGGAAACAAATTAAATGTCCTCATACTTATCTTCATTCTCACGTTGGGTATTGTCCTCACCAATAAATTTAGTTTTAGCTTTAGTCGTACTACTCACCAGCATTCTTGCTATAACACACATTCAGCAACCAACAATACACAACCATTGTCAGGTCATCATTGAATGTCCTCATACTTATCTTCATTCTCACGTTGGGTATTGTCCTCACCAATAAATTTAGTTTTAGCTTTAGTCGTACTACTCACCAGCATTCTTGCTATAACACACATTCAGCAACCAACAATACACAACCATTGTCAGGTCATCATTGACGGTGCTGCAATAGTCATAACAAATTGTGAGAACACACCAGAAGTGCTTAAAGCAATAAACTTCTCCCCTTGGAACGGGTTAAGTTTTCCTAAATTTGAAAATTAAATGCCTGACACAACACCTGTTGCTGCCACTTCAAGTGCACCACCCACAGCCAAAGATGCTGGTGCCAAAGCTCCTTCTGACTTCTCAAATCCCAATACAGCTCCTAGTCTCAGTGATTTGAAGAAAGTCAAGTATGTCTCCACCGTGACCTCCGTGGCCACACCAGCTGAAATTGAAGCCCTAGGCAAAATCTTCACCGCTATGGGCCTTGCCGCCAATGAGACTGGTCCGGCCATGTGGGATCTAGCTCGTGCATATGCTGATGTGCAGAGTTCTAAATCGGCACAGCTGATTGGAGCTACTCCTTCCAACCCTGCACTATCACGCCGAGCCCTTGCTGCTCAGTTTGATCGAATCAATATAACCCCCAGGCAATTTTGCATGTACTTTGCCAAAGTTGTTTGGAACATACTTCTCGACAGCAACATTCCACCAGCAAATTGGGCCAAACTTGGTTACCAAGAAGATACAAAATTTGCTGCATTTGACTTCTTCGATGGAGTCACCAACCCTGCCAGCCTGCAGCCTGCTGATGGTCTTATCAGGCAGCCAAATGAGAAAGAACTAGCTGCTCACTCCGTAGCTAAGTACGGCGCCTTGGCTAGGCAAAAGATCTCCACAGGTAATTATATTACCACACTTGGAGAAGTCACACGTGGACACATGGGAGGAGCTAACACCATGTACGCGATAGACGCACCCCCTGAACTTTAA

>Sp13_P8_a3

ATGGAAAGATCAACTCTGATTAATTTACTTCAATTGCACCACTTCGAGCCAAAACTCAGTGTTGAAGGAATCATAGTTGTGCACGGAATTGCAGGCACTGGGAAAACCACTTTACTTAGGACTTTATTTTCTGCTTACCCTAGCTTAGTTATAGGTTCACCTAGGCCTTGCTATTTAGATAAACAAAACAAAATTTCACAAGTTTGCTTATCTTGCTTTCCCAATACCCATTGTGATATTGTCGATGAGTATCATTTGCTAGAAAGCTTTCTAGAACCAAAATTGGCTATCTTTGGTGACCCCTGTCAATGCACATACATTGAGAGACTTAGAGTCCCACATTACACTTCCTTCAGAACTCATAGATTTGGAAAGTCAACTGCTGAGATTTTGAACAAACTGTTTGACCTTAATATAGTCTCAGTTAAGAAAGAAGACGACATCGTTGAATTCTTTAACCCTTTTGAAGTTGACCCCACTGAGCATATCTCTGCCTCTGAAGAAGAAGTCTTGGACTTTGTTTCTGACCAAGTGGTGACCACTAGCTCAGAGGAACTAGCAGGACTTGAGTTTGCAGAAACAACTTTCTACTGCACAACATTGGCCGCAGCTGTTGCTGAAAATCCTGCTAAGACTTTCATCTCTCTGACTAGACACACCCACAAACTCACCATTGGGGAACTAAATGCCAGGTCTAACTCCTAGATGCCAGGTCTAACTCCTAGAGCTGACCTCACTGACACATACAAAATCATTGCCATTGCTTTCTTGTTGTCAGCTTGCATTTACTTCCAAAATAGCCACTACCAACCTGTTGCTGGAGACAACTTGCACCGTTTGCCTTTTGGTGGCCAATATCAAGACGGCACCAAAAAGATATCTTATTTTCCACAACAGCAGTCATACTTTCATTCTGGAAACAAATTAAATGTCCTCATACTTATCTTCATTCTCACGTTGGGTATTGTCCTCACCAATAAATTTAGTTTTAGCTTTAGTCGTACTACTCACCAGCATTCTTGCTATAACACACATTCAGCAACCAACAATACACAACCATTGTCAGGTCATCATTGAATGTCCTCATACTTATCTTCATTCTCACGTTGGGTATTGTCCTCACCAATAAATTTAGTTTTAGCTTTAGTCGTACTACTCACCAGCATTCTTGCTATAACACACATTCAGCAACCAACAATACACAACCATTGTCAGGTCATCATTGACGGTGCTGCAATAGTCATAACAAATTGTGAGAACACACCAGAAGTGCTTAAAGCAATAAACTTCTCCCCTTGGAACGGGTTAAGTTTTCCTAAATTTGAAAATTAAATGCCTGACACAACACCTGTTGCTGCCACTTCAAGTGCACCACCCACAGCCAAAGATGCTGGTGCCAAAGCTCCTTCTGACTTCTCAAATCCCAATACAGCTCCTAGTCTCAGTGATTTGAAGAAAGTCAAGTATGTCTCCACCGTGACCTCCGTGGCCACACCAGCTGAAATTGAAGCCCTAGGCAAAATCTTCACCGCTATGGGCCTTGCCGCCAATGAGACTGGTCCGGCCATGTGGGATCTAGCTCGTGCATATGCTGATGTGCAGAGTTCTAAATCGGCACAGCTGATTGGAGCTACCCCTTCCAACCCTGCACTATCACGCCGAGCCCTTGCTGCTCAGTTTGATCGAATCAATATAACCCCCAGGCAATTTTGCATGTACTTTGCCAAAGTTGTTTGGAACATACTTCTCGACAGCAACATTCCACCAGCAAATTGGGCCAAACTTGGTTACCAAGAAGATACAAAATTTGCTGCATTTGACTTCTTCGATGGAGTCACCAACCCTGCCAGCCTGCAGCCTGCTGATGGTCTTATCAGGCAGCCAAATGAGAAAGAACTAGCTGCTCACTCCGTAGCTAAGTACGGCGCCTTGGCTAGGCAAAAGATCTCCACAGGTAATTATATTACCACACTTGGAGAAGTCACACGTGGACACATGGGAGGGGCTAACACCATGTACGCGATAGACGCACCCCCTGAACTTTAA

>Sp13_P8_a2

ATGGAAAGATCAACTCTGATTAATTTACTTCAATTGCACCACTTCGAGCCAAAACTCAGTGTTGAAGAAATCATAGTTGTGCACGGAATTGCAGGCACTGGGAAAACCACTTTACTTAGGACTTTATTTTCTGCTTACCCTAGCTTAGTTATAGGTTCACCTAGGCCTTGCTATTTAGATAAACAAAACAAAATTTCACAAGTTTGCTTATCTTGCTTTCCCAATACCCATTGTGATATTGTCGATGAGTATCATTTGCTAGAAAGCTTTCTAGAACCAAAATTGGCTATCTTTGGTGACCCCTGTCAATGCACATACATTGAGAGACTTAGAGTCCCACATTACACTTCCTTCAGAACTCATAGATTTGGAAAGTCAACTGCTGAGATTTTGAACAAACTGTTTGACCTTAATATAGTCTCAGTTAAGAAAGAAGACGACATCGTTGAATTCTTTAACCCTTTTGAAGTTGACCCCACTGAGCATATCTCTGCCTCTGAAGAAGAAGTCTTGGACTTTGTTTCTGACCAAGTGGTGACCACTAGCTCAGAGGAACTAGCAGGACTTGAGTTTGCAGAAACAACTTTCTACTGCACAACATTGGCCGCAGCTGTTGCTGAAAATCCTGCTAAGACTTTCATCTCTCTGACTAGACACACCCACAAACTCACCATTGGGGAACTAAATGCCAGGTCTAACTCCTAGATGCCAGGTCTAACTCCTAGAGCTGACCTCACTGACACATACAAAATCATTGCCATTGCTTTCTTGTTGTCAGCTTGCATTTACTTCCAAAATAGCCACTACCAACCTGTTGCTGGAGACAACTTGCACCGTTTGCCTTTTGGTGGCCAATATCAAGACGGCACCAAAAAGATATCTTATTTTCCACAACAGCAGTCATACTTTCATTCTGGAAACAAATTAAATGTCCTCATACTTATCTTCATTCTCACGTTGGGTATTGTCCTCACCAATAAATTTAGTTTTAGCTTTAGTCGCACTACTCACCAGCATTCTTGCTATAACACACATTCAGCAACCAACAATACACAACCATTGTCAGGTCATCATTGAATGTCCTCATACTTATCTTCATTCTCACGTTGGGTATTGTCCTCACCAATAAATTTAGTTTTAGCTTTAGTCGCACTACTCACCAGCATTCTTGCTATAACACACATTCAGCAACCAACAATACACAACCATTGTCAGGTCATCATTGACGGTGCTGCAATAGTCATAACAAATTGTGAGAACACACCAGAAGTGCTTAAAGCAATAAACTTCTCCCCTTGGAACGGGTTAAGTTTTCCTAAATTTGAAAATTAAATGCCTGACACAACACCTGTTGCTGCCACTTCAAGTGCACCACCCACAGCCAAAGATGCTGGTGCCAAAGCTCCTTCTGACTTCTCAAATCCCAATACAGCTCCTAGTCTCAGCGATTTGAAGAAAGTCAAGTATGTCTCCACCGTGACCTCCGTGGCCACACCAGCTGAAATTGAAGCCCTAGGCAAAATCTTCACCGCTATGGGCCTTGCCGCCAATGAGACTGGTCCGGCCATGTGGGATCTAGCTCGTGCATATGCTGATGTGCAGAGTTCTAAATCGGCACAGCTGATTGGAGCTACCCCTTCCAACCCTGCACTATCACGCCGAGCCCTTGCTGCTCAGTTTGATCGAATCAATATAACCCCCAGGCAATTTTGCATGTACTTTGCCAAAGTTGTTTGGAACATACTTCTCGACAGCAACATTCCACCAGCAAATTGGGCCAAACTTGGTTACCAAGAAGATACAAAATTTGCTGCATTTGACTTCTTCGATGGAGTCACCAACCCTGCCAGCCTGCAGCCTGCTGATGGTCTTATCAGGCAGCCAAATGAGAAAGAACTAGCTGCTCACTCCGTAGCTAAGTACGGCGCCTTGGCTAGGCAAAAGATCTCCACAGGTAATTATATTACCACACTTGGAGAAGTCACACGTGGACACATGGGAGGAGCTAACACCATGTACGCGATAGACGCACCCCCTGAACTTTAA

>Sp13_P8_a1

ATGGAAAGATCAACTCTGATTAATTTACTTCAATTGCACCACTTCGAGCCAAAACTCAGTGTTGAAGGAATCATAGTTGTGCACGGAATTGCAGGCACTGGGAAAACCACTTTACTTAGGACTTTATTTTCTGCTTACCCTAGCTTAGTTATAGGTTCACCTAGGCCTTGCTATTTAGATAAACAAAACAAAATTTCACAAGTTTGCTTATCTTGCTTTCCCAATACCCATTGTGATATTGTCGATGAGTATCATTTGCTAGAAAGCTTTCTAGAACCAAAATTGGCTATCTTTGGTGACCCCTGTCAATGCACATACATTGAGAGACTTAGAGTCCCACATTACACTTCCTTCAGAACTCATAGATTTGGAAAGTCAACTGCTGAGATTTTGAACAAACTGTTTGACCTTAATATAGTCTCAGTTAAGAAAGAAGACGACATCGTTGAATTCTTTAACCCTTTTGAAGTTGACCCCACTGAGCATATCTCTGCCTCTGAAGAAGAAGTCTTGGACTTTGTTTCTGACCAAGTGGTGACCACTAGCTCAGAGGAACTAGCAGGACTTGAGTTTGCAGAAACAACTTTCTACTGCACAACATTGGCCGCAGCTGTTGCTGAAAATCCTGCTAAGACTTTCATCTCTCTGACTAGACACACCCACAAACTCACCATTGGGGAACTAAATGCCAGGTCTAACTCCTAGATGCCAGGTCTAACTCCTAGAGCTGACCTCACTGACACATACAAAATCATTGCCATTGCTTTCTTGTTGTCAGCTTGCATTTACTTCCAAAATAGCCACTACCAACCTGTTGCTGGAGACAACTTGCACCGTTTGCCTTTTGGTGGCCAATATCAAGACGGCACCAAAAAGATATCTTATTTTCCACAACAGCAGTCATACTTTCATTCTGGAAACAAATTAAATGTCCTCATACTTATCTTCATTCTCACGTTGGGTATTGTCCTCACCAATAAATTTAGTTTTAGCTTTAGTCGTACTACTCACCAGCATTCTTGCTATAACACACATTCAGCAACCAACAATACACAACCATTGTCAGGTCATCATTGAATGTCCTCATACTTATCTTCATTCTCACGTTGGGTATTGTCCTCACCAATAAATTTAGTTTTAGCTTTAGTCGTACTACTCACCAGCATTCTTGCTATAACACACATTCAGCAACCAACAATACACAACCATTGTCAGGTCATCATTGACGGTGCTGCAATAGTCATAACAAATTGTGAGAACACACCAGAAGTGCTTAAAGCAATAAACTTCTCCCCTTGGAACGGGTTAAGTTTTCCTAAATTTGAAAATTAAATGCCTGACACAACACCTGTTGCTGCCACTTCAAGTGCACCACCCACAGCCAAAGATGCTGGTGCCAAAGCTCCTTCTGACTTCTCAAATCCCAATACAGCTCCTAGTCTCAGTGATTTGAAGAAAGTCAAGTATGTCTCCACCGTGACCTCCGTGGCCACACCAGCTGAAATTGAAGCCCTAGGCAAAATCTTCACCGCTATGGGCCTTGCCGCCAATGAGACTGGTCCGGCCATGTGGGATCTAGCTCGTGCATATGCTGATGTGCAGAGTTCTAAATCGGCACAGCTGATTGGAGCTACCCCTTCCAACCCTGCACTATCACGCCGAGCCCTTGCTGCTCAGTTTGATCGAATCAATATAACCCCCAGGCAATTTTGCATGTACTTTGCCAAAGTTGTTTGGAACATACTTCTCGACAGCAACATTCCACCAGCAAATTGGGCCAAACTTGGTTACCAAGAAGATACAAAATTTGCTGCATTTGACTTCTTCGATGGAGTCACCAACCCTGCCAGCCTGCAGCCTGCTGATGGTCTTATCAGGCAGCCAAATGAGAAAGAACTAGCTGCTCACTCCGTAGCTAAGTACGGCGCCTTGGCTAGGCAAAAGATCTCCACAGGTAATTATATTACCACACTTGGAGAAGTCACACGTGGACACATGGGAGGAGCTAACACCATGTACGCGATAGACGCACCCCCTGAACTTTAA

>PS5*_P1_consensus_sequence

ATGGAAAGATCAACTTTGATCAATTTACTTCTGTTACACAAATTTGAACACAAGATTAACACTGAAGGAATCATTGTTGTGCACGGAATTGCTGGAACTGGGAAAACCACATTGCTTAGGACTTTATTTTCTGCTTACCCTAGCTTAGTTATAGGTTCACCTAGGCCTTGTTACTTAGATAAAGCTAATAAAATTTCACAAGTTTGCCTTTCTTGTTTTCCAAATACCTTGTGTGACATTGTTGACGAGTACCATCTCTTAGAAAGTTTTCCTGAACCAAAACTAGCCATTTTTGGTGACCCCTGTCAGTGCACTTACATTGAAAGGTTGAGAACACCCAACTACACATCCTTCAGAACACACCGATTTGGCAAATCCACTGCTGCTCTACTAAACAAGTTATTTGATCTTAACATTGAGTCAGTCAAAGCACAAGACGACACAGTAGAATACTTTGATCCTTTCGCAGTGGACCCCTCTGAACACATTTCTGCTTCAGAAAAAGAAGTTTTGGAATTTGTAGGTGATCAAGTTGAGACTACAAGCTCTGAAGAACTAGCTGGTCTCGAGTTTAGTGAAGTTACTTTCTACTGTACCACACTTGCTGGTGCTGTTCAAGAAAATCCTGCCAAAACCTTCATTTCACTCACTAGACACACTTCAAAGCTCACAATTGGTGAACTAAATGCCAGGTCTGACTCCTAGATGCCAGGTCTGACTCCTAGAGCTGATCTTACTGACACGTATAAAATCATTGCTATAGCCTTTCTACTGTCAGCTTGCATTTACTTCCAAAACAGTCATTATCAACCAGTTGCAGGTGATAATTTGCACAGACTACCCTTTGGTGGTCAGTATCAAGACGGAACTAAGAAGATCTCTTACTTTCCGCAGCAACAATCCTACTTTCACTCAGGAAACAAGCTTAATGTCCTCATACTTATCTTCATTCTTACACTGGGTATTGTCCTCACCAATAAATTTAGTTTTAGCATTAGCCGTAATACTCACCAGCATCATTGCTACAACACACATTCTGCAACCCAAACAGGTCAATCAGTGCCAGGTCATCATTGAATGTCCTCATACTTATCTTCATTCTTACACTGGGTATTGTCCTCACCAATAAATTTAGTTTTAGCATTAGCCGTAATACTCACCAGCATCATTGCTACAACACACATTCTGCAACCCAAACAGGTCAATCAGTGCCAGGTCATCATTGATGGTGCAGCCATAGTCATAACAAATTGTCCAAACACACCCGAAGTTCTTAAAGCAATCAACTTCTCCCCTTGGAACGGGTTAAGTTTTCCTCAATTGTGAATGGAAAACCAACCTACAGCTTCTAACCCATCAGATGTACCACCAACTGCTGCTCAAGCTGGTGCCCAGAGCCCAGCCGACTTCTCAAATCCTAATACAGCTCCTTCCCTAAGTGATTTGAAGAAGATCAAATACGTGTCAACTGTCACTTCAGTTGCCACGCCTGCTGAAATTGAGGCCCTTGGCAAGATCTTTACTGCCATGGGTTTAGCAGCCAATGAGACCGGACCTGCCATGTGGGACCTCGCTCGTGCTTATGCTGATGTGCAAAGTTCAAAATCTGCACAACTTATAGGTGCCACACCATCCAACCCTGCTTTGTCTAGACGTGCACTTGCTGCACAGTTTGATCGTATCAATATCACACCCAGACAATTCTGCATGTATTTTGCAAAAATTGTTTGGAACATACTGTTAGACAGCAATGTGCCACCTGCCAACTGGGCAAAATTGGGCTATCAGGAAGATACCAAGTTTGCTGCTTTTGACTTCTTTGATGGAGTCACAAATCCAGCTAGTCTACAGCCTGCAGATGGCCTAATCAGGCAGCCCAATGAAAAAGAGCTTGCTGCTCACTCGGTTGCTAAATATGGTGCCCTTGCCCGCCAGAAAATATCCACTGGTAACTACATCACCACCCTTGGTGAAGTTACACGTGGTCACATGGGCGGCGCCAACACTATGTACGCAATTGATGCACCTCCTGAACTTTAA

>PS5*_P8_c14

ATGGAAAGATCAACTTTGATCAATTTACTTCTGTTACACAAATTTGAACACAAGATTAACACTGAAGGAATCATTGTTGTGCACGGAATTGCTGGAACTGGGAAAACCACATTGCTTAGGACTTTATTTTCTGCTTACCCTAGCTTAGTTATAGGTTCACCTAGGCCTTGTTACTTAGATAAAGCTAATAAAATTTCACAAGTTTGCCTTTCTTGTTTTCCAAATACCTTGTGTGACATTGTTGACGAGTACCATCTCTTAGAAAGTTTTCCTGAACCAAAACTAGCCATTTTTGGTGACCCCTGTCAGTGCACTTACATTGAAAGGTTGAGAACACCCAACTACACATCCTTCAGAACACACCGATTTGGCAAATCCACTGCTGCTCTACTAAACAAGTTATTTGATCTTAACATTGAGTCAGTCAAAGCACAAGACGACACAGTAGAATACTTTGATCCTTTCGCAGTGGACCCCTCTGAACACATTTCTGCTTCAGAAAAAGAAGTTTTGGAATTTGTAGGTGATCAAGTTGAGACTACAAGCTCTGAAGAACTAGCTGGTCTCGAGTTTAGTGAAGTTACTTTCTACTGTACCACACTTGCTGGTGCTGTTCAAGAAAATCCTGCCAAAACCTTCATTTCACTCACTAGACACACTTCAAAGCTCACAATTGGTGAACTAAATGCCAGGTCTGACTCCTAGATGCCAGGTCTGACTCCTAGAGCTGATCTTACTGACACGTATAAAATCATTGCTATAGCCTTTCTACTGTCAGCTTGCATTTACTTCCAAAACAGTCATTATCAACCAGTCGCAGGTGATAATTTGCACAGACTACCCTTTGGTGGTCAGTATCAAGATGGAACTAAGAAGATCTCTTACTTTCCGCAGCAACAATCCTACTTTCACTCAGGAAACAAGCTTAATGTCCTCATACTTATCTTCATTCTTACACTGGGTATTGTCCTCACCAATAAATTTAGTTTTAGCATTAGCCGTAATACTCACCAGCATCATTGCTACAACACACATTCTGCAACCCAAACAGGTCAATCAGTGCCAGGTCATCATTGAATGTCCTCATACTTATCTTCATTCTTACACTGGGTATTGTCCTCACCAATAAATTTAGTTTTAGCATTAGCCGTAATACTCACCAGCATCATTGCTACAACACACATTCTGCAACCCAAACAGGTCAATCAGTGCCAGGTCATCATTGATGGTGCAGCCATAGTCATAACAAATTGTCCAAACACACCCGAAGTTCTTAAAGCAATCAACTTCTCCCCTTGGAACGGGTTAAGTTTTCCTCAATTGTGAATGGAAAACCAACCTACAGCTTCTAACCCATCAGATGTACCACCAACTGCTGCTCAAGCTGGTGCCCAGAGCCCAGCCGACTTCTCAAATCCTAATACAGCTCCTTCCCTAAGTGATTTGAAGAAGATCAAATACGTGTCAACTGTCACTTCAGTTGCCACGCCTGCTGAAATTGAGGCCCTTGGCAAGATCTTTACTGCCATGGGTTTAGCAGCCAATGAGACCGGACCTGCCATGTGGGACCTCGCTCGTGCTTATGCTGATGTGCAAAGTTCAAAATCTGCACAACTTATAGGTGCCACACCATCCAACCCTGCTTTGTCTAGACGTGCACTTGCTGCACAGTTTGATCGTATCAATATCACACCCAGACAATTCTGCATGTATTTTGCAAAAATTGTTTGGAACATACTGTTAGACAGCAATGTGCCACCTGCCAACTGGGCAAAATTGGGCTATCAGGAAGATACCAAGTTTGCTGCTTTTGACTTCTTTGATGGAGTCACAAATCCAGCTAGTCTACAGCCTGCAGATGGCCTAATCAGGCAGCCCAATGAAAAAGAGCTTGCTGCTCACTCGGTTGCTAAATATGGTGCCCTTGCCCGCCAGAAAATATCCACTGGTAACTACATCACCACCCTTGGTGAAGTTACACGTGGTCACATGGGCGGCGCCAACACTATGTACGCAATTGATGCACCTCCTGAACTTTAA

>PS5*_P8_c13

ATGGAAAGATCAACTTTGATCAATTTACTTCTGTTACACAAATTTGAACACAAGATTAACACTGAAGGAATCATTGTTGTGCACGGAATTGCTGGAACTGGGAAAACCACATTGCTTAGGACTTTATTTTCTGCTTACCCTAGCTTAGTTATAGGTTCACCTAGGCCTTGTTACTTAGATAAAGCTAATAAAATTTCACAAGTTTGCCTTTCTTGTTTTCCAAATACCTTGTGTGACATTGTTGACGAGTACCATCTCTTAGAAAGTTTTCCTGAACCAAAACTAGCCATTTTTGGTGACCCCTGTCAGTGCACTTACATTGAAAGGTTGAGAACACCCAACTACACATCCTTCAGAACACACCGATTTGGCAAATCCACTGCTGCTCTACTAAACAAGTTATTTGATCTTAACATTGAGTCAGTCAAAGCACAAGACGACACAGTAGAATACTTTGATCCTTTCGCAGTGGACCCCTCTGAACACATTTCTGCTTCAGAAAAAGAAGTTTTGGAATTTGTAGGTGATCAAGTTGAGACTACAAGCTCTGAAGAACTAGCTGGTCTCGAGTTTAGTGAAGTTACTTTCTACTGTACCACACTTGCTGGTGCTGTTCAAGAAAATCCTGCCAAAACCTTCATTTCACTCACTAGACACACTTCAAAGCTCACAATTGGTGAACTAAATGCCAGGTCTGACTCCTAGATGCCAGGTCTGACTCCTAGAGCTGATCTTACTGACACGTATAAAATCATTGCTATAGCCTTTCTACTGTCAGCTTGCATTTACTTCCAAAACAGTCATTATCAACCAGTCGCAGGTGATAATTTGCACAGACTACCCTTTGGTGGTCAGTATCAAGACGGAACTAAGAAGATCTCTTACTTTCCGCAGCAACAATCCTACTTTCACTCAGGAAACAAGCTTAATGTCCTCATACTTATCTTCATTCTTACACTGGGTATTGTCCTCACCAATAAATTTAGTTTTAGCATTAGCCGTAATACTCACCAGCATCATTGCTACAACACACATTCTGCAACCCAAACAGGTCAATCAGTGCCAGGTCATCATTGAATGTCCTCATACTTATCTTCATTCTTACACTGGGTATTGTCCTCACCAATAAATTTAGTTTTAGCATTAGCCGTAATACTCACCAGCATCATTGCTACAACACACATTCTGCAACCCAAACAGGTCAATCAGTGCCAGGTCATCATTGATGGTGCAGCCATAGTCATAACAAATTGTCCAAACACACCCGAAGTTCTTAAAGCAATCAACTTCTCCCCTTGGAACGGGTTAAGTTTTCCTCAATTGTGAATGGAAAACCAACCTACAGCTTCTAACCCATCAGATGTACCACCAACTGCTGCTCAAGCTGGTGCCCAGAGCCCAGCCGACTTCTCAAATCCTAATACAGCTCCTTCCCTAAGTGATTTGAAGAAGATCAAATACGTGTCAACTGTCACTTCAGTTGCCACGCCTGCTGAAATTGAGGCCCTTGGCAAGATCTTTACTGCCATGGGTTTAGCAGCCAATGAGACCGGACCTGCCATGTGGGACCTCGCTCGTGCTTATGCTGATGTGCAAAGTTCAAAATCTGCACAACTTATAGGTGCCACACCATCCAACCCTGCTTTGTCTAGACGTGCACTTGCTGCACAGTTTGATCGTATCAATATCACACCCAGACAATTCTGCATGTATTTTGCAAAAATTGTTTGGAACATACTGTTAGACAGCAATGTGCCACCTGCCAACTGGGCAAAATTGGGCTATCAGGAAGATACCAAGTTTGCTGCTTTTGACTTCTTTGATGGAGTCACAAATCCAGCTAGTCTACAGCCTGCAGATGGCCTAATCAGGCAGCCCAATGAAAAAGAGCTTGCTGCTCACTCGGTTGCTAAATATGGTGCCCTTGCCCGCCAGAAAATATCCACTGGTAACTACATCACCACCCTTGGTGAAGTTACACGTGGTCACATGGGCGGCGCCAACACTATGTACGCAATTGATGCACCTCCTGAACTTTAA

>PS5*_P8_c12

ATGGAAAGATCAACTTTGATCAATTTACTTCTGTTACACAAATTTGAACACAAGATTAACACTGAAGGAATCATTGTTGTGCACGGAATTGCTGGAACTGGGAAAACCACATTGCTTAGGACTTTATTTTCTGCTTACCCTAGCTTAGTTATAGGTTCACCTAGGCCTTGTTACTTAGATAAAGCTAATAAAATTTCACAAGTTTGCCTTTCTTGTTTTCCAAATACCTTGTGTGACATTGTTGACGAGTACCATCTCTTAGAAAGTTTTCCTGAACCAAAACTAGCCATTTTTGGTGACCCCTGTCAGTGCACTTACATTGAAAGGTTGAGAACACCCAACTACACATCCTTCAGAACACACCGATTTGGCAAATCCACTGCTGCTCTACTAAACAAGTTATTTGATCTTAACATTGAGTCAGTCAAAGCACAAGACGACACAGTAGAATACTTTGATCCTTTCGCAGTGGACCCCTCTGGACACATTTCTGCTTCAGAAAAAGAAGTTTTGGAATTTGTAGGTGATCAAGTTGAGACTACAAGCTCTGAAGAACTAGCTGGTCTCGAGTTTAGTGAAGTTACTTTCTACTGTACCACACTTGCTGGTGCTGTTCAAGAAAATCCTGCCAAAACCTTCATTTCACTCACTAGACACACTTCAAAGCTCACAATTGGTGAACTAAATGCCAGGTCTGACTCCTAGATGCCAGGTCTGACTCCTAGAGCTGATCTTACTGACACGTATAAAATCATTGCTATAGCCTTTCTACTGTCAGCTTGCATTTACTTCCAAAACAGTCATTATCAACCAGTCGCAGGTGATAATTTGCACAGACTACCCTTTGGTGGTCAGTATCAAGACGGAACTAAGAAGATCTCTTACTTTCCGCAGCAACAATCCTACTTTCACTCAGGAAACAAGCTTAATGTCCTCATACTTATCTTCATTCTTACACTGGGTATTGTCCTCACCAATAAATTTAGTTTTAGCATTAGCCGTAATACTCACCAGCATCATTGCTACAACACACATTCTGCAACCCAAACAGGTCAATCAGTGCCAGGTCATCATTGAATGTCCTCATACTTATCTTCATTCTTACACTGGGTATTGTCCTCACCAATAAATTTAGTTTTAGCATTAGCCGTAATACTCACCAGCATCATTGCTACAACACACATTCTGCAACCCAAACAGGTCAATCAGTGCCAGGTCATCATTGATGGTGCAGCCATAGTCATAACAAATTGTCCAAACACACCCGAAGTTCTTAAAGCAATCAACTTCTCCCCTTGGAACGGGTTAAGTTTTCCTCAATTGTGAATGGAAAACCAACCTACAGCTTCTAACCCATCAGATGTACCACCAACTGCTGCTCAAGCTGGTGCCCAGAGCCCAGCCGACTTCTCAAATCCTAATACAGCTCCTTCCCTAAGTGATTTGAAGAAGATCAAATACGTGTCAACTGTCACTTCAGTTGCCACGCCTGCTGAAATTGAGGCCCTTGGCAAGATCTTTACTGCCATGGGTTTAGCAGCCAATGAGACCGGACCTGCCATGTGGGACCTCGCTCGTGCTTATGCTGATGTGCAAAGTTCAAAATCTGCACAACTTATAGGTGCCACACCATCCAACCCTGCTTTGTCTAGACGTGCACTTGCTGCACAGTTTGATCGTATCAATATCACACCCAGACAATTCTGCATGTATTTTGCAAAAATTGTTTGGAACATACTGTTAGACAGCAATGTGCCACCTGCCAACTGGGCAAAATTGGGCTATCAGGAAGATACCAAGTTTGCTGCTTTTGACTTCTTTGATGGAGTCACAAATCCAGCTAGTCTACAGCCTGCAGATGGCCTAATCAGGCAGCCCAATGAAAAAGAGCTTGCTGCTCACTCGGTTGCTAAATATGGTGCCCTTGCCCGCCAGAAAATATCCACTGGTAACTGCATCACCACCCTTGGTGAAGTTACACGTGGTCACATGGGCGGCGCCAACACTATGTACGCAATTGATGCACCTCCTGAACTTTAA

>PS5*_P8_c11

ATGGAAAGATCAACTTTGATCAATTTACTTCTGTTACACAAATTTGAACACAAGATTAACACTGAAGGAATCATTGTTGTGCACGGAATTGCTGGAACTGGGAAAACCACATTGCTTAGGACTTTATTTTCTGCTTACCCTAGCTTAGTTATAGGTTCACCTAGGCCTTGTTACTTAGATAAAGCTAATAAAATTTCACAAGTTTGCCTTTCTTGTTTTCCAAATACCTTGTGTGACATTGTTGACGAGTACCATCTCTTAGAAAGTTTTCCTGAACCAAAACTAGCCATTTTTGGTGACCCCTGTCAGTGCACTTACATTGAAAGGTTGAGAACACCCAACTACACATCCTTCAGAACACACCGATTTGGCAAATCCACTGCTGCTCTACTAAACAAGTTATTTGATCTTAACATTGAGTCAGTCAAAGCACAAGACGACACAGTAGAATACTTTGATCCTTTCGCAGTGGGCCCCTCTGAACACATTTCTGCTTCAGAAAAAGAAGTTTTGGAATTTGTAGGTGATCAAGTTGAGACTACAAGCTCTGAAGAACTAGCTGGTCTCGAGTTTAGTGAAGTTACTTTCTACTGTACCACACTTGCTGGTGCTGTTCAAGAAAATCCTGCCAAAACCTTCATTTCACTCACTAGACACACTTCAAAGCTCACAATTGGTGAACTAAATGCCAGGTCTGACTCCTAGATGCCAGGTCTGACTCCTAGAGCTGATCTTACTGACACGTATAAAATCATTGCTATAGCCTTTCTACTGTCAGCTTGCATTTACTTCCAAAACAGTCATTATCAACCAGTCGCAGGTGATAATTTGCACAGACTACCCTTTGGTGGTCAGTATCAAGACGGAACTAAGAAGATCTCTTACTTTCCGCAGCAACAATCCTACTTTCACTCAGGAAACAAGCTTAATGTCCTCATACTTATCTTCATTCTTACACTGGGTATTGTCCTCACCAATAAATTTAGTTTTAGCATTAGCCGTAATACTCACCAGCATCATTGCTACAACACACATTCTGCAACCCAAACAGGTCAATCAGTGCCAGGTCATCATTGAATGTCCTCATACTTATCTTCATTCTTACACTGGGTATTGTCCTCACCAATAAATTTAGTTTTAGCATTAGCCGTAATACTCACCAGCATCATTGCTACAACACACATTCTGCAACCCAAACAGGTCAATCAGTGCCAGGTCATCATTGATGGTGCAGCCATAGTCATAACAAATTGTCCAAACACACCCGAAGTTCTTAAAGCAATCAACTTCTCCCCTTGGAACGGGTTAAGTTTTCCTCAATTGTGAATGGAAAACCAACCTACAGCTTCTAACCCATCAGATGTACCACCAACTGCTGCTCAAGCTGGTGCCCAGAGCCCAGCCGACTTCTCAAATCCTAATACAGCTCCTTCCCTAAGTGATTTGAAGAAGATCAAATACGTGTCAACTGTCACTTCAGTTGCCACGCCTGCTGAAATTGAGGCCCTTGGCAAGATCTTTACTGCCATGGGTTTAGCAGCCAATGAGACCGGACCTGCCATGTGGGACCTCGCTCGTGCTTATGCTGATGTGCAAAGTTCAAAATCTGCACAACTTATAGGTGCCACACCATCCAACCCTGCTTTGTCTAGACGTGCACTTGCTGCACAGTTTGATCGTATCAATATCACACCCAGACAATTCTGCATGTATTTTGCAAAAATTGTTTGGAACATACTGTTAGACAGCAATGTGCCACCTGCCAACTGGGCAAAATTGGGCTATCAGGAAGATACCAAGTTTGCTGCTTTTGACTTCTTTGATGGAGTCACAAATCCAGCTAGTCTACAGCCTGCAGATGGCCTAATCAGGCAGCCCAATGAAAAAGAGCTTGCTGCTCACTCGGTTGCTAAATATGGTGCCCTTGCCCGCCAGAAAATATCCACTGGTAACTACATCACCACCCTTGGTGAAGTTACACGTGGTCACATGGGCGGCGCCAACACTATGTACGCAATTGATGCACCTCCTGAACTTTAA

>PS5*_P8_c10

ATGGAAAGATCAACTTTGATCAATTTACTTCTGTTACACAAATTTGAACACAAGATTAACACTGAAGGAATCATTGTTGTGCACGGAATTGCTGGAACTGGGAAAACCACATTGCTTAGGACTTTATTTTCTGCTTACCCTAGCTTAGTTATAGGTTCACCTAGGCCTTGTTACTTAGATAAAGCTAATAAAATTTCACAAGTTTGCCTTTCTTGTTTTCCAAATACCTTGTGTGACATTGTTGACGAGTACCATCTCTTAGAAAGTTTTCCTGAACCAAAACTAGCCATTTTTGGTGACCCCTGTCAGTGCACTTACATTGAAAGGTTGAGAACACCCAACTACACATCCTTCAGAACACACCGATTTGGCAAATCCACTGCTGCTCTACTAAACAAGTTATTTGATCTTAACATTGAGTCAGTCAAAGCACAAGACGACACAGTAGAATACTTTGATCCTTTCGCAGTGGACCCCTCTGAACACATTTCTGCTTCAGAAAAAGAAGTTTTGGAATTTGTAGGTGATCAAGTTGAGACTACAAGCTCTGAAGAACTAGCTGGTCTCGAGTTTAGTGAAGTTACTTTCTACTGTACCACACTTGCTGGTGCTGTTCAAGAAAATCCTGCCAAAACCTTCATTTCACTCACTAGACACACTTCAAAGCTCACAATTGGTGAACTAAATGCCAGGTCTGACTCCTAGATGCCAGGTCTGACTCCTAGAGCTGATCTTACTGACACGTATAAAATCATTGCTATAGCCTTTCTACTGTCAGCTTGCATTTACTTCCAAAACAGTCATTATCAACCAGTCGCAGGTGATAATTTGCACAGACTACCCTTTGGTGGTCAGTATCAAGACGGAACTAAGAAGATCTCTTACTTTCCGCAGCAACAATCCTACTTTCACTCAGGAAACAAGCTTAATGTCCTCATACTTATCTTCATTCTTACACTGGGTATTGTCCTCACCAATAAATTTAGTTTTAGCATTAGCCGTAATACTCACCAGCATCATTGCTACAACACACATTCTGCAACCCAAACAGGTCAATCAGTGCCAGGTCATCATTGAATGTCCTCATACTTATCTTCATTCTTACACTGGGTATTGTCCTCACCAATAAATTTAGTTTTAGCATTAGCCGTAATACTCACCAGCATCATTGCTACAACACACATTCTGCAACCCAAACAGGTCAATCAGTGCCAGGTCATCATTGATGGTGCAGCCATAGTCATAACAAATTGTCCAAACACACCCGAAGTTCTTAAAGCAATCAACTTCTCCCCTTGGAACGGGTTAAGTTTTCCTCAATTGTGAATGGAAAACCAACCTACAGCTTCTAACCCATCAGATGTACCACCAACTGCTGCTCAAGCTGGTGCCCAGAGCCCAGCCGACTTCTCAAATCCTAATACAGCTCCTTCCCTAAGTGATTTGAAGAAGATCAAATACGTGTCAACTGTCACTTCAGTTGCCACGCCTGCTGAAATTGAGGCCCTTGGCAAGATCTTTACTGCCATGGGTTTAGCAGCCAATGAGACCGGACCTGCCATGTGGGACCTCGCTCGTGCTTATGCTGATGTGCAAAGTTCAAAATCTGCACAACTTATAGGTGCCACACCATCCAACCCTGCTTTGTCTAGACGTGCACTTGCTGCACAGTTTGATCGTATCAATATCACACCCAGACAATTCTGCATGTATTTTGCAAAAATTGTTTGGAACATACTGTTAGACAGCAATGTGCCACCTGCCAACTGGGCAAAATTGGGCTATCAGGAAGATACCAAGTTTGCTGCTTTTGACTTCTTTGATGGAGTCACAAATCCAGCTAGTCTACAGCCTGCAGATGGCCTAATCAGGCAGCCCAATGAAAAAGAGCTTGCTGCTCACTCGGTTGCTAAATATGGTGCCCTTGCCCGCCAGAAAATATCCACTGGTAACTACATCACCACCCTTGGTGAAGTTACACGTGGTCACATGGGCGGCGCCAACACTATGTACGCAATTGATGCACCTCCTGAACTTTAA

>PS5*_P8_c9

ATGGAAAGATCAACTTTGATCAATTTACTTCTGTTACACAAATTTGAACACAAGATTAACACTGAAGGAATCATTGTTGTGCACGGAATTGCTGGAACTGGGAAAACCACATTGCTTAGGACTTTATTTTCTGCTTACCCTAGCTTAGTTATAGGTTCACCTAGGCCTTGTTACTTAGATAAAGCTAATAAAATTTCACAAGTTTGCCTTTCTTGTTTTCCAAATACCTTGTGTGACATTGTTGACGAGTACCATCTCTTAGAAAGTTTTCCTGAACCAAAACTAGCCATTTTTGGTGACCCCTGTCAGTGCACTTACATTGAAAGGTTGAGAACACCCAACTACACATCCTTCAGAACACACCGATTTGGCAAATCCACTGCTGCTCTACTAAACAAGTTATTTGATCTTAACATTGAGTCAGTCAAAGCACAAGACGACACAGTAGAATACTTTGATCCTTTCGCAGTGGACCCCTCTGAACACATTTCTGCTTCAGAAAAAGAAGTTTTGGAATTTGTAGGTGATCAAGTTGAGACTACAAGCTCTGAAGAACTAGCTGGTCTCGAGTTTAGTGAAGTTACTTTCTACTGTACCACACTTGCTGGTGCTGTTCAAGAAAATCCTGCCAAAACCTTCATTTCACTCACTAGACACACTTCAAAGCTCACAATTGGTGAACTAAATGCCAGGTCTGACCCCTAGATGCCAGGTCTGACCCCTAGAGCTGATCTTACTGACACGTATAAAATCATTGCTATAGCCTTTCTACTGTCAGCTTGCATTTACTTCCAAAACAGTCATTATCAACCAGTTGCAGGTGATAATTTGCACAGACTACCCTTTGGTGGTCAGTATCAAGACGGAACTAAGAAGATCTCTTACTTTCCGCAGCAACAATCCTACTTTCACTCAGGAAACAAGCTTAATGTCCTCATACTTATCTTCATTCTTACACTGGGTATTGTCCTCACCAATAAATTTAGTTTTAGCATTAGCCGTAATACTCACCAGCATCATTGCTACAACACACATTCTGCAACCCAAACAGGTCAATCAGTGCCAGGTCATCATTGAATGTCCTCATACTTATCTTCATTCTTACACTGGGTATTGTCCTCACCAATAAATTTAGTTTTAGCATTAGCCGTAATACTCACCAGCATCATTGCTACAACACACATTCTGCAACCCAAACAGGTCAATCAGTGCCAGGTCATCATTGATGGTGCAGCCATAGTCATAACAAATTGTCCAAACACACCCGAAGTTCTTAAAGCAATCAACTTCTCCCCTTGGAACGGGTTAAGTTTTCCTCAATTGTGAATGGAAAACCAACCTACAGCTTCTAACCCATCAGATGTACCACCAACTGCTGCTCAAGCTGGTGCCCAGAGCCCAGCCGACTTCTCAAATCCTAATACAGCTCCTTCCCTAAGTGATTTGAAGAAGATCAAATACGTGTCAACTGTCACTTCAGTTGCCACGCCTGCTGAAATTGAGGCCCTTGGCAAGATCTTTACTGCCATGGGTTTAGCAGCCAATGAGACCGGACCTGCCATGTGGGACCTCGCTCGTGCTTATGCTGATGTGCAAAGTTCAAAATCTGCACAACTTATAGGTGCCACACCATCCAACCCTGCTTTGTCTAGACGTGTACTTGCTGCACAGTTTGATCGTATCAATATCACACCCAGACAATTCTGCATGTATTTTGCAAAAATTGTTTGGAACATACTGTTAGACAGCAATGTGCCACCTGCCAACTGGGCAAAATTGGGCTATCAGGAAGATACCAAGTTTGCTGCTTTTGACTTCTTTGATGGAGTCACAAATCCAGCTAGTCTACAGCCTGCAGATGGCCTAATCAGGCAGCCCAATGAAAAAGAGCTTGCTGCTCACTCGGTTGCTAAATATGGTGCCCTTGCCCGCCAGAAAATATCCACTGGTAACTACATCACCACCCTTGGTGAAGTTACACGTGGTCACATGGGCGGCGCCAACACTATGTACGCAATTGATGCACCTCCTGAACTTTAA

>PS5*_P8_c8

ATGGAAAGATCAACTTTGATCAATTTACTTCTGTTACACAAATTTGAACACAAGATTAACACTGAAGGAATCATTGTTGTGCACGGAATTGCTGGAACTGGGAAAACCACATTGCTTAGGACTTTATTTTCTGCTTACCCTAGCTTAGTTATAGGTTCACCTAGGCCTTGTTACTTAGATAAAGCTAATAAAATTTCACAAGTTTGCCTTTCTTGTTTTCCAAATACCTTGTGTGACATTGTTGACGAGTACCATCTCTTAGAAAGTTTTCCTGAACCAAAACTAGCCATTTTTGGTGACCCCTGTCAGTGCACTTACATTGAAAGGTTGAGAACACCCAACTACACATCCTTCAGAACACACCGATTTGGCAAATCCACTGCTGCTCTACTAAACAAGTTATTTGATCTTAACATTGAGTCAGTCAAAGCACAAGACGACACAGTAGAATACTTTGATCCTTTCGCAGTGGACCCCTCTGAACACATTTCTGCTTCAGAAAAAGAAGTTTTGGAATTTGTAGGTGATCAAGTTGAGACTACAAGCTCTGAAGAACTAGCTGGTCTCGAGTTTAGTGAAGTTACTTTCTACTGTACCACACTTGCTGGTGCTGTTCAAGAAAATCCTGCCAAAACCTTCATTTCACTCACTAGACACACTTCAAAGCTCACAATTGGTGAACTAAATGCCAGGTCTGACTCCTAGATGCCAGGTCTGACTCCTAGAGCTGATCTTACTGACACGTATAAAATCATTGCTATAGCCTTTCTACTGTCAGCTTGCATTTACTTCCAAAACAGTCATTATCAACCAGTTGCAGGTGATAATTTGCACAGACTACCCTTTGGTGGTCAGTATCAAGACGGAACTAAGAAGATCTCTTACTTTCCGCAGCAACAATCCTACTTTCACTCAGGGAACAAGCTTAATGTCCTCATACTTATCTTCATTCTTACACTGGGTATTGTCCCCACCAATAAATTTAGTTTTAGCATTAGCCGTAATACTCACCAGCATCATTGCTACAACACACATTCTGCAATCCAAACAGGTCAATCAGTGCCAGGTCATCATTGAATGTCCTCATACTTATCTTCATTCTTACACTGGGTATTGTCCCCACCAATAAATTTAGTTTTAGCATTAGCCGTAATACTCACCAGCATCATTGCTACAACACACATTCTGCAATCCAAACAGGTCAATCAGTGCCAGGTCATCATTGATGGTGCAGCCATAGTCATAACAAATTGTCCAAACACACCCGAAGTTCTTAAAGCAATCAACTTCTCCCCTTGGAACGGGTTAAGTTTTCCTCAATTGTGAATGGAAAACCAACCTACAGCTTCTAACCCATCAGATGTACCACCAACTGCTGCTCAAGCTGGTGCCCAGAGCCCAGCCGACTTCTCAAATCCTAATACAGCTCCTTCCCTAAGTGATTTGAAGAAGATCAAATACGTGTCAACTGTCACTTCAGTTGCCACGCCTGCTGAAATTGAGGCCCTTGGCAAGATCTTTACTGCCATGGGTTTAGCAGCCAATGAGACCGGACCTGCCATGTGGGACCTCGCTCGTGCTTATGCTGATGTGCAAAGTTCAAAATCTGCACAACTTATAGGTGCCACACCATCCAACCCTGCTTTGTCTAGACGTGCACTTGCTGCACAGTTTGATCGTATCAATATCACACCCAGACAATTCTGCATGTATTTTGCAAAAATTGTTTGGAACATACTGTTAGACAGCAATGTGCCACCTGCCAACTGGGCAAAATTGGGCTATCAGGAAGATACCAAGTTTGCTGCTTTTGACTTCTTTGATGGAGTCACAAATCCAGCTAGTCTACAGCCTGCAGATGGCCTAATCAGGCAGCCCAATGAAAAAGAGCTTGCTGCTCACTCGGTTGCTAAATATGGTGCCCTTGCCCGCCAGAAAATATCCACTGGTAACTACATCACCACCCTTGGTGAAGTTACACGTGGTCACATGGGCGGCGCCAACACTATGTACGCAATTGATGCACCTCCTGAACTTTAA

>PS5*_P8_c7

ATGGAAAGATCAACTTTGATCAATTTACTTCTGTTACACAAATTTGAACACAAGATTAACACTGAAGGAATCATTGTTGTGCACGGAATTGCTGGAACTGGGAAAACCACATTGCTTAGGACTTTATTTTCTGCTTACCCTAGCTTAGTTATAGGTTCACCTAGGCCTTGTTACTTAGATAAAGCTAATAAAATTTCACAAGTTTGCCTTTCTTGTTTTCCAAATACCTTGTGTGACATTGTTGACGAGTACCATCTCTTAGAAAGTTTTCCTGAACCAAAACTAGCCATTTTTGGTGACCCCTGTCAGTGCACTTACATTGAAAGGTTGAGAACACCCAACTACACATCCTTCAGAACACACCGATTTGGCAAATCCACTGCTGCTCTACTAAACAAGTTATTTGATCTTAACATTGAGTCAGTCAAAGCACAAGACGACACAGTAGAATACTTTGATCCTTTCGCAGTGGACCCCTCTGAACACATTTCTGCTTCAGAAAAAGAAGTTTTGGAATTTGTAGGTGATCAAGTTGAGACTACAAGCTCTGAAGAACTAGCTGGTCTCGAGTTTAGTGAAGTTACTTTCTACTGTACCACACTTGCTGGTGCTGTTCAAGAAAATCCTGCCAAAACCTTCATTTCACTCACTAGACACACTTCAAAGCTCACAATTGGTGAACTAAATGCCAGGTCTGACTCCTAGATGCCAGGTCTGACTCCTAGAGCTGATCTTACTGACACGTATAAAATCATTGCTATAGCCTTTCTACTGTCAGCTTGCATTTACTTCCAAAACAGTCATTATCAACCAGTCGCAGGTGATAATTTGCACAGACTACCCTTTGGTGGTCAGTATCAAGACGGAACTAAGAAGATCTCTTACTTTCCGCAGCAACAATCCTACTTTCACTCAGGAAACAAGCTTAATGTCCTCATACTTATCTTCATTCTTACACTGGGTATTGTCCTCACCAATAAATTTAGTTTTAGCATTAGCCGTAATATTCACCAGCATCATTGCTACAACACACATTCTGCAACCCAAACAGGTCAATCAGTGCCAGGTCATCATTGAATGTCCTCATACTTATCTTCATTCTTACACTGGGTATTGTCCTCACCAATAAATTTAGTTTTAGCATTAGCCGTAATATTCACCAGCATCATTGCTACAACACACATTCTGCAACCCAAACAGGTCAATCAGTGCCAGGTCATCATTGATGGTGCAGCCATAGTCATAACAAATTGTCCAAACACACCCGAAGTTCTTAAAGCAATCAACTTCTCCCCTTGGAACGGGTTAAGTTTTCCTCAATTGTGGATGGAAAACCAACCTACAGCTTCTAACCCATCAGATGTACCACCAACTGCTGCTCAAGCTGGTGCCCAGAGCCCAGCCGACTTCTCAAATCCTAATACAGCTCCTTCCCTAAGTGATTTGAAGAAGATCAAATACGTGTCAACTGTCACTTCAGTTGCCACGCCTGCTGAAATTGAGGCCCTTGGCAAGATCTTTACTGCCATGGGTTTAGCAGCCAATGAGACCGGACCTGCCATGTGGGACCTCGCCCGTGCTTATGCTGATGTGCAAAGTTCAAAATCTGCACAACTTATAGGTGCCACACCATCCAACCCTGCTTTGTCTAGACGTGCACTTGCTGCACAGTTTGATCGTATCAATATCACACCCAGACAATTCTGCATGTATTTTGCAAAAATTGTTTGGAACATACTGTTAGACAGCAATGTGCCACCTGCCAACTGGGCAAAATTGGGCTATCAGGAAGATACCAAGTTTGCTGCTTTTGACTTCTTTGATGGAGTCACAAATCCAGCTAGTCTACAGCCTGCAGATGGCCTAATCAGGCAGCCCAATGAAAAAGAGCTTGCTGCTCACTCGGTTGCTAAATATGGTGCCCTTGCCCGCCAGAAAATATCCACTGGTAACTACATCACCACCCTTGGTGAAGTTACACGTGGTCACATGGGCGGCGCCAACACTATGTACGCAATTGATGCACCTCCTGAACTTTAA

>PS5*_P8_c6

ATGGAAAGATCAACTTTGATCAATTTACTTCTGTTACACAAATTTGAACACAAGATTAACACTGAAGGAATCATTGTTGTGCACGGAATTGCTGGAACTGGGAAAACCACATTGCTTAGGACTTTATTTTCTGCTTACCCTAGCTTAGTTATAGGTTCACCTAGGCCTTGTTACTTAGATAAAGCTAATAAAATTTCACAAGTTTGCCTTTCTTGTTTTCCAAATACCTTGTGTGACATTGTTGACGAGTACCATCTCTTAGAAAGTTTTCCTGAACCAAAACTAGCCATTTTTGGTGACCCCTGTCAGTGCACTTACATTGAAAGGTTGAGAACACCCAACTACACATCCTTCAGAACACACCGATTTGGCAAATCCACTGCTGCTCTACTAAACAAGTTATTTGATCTTAACATTGAGTCAGTCAAAGCACAAGACGACACAGTAGAATACTTTGATCCTTTCGCAGTGGACCCCTCTGAACACATTTCTGCTTCAGAAAAAGAAGTTTTGGAATTTGTAGGTGATCAAGTTGAGACTACAAGCTCTGAAGAACTAGCTGGTCTCGAGTTTAGTGAAGTTACTTTCTACTGTACCACACTTGCTGGTGCTGTTCAAGAAAATCCTGCCAAAACCTTCATTTCACTCACTAGACACACTTCAAAGCTCACAATTGGTGAACTAAATGCCAGGTCTGACTCCTAGATGCCAGGTCTGACTCCTAGAGCTGATCTTACTGACACGTATAAAATCATTGCTATAGCCTTTCTACTGTCAGCTTGCATTTACTTCCAAAACAGTCATTATCAACCAGTCGCAGGTGATAATTTGCACAGACTACCCTTTGGTGGTCAGTATCAAGACGGAACTAAGAAGATCTCTTACTTTCCGCAGCAACAATCCTACTTTCACTCAGGAAACAAGCTTAATGTCCTCATACTTATCTTCATTCTTACACTGGGTATTGTCCTCACCAATAAATTTAGTTTTAGCATTAGCCGTAATACTCACCAGCATCATTGCTACAACACACATTCTGCAACCCAAACAGGTCAATCAGTGCCAGGTCATCATTGAATGTCCTCATACTTATCTTCATTCTTACACTGGGTATTGTCCTCACCAATAAATTTAGTTTTAGCATTAGCCGTAATACTCACCAGCATCATTGCTACAACACACATTCTGCAACCCAAACAGGTCAATCAGTGCCAGGTCATCATTGATGGTGCAGCCATAGTCATAACAAATTGTCCAAACACACCCGAAGTTCTTAAAGCAATCAACTTCTCCCCTTGGAACGGGTTAAGTTTTCCTCAATTGTGAATGGAAAACCAACCTACAGCTTCTAACCCATCAGATGTACCACCAACTGCTGCTCAAGCTGGTGCCCAGAGCCCAGCCGACTTCTCAAATCCTAATACAGCTCCTTCCCTAAGTGATTTGAAGAAGATCAAATACGTGTCAACTGTCACTTCAGTTGCCACGCCTGCTGAAATTGAGGCCCTTGGCAAGATCTTTACTGCCATGGGTTTAGCAGCCAATGAGACCGGACCTGCCATGTGGGACCTCGCTCGTGCTTATGCTGATGTGCAAAGTTCAAAATCTGCACAACTTATAGGTGCCACACCATCCAACCCTGCTTTGTCTAGACGTGCACTTGCTGCACAGTTTGATCGTATCAATATCACACCCAGACAATTCTGCATGTATTTTGCAAAAATTGTTTGGAACATACTGTTAGACAGCAATGTGCCACCTGCCAACTGGGCAAAATTGGGCTATCAGGAAGATACCAAGTTTGCTGCTTTTGACTTCTTCGATGGAGTCACAAATCCAGCTAGTCTACAGCCTGCAGATGGCCTAATCAGGCAGCCCAATGAAAAAGAGCTTGCTGCTCACTCGGTTGCTAAATATGGTGCCCTTGCCCGCCAGAAAATATCCACTGGTAACTACATCACCACCCTTGGTGAAGTTACACGTGGTCACATGGGCGGCGCCAACACTATGTACGCAATTGATGCACCTCCTGAACTTTAA

>PS5*_P8_c5

ATGGAAAGATCAACTTTGATCAATTTACTTCTGTTACACAAATTTGAACACAAGATTAACACTGAAGGAATCATTGTTGTGCACGGAATTGCTGGAACTGGGAAAACCACATTGCTTAGGACTTTATTTTCTGCTTACCCTAGCTTAGTTATAGGTTCACCTAGGCCTTGTTACTTAGATAAAGCTAATAAAATTTCACAAGTTTGCCTTTCTTGTTTTCCAAATACCTTGTGTGACATTGTTGACGAGTACCATCTCTTAGAAAGTTTTCCTGAACCAAAACTAGCCATTTTTGGTGACCCCTGTCAGTGCACTTACATTGAAAGGTTGAGAACACCCAACTACACATCCTTCAGAACACACCGATTTGGCAAATCCACTGCTGCTCTACTAAACAAGTTATTTGATCTTAACATTGAGTCAGTCAAAGCACAAGACGACACAGTAGAATACTTTGATCCTTTCGCAGTGGACCCCTCTGAACACATTTCTGCTTCAGAAAAAGAAGTTTTGGAATTTGTAGGTGATCAAGTTGAGACTACAAGCTCTGAAGAACTAGCTGGTCTCGAGTTTAGTGAAGTTACTTTCTACTGTACCACACTTGCTGGTGCTGTTCAAGAAAATCCTGCCAAAACCTTCATTTCACTCACTAGACACACTTCAAAGCTCACAATTGGTGAACTAAATGCCAGGTCTGACTCCTAGATGCCAGGTCTGACTCCTAGAGCTGATCTTACTGACACGTATAAAATCATTGCTATAGCCTTTCTACTGTCAGCTTGCATTTACTTCCAAAACAGTCATTATCAACCAGTCGCAGGTGATAATTTGCACAGACTACCCTTTGGTGGTCAGTATCAAGACGGAACTAAGAAGATCTCTTACTTTCCGCAGCAACAATCCTACTTTCACTCAGGAAACAAGCTTAATGTCCTCATACTTATCTTCATTCTTACACTGGGTATTGTCCTCACCAATAAATTTAGTTTTAGCATTAGCCGTAATACTCACCAGCATCATTGCTACAACACACATTCTGCAACCCAAACAGGTCAATCAGTGCCAGGTCATCATTGAATGTCCTCATACTTATCTTCATTCTTACACTGGGTATTGTCCTCACCAATAAATTTAGTTTTAGCATTAGCCGTAATACTCACCAGCATCATTGCTACAACACACATTCTGCAACCCAAACAGGTCAATCAGTGCCAGGTCATCATTGATGGTGCAGCCATAGTCATAACAAATTGTCCAAACACACCCGAAGTTCTTAAAGCAATCAACTTCTCCCCTTGGAACGGGTTAAGTTTTCCTCAATTGTGAATGGAAAACCAACCTACAGCTTCTAACCCATCAGATGTACCACCAACTGCTGCTCAAGCTGGTGCCCAGAGCCCAGCCGACTTCTCAAATCCTAATACAGCTCCTTCCCTAAGTGATTTGAAGAAGATCAAATACGTGTCAACTGTCACTTCAGTTGCCACGCCTGCTGAAATTGAGGCCCTTGGCAAGATCTTTACTGCCATGGGTTTAGCAGCCAATGAGACCGGACCTGCCATGTGGGACCTCGCTCGTGCTTATGCTGATGTGCAAAGTTCAAAATCTGCACAACTTATAGGTGCCACACCATCCAACCCTGCTTTGTCTAGACGTGCACTTGCTGCACAGTTTGATCGTATCAATATCACACCCAGACAATTCTGCATGTATTTTGCAAAAATTGTTTGGAACATACTGTTAGACAGCAATGTGCCACCTGCCAACTGGGCAAAATTGGGCTATCAGGAAGATACCAAGTTTGCTGCTTTTGACTTCTTTGATGGAGTCACAAATCCAGCTAGTCTACAGCCTGCAGATGGCCTAATCAGGCAGCCCAATGAAAAAGAGCTTGCTGCTCACTCGGTTGCTAAATATGGTGCCCTTGCCCGCCAGAAAATATCCACTGGTAACTACATCACCGCCCTTGGTGAAGTTACACGTGGTCACATGGGCGGCGCCAACACTATGTACGCAATTGATGCACCTCCTGAACTTTAA

>PS5*_P8_c4

ATGGAAAGATCAACTTTGATCAATTTACTTCTGTTACACAAATTTGAACACAAGATTAACACTGAAGGAATCATTGTTGTGCACGGAATTGCTGGAACTGGGAAAACCACATTGCTTAGGACTTTATTTTCTGCTTACCCTAGCTTAGTTATAGGTTCACCTAGGCCTTGTTACTTAGATAAAGCTAATAAAATTTCACAAGTTTGCCTTTCTTGTTTTCCAAATACCTTGTGTGACATTGTTGACGAGTACCATCTCTTAGAAAGTTTTCCTGAACCAAAACTAGCCATTTTTGGTGACCCCTGTCAGTGCACTTACATTGAAAGGTTGAGAACACCCAACTACACATCCTTCAGAACACACCGATTTGGCAAATCCACTGCTGCTCTACTAAACAAGTTATTTGATCTTAACATTGAGTCAGTCAAAGCACAAGACGACACAGTAGAATACTTTGATCCTTTCGCAGTGGACCCCTCTGAACACATTTCTGCTTCAGAAAAAGAAGTTTTGGAATTTGTAGGTGATCAAGTTGAGACTACAAGCTCTGAAGAACTAGCTGGTCTCGAGTTTAGTGAAGTTACTTTCTACTGTACCACACTTGCTGGTGCTGTTCAAGAAAATCCTGCCAAAACCTTCATTTCACTCACTAGACACACTTCAAAGCTCACAATTGGTGAACTAAATGCCAGGTCTGACTCCTAGATGCCAGGTCTGACTCCTAGAGCTGATCTTACTGACACGTATAAAATCATTGCTATAGCCTTTCTACTGTCAGCTTGCATTTACTTCCAAAACAGTCATTATCAACCAGTCGCAGGTGATAATTTGCACAGACTACCCTTTGGTGGTCAGTATCAAGACGGAACTAAGAAGATCTCTTACTTTCCGCAGCAACAATCCTACTTTCACTCAGGAAACAAGCTTAATGTCCTCATACTTATCTTCATTCTTACACTGGGTATTGTCCTCACCAATAAATTTAGTTTTAGCATTAGCCGTAATACTCACCAGCATCATTGCTACAACACACATTCTGCAACCCAAACAGGTCAATCAGTGCCAGGTCATCATTGAATGTCCTCATACTTATCTTCATTCTTACACTGGGTATTGTCCTCACCAATAAATTTAGTTTTAGCATTAGCCGTAATACTCACCAGCATCATTGCTACAACACACATTCTGCAACCCAAACAGGTCAATCAGTGCCAGGTCATCATTGATGGTGCAGCCATAGTCATAACAAATTGTCCAAACACACCCGAAGTCCTTAAAGCAATCAACTTCTCCCCTTGGAACGGGTTAAGTTTTCCTCAATTGTGAATGGAAAACCAACCTACAGCTTCTAACCCATCAGATGTACCACCAACTGCTGCTCAAGCTGGTGCCCAGAGCCCAGCCGACTTCTCAAATCCTAATACAGCTCCTTCCCTAAGTGATTTGAAGAAGATCAAATACGTGTCAACTGTCACTTCAGTTGCCACGCCTGCTGAAATTGAGGCCCTTGGCAAGATCTTTACTGCCATGGGTTTAGCAGCCAATGAGACCGGACCTGCCATGTGGGACCTCGCTCGTGCTTATGCTGATGTGCAAAGTTCAAAATCTGCACAACTTATAGGTGCCACACCATCCAACCCTGCTTTGTCTAGACGTGCACTTGCTGCACAGTTTGATCGTATCAATATCACACCCAGACAATTCTGCATGTATTTTGCAAAAATTGTTTGGAACATACTGTTAGACAGCAATGTGCCACCTGCCAACTGGGCAAAATTGGGCTATCAGGAAGATACCAAGTTTGCTGCTTTTGACTTCTTTGATGGAGTCACAAATCCAGCTAGTCTACAGCCTGCAGATGGCCTAATCAGGCAGCCCAATGAAAAAGAGCTTGCTGCTCACTCGGTTGCTAAATATGGTGCCCTTGCCCGCCAGAAAATATCCACTGGTAACTACATCACCACCCTTGGTGAAGTTACACGTGGTCACATGGGCGGCGCCAACACTATGTACGCAATTGATGCACCTCCTGAACTTTAA

>PS5*_P8_c3

ATGGAAAGATCAACTTTGATCAATTTACTTCTGTTACACAAATTTGAACACAAGATTAACACTGAAGGAATCATTGTTGTGCACGGAATTGCTGGAACTGGGAAAACCACATTGCTTAGGACCTTATTTTCTGCTTACCCTAGCTTAGTTATAGGTTCACCTAGGCCTTGTTACTTAGATAAAGCTAATAAAATTTCACAAGTTTGCCTTTCTTGTTTTCCAAATACCTTGTGTGACATTGTTGACGAGTACCATCTCTTAGAAAGTTTTCCTGAACCAAAACTAGCCATTTTTGGTGACCCCTGTCAGTGCACCTACATTGAAAGGTTGAGAACACCCAACTACACATCCTTCAGAACACACCGATTTGGCAAATCCACTGCTGCTCTACTAAACAAGTTATTTGATCTTAACATTGAGTCAGTCAAAGCACAAGACGACACAGTAGAATACTTTGATCCTTTCGCAGTGGACCCCTCTGAACACATTTCTGCTTCAGAAAAAGAAGTTTTGGAATTTGTAGGTGATCAAGTTGAGACTACAAGCTCTGAAGAACTAGCTGGTCTCGAGTTTAGTGAAGTTACTTTCTACTGTACCACACTTGCTGGTGCTGTTCAAGAAAATCCTGCCAAAACCTTCATTTCACTCACTAGACACACTTCAAAGCTCACAATTGGTGAACTAAATGCCAGGTCTGACTCCTAGATGCCAGGTCTGACTCCTAGAGCTGATCTTACTGACACGTATAAAATCATTGCTATAGCCTTTCTACTGTCAGCTTGCATTTACTTCCAAAACAGTCATTATCAACCAGTCGCAGGTGATAATTTGCACAGACTACCCTTTGGTGGTCAGTATCAAGACGGAACTAAGAAGATCTCTTACTTTCCGCAGCAACAATCCTACTTTCACTCAGGAAACAAGCTTAATGTCCTCATACTTATCTTCATTCTTACACTGGGTATTGTCCTCACCAATAAATTTAGTTTTAGCATTAGCCGTAATACTCACCAGCATCATTGCTACAACACACATTCTGCAACCCAAACAGGTCAATCAGTGCCAGGTCATCATTGAATGTCCTCATACTTATCTTCATTCTTACACTGGGTATTGTCCTCACCAATAAATTTAGTTTTAGCATTAGCCGTAATACTCACCAGCATCATTGCTACAACACACATTCTGCAACCCAAACAGGTCAATCAGTGCCAGGTCATCATTGATGGTGCAGCCATAGTCATAACAAATTGTCCAAACACACCCGAAGTTCTTAAAGCAATCAACTTCTCCCCTTGGAACGGGTTAAGTTTTCCTCAATTGTGAATGGAAAACCAACCTACAGCTTCTAACCCATCAGATGTACCACCAACTGCTGCTCAAGCTGGTGCCCAGAGCCCAGCCGACTTCTCAAATCCTAATACAGCTCCTTCCCTAAGTGATTTGAAGAAGATCAAATACGTGTCAACTGTCACTTCAGTTGCCACGCCTGCTGAAATTGAGGCCCTTGGCAAGATCTTTACTGCCATGGGTTTAGCAGCCAATGAGACCGGACCTGCCATGTGGGACCTCGCTCGTGCTTATGCTGATGTGCAAAGTTCAAAATCTGCACAACTTATAGGTGCCACACCATCCAACCCTGCTTTGTCTAGACGTGCACTTGCTGCACAGTTTGATCGTATCAATATCACACCCAGACAATTCTGCATGTATTTTGCAAAAATTGTTTGGAACATACTGTTAGACAGCAATGTGCCACCTGCCAACTGGGCAAAATTGGGCTATCAGGAAGATACCAAGTTTGCTGCTTTTGACTTCTTTGATGGAGTCACAAATCCAGCTAGTCTACAGCCTGCAGATGGCCTAATCAGGCAGCCCAATGAAAAAGAGCTTGCTGCTCACTCGGTTGCTAAATATGGTGCCCTTGCCCGCCAGAAAATATCCACTGGTAACTACATCACCACCCTTGGTGAAGTTACACGTGGTCACATGGGCGGCGCCAACACTATGTACGCAATTGATGCACCTCCTGAACTTTAA

>PS5*_P8_c2

ATGGAAAGATCAACTTTGATCAATTTACTTCTGTTACACAAATTTGAACACAAGATTAACACTGAAGGAATCATTGTTGTGCACGGAATTGCTGGAACTGGGAAAACCACATTGCTTAGGACTTTATTTTCTGCTTACCCTAGCTTAGTTATAGGTTCACCTAGGCCTTGTTACTTAGATAAAGCTAATAAAATTTCACAAGTTTGCCTTTCTTGTTTTCCAAATACCTTGTGTGACATTGTTGACGAGTACCATCTCTTAGAAAGTTTTCCTGAACCAAAACTAGCCATTTTTGGTGACCCCTGTCAGTGCACTTACATTGAAAGGTTGAGAACACCCAACTACACATCCTTCAGAACACACCGATTTGGCAAATCCACTGCTGCTCTACTAAACAAGTTATTTGATCTTAACATTGAGTCAGTCAAAGCACAAGACGACACAGTAGAATACTTTGATCCTTTCGCAGTGGACCCCTCTGAACACATTTCTGCTTCAGAAAAAGAAGTTTTGGAATTTGTAGGTGATCAAGTTGAGACTACAAGCTCTGAAGAACTAGCTGGTCTCGAGTTTAGTGAAGTTACTTTCTACTGTACCACACTTGCTGGTGCTGTTCAAGAAAATCCTGCCAAAACCTTCATTTCACTCACTAGACACACTTCAAAGCTCACAATTGGTGAACTAAATGCCAGGTCTGACTCCTAGATGCCAGGTCTGACTCCTAGAGCTGATCTTACTGACACGTATAAAATCATTGCTATAGCCTTTCTACTGTCAGCTTGCATTTACTTCCAAAACAGTCATTATCAACCAGTCGCAGGTGATAATTTGCACAGACTACCCTTTGGTGGTCAGTATCAAGACGGAACTAAGAAGATCTCTTACTTTCCGCAGCAACAATCCTACTTTCACTCAGGAAACAAGCTTAATGTCCTCATACTTATCTTCATTCTTACACTGGGTATTGTCCTCACCAATAAATTTAGTTTTAGCATTAGCCGTAATACTCACCAGCATCATTGCTACAACACACATTCTGCAACCCAAACAGGTCAATCAGTGCCAGGTCATCATTGAATGTCCTCATACTTATCTTCATTCTTACACTGGGTATTGTCCTCACCAATAAATTTAGTTTTAGCATTAGCCGTAATACTCACCAGCATCATTGCTACAACACACATTCTGCAACCCAAACAGGTCAATCAGTGCCAGGTCATCATTGATGGTGCAGCCATAGTCATAACAAATTGTCCAAACACACCCGAAGTTCTTAAAGCAATCAACTTCTCCCCTTGGAACGGGTTAAGTTTTCCTCAATTGTGAATGGAAAACCAACCTACAGCTTCTAACCCATCAGATGTACCACCAACTGCTGCTCAAGCTGGTGCCCAGAGCCCAGCCGACTTCTCAAATCCTAATACAGCTCCTTCCCTAAGTGATTTGAAGAAGATCAAATACGTGTCAACTGTCACTTCAGTTGCCACGCCTGCTGAAATTGAGGCCCTTGGCAAGATCTTTACTGCCATGGGTTTAGCAGCCAATGAGACCGGACCTGCCATGTGGGACCTCGCCCGTGCTTATGCTGATGTGCAAAGTTCAAAATCTGCACAACTTATAGGTGCCACACCATCCAACCCTGCTTTGTCTAGACGTGCACTTGCTGCACAGTTTGATCGTATCAATATCACACCCAGACAATTCTGCATGTATTTTGCAAAAATTGTTTGGAACATACTGTTAGACAGCAATGTGCCACCTGCCAACTGGGCAAAATTGGGCTATCAGGAAGATACCAAGTTTGCTGCCTTTGACTTCTTTGATGGAGTCACAAATCCAGCTAGTCTACAGCCTGCAGATGGCCTAATCAGGCAGCCCAATGAAAAAGAGCTTGCTGCTCACTCGGTTGCTAAATATGGTGCCCTTGCCCGCCAGAAAATATCCACTGGTAACTACATCACCACCCTTGGTGAAGTTACACGTGGTCACATGGGCGGCGCCAACACTATGTACGCAATTGATGCACCTCCTGAACTTTAA

>PS5*_P8_c1

ATGGAAAGATCAACTTTGATCAATTTACTTCTGTTACACAAATTTGAACACAAGATTAACACTGAAGGAATCATTGTTGTGCACGGAATTGCTGGAACTGGGAAAACCACATTGCTTAGGACTTTATTTTCTGCTTACCCTAGCTTAGTTATAGGTTCACCTAGGCCTTGTTACTTAGATAAAGCTAATAAAATTTCACAAGTTTGCCTTTCTTGTTTTCCAAATACCTTGTGTGACATTGTTGACGAGTACCATCTCTTAGAAAGTTTTCCTGAACCAAAACTAGCCATTTTTGGTGACCCCTGTCAGTGCACTTACATTGAAAGGTTGAGAACACCCAACTACACATCCTTCAGAACACACCGATTTGGCAAATCCACTGCTGCTCTACTAAACAAGTTATTTGATCTTAACATTGAGTCAGTCAAAGCACAAGACGACACAGTAGAATACTTTGATCCTTTCGCAGTGGACCCCTCTGAACACATTTCTGCTTCAGAAAAAGAAGTTTTGGAATTTGTAGGTGATCAAGTTGAGACTACAAGCTCTGAAGAACTAGCTGGTCTCGAGTTTAGTGAAGTTACTTTCTACTGTACCACACTTGCTGGTGCTGTTCAAGAAAATCCTGCCAAAACCTTCATTTCACTCACTAGACACACTTCAAAGCTCACAATTGGTGAACTAAATGCCAGGTCTGACTCCTAGATGCCAGGTCTGACTCCTAGAGCTGATCTTACTGACACGTATAAAATCATTGCTATAGCCTTTCTACTGTCAGCTTGCATTTACTTCCAAAACAGTCATTATCAACCAGTCGCAGGTGATAATTTGCACAGACTACCCTTTGGTGGTCAGTATCAAGACGGAACTAAGAAGATCTCTTACTTTCCGCAGCAACAATCCTACTTTCACTCAGGAAACAAGCTTAATGTCCTCATACTTATCTTCATTCTTACACTGGGTATTGTCCTCACCAATAAATTTAGTTTTAGCATTAGCCGTAATACTCACCAGCATCATTGCTACAACACACATTCTGCAACCCAAACAGGTCAATCAGTGCCAGGTCATCATTGAATGTCCTCATACTTATCTTCATTCTTACACTGGGTATTGTCCTCACCAATAAATTTAGTTTTAGCATTAGCCGTAATACTCACCAGCATCATTGCTACAACACACATTCTGCAACCCAAACAGGTCAATCAGTGCCAGGTCATCATTGATGGTGCAGCCATAGTCATAACAAATTGTCCAAACACACCCGAAGTTCTTAAAGCAATCAACTTCTCCCCTTGGAACGGGTTAAGTTTTCCTCAATTGTGAATGGAAAACCAACCTACAGCTTCTAACCCATCAGATGTACCACCAACTGCTGCTCAAGCTGGTGCCCAGAGCCCAGCCGACTTCTCAAATCCTAATACAGCTCCTTCCCTAAGTGATTTGAAGAAGATCAAATACGTGTCAACTGTCACTTCAGTTGCCACGCCTGCTGAAATTGAGGCCCTTGGCAAGATCTTTACTGCCATGGGTTTAGCAGCCAATGAGACCGGACCTGCCATGTGGGACCTCGCTCGTGCTTATGCTGATGTGCAAAGTTCAAAATCTGCACAACTTATAGGTGCCACACCATCCAACCCTGCTTTGTCTAGACGTGCACTTGCTGCACAGTTTGATCGTATCAATATCACACCCAGACAATTCTGCATGTATTTTGCAAAAATTGTTTGGAACATACTGTTAGACAGCAATGTGCCACCTGCCAACTGGGCAAAATTGGGCTATCAGGAAGATACCAAGTTTGCTGCTTTTGACTTCTTTGATGGAGTCACAAATCCAGCTAGTCTACAGCCTGCAGATGGCCTAATCAGGCAGCCCAATGAAAAAGAGCTTGCTGCTCACTCGGTTGCTAAATATGGTGCCCTTGCCCGCCAGAAAATATCCACTGGTAACTACATCACCACCCTTGGTGAAGTTACACGTGGTCACATGGGCGGCGCCAACACTATGTACGCAATTGATGCACCTCCTGAACTTTAA

>PS5*_P8_b14

ATGGAAAGATCAACTTTGATCAATTTACTTCTGTTACACAAATTTGAACACAAGATTAACACTGAAGGAATCATTGTTGTGCACGGAATTGCTGGAACTGGGAAAACCACATTGCTTAGGACTTTATTTTCTGCTTACCCTAGCTTAGTTATAGGTTCACCTAGGCCTTGTTACTTAGATAAAGCTAATAAAATTTCACAAGTTTGCCTTTCTTGTTTTCCAAATACCTTGTGTGACATTGTTGACGAGTACCATCTCTTAGAAAGTTTTCCTGAACCAAAACTAGCCATTTTTGGTGACCCCTGTCAGTGCACTTACATTGAAAGGTTGAGAACACCCAACTACACATCCTTCAGAACACACCGATTTGGCAAATCCACTGCTGCTCTACTAAACAAGTTATTTGATCTTAACATTGAGTCAGTCAAAGCACAAGACGACACAGTAGAATACTTTGATCCTTTCGCAGTGGACCCCTCTGAACACATTTCTGCTTCAGAAAAAGAAGTTTTGGAATTTGTAGGTGATCAAGTTGAGACTACAAGCTCTGAAGAACTAGCTGGTCTCGAGTTTAGTGAAGTTACTTTCTACTGTACCACACTTGCTGGTGCTGTTCAAGAAAATCCTGCCAAAACCTTCATTTCACTCACTAGACACACTTCAAAGCTCACAATTGGTGAACTAAATGCCAGGTCTGACTCCTAGATGCCAGGTCTGACTCCTAGAGCTGATCTTACTGACACGTATAAAATCATTGCTATAGCCTTTCTACTGTCAGCTTGCATTTACTTCCAAAACAGTCATTATCAACCAGTTGCAGGTGATAATTTGCACAGACTACCCTTTGGTGGTCAGTATCAAGACGGAACTAAGAAGATCTCTTACTTTCCGCAGCAACAATCCTACTTTCACTCAGGAAACAAGCTTAATGTCCTCATACTTATCTTCATTCTTACACTGGGTATTGTCCTCACCAATAAATTTAGTTTTAGCATTAGCCGTAATACTCACCAGCATCATTGCTACAACACACATTCTGCAACCCAAACAGGTCAATCAGTGCCAGGTCATCATTGAATGTCCTCATACTTATCTTCATTCTTACACTGGGTATTGTCCTCACCAATAAATTTAGTTTTAGCATTAGCCGTAATACTCACCAGCATCATTGCTACAACACACATTCTGCAACCCAAACAGGTCAATCAGTGCCAGGTCATCATTGATGGTGCAGCCATAGTCATAACAAATTGTCCAAACACACCCGAAGTTCTTAAAGCAATCAACTTCTCCCCTTGGAACGGGTTAAGTTTTCCTCAATTGTGAATGGAAAACCAACCTACAGCTTCTAACCCATCAGATGTACCACCAACTGCTGCTCAAGCTGGTGCCCAGAGCCCAGCCGACTTCTCAAATCCTAATACAGCTCCTTCCCTAAGTGATTTGAAGAAGATCAAATACGTGTCAACTGTCACTTCAGTTGCCACGCCTGCTGAAATTGAGGCCCTTGGCAAGATCTTTACTGCCATGGGTTTAGCAGCCAATGAGACCGGACCTGCCATGTGGGACCTCGCTCGTGCTTATGCTGATGTGCAAAGTTCAAAATCTGCACAACTTATAGGTGCCACACCATCCAACCCTGCTTTGTCTAGACGTGCACTTGCTGCACAGTTTGATCGTATCAATATCACACCCAGACAATTCTGCATGTATTTTGCAAAAATTGTTTGGAACATACTGTTAGACAGCAATGTGCCACCTGCCAACTGGGCAAAATTGGGCTATCAGGAAGATACCAAGTTTGCTGCTTTTGACTTCTTTGATGGAGTCACAAATCCAGCTAGTCTACAGCCTGCAGATGGCCTAATCAGGCAGCCCAATGAAAAAGAGCTTGCTGCTCACTCGGTTGCTAAATATGGTGCCCTTGCCCGCCAGAAAATATCCACTGGTAACTACATCACCACCCTTGGTGAAGTTACACGTGGTCACATGGGCGGCGCCAACACTATGTACGCAATTGATGCACCTCCTGAACTTTAA

>PS5*_P8_b13

ATGGAAAGATCAACTTTGATCAATTTACTTCTGTTACACAAATTTGAACACAAGATTAACACTGAAGGAATCATTGTTGTGCACGGAATTGCTGGAACTGGGAAAACCACATTGCTTAGGACTTTATTTTCTGCTTACCCTAGCTTAGTTATAGGTTCACCTAGGCCTTGTTACTTAGATAAAGCTAATAAAATTTCACAAGTTTGCCTTTCTTGTTTTCCAAATACCTTGTGTGACATTGTTGACGAGTACCATCTCTTAGAAAGTTTTCCTGAACCAAAACTAGCCATTTTTGGTGACCCCTGTCAGTGCACTTACATTGAAAGGTTGAGAACACCCAACTACACATCCTTCAGAACACACCGATTTGGCAAATCCACTGCTGCTCTACTAAACAAGTTATTTGATCTTAACATTGAGTCAGTCAAAGCACAAGACGACACAGTAGAATACTTTGATCCTTTCGCAGTGGACCCCTCTGAACACATTTCTGCTTCAGAAAAAGAAGTTTTGGAATTTGTAGGTGATCAAGTTGAGACTACAAGCTCTGAAGAACTAGCTGGTCTCGAGTTTAGTGAAGTTACTTTCTACTGTACCACACTTGCTGGTGCTGTTCAAGAAAATCCTGCCAAAACCTTCATTTCACTCACTAGACACACTTCAAAGCTCACAATTGGTGAACTAAATGCCAGGTCTGACTCCTAGATGCCAGGTCTGACTCCTAGAGCTGATCTTACTGACACGTATAAAATCATTGCTATAGCCTTTCTACTGTCAGCTTGCATTTACTTCCAAAACAGTCATTATCAACCAGTTGCAGGTGATAATTTGCACAGACTACCCTTTGGTGGTCAGTATCAAGACGGAACTAAGAAGATCTCTTACTTTCCGCAGCAACAATCCTACTTTCACTCAGGAAACAAGCTTAATGTCCTCATACTTATCTTCATTCTTACACTGGGTATTGTCCTCACCAATAAATTTAGTTTTAGCATTAGCCGTAATACTCACCAGCATCATTGCTACAACACACATTCTGCAACCCAAACAGGTCAATCAGTGCCAGGTCATCATTGAATGTCCTCATACTTATCTTCATTCTTACACTGGGTATTGTCCTCACCAATAAATTTAGTTTTAGCATTAGCCGTAATACTCACCAGCATCATTGCTACAACACACATTCTGCAACCCAAACAGGTCAATCAGTGCCAGGTCATCATTGATGGTGCAGCCATAGTCATAACAAATTGTCCAAACACACCCGAAGTTCTTAAAGCAATCAACTTCTCCCCTTGGAACGGGTTAAGTTTTCCTCAATTGTGAATGGAAAACCAACCTACAGCTTCTAACCCATCAGATGTACCACCAACTGCTGCTCAAGCTGGTGCCCAGAGCCCAGCCGACTTCTCAAATCCTAATACAGCTCCTTCCCTAAGTGATTTGAAGAAGATCAAATACGTGTCAACTGTCACTTCAGTTGCCACGCCTGCTGAAATTGAGGCCCTTGGCAAGATCTTTACTGCCATGGGTTTAGCAGCCAATGAGACCGGACCTGCCATGTGGGACCTCGCTCGTGCTTATGCTGATGTGCAAAGTTCAAAATCTGCACAACTTATAGGTGCCACACCATCCAACCCTGCTTTGTCTAGACGTGCACTTGCTGCACAGTTTGATCGTATCAATATCACACCCAGACAATTCTGCATGTATTTTGCAAAAATTGTTTGGAACATACTGTTAGACAGCAATGTGCCACCTGCCAACTGGGCAAAATTGGGCTATCAGGAAGATACCAAGTTTGCTGCTTTTGACTTCTTTGATGGAGTCACAAATCCAGCTAGTCTACAGCCTGCAGATGGCCTAATCAGGCAGCCCAATGAAAAAGAGCTTGCTGCTCACTCGGTTGCTAAATATGGTGCCCTTGCCCGCCAGAAAATATCCACTGGTAACTACATCACCACCCTTGGTGAAGTTACACGTGGTCACATGGGCGGCGCCAACACTATGTACGCAATTGATGCACCTCCTGAACTTTAA

>PS5*_P8_b12

ATGGAAAGATCAACTTTGATCAATTTACTTCTGTTACACAAATTTGAACACAAGATTAACACTGAAGGAATCATTGTTGTGCACGGAATTGCTGGAACTGGGAAAACCACATTGCTTAGGACTTTATTTTCTGCTTACCCTAGCTTAGTTATAGGTTCACCTAGGCCTTGTTACTTAGATAAAGCTAATAAAATTTCACAAGTTTGCCTTTCTTGTTTTCCAAATACCTTGTGTGACATTGTTGACGAGTACCATCTCTTAGAAAGTTTTCCTGAACCAAAACTAGCCATTTTTGGTGACCCCTGTCAGTGCACTTACATTGAAAGGTTGAGAACACCCAACTACACATCCTTCAGAACACACCGATTTGGCAAATCCACTGCTGCTCTACTAAACAAGTTATTTGATCTTAACATTGAGTCAGTCAAAGCACAAGACGACACAGTAGAATACTTTGATCCTTTCGCAGTGGACCCCTCTGAACACATTTCTGCTTCAGAAAAAGAAGTTTTGGAATTTGTAGGTGATCAAGTTGAGACTACAAGCTCTGAAGAACTAGCTGGTCTCGAGTTTAGTGAAGTTACTTTCTACTGTACCACACTTGCTGGTGCTGTTCAAGAAAATCCTGCCAAAACCTTCATTTCACTCACTAGACACACTTCAAAGCTCACAATTGGTGAACTAAATGCCAGGTCTGACTCCTAGATGCCAGGTCTGACTCCTAGAGCTGATCTTACTGACACGTATAAAATCATTGCTATAGCCTTTCTACTGTCAGCTTGCATTTACTTCCAAAACAGTCATTATCAACCAGTTGCAGGTGATAATTTGCACAGACTACCCTTTGGTGGTCAGTATCAAGACGGAACTAAGAAGATCTCTTACTTTCCGCAGCAACAATCCTACTTTCACTCAGGAAACAAGCTTAATGTCCTCATACTTATCTTCATTCTTACACTGGGTATTGTCCTCACCAATAAATTTAGTTTTAGCATTAGCCGTAATACTCACCAGCATCATTGCTACAACACACATTCTGCAACCCAAACAGGTCAATCAGTGCCAGGTCATCATTGAATGTCCTCATACTTATCTTCATTCTTACACTGGGTATTGTCCTCACCAATAAATTTAGTTTTAGCATTAGCCGTAATACTCACCAGCATCATTGCTACAACACACATTCTGCAACCCAAACAGGTCAATCAGTGCCAGGTCATCATTGATGGTGCAGCCATAGTCATAACAAATTGTCCAAACACACCCGAAGTTCTTAAAGCAATCAACTTCTCCCCTTGGAACGGGTTAAGTTTTCCTCAATTGTGAATGGAAAACCAACCTACAGCTTCTAACCCATCAGATGTACCACCAACTGCTGCTCAAGCTGGTGCCCAGAGCCCAGCCGACTTCTCAAATCCTAGTACAGCTCCTTCCCTAAGTGATTTGAAGAAGATCAAATACGTGTCAACTGTCACTTCAGTTGCCACGCCTGCTGAAATTGAGGCCCTTGGCAAGATCTTTACTGCCATGGGTTTAGCAGCCAATGAGACCGGACCTGCCATGTGGGACCTCGCTCGTGCTTATGCTGATGTGCAAAGTTCAAAATCTGCACAACTTATAGGTGCCACACCATCCAACCCTGCTTTGTCTAGACGTGCACTTGCTGCACAGTTTGATCGTATCAATATCACACCCAGACAATTCTGCATGTATTTTGCAAAAATTGTTTGGAACATACTGTTAGACAGCAATGTGCCACCTGCCAACTGGGCAAAATTGGGCTATCAGGAAGATACCAAGTTTGCTGCTTTTGACTTCTTTGATGGAGTCACAAATCCAGCTAGTCTACAGCCTGCAGATGGCCTAATCAGGCAGCCCAATGAAAAAGAGCTTGCTGCTCACTCGGTTGCTAAATATGGTGCCCTTGCCCGCCAGAAAATATCCACTGGTAACTACATCACCACCCTTGGTGAAGTTACACGTGGTCACATGGGCGGCGCCAACACTATGTACGCAATTGATGCACCTCCTGAACTTTAA

>PS5*_P8_b11

ATGGAAAGATCAACTTTGATCAATTTACTTCTGTTACACAAATTTGAACACAAGATTAACACTGAAGGAATCATTGTTGTGCACGGAATTGCTGGAACTGGGAAAACCACATTGCTTAGGACTTTATTTTCTGCTTACCCTAGCTTAGTTATAGGTTCACCTAGGCCTTGTTACTTAGATAAAGCTAATAAAATTTCACAAGTTTGCCTTTCTTGTTTTCCAAATACCTTGTGTGACATTGTTGACGAGTACCATCTCTTAGAAAGTTTTCCTGAACCAAAACTAGCCATTTTTGGTGACCCCTGTCAGTGCACTTACATTGAAAGGTTGAGAACACCCAACTACACATCCTTCAGAACACACCGATTTGGCAAATCCACTGCTGCTCTACTAAACAAGTTATTTGATCTTAACATTGAGTCAGTCAAAGCACAAGACGACACAGTAGAATACTTTGATCCTTTCGCAGTGGACCCCTCTGAACACATTTCTGCTTCAGAAAAAGAAGTTTTGGAATTTGTAGGTGATCAAGTTGAGACTACAAGCTCTGAAGAACTAGCTGGTCTCGAGTTTAGTGAAGTTACTTTCTACTGTACCACACTTGCTGGTGCTGTTCAAGAAAATCCTGCCAAAACCTTCATTTCACTCACTAGACACACTTCAAAGCTCACAATTGGTGAACTAAATGCCAGGTCTGACTCCTAGATGCCAGGTCTGACTCCTAGAGCTGATCTTACTGACACGTATAAAATCATTGCTATAGCCTTTCTACTGTCAGCTTGCATTTACTTCCAAAACAGTCATTATCAACCAGTTGCAGGTGATAATTTGCACAGACTACCCTTTGGTGGTCAGTATCAAGACGGAACTAAGAAGATCTCTTACTTTCCGCAGCAACAATCCTACTTTCACTCAGGAAACAAGCTTAATGTCCTCATACTTATCTTCATTCTTACACTGGGTATTGTCCTCACCAATAAATTTAGTTTTAGCATTAGCCGTAATACTCACCAGCATCATTGCTACAACACACATTCTGCAACCCAAACAGGTCAATCAGTGCCAGGTCATCATTGAATGTCCTCATACTTATCTTCATTCTTACACTGGGTATTGTCCTCACCAATAAATTTAGTTTTAGCATTAGCCGTAATACTCACCAGCATCATTGCTACAACACACATTCTGCAACCCAAACAGGTCAATCAGTGCCAGGTCATCATTGATGGTGCAGCCATAGTCATAACAAATTGTCCAAACACACCCGAAGTTCTTAAAGCAATCAACTTCTCCCCTTGGAACGGGTTAAGTTTTCCTCAATTGTGAATGGAAAACCAACCTACAGCTTCTAACCCATCAGATGTACCACCAACTGCTGCTCAAGCTGGTGCCCAGAGCCCAGCCGACTTCTCAAATCCTAATACAGCTCCTTCCCTAAGTGATTTGAAGAAGATCAAATACGTGTCAACTGTCACTTCAGTTGCCACGCCTGCTGAAATTGAGGCCCTTGGCAAGATCTTTACTGCCATGGGTTTAGCAGCCAATGAGACCGGACCTGCCATGTGGGACCTCGCTCGTGCTTATGCTGATGTGCAAAGTTCAAAATCTGCACAACTTATAGGTGCCACACCATCCAACCCTGCTTTGTCTAGACGTGCACTTGCTGCACAGTTTGATCGTATCAATATCACACCCAGACAATTCTGCATGTATTTTGCAAAAATTGTTTGGAACATACTGTTAGACAGCAATGTGCCACCTGCCAACTGGGCAAAATTGGGCTATCAGGAAGATACCAAGTTTGCTGCTTTTGACTTCTTTGATGGAGTCACAAATCCAGCTAGTCTACAGCCTGCAGATGGCCTAATCAGGCAGCCCAATGAAAAAGAGCTTGCTGCTCACTCGGTTGCTAAATATGGTGCCCTTGCCCGCCAGAAAATATCCACTGGTAACTACATCACCACCCTTGGTGAAGTTACACGTGGTCACATGGGCGGCGCCAACACTATGTACGCAATTGATGCACCTCCTGAACTTTAA

>PS5*_P8_b10

ATGGAAAGATCAACTTTGATCAATTTACTTCTGTTACACAAATTTGAACACAAGATTAACACTGAAGGAATCATTGTTGTGCACGGAATTGCTGGAACTGGGAAAACCACATTGCTTAGGACTTTATTTTCTGCTTACCCTAGCTTAGTTATAGGTTCACCTAGGCCTTGTTACTTAGATAAAGCTAATAAAATTTCACAAGTTTGCCTTTCTTGTTTTCCAAATACCTTGTGTGACATTGTTGACGAGTACCATCTCTTAGAAAGTTTTCCTGAACCAAAACTAGCCATTTTTGGTGACCCCTGTCAGTGCACTTACATTGAAAGGTTGAGAACACCCAACTACACATCCTTCAGAACACACCGATTTGGCAAATCCACTGCTGCTCTACTAAACAAGTTATTTGATCTTAACATTGAGTCAGTCAAAGCACAAGACGACACAGTAGAATACTTTGATCCTTTCGCAGTGGACCCCTCTGAACACATTTCTGCTTCAGAAAAAGAAGTTTTGGAATTTGTAGGTGATCAAGTTGAGACTACAAGCTCTGAAGAACTAGCTGGTCTCGAGTTTAGTGAAGTTACTTTCTACTGTACCACACTTGCTGGTGCTGTTCAAGAAAATCCTGCCAAAACCTTCATTTCACTCACTAGACACACTTCAAAGCTCACAATTGGTGAACTAAATGCCAGGTCTGACTCCTAGATGCCAGGTCTGACTCCTAGAGCTGATCTTACTGACACGTATAAAATCATTGCTATAGCCTTTCTACTGTCAGCTTGCATTTACTTCCAAAACAGTCATTATCAACCAGTTGCAGGTGATAATTTGCACAGACTACCCTTTGGTGGTCAGTATCAAGACGGAACTAAGAAGATCTCTTACTTTCCGCAGCAACAATCCTACTTTCACTCAGGAAACAAGCTTAATGTCCTCATACTTATCTTCATTCTTACACTGGGTATTGTCCTCACCAATAAATTTAGTTTTAGCATTAGCCGTAATACTCACCAGCATCATTGCTACAACACACATTCTGCAACCCAAACAGGTCAATCAGTGCCAGGTCATCATTGAATGTCCTCATACTTATCTTCATTCTTACACTGGGTATTGTCCTCACCAATAAATTTAGTTTTAGCATTAGCCGTAATACTCACCAGCATCATTGCTACAACACACATTCTGCAACCCAAACAGGTCAATCAGTGCCAGGTCATCATTGATGGTGCAGCCATAGTCATAACAAATTGTCCAAACACACCCGAAGTTCTTAAAGCAATCAACTTCTCCCCTTGGAACGGGTTAAGTTTTCCTCAATTGTGAATGGAAAACCAACCTACAGCTTCTAACCCATCAGATGTACCACCAACTGCTGCTCAAGCTGGTGCCCAGAGCCCAGCCGACTTCTCAAATCCTAATACAGCTCCTTCCCTAAGTGATTTGAAGAAGATCAAATACGTGTCAACTGTCACTTCAGTTGCCACGCCTGCTGAAATTGAGGCCCTTGGCAAGATCTTTACTGCCATGGGTTTAGCAGCCAATGAGACCGGACCTGCCATGTGGGACCTCGCTCGTGCTTATGCTGATGTGCAAAGTTCAAAATCTGCACAACTTATAGGTGCCACACCATCCAACCCTGCTTTGTCTAGACGTGCACTTGCTGCACAGTTTGATCGTATCAATATCACACCCAGACAATTCTGCATGTATTTTGCAAAAATTGTTTGGAACATACTGTTAGACAGCAATGTGCCACCTGCCAACTGGGCAAAATTGGGCTATCAGGAAGATACCAAGTTTGCTGCTTTTGACTTCTTTGATGGAGTCACAAATCCAGCTAGTCTACAGCCTGCAGATGGCCTAATCAGGCAGCCCAATGAAAAAGAGCTTGCTGCTCACTCGGTTGCTAAATATGGTGCCCTTGCCCGCCAGAAAATATCCACTGGTAACTACATCACCACCCTTGGTGAAGTTACACGTGGTCACATGGGCGGCGCCAACACTATGTACGCAATTGATGCACCTCCTGAACTTTAA

>PS5*_P8_b9

ATGGAAAGATCAACTTTGATCAATTTACTTCTGTTACACAAATTTGAACACAAGATTAACACTGAAGGAATCATTGTTGTGCACGGAATTGCTGGAACTGGGAAAACCACATTGCTTAGGACTTTATTTTCTGCTTACCCTAGCTTAGTTATAGGTTCACCTAGGCCTTGTTACTTAGATAAAGCTAATAAAATTTCACAAGTTTGCCTTTCTTGTTTTCCAAATACCTTGTGTGACATTGTTGACGAGTACCATCTCTTAGAAAGTTTTCCTGAACCAAAACTAGCCATTTTTGGTGACCCCTGTCAGTGCACTTACATTGAAAGGTTGAGAACACCCAACTACACATCCTTCAGAACACACCGATTTGGCAAATCCACTGCTGCTCTACTAAACAAGTTATTTGATCTTAACATTGAGTCAGTCAAAGCACAAGACGACACAGTAGAATACTTTGATCCTTTCGCAGTGGACCCCTCTGAACACATTTCTGCTTCAGAAAAAGAAGTTTTGGAATTTGTAGGTGATCAAGTTGAGACTACAAGCTCTGAAGAACTAGCTGGTCTCGAGTTTAGTGAAGTTACTTTCTACTGTACCACACTTGCTGGTGCTGTTCAAGAAAATCCTGCCAAAACCTTCATTTCACTCACTAGACACACTTCAAAGCTCACAATTGGTGAACTAAATGCCAGGTCTGACTCCTAGATGCCAGGTCTGACTCCTAGAGCTGATCTTACTGACACGTATAAAATCATTGCTATAGCCTTTCTACTGTCAGCTTGCATTTACTTCCAAAACAGTCATTATCAACCAGTTGCAGGTGATAATTTGCACAGACTACCCTTTGGTGGTCAGTATCAAGACGGAACTAAGAAGATCTCTTACTTTCCGCAGCAACAATCCTACTTTCACTCAGGAAACAAGCTTAATGTCCTCATACTTATCTTCATTCTTACACTGGGTATTGTCCTCACCAATAAATTTAGTTTTAGCATTAGCCGTAATACTCACCAGCATCATTGCTACAACACACATTCTGCAACCCAAACAGGTCAATCAGTGCCAGGTCATCATTGAATGTCCTCATACTTATCTTCATTCTTACACTGGGTATTGTCCTCACCAATAAATTTAGTTTTAGCATTAGCCGTAATACTCACCAGCATCATTGCTACAACACACATTCTGCAACCCAAACAGGTCAATCAGTGCCAGGTCATCATTGATGGTGCAGCCATAGTCATAACAAATTGTCCAAACACACCCGAAGTTCTTAAAGCAATCAACTTCTCCCCTTGGAACGGGTTAAGTTTTCCTCAATTGTGAATGGAAAACCAACCTACAGCTTCTAACCCATCAGATGTACCACCAACTGCTGCTCAAGCTGGTGCCCAGAGCCCAGCCGACTTCTCAAATCCTAATACAGCTCCTTCCCTAAGTGATTTGAAGAAGATCAAATACGTGTCAACTGTCACTTCAGTTGCCACGCCTGCTGAAATTGAGGCCCTTGGCAAGATCTTTACTGCCATGGGTTTAGCAGCCAATGAGACCGGACCTGCCATGTGGGACCTCGCTCGTGCTTATGCTGATGTGCAAAGTTCAAAATCTGCACAACTTATAGGTGCCACACCATCCAACCCTGCTTTGTCTAGACGTGCACTTGCTGCACAGTTTGATCGTATCAATATCACACCCAGACAATTCTGCATGTATTTTGCAAAAATTGTTTGGAACATACTGTTAGACAGCAATGTGCCACCTGCCAACTGGGCAAAATTGGGCTATCAGGAAGATACCAAGTTTGCTGCTTTTGACTTCTTTGATGGAGTCACAAATCCAGCTAGTCTACAGCCTGCAGATGGCCTAATCAGGCAGCCCAATGAAAAAGAGCTTGCTGCTCACTCGGTTGCTAAATATGGTGCCCTTGCCCGCCAGAAAATATCCACTGGTAACTACATCACCACCCTTGGTGAAGTTACACGTGGTCACATGGGCGGCGCCAACACTATGTACGCAATTGATGCACCTCCTGAACTTTAA

>PS5*_P8_b8

ATGGAAAGATCAACTTTGATCAATTTACTTCTGTTACACAAATTTGAACACAAGATTAACACTGAAGGAATCATTGTTGTGCACGGAATTGCTGGAACTGGGAAAACCACATTGCTTAGGACTTTATTTTCTGCTTACCCTAGCTTAGTTATAGGTTCACCTAGGCCTTGTTACTTAGATAAAGCTAATAAAATTTCACAAGTTTGCCTTTCTTGTTTTCCAAATACCTTGTGTGACATTGTTGACGAGTACCATCTCTTAGAAAGTTTTCCTGAACCAAAACTAGCCATTTTTGGTGACCCCTGTCAGTGCACTTACATTGAAAGGTTGAGAACACCCAACTACACATCCTTCAGAACACACCGATTTGGCAAATCCACTGCTGCTCTACTAAACAAGTTATTTGATCTTAACATTGAGTCAGTCAAAGCACAAGACGACACAGTAGAATACTTTGATCCTTTCGCAGTGGACCCCTCTGAACACATTTCTGCTTCAGAAAAAGAAGTTTTGGAATTTGTAGGTGATCAAGTTGAGACTACAAGCTCTGAAGAACTAGCTGGTCTCGAGTTTAGTGAAGTTACTTTCTACTGTACCACACTTGCTGGTGCTGTTCAAGAAAATCCTGCCAAAACCTTCATTTCACTCACTAGACACACTTCAAAGCTCACAATTGGTGAACTAAATGCCAGGTCTGACTCCTAGATGCCAGGTCTGACTCCTAGAGCTGATCTTACTGACACGTATAAAATCATTGCTATAGCCTTTCTACTGTCAGCTTGCATTTACTTCCAAAACAGTCATTATCAACCAGTTGCAGGTGATAATTTGCACAGACTACCCTTTGGTGGTCAGTATCAAGACGGAACTAAGAAGATCTCTTACTTTCCGCAGCAACAATCCTACTTTCACTCAGGAAACAAGCTTAATGTCCTCATACTTATCTTCATTCTTACACTGGGTATTGTCCTCACCAATAAATTTAGTTTTAGCATTAGCCGTAATACTCACCAGCATCATTGCTACAACACACATTCTGCAACCCAAACAGGTCAATCAGTGCCAGGTCATCATTGAATGTCCTCATACTTATCTTCATTCTTACACTGGGTATTGTCCTCACCAATAAATTTAGTTTTAGCATTAGCCGTAATACTCACCAGCATCATTGCTACAACACACATTCTGCAACCCAAACAGGTCAATCAGTGCCAGGTCATCATTGATGGTGCAGCCATAGTCATAACAAATTGTCCAAACACACCCGAAGTTCTTAAAGCAATCAACTTCTCCCCTTGGAACGGGTTAAGTTTTCCTCAATTGTGAATGGAAAACCAACCTACAGCTTCTAACCCATCAGATGTACCACCAACTGCTGCTCAAGCTGGTGCCCAGAGCCCAGCCGACTTCTCAAATCCTAATACAGCTCCTTCCCTAAGTGATTTGAAGAAGATCAAATACGTGTCAACTGTCACTTCAGTTGCCACGCCTGCTGAAATTGAGGCCCTTGGCAAGATCTTTACTGCCATGGGTTTAGCAGCCAATGAGACCGGACCTGCCATGTGGGACCTCGCTCGTGCTTATGCTGATGTGCAAAGTTCAAAATCTGCACAACTTATAGGTGCCACACCATCCAACCCTGCTTTGTCTAGACGTGCACTTGCTGCACAGTTTGATCGTATCAATATCACACCCAGACAATTCTGCATGTATTTTGCAAAAATTGTTTGGAACATACTGTTAGACAGCAATGTGCCACCTGCCAACTGGGCAAAATTGGGCTATCAGGAAGATACCAAGTTTGCTGCTTTTGACTTCTTTGATGGAGTCACAAATCCAGCTAGTCTACAGCCTGCAGATGGCCTAATCAGGCAGCCCAATGAAAAAGAGCTTGCTGCTCACTCGGTTGCTAAATATGGTGCCCTTGCCCGCCAGAAAATATCCACTGGTAACTACATCACCACCCTTGGTGAAGTTACACGTGGTCACATGGGCGGCGCCAACACTATGTACGCAATTGATGCACCTCCTGAACTTTAA

>PS5*_P8_b7

ATGGAAAGATCAACTTTGATCAATTTACTTCTGTTACACAAATTTGAACACAAGATTAACACTGAAGGAATCATTGTTGTGCACAGAATTGCTGGAACTGGGAAAACCACATTGCTTAGGACTTTATTTTCTGCTTACCCTAGCTTAGTTATAGGTTCACCTAGGCCTTGTTACTTAGATAAAGCTAATAAAATTTCACAAGTTTGCCTTTCTTGTTTTCCAAATACCTTGTGTGACATTGTTGACGAGTACCATCTCTTAGAAAGTTTTCCTGAACCAAAACTAGCCATTTTTGGTGACCCCTGTCAGTGCACTTACATTGAAAGGTTGAGAACACCCAACTACACATCCTTCAGAACACACCGATTTGGCAAATCCACTGCTGCTCTACTAAACAAGTTATTTGATCTTAACATTGAGTCAGTCAAAGCACAAGACGACACAGTAGAATACTTTGATCCTTTCGCAGTGGACCCCTCTGAACACATTTCTGCTTCAGAAAAAGAAGTTTTGGAATTTGTAGGTGATCAAGTTGAGACTACAAGCTCTGAAGAACTAGCTGGTCTCGAGTTTAGTGAAGTTACTTTCTACTGTACCACACTTGCTGGTGCTGTTCAAGAAAATCCTGCCAAAACCTTCATTTCACTCACTAGACACACTTCAAAGCTCACAATTGGTGAACTAAATGCCAGGTCTGACTCCTAGATGCCAGGTCTGACTCCTAGAGCTGATCTTACTGACACGTATAAAATCATTGCTATAGCCTTTCTACTGTCAGCTTGCATTTACTTCCAAAACAGTCATTATCAACCAGTTGCAGGTGATAATTTGCACAGACTACCCTTTGGTGGTCAGTATCAAGACGGAACTAAGAAGATCTCTTACTTTCCGCAGCAACAATCCTACTTTCACTCAGGAAACAAGCTTAATGTCCTCATACTTATCTTCATTCTTACACTGGGTATTGTCCTCACCAATAAATTTAGTTTTAGCATTAGCCGTAATACTCACCAGCATCATTGCTACAACACACATTCTGCAACCCAAACAGGTCAATCAGTGCCAGGTCATCATTGAATGTCCTCATACTTATCTTCATTCTTACACTGGGTATTGTCCTCACCAATAAATTTAGTTTTAGCATTAGCCGTAATACTCACCAGCATCATTGCTACAACACACATTCTGCAACCCAAACAGGTCAATCAGTGCCAGGTCATCATTGATGGTGCAGCCATAGTCATAACAAATTGTCCAAACACACCCGAAGTTCTTAAAGCAATCAACTTCTCCCCTTGGAACGGGTTAAGTTTTCCTCAATTGTGAATGGAAAACCAACCTACAGCTTCTAACCCATCAGATGTACCACCAACTGCTGCTCAAGCTGGTGCCCAGAGCCCAGCCGACTTCTCAAATCCTAATACAGCTCCTTCCCTAAGTGATTTGAAGAAGATCAAATACGTGTCAACTGTCACTTCAGTTGCCACGCCTGCTGAAATTGAGGCCCTTGGCAAGATCTTTACTGCCATGGGTTTAGCAGCCAATGAGACCGGACCTGCCATGTGGGACCTCGCTCGTGCTTATGCTGATGTGCAAAGTTCAAAATCTGCACAACTTATAGGTGCCACACCATCCAACCCTGCTTTGTCTAGACGTGCACTTGCTGCACAGTTTGATCGTATCAATATCACACCCAGACAATTCTGCATGTATTTTGCAAAAATTGTTTGGAACATACTGTTAGACAGCAATGTGCCACCTGCCAACTGGGCAAAATTGGGCTATCAGGAAGATACCAAGTTTGCTGCTTTTGACTTCTTTGATGGAGTCACAGATCCAGCTAGTCTACAGCCTGCAGATGGCCTAATCAGGCAGCCCAATGAAAAAGAGCTTGCTGCTCACTCGGTTGCTAAATATGGTGCCCTTGCCCGCCAGAAAATATCCACTGGTAACTACATCACCACCCTTGGTGAAGTTACACGTGGTCACATGGGCGGCGCCAACACTATGTACGCAATTGATGCACCTCCTGAACTTTAA

>PS5*_P8_b6

ATGGAAAGATCAACTTTGATCAATTTACTTCTGTTACACAAATTTGAACACAAGATTAACACTGAAGGAATCATTGTTGTGCACGGAATTGCTGGAACTGGGAAAACCACATTGCTTAGGACTTTATTTTCTGCTTACCCTAGCTTAGTTATAGGTTCACCTAGGCCTTGTTACTTAGATAAAGCTAATAAAATTTCACAAGTTTGCCTTTCTTGTTTTCCAAATACCTTGTGTGACATTGTTGACGAGTACCATCTCTTAGAAAGTTTTCCTGAACCAAAACTAGCCATTTTTGGTGACCCCTGTCAGTGCACTTACATTGAAAGGTTGAGAACACCCAACTACACATCCTTCAGAACACACCGATTTGGCAAATCCACTGCTGCTCTACTAAACAAGTTATTTGATCTTAACATTGAGTCAGTCAAAGCACAAGACGACACAGTAGAATACTTTGATCCTTTCGCAGTGGACCCCTCTGAACACATTTCTGCTTCAGAAAAAGAAGTTTTGGAATTTGTAGGTGATCAAGTTGAGACTACAAGCTCTGAAGAACTAGCTGGTCTCGAGTTTAGTGAAGTTACTTTCTACTGTACCACACTTGCTGGTGCTGTTCAAGAAAATCCTGCCAAAACCTTCATTTCACTCACTAGACACACTTCAAAGCTCACAATTGGTGAACTAAATGCCAGGTCTGACTCCTAGATGCCAGGTCTGACTCCTAGAGCTGATCTTACTGACACGTATAAAATCATTGCTATAGCCTTTCTACTGTCAGCTTGCATTTACTTCCAAAACAGTCATTATCAACCAGTTGCAGGTGATAATTTGCACAGACTACCCTTTGGTGGTCAGTATCAAGACGGAACTAAGAAGATCCCTTACTTTCCGCAGCAACAATCCTACTTTCACTCAGGAAACAAGCTTAATGTCCTCATACTTATCTTCATTCTTACACTGGGTATTGTCCTCACCAATAAATTTAGTTTTAGCATTAGCCGTAATACTCACCAGCATCATTGCTACAACACACATTCTGCAACCCAAACAGGTCAATCAGTGCCAGGTCATCATTGAATGTCCTCATACTTATCTTCATTCTTACACTGGGTATTGTCCTCACCAATAAATTTAGTTTTAGCATTAGCCGTAATACTCACCAGCATCATTGCTACAACACACATTCTGCAACCCAAACAGGTCAATCAGTGCCAGGTCATCATTGATGGTGCAGCCATAGTCATAACAAATTGTCCAAACACACCCGAAGTTCTTAAAGCAATCAACTTCTCCCCTTGGAACGGGTTAAGTTTTCCTCAATTGTGAATGGAAAACCAACCTACAGCTTCTAACCCATCAGATGTACCACCAACTGCTGCTCAAGCTGGTGCCCAGAGCCCAGCCGACTTCTCAAATCCTAATACAGCTCCTTCCCTAAGTGATTTGAAGAAGATCAAATACGTGTCAACTGTCACTTCAGTTGCCACGCCTGCTGAAATTGAGGCCCTTGGCAAGATCTTTACTGCCATGGGTTTAGCAGCCAATGAGACCGGACCTGCCATGTGGGACCTCGCTCGTGCTTATGCTGATGTGCAAAGTTCAAAATCTGCACAACTTATAGGTGCCACACCATCCAACCCTGCTTTGTCTAGACGTGCACTTGCTGCACAGTTTGATCGTATCAATATCACACCCAGACAATTCTGCATGTATTTTGCAAAAATTGTTTGGAACATACTGTTAGACAGCAATGTGCCACCTGCCAACTGGGCAAAATTGGGCTATCAGGAAGATACCAAGTTTGCTGCTTTTGACTTCTTTGATGGAGTCACAAATCCAGCTAGTCTACAGCCTGCAGATGGCCTAATCAGGCAGCCCAATGAAAAAGAGCTTGCTGCTCACTCGGTTGCTAAATATGGTGCCCTTGCCCGCCAGAAAATATCCACTGGTAACTACATCACCACCCTTGGTGAAGTTACACGTGGTCACATGGGCGGCGCCAACACTATGTACGCAATTGATGCACCTCCTGAACTTTAA

>PS5*_P8_b5

ATGGAAAGATCAACTTTGATCAATTTACTTCTGTTACACAAATTTGAACACAAGATTAACACTGAAGGAATCATTGTTGTGCACGGAATTGCTGGAACTGGGAAAACCACATTGCTTAGGACTTTATTTTCTGCTTACCCTAGCTTAGTTATAGGTTCACCTAGGCCTTGTTACTTAGATAAAGCTAATAAAATTTCACAAGTTTGCCTTTCTTGTTTTCCAAATACCTTGTGTGACATTGTTGACGAGTACCATCTCTTAGAAAGTTTTCCTGAACCAAAACTAGCCATTTTTGGTGACCCCTGTCAGTGCACTTACATTGAAAGGTTGAGAACACCCAACTACACATCCTTCAGAACACACCGATTTGGCAAATCCACTGCTGCTCTACTAAACAAGTTATTTGATCTTAACATTGAGTCAGTCAAAGCACAAGACGACACAGTAGAATACTTTGATCCTTTCGCAGTGGACCCCTCTGAACACATTTCTGCTTCAGAAAAAGAAGTTTTGGAATTTGTAGGTGATCAAGTTGAGACTACAAGCTCTGAAGAACTAGCTGGTCTCGAGTTTAGTGAAGTTACTTTCTACTGTACCACACTTGCTGGTGCTGTTCAAGAAAATCCTGCCAAAACCTTCATTTCACTCACTAGACACACTTCAAAGCTCACAATTGGTGAACTAAATGCCAGGTCTGACTCCTAGATGCCAGGTCTGACTCCTAGAGCTGATCTTACTGACACGTATAAAATCATTGCTATAGCCTTTCTACTGTCAGCTTGCATTTACTTCCAAAACAGTCATTATCAACCAGTTGCAGGTGATAATTTGCACAGACTACCCTTTGGTGGTCAGTATCAAGACGGAACTAAGAAGATCTCTTACTTTCCGCAGCAACAATCCTACTTTCACTCAGGAAACAAGCTTAATGTCCTCATACTTATCTTCATTCTTACACTGGGTATTGTCCTCACCAATAAATTTAGTTTTAGCATTAGCCGTAATACTCACCAGCATCATTGCTACAACACACATTCTGCAACCCAAACAGGTCAATCAGTGCCAGGTCATCATTGAATGTCCTCATACTTATCTTCATTCTTACACTGGGTATTGTCCTCACCAATAAATTTAGTTTTAGCATTAGCCGTAATACTCACCAGCATCATTGCTACAACACACATTCTGCAACCCAAACAGGTCAATCAGTGCCAGGTCATCATTGATGGTGCAGCCATAGTCATAACAAATTGTCCAAACACACCCGAAGTTCTTAAAGCAATCAACTTCTCCCCTTGGAACGGGTTAAGTTTTCCTCAATTGTGAATGGAAAACCAACCTACAGCTTCTAACCCATCAGATGTACCACCAACTGCTGCTCAAGCTGGTGCCCAGAGCCCAGCCGACTTCTCAAATCCTAATACAGCTCCTTCCCTAAGTGATTTGAAGAAGATCAAATACGTGTCAACTGTCACTTCAGTTGCCACGCCTGCTGAAATTGAGGCCCTTGGCAAGATCTTTACTGCCATGGGTTTAGCAGCCAATGAGACCGGACCTGCCATGTGGGACCTCGCTCGTGCTTATGCTGATGTGCAAAGTTCAAAATCTGCACAACTTATAGGTGCCACACCATCCAACCCTGCTTTGTCTAGACGTGCACTTGCTGCACAGTTTGATCGTATCAATATCACACCCAGACAATTCTGCATGTATTTTGCAAAAATTGTTTGGAACATACTGTTAGACAGCAATGTGCCACCTGCCAACTGGGCAAAATTGGGCTATCAGGAAGATACCAAGTTTGCTGCTTTTGACTTCTTTGATGGAGTCACAAATCCAGCTAGTCTACAGCCTGCAGATGGCCTAATCAGGCAGCCCAATGAAAAAGAGCTTGCTGCTCACTCGGTTGCTAAATATGGTGCCCTTGCCCGCCAGAAAATATCCACTGGTAACTACATCACCACCCTTGGTGAAGTTACACGTGGTCACATGGGCGGCGCCAACACTATGTACGCAATTGATACACCTCCTGAACTTTAA

>PS5*_P8_b4

ATGGAAAGATCAGCTTTGATCAATTTACTTCTGTTACACAAATTTGAACACAAGATTAACACTGAAGGAATCATTGTTGTGCACGGAATTGCTGGAACTGGGAAAACCACATTGCTTAGGACTTTATTTTCTGCTTACCCTAGCTTAGTTATAGGTTCACCTAGGCCTTGTTACTTAGATAAAGCTAATAAAATTTCACAAGTTTGCCTTTCTTGTTTTCCAAATACCTTGTGTGACATTGTTGACGAGTACCATCTCTTAGAAAGTTTTCCTGAACCAAAACTAGCCATTTTTGGTGACCCCTGTCAGTGCACTTACATTGAAAGGTTGAGAACACCCAACTACACATCCTTCAGAACACACCGATTTGGCAAATCCACTGCTGCTCTACTAAACAAGTTATTTGATCTTAACATTGAGTCAGTCAAAGCACAAGACGACACAGTAGAATACTTTGATCCTTTCGCAGTGGACCCCTCTGAACACATTTCTGCTTCAGAAAAAGAAGTTTTGGAATTTGTAGGTGATCAAGTTGAGACTACAAGCTCTGAAGAACTAGCTGGTCTCGAGTTTAGTGAAGTTACTTTCTACTGTACCACACTTGCTGGTGCTGTTCAAGAAAATCCTGCCAAAACCTTCATTTCACTCACTAGACACACTTCAAAGCTCACAATTGGTGAACTAAATGCCAGGTCTGACTCCTAGATGCCAGGTCTGACTCCTAGAGCTGATCTTACTGACACGTATAAAATCATTGCTATAGCCTTTCTACTGTCAGCTTGCATTTACTTCCAAAACAGTCATTATCAACCAGTTGCAGGTGATAATTTGCACAGACTACCCTTTGGTGGTCAGTATCAAGACGGAACTAAGAAGATCTCTTACTTTCCGCAGCAACAATCCTACTTTCACTCAGGAAACAAGCTTAATGTCCTCATACTTATCTTCATTCTTACACTGGGTATTGTCCTCACCAATAAATTTAGTTTTAGCATTAGCCGTAATACTCACCAGCATCATTGCTACAACACACATTCTGCAACCCAAACAGGTCAATCAGTGCCAGGTCATCATTGAATGTCCTCATACTTATCTTCATTCTTACACTGGGTATTGTCCTCACCAATAAATTTAGTTTTAGCATTAGCCGTAATACTCACCAGCATCATTGCTACAACACACATTCTGCAACCCAAACAGGTCAATCAGTGCCAGGTCATCATTGATGGTGCAGCCATAGTCATAACAAATTGTCCAAACACACCCGAAGTTCTTAAAGCAATCAACTTCTCCCCTTGGAACGGGTTAAGTTTTCCTCAATTGTGAATGGAAAACCAACCTACAGCTTCTAACCCATCAGATGTACCACCAACTGCTGCTCAAGCTGGTGCCCAGAGCCCAGCCGACTTCTCAAATCCTAATACAGCTCCTTCCCTAAGTGATTTGAAGAAGATCAAATACGTGTCAACTGTCACTTCAGTTGCCACGCCTGCTGAAATTGAGGCCCTTGGCAAGATCTTTACTGCCATGGGTTTAGCAGCCAATGAGACCGGACCTGCCATGTGGGACCTCGCTCGTGCTTATGCTGATGTGCAAAGTTCAAAATCTGCACAACTTATAGGTGCCACACCATCCAACCCTGCTTTGTCTAGACGTGCACTTGCTGCACAGTTTGATCGTATCAATATCACACCCAGACAATTCTGCATGTATTTTGCAAAAATTGTTTGGAACATACTGTTAGACAGCAATGTGCCACCTGCCAACTGGGCAAAATTGGGCTATCAGGAAGATACCAAGTTTGCTGCTTTTGACTTCTTTGATGGAGTCACAAATCCAGCTAGTCTACAGCCTGCAGATGGCCTAATCAGGCAGCCCAATGAAAAAGAGCTTGCTGCTCACTCGGTTGCTAAATATGGTGCCCTTGCCCGCCAGAAAATATCCACTGGTAACTACATCACCACCCTTGGTGAAGTTACACGTGGTCACATGGGCGGCGCCAACACTATGTACGCAATTGATGCACCTCCTGAACTTTAA

>PS5*_P8_b3

ATGGAAAGATCAACTTTGATCAATTTACTTCTGTTACACAAATTTGAACACAAGATTAACACTGAAGGAATCATTGTTGTGCACGGAATTGCTGGAACTGGGAAAACCACATTGCTTAGGACTTTATTTTCTGCTTACCCTAGCTTAGTTATAGGTTCACCTAGGCCTTGTTACTTAGATAAAGCTAATAAAATTTCACAAGTTTGCCTTTCTTGTTTTCCAAATACCTTGTGTGACATTGTTGACGAGTACCATCTCTTAGAAAGTTTTCCTGAACCAAAACTAGCCATTTTTGGTGACCCCTGTCAGTGCACTTACATTGAAAGGTTGAGAACACCCAACTACACATCCTTCAGAACACACCGATTTGGCAAATCCACTGCTGCTCTACTAAACAAGTTATTTGATCTTAACATTGAGTCAGTCAAAGCACAAGACGACACAGTAGAATACTTTGATCCTTTCGCAGTGGACCCCTCTGAACACATTTCTGCTTCAGAAAAAGAAGTTTTGGAATTTGTAGGTGATCAAGTTGAGACTACAAGCTCTGAAGAACTAGCTGGTCTCGAGTTTAGTGAAGTTACTTTCTACTGTACCACACTTGCTGGTGCTGTTCAAGAAAATCCTGCCAAAACCTTCATTTCACTCACTAGACACACTTCAAAGCTCACAATTGGTGAACTAAATGCCAGGTCTGACTCCTAGATGCCAGGTCTGACTCCTAGAGCTGATCTTACTGACACGTATAAAATCATTGCTATAGCCTTTCTACTGTCAGCTTGCATTTACTTCCAAAACAGTCATTATCAACCAGTTGCAGGTGATAATTTGCACAGACTACCCTTTGGTGGTCAGTATCAAGACGGAACTAAGAAGATCTCTTACTTTCCGCAGCAACAATCCTACTTTCACTCAGGAAACAAGCTTAATGTCCTCATACTTATCTTCATTCTTACACTGGGTATTGTCCTCACCAATAAATTTAGTTTTAGCATTAGCCGTAATACTCACCAGCATCATTGCTACAACACACATTCTGCAACCCAAACAGGTCAATCAGTGCCAGGTCATCATTGAATGTCCTCATACTTATCTTCATTCTTACACTGGGTATTGTCCTCACCAATAAATTTAGTTTTAGCATTAGCCGTAATACTCACCAGCATCATTGCTACAACACACATTCTGCAACCCAAACAGGTCAATCAGTGCCAGGTCATCATTGATGGTGCAGCCATAGTCATAACAAATTGTCCAAACACACCCGAAGTTCTTAAAGCAATCAACTTCTCCCCTTGGAACGGGTTAAGTTTTCCTCAATTGTGAATGGAAAACCAACCTACAGCTTCTAACCCATCAGATGTACCACCAACTGCTGCTCAAGCTGGTGCCCAGAGCCCAGCCGACTTCTCAAATCCTAATACAGCTCCTTCCCTAAGTGATTTGAAGAAGATCAAATACGTGTCAACTGTCACTTCAGTTGCCACGCCTGCTGAAATTGAGGCCCTTGGCAAGATCTTTACTGCCATGGGTTTAGCAGCCAATGAGACCGGACCTGCCATGTGGGACCTCGCTCGTGCTTATGCTGATGTGCAAAGTTCAAAATCTGCACAACTTATAGGTGCCACACCATCCAACCCTGCTTTGTCTAGACGTGCACTTGCTGCACAGTTTGATCGTATCAATATCACACCCAGACAATTCTGCATGTATTTTGCAAAAATTGTTTGGAACATACTGTTAGACAGCAATGTGCCACCTGCCAACTGGGCAAAATTGGGCTATCAGGAAGATACCAAGTTTGCTGCTTTTGACTTCTTTGATGGAGTCACAAATCCAGCTAGTCTACAGCCTGCAGATGGCCTAATCAGGCAGCCCAATGAAAAAGAGCTTGCTGCTCACTCGGTTGCTAAATATGGTGCCCTTGCCCGCCAGAAAATATCCACTGGTAACTACATCACCACCCTTGGTGAAGTTACACGTGGTCACATGGGCGGCGCCAACACTATGTACGCAATTGATGCACCTCCTGAACTTTAA

>PS5*_P8_b2

ATGGAAAGATCAACTTTGATCAATTTACTTCTGTTACACAAATTTGAACACAAGATTAACACTGAAGGAATCATTGTTGTGCACGGAATTGCTGGAACTGGGAAAACCACATTGCTTAGGACTTTATTTTCTGCTTACCCTAGCTTAGTTATAGGTTCACCTAGGCCTTGTTACTTAGATAAAGCTAATAAAATTTCACAAGTTTGCCTTTCTTGTTTTCCAAATACCTTGTGTGACATTGTTGACGAGTACCATCTCTTAGAAAGTTTTCCTGAACCAAAACTAGCCATTTTTGGTGACCCCTGTCAGTGCACTTACATTGAAAGGTTGAGAACACCCAACTACACATCCTTCAGAACACACCGATTTGGCAAATCCACTGCTGCTCTACTAAACAAGTTATTTGATCTTAACATTGAGTCAGTCAAAGCACAAGACGACACAGTAGAATACTTTGATCCTTTCGCAGTGGACCCCTCTGAACACATTTCTGCTTCAGAAAAAGAAGTTTTGGAATTTGTAGGTGATCAAGTTGAGACTACAAGCTCTGAAGAACTAGCTGGTCTCGAGTTTAGTGAAGTTACTTTCTACTGTACCACACTTGCTGGTGCTGTTCAAGAAAATCCTGCCAAAACCTTCATTTCACTCACTAGACACACTTCAAAGCTCACAATTGGTGAACTAAATGCCAGGTCTGACTCCTAGATGCCAGGTCTGACTCCTAGAGCTGATCTTACTGACACGTATAAAATCATTGCTATAGCCTTTCTACTGTCAGCTTGCATTTACTTCCAAAACAGTCATTATCAACCAGTTGCAGGTGATAATTTGCACAGACTACCCTTTGGTGGTCAGTATCAAGACGGAACTAAGAAGATCCCTTACTTTCCGCAGCAACAATCCTACTTTCACTCAGGAAACAAGCTTAATGTCCTCATACTTATCTTCATTCTTACACTGGGTATTGTCCTCACCAATAAATTTAGTTTTAGCATTAGCCGTAATACTCACCAGCATCATTGCTACAACACACATTCTGCAACCCAAACAGGTCAATCAGTGCCAGGTCATCATTGAATGTCCTCATACTTATCTTCATTCTTACACTGGGTATTGTCCTCACCAATAAATTTAGTTTTAGCATTAGCCGTAATACTCACCAGCATCATTGCTACAACACACATTCTGCAACCCAAACAGGTCAATCAGTGCCAGGTCATCATTGATGGTGCAGCCATAGTCATAACAAATTGTCCAAACACACCCGAAGTTCTTAAAGCAATCAACTTCTCCCCTTGGAACGGGTTAAGTTTTCCTCAATTGTGAATGGAAAACCAACCTACAGCTTCTAACCCATCAGATGTACCACCAACTGCTGCTCAAGCTGGTGCCCAGAGCCCAGCCGACTTCTCAAATCCTAATACAGCTCCTTCCCTAAGTGATTTGAAGAAGATCAAATACGTGTCAACTGTCACTTCAGTTGCCACGCCTGCTGAAATTGAGGCCCTTGGCAAGATCTTTACTGCCATGGGTTTAGCAGCCAATGAGACCGGACCTGCCATGTGGGACCTCGCTCGTGCTTATGCTGATGTGCAAAGTTCAAAATCTGCACAACTTATAGGTGCCACACCATCCAACCCTGCTTTGTCTAGACGTGCACTTGCTGCACAGTTTGATCGTATCAATATCACACCCAGACAATTCTGCATGTATTTTGCAAAAATTGTTTGGAACATACTGTTAGACAGCAATGTGCCACCTGCCAACTGGGCAAAATTGGGCTATCAGGAAGATACCAAGTTTGCTGCTTTTGACTTCTTTGATGGAGTCACAAATCCAGCTAGTCTACAGCCTGCAGATGGCCTAATCAGGCAGCCCAATGAAAAAGAGCTTGCTGCTCACTCGGTTGCTAAATATGGTGCCCTTGCCCGCCAGAAAATATCCACTGGTAACTACATCACCACCCTTGGTGAAGTTACACGTGGTCACATGGGCGGCGCCAACACTATGTACGCAATTGATGCACCTCCTGAACTTTAA

>PS5*_P8_b1

ATGGAAAGATCAACTTTGATCAATTTACTTCTGTTACACAAATTTGAACACAAGATTAACACTGAAGGAATCATTGTTGTGCACGGAATTGCTGGAACTGGGAAAACCACATTGCTTAGGACTTTATTTTCTGCTTACCCTAGCTTAGTTATAGGTTCACCTAGGCCTTGTTACTTAGATAAAGCTAATAAAATTTCACAAGTTTGCCTTTCTTGTTTTCCAAATACCTTGTGTGACATTGTTGACGAGTACCATCTCTTAGAAAGTTTTCCTGAACCAAAACTAGCCATTTTTGGTGACCCCTGTCAGTGCACTTACATTGAAAGGTTGAGAACACCCAACTACACATCCTTCAGAACACACCGATTTGGCAAATCCACTGCTGCTCTACTAAACAAGTTATTTGATCTTAACATTGAGTCAGTCAAAGCACAAGACGACACAGTAGAATACTTTGATCCTTTCGCAGTGGACCCCTCTGAACACATTTCTGCTTCAGAAAAAGAAGTTTTGGAATTTGTAGGTGATCAAGTTGAGACTACAAGCTCTGAAGAACTAGCTGGTCTCGAGTTTAGTGAAGTTACTTTCTACTGTACCACACTTGCTGGTGCTGTTCAAGAAAATCCTGCCAAAACCTTCATTTCACTCACTAGACACACTTCAAAGCTCACAATTGGTGAACTAAATGCCAGGTCTGACTCCTAGATGCCAGGTCTGACTCCTAGAGCTGATCTTACTGACACGTATAAAATCATTGCTATAGCCTTTCTACTGTCAGCTTGCATTTACTTCCAAAACAGTCATTATCAACCAGTTGCAGGTGATAATTTGCACAGACTACCCTTTGGTGGTCAGTATCAAGACGGAACTAAGAAGATCTCTTACTTTCCGCAGCAACAATCCTACTTTCACTCAGGAAACAAGCTTAATGTCCTCATACTTATCTTCATTCTTACACTGGGTATTGTCCTCACCAATAAATTTAGTTTTAGCATTAGCCGTAATACTCACCAGCATCATTGCTACAACACACATTCTGCAACCCAAACAGGTCAATCAGTGCCAGGTCATCATTGAATGTCCTCATACTTATCTTCATTCTTACACTGGGTATTGTCCTCACCAATAAATTTAGTTTTAGCATTAGCCGTAATACTCACCAGCATCATTGCTACAACACACATTCTGCAACCCAAACAGGTCAATCAGTGCCAGGTCATCATTGATGGTGCAGCCATAGTCATAACAAATTGTCCAAACACACCCGAAGTTCTTAAAGCAATCAACTTCTCCCCTTGGAACGGGTTAAGTTTTCCTCAATTGTGAATGGAAAACCAACCTACAGCTTCTAACCCATCAGATGTACCACCAACTGCTGCTCAAGCTGGTGCCCAGAGCCCAGCCGACTTCTCAAATCCTAATACAGCTCCTTCCCTAAGTGATTTGAAGAAGATCAAATACGTGTCAACTGTCACTTCAGTTGCCACGCCTGCTGAAATTGAGGCCCTTGGCAAGATCTTTACTGCCATGGGTTTAGCAGCCAATGAGACCGGACCTGCCATGTGGGACCTCGCTCGTGCTTATGCTGATGTGCAAAGTTCAAAATCTGCACAACTTATAGGTGCCACACCATCCAACCCTGCTTTGTCTAGACGTGCACTTGCTGCACAGTTTGATCGTATCAATATCACACCCAGACAATTCTGCATGTATTTTGCAAAAATTGTTTGGAACATACTGTTAGACAGCAATGTGCCACCTGCCAACTGGGCAAAATTGGGCTATCAGGAAGATACCAAGTTTGCTGCTTTTGACTTCTTTGATGGAGTCACAAATCCAGCTAGTCTACAGCCTGCAGATGGCCTAATCAGGCAGCCCAATGAAAAAGAGCTTGCTGCTCACTCGGTTGCTAAATATGGTGCCCTTGCCCGCCAGAAAATATCCACTGGTAACTACATCACCACCCTTGGTGAAGTTACACGTGGTCACATGGGCGGCGCCAACACTATGTACGCAATTGATGCACCTCCTGAACTTTAA

>PS5*_P8_a14

ATGGAAAGATCAACTTTGATCAATTTACTTCTGTTACACAAATTTGAACACAAGATTAACACTGAAGGAATCATTGTTGTGCACGGAATTGCTGGAACTGGGAAAACCACATTGCTTAGGACTTTATTTTCTGCTTACCCTAGCTTAGTTATAGGTTCACCTAGGCCTTGTTACTTAGATAAAGCTAATAAAATTTCACAAGTTTGCCTTTCTTGTTTTCCAAATACCTTGTGTGACATTGTTGACGAGTACCATCTCTTAGAAAGTTTTCCTGAACCAAAACTAGCCATTTTTGGTGACCCCTGTCAGTGCACTTACATTGAAAGGTTGAGAACACCCAACTACACATCCTTCAGAACACACCGATTTGGCAAATCCACTGCTGCTCTACTAAACAAGTTATTTGATCTTAACATTGAGTCAGTCAAAGCACAAGACGACACAGTAGAATACTTTGATCCTTTCGCAGTGGACCCCTCTGAACACATTTCTGCTTCAGAAAAAGAAGTTTTGGAATTTGTAGGTGATCAAGTTGAGACTACAAGCTCTGAAGAACTAGCTGGTCTCGAGTTTAGTGAAGTTACTTTCTACTGTACCACACTTGCTGGTGCTGTTCAAGAAAATCTTGCCAAAACCTTCATTTCACTCACTAGACACACTTCAAAGCTCACAATTGGTGAACTAAATGCCAGGTCTGACTCCTAGATGCCAGGTCTGACTCCTAGAGCTGATCTTACTGACACGTATAAAATCATTGCTATAGCCTTTCTACTGTCAGCTTGCATTTACTTCCAAAACAGTCATTATCAACCAGTTGCAGGTGATAATTTGCACAGACTACCCTTTGGTGGTCAGTATCAAGACGGAACTAAGAAGATCTCTTACTTTCCGCAGCAACAATCCTACTTTCACTCAGGAAACAAGCTTAATGTCCTCATACTTATCTTCATTCTTACACTGGGTATTGTCCTCACCAATAAATTTAGTTTTAGCATTAGCCGTAATACTCACCAGCATCATTGCTACAACACACATTCTGCAACCCAAACAGGTCAATCAGTGCCAGGTCATCATTGAATGTCCTCATACTTATCTTCATTCTTACACTGGGTATTGTCCTCACCAATAAATTTAGTTTTAGCATTAGCCGTAATACTCACCAGCATCATTGCTACAACACACATTCTGCAACCCAAACAGGTCAATCAGTGCCAGGTCATCATTGATGGTGCAGCCATAGTCATAACAAATTGTCCAAACACACCCGAAGTTCTTAAAGCAATCAACTTCTCCCCTTGGAACGGGTTAAGTTTTACTCAATTGTGAATGGAAAACCAACCTACAGCTTCTAACCCATCAGATGTACCACCAACTGCTGCTCAAGCTGGTGCCCAGAGCCCAGCCGACTTCTCAAATCCTAATACAGCTCCTTCCCTAAGTGATTTGAAGAAGATCAAATACGTGTCAACTGTCACTTCAGTTGCCACGCCTGCTGAAATTGAGGCCCTTGGCAAGATCTTTACTGCCATGGGTTTAGCAGCCAATGAGACCGGACCTGCCATGTGGGACCTCGCTCGTGCTTATGCTGATGTGCAAAGTTCAAAATCTGCACAACTTATAGGTGCCACACCATCCAACCCTGCTTTGTCTAGACGTGCACTTGCTGCACAGTTTGATCGTATCAATATCACACCCAGACAATTCTGCATGTATTTTGCAAAAATTGTTTGGAACATACTGTTAGACAGCAATGTGCCACCTGCCAACTGGGCAAAATTGGGCTATCAGGAAGATACCAAGTTTGCTGCTTTTGACTTCTTTGATGGAGTCACAAATCCAGCTAGTCTACAGCCTGCAGATGGCCTAATCAGGCAGCCCAATGAAAAAGAGCTTGCTGCTCACTCGGTTGCTAAATATGGTGCCCTTGCCCGCCAGAAAATATCCACTGGTAACTACATCACCACCCTTGGTGAAGTTACACGTGGTCACATGGGCGGCGCCAACACTATGTACGCAATTGATGCACCTCCTGAACTTTAA

>PS5*_P8_a13

ATGGAAAGATCAACTTTGATCAATTTACTTCTGTTACACAAATTTGAACACAAGATTAACACTGAAGGAATCATTGTTGTGCACGGAATTGCTGGAACTGGGAAAACCACATTGCTTAGGACTTTATTTTCTGCTTACCCTAGCTTAGTTATAGGTTCACCTAGGCCTTGTTACTTAGATAAAGCTAATAAAATTTCACAAGTTTGCCTTTCTTGTTTTCCAAATACCTTGTGTGACATTGTTGACGAGTACCATCTCTTAGAAAGTTTTCCTGAACCAAAACTAGCCATTTTTGGTGACCCCTGTCAGTGCACTTACATTGAAAGGTTGAGAACACCCAACTACACATCCTTCAGAACACACCGATTTGGCAAATCCACTGCTGCTCTACTAAACAAGTTATTTGATCTTAACATTGAGTCAGTCAAAGCACAAGACGACACAGTAGAATACTTTGATCCTTTCGCAGTGGACCCCTCTGAACACATTTCTGCTTCAGAAAAAGAAGTTTTGGAATTTGTAGGTGATCAAGTTGAGACTACAAGCTCTGAAGAACTAGCTGGTCTCGAGTTTAGTGAAGTTACTTTCTACTGTACCACACTTGCTGGTGCTGTTCAAGAAAATCCTGCCAAAACCTTCATTCCACTCACTAGACACACTTCAAAGCTCACAATTGGTGAACTAAATGCCAGGTCTGACTCCTAGATGCCAGGTCTGACTCCTAGAGCTGATCTTACTGACACGTATAAAATCATTGCTATAGCCTTTCTACTGTCAGCTTGCATTTACTTCCAAAACAGTCATTATCAACCAGTTGCAGGTGATAATTTGCACAGACTACCCTTTGGTGGTCAGTATCAAGACGGAACTAAGAAGATCTCTTACTTTCCGCAGCAACAATCCTACTTTCACTCAGGAAACAAGCTTAATGTCCTCATACTTATCTTCATTCTTACACTGGGTATTGTCCTCACCAATAAATTTAGTTTTAGCATTAGCCGTAATACTCACCAGCATCATTGCTACAACACACATTCTGCAACCCAAACAGGTCAATCAGTGCCAGGTCATCATTGAATGTCCTCATACTTATCTTCATTCTTACACTGGGTATTGTCCTCACCAATAAATTTAGTTTTAGCATTAGCCGTAATACTCACCAGCATCATTGCTACAACACACATTCTGCAACCCAAACAGGTCAATCAGTGCCAGGTCATCATTGATGGTGCAGCCATAGTCATAACAAATTGTCCAAACACACCCGAAGTTCTTAAAGCAATCAACTTCTCCCCTTGGAACGGGTTAAGTTTTACTCAATTGTGAATGGAAAACCAACCTACAGCTTCTAACCCATCAGATGTACCACCAACTGCTGCTCAAGCTGGTGCCCAGAGCCCAGCCGACTTCTCAAATCCTAATACAGCTCCTTCCCTAAGTGATTTGAAGAAGATCAAATACGTGTCAACTGTCACTTCAGTTGCCACGCCTGCTGAAATTGAGGCCCTTGGCAAGATCTTTACTGCCATGGGTTTAGCAGCCAATGAGACCGGACCTGCCATGTGGGACCTCGCTCGTGCTTATGCTGATGTGCAAAGTTCAAAATCTGCACAACTTATAGGTGCCACACCATCCAACCCTGCTTTGTCTAGACGTGCACTTGCTGCACAGTTTGATCGTATCAATATCACACCCAGACAATTCTGCATGTATTTTGCAAAAATTGTTTGGAACATACTGTTAGACAGCAATGTGCCACCTGCCAACTGGGCAAAATTGGGCTATCAGGAAGATACCAAGTTTGCTGCTTTTGACTTCTTTGATGGAGTCACAAATCCAGCTAGTCTACAGCCTGCAGATGGCCTAATCAGGCAGCCCAATGAAAAAGAGCTTGCTGCTCACTCGGTTGCTAAATATGGTGCCCTTGCCCGCCAGAAAATATCCACTGGTAACTACATCACCACCCTTGGTGAAGTTACACGTGGTCACATGGGCGGCGCCAACACTATGTACGCAATTGATGCACCTCCTGAACTTTAA

>PS5*_P8_a12

ATGGAAAGATCAACTTTGATCAATTTACTTCTGTTACACAAATTTGAACACAAGATTAACACTGAAGGAATCATTGTTGTGCACGGAATTGCTGGAACTGGGAAAACCACATTGCTTAGGACTTTATTTTCTGCTTACCCTAGCTTAGTTATAGGTTCACCTAGGCCTTGTTACTTAGATAAAGCTAATAAAATTTCACAAGTTTGCCTTTCTTGTTTTCCAAATACCTTGTGTGACATTGTTGACGAGTACCATCTCTTAGAAAGTTTTCCTGAACCAAAACTAGCCATTTTTGGTGACCCCTGTCAGTGCACTTACATTGAAAGGTTGAGAACACCCAACTACACATCCTTCAGAACACACCGATTTGGCAAATCCACTGCTGCTCTACTAAACAAGTTATTTGATCTTAACATTGAGTCAGTCAAAGCACAAGACGACACAGTAGAATACTTTGATCCTTTCGCAGTGGACCCCTCTGAACACATTTCTGCTTCAGAAAAAGAAGTTTTGGAATTTGTAGGTGATCAAGTTGAGACTACAAGCTCTGAAGAACTAGCTGGTCTCGAGTTTAGTGAAGTTACTTTCTACTGTACCACACTTGCTGGTGCTGTTCAAGAAAATCCTGCCAAAACCTTCATTTCACTCACTAGACACACTTCAAAGCTCACAATTGGTGAACTAAATGCCAGGTCTGACTCCTAGATGCCAGGTCTGACTCCTAGAGCTGACCTTACTGACACGTATAAAATCATTGCTATAGCCTTTCTACTGTCAGCTTGCATTTACTTCCAAAACAGTCATTATCAACCAGTTGCAGGTGATAATTTGCACAGACTACCCTTTGGTGGTCAGTATCAAGACGGAACTAAGAAGATCTCTTACTTTCCGCAGCAACAATCCTACTTTCACTCAGGAAACAAGCTTAATGTCCTCATACTTATCTTCATTCTTACACTGGGTATTGTCCTCACCAATAAATTTAGTTTTAGCATTAGCCGTAATACTCACCAGCATCATTGCTACAACACACATTCTGCAACCCAAACAGGTCAATCAGTGCCAGGTCATCATTGAATGTCCTCATACTTATCTTCATTCTTACACTGGGTATTGTCCTCACCAATAAATTTAGTTTTAGCATTAGCCGTAATACTCACCAGCATCATTGCTACAACACACATTCTGCAACCCAAACAGGTCAATCAGTGCCAGGTCATCATTGATGGTGCAGCCATAGTCATAACAAATTGTCCAAACACACCCGAAGTTCTTAAAGCAATCAACTTCTCCCCTTGGAACGGGTTAAGTTTTACTCAATTGTGAATGGAAAACCAACCTACAGCTTCTAACCCATCAGATGTACCACCAACTGCTGCTCAAGCTGGTGCCCAGAGCCCAGCCGACTTCTCAAATCCTAATACAGCTCCTTCCCTAAGTGATTTGAAGAAGATCAAATACGTGTCAACTGTCACTTCAGTTGCCACGCCTGCTGAAATTGAGGCCCTTGGCAAGATCTTTACTGCCATGGGTTTAGCAGCCAATGAGACCGGACCTGCCATGTGGGACCTCGCTCGTGCTTATGCTGATGTGCAAAGTTCAAAATCTGCACAACTTATAGGTGCCACACCATCCAACCCTGCTTTGTCTAGACGTGCACTTGCTGCACAGTTTGATCGTATCAATATCACACCCAGACAATTCTGCATGTATTTTGCAAAAATTGTTTGGAACATACTGTTAGACAGCAATGTGCCACCTGCCAACTGGGCAAAATTGGGCTATCAGGAAGATACCAAGTTTGCTGCTTTTGACTTCTTTGATGGAGTCACAAATCCAGCTAGTCTACAGCCTGCAGATGGCCTAATCAGGCAGCCCAATGAAAAAGAGCTTGCTGCTCACTCGGTTGCTAAATATGGTGCCCTTGCCCGCCAGAAAATATCCACTGGTAACTACATCACCACCCTTGGTGAAGTTACACGTGGTCACATGGGCGGCGCCAACACTATGTACGCAATTGATGCACCTCCTGAACTTTAA

>PS5*_P8_a11

ATGGAAAGATCAACTTTGATCAATTTACTTCTGTTACACAAATTTGAACACAAGATTAACACTGAAGGAATCATTGTTGTGCACGGAATTGCTGGAACTGGGAAAACCACATTGCTTAGGACTTTATTTTCTGCTTACCCTAGCTTAGTTATAGGTTCACCTAGGCCTTGTTACTTAGATAAAGCTAATAAAATTTCACAAGTTTGCCTTTCTTGTTTTCCAAATACCTTGTGTGACATTGTTGACGAGTACCATCTCTTAGAAAGTTTTCCTGAACCAAAACTAGCCATTTTTGGTGACCCCTGTCAGTGCACTTACATTGAAAGGTTGAGAACACCCAACTACACATCCTTCAGAACACACCGATTTGGCAAATCCACTGCTGCTCTACTAAACAAGTTATTTGATCTTAACATTGAGTCAGTCAAAGCACAAGACGACACAGTAGAATACTTTGATCCTTTCGCAGTGGACCCCTCTGAACACATTTCTGCTTCAGAAAAAGAAGTTTTGGAATTTGTAGGTGATCAAGTTGAGACTACAAGCTCTGAAGAACTAGCTGGTCTCGAGTTTAGTGAAGTTACTTTCTACTGTACCACACTTGCTGGTGCTGTTCAAGAAAATCCTGCCAAAACCTTCATTTCACTCACTAGACACACTTCAAAGCTCACAATTGGTGAACTAAATGCCAGGTCTGACTCCTAGATGCCAGGTCTGACTCCTAGAGCTGATCTTACTGACACGTATAAAATCATTGCTATAGCCTTTCTACTGTCAGCTTGCATTTACTTCCAAAACAGTCATTATCAACCAGTTGCAGGTGATAATTTGCACAGACTACCCTTTGGTGGTCAGTATCAAGACGGAACTAAGAAGATCTCTTACTTTCCGCAGCAACAATCCTACTTTCACTCAGGAAACAAGCTTAATGTCCTCATACTTATCTTCATTCTTACACTGGGTATTGTCCTCACCAATAAATTTAGTTTTAGCATTAGCCGTAATACTCACCAGCATCATTGCTACAACACACATTCTGCAACCCAAACAGGTCAATCAGTGCCAGGTCATCATTGAATGTCCTCATACTTATCTTCATTCTTACACTGGGTATTGTCCTCACCAATAAATTTAGTTTTAGCATTAGCCGTAATACTCACCAGCATCATTGCTACAACACACATTCTGCAACCCAAACAGGTCAATCAGTGCCAGGTCATCATTGATGGTGCAGCCATAGTCATAACAAATTGTCCAAACACACCCGAAGTTCTTAAAGCAATCAACTTCTCCCCTTGGAACGGGTTAAGTTTTACTCAATTGTGAATGGAAAACCAACCTACAGCTTCTAACCCATCAGATGTACCACCAACTGCTGCTCAAGCTGGTGCCCAGAGCCCAGCCGACTTCTCAAATCCTAATACAGCTCCTTCCCTAAGTGATTTGAAGAAGATCAAATACGTGTCAACTGTCACTTCAGTTGCCACGCCTGCTGAAATTGAGGCCCTTGGCAAGATCTTTACTGCCATGGGTTTAGCAGCCAATGAGACCGGACCTGCCATGTGGGACCTCGCTCGTGCTTATGCTGATGTGCAAAGTTCAAAATCTGCACAACTTATAGGTGCCACACCATCCAACCCTGCTTTGTCTAGACGTGCACTTGCTGCACAGTTTGATCGTATCAATATCACACCCAGACAATTCTGCATGTATTTTGCAAAAATTGTTTGGAACATACTGTTAGACAGCAATGTGCCACCTGCCAACTGGGCAAAATTGGGCTATCAGGAAGATACCAAGTTTGCTGCTTTTGACTTCTTTGATGGAGTCACAAATCCAGCTAGTCTACAGCCTGCAGATGGCCTAATCAGGCAGCCCAATGAAAAAGAGCTTGCTGCTCACTCGGTTGCTAAATATGGTGCCCTTGCCCGCCAGAAAATATCCACTGGTAACTACATCACCACCCTTGGTGAAGTTACACGTGGTCACATGGGCGGCGCCAACACTATGTACGCAATTGATGCACCTCCTGAACTTTAA

>PS5*_P8_a10

ATGGAAAGATCAACTTTGATCAATTTACTTCTGTTACACAAATTTGAACACAAGATTAACACTGAAGGAATCATTGTTGTGCACGGAATTGCTGGAACTGGGAAAACCACATTGCTTAGGACTTTATTTTCTGCTTACCCTAGCTTAGTTATAGGTTCACCTAGGCCTTGTTACTTAGATAAAGCTAATAAAATTTCACAAGTTTGCCTTTCTTGTTTTCCAAATACCTTGTGTGACATTGTTGACGAGTACCATCTCTTAGAAAGTTTTCCTGAACCAAAACTAGCCATTTTTGGTGACCCCTGTCAGTGCACTTACATTGAAAGGTTGAGAACACCCAACTACACATCCTTCAGAACACACCGATTTGGCAAATCCACTGCTGCTCTACTAAACAAGTTATTTGATCTTAACATTGAGTCAGTCAAAGCACAAGACGACACAGTAGAATACTTTGATCCTTTCGCAGTGGACCCCTCTGAACACATTTCTGCTTCAGAAAAAGAAGTTTTGGAATTTGTAGGTGATCAAGTTGAGACTACAAGCTCTGAAGAACTAGCTGGTCTCGAGTTTAGTGAAGTTACTTTCTACTGTACCACACTTGCTGGTGCTGTTCAAGAAAATCCTGCCAAAACCTTCATTTCACTCACTAGACACACTTCAAAGCTCACAATTGGTGAACTAAATGCCAGGTCTGACTCCTAGATGCCAGGTCTGACTCCTAGAGCTGATCTTACTGACACGTATAAAATCATTGCTATAGCCTTTCTACTGTCAGCTTGCATTTACTTCCAAAACAGTCATTATCAACCAGTTGCAGGTGATAATTTGCACAGACTACCCTTTGGTGGTCAGTATCAAGACGGAACTAAGAAGATCTCTTACTTTCCGCAGCAACAATCCTACTTTCACTCAGGAAACAAGCTTAATGTCCTCATACTTATCTTCATTCTTACACTGGGTATTGTCCTCACCAATAAATTTAGTTTTAGCATTAGCCGTAATACTCACCAGCATCATTGCTACAACACACATTCTGCAACCCAAACAGGTCAATCAGTGCCAGGTCATCATTGAATGTCCTCATACTTATCTTCATTCTTACACTGGGTATTGTCCTCACCAATAAATTTAGTTTTAGCATTAGCCGTAATACTCACCAGCATCATTGCTACAACACACATTCTGCAACCCAAACAGGTCAATCAGTGCCAGGTCATCATTGATGGTGCAGCCATAGTCATAACAAATTGTCCAAACACACCCGAAGTTCTTAAAGCAATCAACTTCTCCCCTTGGAACGGGTTAAGTTTTACTCAATTGTGAATGGAAAACCAACCTACAGCTTCTAACCCATCAGATGTACCACCAACTGCTGCTCAAGCTGGTGCCCAGAGCCCAGCCGACTTCTCAAATCCTAATACAGCTCCTTCCCTAAGTGATTTGAAGAAGATCAAATACGTGTCAACTGTCACTTCAGTTGCCACGCCTGCTGAAATTGAGGCCCTTGGCAAGATCTTTACTGCCATGGGTTTAGCAGCCAATGAGACCGGACCTGCCATGTGGGACCTCGCTCGTGCTTATGCTGATGTGCAAAGTTCAAAATCTGCACAACTTATAGGTGCCACACCATCCAACCCTGCTTTGTCTAGACGTGCACTTGCTGCACAGTTTGATCGTATCAATATCACACCCAGACAATTCTGCATGTATTTTGCAAAAATTGTTTGGAACATACTGTTAGACAGCAATGTGCCACCTGCCAACTGGGCAAAATTGGGCTATCAGGAAGATACCAAGTTTGCTGCTTTTGACTTCTTTGATGGAGTCACAAATCCAGCTAGTCTACAGCCTGCAGATGGCCTAATCAGGCAGCCCAATGAAAAAGAGCTTGCTGCTCACTCGGTTGCTAAATATGGTGCCCTTGCCCGCCAGAAAATATCCACTGGTAACTACATCACCACCCTTGGTGAAGTTACACGTGGTCACATGGGCGGCGCCAACACTATGTACGCAATTGATGCACCTCCTGAACTTTAA

>PS5*_P8_a9

ATGGAAAGATCAACTTTGATCAATTTACTTCTGTTACACAAATTTGAACACAAGATTAACACTGAAGGAATCATTGTTGTGCACGGAATTGCTGGAACTGGGAAAACCACATTGCTTAGGACTTTATTTTCTGCTTACCCTAGCTTAGTTATAGGTTCACCTAGGCCTTGTTACTTAGATAAAGCTAATAAAATTTCACAAGTTTGCCTTTCTTGTTTTCCAAATACCTTGTGTGACATTGTTGACGAGTACCATCTCTTAGAAAGTTTTCCTGAACCAAAACTAGCCATTTTTGGTGACCCCTGTCAGTGCACTTACATTGAAAGGTTGAGAACACCCAACTACACATCCTTCAGAACACACCGATTTGGCAAATCCACTGCTGCTCTACTAAACAAGTTATTTGATCTTAACATTGAGTCAGTCAAAGCACAAGACGACACAGTAGAATACTTTGATCCTTTCGCAGTGGACCCCTCTGAACACATTTCTGCTTCAGAAAAAGAAGTTTTGGAATTTGTAGGTGATCAAGTTGAGACTACAAGCTCTGAAGAACTAGCTGGTCTCGAGTTTAGTGAAGTTACTTTCTACTGTACCACACTTGCTGGTGCTGTTCAAGAAAATCCTGCCAAAACCTTCATTTCACTCACTAGACACACTTCAAAGCTCACAATTGGTGAACTAAATGCCAGGTCTGACTCCTAGATGCCAGGTCTGACTCCTAGAGCTGATCTTACTGACACGTATAAAATCATTGCTATAGCCTTTCTACTGTCAGCTTGCATTTACTTCCAAAACAGTCATTATCAACCAGTTGCAGGTGATAATTTGCACAGACTACCCTTTGGTGGTCAGTATCAAGACGGAACTAAGAAGATCTCTTACTTTCCGCAGCAACAATCCTACTTTCACTCAGGAAACAAGCTTAATGTCCTCATACTTATCTTCATTCTTACACTGGGTATTGTCCTCACCAATAAATTTAGTTTTAGCATTAGCCGTAATACTCACCAGCATCATTGCTACAACACACATTCTGCAACCCAAACAGGTCAATCAGTGCCAGGTCATCATTGAATGTCCTCATACTTATCTTCATTCTTACACTGGGTATTGTCCTCACCAATAAATTTAGTTTTAGCATTAGCCGTAATACTCACCAGCATCATTGCTACAACACACATTCTGCAACCCAAACAGGTCAATCAGTGCCAGGTCATCATTGATGGTGCAGCCATAGTCATAACAAATTGTCCAAACACACCCGAAGTTCTTAAAGCAATCAACTTCTCCCCTTGGAACGGGTTAAGTTTTCCTCAATTGTGAATGGAAAACCAACCTACAGCTTCTAACCCATCAGATGTACCACCAACTGCTGCTCAAGCTGGTGCCCAGAGCCCAGCCGACTTCTCAAATCCTAATACAGCTCCTTCCCTAAGTGATTTGAAGAAGATCAAATACGTGTCAACTGTCACTTCAGTTGCCACGCCTGCTGAAATTGAGGCCCTTGGCAAGATCTTTACTGCCATGGGTTTAGCAGCCAATGAGACCGGACCTGCCATGTGGGACCTCGCTCGTGCTTATGCTGATGTGCAAAGTTCAAAATCTGCACAACTTATAGGTGCCACACCATCCAACCCTGCTTTGTCTAGACGTGCACTTGCTGCACAGTTTGATCGTATCAATATCACACCCAGACAATTCTGCATGTATTTTGCAAAAATTGTTTGGAACATACTGTTAGACAGCAATGTGCCACCTGCCAACTGGGCAAAATTGGGCTATCAGGAAGATACCAAGTTTGCTGCTTTTGACTTCTTTGATGGAGTCACAAATCCAGCTAGTCTACAGCCTGCAGATGGCCTAATCAGGCAGCCCAATGAAAAAGAGCTTGCTGCTCACTCGGTTGCTAAATATGGTGCCCTTGCCCGCCAGAAAATATCCACTGGTAACTACATCACCACCCTTGGTGAAGTTACACGTGGTCACATGGGCGGCGCCAACACTATGTACGCAATTGATGCACCTCCTGAACTTTAA

>PS5*_P8_a8

ATGGAAAGATCAACTTTGATCAATTTACTTCTGTTACACAAATTTGAACACAAGATTAACACTGAAGGAATCATTGTTGTGCACGGAATTGCTGGAACTGGGAAAACCACATTGCTTAGGACTTTATTTTCTGCTTACCCTAGCTTAGTTATAGGTTCACCTAGGCCTTGTTACTTAGATAAAGCTAATAAAATTTCACAAGTTTGCCTTTCTTGTTTTCCAAATACCTTGTGTGACATTGTTGACGAGTACCATCTCTTAGAAAGTTTTCCTGAACCAAAACTAGCCATTTTTGGTGACCCCTGTCAGTGCACTTACATTGAAAGGTTGAGAACACCCAACTACACATCCTTCAGAACACACCGATTTGGCAAATCCACTGCTGCTCTACTAAACAAGTTATTTGATCTTAACATTGAGTCAGTCAAAGCACAAGACGACACAGTAGAATACTTTGATCCTTTCGCAGTGGACCCCTCTGAACACATTTCTGCTTCAGAAAAAGAAGTTTTGGAATTTGTAGGTGATCAAGTTGAGACTACAAGCTCTGAAGAACTAGCTGGTCTCGAGTTTAGTGAAGTTACTTTCTACTGTACCACACTTGCTGGTGCTGTTCAAGAAAATCCTGCCAAAACCTTCATTTCACTCACTAGACACACTTCAAAGCTCACAATTGGTGAACTAAATGCCAGGTCTGACTCCTAGATGCCAGGTCTGACTCCTAGAGCTGATCTTACTGACACGTATAAAATCATTGCTATAGCCTTTCTACTGTCAGCTTGCATTTACTTCCAAAACAGTCATTATCAACCAGTTGCAGGTGATAATTTGCACAGACTACCCTTTGGTGGTCAGTATCAAGACGGAACTAAGAAGATCTCTTACTTTCCGCAGCAACAATCCTACTTTCACTCAGGAAACAAGCTTAATGTCCTCATACTTATCTTCATTCTTACACTGGGTATTGTCCTCACCAATAAATTTAGTTTTAGCATTAGCCGTAATACTCACCAGCATCATTGCTACAACACACATTCTGCAACCCAAACAGGTCAATCAGTGCCAGGTCATCATTGAATGTCCTCATACTTATCTTCATTCTTACACTGGGTATTGTCCTCACCAATAAATTTAGTTTTAGCATTAGCCGTAATACTCACCAGCATCATTGCTACAACACACATTCTGCAACCCAAACAGGTCAATCAGTGCCAGGTCATCATTGATGGTGCAGCCATAGTCATAACAAATTGTCCAAACACACCCGAAGTTCTTAAAGCAATCAACTTCTCCCCTTGGAACGGGTTAAGTTTTACTCAATTGTGAATGGAAAACCAACCTACAGCTTCTAATCCATCAGATGTACCACCAACTGCTGCTCAAGCTGGTGCCCAGAGCCCAGCCGACTTCTCAAATCCTAATACAGCTCCTTCCCTAAGTGATTTGAAGAAGATCAAATACGTGTCAACTGTCACTTCAGTTGCCACGCCTGCTGAAATTGAGGCCCTTGGCAAGATCTTTACTGCCATGGGTTTAGCAGCCAATGAGACCGGACCTGCCATGTGGGACCTCGCTCGTGCTTATGCTGATGTGCAAAGTTCAAAATCTGCACAACTTATAGGTGCCACACCATCCAACCCTGCTTTGTCTAGACGTGCACTTGCTGCACAGTTTGATCGTATCAATATCACACCCAGACAATTCTGCATGTATTTTGCAAAAATTGTTTGGAACATACTGTTAGACAGCAATGTGCCACCTGCCAACTGGGCAAAATTGGGCTATCAGGAAGATACCAAGTTTGCTGCTTTTGACTTCTTTGATGGAGTCACAAATCCAGCTAGTCTACAGCCTGCAGATGGCCTAATCAGGCAGCCCAATGAAAAAGAGCTTGCTGCTCACTCGGTTGCTAAATATGGTGCCCTTGCCCGCCAGAAAATATCCACTGGTAACTACATCACCACCCTTGGTGAAGTTACACGTGGTCACATGGGCGGCGCCAACACTATGTACGCAATTGATGCACCTCCTGAACTTTAA

>PS5*_P8_a7
[truncated: 103,068 more chars]
