# Supplementary figures and images for: Stable and Broad Spectrum Cross-Protection Against Pepino Mosaic Virus Attained by Mixed Infection
Source: Front Plant Sci. 2018 Dec 6;9:1810. doi: 10.3389/fpls.2018.01810 (PMC6291676; doi:10.3389/fpls.2018.01810)

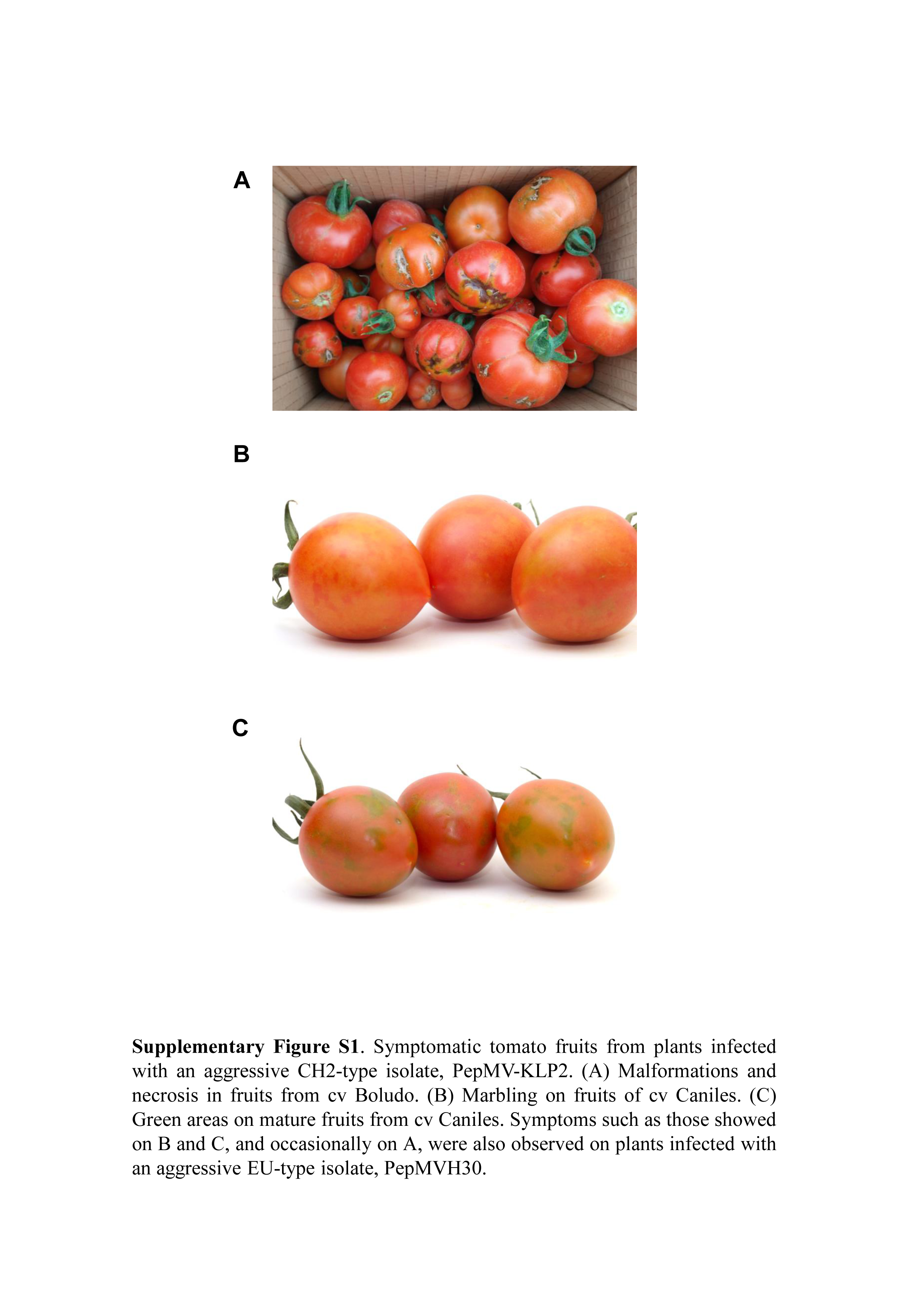

Supplement: Supplementary file 2 [file Image_1.tif]

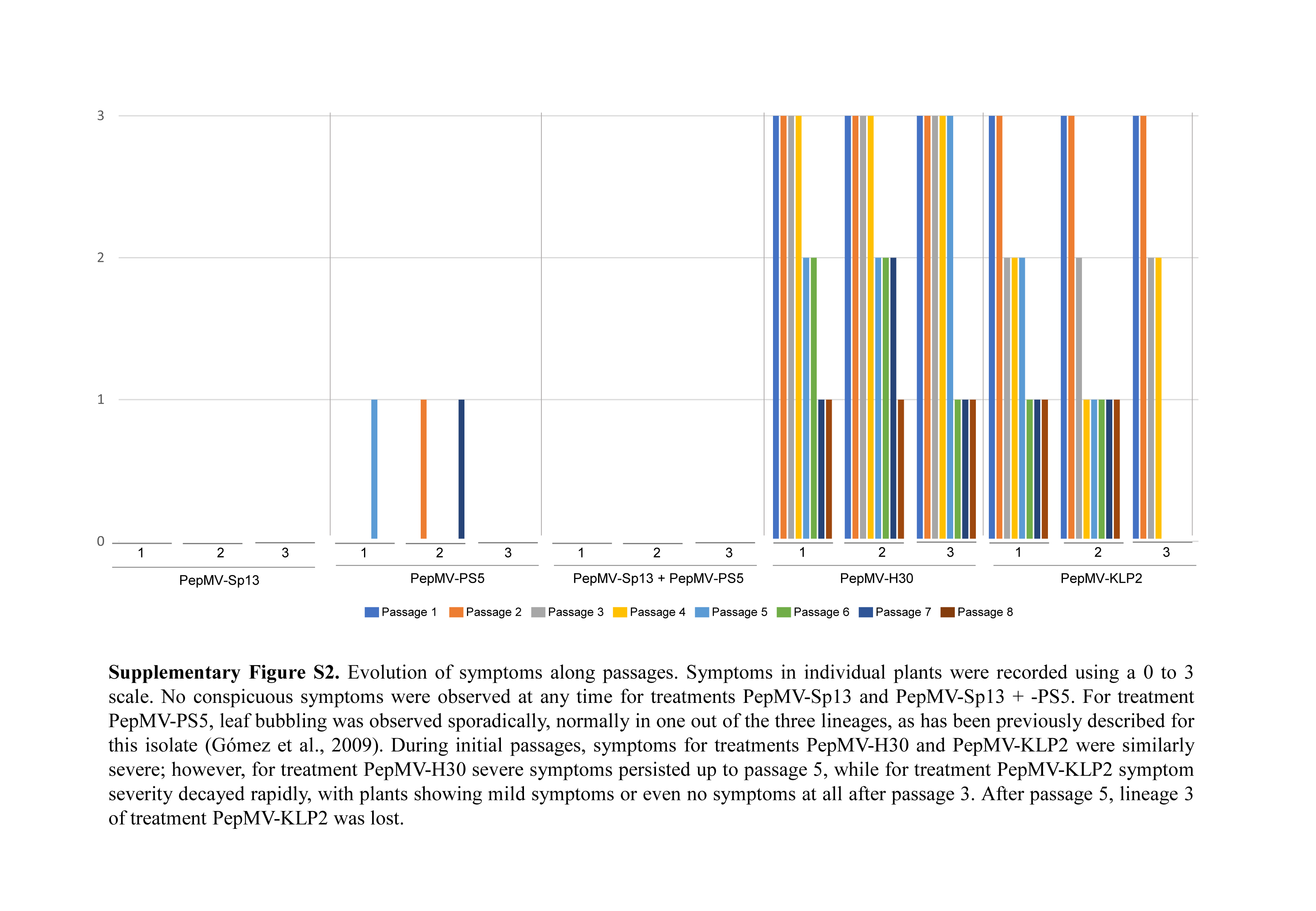

Supplement: Supplementary file 3 [file Image_2.TIF]
